# Systematic classification of vertebrate chemokines based on conserved synteny and evolutionary history

**DOI:** 10.1111/gtc.12013

**Published:** 2012-11-12

**Authors:** Hisayuki Nomiyama, Naoki Osada, Osamu Yoshie

**Affiliations:** 1 Department of Molecular Enzymology, Kumamoto University Graduate School of Medical SciencesHonjo, Kumamoto, 860-8556, Japan; 2 Division of Evolutionary Genetics, Department of Population Genetics, National Institute of GeneticsMishima, Shizuoka, 411-8540, Japan; 3 Department of Microbiology, Kinki University Faculty of MedicineOsaka-Sayama, Osaka, 589-8511, Japan

## Abstract

The genes involved in host defences are known to undergo rapid evolution. Therefore, it is often difficult to assign orthologs in multigene families among various vertebrate species. Chemokines are a large family of small cytokines that orchestrate cell migration in health and disease. Herein, we have surveyed the genomes of 18 representative vertebrate species for chemokine genes and identified a total of 553 genes. We have determined their orthologous relationships and classified them in accordance with the current systematic chemokine nomenclature system. Our study reveals an interesting evolutionary history that gave origin and diversification to the vertebrate chemokine superfamily.

## Introduction

As the number of the genomes that have been sequenced or are in the process of being sequenced increases, it becomes possible to assign orthologous genes between species and this way follow the evolutionary path of a particular gene family. Orthologous genes are descendants of a single gene in the last common ancestor of two species, and this knowledge is critical for understanding their functions in each species. This is particularly important in multigene families where the orthologous relationships are often obscure. Their diversity results from recurrent gene duplications in a given lineage during evolution. Following duplication, one of the “progeny” genes often becomes a pseudogene (Nei & Rooney 2005). Alternatively, one copy acquires a new function, or each copy adopts a portion of the functional role of the parental gene. Thus, the descendent genes in two species may differ substantially in their sequences and copy numbers, further complicating the determination of their orthologous relationships. In some cases, another cause of diversification is whole-genome duplication (WGD). Two rounds of WGD are assumed to have occurred at the base of vertebrate evolution before the divergence of jawless and jawed vertebrates (Dehal & Boore 2005; Kasahara 2007). As a result of such duplications, vertebrate genomes contain closely linked sets of paralogs on more than two, and occasionally four, chromosomes (Dehal & Boore 2005); for example, there are four homeobox (HOX) gene clusters in the human genome. However, among vertebrates, the retained genes vary within each cluster and within each species (Hoegg & Meyer 2005). Furthermore, teleost fish, which account for over half of all known vertebrate species, experienced a teleost-specific third round of WGD before their divergence (Jaillon *et al*. 2004; Meyer & Van de Peer 2005). Thus, teleosts have duplicates of many genes that are present only as single copies in other vertebrate species. Finally, the selective pressures imposed by pathogens may be another potential cause of diversity, and they specially shape the repertoire of host defence proteins. For example, a number of viruses encode viral mimics of host defence proteins to subvert the immune system (Murphy 1993). To counteract this “molecular mimicry” by pathogens, the diversity of host defence proteins may be markedly enhanced, making it more difficult to determine orthologous relationships within gene families.

**Figure 1:**
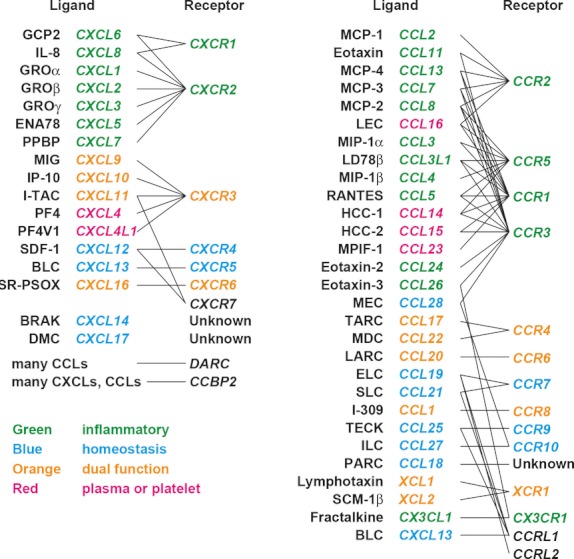
Chemokine ligand–receptor binding relationships. Five subfamilies of chemokines, CXC, CC, XC, CX3C, and CX, have been recognized on the basis of the arrangement of the two N-terminal residues of four conserved cysteines. One and three amino acids separate the first and second cysteines in the CXC and CX3C chemokines, respectively, whereas the two cysteines are adjacent to each other in the CC subfamily. The XC (or C) subfamily lacks the first and paired third cysteine residues. The fifth subfamily, CX, which has so far been identified only in zebrafish, lacks one of the two N-terminal cysteine residues but retains the third and fourth (Nomiyama *et al*. 2008). Chemokines can also be functionally classified into several groups, based on their mode of expression and function (Zlotnik & Yoshie 2000; Moser *et al*. 2004; Mantovani *et al*. 2006). These groups are shown in different colors. Both the common names and systematic nomenclature are shown in the figure. Recently, Islam *et al*. (2011) showed that mouse *Ccl8* serves as an agonist for *Ccr8* but not for *Ccr2*, whereas human *CCL8* binds *CCR2* but not *CCR8*. We previously proposed that the mouse ortholog of human *CCL8* is *Ccl12* rather than *Ccl8* (Nomiyama *et al*. 2003). Thus, mouse *Ccl8* is now likely to be a mouse-specific gene without a human counterpart. Consistent with this, mouse *Ccl12* has been shown to bind *Ccr2*, as does human *CCL8* (Sarafi *et al*. 1997). Therefore, the mouse genes require renaming. The other discrepancies between human and mouse chemokine gene names are described in our previous review (Nomiyama *et al*. 2010). All known chemokine receptors are seven-transmembrane G protein-coupled receptors. Chemokine receptors are classified according to their ability to bind a specific subclass of chemokines (CXCR, CCR, XCR, and CX3CR) (Murphy *et al*. 2000). However, mouse *Cxcr3* and human *CX3CR1* have been shown to bind ligands of a different subclass, mouse *Ccl21* (Soto *et al*. 1998) and human *CCL26* (Nakayama *et al*. 2010), respectively, in addition to their cognate ligands. The receptor(s) for the CX chemokines has not yet been identified. Thus far, 18 signaling chemokine receptors have been identified in the human genome. Besides these classic chemokine receptors, five atypical (nonsignaling) chemokine receptors have been identified (*DARC, CCBP2, CCRL1, CCRL2,* and *CXCR7*) (Graham 2009; Leick *et al*. 2010; Naumann *et al*. 2010). These atypical receptors bind chemokines but do not elicit standard chemotactic responses after ligand binding. Both *DARC* and *CCBP2* primarily bind inflammatory chemokines of the CXC and CC subfamilies. The ligand specificity of the receptors shown here may change by post-translational modification of the ligands (Mortier *et al*. 2008).

Chemokines are a large family of small cytokines (Fig. 1). They were originally described as pro-inflammatory cytokines; however, recent studies indicate that their biological activities reach beyond that category: chemokines also play critical roles in development (Raz & Mahabaleshwar 2009) and homeostasis (Mantovani 1999; Zlotnik & Yoshie 2000; Moser *et al*. 2004; Zlotnik *et al*. 2011; Zlotnik & Yoshie 2012). In the human genome, there are 44 or more chemokine genes, which are a result of some copy number variations (Colobran *et al*. 2010; Nomiyama *et al*. 2010). Chemokine receptors belong to the family of seven-transmembrane G protein-coupled receptors (Murphy *et al*. 2000), and thus far, chemokines and chemokine receptors have only been described in vertebrates (DeVries *et al*. 2006; Bajoghli *et al*. 2009; Nomiyama *et al*. 2011). Interestingly, however, chemokine and chemokine receptor homologues are also found in some viral genomes (Lalani *et al*. 2000), suggesting that some chemokines and chemokine receptors genes have been “hijacked” by viruses to increase their pathogenicity. In our previous study, we surveyed the genome databases of 10 mammalian species for chemokine genes, which revealed their rapid evolution and generation of many lineage-specific chemokines (Nomiyama *et al*. 2010). We carefully determined the orthologous relationships of the chemokine genes among different species and pointed out some errors in the mouse chemokine terminology (Nomiyama *et al*. 2010). Such nomenclature errors may cause considerable confusion in extrapolation of mouse experimental results to humans. In an effort to better understand the evolution of chemokine genes, we have now extended our analysis to a wider group of vertebrate genomes, including some nonmammalian vertebrate species. We have identified a total of 553 vertebrate chemokines and determined their orthologous relationships through synteny conservation and evolutionary history analyses; this has allowed us to classify them into 63 orthologous groups. Previously, we surveyed the genomes of 16 vertebrate species for chemokine receptor genes and determined their orthologous relationships from phylogenetic and comparative genomic analyses (Nomiyama *et al*. 2011). Compared with chemokine genes, however, chemokine receptor genes were found to be relatively well conserved across vertebrate species. Here, to reflect the recent achievements of palaeogenomics in chemokine receptor classification, we have resurveyed the wider vertebrate genome databases for chemokine receptor genes. We have identified 364 chemokine receptor genes, determined their orthologous relationships through the same synteny conservation and evolutionary history analyses. We have classified them into 25 orthologous groups. Our present systematic classifications of vertebrate chemokines and chemokine receptors support the current chemokine and chemokine receptor nomenclature systems and are applicable to other species not included in this study.

## Identification of chemokine and chemokine receptor genes

**Figure 2:**
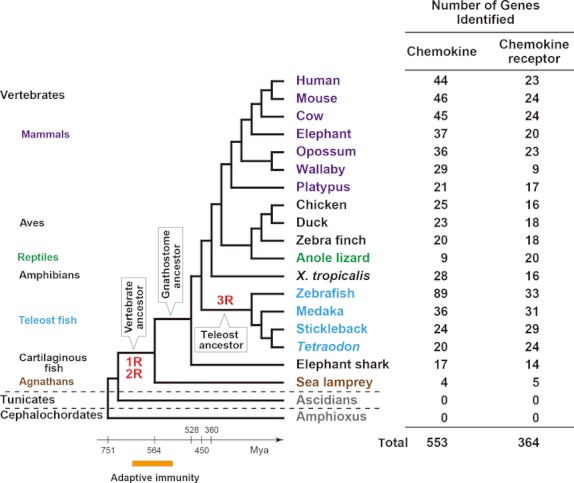
Number of chemokine and chemokine receptor genes identified in vertebrate genomes. We had previously identified chemokine genes from 10 mammalian genomes (Nomiyama *et al*. 2010). Here, we have omitted five genomes from the survey list but added two genomes to cover a wide range of mammals. In total, we searched seven mammalian genomes (human, mouse, cow, elephant, opossum, wallaby, and platypus) for the analyses. In addition, three birds (chicken, zebra finch, and duck), a reptile (anole lizard), an amphibian (*Xenopus*), four teleost fish (medaka, stickleback, zebrafish, and *Tetraodon*), a cartilaginous fish (elephant shark) and a jawless fish (sea lamprey) were included in the survey. Phylogenetic relationships of these organisms among chordates are shown. More detailed taxonomic classifications are shown in Fig. S1 in Supporting Information. The first split in vertebrates occurred between jawed and jawless vertebrates (gnathostomes and agnathans), followed by the divergence of jawed vertebrates into cartilaginous and bony fish (chondrichthyes and osteichthyes). Divergence times (Mya, million years ago) (Hedges & Kumar 2003) are not to scale. A hypothetical origin time for the adaptive immune system is indicated. The timings of the two successive rounds of WGD (1R and 2R) and the teleost-specific WGD (3R) are also shown. Although the timing of the 2R has long been in dispute, Kuraku *et al*. (2009) recently showed that both 1R and 2R occurred before the split between jawed and jawless vertebrates. Recent studies indicate that tunicates (previously known as urochordates) are the invertebrates most closely related to vertebrates (Delsuc *et al*. 2006). The amino acid sequences of the chemokines and their database accession numbers are shown in Fig. S1A in Supporting Information. The chemokine receptor sequences and their accession numbers (Nomiyama *et al*. 2011) have been updated and are shown in Fig. S1B in Supporting Information. Phylogenetic trees of vertebrate chemokines and chemokine receptors are shown in Fig. S4 in Supporting Information.

We searched for chemokine and chemokine receptor genes in vertebrate and invertebrate chordate genomes and replaced or added some organisms to our previous surveys (Nomiyama *et al*. 2010) so that the selected species cover a wider range of vertebrates. The sequences of most of the genomes are still incomplete. Data on the genomes of the elephant shark (cartilaginous fish) and sea lamprey (agnathan) are especially fragmented. However, we have included these genomes because they occupy key positions in the vertebrate evolution. In total, we have identified 553 chemokine genes and 364 chemokine receptor genes from 18 vertebrate species by BLAST searches of genome databases of these species in Ensembl (http://www.ensembl.org) (Fig. 2 and Fig. S1 in Supporting Information). Interestingly, we could not identify any chemokine genes in the genomes of invertebrate chordates such as lancelets (amphioxus) or sea squirts (ascidians).

There are large differences in gene numbers between mammals, birds, and teleosts. Although the genome assemblies are incomplete in most species surveyed, they reflect the lineage-specific expansion and contraction of the chemokine system. In general, mammals (represented by monotremes, marsupials, and placental mammals) and teleost fish have more chemokine and chemokine receptor genes than birds, whose immune gene families are generally much simpler than those of mammals (Dalloul *et al*. 2010). The major reason for the abundance of chemokine genes in mammals is attributable to the presence of large gene clusters (DeVries *et al*. 2006; Colobran *et al*. 2007; Nomiyama *et al*. 2010), whereas birds and teleosts have no such clusters in the corresponding chromosomal regions (Fig. S2 in Supporting Information). The chemokine receptor genes also tend to be clustered in mammals (DeVries *et al*. 2006; Nomiyama *et al*. 2011), although most of these genes arose before the emergence of birds. However, in teleosts, small-scale gene duplications and teleost-specific WGD have led to large increases in both chemokines and chemokine receptors (DeVries *et al*. 2006; Peatman & Liu 2007; Nomiyama *et al*. 2008) (Fig. S3 in Supporting Information).

## Conserved synteny analysis

Phylogenetic analysis is widely used to determine orthologs in multiple genomes. However, this method is insufficient for determining the orthologous relationships of many chemokine genes, because the phylogenetic trees of vertebrate chemokines exhibit many collapsed or poorly supported nodes (Fig. S4 in Supporting Information). This is due in part to the short alignment of chemokine domains used for tree construction and also to gene duplications followed by gene losses or rapid lineage-specific gene expansions. In addition, most genomes are still incomplete, and the orthology assignments can be wrong. To overcome these problems, we also used micro- and macro-conserved synteny analyses. Although the linear order of a genome segment containing a set of genes may have been shuffled considerably during evolution, we are still able to trace conserved synteny even between relatively divergent vertebrate species (Nakatani *et al*. 2007; Catchen *et al*. 2011). Thus, conserved synteny analysis represents a powerful tool for establishing gene orthology between species.

**Figure 3:**
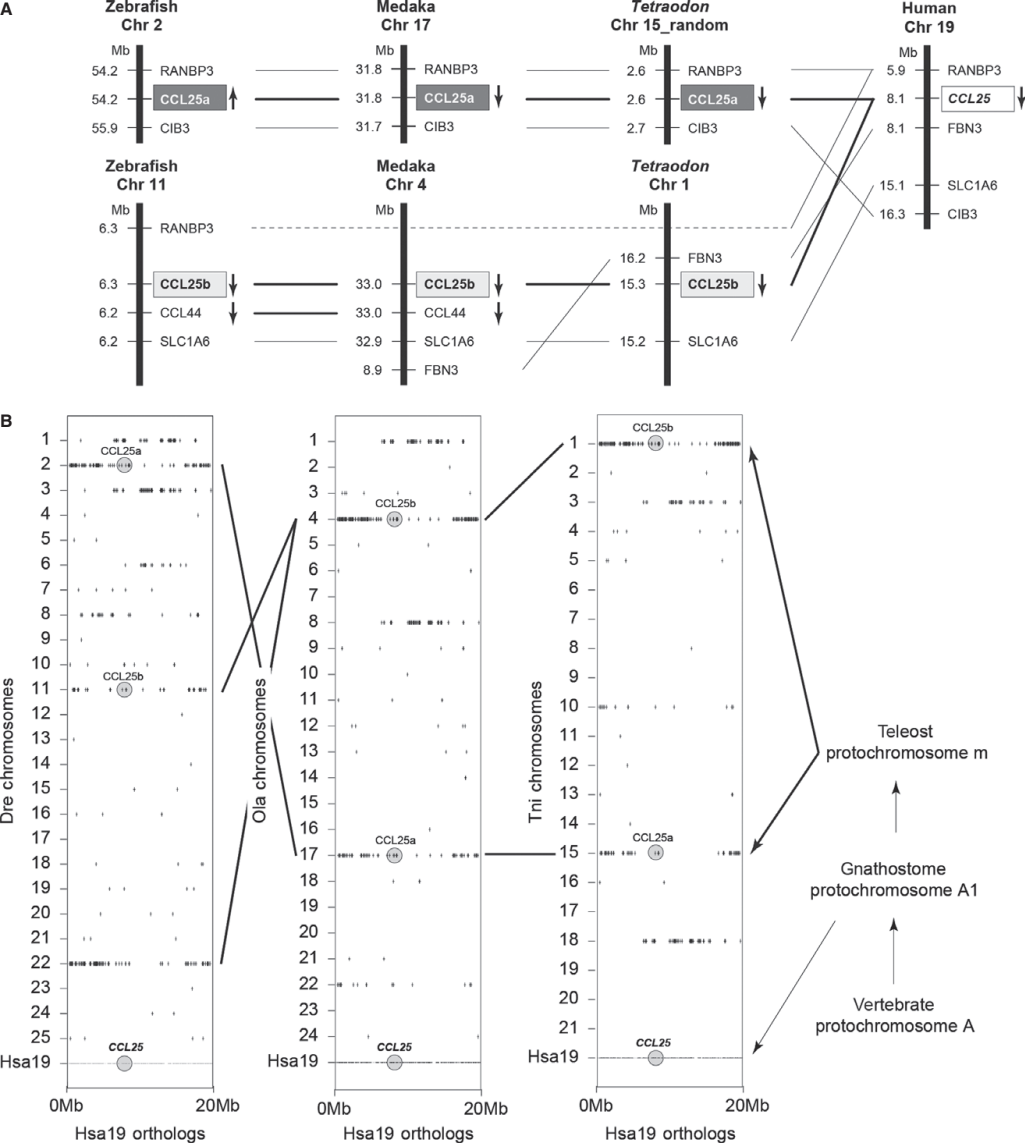
Conserved synteny analysis of vertebrate chemokine *CCL25*. (A) Comparative maps of *CCL25* gene regions. Fish-specific *CCL44* genes are also shown. Arrows indicate transcriptional orientation. Comparative maps of other chemokine and chemokine receptor genes are shown in Fig. S3 in Supporting Information. (B) Conserved synteny dot plots. The plots were drawn using the Synteny Database (Catchen *et al*. 2009, 2011). In the plots, fish orthologs of genes on Hsa19 (0–20 Mb) are indicated as red crosses on fish chromosomes in the order found on the human chromosome (gene orders on fish chromosomes are different from those of humans). Correspondence of the chromosomes among teleosts containing the *CCL25* genes was examined as follows. First, the CVL number was obtained using the human *CCL25* gene locus (chromosome 19, 8.1 Mb) (Nakatani *et al*. 2007). Supplemental Fig. S2 in reference 20 shows that the human genes in this CVL block “88” are orthologous to the genes on medaka chromosomes 4 and 17, where the two medaka *CCL25* genes are located. Next, protochromosome numbers of the teleost, gnathostome, and vertebrate ancestors (m, A1 and A, respectively) were identified from Supplemental Table S2 in reference 20. Using the teleost protochromosome number, the orthologous chromosomes of the three teleosts were then identified in the Supplemental information of reference 25. In case of teleost-specific genes, teleost protochromosome numbers and correspondence of the chromosomes in each teleost can be obtained by consulting the Supplemental information in reference 25. Dre2, Ola17, and Tni15, all containing *CCL25a*, were derived from one of the duplicated chromosomes of teleost protochromosome m. Dre11, Ola4, and Tni1, all containing *CCL25b*, were derived from another duplicated copy of the same protochromosome. Dre11 and Dre22 are the products of chromosome fission. Human and teleost chromosomes containing the *CCL25* regions were all derived from gnathostome protochromosome A1 and also from vertebrate ancestral chromosome A (see also Fig. 4). Synteny dot plots of other chemokine and chemokine receptor genes and the CVL numbers are shown in Fig. S5 and Table S4 in Supporting Information, respectively.

First, we applied conserved synteny analysis to teleost genes, which had expanded by teleost-specific WGD. One such analysis is shown in Fig. 3. *CCL25* is known to play an important role in T-cell differentiation in the thymus and mucosal immunity in the small intestine in human and mouse (Vicari *et al*. 1997; Wurbel *et al*. 2007), but its orthologs have not yet been characterized in teleosts. By using the Ensembl genome data (http://www.ensembl.org), we prepared microsynteny maps covering the genomic regions containing *CCL25* from zebrafish, medaka, *Tetraodon,* and human (Fig. 3A). The maps reveal that each teleost fish contains two orthologs of human *CCL25* located on different chromosomes. The conserved synteny dot plots drawn using Synteny Database (Catchen *et al*. 2009) (http://teleost.cs.uoregon.edu/synteny_db/), which is specifically designed to identify conserved synteny using Ensembl data, show that a large region of human chromosome 19 (Hsa19) surrounding *CCL25* shares a well-recognizable synteny with the *CCL25* regions on two chromosomes of each teleost (Fig. 3B), providing solid evidence for conserved synteny. Recently, ancestral vertebrate genomes have been reconstructed through identification of conserved vertebrate linkage (CVL) blocks in genomes (Kasahara *et al*. 2007; Nakatani *et al*. 2007). CVL blocks are groups of genes located on a single chromosome even after the two rounds of WGD and are similar to the conserved synteny mentioned earlier or the “doubly conserved synteny” (Jaillon *et al*. 2004; Kasahara *et al*. 2007) used for teleosts that experienced the teleost-specific third WGD. Correspondence of the orthologous chromosomes among teleosts can be determined by locating a gene of interest in a specific CVL block. Because ancestral chromosomes are represented by various combinations of CVL blocks, this method also allows us to deduce the evolutionary history of vertebrate genes and has been used successfully for orthology assignments of several genes (Laisney *et al*. 2010; Braasch & Postlethwait 2011). Using this method, we can conclude that the two regions containing fish *CCL25* genes originated from a copy on a preduplication chromosome (protochromosome) “m” of the common teleost ancestor living before the teleost-specific WGD (Fig. 3B). Furthermore, the analysis shows that the *CCL25* regions of medaka chromosome 17 (Ola17), zebrafish chromosome 2 (Dre2), and *Tetraodon* chromosome 15 (Tni15) are derived from the same protochromosome “m”. Therefore, we refer to the *CCL25* genes on these chromosomes as *CCL25a*. Consequently, the *CCL25* genes on medaka chromosome 4 (Ola4), zebrafish chromosome 11 (Dre11), and *Tetraodon* chromosome 1 (Tni1) are referred to as *CCL25b*. Zebrafish chromosome 22 (Dre22) also contains a region derived from the teleost protochromosome “m” after the teleost-specific WGD and subsequent chromosomal fusion and fission, but this region has apparently lost its *CCL25* gene copy.

From similar analyses, we have determined the orthologous relationships of other chemokine genes duplicated by the teleost-specific WGD in the three teleosts (Figs. S3 and S5 in Supporting Information). These assignments may be useful for investigating biological functions, because the functions of co-orthologous genes may have diverged substantially since the teleost-specific WGD, which occurred approximately 350 million years ago (Mya). In zebrafish, for example, the chemokine receptor *CXCR4b* regulates migration of various cell types, including primordial germ cells (David *et al*. 2002; Doitsidou *et al*. 2002; Knaut *et al*. 2003), whereas *CXCR4a* has been shown to mediate endodermal migration (Nair & Schilling 2008). The original *CXCR4* in the teleost ancestor may have had both functions, which may have been separated in the two co-orthologous genes in teleosts.

## Evolutionary history analysis

We next applied the method of evolutionary history analysis to chemokine and chemokine receptor genes to track them back to a protochromosome of a common ancestor of teleosts and amniotes. For this purpose, we used chemokines and chemokine receptors common to teleosts and amniotes, most of which have homeostatic functions. Some chemokines present in only one lineage may have been lost in the other lineage during evolution. We omitted such chemokines from the analysis because they may not have existed at all in the common ancestor of teleosts and amniotes.

**Figure 4:**
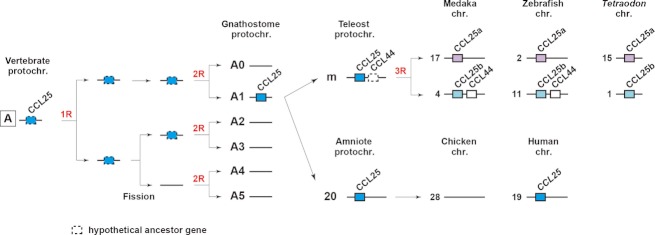
Proposed ancestry of vertebrate chemokine *CCL25*. Vertebrate protochromosome A, on which an ancestral *CCL25* gene is assumed to reside, was duplicated by the 1R and 2R WGDs and also by a fission event between the 1R and 2R, resulting in six gnathostome protochromosomes (Nakatani *et al*. 2007). The *CCL25* gene on gnathostome protochromosome A1 was transferred to teleost and amniote protochromosomes, whereas the genes on the other gnathostome protochromosomes were lost. Fish-specific *CCL44* must have been generated by tandem duplication of the ancestral *CCL25* gene on teleost protochromosome m. Two copies of the teleost *CCL25* gene were maintained on duplicated chromosomes, and one of the *CCL44* copies on one of the duplicated chromosomes may have been lost. *Tetraodon CCL44* and bird *CCL25* genes have not yet been identified. The evolutionary history of other chemokine and chemokine receptor genes are shown in Fig. S6 in Supporting Information. 3R indicates the teleost-specific WGD.

We again use *CCL25* as an example. Fig. 4 shows a deduced evolutionary history of the *CCL25* gene. As mentioned earlier, teleosts contain two *CCL25* genes (*CCL25a* and *CCL25b*), each on a different chromosome. The regions containing these two genes are derived from the protochromosome “m” of the teleost ancestor (Kasahara *et al*. 2007; Nakatani *et al*. 2007). Given that amniotes have a single *CCL25* gene (except birds, where *CCL25* has not been identified), the ancestor of bony vertebrates (including teleosts and amniotes) must have had only a single *CCL25* gene. According to the reconstructed protochromosomes, the *CCL25* ancestral gene is located on protochromosome “A1” of the six protochromosomes of the ancestral gnathostome (jawed vertebrate), the common ancestor of both bony vertebrates and cartilaginous fish. This assumption is strengthened by the existence of a single *CCL25* gene in the elephant shark, a cartilaginous fish that diverged from bony vertebrates shortly after the second round of WGD and thus did not undergo the teleost-specific WGD. The reconstruction also shows that protochromosome “A1” was in turn derived from the vertebrate protochromo-some “A”. However, the presence of a *CCL25* gene on the very first vertebrate protochromosome “A” is only hypothetical. If indeed the gene existed on protochromosome “A”, then the *CCL25* ancestral genes on the other five gnathostome protochromosomes (known as ohnologs (Wolfe 2000)) must have “gone missing”. The ancestral gene of the fish-specific *CCL44*, which is closely linked to *CCL25b* but exhibits low similarity to *CCL25*, may also have been on the teleost protochromosome “m”; but, whether the gnathostome or bony vertebrate protochromosome had *CCL44* is unknown because amniotes do not have this gene. Similarly, we reconstructed the evolutionary history of eight other chemokine genes and 12 chemokine receptor genes that are shared by teleosts and amniotes (Fig. S6 in Supporting Information).

## Nomenclature

As mentioned earlier, we use “a” and “b” to distinguish duplicates such as *CCL25a* and *CCL25b* on the teleost chromosomes originating from the same protochromosome. We add a further suffix “a” or “b” to designate the locally duplicated copies of the co-orthologs; for example, the three tandem *CXCL8* genes on zebrafish chromosome 7 (Dre7) are thus designated *CXCL8ba, CXCL8bb, and CXCL8bc*. According to the zebrafish nomenclature guidelines at the Zebrafish Model Organism Database (ZFIN), these genes should be designated as *CXCL8b.1, CXCL8b.2,* and *CXCL8b.3*, respectively. However, this rule can be applicable only to transcribed genes, and one or two of such co-orthologous genes may still be a pseudogene or the result of sequence editing errors. Therefore, we have not followed this rule here.

The nomenclature of chemokine and chemokine receptor genes of the organisms other than teleosts is basically identical to that in our previous reports (Nomiyama *et al*. 2010, 2011). In Table S1 in Supporting Information, we list the names of the chemokines and chemokine receptors used in previous reports together with those proposed in this article. We should point out that the gene names used by other researchers based exclusively on BLAST searches or percent similarities often differ from the names we propose here, particularly in species other than mammals.

## Classifying the vertebrate chemokines and chemokine receptors

There are closely related sets of chemokines that are apparently the products of recent duplications and thus are very similar to one another. Most of these are species- or lineage-specific chemokines that are tandemly organized in the genomes. In addition, teleost chemokines that were duplicated by teleost-specific WGD are also highly similar to one another. Such chemokines, which are likely to bind the same or closely related receptor(s), can be grouped together. We thus classify the vertebrate chemokines into 63 groups in accordance with the current human chemokine nomenclature system and used “G” to indicate each group (Table 1, and Tables S2 and S3 in Supporting Information). Similarly, we classify the vertebrate chemokine receptors into 25 groups (Nomiyama *et al*. 2011), again using “G” to indicate each group (Table 2). For example, the set of chemokines *CXCL1*, *CXCL2,* and *CXCL3* are grouped to CXCL1/2/3G because they are quite similar to each other, and we cannot distinguish between these genes in species other than human. Likewise, three sets of chemokine receptors, *CXCR1* and *CXCR2*, *CCR1* and *CCR3,* and *CCR2* and *CCR5*, are similar enough to be grouped into CXCR1/2G, CCR1/3G, and CCR2/5G, respectively.

**Table 1:** Vertebrate CXC chemokine gene groups*

Cluster (human)†	Chemokine Group	Human	Mouse	Cow	Elephant	Opossum	Wallaby	Platypus	Chicken	Zebra finch	Duck	Anole lizard	*X. tropicalis*‡	Medaka	Stickleback	Zebrafish	*Tetraodon*§	Elephant shark	Sea lamprey
C	CXCL1/2/3G	3	3	3	1	2	2	2	–	–	–	–	–	–	–	–	–	–	–
C	CXCL4/4LG	2	1	1	1	–	–	–	–	–	–	–	–	–	–	–	–	–	–
C	CXCL5/6G	2	1	1	1	–	–	–	–	–	–	–	–	–	–	–	–	–	–
C	CXCL7G	1	1	1	1	–	–	–	–	–	–	–	–	–	–	–	–	–	–
C	CXCL8G	1	–	1	1	1	–	1	3	2	2	2	5	2	1	4	1	4	1
C	CXCL9G	1	1	1	1	1	–	–	–	–	–	–	–	–	–	–	–	–	–
C	CXCL10G	1	1	1	1	1	1	1	–	–	–	–	3	–	–	–	–	–	–
C	CXCL11G	1	1	1	1	1	–	1	–	–	–	–	–	–	–	8	–	–	–
	CXCL12G	1	1	1	1	1	1	1	1	1	1	1	1	2	2	2	2	2	1
C	CXCL13G	1	1	1	1	–	–	–	3	2	3	2	2	1	1	1	1	1	–
	CXCL14G	1	1	1	1	1	1	1	1	1	1	1	1	1	1	1	1	1	–
	CXCL15G	–	1	1	–	–	–	2	–	–	–	–	–	–	–	–	–	–	1
	CXCL16G¶	1	1	1	1	–	–	–	–	–	–	–	1	–	–	–	–	–	–
	CXCL17G	1	1	1	1	–	–	–	–	–	–	–	–	–	–	–	–	–	–
	CXCL18G	–	–	–	–	–	–	–	–	–	–	–	1	2	2	3	1	–	–
	CXCL19G	–	–	–	–	–	–	–	–	–	–	–	–	1	1	1	1	–	–
	CXCL20G	–	–	–	–	–	–	–	–	–	–	–	–	2	1	1	1	–	–
	CXCL21G	–	–	–	–	–	–	–	–	–	–	–	–	–	–	–	–	–	1
	CXCL22G	–	–	–	–	–	–	–	–	–	–	–	–	–	–	–	–	4	–
	CXCL23G	–	–	–	–	–	–	–	–	–	–	–	–	–	–	–	–	1	–

	Total††	>44	46	45	37	36	29	21	25	20	23	9	28	36	24	89	20	17	4

* Only CXC chemokine gene groups are shown. Other gene groups (CC, CX3C and XC chemokines) are shown in Table S1 in Supporting Information.

† ‘C’ indicates chemokine genes in the human major clusters (see also Fig. 3).

‡ Genes identified in *X. laevis* are included.

§ Genes identified in *Takifugu rubripes* are included.

¶ *X. tropicalis* CXCL16 lacks the amino acid residue between the first and second conserved cysteine residues.

†† Total gene numbers of each species include those of other gene groups (CC, CX3C and XC chemokines).

**Table 2:** Vertebrate chemokine receptor gene groups

Cluster (human)*	Chemokine Receptor Group	Human	Mouse	Cow	Elephant	Opossum	Wallaby	Platypus	Chicken	Zebra finch	Duck	Anole lizard	*X. tropicalis*†	Medaka	Stickleback	Zebrafish	*Tetraodon*‡	Elephant shark	Sea lamprey
	CXCR1/2G	2	2	2	2	2	–	2	1	–	1	2	1	3	3	2	3	2	–
	CXCR3G	1	1	1	1	–	–	–	–	–	–	1	1	4	2	2	2	–	–
	CXCR3LG	–	–	–	–	–	–	–	–	–	–	1	1	1	1	1	1	–	–
	CXCR4G	1	1	1	1	1	1	1	1	1	1	1	2	2	2	2	2	1	1
	CXCR5G	1	1	1	–	1	1	–	1	1	1	1	1	1	1	1	1	1	–
C	CXCR6G	1	1	1	1	1	–	2	–	–	1	1	1	–	–	–	–	1	–
C	CCR1/3G	2	3	3	2	2	1	2	–	–	–	–	–	–	–	–	–	–	–
C	CCR2/5G	2	2	2	2	2	–	2	2	2	2	3	–	–	–	–	–	–	–
C	CCR4G	1	1	1	1	1	1	1	1	1	1	1	–	–	–	–	–	1	–
	CCR4LG	–	–	–	–	–	–	–	–	–	–	–	–	3	2	3	2	–	–
	CCR6G	1	1	1	1	1	–	1	1	1	1	1	1	2	2	2	1	1	–
	CCR7G	1	1	1	1	1	1	–	1	1	1	1	1	1	1	1	1	1	–
C	CCR8G	1	1	1	1	2	–	–	2	3	3	–	1	–	–	–	–	–	–
C	CCR9G	1	1	1	1	1	1	1	1	1	1	1	1	2	2	2	2	1	–
	CCR10G	1	1	1	1	1	1	–	–	–	–	1	1	1	1	1	1	1	–
	CCR11G	–	–	–	–	–	–	–	–	–	–	–	–	2	4	3	1	–	–
	CCR12G	–	–	–	–	–	–	–	–	–	–	–	1	2	4	3	2	–	–
	CCR13G	–	–	–	–	–	–	–	–	–	–	–	–	–	–	–	–	–	2
C	CX3CR1G	1	1	1	1	1	–	1	1	1	1	1	–	–	–	–	–	–	–
C	XCR1G	1	1	1	–	1	–	1	1	1	1	1	1	4	2	6	2	3	–
	CXCR7G	1	1	1	–	1	1	1	1	1	1	1	1	1	–	2	1	1	2
C	CCBP2G	1	1	1	1	1	–	–	1	2	1	–	–	–	–	–	–	–	–
C	CCRL1G	1	1	1	1	1	1	1	1	2	1	1	1	2	2	2	2	–	–
C	CCRL2G	1	1	1	1	1	–	1	–	–	–	–	–	–	–	–	–	–	–
	DARC-G	1	1	1	1	1	–	–	–	–	–	1	–	–	–	–	–	–	–

	Total	23	24	24	20	23	9	17	16	18	18	20	16	31	29	33	24	14	5

* ‘C’ indicates chemokine receptor genes in the human major cluster (see also Fig. 3).

† Genes identified in *X. laevis* are included.

‡ Genes identified in *Takifugu rubripes* are included.

## Gene clusters and binding promiscuity

A prominent feature of the chemokine system is its high degree of promiscuity that allows a single receptor to bind several chemokines and a single chemokine to bind several receptors (Mantovani 1999). It is likely that this ligand–receptor promiscuity, together with redundancy in their actions makes this a highly robust biological system. Among the chemokines, the inflammatory and related plasma/platelet chemokines are the most promiscuous (Fig. 1). Their genes reside within the two major clusters in the genomes of mammals (Fig. S2 in Supporting Information), and consequently, most of them have been generated relatively recently in mammalian evolution (Nomiyama *et al.* 2010). Correspondingly, most of their receptors (*CCR1, 2, 3*, and *5* and *CXCR1* and *2*) also form gene clusters (Nomiyama et al. 2011) (Fig. S2 in Supporting Information). Although human *CXCR3* is not located in these clusters, it may have been within the cluster of the amniote ancestor where *CCR1, 2, 3*, and *5* existed (see Fig. S6 in Supporting Information). As mentioned previously, *CCR1* and *CCR3* are closely related and so are *CCR2* and *CCR5*. They are found only in the lineages leading to mammals and birds. Mammals have all four genes, whereas birds have only two genes, which we tentatively refer to here as bird *CCR2* and *CCR5*. Thus, gene duplications occurred in the mammalian lineage. Their recent duplications coupled with sequence homogenization by gene conversion (Shields 2000; Vazquez-Salat *et al*. 2007) could account for their high promiscuity. In contrast, the origins of *CXCR1*, *CXCR2,* and *CXCR3* are much older than the four *CCRs* (Table 2), dating back to a teleost ancestor or even to a gnathostome ancestor (Fig. S6 in Supporting Information). Nevertheless, except for *CXCL8*, their ligands have been duplicated recently. Thus, each of them has multiple specific ligands. The exceptions are *CXCL6* and *CXCL8*, which are shared by *CXCR1* and *CXCR2*. *CXCR1* and *CXCR2* may have been generated by gene duplication early in the amniote lineage (Fig. S6 in Supporting Information) and may have been homogenized by gene conversion (Shields 2000). It therefore seems logical that they share some of their ligands.

## Functional nonredundancy of the chemokine system

In addition to the binding promiscuity of the chemokine system, the presence of multiple chemokine receptors on a single cell type also causes biologic redundancy, where a single cell responds to many chemokines and, conversely, one chemokine might act on different cell types. After gene duplication, one of the duplicated copies can acquire new roles or functions based on changes in the regulatory regions or coding regions of the gene. When the duplicated copy has different temporal or spatial regulation of gene expression from the other copy, more refined and robust regulation of cell recruitment might be possible. Fig. S7A in Supporting Information illustrates cell recruitment by multiple chemokine, where cells expressing two promiscuous receptors generated by recent gene duplication. In this case, the cells are guided by chemokines, which may bind one or both of the receptors on the cells, expressed at somewhere along the way between the start and final destination. Because the distance between the location of the cells and the site where each chemokine is secreted is short, the gradient of each chemokine can be very steep and the cells can move quickly between the sites. This is in contrast to the case where the cells express only one type of nonpromiscuous receptors. The long distance between the start and final destination makes the chemokine gradient small, making the cells to take time to reach the final destination (Fig. S7B in Supporting Information). Therefore, the former strategy is suitable for coping with acute situations, whereas the latter may be adequate in homeostasis. One example for the former case can be seen in the roles of chemokine receptors CCR2 and CCR5 in West Nile virus (WNV) infection. As described previously, CCR2 and CCR5 have been generated by duplication relatively recently in vertebrate evolution and share some ligands. Infection with WNV causes severe meningitis and encephalitis in a subset of susceptible humans. WNV encephalitis is characterized by infiltration of leukocytes, including monocytes and T cells in the central nervous system (CNS). Although both CCR2 and CCR5 have been shown to control leukocyte recruitment during infection, CCR2 is required for the release of monocytes from bone marrow to blood, while CCR5 is likely involved in the migration of monocytes from circulation to CNS (Lim *et al*. 2006, 2011). Thus, these promiscuous receptors have in fact nonredundant functions *in vivo*, and similar nonredundant and highly cooperative functions are also likely to be played by the promiscuous chemokines.

## Search for missing binding partners

The groups of chemokines and chemokine receptors in our classification may provide a valuable source to search for missing binding partners (Tables 1 and 2, and Table S2 in Supporting Information). Based on the ligand–receptor relationships in human (Fig. 1), which are usually applicable to other animals, some ligands and receptors appear to be missing in some species. This may be due to incomplete genome sequencing in these species. Alternatively, the binding partners in such species may be different from those in humans. One example is *CCR9*, the receptor for *CCL25* in humans. The *CCR9* gene has been identified in all surveyed vertebrate species except for sea lamprey. However, the *CCL25* gene is not found in bird genomes (Table S2 in Supporting Information) and *CCL25* expressed sequence tags (ESTs) have not been identified in birds so far. Therefore, bird *CCR9* may have a different ligand(s) than *CCL25*. Novel bird chemokines in the CCL30, 31 or 41 groups may include the bird *CCR9* ligand(s). Another example is fish *XCR1*. Its ligand, *XCL1*, is missing in fish. Again, the groups CCL32, 33, 34, 35, 36, 40, or 44, most of which have been identified only in fish (Table S2 in Supporting Information), may contain the ligand(s) for fish *XCR1*. Because XC chemokines are more homologous to CC chemokines than to other classes of chemokines, it is not surprising that a CC chemokine(s) binds *XCR1* in fish. In fact, a CC chemokine, *vCCL3*, encoded by Kaposi sarcoma-associated herpes virus is a highly selective and potent agonist of human *XCR1* (Luttichau *et al*. 2007). Thus, a close comparison of the ligand and receptor groups could be informative for locating missing binding partners. Increasing the number of surveyed genomes will further narrow the binding partner candidates.

## Vertebrate ancestral chemokine and chemokine receptor genes

**Figure 5:**
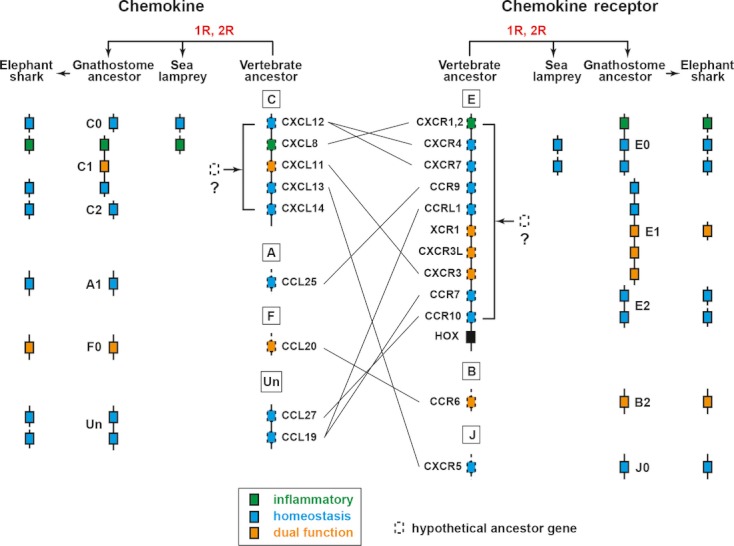
Vertebrate ancestral genes for chemokines and chemokine receptors. The vertebrate and gnathostome protochromosomes on which chemokine and chemokine receptor genes were localized are shown. Among the genes contained by sea lamprey and elephant shark, only those that are shared by vertebrate or gnathostome ancestors are shown. Because the genome sequences of sea lamprey and elephant shark are still fragmented, it is not known whether the genes are linked on the same chromosomes. The lines that link the vertebrate chemokine ancestors with the chemokine receptor ancestors indicate the ligand–receptor relationships based on the human chemokine system. The receptor for *CXCL14* and the ligand(s) for *CXCR3L* have not yet been identified. The ligand(s) for the ancestral *XCR1* is unknown. The predicted locations of HOX gene clusters on the protochromosomes are also indicated. Colocalization of HOX clusters with chemokine receptor genes on vertebrate protochromosome E shows that some of the chemokine receptor genes accompanied HOX cluster duplication (DeVries *et al*. 2006). *HOXD*, *HOXA,* and *HOXB* on gnathostome protochromosomes E0, E1, and E2, respectively, were omitted for simplicity (see also Fig. S6 in Supporting Information). 1R and 2R indicate the two successive rounds of WGD.

The candidates for ancestral chemokine genes are located on two to four vertebrate protochromosomes, of which the total number is assumed to be 10 to 13 (Fig. 5). There are five CXC genes (*CXCL8, 11, 12, 13,* and *14*) on protochromosome “C” and two CC genes, *CCL25* and *CCL20*, on protochromosomes “A” and “F”, respectively. We could not specify the protochromosome(s) that have the ancestral *CCL27* and *CCL19* genes. As for the chemokine receptors, 10 genes (*CXCR1, 3, 3L, 4,* and *7; CCR7, 9,* and *10; XCR1* and *CCRL1*) mapped to protochromosome “E”), whereas *CCR6* and *CXCR5* localized to protochromosomes “B” and “J”, respectively. Obviously, most of the receptors on the protochromosomes have their ligands on protochromosomes, supporting their ancient evolutionary origin and ligand–receptor coevolution (Zlotnik *et al*. 2006) (Fig. 5). However, it is not certain whether all these genes actually existed in the vertebrate ancestor. Among these genes, only two chemokines (*CXCL8* and *CXCL12*) and two chemokine receptors (*CXCR4* and *CXCR7*) have been identified in the agnathan sea lamprey, the oldest extant vertebrate species. Therefore, even after considering the incompleteness of the genome data, it seems likely that only one or a few chemokine ligand–receptor pairs were originally present on the vertebrate protochromosome(s). If this were the case, the protochromosomes carrying the oldest ancestral genes may have been “C” (ligand) and “E” (receptor), where the genes may have expanded by repeated intrachromosomal gene duplications during evolution from the vertebrate ancestor to the gnathostome ancestor. If one of the binding partners does not map to protochromosome “C” or “E,” it may have undergone an interchromosomal translocation; for example, *CCR9* (protochromosome “E”) and its sole ligand *CCL25* (protochromosome “A”). However, *CCL20* and *CCR6*, another specific pair, are located on protochromosomes “F” and “B,” respectively. Although both genes could have translocated from the protochromosomes “C” and “E,” respectively, another possibility is that there were originally two ligand–receptor pairs in the vertebrate ancestor, one pair on protochromosomes “C” and “E” and another pair on different protochromosomes. In that case, the ultimate original pair might be tracked to an invertebrate genome.

## Origin of the chemokine system

Among the genes localized on vertebrate protochromosome “C,” *CXCL8* and *CXCL12* are both found in sea lamprey. Although the receptor for *CXCL8* has not been identified in sea lamprey, the receptors for *CXCL12* (*CXCR4* and *CXCR7*) have been found. Given that *CXCL8* (Oppenheim *et al*. 1991) and *CXCL12* (Nagasawa *et al*. 1996; Raz & Mahabaleshwar 2009) are the prototype inflammatory and homeostatic chemokines, respectively, they may represent the primordial set of chemokines essential for the survival of vertebrate ancestors. Furthermore, most of the chemokine and chemokine receptor genes localized on vertebrate protochromosomes are present in elephant shark (a cartilaginous fish) and are therefore likely to have existed in the genome of the gnathostome ancestor.

The host defence system is composed of two major branches: germ-line-encoded innate immunity and somatically modified adaptive immunity. The chemokines play essential roles in both innate and adaptive immunity (Yoshie *et al*. 2001; Luster 2002; Coelho *et al*. 2005). Although a complex innate immune system is found in every multicellular organism, the immunoglobulin-type adaptive immune system is thought to have emerged before the divergence of gnathostomes (Cooper & Alder 2006) (Fig. 2). The chemokine system also seems to have emerged at about the time when agnathans appeared. However, the sea lamprey has only a few chemokines: *CXCL8* and *CXCL12*, the primordial combination of inflammatory and homeostatic chemokines mentioned earlier, and two other chemokines of unknown function. In contrast, the elephant shark seems to have already acquired the basic sets of chemokines and chemokine receptors that are common in extant vertebrates (Fig. 5). Thus, the basic set of chemokines for vertebrate species may have been established early in the gnathostome lineage, in parallel with the appearance of adaptive immunity.

Although the chemokines and chemokine receptors identified in sea lamprey and elephant shark provide a crucial guide for our argument, their sequenced genomes are still far from complete to enable retrieval of a complete set of genes encoded by these species. Furthermore, as we have previously observed in the *Tetraodon* and Fugu genomes (Nomiyama *et al*. 2008, 2011), some species may have lost a considerable number of chemokine and chemokine receptor genes during evolution. Therefore, we must wait until more genomes of agnathans, cartilaginous fish and sea squirts are sequenced to near completion to draw a more definitive conclusion concerning the origin and evolution of the ancestral chemokine and chemokine receptor genes.

## Concluding remarks

Based on conserved synteny and evolutionary history, we have been able to deduce the orthologous relationships of vertebrate chemokine and chemokine receptor genes. We have classified the vertebrate chemokines and chemokine receptors into 63 and 25 groups, respectively. Our method has proved useful, even for such a large and rapidly evolving gene family such as the chemokine system. Our study has reconstructed the evolutionary history of the chemokine system to a substantial extent, providing a useful platform for understanding this rapidly diversifying multigene family. In particular, the deduced evolutionary history of the duplicated genes among teleosts will greatly facilitate their functional investigation. Our classifications based on the orthologous relationships will be useful for studying chemokines in each species and also for identifying missing binding partners. With an increasing amount of genome data becoming available for vertebrates and invertebrate chordates, it will be of great interest to elucidate the evolutionary histories of various multigene families using the strategy presented in this study.

## Supporting Information/Supplementary Material

**Figure S1** Amino acid sequences of vertebrate chemokines and chemokine receptors.

**Figure S2** Chemokine and chemokine receptor gene clusters.

**Figure S3** Conserved synteny around chemokine and chemokine receptor genes in vertebrates.

**Figure S4** Phylogenetic trees of vertebrate chemokines and chemokine receptors.

**Figure S5** Conserved synteny dot plots of chemokine and chemokine receptor genes.

**Figure S6** Proposed ancestry of vertebrate chemokine and chemokine receptor genes.

**Figure S7** Functional nonredundancy of the chemokine system.

**Table S1** Comparison of the chemokine genes identified in this study with those reported in other studies.

**Table S2** Vertebrate chemokine and chemokine receptor gene groups.

**Table S3** Members of the gene groups of vertebrate chemokines and chemokine receptors.

**Table S4** CVL blocks and ancestral chromosomes containing chemokine and chemokine receptor genes.
