# Supplementary material for: Systematic classification of vertebrate chemokines based on conserved synteny and evolutionary history
Source: Genes Cells. 2012 Nov 12;18(1):1–16. doi: 10.1111/gtc.12013 (PMC3568907; doi:10.1111/gtc.12013)
Supplement: Supplementary file 1 [file gtc0018-0001-SD1.doc]

**Fig. S1** Amino acid sequences of vertebrate chemokines and chemokine receptors.

**A**. Amino acid sequences of vertebrate chemokines in FASTA format.

Conserved cysteine residues are indicated with red letters. Amino acid residues overlapping splice sites are indicated with blue letters. Transmembrane regions are underlined. GenBank or Ensembl accession numbers and the chromosomal numbers are written in the comment lines. ‘Blast search’ indicates novel chemokine genes identified in this study.

Species:

Vertebrata (Subphylum)

Mammalia (mammals)

Prototheria (monotremes)

oan, *Ornithorhynchus anatinus* (platypus)

Theria (live-bearing mammals)

Metatheria (marsupials)

mdo, *Monodelphis domestica* (opossum)

meu, *Macropus eugenii* (wallaby)

Eutheria (placentals)

Afrotheria

laf, *Loxodonta africana* (elephant)

Xenarthra

Supraprimates

hsa, *Homo sapiens* (human)

mus, *Mus musculus* (mouse)

Laurasiatheria

bta, *Bos taurus* (cow)

Aves (birds)

Palaeognathae

Neognathae

Galliformes (fowl)

gga, *Gallus gallus* (chicken)

Passeriformes (passerines)

tgu, *Taeniopygia guttata* (zebra finch)

Anseriformes (waterfowl)

apl, *Anas platyrhynchos* (duck)

Reptilia (reptiles)

aca, *Anolis carolinensis* (anole lizard)

Amphibia (amphibians)

xtr, *Xenopus tropicalis* (Western clawed frog)

xla, *Xenopus laevis* (African clawed frog)

Osteichthyes (bony fishes) (Superclass)

Sarcopterygii (lobe-finned fish) (Class)

Actinopterygii (ray-finned fish) (Class)

Chondrostei (Subclass)

Neopterygii (Subclass)

Holostei (Infraclass)

Teleostei (Infraclass)

Ostariophysi (Superorder)

Cypriniformes (order)

dre, *Danio rerio* (zebrafish)

Acanthopterygii (Superorder)

Beloniformes (order)

ola, *Oryzias latipes* (medaka)

Tetraodontiformes (order)

tni, *Tetraodon nigroviridis* (Tetraodon)

tru, *Takifugu rubripes* (Fugu)

Gasterosteiformes (order)

gac, *Gasterosteus aculeatus* (stickleback)

Chondrichthyes (cartilaginous fish) (Class)

Elasmobranchii (Subclass)

Holocephali (Subclass)

Chimaeriformes (Chimaeras) (order)

cmi, *Callorhinchus milii* (elephant shark)

Agnatha (jawless fish)

Myxini (hagfish)

Petromyzontida

Petromyzontiformes

pma, *Petromyzon marinus* (sea lamprey)

Genome sequence versions:

Human, GRCh37 Feb 2009; Mouse, NCBIM37, Apr 2007; Cow, Btau_4.0 Oct 2007, 7x coverage; Elephant, loxAfr3 Jul 2009, 7x coverage; Opossum, monDom5 Oct 2006, 7.33X coverage; Wallaby, Meug_1.0 Dec 2008, 2x coverage; Platypus, Ornithorhynchus_anatinus-5.0 Dec 2005, 6X coverage

Chicken, WASHUC2 May 2006; Zebra finch, Taeniopygia_guttata-3.2.4 Aug 2008, 58.1d; Duck, duck1 Aug 2009; Anole lizard, AnoCar2.0 May 2010; Xenopus, JGI 4.2 Nov 2009, 62.42

Medaka, HdrR Oct 2005; Stickleback, BROAD S1, Feb 2006; Zebrafish, Zv9 Apr 2010; Tetraodon, TETRAODON 8.0 Mar 2007; Elephant shark, whole genome shotgun sequences9; Sea lamprey, Petromyzon_marinus_7.0, Jan 2011

**(a) Complete sequences**

>hsaCXCL1 NP_001502.1 Chr 4

maraalsaapsnprllrvallllllvaagrraagASVATELRCQCLQTLQGIHPKNIQSVNVKSPGPHCAQTEVIATLKNGRKACLNPASPIVKKIIEKMLNSDKNN

>hsaCXCL2 NP_002080.1 Chr 4

maratlsaapsnprllrvallllllvaasrraagAPLATELRCQCLQTLQGIHLKNIQSVKVKSPGPHCAQTEVIATLKNGQKACLNPASPMVKKIIEKMLKNGKSN

>hsaCXCL3 NP_002081.2 Chr 4

mahatlsaapsnprllrvallllllvaasrraagASVVTELRCQCLQTLQGIHLKNIQSVNVRSPGPHCAQTEVIATLKNGKKACLNPASPMVQKIIEKILNKGSTN

>hsaCXCL4 NP_002610.1 Chr 4

mssaagfcasrpgllflgllllplvvafasaEAEEDGDLQCLCVKTTSQVRPRHITSLEVIKAGPHCPTAQLIATLKNGRKICLDLQAPLYKKIIKKLLES

>hsaCXCL4L1 NP_002611.1 Chr 4

mssaarsrltratrqemlflallllpvvvaFARAEAEEDGDLQCLCVKTTSQVRPRHITSLEVIKAGPHCPTAQLIATLKNGRKICLDLQALLYKKIIKEHLES

>hsaCXCL5 NP_002985.1 Chr 4

msllssraarvpgpssslcallvllllltqpgpiasAGPAAAVLRELRCVCLQTTQGVHPKMISNLQVFAIGPQCSKVEVVASLKNGKEICLDPEAPFLKKVIQKILDGGNKEN

>hsaCXCL6 NP_002984.1 Chr 4

mslpssraarvpgpsgslcallallllltppgplasaGPVSAVLTELRCTCLRVTLRVNPKTIGKLQVFPAGPQCSKVEVVASLKNGKQVCLDPEAPFLKKVIQKILDSGNKKN

>hsaCXCL7 NP_002695.1 Chr 4

mslrldttpscnsarplhalqvllllsllltalaSSTKGQTKRNLAKGKEESLDSDLYAELRCMCIKTTSGIHPKNIQSLEVIGKGTHCNQVEVIATLKDGRKICLDPDAPRIKKIVQKKLAGDESAD

>hsaCXCL8 NP_000575.1 Chr 4

mtsklavallaaflisaalcEGAVLPRSAKELRCQCIKTYSKPFHPKFIKELRVIESGPHCANTEIIVKLSDGRELCLDPKENWVQRVVEKFLKRAENS

>hsaCXCL9 NP_002407.1 Chr 4

mkksgvlfllgiillvligvqgTPVVRKGRCSCISTNQGTIHLQSLKDLKQFAPSPSCEKIEIIATLKNGVQTCLNPDSADVKELIKKWEKQVSQKKKQKNGKKHQKKKVLKVRKSQRSRQKKTT

>hsaCXCL10 NP_001556.2 Chr 4

mnqtailicclifltlsgiqgVPLSRTVRCTCISISNQPVNPRSLEKLEIIPASQFCPRVEIIATMKKKGEKRCLNPESKAIKNLLKAVSKERSKRSP

>hsaCXCL11 NP_005400.1 Chr 4

msvkgmaialavilcatvvqgFPMFKRGRCLCIGPGVKAVKVADIEKASIMYPSNNCDKIEVIITLKENKGQRCLNPKSKQARLIIKKVERKNF

>hsaCXCL12 NP_000600.1 Chr 10

mnakvvvvlvlvltalclsdgKPVSLSYRCPCRFFESHVARANVKHLKILNTPNCALQIVARLKNNNRQVCIDPKLKWIQEYLEKALNKRFKM

>hsaCXCL13 NP_006410.1 Chr 4

mkfistslllmllvsslspvqgVLEVYYTSLRCRCVQESSVFIPRRFIDRIQILPRGNGCPRKEIIVWKKNKSIVCVDPQAEWIQRMMEVLRKRSSSTLPVPVFKRKIP

>hsaCXCL14 NP_004878.2 Chr 5

msllprrappvsmrllaaallllllalytarvdgSKCKCSRKGPKIRYSDVKKLEMKPKYPHCEEKMVIITTKSVSRYRGQEHCLHPKLQSTKRFIKWYNAWNEKRRVYEE

>hsaCXCL16 NP_071342.2 Chr 17

msgsqsevapspqsprspemgrdlrpgsrvlllllllllvyltqpgngNEGSVTGSCYCGKRISSDSPPSVQFMNRLRKHLRAYHRCLYYTRFQLLSWSVCGGNKDPWVQELMSCLDLKECGHAYSGIVAHQKHLLPTSPPISQASEGASSDIHTPAQMLLSTLQSTQRPTLPVGSLSSDKELTRPNETTIHTAGHSLAAGPEAGENQKQPEKNAGPTARTSATVPVLCLLAIIFILTAALSYVLCKRRRGQSPQSSPDLPVHYIPVAPDSNT

>hsaCXCL17 NP_940879.1 Chr 19

mkvlisslllllplmlmsmvsSSLNPGVARGHRDRGQASRRWLQEGGQECECKDWFLRAPRRKFMTVSGLPKKQCPCDHFKGNVKKTRHQRHHRKPNKHSRACQQFLKQCQLRSFALPL

>hsaCCL1 NP_002972.1 Chr 17

mqiittalvclllagmwpedvdsKSMQVPFSRCCFSFAEQEIPLRAILCYRNTSSICSNEGLIFKLKRGKEACALDTVGWVQRHRKMLRHCPSKRK

>hsaCCL2 NP_002973.1 Chr 17

mkvsaallcllliaatfipqglaQPDAINAPVTCCYNFTNRKISVQRLASYRRITSSKCPKEAVIFKTIVAKEICADPKQKWVQDSMDHLDKQTQTPKT

>hsaCCL3 NP_002974.1 Chr 17

mqvstaalavllctmalcnqfsaSLAADTPTACCFSYTSRQIPQNFIADYFETSSQCSKPGVIFLTKRSRQVCADPSEEWVQKYVSDLELSA

>hsaCCL3L1 NP_066286.1 Chr 17

mqvstaalavllctmalcnqvlsAPLAADTPTACCFSYTSRQIPQNFIADYFETSSQCSKPSVIFLTKRGRQVCADPSEEWVQKYVSDLELSA

>hsaCCL3L3 NP_001001437.2 Chr 17

mqvstaalavllctmalcnqvlsAPLAADTPTACCFSYTSRQIPQNFIADYFETSSQCSKPSVIFLTKRGRQVCADPSEEWVQKYVSDLELSA

>hsaCCL4 NP_002975.1 Chr 17

mklcvtvlsllmlvaafcspalsAPMGSDPPTACCFSYTARKLPRNFVVDYYETSSLCSQPAVVFQTKRSKQVCADPSESWVQEYVYDLELN

>hsaCCL4L1 NP_001001435.1 Chr 17

mklcvtvlsllvlvaafcslalsAPMGSDPPTACCFSYTARKLPRNFVVDYYETSSLCSQPAVVFQTKRGKQVCADPSESWVQEYVYDLELN

>hsaCCL4L2 NP_996890.1 Chr 17

mklcvtvlsllvlvaafcslalsAPMGSDPPTACCFSYTARKLPRNFVVDYYETSSLCSQPAVVFQTKRGKQVCADPSESWVQEYVYDLELN

>hsaCCL5 NP_002976.2 Chr 17

mkvsaaalaviliatalcapasaSPYSSDTTPCCFAYIARPLPRAHIKEYFYTSGKCSNPAVVFVTRKNRQVCANPEKKWVREYINSLEMS

>hsaCCL7 NP_006264.2 Chr 17

mkasaallcllltaaafspqglaQPVGINTSTTCCYRFINKKIPKQRLESYRRTTSSHCPREAVIFKTKLDKEICADPTQKWVQDFMKHLDKKTQTPKL

>hsaCCL8 NP_005614.2 Chr 17

mkvsaallclllmaatfspqglaQPDSVSIPITCCFNVINRKIPIQRLESYTRITNIQCPKEAVIFKTKRGKEVCADPKERWVRDSMKHLDQIFQNLKP

>hsaCCL11 NP_002977.1 Chr 17

mkvsaallwllliaaafspqglaGPASVPTTCCFNLANRKIPLQRLESYRRITSGKCPQKAVIFKTKLAKDICADPKKKWVQDSMKYLDQKSPTPKP

>hsaCCL13 NP_005399.1 Chr 17

mkvsavllclllmtaaFNPQGLAQPDALNVPSTCCFTFSSKKISLQRLKSYVITTSRCPQKAVIFRTKLGKEICADPKEKWVQNYMKHLGRKAHTLKT

>hsaCCL14 NP_116739.1 Chr 17

mkisvaaipffllitialgTKTESSSRGPYHPSECCFTYTTYKIPRQRIMDYYETNSQCSKPGIVFITKRGHSVCTNPSDKWVQDYIKDMKEN

>hsaCCL15 NP_116741.1 Chr 17

mkvsvaalsclmlvavlgsqaQFINDAETELMMSKLPLENPVVLNSFHFAADCCTSYISQSIPCSLMKSYFETSSECSKPGVIFLTKKGRQVCAKPSGPGVQDCMKKLKPYSI

>hsaCCL16 NP_004581.1 Chr 17

mkvseaalsllvliliitsasrsQPKVPEWVNTPSTCCLKYYEKVLPRRLVVGYRKALNCHLPAIIFVTKRNREVCTNPNDDWVQEYIKDPNLPLLPTRNLSTVKIITAKNGQPQLLNSQ

>hsaCCL17 NP_002978.1 Chr 16

maplkmlalvtlllgaslqhihaARGTNVGRECCLEYFKGAIPLRKLKTWYQTSEDCSRDAIVFVTVQGRAICSDPNNKRVKNAVKYLQSLERS

>hsaCCL18 NP_002979.1 Chr 17

mkglaaallvlvctmalcscAQVGTNKELCCLVYTSWQIPQKFIVDYSETSPQCPKPGVILLTKRGRQICADPNKKWVQKYISDLKLNA

>hsaCCL19 NP_006265.1 Chr 9

malllalsllvlwtspaptlsGTNDAEDCCLSVTQKPIPGYIVRNFHYLLIKDGCRVPAVVFTTLRGRQLCAPPDQPWVERIIQRLQRTSAKMKRRSS

>hsaCCL20 NP_004582.1 Chr 2

mcctkslllaalmsvlllhlcgeseaASNFDCCLGYTDRILHPKFIVGFTRQLANEGCDINAIIFHTKKKLSVCANPKQTWVKYIVRLLSKKVKNM

>hsaCCL21 NP_002980.1 Chr 9

maqslalsllilvlafgiprtqgSDGGAQDCCLKYSQRKIPAKVVRSYRKQEPSLGCSIPAILFLPRKRSQAELCADPKELWVQQLMQHLDKTPSPQKPAQGCRKDRGASKTGKKGKGSKGCKRTERSQTPKGP

>hsaCCL22 NP_002981.2 Chr 16

mdrlqtallvvlvllavalqateaGPYGANMEDSVCCRDYVRYRLPLRVVKHFYWTSDSCPRPGVVLLTFRDKEICADPRVPWVKMILNKLSQ

>hsaCCL23 NP_665905.1 Chr 17

mkvsvaalsclmlvtalgsqaRVTKDAETEFMMSKLPLENPVLLDRFHATSADCCISYTPRSIPCSLLESYFETNSECSKPGVIFLTKKGRRFCANPSDKQVQVCVRMLKLDTRIKTRKN

>hsaCCL24 NP_002982.2 Chr 24

maglmtivtsllflgvcahhiiptgsVVIPSPCCMFFVSKRIPENRVVSYQLSSRSTCLKAGVIFTTKKGQQFCGDPKQEWVQRYMKNLDAKQKKASPRARAVAVKGPVQRYPGNQTTC

>hsaCCL25 NP_005615.2 Chr 19

mnlwllaclvagflgawapavhtQGVFEDCCLAYHYPIGWAVLRRAWTYRIQEVSGSCNLPAAIFYLPKRHRKVCGNPKSREVQRAMKLLDARNKVFAKLHHNTQTFQAGPHAVKKLSSGNSKLSSSKFSNPISSSKRNVSLLISANSGL

>hsaCCL26 NP_006063.1 Chr 7

mmglslasavllasllslhlgtaTRGSDISKTCCFQYSHKPLPWTWVRSYEFTSNSCSQRAVIFTTKRGKKVCTHPRKKWVQKYISLLKTPKQL

>hsaCCL27 NP_006655.1 Chr 9

mkgpptfcsllllslllspdptaaFLLPPSTACCTQLYRKPLSDKLLRKVIQVELQEADGDCHLQAFVLHLAQRSICIHPQNPSLSQWFEHQERKLHGTLPKLNFGMLRKMG

>hsaCCL28 NP_683513.1 Chr 5

mqqrglaivalavcaalhaseaILPIASSCCTEVSHHISRRLLERVNMCRIQRADGDCDLAAVILHVKRRRICVSPHNHTVKQWMKVQAAKKNGKGNVCHRKKHHGKRNSNRAHQGKHETYGHKTPY

>hsaCX3CL1 NP_002987.1 Chr 16

mapislswllrlatfchltvllagQHHGVTKCNITCSKMTSKIPVALLIHYQQNQASCGKRAIILETRQHRLFCADPKEQWVKDAMQHLDRQAAALTRNGGTFEKQIGEVKPRTTPAAGGMDESVVLEPEATGESSSLEPTPSSQEAQRALGTSPELPTGVTGSSGTRLPPTPKAQDGGPVGTELFRVPPVSTAATWQSSAPHQPGPSLWAEAKTSEAPSTQDPSTQASTASSPAPEENAPSEGQRVWGQGQSPRPENSLEREEMGPVPAHTDAFQDWGPGSMAHVSVVPVSSEGTPSREPVASGSWTPKAEEPIHATMDPQRLGVLITPVPDAQAATRRQAVGLLAFLGLLFCLGVAMFTYQSLQGCPRKMAGEMAEGLRYIPRSCGSNSYVLVPV

>hsaXCL1 NP_002986.1 Chr 1

mrllilallgicsltayivegVGSEVSDKRTCVSLTTQRLPVSRIKTYTITEGSLRAVIFITKRGLKVCADPQATWVRDVVRSMDRKSNTRNNMIQTKPTGTQQSTNTAVTLTG

>hsaXCL2 NP_003166.1 Chr 1

mrllilallgicsltayivegVGSEVSHRRTCVSLTTQRLPVSRIKTYTITEGSLRAVIFITKRGLKVCADPQATWVRDVVRSMDRKSNTRNNMIQTKPTGTQQSTNTAVTLTG

>musCXCL1 NP_032202.1 Chr 5

mipatrsllcaallllatsrlatgAPIANELRCQCLQTMAGIHLKNIQSLKVLPSGPHCTQTEVIATLKNGREACLDPEAPLVQKIVQKMLKGVPK

>musCXCL2 NP_033166.1 Chr 5

mapptcrllsaalvlllllatnhqatgAVVASELRCQCLKTLPRVDFKNIQSLSVTPPGPHCAQTEVIATLKGGQKVCLDPEAPLVQKIIQKILNKGKAN

>musCXCL3 NP_976065.1 Chr 5

mapptcrllsaalvlllllatnhqatgAVVASELRCQCLNTLPRVDFETIQSLTVTPPGPHCTQTEVIATLKDGQEVCLNPQGPRLQIIIKKILKSGKSS

>musCXCL4L1 NP_064316.1 Chr 5

msvaavfrglrpspellllgllflpavvaVTSAGPEESDGDLSCVCVKTISSGIHLKHITSLEVIKAGRHCAVPQLIATLKNGRKICLDRQAPLYKKVIKKILES

>musCXCL6 NP_033167.2 Chr 5

mslqlrssaripsgsispfmrmaplaflllftlpqhlaeaAPSSVIAATELRCVCLTVTPKINPKLIANLEVIPAGPQCPTVEVIAKLKNQKEVCLDPEAPVIKKIIQKILGSDKKKAKRNALAVERTASVQ

>musCXCL7b NP_076274.1 Chr 5

mgfrlrptssctracplhnlqillllglilvalapltagKSDGMDPYIELRCRCTNTISGIPFNSISLVNVYRPGVHCADVEVIATLKNGQKTCLDPNAPGVKRIVMKILEGY

>musCXCL9 NP_032625.2 Chr 5

mksavlfllgiifleqcgvrgTLVIRNARCSCISTSRGTIHYKSLKDLKQFAPSPNCNKTEIIATLKNGDQTCLDPDSANVKKLMKEWEKKISQKKKQKRGKKHQKNMKNRKPKTPQSRRRSRKTT

>musCXCL10 NP_067249.1 Chr 5

mnpsaavifclillglsgtqgIPLARTVRCNCIHIDDGPVRMRAIGKLEIIPASLSCPRVEIIATMKKNDEQRCLNPESKTIKNLMKAFSQKRSKRAP

>musCXCL11 NP_062367.1 Chr 5

mnrkvtaialaaiwataaqgFLMFKQGRCLCIGPGMKAVKMAEIEKASVIYPSNGCDKVEVIVTMKAHKRQRCLDPRSKQARLIMQAIEKKNFLRRQNM

>musCXCL12 NP_001012495.1 Chr 6

MdakvvavlalvlaalcisdgKPVSLSYRCPCRFFESHIARANVKHLKILNTPNCALQIVARLKNNNRQVCIDPKLKWIQEYLEKALNKGRREEKVGKKEKIGKKKRQKKRKAAQKRKN

>musCXCL13 NP_061354.1 Chr 5

mrlstatlllllasclspghgILEAHYTNLKCRCSGVISTVVGLNIIDRIQVTPPGNGCPKTEVVIWTKMKKVICVNPRAKWLQRLLRHVQSKSLSSTPQAPVSKRRAA

>musCXCL14 NP_062514.2 Chr 13

mrllaaallllllalcasrvdgSKCKCSRKGPKIRYSDVKKLEMKPKYPHCEEKMVIVTTKSMSRYRGQEHCLHPKLQSTKRFIKWYNAWNEKRRVYEE

>musCXCL15 NP_035469.1 Chr 5

maaqgwsmlllavlnlgifvrpcdtQELRCLCIQEHSEFIPLKLIKNIMVIFETIYCNRKEVIAVPKNGSMICLDPDAPWVKATVGPITNRFLPEDLKQKEFPPAMKLLYSVEHEKPLYLSFGRPENKRIFPFPIRETSRHFADLAHNSDRNFLRDSSEVSLTGSDA

>musCXCL16 NP_075647.3 Chr 11

mrrgfgplslafflfllalltlpgdgNQGSVAGSCSCDRTISSGTQIPQGTLDHIRKYLKAFHRCPFFIRFQLQSKSVCGGSQDQWVRELVDCFERKECGTGHGKSFHHQKHLPQASTQTPEAAEGTPSDTSTPAHSQSTQHSTLPSGALSLNKEHTQPWEMTTLPSGYGLEARPEAEANEKQQDDRQQEAPGAGASTPAWVPVLSLLAIVFFLTAAMAYVLCNRRATQQNSAGLQLWYTPVEPRP

>musCXCL17 NP_705804.2 Chr 7

mkllaspfllllpvmlmsmvfsSPNPGVARSHGDQHLAPRRWLLEGGQECECKDWFLQAPKRKATAVLGPPRKQCPCDHVKGREKKNRHQKHHRKSQRPSRACQQFLKRCHLASFALPL

>musCCL1 NP_035459.1 Chr 11

mkptamalmclllaavwiqdvdsKSMLTVSNSCCLNTLKKELPLKFIQCYRKMGSSCPDPPAVVFRLNKGRESCASTNKTWVQNHLKKVNPC

>musCCL2 NP_035463.1 Chr 11

mqvpvmllgllftvagwsihvlaQPDAVNAPLTCCYSFTSKMIPMSRLESYKRITSSRCPKEAVVFVTKLKREVCADPKKEWVQTYIKNLDRNQMRSEPTTLFKTASALRSSAPLNVKLTRKSEANASTTFSTTTSSTSVGVTSVTVN

>musCCL3 NP_035467.1 Chr 11

mkvsttalavllctmtlcnqvfsAPYGADTPTACCFSYSRKIPRQFIVDYFETSSLCSQPGVIFLTKRNRQICADSKETWVQEYITDLELNA

>musCCL4 NP_038680.1 Chr 11

mklcvsalsllllvaafcapgfsAPMGSDPPTSCCFSYTSRQLHRSFVMDYYETSSLCSKPAVVFLTKRGRQICANPSEPWVTEYMSDLELN

>musCCL5 NP_038681.2 Chr 11

mkisaaaltiiltaaalctpapaSPYGSDTTPCCFAYLSLALPRAHVKEYFYTSSKCSNLAVVFVTRRNRQVCANPEKKWVQEYINYLEMS

>musCCL6 NP_033165.1 Chr 11

mrnsktaisffilvavlgsqaGLIQGFQDTSSDCCFSYATQIPCKRFIYYFPTSGGCIKPGIIFISRRGTQVCADPSDRRVQRCLSTLKQGPRSGNKVIA

>musCCL7 NP_038682.1 Chr 11

mrisatllcllliaaafsiqvwaQPDGPNASTCCYVKKQKIPKRNLKSYRRITSSRCPWEAVIFKTKKGMEVCAEAHQKWVEEAIAYLDMKTPTPKP

>musCCL8 NP_067418.1 Chr 11

mkiyavllcllliavpvspEKLTGPDKAPVTCCFHVLKLKIPLRVLKSYERINNIQCPMEAVVFQTKQGMSLCVDPTQKWVSEYMEILDQKSQILQP

>musCCL9 NP_035468.1 Chr 11

mkpfhtalsflilttalgiwaQITHGFQDSSDCCLSYNSRIQCSRFIGYFPTSGGCTRPGIIFISKRGFQVCANPSDRRVQRCIERLEQNSQPRTYKQ

>musCCL11 NP_035460.1 Chr 11

mqsstallfllltvtsftsqvlaHPGSIPTSCCFIMTSKKIPNTLLKSYKRITNNRCTLKAIVFKTRLGKEICADPKKKWVQDATKHLDQKLQTPKP

>musCCL12 NP_035461.2 Chr 11

mkistllcllliattispqvlaGPDAVSTPVTCCYNVVKQKIHVRKLKSYRRITSSQCPREAVIFRTILDKEICADPKEKWVKNSINHLDKTSQTFILEPSCLG

>musCCL17 NP_035462.2 Chr 8

mktftsafgtmrslqmlllaalllgtflqharaARATNVGRECCLDYFKGAIPIRKLVSWYKTSVECSRDAIVFLTVQGKLICADPKDKHVKKAIRLVKNPRP

>musCCL19a NP_036018.1 Chr 4

maprvtpllafsllvlwtfpaptlgGANDAEDCCLSVTQRPIPGNIVKAFRYLLNEDGCRVPAVVFTTLRGYQLCAPPDQPWVDRIIRRLKKSSAKNKGNSTRRSPVS

>musCCL19b XP_001472218.1 Chr 4

maprvtpllafsllvlwtfpaptlgGANDAEDCCLSVTQRPIPGNIVKAFRYLLNEDGCRVPAVVFTTLRGYQLCAPPDQPWVDRIIRRLKKSSAKNKGNSTRRSPVS

>musCCL20 NP_058656.1 Chr 1

macggkrllflalawvllahlcsqaeaASNYDCCLSYIQTPLPSRAIVGFTRQMADEACDINAIIFHTKKRKSVCADPKQNWVKRAVNLLSLRVKKM

>musCCL21a NP_035254.1 Chr 4

maqmmtlsllslvlalcipwtqgSDGGGQDCCLKYSQKKIPYSIVRGYRKQEPSLGCPIPAILFSPRKHSKPELCANPEEGWVQNLMRRLDQPPAPGKQSPGCRKNRGTSKSGKKGKGSKGCKRTEQTQPSRG

>musCCL21b NP_035465.2 Chr 4

maqmmtlsllslvlalcipwtqgSDGGGQDCCLKYSQKKIPYSIVRGYRKQEPSLGCPIPAILFLPRKHSKPELCANPEEGWVQNLMRRLDQPPAPGKQSPGCRKNRGTSKSGKKGKGSKGCKRTEQTQPSRG

>musCCL21c NP_075539.1 Chr 4

maqmmtlsllslvlalcipwtqgSDGGGQDCCLKYSQKKIPYSIVRGYRKQEPSLGCPIPAILFLPRKHSKPELCANPEEGWVQNLMRRLDQPPAPGKQSPGCRKNRGTSKSGKKGKGSKGCKRTEQTQPSRG

>musCCL21d NP_001180595.1 Chr 4

maqmmtlsllslvlalcipwtqgSDGGGQDCCLKYSQKKIPYSIVRGYRKQEPSLGCPIPAILFLPRKHSKPELCANPEEGWVQNLMRRLDQPPAPGKQSPGCRKNRGTSKSGKKGKGSKGCKRTEQTQPSRG

>musCCL21e NP_001180597.1 Chr 4

maqmmtlsllslvlalcipwtqgSDGGGQDCCLKYSQKKIPYSIVRGYRKQEPSLGCPIPAILFLPRKHSKPELCANPEEGWVQNLMRRLDQPPAPGKQSPGCRKNRGTSKSGKKGKGSKGCKRTEQTQPSRG

>musCCL22 NP_033163.1 Chr 8

matlrvpllvalvllavaiqtsdaGPYGANVEDSICCQDYIRHPLPSRLVKEFFWTSKSCRKPGVVLITVKNRDICADPRQVWVKKLLHKLS

>musCCL24 NP_062523.1 Chr 5

magsativagllllvacaccifpidsVTIPSSCCTSFISKKIPENRVVSYQLANGSICPKAGVIFITKKGHKICTDPKLLWVQRHIQKLDAKKNQPSKGAKAVRTKFAVQRRRGNSTEV

>musCCL25 NP_033164.1 Chr 8

mklwlfaclvacfvgawmpvvhaQGAFEDCCLGYQHRIKWNVLRHARNYHQQEVSGSCNLRAVRFYFRQKVVCGNPEDMNVKRAMRILTARKRLVHWKSASDSQTERKKSNHMKSKVENPNSTSVRSATLGHPRMVMMPRKTNN

>musCCL26 NP_001013430.2 Chr 5

mffdlgllvllaiflsvqlgvaTCGSSIAMSCCPNFSYYVIPWSWVYSYKFTDKSCTSDGVIFFTKTGKQFCVQPGAKWVQRFISLVNTRNHL

>musCCL27a NP_035466.1 Chr 4

mmeglspasslpllllllspapeaaLPLPSSTSCCTQLYRQPLPSRLLRRIVHMELQEADGDCHLQAVVLHLARRSVCVHPQNRSLARWLERQGKRLQGTVPSLNLVLQKKMYSNPQQQN

>musCCL27b NP_001186888.1 Chr 4

mmeglspasslpllllllspapeaaLPLPSSTSCCTQLYRQPLPSRLLRRIVHMELQEADGDCHLQAVVLHLARRSVCVHPQNRSLARWLERQGKRLQGTVPSLNLVLQKKMYSHPQQQN

>musCCL27c NP_001157518.1 Chr 4

mmeglspasslpllllllspapeaaLPLPSSTSCCTQLYRQPLPSRLLRRIVHMELQEADGDCHLQAVVLHLARRSVCVHPQNRSLARWLERQGKRLQGTVPSLNLVLQKKMYSHPQQQN

>musCCL27d NP_001186894.1 Chr 4

mmeglspasslpllllllspapeaaLPLPSSTSCCTQLYRQPLPSRLLRRIVHMELQEADGDCHLQAVVLHLARRSVCVHPQNRSLARWLERQGKRLQGTVPSLNLVLQKKMYSHPQQQN

>musCCL28 NP_064675.1 Chr 13

mqqagltlmavavcvafqtseaILPMASSCCTEVSHHVSGRLLERVSSCSIQRADGDCDLAAVILHVKRRRICISPHNRTLKQWMRASEVKKNGRENVCSGKKQPSRKDRKGHTTRKHRTRGTHRHEASR

>musCX3CL1 NP_033168.2 Chr 8

mapsplawllrlaaffhlctllpgQHLGMTKCEIMCDKMTSRIPVALLIRYQLNQESCGKRAIVLETTQHRRFCADPKEKWVQDAMKHLDHQAAALTKNGGKFEKRVDNVTPGITLATRGLSPSALTKPESATLEDLALELTTISQEARGTMGTSQEPPAAVTGSSLSTSEAQDAGLTAKPQSIGSFEAADISTTVWPSPAVYQSGSSSWAEEKATESPSTTAPSPQVSTTSPSTPEENVGSEGQPPWVQGQDLSPEKSLGSEEINPVHTDNFQERGPGNTVHPSVAPISSEETPSPELVASGSQAPKIEEPIHATADPQKLSVLITPVPDTQAATRRQAVGLLAFLGLLFCLGVAMFAYQSLQGCPRKMAGEMVEGLRYVPRSCGSNSYVLVPV

>musXCL1 NP_032536.1 Chr 1

mrlllltflgvccltpwvvegVGTEVLEESSCVNLQTQRLPVQKIKTYIIWEGAMRAVIFVTKRGLKICADPEAKWVKAAIKTVDGRASTRKNMAETVPTGAQRSTSTAITLTG

>btaCXCL1 NP_776724.1 Chr 6

mapaassaprllraamlllllvaagrraagAPVVNELRCQCLQTLQGIHLKNIQSVKVTTPGPHCDQTEVIATLKTGQEVCLNPAAPMVKKIIDKMLN

>btaCXCL2 NP_001041630.1 Chr 6

mapaataaaprllraamlllllvaagrraagAPVVNELRCQCLQTLQGIHLKNIQSVKVTTPGPHCDQTEVIASLKTGQEVCLNPTAPMVKKIIDKMLNKASAN

>btaCXCL3 NP_001039978.1 Chr 6

maraataaaprllraamlllllvaagrraagAPVVNELRCHCLQTLQGIHLKNIQSVKVTPPGPHCGQTEVIATLKNGQEACLNPEAPMVKKIINKMLNKGSTN

>btaCXCL4L1 NP_001094532.1 Chr 6

mnqavgprasrprsspgllllgllllpaialaQESSFPATFVPLPADSEGGESEDLQCVCLKTTSGINPRHISSLEVIGAGLHCPSPQLIATLKTGRKICLDQQNPLYKKIIKRLLKS

>btaCXCL6 NP_776725.1 Chr 6

mrllssraarvsgpsgslcallalllltppgplasAGPVAAVVRELRCVCLTTTPGIHPKTVSDLQVIAAGPQCSKVEVIATLKNGREVCLDPEAPLIKKIVQKILDSGKNN

>btaCXCL7b XP_869864.1 Chr 6

mslrpdatssytvpsplpvlqvlllmslllvmlvpstngKLSSKERFLHIELRCLCVKTISGIPSSNIQSLEVNRAGPHCNKVEVIAELKNGKKICLNPEGPRIKKIVQKILEDGGLAA

>btaCXCL8 NP_776350.1 Chr 6

mtsklavallaafllsaalceaAVLSRMSTELRCQCIKTHSTPFHPKFIKELRVIESGPHCENSEIIVKLTNGNEVCLNPKEKWVQKVVQVFVKRAEKQDP

>btaCXCL9 NP_001106643.1 Chr 6

mkksaplflgiifltltgvqgVPAIRNGRCSCINTSQGMIHPKSLKDLKQFAPSPSCEKTEIIATMKNGNEACLNPDLPEVKELIKEWEKQVNQKKKQRKGKKYKKTKKVPKVKRSQRPSQKKTT

>btaCXCL10 NP_001040016.1 Chr 6

mnksgflifclilltlsqgVPLSRNTRCSCIEISNGSVNPRSLEKLEVIPASQSCPRVEIIATMKKNGEKRCLNPESKTIKNLLKAINKQRTKRSPRTRKEA

>btaCXCL11 NP_001106644.1 Chr 6

msvkgmaivltvilcaaivqgFPMFKGGRCLCIGPGVKAVKVADIEKVSIIYPTNNCDKTEVIITLKTHKGQRCLNPKAKQAKAIIKKVQRKNSEKYKNI

>btaCXCL12 NP_001106645.1 Chr 28

mdakvfvvlalvltalclsdaKPVSLSYRCPCRFFESHVAKANVKHLKILNTPNCSLQIVARLKNNNRQVCIDPKLKWIQEYLDKALNK

>btaCXCL13 NP_001015576.1 Chr 6

mrftpgalilmllafslspvhgVLETNNTNLKCKCIRKTVSFFPVNLIERLNIIPRGRGCPNTEIIVWMKNKLVICLNPQAKWTQTLIKVLSKRILSTSPAPVVKKRSD

>btaCXCL14 NP_001029582.1 Chr 7

mrlltaallllllalcaarvdgSKCKCSRKGPKIRYSDVKKLEMKPKYPHCEEKMVIITTKSMSRYRGQEHCLHPKLQSTKRFIKWYNAWNEKRRVYEE

>btaCXCL15 blast search Chr 6

matessqmflllavlvlgifaDPSESQELRCQCIQTQSDFISPKFIAKVQIIPEGAHCNRKEIIVTLKDGQLICLDPEAEWVMNIIKKIV

>btaCXCL16 NP_001039560.1 Chr 19

mmlgrtsrlllvllfiayattsgngNEGSKVGSCPCDHTVSSHSPPNENIMRHLRKYLKAYQRCFSYVRFQLPLKNVCGGSTDGWVQELMHCFDSGECGHAQPRVVDAPLHRTQLPEPTEAAPSDTATTSQTYLPSTLQRTQQPTPLEGALSLDSKLIPTHETTTYTSGHSLGAEPEAKENQKQLKENRGPQAGTSATVPVLSLLAIVFILAGVLLYVVCKRRKNQLLQHPPDLAASLYTCSRRTRAENGTL

>btaCXCL17 NP_001077268.1 Chr 18

mkvlisslllllplmlmsvvssSSHTGVARGQRDQRQASGRWLREGGQECECQDWFLRAPRRTLMAAPRLTKPCPCDHFKGRMKKTRHQRHHRKSNKPSRACQQFLTRCLLESFALPL

>btaCCL1a XP_001253012.1 Chr 19

mkliiaalvclllagmwlqdvdaKSMHVPSSNCCFRTVKGKISPKKIQCYKNISSTCSYNDRLIFKLTGGLQSCVLQKDLWVQAYLKRINLCQ

>btaCCL1b XP_001253062.1 Chr 19

mkliiaalvclllagmwlqdvdaKSMHVPSSNCCFRTVKGKISPKKIQCYKNISSTCSYNDRLIFKLTGGLQSCVLQKDLWVQAYLKRINLCQ

>btaCCL2 NP_776431.1 Chr 19

mkvsaallcllltvaafstevlaQPDAINSQVACCYTFNSKKISMQRLMNYRRVTSSKCPKEAVIFKTILGKELCADPKQKWVQDSINYLNKKNQTPKP

>btaCCL3a XP_873462.2 Chr 19

mevpvaalavllltaalsahtcsASLGANTPTACCFSFVSRQIPRKFVDDYYETSSQCSKPGIIFKTKRGRQVCADPSEDWVQEYITDLELNP

>btaCCL3b NP_776936.1 Chr 19

mkapvaalavllcamalcsqvfsAPFGADTPTACCFSYVARQLSRKIVADYFETSSQCSKPGVIFQTKKGRQVCANPTEDWVQEYITDLELNP

>btaCCL3c XP_603768.2 Chr 19

meipgaslavllltaalsahtcsaTFSTNTPTDCCFSFVSRQIPRKLVDDYYETSSQCSKPGIIFKTKRGRQVCADPSEDWVQEYIIDLELNP

>btaCCL4 NP_001068615.1 Chr 19

MKLCVTVLSLLVLMAAFCSpalsAPMGSDPPTACCFSYTLRKIPRNFVNDYFETSSLCSQPAVVFQTKKGRQVCANPSEPWVQEYVDDLELN

>btaCCL5 NP_787021.1 Chr 19

mkvsatafavllmaaalcapasaSPYASDTTPCCFAYISRPLPRTHVQEYFYTSSKCSMAAVVFITRKKRQVCANPEKKWVREYINALELS

>btaCCL8a NP_776432.1 Chr 19

mkvsagilclllvaatfgtqvlaQPDSVSTPITCCFSVINGKIPFKKLDSYTRITNSQCPQEAVIFKTKADRDVCADPKQKWVQTSIRLLDQKSRTPKP

>btaCCL8b XP_001255313.2 Chr 19

mkvsagilclllvaatfgtqvlaQPVSTPITCCFSVINGKIPFKKLDSYTRITNSQCPQEAVIFKTKADRDVCADPKQKWVQTSIRLLDQKSRTPKP

>btaCCL11 NP_991342.1 Chr 19

mkvsavllcllltatlcsiqvlaQPASIPTICCFNMSKKKISIQRLQSYRKITSSKCPQKAVIFNTKQNKKICVDPQEKWVQNAMEYLNQKSQTLKS

>btaCCL14a NP_001040050.1 Chr 19

mkvsmaavsfllllsitvalgSKNGSSSRGPYHPAECCLTYVSRPVPRQRVSSYYETSSQCPKPGIIFITKKGHYICANPRDGWVQDYIKELEE

>btaCCL14b ENSBTAT00000028567 Chr 19

mkvlvaavfvlctvalcscARKRVHTPPTCCFTYTSGKIPRGNVVNYFKTSSNCPKSGIIFLTRRGLSVCVNPADSWVQEYIRDLEKSP

>btaCCL16 XP_873927.1 Chr 19

mkvsvaalfllvltitsvvhsQPKIPESVNPPPNCCLKYHEKVLPRKLVVGYRQALNCHLPAIVFITKRKREVCTNPNNDWVQEYIKDPRLHPRHSRRLA

>btaCCL17 XP_001788995.1 Chr 18

maplktvllvavllgallqdthaARAGNVGRECCLQFYKGSIPQKVLVGWYQTSDDCPNKAIVLVTRSGRTICANPKDKTVKKAMKYLQKQKKSPASALQES

>btaCCL19 NP_001029517.1 Chr 8

mafqtasllavsllilwvspalgGANDAEDCCLSVTQRPIPVFLVRAYRYLLLKDGCRLPAVVFTTQRGHELCAPPDQPWVGRIIRRLKKNSARRSVPAVTL

>btaCCL20 NP_776688.1 Chr 2

mmcssknlllaalmsvlllhfcskseaASNFDCCLRYTERILHPSILVGFTQQLANEACDINAVVFYTRKKLAVCADPKKKWVKQVVHMLSQRVKRM

>btaCCL21 NP_001033165.1 Chr 8

maqslvlsilvlvlafcilqvqgSDGGAQDCCLRYSRKKIPANIVRSYRKQDPTLSCAMPAILFSPRKRSQPELCADPKEAWVQKLMQHLDKPSAPRKPVQDCKKEKGAPKSGKKGKGSKGCKRTEISKGP

>btaCCL22 NP_001092632.1 Chr 18

maslqtpllaalillamilqateaGPYGANVEDSVCCRDYIRYPLPLRLVKYYYWTSNSCRRPGVVLLTVKDREICADPKQHWVKKILQKLNQ

>btaCCL23 XP_585475.2 Chr 19

mktsiaalpflilaaqaalgFYRPTDCCMSYTPRNIRCVFMENYIETSSACSRPAVIFITKKGQSVCADPNNEGVQKCKSELRLGSAVEDLRSLLVERKRLDRAPSSPSLLPHRPHWRAVA

>btaCCL24 NP_001040061.1 Chr 25

magpvtmatsllllalcippadsVTLPSSCCITFISKKIPESRVISYQLTNRSICPQAGVIFTTQKGQKFCGNPKLPWVQKYVKNLDAKQKKASARARAMSTTAPFWRHLANSTFI

>btaCCL25 NP_001040034.2 Chr 7

mnpwllvclvacfavawgptvhaQGAFEDCCLAYHRRARLSLLRHAQSYHRQDVSGSCNLPAVIFFLPQKNKMVCGRPGDRWVRAWMKILDARKNSQYHHGTRRNFQVPHSGVRKLSSGTSTLPLSRFSGPTRNNKRKTSLLSTANPAGP

>btaCCL26 XP_585155.1 Chr 25

mknfpmasllflvlilsvdlgaaTRGSDVAKFCCFQYSQKTLPWKQVHSYEFTRNICSLKAVIFTTKRGRKVCAQPKEEWVQRHISKLRAQQQL

>btaCCL27 ENSBTAT00000019846 Chr 8

mkgpsptssllllllflspgpggaFPLALSTACCTQLYRQPLPNKLLRRVIRVELQEADGDCHLQAFVLHLSQRRVCIHPQNRSLIRWFERQGKMLQGTQPNQSLELKGKMGWGPQKPK

>btaCCL28 NP_001094633.1 Chr 20

mgmqqtglallalaacvafrpseaILPIASSCCTEVSHHISRRLLERVTTCRIQRADGDCDLAAVILHVKRRRVCVSPHNHVIKQWMNEQAAKKEAKGNICHKKRHHGRRNSKGAHRERQETHGHKTPY

>btaCCL29 NP_001029392.1 Chr 19

mrvslaalaflltlavlhseaNEEPAGNMRVCCFSSVTRKIPLSLVKNYERTGDKCPQEAVIFQTRSGRSICANPGQAWVQKYIEYLDQMSK

>btaCX3CL1 ENSBTAT00000034627 Chr 18

GASVGLGQQHGVNKCNLFCNKMTQKIPESRLVGYQRNRESCNDGAVILKTVKGKSFCADPKEEWVQKAMKHLDHKASVSQKSGTFERQTSEGKPRTTLGAREMYRSAASEVNFTGESSSLGAESALGTSPGVAGSMGTRSSSTSKAPDGGTQKIELFNEAAFTTATSWQSSAADQPEAGLWTEGKASEATSTLVPSTQTLPTPVPSTQADSSGPGSLTVGIKVQDSSKNSLGSKEMSPSSAHTDAFLGSATVSSIFEVTVASEGTPSMDALASGSWGPETEELPKAKEPILTTAGPRSLGILMTSVPDSQVATRRQAVGLLAFLGLLFCLGVAMFAYQRLQSCPHKMVGDVVEGICYVPRSCGSNSYVLVPV

>btaXCL1 ENSBTAT00000013111 Chr 16

mklliltclvicslaAYTVEGVGSEVLEKSICVSLTTQRLPIKNIKTYTIKEGSVKAVIFITRRGFKICADPQAAWVKKAVQKIDRKNISQAKPTGA

>btaXCL2 NP_783647.1 Chr 16

mklliltclvicslaAYTVEGVGSEVLEKSICVSLTTQRLPIKNIKTYTIKEGSVKAVIFITRRGFKICADPQAAWVKKAVQKIDRKNISQAKPTGA

>lafCXCL1 ENSLAFT00000021894 Scaffold 30

martalpaaprllrvallllllvaagrraagAPMGSELRCQCVKTVQGIHPKNIASVKVTPPGPHCADTEVIATLKNGQEVCLNPTAPMVIRLIEKILNTNPG

>lafCXCL4L1 ENSLAFT00000011085 Scaffold 30

mslprssgawharprmrplllgllllpamvafanaSAEPAEEDSDLRCLCVSTTSTVHPKHVISLEVIKAGLHCPKAQLIATLKNGRKICLDQQARLYKKIIKKLLEN

>lafCXCL6 (ENSLAFT00000018004) Scaffold 30

mslrsspavripgpscslclllsllllltppgplasAGPVAAVVRELRCMCLVITPGIHPKMISNLQVIAAGPQCPKVEAIGYLKSGKEVCLDPEAPLIKKIIQRILDGPGLE

>lafCXCL7b ENSLAFT00000025891 Scaffold 30

mslrhdtaslctsarrfgvlqvwlllsllltalvpctsQETNYVEAELYAELRCMCVKTTSGVRPSYINNLQVIRAGAHCDKVEVIVTLKDGKKICLDPEAPVIKKIVQKILQGD

>lafCXCL8 ENSLAFT00000000483 Scaffold 30

mssklvfvllaalvlsaalckaAVLPMVASELRCQCIKTHSTPFHPKYIKELRVIESGPHCPNSEIIVKIVTKEGEEGKEVCLDPTAKWVQKVVQVFLKRELRKKHEKK

>lafCXCL9 ENSLAFT00000032938 Scaffold 30

mkksgvplvlgiiflvlievqgIPTMKNGRCFCININQEKIQLQSLKDLQQFAPSSSCGKAEIIATLKNGDQTCLNPDLKDVKKLVKAWEKQTSQKKKQKKLGKRWKIKKGKTVGKFQRPRQKKTA

>lafCXCL10 ENSLAFT00000007924 Scaffold 30

mdqrailllclifltlsgtqgIPLSRTTRCTCIKISNQPVNPKFLEKLEMIPASLSCPHVEIIVTMKKSGEKRCLNPESKNVKNILKAIRKERSTMSP

>lafCXCL11 ENSLAFT00000033521 Scaffold 30

mnvkgmaialavlfcativqgFPMFKAGRCLCIGPGVKAVRVADIEKASIIYPSYNCDKLEVIIILKANKGQRCLNPRSKQANLIIKVENSKNELRHQNI

>lafCXCL12 ENSLAFT00000016163 Scaffold 10

mdakavavlalllaalclsdgKPVSLSYRCPCRFFESHVARANVKHLKILSTPNCALQIVARLKNNNRQVCIDPRLKWIQEYLDKTLNNQLSH

>lafCXCL13 ENSLAFT00000010038 Scaffold 30

mrftpasllllllaislspvqgFLEANYTNLKCKCLQQTSVYIPAQNIQRLQIFPAGSGCPNVEVIVWMKNNRVVCSNPYTKWFQKLLKVLQKDHLRLSTSPAPMLKRTT

>lafCXCL14 ENSLAFT00000016260 Scaffold 1

raalvllllalcaarvdgSKCKCSRKGPKIRYSDVKKLEMKPKYPHCEEKMVIITTKSVSRYRGQEHCLHPKLQSTKRFIKWYNAWNEKRRVYEE

>lafCXCL16 ENSLAFT00000028866 Scaffold 47

mcrglelefrvflllliagltlpGDGNEGSVTGSCYCDRLISTPTMEQKEHFRKHLKGYTCCRLFVRFELHSRTVCGGSTKPWVLHLMSCFDSGECGRPLWESQARQKHLPPLRTQVPEPTDEAPSDLGTPALTYLPSTLQSTQQPSLPDRGLTYSSEATTLGVEAGENQKQLEDNVGPPGGMSAMVPVLSLLAVTFILIAVLLYVLCKRRREQSRLYYPDLQFHYKPVAPDS

>lafCXCL17 ENSLAFT00000016395 Scaffold 4

mkvliffflllpltlmsvvssSPNPGVARGHRDQRQASRRSLQKDSQECECKDWLLGAPKRKSMTVTVPGLPKKPCPCDHFKGNVKKIRHQRHHKKPNKHSRACQQFLQRCQLASFALPI

>lafCCL1 ENSLAFT00000002372 Scaffold 31

mklitvalvclllagmwtqavdsKSMHVSSSNCCYKFMNKSISLKNIQCYKNTSSTCPYDALILKLKKGRESCVLKTVKWVQDNFSKMKLCLPKRV

>lafCCL2 ENSLAFT00000004718 Scaffold 31

mkvsvalvcllvaaatficqvfsQPDAVNSPVTCCYTFVDKKIVVQRLASYRKVTNARCPKEAVIFKTKLAKELCADPKQKWVRDSTAYLDKKTQMSKPKTHNSAAHSTTQESTIYSTTRESTTYPTTGESAA

>lafCCL3 ENSLAFT00000033251 Scaffold 31

mkvpvaalaillciaalcthvfsTPIGADSPTACCFSYVSRQMKFKSITDYYETNSQCSKPGIIFITKKNQQVCAKPSEAWVQEYITNLELNATR

>lafCCL4a ENSLAFT00000011863 Scaffold 31

mklcvavlsllvlvaafcspalsAPMGSDPPTACCFSYTVRQLPRNFVIDYYETSSLCSNPAVVFQTKRGRQVCANPSESWVQEYMDDLELN

>lafCCL4b ENSLAFT00000029407 Scaffold 31

mkhcmailsllvlvaafcspalsAPMGSDPPTACCFSYALRKLPRNFVIDYYETSSLCAKPAVVFQTKRGREVCANPSEPWVQEYMDDLELN

>lafCCL4c ENSLAFT00000026767 Scaffold 31

meipgasliilllaaalcsqtcsaSFGADTPTACCFSYTSRQIQRKFVDSYYETSSQCSKPAVVFLTKRGRQVCANPSDAWVQEYVTDLELNF

>lafCCL5 ENSLAFT00000018337 Scaffold 31

mkvsaaaltvlvtaaalcvpasaSPYASDTTPCCFAYTPRTLPHAHVKEYFYTSGKCSMPAVVFVTRRNRQVCADPEKKWVKEYINTLEMN

>lafCCL8 ENSLAFT00000022557 Scaffold 31

mkvsavllillltaatfttqlfaQPVAVSIPITCCFNVVSRKIPIQNLVSYTRITNSECPRKAVIFKTKLAKEVCADSEERWVQNSMKRLDQK

>lafCCL11a ENSLAFT00000004717 Scaffold 31

mkvsaellclllvaatfssqvltQPASISNMCCFKVASKKIPIQRLESYTQVASSKCPLTAVVFKTKLAKKICTDPKKKWVRDSMKYLDQKSHTPKP

>lafCCL11b ENSLAFT00000013120 Scaffold 31

mkafvallcllltvatsspqvlaQSDSVSIPPVCCFKVVSKKILIQKLVSYTRITSSQCPREDVIFKAKLDKEICAEPRKKWVQDAMKHLDQNSQ

>lafCCL14 ENSLAFT00000020874 Scaffold 31

mkvltvtvsfliiastlgSKTEPSFEGPYHPTECCFTYITRRVPRTLVSGYYETNSQCTKPGVVFITKKGYALCANPSDDWVQDYIKGLEEN

>lafCCL16 blast search Scaffold 31

IPEAVNQQVTCCLKYHEKVLPRRLVMGYRKALSCHLPAIMNREVCANPNDDWVQEYIKNPNLPLLPSRHLAWVKITIPGKGQP

>lafCCL17 ENSLAFT00000002123 Scaffold 43

mtpmkmlllaalllgaslqhtqaARATNVGRECCLEYFKGAIPLKKLTSWYRTSEECPRDAIVFLTVQGRAICSDPKDTRVRKAVGHLKNLMK

>lafCCL19 ENSLAFT00000033406 Scaffold 6

mapctasllalsllvlwtspalgGANDAEDCCLSVTQRPIPGNIVRAFHYLLLKDGCRVPAVVFTTMRGHQLCAPPDQPWVGRIIRRLQKITPKASLALSPPQ

>lafCCL20 blast search Scaffold 55

micsgkslllaalmavvllhlcsrseaASSFDCCLRYTEHVIHPKLITGFTQQLSSEACDIDAVIFHTKRRLAVCADPKKVWVKQAVWIL

>lafCCL21 ENSLAFT00000022216 Scaffold 6

GAQDCCLKYSRRTIPARVVQSYRKQEPSLGCPIPAILFKPRKQSQPELCADPKEDWVQQLMQQLDKPPAPRKGSQGCRKDKGGPKPGKKGRGSKACKRTEQPTKPKTVIA

>lafCCL22 ENSLAFT00000013608 Scaffold 43

maslqtpllaalillavalqgteaGPYGANVEDSVCCREYIRYPLPLRLVKYFFWTSDSCRRPGVVLLTIKDREICADPRLPWVKKVLQKLR

>lafCCL23 (ENSLAFT00000008588) Scaffold 31

mkvstaalsflilafafgsqgQLIQGFHRPSDCCITYTARNIRCAFVDHYFETSSGCSQPGVIFITKKGQRVCANPLIKHVQDCVNNLKLDLLHKEAGKIALA

>lafCCL24 ENSLAFT00000012480 Scaffold 45

magtatliasllllalcahsitpagsVAIPSSCCMSFISKKIPEGRVVGYQVSNGSVCPKAGVIFTTKKGQKFCGDPKQLWVQRYMKNLEARRKKASPGDKAIGRKAPVRRHPANSTTI

>lafCCL25 ENSLAFT00000022858 Scaffold 114

mnpwllaclvacfigvwvpavhaQGAFEDCCLAYHRHAGRAIVQRAQGYLRQEVSGSCNLPAVIFFFPRRNRKVCGNPQDRWVQNGMKLLDARNKALPKIHKGPLKTRRAGHWGPKTMIPHISRISHTRGAGSFST

>lafCCL27 ENSLAFT00000009964 Scaffold 6

smkgtlptsslllllllnsdpgaaLLSPPSITCCTQLYQQPLPNKLLRKVIRVDLQKADGDCHLQAFVLHLAQHSVCIHPQNHSLAQWFKRQGKRIQGTPPS

>lafCCL28 ENSLAFT00000026418 Scaffold 7

AILPIASSCCTEVSHHISRRLLERVTTCRIQRADGDCDLAAVILHVRRRRICASPHSHTVKQWMKAQAVKKNAKGNICHKKSHHSKRTSKGEQQGTHETSGHRTPY

>lafCX3CL1 ENSLAFT00000021869 Scaffold 43

PGQPRGMNKCEITCNKMTRPIPVKLLLRYEGNPESCGKPAIILVTKKHRLFCANPDEKWVQEAMEHLDRQAAALTENGGTFEKLMGTGGPTATPAKWETYKYAVSEPKAAGESSGPELTPPSQEAQNALGTSPELPTRVAISSGTKSPSTSKAEDEGPPPGPEGTELFNVAAVSTMTSTMITWQSSAAYQPGSGLWVEGTASEAPSTEAPSTEAHSTQDPSTEAPTISHPAPEDSVGAEGQLVWVQEQSPMPENSPGSEEIGPIAAHTDAFLDWGPSSMAHTSVVPVSFVGTPSRERGAKGSCAPKAEETIHTAMDPQRLGALITPIPDPQAATRRQAVGLLAFLGLLFSLGVAMFAYQSFQGCSHRMAGEMVEGLRYGPLICGSNSYVLVPV

>lafXCL1 ENSLAFT00000017778 Scaffold 33

mrllilailaicclgayivegvgsEVPDRSVCVSLHTRPLPVQKIKSYTIKEGSMKAVIFITKRGLKICADPQAKWVKSAVRSMDKKSTTRRNMVQTEPTGAQQSTNTAVTLTG

>mdoCXCL1a ENSMODT00000024367 Chr 5

mrpstcasatagasllrsgllpllllltlawiasaDPPVGFGELRCKCVRTTQGIHPKHIARVEVIVAGPHCHSNEVIATLKTGNVICLNPKAPWVKKFIQKTLDNGPSL

>mdoCXCL1c XP_001375224.1 Chr 5

msrslcqmssllllcllsvfltpsvrlatgAPVANELRCQCLQTVQGVPFKAIGSLKVIPAGPHCSNVEVIATLKNGNQRCLNPEAPQVKKLVEKALKR

>mdoCXCL8 ENSMODT00000024364 Chr 5

mtskllvtllaiclisaalnegAVLSSSVSELRCKCIKTNSKPFHPKHIKELRVIESGPHCLNTEIIVTLQDDRELCLDPHANWVQRVLQAFLKR

>mdoCXCL9 ENSMODT00000024500 Chr 5

mkrnqiaflcglvfltltgvqaFWSSKSRRCSCIDVSADIHRKNILHLEQFPPGSSCSNTEIIATLENGIKKCLDPDSPLVKKAVKAWKKMVSGK

>mdoCXCL10 ENSMODT00000039140 Chr 5

GSPLLRNIRCRCIKSYDAVPNVKVLQKLEVIPQSSSCSHTEIIATIKRTQEQRCLNPDSKQIQNLIKLINNKR

>mdoCXCL11 XP_001376756.1 Chr 5

mnlkvlaillavllypssvhgFSMFRGGWCLCRSSTVNSVYIANIKKVSIFFPSGSCNKIEIIITLKEGMGQICLNPESKQAKLILKRATKKNS

>mdoCXCL12 XP_001365052.1 Chr 1

mdvkvvalvtllvatlalsegKPLSLSYRCPCRFTESNVAKANIKHLKILNTPNCALQIVARLKNSSRQVCIDPKLKWIQEYLEKALNK

>mdoCXCL14 XP_001365117.1 Chr 1

mklltglllllllvlccvgaegSKCKCSRKGPKIRYSDVKKLEMKPKYPHCEEKMVIITTKSVSRYRGQEHCLHPKLQSTKRFIKWYNAWNEKRRVYEE

>mdoCCL1 blast search Chr 2

VNSETTCCYEFQLRKCAYSHIKSYELTSSSCSYKAVIFTLKKGQKICVHLEEKWAQNYLNKLKKSSIKTHI

>mdoCCL2a ENSMODT00000024336 Chr 2

mkvsgavlylvliiaalccqvhaSPYGPTTPTTCCFEFTSKKIPSKFVVSYGITGSRCSKEGVIFVTRRNFEICADPNEQWVQNIINDLDMKNAKTQSP

>mdoCCL2b XP_001374170.1 Chr 2

mkvsgavltlvliaaafwcgvqaTDGSNHPAICCFEFTTKKIPPKLVVNYEATSSTCANNGVIFFTKRGFEICANPEEKWVQNIVKLLDNKKTTTMMTTTTAASS

>mdoCCL2c ENSMODT00000024329 Chr 2

mkmsgallsvvlftayficqiyaMPDGVAIPKTCCFEFTNKKIPLKLLAYYQRTSNMCSKEAVILVTKSGYNLCANPKVHWVNEVMKHLDKMKAKTMKSTKAPTTSSYSSVTSTMTPTVTPTVISTMTPTMTYNVTSTMTPTVASNVTSTMTPTVTSNVTSTMTPTMTSNVTSTMTPTVTSNVTSTMTPTVTSNVTSTMTPTMTSNVTSTMTPTVTSNVTSTPKWE

>mdoCCL3a XP_001373246.1 Chr 2

LGYDIPTSCCHSYTHHRLPYHLIVDFYETSSLCLKPAIVFLTSKGRQICANPKYEWVQRYILLLKQKIRTE

>mdoCCL3b ENSMODT00000024287 Chr 2

mtssvvvlsvlvmaiafcsqvssLSVGGDIPTACCFSYTSWKIPQTRVVDYYETSSKCSKPAIIFITKKGLQACANPRDPWVQELIKSVETRKKKVKFVPSETPRNQGQESVSTMKMATSE

>mdoCCL4a XP_001367613.1 Chr 2

mkvsvvalsilmvmvfsslassAPMGSDPPTSCCFSYVSQQIPRIFVTDYFETSSLCSRPAVVFQTKKGRQVCANPSDPWVQTYVEDLELN

>mdoCCL4b XP_001367565.1 Chr 2

mkisvvalsifmvmafsslalsVPMGSDPPTSCCFSYVSQQIHRKFVTDYYETSSLCSQPAVVFQTKRGRQLCANPSDAWVQTYMEELELN

>mdoCCL4c XP_001367523.1 Chr 2

mkvsvatlsilmvmafsnlassAPLGSDPPTSCCFSYVSQQIHRKFVIDYYETSSLCSQPAVVFQTKRGREVCANPSDAWVQTYMEELEMN

>mdoCCL4d XP_001367476.1 Chr 2

mkvsvvalsilmvmafsslassAPMGSDPPTSCCFSYVSQQIHRKFVIDYYETSSLCSQPAVVFQTKRGRQVCANPSDDWVQTYMEELELN

>mdoCCL4e XP_001367433.1 Chr 2

mkvsaaalsilmvmafnslvssAPLGSDSPMSCCFSYVSKQIPRKFVTDYYETSILCSQPAVVFQTKKGRQVCANPSDDWVQKYKDDPELN

>mdoCCL4f XP_001373172.1 Chr 2

mkvsmtalfilmamavcslaFSVPMSIPTTCCFSYIPQPIPRTFVTGYYRTDSMCPKPAVVFKTIKNQQQCANPNEDWVKTYIEDLERTGQGPEQ

>mdoCCL5 ENSMODT00000024288 Chr 2

mrasaveisiflvlgtlyclaSSSPYASDTTPCCFTFSTRPPPLVHIREYFYTSSRCGNLGVVFITRKNRQLCANPEKKWVRSYINSLEMS

>mdoCCL17a ENSMODT00000040202 Chr 1

mkdlkmsllvalflgillqhshsARAPNLGHDCCITYTKAAIPFSKLVAWFKTPTDCRKEAIVFVTVLKKSICANPNEKWVKKAISFLAKGKKRADSNPETNSDPQNLRLFNPTPASSQTPFNNSAQLPSGSQLNSTWQTNSTQHINSTSLTSSSHLKN

>mdoCCL17b XP_001373626.1 Chr 1

malfiifllgmflqhnhsAGTPHLGHDCCITYTKAAIPFSKLVAWFKTPTDCRKEAIVFVTVLKKSICANPNEKWVKKAISFLAKVSDLLDKLLMGQRVYSHYWKLQQRKNLKQEKGFVIQE

>mdoCCL19 ENSMODT00000039885 Chr 6

LTLRPIPPAAWSGTNDAEDCCLSVTKSVIPRHIVCAYRRLTPENGCRLPAVVFTTLKGHQLCAPPDQHWVKRLIKRLDKNSSQ

>mdoCCL20 ENSMODT00000026993 Chr 7

LHLCGNLEAASSFDCCLQYIEHPINSKFIKGYAEQRSYEACDIDAIIFYTRKHVVCANPKETWVKNVLRILRY

>mdoCCL21 ENSMODT00000009967 Chr 6

mlaflvltvgsrgiqgSDSGALDCCLKYSQKKIPASIVRTYRRQELNQGCSIPAIIFSPWKKSQADLCADPTVQWVQDLVKRLDEHGGTLPKPRKNPCKKDKGSSKSGKKGRDTRGCKR

>mdoCCL22 ENSMODT00000017224 Chr 1

mlvalilgtsvpftqaGPYGVNMENSICCKDFVRFPVPLKFLTNFHFTSKTCRRQGVILTTVKGREICADPQKLWVKNVLKHLLEKN

>mdoCCL24 ENSMODT00000017457 Chr 2

mkasmmvllhliflaicyplhisgaAVVTSSCCTNYMRKTIPRNLVISYRVTNRSACSIPGVIFITKVKREVCADPTKQWVKDYMKMIDTNKAKVSMPVGLKTLKKSLRTPSSNSTSI

>mdoCCL26 ENSMODT00000017454 Chr 2

mkasrlalcvlllgvlcskaqaATHGSNIARYCCETYSPRPIAWKLVQSYELTKSSCSLSAVIFTTKKGQKVCADPKAKWTQRYVASLKSQKVP

>mdoCCL28 XP_001370759.1 Chr 3

mgltgaallaagvllviqaseaILPVASSCCTEVSHHISRTLLPRVKRCHIQRADGVCDLPAVILHVHHRKFCVSPHNHTLKKWMKMQKNKQNGKGIICGKKQHPHKKKSKSRLWAPEKYREFILSSQGFINQEGEASSSCLATCQTPWRGQDPPPPPSLSLPSGGTHSCHWPPKPLAALLLARRPSPFAPVGALSLSPRSIGRLALGPHDVTPSPHFLCCPILPHPGPTEGGSPGEATPPLPFSDWREREGEAGAARRLCGPRLAAGASAPPSHRLLVMRELGEAGEL

>mdoCCL31 blast search Chr 2

TPHACCFRYYSGKIRNVVACYETSSQCPKPGV

>mdoCCL37a blast search Chr 2

mvsltvltiffislssiclgTPYDENTVCCFSYIGRIPRTVLINYKYTSQSCPTPGVIFFTRRGHHQICANPEEQWVQNMVSSLPPKKESEEYEHLEFYP

>mdoCCL37b blast search Chr 2

masltvlaifflsisgiclgSPNSEGVESPRKNHTCCFSYTKLKIPKRHVVDYTYTSEMCSQPGVIFITRKGLQICADPSSRWVQTYVASLEAKVRGS

>mdoCCL43 blast search Chr 2

LEPEPLACCKDFVRKRIPQSFMLGYTRSNPRCSKPAIMFETNKGVKVCANPHTPWVRNYVRNLGGSVGTEEEEGILV

>mdoCX3CL1 ENSMODT00000017225 Chr 1

FLILVTFIALGQHFSMSKCTHECPPLNRDMPQIPNHRLKNYTATSPSCRPKAIIFFTARNKIICADPDVKWVQEAVRFLDESAAAQKMTQGKEDRGKFEKLMGVTGPPTTQVNGGESTVPATTLAPEAANTPVSTTVSPSPSPTPAFSEEEMALMTSPGLPASIVSSSVSFSDSLLVGSTSAMEGEKTLDTGTETSSIKAISATVTPIPESSDSGPLAKGEMSEEMPTLSYPGEEVMGQSEDHPEPVEESDFTTENPIISEEASTVLTLTNPFSNLATDVAEHYFPASIPASESPNQGLVSRTSLPSKFEETAHATVDPQRLEVAITAIPDSPQAATRRQAFGLLAFLGFLFCLGVAMFAYQSLQSCPRRMAGEVVEGLRYIPRSCGSNSYVLVPV

>mdoXCL1 XP_001371932.1 Chr 2

mklfllaflcfcgliaytvegvgsEVMKRRFCVSLTSKRIPVNSVKSYIIEEGSMRAVIFVTRKGIKICADPEVSWAKGVIRAVDSRTTKRNLTQTKPTSQPSTDRIMSISG

>meuCXCL1a blast search Scaffold 78776

SWTGTVGELRCRCVKTMQGIHPKNIVSLEVIRAGPHCPNHEVIATLKKGSEICLKAEAPWVKKFIQRYLTYGSAR

>meuCXCL1c ENSMEUT00000016371 Scaffold 392552

APVANELRCQCLQTVQGIPYKNIANLKVIPAGPHCSNLEVIVTLKNGNERCLNPAAPQVKKIVEKALK

>meuCXCL10 ENSMEUT00000011517 Scaffold 84604

mnrsatlctvlllcwlfiilprvqgSPLSRSVRCRCIKPHDGVPNVKSLQKLEVIPQSSSCPRTEI

>meuCXCL12 blast search Scaffold 5023

GKPVSLSYRCPCRFTESNVAKANIKHLKILNTPNCALQIV

>meuCXCL14 ENSMEUT00000000899 Scaffold 39936

mklltglllllllvlccvgvdgSKCKCSRKGPKIRYSDVKKLEMKPKYPHCEEKVVIITTKTMSRYRGQEHCLHPKLQSTKRFIKWYNAWNEKR

>meuCCL1 blast search Scaffold 135138

VDSKPTCCFAFLKRSLPHNLIKSYAITSRSCSYKAVMFTLKKDKKYCAHLEDKWVQDYLKKNEHKNSNQS

>meuCCL2a ENSMEUT00000000694 Scaffold 70506

GPSTPTTCCFEYTAKKIPPKYVVSYEVTSSRCAKEGVIFVTKRGFQICTNPKEQWVQDIRNHLDKNNAKTQT

>meuCCL2b ENSMEUT00000009125 Scaffold 335857

GPGTPTCCFDFTSKRIPSKLVVSYGTTSSRCAKEGVIFITRQGHNICANPKEQWVQHVKKQLDSKTAKTQSS

>meuCCL2c ENSMEUT00000005281 Scaffold 89888

mkisgavlpmillavyficqvyaTPDGVYSPENCCFEFTNKKIPLKLLVSYKNTSSMCPKEAVIFVTKRGFNICANLKDLWVQDLMKNLDKMKTKIMK

>meuCCL3a (ENSMEUT00000014449) Scaffold 10172

THYVPNACCHSYTRRRIQYSLLVDFYETSSLCLKPGIIFLTNKGHQICANPKSEWVQEYIFQAEREEKD

>meuCCL3b ENSMEUT00000009992 Scaffold 10173

GSVAPTACCFSYTSRKIPRAVVVDFYETSGHCSQPAIIFLTKKGYQACANPSEPWVQKYTKELK

>meuCCL4a ENSMEUT00000007560 Scaffold 25802

mkvsvaafsillvmafsslassAPVGSDPPTSCCFSYVSQQIPRKFVTDYFETSSLCSQPAVVFQTKRGRQVCANPSDAWVQSYVEDLEL

>meuCCL4b ENSMEUT00000007568 Scaffold 25802

mkvsvaalsillvmafnslassAPMGSDPPTSCCFSYASQQIPRKFVTDYFETSSLCSQPAVVFQTKRGRQVCANPSDAWVQSYVEDLEL

>meuCCL4c ENSMEUT00000005799 Scaffold 15037

mkvsvaalsillvmafsslassAPLGSDPPTSCCFSYVSQQIPRKFVTDYFETSSLCSQPAVVFQTKRGRQVCANPSDAWVQSYVEDPEL

>meuCCL4d ENSMEUT00000005803 Scaffold 15037

mkvsvaafsilmvmafsslassTPMGSNSPTSCCFSYVRQQIPRKFVIEYYETSSLCSQPGVVFMTKKGRKMCANPSDNWVQKYMADPEL

>meuCCL4e ENSMEUT00000005813 Scaffold 15037

mkvsvaalsilmvmafsaltfsAPMGTNPPTSCCFFYARQQIPRIFVTGYYRTSGLCSKPGVVFNTTIGKESCANPSDDWVKEYMADPKL

>meuCCL4f (ENSMEUT00000009271) Scaffold 216887

mkvsvvalsillvmafsslassAPTGSDPPTSCCFSYVSQQIPRKFVTDYFETSSLCSQPAVVQKEVGRCVPTPSDIWVQSYVEDLELN

>meuCCL4g ENSMEUT00000006796 Scaffold 272879

GSDPPTSCCFSYVSQQIPRKFVTDYFETSSLCSQPAVVFQTKRGREVCANPSDAWVQ

>meuCCL5 (ENSMEUT00000008832) Scaffold 10207

ASDTTPCCFSFTSLPPPLTHVREYFYTSSRCGNQGVVFITRKNRQLCASPEKKWVRSYINSLEMS

>meuCCL19 ENSMEUT00000014847 Scaffold 50948

AAWGGTNDAEDCCLSVTNLIIPRHIVCAYRRLNPENGCGLPAVVFTTQKGHQLCAPPDRFWVKRLIKRLERNNPQVS

>meuCCL20 blast search Scaffold 62606

SSFDCCLRYTERPVNTKLIKGYAEQWSYEACDIDAIIFYTRRYAVCADPKEAWVKHALKILRY

>meuCCL22 blast search Scaffold 29904

NMESSVCCRNFVQFPLPLKVLTTFYYTSKNCRKRGVILKTVKKLEICADPQKVWVQRALATLKKKK

>meuCCL25 blast search Scaffold 30030

GIYEDCCLKYSKHRKPALLRHILRYRIQEVNGSCNLRAV

>meuCCL26 ENSMEUT00000006341 Scaffold 131585

lgssvllsgvlcskgqaAIQGSNIAKYCCEKYSSRPIPRKLVQTFELTRSNCGLSAVIFITKKGQKVCANPKAEWTQKYVASLKFL

>meuCCL28 ENSMEUT00000013038 Scaffold 5978

ILPVASNCCTEVSHQVSRRLLAKVKRCHFQRADGVCDLPAVILHVHHRKFCVSPHNHTLKTWMETQKNKKNTKGIICNKKKHPHENKRKSKVTKDHKHGQKTPY

>meuCCL31 blast search Scaffold 10173

PNACCFHYATRKIRNVVACYETSSRCPKPGVM

>meuCCL37 ENSMEUT00000009996 Scaffold 10173

DASPASCCFSYVTRLIPRKFVVDYDYTSQECFNPAVIFTTSRGFKICADPQKQWVKQYVANLK

>meuCCL43 ENSMEUT00000008839 Scaffold 10207

DSEDSTPCCNNFVNHRIPQSLVIGFVRTSLRCPKPGVLFETKQGLKVCANPAVHWVQRYMKNLGLHTW

>meuCX3CL1 (ENSMEUT00000013957) Scaffold 40468

QHFSMNKCTHECPPLSRNLPQIPRQFLKRYDLTSPSCRIQAILFTTIKNRIICADPNEKWVQDAIQFL

>oanCXCL1a ENSOANT00000011013 Ultra 362

LFAAAPPGARLATELRCQCVKTTQGIHHSNMAKVEVIPAGPSCANVEVIVTLKNGKNVCLNPDAPRVKKLIEKMLNGKDRRFLSLSAKKKERTDKVQQHLSR

>oanCXCL1b ENSOANT00000011017 Ultra 362

LFTAAPQQLLLPSELRCKCVRVTQGIHHSKIQNVEVIPAGPHCSEVEVIATLKNSQIVCLNPQAPLVKKLIKKLLNKGEYN

>oanCXCL8 XP_001511324.1 Ultra 362

mtgkfvfallavmiltatvseaASISKANIQLRCRCISTHPKRIARKHIKSVEVIFKGPHCSLDEVIVTLQDNKEVCLDTTKDWVQELIEKYKKIIEKNNQM

>oanCXCL10 ENSOANT00000011036 Ultra 362

msragvapvllavpllmlalapvrgPSGSPLHGPGRCRCLENSSKFIHPNALAKLEFLPKSSSCSNNEVIATLKKSKEEICLNPDSRNVKKLLDTILKKNQVR

>oanCXCL11 XP_001510031.1 Ultra 362

MSSPLPPVVPRFRGSRCLCIGPRVNSVTPMQIKSISVFLPTSTCDRKEVIVTLKKGKGQRCLNLDSKQAQLILKVGPSPRPQGRPKSIRERGPGPWRSPRRRVGDDGQVPPGCLQLEPSPHVAAFLRSPLLQSPSSAYWAFSALEPRITAKLPAYTLQ

>oanCXCL12 ENSOANT00000001026 Contig 4520

mdvkvpallvlllvtlslsegKPISLSYRCPCRFSESNVAKANIKQLKILNTPNCALQIVARLKNSRQVCIDPKLKWIQEYLEKALNKKKKRFQA

>oanCXCL14 ENSOANT00000031622 Contig 20394

mklvtvaflllfvaicsvgvdgSKCKCSRKGPKIRYSDVKKLEVKPKYPHCEEKMVIITTKSVSRYRGQEHCLHPKLQSTKKFIKWYNAWNEKRSVYIL

>oanCXCL15a ENSOANT00000032411 Ultra 362

manklsltglllfilfldfsesSKGLQLRCQCISTHSDPILRHLIVNVRHIPKGAHCSTTEIIAELINGQLVCLNPEAKWVKILIERILKSREGQINININK

>oanCXCL15b XP_001511166.1 Ultra 362

IQLRCQCIRTHSDFIHPKFFANIQYIPAGPLCDTPEVIAEMKQGNEICLDPNANWVKIIIQKILSS

>oanCCL2a ENSOANT00000004655 Ultra 42

EVHIPQSCCYSWSKKPIPLHLLSGYFVTSSKCSLEAVIFKTVKGVEVCADPKEKWVQDRMRRLKIRRKNP

>oanCCL2b XP_001518964.1 Contig 16219

GVNIPQSCCFSWSKKPIPLHLLSGYFVTSSKCSLESVIFRTVKGVEICTDPKEKWVQDRIKRLESRRKKP

>oanCCL3a XP_001512383.1 Ultra 42

mqgpvvtlsalllvaalcsptssAPVGTDIPTICCFSYTSRPLPIRLLSYYEETSSRCSKPAIIFTTKKGREVCADPSEEWVQDRIQDLKHS

>oanCCL3b ENSOANT00000004651 Ultra 42

VHVPVTCCTNYLHRPLPQKLVKSYFQNRSQCPKPGIIFITVKGRKVCADPSEAWVQKIQARIRDLEWNR

>oanCCL17 (ENSOANT00000029855) Ultra 517

TNTGKECCSDYIKKPIKFCKLVSYQKTSVDCLKDAIVLNTIQNNTICANPNQPWVKSAVKYLKRRKSKTRPASHSC

>oanCCL20a ENSOANT00000028815 Contig 7266

mlalnhkklvlasvmlvlslylfraseaSDFDCCLRYTERHYGSRIIKGYTEQFSNEVCDIDAIIFHTKRGALVCANPQEQWVKHVLHDLSKRLKRM

>oanCCL20b ENSOANT00000004505 Ultra 477

mpgvtsqtlvlasslllllfsaskaKDDLDCCLSYIQKPLPYQLIKDFIEQLPSETCDISAVILITRKKRLLCANPKDKWVKELILRYEDLSKNPKL

>oanCCL21 blast search Contig 8421

GDNGAPDCCLAYTQQIVRLNIVRSYRQQDTNGGCTIPAVIFSPKNPKRKDICADPSMPWVRELLRKLDRFPKGSCGKNRSQGQTCRR

>oanCCL22 ENSOANT00000021683 Ultra 517

mkqlmvtflvvlvlvaslpsgqaVPYATNLEDSICCTEFVKWPVRFRYLTEFYFTSLSCRRRGVVLKTVKNLEICADPQIPWVKKAIDLLKL

>oanCCL24a blast search Contig 42

EWCCFTFTRKMLSWSLIMSYFRANKDVCPEPAILFVTKKGLRICANPKEDWVQRYVKALDQL

>oanCCL24b blast search Contig 42

AYPVADACCVSYSQKRIPLNRVASFSVSNSGICSKPGVM

>oanCCL27 ENSOANT00000000752 Contig 6869

LRVCPRTALFPFPAASCCTELSRHLLKPQLLKKVTGVKLQETGGGCHLRAIVLHLGRRFLCIHPKNRSLTRWFEQLRKLGRGHLIPRGA

>ggaCXCL8a NP_990349.1 Chr 4

mmgkavaavmalllismagaKGMAQARSAIELRCQCIETHSKFIHPKFIQNVNLTPSGPHCKNVEVIATLKDGREVCLDPTAPWVKLIIKAILDKADTNNKTAS

>ggaCXCL8b NP_990829.1 Chr 4

mngklgavlalllvsaalsqgRTLVKMGNELRCQCISTHSKFIHPKSIQDVKLTPSGPHCKNVEIIATLKDGREVCLDPTAPWVQLIVKALMAKAQLNSDAPL

>ggaCXCL8c XP_420608.1 Chr 4

msprlllplllaataalcwgAPPAGELRCRCVRAVAERIPPRHLVQVELVPEGPHCAAPEVIATTKQGHTLCLSPSVPWVKLLVARFLNSAAQRS

>ggaCXCL12 NP_989841.1 Chr 6

mdlralallafalavisLSEEKPVSLTYRCPCRFFESNVARANIKHLKILSTPNCSLQIVARLKSNSKQVCIDPKLKWIQEYLEKALNK

>ggaCXCL13a ENSGALT00000016833 Chr 4

ILEVNGNLNCRCVKTTSDYISPKRYDSIELRPVGSTCRRIEIIIKLKSSAKVCVNPDAPWVKKLLKRIAGTKKR

>ggaCXCL13b XP_420474.1 Chr 4

mavraalllglllvvlcpgdaAILEANGNLNCRCAKTTTAFIPLRKYESVEVRPVGSSCRRLEVLIKLKTLERICVDPNTPWVRKLLQDLPKLKKKAAPQ

>ggaCXCL13c XP_420473.2 Chr 4

mralqaalalglllssllpgdgLSLESLLTNKRCKCVKVTAQIISLGLILAIDVMPPGIHCRRKEIILTLKRNKKVCVAPEAPWIQLLIHKLTQTDVSKKEAAAVARSRGEAGRQPPVP

>ggaCXCL14 NP_990043.1 Chr 13

mklltaallllviamclasaegVKCKCSRKGPKIRFSNVRKLEIKPRYPFCVEEMIIVTLWTKVRGEQQHCLNPKRQNTVRLLKWYRVWKEKGRVYEE

>ggaCCL1 ENSGALT00000003670 Chr 19

mkvfslvmvtlllaavwtessgKSFRSSYSSCCYKNMFIQKEINTSLIRRYRETPPNCSRRAIIVELKKGKKFCVDPAEGWFQQYLQGKKLSNTST

>ggaCCL4a NP_001025531.2 Chr 19

mkvsvaalavlliaicyqtsaAPVGSDPPTSCCFTYISRQLPFSFVADYYETNSQCPHAGVVFITRKGREVCANPENDWVQDYMNKMELN

>ggaCCL4b NP_001039296.1 Chr 19

mksstaaiavlivaalcyqvssTPLAVGSNGRCCYKFLNRALPSSKVMMYEYTGSRCPYHGVIFTTFEGKKCCANPEEKWVQDILNVEKHTDGSK

>ggaCCL5 NP_001039297.1 Chr 19

mmtavavslsillvaalfpqassSPFGADTTVCCFNYSVRKLPQNHVKDYFYTSSKCPQAAVVFITRKGRQVCANPDARWVKEYINFLELQ

>ggaCCL17 XP_414018.1 Chr 11

mlstklvllvllllsifqyssaAPYAPSECCYEHTKFALRLEALKSFYETSHDCLLQAIVFVTKNGTKVCSKPNAPWVKKAVKYLQKKNNPQAV

>ggaCCL19 XP_424980.2 Chr Un_random

mqrlhvlclsllvlrcvlhvyaGNNVLDCCLRTSEKPIPWRIVQDYRMQLVQDGCDIPATVFITAKGKRLCAPPQAPWVLRLREKLDTSSARK

>ggaCCL20 NP_989769.1 Chr 9

mpglstkslilasllgllllllcstsqaQSNQDCCLSYSKVRLPRKVIKGFTEQLSGEVCDIDAIIFHTVRGLKACVNPKEDWVKKHLLFLSQKLKRMSM

>ggaCCL21 CR522995 Chr Un

malrillpllllaaalllhqaegVDNPASDCCLKTSQKAISMKWVKSYSIQGPESGCVLRAVVFTTKKNKKICSSPTDPIVQKLIKSLDSKRKSTPQRKSKRQKRKQV

>ggaCCL28 blast search Chr Z

LFPEAFNCCTKISEEIPKGILRRVERFEIQKYDGLCHLHAVILHIEGRKFCVSPRIKRLKKWMKKNKHKIPRKKHHGRRQRRTKILKKENRQ

>ggaCCL30a NP_001025883.1 Chr 19

mkgsaaalaallllalcssavaHLDGLPTTCCFSYVQRPVPRNLIASAYITSSKCRLPAVILVTKKGREICVNPEESWVQKRLELLQKQEN

>ggaCCL30b XP_415781.2 Chr 19

mkgsaaalaallllalcssavaHLDGLPSTCCLSYVQRPVPRNLIASAYITSSKCRLPAVILVTKKGREICANPEESWVQKRLELLQEQEN

>ggaCCL30c XP_001233779.1 Chr 19

mkgsaaalaallllalcssavaHLDGLPSTCCFSYVQRPVPRNLIASAYITSSKCRLPAVILVTKKGREICANPKESWVQKRLELLQEQEN

>ggaCCL30d XP_415780.2 Chr 19

mkgsaaalaallllalcssavaQLLDSDGLPTTCCLSYVQRPVPRNLIASAYITSSKCRLPAVILVTKKGKEICVNPEESWVQKRLELLQNQEN

>ggaCCL31 XP_415779.2 Chr 19

makaagafcilllltalccqslaQRAPAVPDKCCFNFHTRRIKMDNIVACYATSPQCPHRAVVFKVKNGKEICTPADRMWVKRYQQRFQVSSYSIPS

>ggaCCL41 NP_990051.1 Chr 19

mklsavvlalliasfcsrassAPVGPDVPTCCTTYITHKIPRNLIQRHYSTSTSCSKPAIIFITKKEREVCANPSDPWVQRYLQSVKRD

>ggaCX3CL1 NP_001070700.1 Chr 11

mrvaslqipfalrvlclaamaggQPRAPLKCSKWCISFHRAIDQRQIKSYRETEPQCTKKAIIFTTKRNREICANPYEPWVEKIVKKLDQEKASAASPLPRADTSPAAAVPKEPGIFQKHTGLQVPPSPPATAATAASERAPTPAASTEATSKPSPAMQNATHFSAGPSAVTSGVATHSEVVSEANRESLTSAHSTADAVDMALGQRTSYPTAPARDSDSKEEPAGYATSAAGDVRGTTSTSTSDPASISKGLDHPSLPTNVPLDTISARGSTSGTALRSSALPSTPHITEVGMVPSTPQASPSPTQNPTTAIDEGPYVHANKNFSSSAFGTGTLDHLLPSGKQGPLDMLVFTSQIFSDQARAQATGSPSHPPALSSLSGSQMYLVIPVALIGVLIACGVAARWAYVKFEIRPETTSREMVEALLYLKEGHRDNVYPMEVI

>ggaXCL1 NP_990377.1 Chr 1

mklhatvllvivwlgvfalhtaegSVASQSMRKLSCVNLSTQKVDIRSIVNYEKQKVPVEAVMFITANGIRICVHPEQKWVQSAMKRIDRRRTTRRR

>tguCXCL8a XP_002192050.1 Chr 4

mmgktvaavltlllisalgtqgEVVPRSAIELRCQCINTHSKFIHPKFIQNVNLTPSGPHCKNVEVIATLRDGREVCLEPSAPWVKLIIKTILDKANTKPETVY

>tguCXCL8b XP_002193466.1 Chr 4

mngklvailalflisaavsqgRTLARMGTELRCQCIATHSRFIPPKSIQDVKLTQSGPHCKNVEVIATLKDGREVCLEPTAPWVQLIVKAILAKAEQNSDSPL

>tguCXCL12 XP_002187359.1 Chr 6

mdlralallafalavislsEEKPVSLTYRCPCRFYESNVARANIKHLKILSTPNCSLQIVARLKSNSKQVCIDPKLKWIQEYLEKALNK

>tguCXCL13a ENSTGUT00000001797 Chr 4

mgapllpwllllllvmshsahaAILEVNGNLSCRCAKTTSEYISPKKYESIEIRPVGSSCRRVEIIIKLRTSGKVCVNPEAPWVKKLLKRIASTKKK

>tguCXCL13b XP_002193202.1 Chr 4

mavraalllalllllshrpgdaAILEANGNLSCRCLKSTRAFIPPERYSSIEVWPVGSSCRRPEVVLTLKKNKRVCVTPEAPWIQLLIHKLMQSCPKIQGKQFPT

>tguCXCL14 XP_002187341.1, XP_002191481.1 Chr Un

mklltaallllciamclasvegVKCKCSRKGPKIRFSNVRKLEIKPRYPFCVEEMIIVTLWTRVRGEQQHCLNPKRQNTVRLLKWYRVWKEKG

>tguCCL1 XP_002193626.1 Chr 19

mkissallilllaaawtrsqgLSLRSSKAHCCSKEMSSRRKIPEFKIQGYLETPSTCTYRAVLVQLQKGMVCVDPEKKWFQEYLRKQKKPNSTST

>tguCCL4a XP_002187982.1 Chr 19

mkvfvialtilmaafcyqtsaAPLGSDPPTSCCFSYISRQLPRSFVKDYYETNSQCSQPAVVFITRKGREVCANPAEEWVQQYVNELELD

>tguCCL4b XP_002188128.1 Chr 19

mktfpaalsvlfvvvvfyqatsSPISLNFYGPCCVEYITRPLPLSRVVKYEHTGSHCSPPAVIFTTIKDKLVCANPNDKWVQDIMNELKDHKHSG

>tguCCL5 XP_002188017.1 Chr 19

mnistlclsiilladlfsqalpAPLGSDMALCCFSYISRKLPQNHVQEYFYTSSKCSQPAVVFVTRKKREVCANPDARWVKEYVNSLEMQ

>tguCCL17 ENSTGUT00000005497 Chr 11

AAPYSPAECCFDYVKGVLRLEILVGFYSTSKECFLPAIVFDTKKKAKICANPEEKWVKRAVRVLLKKKGLHA

>tguCCL20 XP_002198509.1 Chr 9

mtgcsksmvlisllgllalllwgtseaHSNQDCCLSYTKARLPRKALKGYTEQLPSEVCDIPAIIFHTASGRNACVNPKEGWVKKHLLFLSQKLRKMSV

>tguCCL21 XP_002189872.1 Chr Z

malrpfllllpllaaallitqaqgIGSSALDCCLKHSTLKKDIPSGVVIAYRQQGPETGCYLRAVVLITKKNKKICVSPTDDTVLKLMQQLDKKAKNDKDKKAKNNKNKRQTQRPRGRPKKQKRQRV

>tguCCL28 blast search Chr Z

LFPGAFNCCMKISDEISKGILRRVERFEIQKAGGLCHLEAVILHMKGRKFCVNPWNKKVKKMMKKMKHKIHRSKFHVRKQRRTRITKQKEQKQ

>tguCCL30a XP_002197551.1 Chr 4

mrvlaaalavlllvaicslaeaDLRVSRSPALSKDEDKTMCCFSYISRRIPRSVISSAYITSNTCSMPAVVLITRQGKKICADPKADWVQKHLKHLERLEH

>tguCCL30d XP_002193653.1 Chr 19

mkvlaatlvtllllatlspaegHLDGVPSTCCFSYQRQPIPLRRVSSVFVTSSSCSLPGVIVVTQKKKQVCADPRAAWVQQLQKHFQSLEN

>tguCCL31 XP_002193593.1 Chr 19

mgtairvvcallvlstlcwhslaQRAPAVPDKCCFNFQTRRIKRDNVVACYPTSPECPHQAVIFKVRNGKEICTQASRPWVKRYQQSFQVSSFSIPS

>tguCCL41 ENSTGUT00000003333 Chr 19

mkvsaaglalllivaafsqtfsGPAGLNIPICCFTYSQHKLPWKLIQHHYITSSSCPQPAVVFVTKEGRQVCANPENAWVRRYLKILGQN

>tguCX3CL1 XP_002195805.1 Chr 11

mkaaclqtlllllralclvslaggQPKAPVKCSNECRNFTRRLPEKLIRSYRETEPGCGRSATILITLKFKEICADSTEDWVKKIKDKLDRKKATVMPPHDVTSAEEPGKQEPSDTVGFTHQALSGQARVQVITVRPNNLPLPSFLSKSEMHFVIPVSVVCGLMVGSVALVWVYLKFGVKPEETSREMVQGLLYQQAGHQDNVYPMEVI

>tguXCL1 XP_002190097.1 Chr 1

mklhttailllfwfgvftvhtvrgSSSSESMRKFKCVNLSTRQLNIRNLVNYEKQQVPTDAIMFLTAGGIKICVRPNQEWVQAAIKRIDERRTAKPHNPSF

>aplCXCL8a ENSAPLT00000013206 Scaffold 229

mmgkvvaagmalllisvigtegMALARSGIELRCQCIETHSKFIHPKFIQNVNLTPSGPHCKNVEVIATLKDGREVCLDPTAPWVKLIIKAILDKKAKIGDVTAQ

>aplCXCL8b ENSAPLT00000013216 Scaffold 229

mngklgvvlalllisvalsqgRSLVRMGNELRCQCISTHSKFIHPKSIQDVKLTQSGPHCKNVEIIATLKDNREVCLDPTAPWVQRIVKAILAKAQLNSDAPL

>aplCXCL12 ENSAPLT00000015984 Scaffold 2353

EKPVSLTYRCPCRFYESNVARANIKHLKILSTPNCSLQIVARLKSNSKQVCIDPKLKWIQEYLEKALNK

>aplCXCL13a ENSAPLT00000003780 Scaffold 1279

AILEVNGNLSCRCVKTTSDYISPKRYESIEIRPVGSTCRRTEIIIKLRTSSKVCVNPEAPWVKKLLKRIAST

>aplCXCL13b ENSAPLT00000003779 Scaffold 1279

ALLEANGNLSCRCAKTTAAFIHPSKYESVEVRPVGSSCRRLEVVIKLKSLERVCVDPNAPWVKKLLQDLPKL

>aplCXCL13c ENSAPLT00000001294 Scaffold 1279

GSGLSLESLLTNKRCKCIKSTAQVISLGLILAIDVTPPGIHCRRKEIM

>aplCXCL14 ENSAPLT00000005384 Scaffold 627

klltaallllfiamclasaegVKCKCSRKGPKIRFSNVRKLEIKPRYPFCVEEMIIVTLWTRVRGEQQHCLNPKRQNTVRLLKWYRVWKEKG

>aplCCL1 ENSAPLT00000010187 Scaffold 108

mklfsltlvalllaavwteswgLSFRSTYSKCCYKFVKNISASSIRSFKYTLPNCSRRAELLQVMELLDGTKVCADPRKEWFQKYLNRQKLSNASA

>aplCCL4a ENSAPLT00000013296 Scaffold C20269202

mkvsvvalavliaafcyqtsaAPIDSDPPTSCCFTYVQRELPRSFVTDYYETNSLCSQPGVVFITRKGREICANPEHDWVKKYVIELELN

>aplCCL4b AY641436 editted Scaffold 436

mksstaalavlvaalccqvlsspaAVNFSGPCCVKYSSKSFSSRHVTMYQNTSSHCPQPGVIFTTFKGKSFCGNPKDEWVQNILKQHKDQADSV

>aplCCL5 ENSAPLT00000010244 Scaffold 436

mnifsaalsilllaglfsqafsGPIGADTTICCFSYTSQKLPQSHVKDYFYTSSKCPQPAVVFITRRTRQVCAKPDARWVKEYVNFLEMH

>aplCCL17a ENSAPLT00000012795 Scaffold 4503

mlstrpvllllllltfsqhcatAPYSPSECCFEFLKPALRYEVLKDFYETPKECFSPGIVFETKNGNKVCAKPNTPWVKKAMEKLLRK

>aplCCL17b blast search Scaffold 2132

LYTPTECCFDYAQKPVRIANIKSFYTTSNDCALPAVVFVTAAGFKVCNNPQKPWVKKTLNKLRRKK

>aplCCL19 ENSAPLT00000005578 Scaffold 1052

qlhllclgllvlgcilhxxxxxhgGNNVLDCCLRTSENPIPRRIVQRYQIQLVQDGCEIPANVFITVRGKRLCAPLEAPWAVRLREKLDSGSARK

>aplCCL20 ENSAPLT00000008502 Scaffold 1369

msafstkslvlasllgllllcstsqaQSNQDCCLSYTKARLPRWAIKGYTEQLSSEVCDIDAIIFHTFSGLKACVNPKDVWVKKHLLFLSHKLKKMSM

>aplCCL21 blast search Scaffold 2382

malrillpllllaaallllpaegVDNVASQCCERTSLEVIPSFLVKSYRIQGPESGCRLHAVVFTTRKEKKICSSLTSRAVQKLIRRLEDKQKNPSQKPRRLSFAQQKAKKTPAPKLISDVEYTALKSEIEMLKSSLGFERETGKTLGIRVDQLLNENDELLNKNNKLVAENDKLVAEYDELLNESEELMNENNRLVAENDKLVNENSRLKQLLERFIHLLNKARPSASVKKVRRLMHTADPESWDGDFWFDIDDNVKEIADSESQSISSRPLVKTETTVDDDDEIYNTVRTIPWPPVELVKIQKFSRWSGKSEVDFVWRVLLEGRD

>aplCCL28 blast search Scaffold 1686

LFPGAFNCCTKISDEIPKGILPRVERFEIQKADGLCHLEAVILYIGDRKFCVSPQIKRVKKWMKKKKHKIAGKKTHGRKQRRSKNNKKSEKQQ

>aplCCL30a ENSAPLT00000010196 Scaffold 108

mkvfaavlaallllalcspavaHLAPTDGVPTTCCFSYQQRPVPRSLIASAYITSTSCSQPGVILVTTKGRDICADPQATWVKAHLKHFEKKN

>aplCCL30d ENSAPLT00000010191 Scaffold 108

mkvsaavlaallllalcspavaQLDNALTKCCFRYLKRPVPRSVIASAYITSTSCSQPGVILVTTKGRDICADPQATWVKAHLKHFEKKN

>aplCCL31 ENSAPLT00000010184 Scaffold 108

makaagafcvllllitlcyenlaQXXXXVPNKCCFNFQTRRIKRDNVVSCYATSPECPHQAVIFKVKSGKEICAQAGRAWVKRYQQSFPVSSFSIP

>aplCCL41 ENSAPLT00000009771 Scaffold 436

mkvssvvlallliaascsqtssAPVGPDHPTCCFSYTSHKLPKKLILRYYVTSTSCSLPALVFITKKGREVCANPSDTWVQRYLQNMKQN

>aplCX3CL1 ENSAPLT00000012814 Scaffold 4503

mklvsfqtllvlrvlglltpaggQPKAPIKCSKVCNRFTRILPEKRIKSYHKTEPQCAKQAIIFITLASLEICADPKNDQVQKIMRKLDQKKASAASPHAATSAAVPEKPGIFHKHIGLTVTAPSQATAATTLFQGTGTTVLERTHAPAAMTEVTSKSPPAMQSTTQFSAGSSEVSSEANRDSLKPAHSTTSAAGMVSSQPILHPTALVHGFDNTVGLTEEPVGYTASATADVQDTSSPSSNSDPVSVTKGLDHPVLSTDESLDPTSARANTSDTASRSSNSDLPSILNSMEISSAPATPVPPETTSTSTLNPTTAIAKGPPAHTNNIFSSSADAVGTRTFGYSSPLGKQDHPGTLVLTTQPFSGQARAQLTTKRPHDSPSLSFLARSQTHFIIPVSLVCGLIACTVAAVGLYTKFGVRTETVSREMVQGLLYQKEGHQNNVYPMEII

>aplXCL1 ENSAPLT00000017145 Scaffold 2455

mklhtaailvifwlgvftmhtakgSIGSQPMRKFSCVTLSTQKLDIRQLVNYEKQQVPLNAVMFITAKGIKICVHPDQKWVQVAMKRIDQKRTAKKK

>acaCXCL8a ENSACAT00000011352 Contig AAWZ02035874

mntkvvvallafflacatlteaLPMARMGSELRCQCISTHSRFIPPRNIQDVKLTQSGPHCTNVEVIATLKDGREVCLEPTANWVKVIIKAIL

>acaCXCL8c blast search Chr 6

APMTGELRCRCIQKVSEVIAPKHFANIELTPEGPHCPVSEVIATLKSGKEVCLDPTSRWVKMIISKILKQVLAKHVN

>acaCXCL12 ENSACAT00000011734 Scaffold GL343297.1

mesrrlallallllallgasqeKPISLTYRCPCRYVESSVAKSHIKHLKILTVPGCSLQIIARLKNSSKQICIDPKLKWIQEYLEKYLHKPKYRCCVRKWRETFGPL

>acaCXCL13a blast search Chr 5

NESCKCLRVRSEFINPTKFARVEILPAGILCQRMEIIITLKGNRKVCVNPESKWVQVLVKLIQNT

>acaCXCL13b blast search Chr 5

IVTHVGRCRCLKQTSSPFSPRQLKSIQVFPHGMQCQNTEIILTLKNKWKVCVDPSAPWVQELLKVVTKR

>acaCXCL14 blast search Scaffold GL343223.1

SKCKCLRKGPKIRFSDIQKLEERPKYPYCKERMIIVTMKSRFRGGHQYCLHPKLPSTKRLLKWYTIWKEKE

>acaCCL20 blast search Chr 3

QNNQDCCLSYTKRPLPRRVIKGFAEQLSSEVCDINAVIFITKNGLRACANPKDQWVKKHLQWLR

>acaCCL28 blast search Chr 2

IFPIIVDCCTEVAHHVPEKWLRRHVLRFDVQKGDLCKIPAVILYTKRKKLCASPHNKNVKRWVRRMSKNHQKAVNHGRKRRKTKRRRKTTRKL

>acaXCL1 blast search Chr 3

TMASQTMALSSCIPLQSTEINIRRIIDYIEQTRPVKAVILITRRGVKICVPHNLPWVKETIKKLKQKKTSKQKKITRPSAATS

>xtrCXCL8a XP_002942576.1 Scaffold GL173653.1

msakifaatfafcllytafsKGVYKIKTSEGLRCQCIHTHSAFIPPRLYKSVELIPSGPHCKNVEVIITITSGERVCVDPSQRWVQRIINSIIESNNNKELLTQIEDF

>xtrCXCL8b XP_002942577.1 Scaffold GL173653.1

metkrtllaimalcllcaavtesMSLTRIQELRCLCIKTESKPIHPKHIKNIEVIPNGPHCKNVEVIVTLTNMEEVCLEPSAPWVKKIIDKILASSKVPEPTPVA

>xtrCXCL8c XP_002942578.1 Scaffold GL173653.1

metkrtvlailafcllcaavtesIPVSRTGELRCLCITTEKKPIHPKHIKNIEMIPKGPHCKNVEVIATLTSGDDVCLEPTAPWVKRIIEKILASSKPAAPVPVL

>xtrCXCL8d blast search Scaffold GL173586.1

IPVSRTGELRCLCITTEKKPIHPKHIKNIEMIPKGPHCKNVEVIATLTSGDDVCLEPTAPWVKRIIEKILAR

>xtrCXCL8e XP_002941815.1 Scaffold GL173479.1

mqsqrnslymigfilllshclpasygDSIMRELRCQCISTVSTPFHKKHIRSLEVTPNGPHCPRVEVIVTLRNGVQHCLNPKAKWLTTVVKKILKRNSQKK

>xtrCXCL10a XP_002940620.1 Scaffold GL173278.1

mavgkalalfcglllilscvcgMSPLGKQRCMCKGRGAERISTQHLKKLEVFPMSFGCDEIEVIVTMKSGSKICLNPQSKFANKLLSALRKKR

>xtrCXCL10b XP_002940621.1 Scaffold GL173278.1

mtpdkacvmivgllvilayvhgMSPGGNRRCLCKRHVAKRFDLRSLKKVAVFPISPGCENVEIIATLKSGQHICIDPESKSINKLIFALKKKGYNKAIG

>xtrCXCL10c XP_002940624.1 Scaffold GL173278.1

mdskyaiiilcvlilsaaliegQGTKGRRCLCKKMSKKLSPKRLIKIEIYPAGYRCENIEYVATMKGSKKTKCFSPNSKLLKEIMSPKGKLQSIKIIKHE

>xtrCXCL12 NP_001015764.1 Scaffold GL173630.1

mdirtlalfsillgslclsegKPVSLVYRCPCRYFESNVPKSNIKHLKILSTSNCSLQIVARLKHNGKQICLDPKTKWIQEYLEKALNKKVKT

>xtrCXCL13a XP_002935498.1 Scaffold GL172787.1

mmsmkhiavvsviallailhcvagLSLEPKLPGGRCKCFKQTNSFIKPSKLTRVEFFPPGRSCPQLECLVTLKNGDIVCVNPQAAWLQRLIAYLKEKSEKNRAVSPI

>xtrCXCL13b XP_002935506.1 Scaffold GL172787.1

LMVGRKCRCLKQTKKRPSSIHRIQVFPESYKCRTQEVLVFLKNKQIVCVDPEARWIQVLIFNSQKERGEKN

>xtrCXCL14 NP_001107714.1 Scaffold GL172813.1

mrpiiaallllliavctlhvegSKCKCSRKGPKIRFTDVQKLEIKPKYPYCEERMIIVTMQNVSRFRGQQYCLHPKLHSTKKFLKWYTIWKDKNRVYED

>xtrC(X)CL16 XP_002940089.1 Scaffold GL173192.1

mkfparmwirlllvcllsvhsavaQFGANAGACCFDMKPRDPPTDALFRLYKEKVKGFEECPHYRVQFKFDKGKICASKHDAWVEKLICHLKKDSYFCAKHGDSGSRPDFSKDSKKVPTTIPPAPDIAGDSKKPPGPAVTQRAPNANTQKATTAPSGTEAAPTEQAAVPGDYPVTPTQYSAVAKEQDKERLENPETIDKETGVSSSMKTAIISLVFISLFLVALVAFLICRRRRKSESATRAETANLTPKGQQETVQTNSQKSFA

>xtrCXCL18 XP_002939600.1 Scaffold GL173138.1

mklsaclvlssllcvisvlqqadtELYFAVPRCQCHQTINEIHQKHIKSFKINIPYEYCKFTEIILTLKDSRDVCLNRQAKMGRNLIRCWKRHSSIDKCRKNVFKRKNSKGQRKPKTDSGGST

>xtrCCL5 XP_002933594.1 Scaffold GL172697.1

mkatliilsvflfvafyngaesAPGYTQRNTETFNRRIDDFSKPTQIGSDVVSCCFDYIKKPIPPKHVADYFYTSSRCSRFAVVLVTRKNRKICANPEDEWVNNIINVLEVPETQS

>xtrCCL19 XP_002943996.1 Scaffold GL174658.1

TGANINPVDCCLRTNNKQIRWQNIRSYFRQDESSGCKIEAVVLITRRNKHLCTPPHEAWVQAITDRLDQKKLKHRN

>xtrCCL20a XP_002939967.1 Scaffold GL173173.1

malswnllrlhvllaglvfiicisqasgDYDCCTSYTQKKIPQKIIKGFYIQRSSEVCDIDAIVFEVAYKSPGNRNVLKSKLCADPKQKWVESRIEELKNKALKMKIQKKARKWKRIKKQNKIWI

>xtrCCL20b XP_002939968.1 Scaffold GL173173.1

msrarylcvlclvalglfcltstgegVMYDCCYTYTRKQLPLKIIKGFTIQNSFEVCDIDAVIFITRKFRVCANPKEQWVINATDAISKRKKKTPQISKE

>xtrCCL20c XP_002935080.1 Scaffold GL172761.1

malswnllrlqvlliglvfiicisqasgDYDCCTSYTQKKIPQKIIKGFYIQRSSEVCDLDAIVFEVAYKSPGNRNVLIKSRLCADPKQKWVESCIEELKNKALKMKIQKKARKWKRVKKQNKIWI

>xtrCCL25 XP_002937977.1 Scaffold GL172963.1

mhlrlvvlvtciyfcisgttgDFENCCLSYAKVTQYSGLYKHIRYYQLQEISESCNMRAVIFYLKKRIICANPMEQWVGLAIKQFHKMKYPKHQQMKAMYSG

>xtrCCL28 blst search Scaffold GL183952.1

FFSGSISCCTEHSDQISRGLLQRVRKFEIQQNNGLCNIRAVVLYTKHKVLCANPDNVLLNQWIEKRKGKEKKKKNISWGSKKKRKGKKKRGKSIKRKNRKPTKKDKKKSVRNHPQQINQGQAPPRGF

>xtrCCL34 XP_002939963.1 Scaffold GL173173.1

mssvktmlcaallitsivfvtcDFGKIVSCCTRVSSRKPKDVLVNFLIQKEDLPCVEAILFTTNEGKFICSRPKAPWVSKKMKEIEAMKETTVNDN

>xtrCCL42a blast search Scaffold GL172807.1

GASSTSCCFKFAKKLLLGRIQSYYPTSGSCPNPAIVFVTKNGKVCAKPNDSWVIDYQNRLDRQSR

>xtrCCL42b XP_002935843.1 Scaffold GL172807.1

mqisvaalslllltaccsqvhcGRGFLPTSCCFKFIKKWLSPKHVKSYYNTSSFCPHSAVVFTTKKGIKMCAKSSDKWVTDLIIQLPAN

>xtrCCL42c blast search Scaffold GL172807.1

mqismvalclllltacctqvqcGPGKNFSPTSCCFHYAKKRMSVGLFKDYYITSSFCSNPGVIFRTNNGNKICAKPSDKWVTDYMTILDQNAFSL

>xtrCCL42d blast search Scaffold GL172807.1

mqismvalclllltaccTQIQCVPGNFSPTSCCFNYAKKRMPIGLFNDYYNTSSSCSSPGVIFRTIKGHEICAKRSDKWVIDYMTRLDQNNLNL

>xtrXCL1 XP_002939964.1 Scaffold GL173173.1

mkllfmalliglgmvlltaetqgQGGELIFGKICLETKLMNKIKCKMLKSYVQQTSPVAAIMFTTQKNITICANPEQPWVKQAVQCLKTKKPRGSNPKKRTKKKKNGKAKSKSTP

>xtrXCL2 XP_002939969.1 Scaffold GL173173.1

mkllhlallicfgmdllmsiqglegEVIRGPVCLETSSPKPLSFILIKDYIEQTNPIKAIMFTTKKDKLICANPEENWVKKAVQYLKRKAKRRTNLTEVTTAKEE

>olaCXCL8aa ENSORLT00000006430 Chr 1

mttnkviilsvvflgfiaiaeaMSLRSLGVELHCRCIQTESKRIGRHIQKIEMIAPNSHCEETEIIATLKTGQEVCLDPEAPWVKKVIQKILSRYESKRTKDMHRKAS

>olaCXCL8ab blast search Chr 1

mfptvfvtllvvlklqegTGVGVQGINLRCQCITKEKTPIGRLIGAVEVNPASSHCKEVEIIATFAKNGKKICLDPEAPWVKKVVNAMRKLPGWTP

>olaCXCL12a ENSORLT00000011349 Chr 15

mdvklfaltaaltmaiyapqthaKPISLAERCSCRSTVNNVPRSFIRELKFIHTPNCPFQVIAKLKTNRELCINPEARWLQQYLKNALTKMKKAKQII

>olaCXCL12b ENSORLT00000018951 Chr 1

mdakllalmalltvathtptskaKPISLVERCWCRSTLNTVPQRSIKELKFLHTPNCPFQVIAKLKNNREVCINPATKWLQQYLKNAINKVKKSKRRNGQNM

>olaCXCL13 blast search Scaffold 982

mkkavllllilccfcitslhaFSRGGCICLRVSRSAVPAKAVSKVEMIPPNGRCRKTEIIITRKNNVRVCAVPQAKWVQDLLRTLNQCKESSSSTATPTTVSTTTDHHRSLFF

>olaCXCL14 ENSORLT00000000239 Chr 10

mgrctvliillvvsfyvlsaeaYKCRCTRKGPKIRYKDVQKLEIKPKHPFCQERMIFVTMENVSRFKGQEYCLHPKLQSTKNLVKWFRIWKDKHRVYEA

>olaCXCL18a ENSORLT00000011381 Chr 15

mklnpqlicqlallsllcllqaVRETDGVYVPGRCLCPQTIPGVRGRLKELLVLPRSASCNTITVIVTMMNNARVCLNPEQPMAKQLIRCWDRSERLGLDKKRCLKRRRVRKGGRQQRQRQRGQALGTNISSSASQ

>olaCXCL18b blast search Chr 1

mglakkdgalllavvaavciqlyqaQHVVGRCRCYEEVRLMAIKRNITDFQVKEKSAACSKIQLIVTFMEANSTAVERCVKPQGYKAKQLLKCWEGINKNESRKMECINH

>olaCXCL19 ENSORLT00000009834 Chr 18

mklysllllgtlvvlidgIPPINRDYNTRCQCLKVESRIIPPDSLKSIKLVTEGPHCPETEVIAGLVTGEKVCLNPRSAWVKKLVQFVLERQLNHQKAPAKNQA

>olaCXCL20aa blast search Chr 18

SECHCLRTSKTVQPSLIKKVQEFLPRPYCSKLEIIVTLKNNVKVCLDPTHKFAKAVLQSIKV

>olaCXCL20ab blast search Chr 18

mklviqsiillvcgvacnsAILKCHCLRTSKTVQPSLIKKVQEFLPRPYCSKLEIIVTLKNNVKVCLDPTHKFAKAVLQSIKV

>olaCCL19aa ENSORLT00000021081 Chr 9

madakmllccltiicccsvamaQMPVDCCLSVKNQTIDKIVVADYYPQAKGCALDATILVTRRKKTLCVPHDEQWVQDVKKHVDRLKRRCKENGYKARRCFGLKRQ

>olaCCL19ab blast search Chr 9

masrvaalillgllcaglaaaQVPKDCCLSIRQEKLNPKNIVSYSIQEEGDGCQISASVFVNKHGKKLCVAHPKDFPWAQKIMNLVDKRKPASQ

>olaCCL20aa blast search Chr 17

mairgiaaasllcfllgmlcpapadsARSNLSCCTAYNKMQLPFQRIRGYREQTSNYCHIEAIIFYTINNRQFCANAKETWVKHSLELLSTKLKRMSKILPGPEKAVMEKPKNPELSKTLEVIPETFHNKTESV

>olaCCL20ab blast search Chr 17

mmalmghssvmvcteyplmiakalaKLCCTRYQSTGNHFPVQRLKSYRVQEDTGNCNIRAIIFLTVKGRVFCANPNQQWVIEAVETLQR

>olaCCL25a ENSORLT00000022583 Chr 17

mrfhalslllilccvclaatQVSYDDCCLKYVRKLSRGTQKHAIAYKIQKADGGCNISALVFIMKRGRMICANPNETWAVTLKENLDAKSAKQIPRDSRKLRPLSRKG

>olaCCL25b blast search Chr 4

mkfhtlvlllfftcmylslaqgSYDNCCLGYVRELGPRKRNNIVSYRIQETDGDCNIRAVVFLMKKRRGLAKQRTVCANPEDPWVQEKMMDLLMRN

>olaCCL27 blast search Chr 9

mdlkgvflslclcalvvtcthgCCYNIGQFQPKKTQRITSIGIQHASGRCQINAVVVHLDGRETPICVHLKREKQIRKLKRKQDMEAQRAQKNQMKTKHFKEKHKSN

>olaCXCL32aa blast search Chr 17

mqftllsstllcviawmnsiqavhghVHDCKCVRPRDTQIPKTKLVSYTIQEESICNVRVVIFTTVKGKILCSDPNSSWTKEGMRTLDVKKAKMEAQRKEAEHNKTPAASTMNPTLTPQTVSTAIPSVSTSTPSVSTVAPTETTTEAAAATSEASTLKPTAAPSSAQTLSSCQKKQKEQNRGNRRHRKSGRQGRNGRKNKGRMKEWQKKNKARKQLQTIFRCKQMLPN

>olaCXCL32ab blast search Chr 17

mhfgllsatfffcttwmnlvhaFIGPMKGCECLRWYDTKVNISLIKSYTIQEETKCSVRVIKFKTVRGKTICSDPNNKWTKNTMKKLDLQKSALVERQNMRIESGIAPTKSALTHRPSTQKPGAVSAAMHLLSTLRPKAQRMTSPASSLISATPTLTPATATSLLAPGASPSAPGASPSAPGASPSAPGASPSAPEASPSAPGASPSAPGASPSAPGASPSTPARSTSPTPKHTIFTPTPTESV

>olaCCL33aa ENSORLT00000019157 Chr 6

mktlcfslglllfvvccsdaMPFGVSASTGPGQCCFDFKNIPPQKIVKDIIQTHRLCSKNAFIVIPVKGKPICMKDDVEWVKELFNLKTSAGSK

>olaCCL33ab blast search Chr 6

mktlcfslgllllvvcfsdaMPHGATNTVGPEECCFSFSTKDNFSENRVTAIIRTHPHCSNKAFIVSTVAGKKICFRDNSKWAVDTFNKFNPEGSG

>olaCCL33ac blast search Chr 6

mkmicfplclllltaccfdaSPVQHDFEESTICCFKFHNLQPPLGRVVNVTKTHACRIQGF

>olaCCL33ad ENSORLT00000019160 Chr 6

mktlcfslglllfvvccsdatahgATNTAGPEKCCFKFSERQLPLKRVTGIIRTHHSCQHQAFVVSTVTGREFCFRDNFKWAVDTFNGIRPEGSG

>olaCCL33ae blast search Chr 6

mktkmkmicfplclllltaccfdaWPMQHDFEESTLCCFKFSKIPIPFVLVVNVKKTSASCPIQGFIVETRKRKICVSKTSIWGHNFFNRVYNLENIGDEDLQH

>olaCCL33af ENSORLT00000019162 Chr 6

mktlcfslglllfvvccsdaGPVGFHHSTVLEKCCFEFSEEPLELRRVTGIIRTPQRCQHQAFIVFAIGREEFCFRDNFDWAMDVCKYFYPEGC

>olaCCL33ag blast search Chr 6

mktlcfslglllfvccsdaMPTGINHSTGPEKCCFVFSENPVPPKKVTAIIKTHHSCQHEAFIVSTEAKKEFCFKDNFQWAVDTFKKLTSEGSG

>olaCCL33ah blast search Chr 6

mktkmkmmcfplclllltaccfdaWPMQHDFEESTLCCFKFSKIPIPFVLVVNVKKTSASCPIQGFILEKAKICVSKTSIWGHNFFNRVYNLENIGDEDLQH

>olaCCL33ai blast search Chr 6

mktlcfslgllllvvccsdaMPVGVSDHGPCCFEFREKQLPPKRVTGIIRTHPRCQHKAFIVSTVRKEFCFRDNFQWAVDTFNKFNPEGSG

>olaCCL34a ENSORLT00000018268 Chr 17

mqlamrklaclalfagvllvlssaTEMKVKSCCTKLSIANISAPIIGYRIQKKNLPCIRAVVFETTEGEICSHWKQDWVYEKIMELEKIRKEKMTDSKTKPSTP

>olaCCL35a blast search Ultracontig 72

MRGAGPKKCCFRFNETPMEKENVVAYMKTSQRCSRPAVLLKTAAGRQLCVRPSAPWVKEVISYLDTMEQSSSM

>olaCCL36aa blast search Chr 18

mrtaailllcilgaalfctvscNNSNGPDNCCFKFFPGTLSASRVRSFTLTDDRCPKPGVILETKKKINICVKQNAAWVQEILQMLVGPARPPASAHTS

>olaCCL36ab blast search Chr 18

mrtaailllcilgaalfctvscNSSSGPDNCCFKFFQGTLSPSRVRSFILTDDRCPKPGVILVTKRNLNICVEQSATWVQDVLQTLMGPAPAHGFCRFDRRNVT

>olaCCL40aa blast search Chr 3

mktlltlgllvlacslshsssSPVGLELLLKDGCCHQPQKINIPKTKVKHVQMTPSGCSTKAIIVTSKLERKFCLDPDWAPAKKLLQKFEESLSVSPKP

>olaCCL40ab blast search Chr 3

mktlltlgllvlacslshsssSPVGLELLLKDGCCHQPQKINIPKTKVKHVQMTPSGCSTKAIIVTSKLERKFCLDPDWARAKKLLQKFEESLSVSPKP

>olaCCL44 ENSORLT00000021360 Chr 4

mlvlqmltvlsftvvflasveaKGVQMQRDVQCCMLYSQGKVRTKDVLRFEVQTEGPDCSIQAIIFYTKKAVKCADPRDRKVKRLLRKLQQRQRTKAHRTPWFFRHDNLPVMSEDKKDNWAILNVE

>gacCXC8a ENSGACT00000002257 Scaffold 882

spcrvtalilfssflriilrmslrslgVEQHCRCIQTESKIILRYIEKVELITANSHCDEAEIIATLKKTGQQVCLNPEAPWVKKVIQRILAKRAPCP

>gacCXCL12a ENSGACT00000009213 Group VI

mdmklfavvavltlvtyappsqaKPISLVERCYCRSTVNSVPKSFIRELRFIHTPNCPFQVIAKLKSNKEVCVNPEIRWLQQYLKNVINKMKKSKQGN

>gacCXCL12b ENSGACT00000024828 Group IX

mdvkllalmalmavathvptsnaKPISLVERCWCRSTLNTVPQRSIKELKFLHTPNCPFQVISAKLKSNREVCINPETKWLQQYLKNAINKGKKSRRRNTKKN

>gacCXCL13 blast search Scaffold 834

FPTRGCRCIRTTSDPVPTRVIRRIEVVPVSGLCRRTEIIVTRRNGSKLCVNPDEAWLHVLLSKPQHNSLIST

>gacCXCL14 ENSGACT00000021780 Group IV

mhrgaavlllvlvavyvlraeaYKCRCTRKGPKIRYKDVQKLEIKPKHPFCHEKMIFVTMENVARFKGQEYCLHPKLQSTKNLVKWFRIWKDKHRVYEA

>gacCXCL18a blast search Group VI

mkldprpvctlaflslcfilttvresdgTFVPGRCLCPHSQPGLRGKLKALTVYPKSPSCDRLTVIVTLQSTNESVCLNPEAPMGKQLIHCWNR

>gacCXCL18b blast search Group IX

mapsqkrcilllavmaavshdflgRCSCLNPIMFIKGSISDFEVLESRPGCDKTELIVTMNNPNNSTEKLCMNTVGKRAKAFLRCWERINKDASRKTECIDRKKKAE

>gacCXCL19 ENSGACT00000025278 Group VII

mklsvllvfgtllvlvygMPPISRDYNTHCRCLQVESRIIPPDSLRSIKLVPEGPHCPDIEVIAGLADGRKVCLNPRAAWVKKLVHFVLERQLHQQGQALSKNQG

>gacCXCL20a ENSGACT00000025280 Group VII

mntatrciillacaaictsNSPILNCRCVKNSDAVSRHLIARIKQLPPRWYCNREELIAVLKDGREKCLAPNGRFAQAIKRYRTQRAMTRKTSTTGPKTTAA

>gacCCL19aa ENSGACT00000018927 Group XIII

mtscgdaklffcilfvtycctvtfsQIPMDCCLRVGNNTIAKQRVVNYFLQIGGQGCAMDAMVLMTRRKKNLCVPADEPWVKEVVKHVVALKEHCKRHQYKGRRCYNVKQK

>gacCCL19ab ENSGACT00000018929 Group XIII

masstaallllgllcvgfasaEMVVDCCLTVAEKPLPLQILRSYTIQEAGKGCSISATGFVTKLGRTLCVSHPNNKLWVRNYIKYLDEKRAGRR

>gacCCL20ab blast search Group III

mvsirataavlallsacllvtdvsaVNYGCCRSYMTSRIPFSRIKGYSVQTMKEMCSINAIIFHTMKGKGCTDPALNWVMQYVNRLR

>gacCCL25a blast search Group III

mrsntvifllvlsgvclslaQTTFEDCCFKYVKKHIRRTQKLAVDYRRQVVDGGCNIPATIGGRLLCADPTRKMVVHLMKRLDKRDRHVHAKLKTVGDFSSACIVKLSIYSNHVRPYLISNTMSLKISLQFPTHVLVFHIPICSSPTALQKLKGHRPAAAAPGRLSSAKSYC

>gacCCL25b ENSGACT00000014866 Group VIII

mksqalvlllvlsctylclaQGSYGNCCLGHHTRIAERVKKNIESYRMQETDGDCNIRAVVFLIKKKPSHAKQLTRCANPDDLWVQKLMKTVDVKMKK

>gacCCL27 (ENSGACG00000006689) Group XIII

melklplvlfclcalaitsteaAIPKCCIRTKNHIPLQMLRKVQRWSMQSGGRACDISALLLHVKDMRTPVCAHPKVLGILKKVRQQTIRNKQK

>gacCCL32a blast search Group III

mtfsrvlaallcftawvsvvhaTHASVSSCCLGWSTRKVPPRCVVNYTVQTDAACSINAIVFRHINGGRICSDPNSDWAKQVILKVNREKRKQSSLQENGQNEDGTTSAVSPAVSLPSKTTLQKTSRNRRGCQRKKPRAGKRGQN

>gacCCL33aa blast search Group XIX

QAVEQMSHGVCCFTFSTVTVPKNNIVSVTKTHHSCPVKAFVVTTVKGRQICVGHYVNWAQKAFKQQKVTEG

>gacCCL33ab (ENSGACT00000000554) Scaffold 163

mrtlsctvglllsvsiycciaMPQAVEQMSHGVCCFTFSTVTVPKNNIVSVTKTHRSCPEKAFVVTTVKGRQFCVGHYVNWAQKAFKQQQVTEG

>gacCCL33ac (ENSGACT00000000554) Scaffold 163

mrtlsctvglllsvsiycciaMPQGVKQMSPVSCCFEFFTGRVPSKQIVSVMETHSSCGAKAFVVTTAKRRKICVGHYVNWAQEAFKKQNVIEG

>gacCCL34a blast search Group III

mqsyirrlaclalvagvlaitasaSEIKIIKCCTEVSVANVTAAILGYRVQRKKPPCVKAVIFETTDGEVCSHWKQEWVFAKVKELE

>gacCCL35a ENSGACT00000002970 Group XIX

maaarltlsvlvlmlaaaslteaGLRGAGPKRCCFRFNENKVPSNNVVGYTKTSQRCSNPAILLQTVTGRKLCVRPSAAWVKELINAKFVQGERSNLETRLI

>gacCCL36a ENSGACT00000026565 Group VII

mrttnilllcilgcaliasaigNSGTGPDHCCFTPYPRRLNKKLIRSYYMTDHRCPKSGVILITQKGRHICVDPDLSWVGNIMSSVDGSTF

>gacCCL40a blast search Group II

PVGPSMLNNGCCVSTSHVHVPRGKVIHIGMSSRECPVTAIIITTEKRQFCIDPDLKWAKTQLAHFKASTTSISHPKHQPTIRSFVHRDDKQNTTESPK

>gacCCL44 blast search Group VII

mftpqmltvisltvillasvegKGVQMQRDVQCCMLYSQGKVRTKDVLRFEVQTEGPDCSIQAIXLYTKKAVKCADPTDRKVKRLLRKLLLRQRTKDHRTMWLLPRDNLPVMSEVRDTTNRTAFNNKKDNWAVLNVE

>dreCXCL8a XP_001342606.2 Chr 1

mtskiisvcvivflafltiiegMSLRGLAVDPRCRCIETESRRIGKHIKSVELFPPSPHCKDLEIIATLMTTGQEICLDPSAPWVKKIIDRIIVNRKP

>dreCXCL8ba XP_002662787.1 Chr 7

mklsisafmllicttalqcTNEGQPPPPPLRCQCVKIYSQPPIPRRQVLALKVNSAGPHCRNEEIIATLKNGQTCLNPTENWVMSLKTQGYDLPALKTEFKWIEDSYVLPVGYEILVVFELSRF

>dreCXCL8bb blast search Chr 7

mmklsvsafmllicttallcanegeaLPPPQRCQCIKTHSKPPIPKRQVLGLKVTPAGSHCRNEEIIATLKKGQICLNPTETWVISLKEKFAASATKLAATAAPAQTTTTFSTIMTTN

>dreCXCL8bc blast search Chr 7

mrcsvfvflacmtllsttevfaARLPIQQLRCQCVKTYKGKPINPKLIQSLQTIPAGARCKNMEVIATVKNGKTCLNPKDEWVTKIIEGRSVKAPTRGPIITLPPNSTSVPQLTSKM

>dreCXCL11aa XP_001339307.1 Chr 5

mktvtalllvslavvaiegQHMKSQRCVCLGAGLNMVKPVLIEKIEILPSSPSCGHMEVIATLKNGAGKRCLNPKSKFTKKIIDKIEKNNRNAR

>dreCXCL11ab blast search Chr 5

QKKINRCSCVGKGLDRVVLRNIEKFEIIHPSPSCGKQDVTMKSSLQKCLNPEFKFTKPLIRRALEKILVF

>dreCXCL11ac blast search Chr 5

mktlaafllltcliagkvngQDNTSRARCFCADKGINMVLLKNIEKVEIFPPSPSCNKNEIVVTLKNGAGQKCLNPDSKFTQNVVLKAIGKRMQQSVPHSTTTGTVKSSMTSSTSAPTAFK

>dreCXCL11ad XP_696046.1 Chr 5

mktlaavvllgyllvikvegQARAPRSRCLCADKGVNMVSPKLIEKVDIIPPTPSCGNLEIVVTLKNGAEPKCLSPDSKFTQKYLMKALEKRTLQK

>dreCXCL11ae ENSDART00000135625 Chr 5

mktaaflvflacllatqvhgQKKFNRCSCVGKGLDRVALRNIEKFEIIHPSPSCGKQEIIVTMKSSEQKCLNPESKFTQELIRRALEKRVILTDEVSVVFSGSTSR

>dreCXCL11af ENSDART00000138213 Chr 5

mktlaaflllscliagevngQDRSSRARCFCVDKGLNMVLLKNLDKVEIFPPSPSCNKHEIVVTLKNGAGQKCLNPDSKFTKNVVLKAIGKR

>dreCXCL11ag ENSDART00000137224 Chr 5

mktlaaflllscliagevngQDRSSRARCFCVDKGLNMVLLKNLEKVEIFPPSPSCNKHEIVVTLKNGAGQKCLNPDSKFTKNVVLKAIGKR

>dreCXCL11ah blast search Chr 5

mktaaafvalgcflmvevkgKIPDLKNRCLCADKGANNVNLKTIEKIQIIHPSPSCKRLEIVVTLMKGAGKKCLNPESNLGKNILKALRKKKLTAVRRMNPA

>dreCXCL12a ENSDART00000053946 Chr 13

mdlkvivvvalmavaihapisnaKPISLVERCWCRSTVNTVPQRSIRELKFLHTPNCPFQVIAKLKNNKEVCINPETKWLQQYLKNAINKMKKAQQQQV

>dreCXCL12b ENSDART00000077411 Chr 22

mdskvvalvallmlafwspetdaKPISLVERCWCRSTLNTVPQRSIREIKFLHTPSCPFQVIAKLKNNREVCINPKTKWLQQYLKNALNKIKKKRSE

>dreCXCL13 blast search Chr 5

malrpslllavtavccftiiiecLYDCFSALPMDGFATENKCKCQTTTSSRIPPRLFQKIEILPAGAHCRKAEIIITKKDNQAVCLHPEARWVKEMVSKIISKRAERETAMPTVA

>dreCXCL14 ENSDART00000125923 Chr 14

mnrcstaalfllviaiyslnteaYKCRCTRKGPKIRYIDVQKLEIKPKHPYCQEKMIFVTMENVSRFKGQEYCLHPRLQSTRNLVKWFKIWKDKHRTFEA

>dreCXCL18aa XP_002664233.1 Chr 13

mafktlqasvkvllllsvcshfisvkmtaaTFIREKCECVKEAGAVQWRKITDYTITPKNPLCNKVQIKLQLSNKEVCLNPESKQGKKLQKCWQKINFNPQRKKVCLTIKKNAPKRLKKL

>dreCXCL18ab ENSDART00000108949 Chr 13

mtqiaytllalnlcfiltaqvvesQHVPKTCQCPQVQKRVRGPFSDLRITPKGPSCLQNEIIVTPKKTNKPVCLSPEGPQGKSLMKCWNRTQKAGINHKICLRPRQRKGKQVKSKKITS

>dreCXCL18b ENSDART00000111598 Chr 1

maftpkallllllavvyvqqgevlaKIPDRCQCEESSLVNRARRDTIKEFYITPKRPNCDKVEIILTQKPENKTTASGQLCLNPQKQQGQLLQNCWTRLNINNTDSLKMSVCWQ

>dreCXCL19 blast search Chr 7

mnvllmlsvvfgvsitlvagAVQPLGAGYNSRCVCLKLESRVIPQDNLRRVVILPRGPHCKTTEVIAGLTSGERICLNPRTHWVKKLIMFIEKKKQENNKL

>dreCXCL20b ENSDART00000112807 Chr 5

mkvsaclinqrllkkgnifpelpvrgsmnqivlillcallfgmslaQSVGHGGGGSQRCRCIGKPYKTVNPRSIQAVDVFQPSPSCSNKEIILTVVEGRGKTKGKGSRKRSKVCLDPNGKQGQRLLKGRWGKKQNQRNRGKKEKNKV

>dreCCL19aa ENSDART00000099611 Chr 5

massimsafclavsalllcfyssptvaQADLALDCCLTVSPRVIPKHVILAYQKQSRGDGCPRDAVIFITRKGLNLCAPPASEESWVRDTMTFLDKRREKCKETRFIERRCHALKFMKF

>dreCCL19ab ENSDART00000051668 Chr 5

mqtstitllliaavfcinteaFPDTAVDCCLTTKDTRIPLQIVASYFHQTTESGCPIAATVFITKKDKKLCAPPEKNTWISRIISHLEKKQRKALQ

>dreCCL19b ENSDART00000057509 Chr 10

mmlsnsivaaallilsvslwscttalgDDAVDCCLTTSDRRIPQKVVTTFTLQTGEGGCRVPATIFVTKKGLKLCAPFPSQNNWVSRLIDHILGREKPAQKRPRKSKGKKQRQQ

>dreCCL20aa blast search Chr 2

mslvsitlisivflsllphtpkaYGPLNYACCVKYTRTPLPFGVIAGFIEQSSLEVCRIDAIIFITQKNKKICASIEDQWVRAALAHLR

>dreCCL20ab blast search Chr 2

mslvsitlisivflsllphtpkaYGPLNYACCVKYTRTPLPFGVIAGFIEQSSLEVCRIDAIIFITQKNKKICASIEDQWVRAALARLR

>dreCCL20ac blast search Chr 2

msrisacvmvltivalgllctdaaaVSCCRKYTKGMIPMSLIKGYSIQTITRSCHINAVIFHTNGGKNICTDPSKGWVMESIRKLREKVQAINKKNSKA

>dreCCL20ad XP_002663056.1 Chr 2

mmsklvltssalllllcvcvnlsqsSPMRCCTMYSIHSLPLNRLMDYTLQDTTKACNIKAVIFTTIKNRQICANPDDPWVQKAISHIQMKNKSA

>dreCCL20b NP_001107067.1 Chr 24

mgnikictlyfivllsflvetesAICCLRYVKNPRRCGFLKGYDIQIMTEGCDLPAIIFHTVTGRSICANPSQNWTQERVLCLKKKAETMKTKTMSMFTTLS

>dreCCL25a ENSDART00000129782 Chr 2

mrfsifififllgfiylttaqgSYEDCCLKYVRKINHSMKNRVIRYRKQEVDGGCNIPAIVFTLKQGRMFCTDPREKWVHELMQRVDRLKARPYLKRKTSRG

>dreCCL25b ENSDART00000104405 Chr 11

metqsstmkfqilalllllacmypsiaqgYYENCCLKYVTGIKKNMRRNIMSYRVQLTDGGCNIPAVVFKMRLKKQLKPKSVCADPRSDWVQAIIKELDEKNKRAM

>dreCCL27a ENSDART00000081457 Chr 8

mefrsscllllvcftiiiltdnkgaaIPTCCLSVLRRIPKRVLRSVRTYEVQDTSGHCEIKALILHFKGKKICAHPKLERFLKKMLKHKPKKP

>dreCCL27b ENSDART00000110202 Chr 10

mdlivlgillciafsgaqgVTPRCCVETTKRFPLDLLKKVNRYEVQTSSGACTIDALVLHVGDMRYCATPKMEQFLQKLMKRMARLKASAV

>dreXCL32aa blast search Chr 2

mklhvsgfsavlllwllvsssvqEDAHKTGCLSTTDTKTPHTNLRSYTIQQKPLFPVHAVRFLTLKGITICSDPTSPWAIKAMKHLNGKKKQRQSNITIRPSVKVVHMDTSTTNMARVSAQLKKQT

>dreCCL32ab blast search Chr 2

mtrqiftacvlvvilgsisvfaDGPPMSCCLRLRDRKLHLDKILNYRIQTEDLCPIRAVLFQTVAGKTLCSDPESSWTKSAMWKVDEEQRKLRGQIPEAVEGASVDGCKGREDPMTTAEMPLNSRVLLTKMQTTKQKNSQTTAKAQKEVK

>dreCCL32ac blast search Chr 2

mkfnqfaaflfsiqwvifgNEQQVYGNTSPPGCCLTVKNIRIPAFNIVGYSIQEIPLCSIKAVRFYTKKNRVICSDPNSDWAKKVIQQLSPTPALKKALQCYTNSIQTTTATHKIPGTHSDTSTAVQPTVTIINTFEPETSSNINTFGPETSSKINTFEPETSSKINTFGPETNTNNYFCLKKEKKAIHFCIKQA

>dreCCL32ad blast search Chr 2

mnftlftavflcigwivevagNGRPANCCSLKDTKIPAENIVDFNIQEAPPCHIKAIRFYTRKNKVICSDPNSHWAKKMIEKLSLTKDTPKTPIQCHVTKAIQTTTSKTPGTQTETSTSTTVQPRVTVINTSGPETSTSTTTATSEAETSTSSTITTSRPETSRSTTAMTSKKSTKKSEPPPQTYLNTMTTCGTGELMTPAKEKKLSGNIVTSKTPAKRTKPSRLKLKARNKSKKEFRKLQMKKKPK

>dreCCL32ae XP_002667365.1 Chr 2

mtgqiftacvlvvilgsisvfaDGPPMSCCLRLKDRKLHLDKILNYRIQTEELCPIRAVLFQTVAGKTLCSDPESSWTKSAMWKVDEEQRKLRGQDPKAVEGASVDGCKGREDPMTTAEMPLNSRVLLTKMQTTKQKNSQTTAKAQKEVK

>dreCXCL32ba ENSDART00000105771 Chr 24

mrsalitllclavmllvqesyqvsssSNCPCLKLSDGVLRKANIKSYIRQRAGVCHIDAIVFTTVRGITFCADPKLTWVIDAMKFLDKKKAASEPKTTTQPINSTFNATSMPNTTANLNTTNTTSDLNTTNTNTTSHLNTTNTNTKAQTKRLFTTIQPC

>dreCXCL32bb blast search Chr 24

mkcvlitlficlttmllcKDSYQKRSSSYCPCLKTSDIVLRKANIKSYTRQRADVCYIDAIVFKTVKGKTFCADPKKTWVKDAMESLDKKKAAPGIKTTAQPIGSTLNTASTLSTTEDSAENGLDELQTELNEYSTSLFCFFFLFIVFSVFSPMHHIGIERTSLLFVCVSAATGRSFCPCLKTAEVVMNEEDIKSYKIHNADVCHVDTVE

>dreCCL32bc blast search Chr 24

msstigfpfclvlvllcynaatsVRLNCCLRTSKSSIPIKRVVDYRVQQPGICPIEAVILVTVKGKRICCDPNTEWIKKTMRKVDQKKLRKQNSDLKASPNPNNNINQRKRRRQN

>dreCCL32ca blast search Scaffold Zv9_NA228

mtgriftacvlviifrsisvfaDGPPVSCCLRHGDRRPHLDKILNYRIQTEELCPIRAVLFQTVAGKTLCSKPESSWTKSAMWKVDEEQ

>dreCCL32cb blast search Scaffold Zv9_NA228

mnfslftavflcigwiveisgIAHPVHCCTKVKNVRISVEKIVSYSIDEPPLCPIKAVSQAKQSYCSDPNSDWAKRVIYNLSPTTVTHKVIQCHTTKTTQTTTQKTPGTKIETSTRTATVQPTVTVINTSGPETSTNTTVTTAETYRPEYSRTTILHTSGLEMDDRKTTETTETHQQDPLNMVEEKQVTLATSVTLLAEVDDANKSTNYFCLKKEKA

>dreCCL32d blast search Scaffold Zv9_NA306

mkfnqfaaflfsiqwmifgneqqvygNEHPFLCCVTVKNIRIPACNIERYSIQKPPLCPIKAVRFHTKKNKVICSDPNSDWAKKVIQQLSQTPALKTALQCYTKSVQTTTATQKIPGTHSETSTTVQPTVTIIHTFEPETSSKINTIGPETSTSTTLKTIETSRAEYSQKTTIATSGLMEDRKTSTETTETDQQDPLSKVQEKQVTSLATSVKLLSKVDDAHKNTNNYFCLKKEKKAIYFCIKQT

>dreCCL33aa ENSDART00000108956 Chr 25

mripvfllflvftmcsiqlvpaMPAIPEFCCINFIDFPIPANKIVSAVITPSRCSSKGIMVTTPRTQFCVKPDEDWIKPIMEKQYKR

>dreCCL33ab ENSDART00000124148 Chr 25

mssppcpwmsttttlapattaITTRAKTTTLPKPKRQIDRSSIPGPAAVPITCCFAFIDFPIPYNKIVSALRTSPRCATKAIVVTTPRTQFCVKPNEDWIRPIMERKLQK

>dreCCL33ac ENSDART00000114049 Chr 25

mrassvflllgltvlmawtseaQPAIPEPCCFNFIDFPIPANKVVSAVRTVSRCAVKGIVVTTPRTQFCVKPDEDWIKPIMEKQQ

>dreCCL34aa blast search Chr 2

mphnqkmmmrlaaiavilsvmimetngQNRFVQCCTSVSTQKIILPITGFKLQIRNPPCVKAVIFFTTEGPRCCHWKEGWVKEKIQELKRFQVWEEKMNSTDSIPLSTTTL

>dreCCL34ab ENSDART00000124897 Chr 2

mqlnqkmmmrlaaiavimsvmimktngQNRFVQCCTSVSTKEITLPITGFKYQKRNPPCVKAVIFFTKEGEQCIHWNQSWVREKIQELIISMEKMNSTVSTPLKMNSTLSTLLRINSTISTALQMNSTLSTPLSTSSS

>dreCCL34ac blast search Chr 2

mphnqkmmmrlaaiavilsvmimetngQNRFVQCCTSVSTQKIILPITGFKLQIRNPPCVKAVIFFTTEGPRCSHWKEGWVKEKIQELKTFQVWEEKMNSTDSIPLSTTTL

>dreCCL34ad ENSDART00000113901 Chr 2

mqlnqkmmmrlaaiavimsvmimktngQNRFVQCCTSVSTKEITLPITGFKYQKRNPPCVKAVIFFTKEGEQCIHWNQSWVREKIQELIISMEKMNSTVSTPLKMNSTLSTLLRINSTISTALQMNSTLSTPLSTSSS

>dreCCL34ba blast search Chr 24

mktqkifmrslavaltasviwtvtvaDNVESCCTPVSTPELTDPIMSVRIQFESLECETAIVFKTEERELCSDPRQLWVRRKVMQFYKNKVTKKTN

>dreCCL34bb blast search Chr 24

mskdshlklgccaiasviwcitidaKTVMPCCTSVSTAEVTDPIISVGIQRESPPCGTPIIFETKEGKICSDPRHEWVLRKVVQFLTQINSPTSPLPPLSSTTSNE

>dreCCL34bc ENSDART00000041770 Chr 24

metqkilvlnwavvliasviwcittdaKDMMLCCTSVSRLEVTDPIIGFRIQRESRPCVNAILFETERGAFCSSPRQPWVRSKVMQFLAQRNSPTSPLPPLSSATSNEKPTITKPDESKGVQFPGSSFPAASPKSTVSSMTAS

>dreCCL34bd blast search Chr 24

mawtarlltiavlialmgcftgaqaNYRRPTRVGVTCCKEVSRGRIPPDIKLTAYKHQPALSPCVDAIIFYAEKERYCTDPKARWIQNRLQGLKELND

>dreCCL34be blast search Chr 24

mdkrilmrsfaivvivsaiwtvtadaERVINCCKSVSTVKVTDPIIGVRIQRKSLPCVNAIIFETDRGHFCSDPRQPWVQRKAQQFIRNLKISQQTSTSLPTSSISERNVEGSAKAPDRL

>dreCCL34bf blast search Chr 24

metqrilvrsltivviasaiwpvtdaiEEKGTNCCKSVSTVEVTDPIIGVRMQHQSLPCVNAIIFETDRGDFCSDPRQPWVRRKVKQFIRNFKTNQQTSTQTLTSTPTLTSTPPLTFTPEPTSSISEQTNGEGYA

>dreCCL34bg blast search Chr 24

mrlfsasyqmiissalllllcaftsgDHLLQVAHPVLTQDATDSDPKEPVCCKTLTTNEPQIKINSCYFLQETSDCLKCVLFVDDMNRMYCIDLTAPWLSERIKCLEEDGVQCINKPNNAYIGTGHL

>dreCCL34bh blast search Chr 24

mrlssafhqmiilsallfllcaftsgDHLLQVAYPVPTRDSRFDRKDTVCCRTLTTNEPQIKINSCYFLQEISKCLKSVLFIDVKNKMHCIDPTAPWLEARIKRLEENGVKCIKTKAH

>dreCCL34bi ENSDART00000110748 Chr 24

mhlstashqliissalllllcasasgDYFMPVMSPVTPPESAPMDLRGMVCCKKFTRKEPQIKINSCFFVQEISNCLKSVVLIDEMNKMHCIHPKAPWLNARIKRLEEIGVQCTVH

>dreCXL34bj blast search Chr 24

mdpssasyqmiilsclflllsaFTPDDSHISQNNGLQAKKAVDQKGKICFRNPTTEEPQIEIRACFNLRKTRHCPACVLFIDKKNRMCCINLKAPWLSAKIEHLEKKGIKCKNKH

>dreCXL34bk blast search Chr 24

mhlssvshqmvisshlllllyaftsvvfisvsegWSSTDKNFDNRPGVCFKVLTTKEPKANIKRCYNLPKTNNCLKCVLFVDASNRMKCIDPNASWLAERLYRLKEKGVTCRGEA

>dreCCL34bl blast search Chr 24

mrlssashqlillsclllllsavtsRVMKNLKEMYLTATYMAFHNPKPSTCCEKHETNLPKIQLKKCLILPATDKCLESVLFVDIRNRRHCFSTTAPWINERIAIFEKKHGKCEKKY

>dreCCL34bm blast search Chr 24

mrlssashqlillsclllllsaftsgGLVTMFIFCHYCYNLISHDSKPKTCCRKYEKNAYFKIQRCYILPETDKCLNSIVFTDPNNRNHCYDPTAPWMTIRMNLLKKNNIPCKDYTKS

>dreCXL34c blast search Chr 12

mnvllmrlssashqmivssglllllcvftsgvfipesDRIQDTSSPNKNVDARPSLCFQVLTTVEPRKNITSCYNLSKKGNCLQCVLFVDAENRMMCMDPNASWLPARLNRLKAKGVTCKEWS

>dreCXL34d blast search Chr 7

mrlssashqmiissglllllcvftsgvFISVSDRIQGRSSSTKNYDARPKLCFQVLTTEQPKANITSCYNLPKTSNCLECVLFVDATNRMMCIDPNASWLSERLNRLEAKGV

>dreCCL34ea blast search Scaffold Zv9_NA2

metqrilvrsltivviasviwgvtdaiEEKGTNCCKSVSTVVVTDPIIGVRMQNQSLPCVKAIIFETDRGDFCSDPRQPWVRRKVMQFIRNFKTNQQTSTQTLTSTPTLTSTPPLTFTPEPTSSISEQTNGEGSA

>dreCCL34eb blast search Scaffold Zv9_NA2

mdkrilmrsfaivvivsaiwtvtadaERVINCCRSVSTVKVTDPIIGVRIQRKSLPCVNAIIFETDRGDFCSDPRQPWVRRKAQQFIRNLKISQQTSTSLPTSSISERNVEGSAKAPDRL

>dreCCL35aa NP_001122280.1 Chr 25

mstsrfvflsavvvllcavslsqgMRIGPKRCCFQYAERQVPFKQVMEYSMTSQQCPKEAVLFKTARGRYVCARPSDPWVQEYMQAIDSKRVGNQGTL

>dreCCL35ab ENSDART00000103203 Chr 25

mstsrfvflsavvvllcavslsqgMRIGPKRCCFQYAERQVPFKQVMEYSMTSQQCPKEAVLFKTARGRYVCARPSDPWVQEYMQAIDSKRVGSQGTL

>dreCCL36aa blast search Chr 7

STGTDCCYNFFKRKIPLSKINSYSLTRVDCTMPGVIFVTQKGLRLCVEPKLNWVKKTIQIIDDSNI

>dreCCL36ab blast search Chr 7

mghycvyllvgllaitflqaDVMGNHANTPDACCFTFFKRKIHPSKINSYNPTRVDCTLPGVIFVTQKGLRLCVEPKLNWVKKTIQIIDDRNI

>dreCCL38aa (ENSDART00000061434) Chr 20

mrrscifiaslvlvafcsvvgSDWSQWSQGPEKCCFSFTNARIPLKQIESYYTTHLQCNMNAVIFIIRAQREICTNPTEKWVRRLMKMVDNQNMKQMTEAGSVDSA

>dreCCL38ab blast search Chr 20

mrsfcifiillalvalcsaVSQIECCFSFSTVRIPVNQVQSYQTTHFECHKKGIVFITKIQKEICTDPTEEWVQRLMGLVDARYILQTTKSGLVDTHRSKPLHEMPETIPETDAMAKTAALIKTTDMPLDETTLTSILKESQDWRDVDTTQENSKTQSAYFCM

>dreCCL38ac (ENSDART00000061432) Chr 20

mkpscnfiaclvlatfclvnggwSQGPVKCCYSFFNARIPVKEVGGYHATHLQCNINAVIFITKAQREICTNPAEKWVQRLMRLVDVQNMKQMTEGRIGDSLDTHRSKPLHEMPKTNQAVSKKAEIITMTEMPLQQQDETTSILYYFQDWTDFDTTQANSKTESVYFCVRPGGE

>dreCCL38ad (ENSDART00000061432) Chr 20

mrqscifiaclvlvafcsvdgSDFSQNPDKCCFSFSTIKIPVKQVQSYHTTHFQCQKNGIIFVTEQKEICADPTERWVQRLMNLVDARLVKDTEASSNGSP

>dreCCL38ae ENSDART00000061311 Chr 20

mrpscisivclvlfafcsvdgSDLSQSPDKCCFSFSNTRIPVKQVESYHTTHLLCSGNGVIFITKAQREICTNPTEKWVQRLMKLVDNQNMKQMTEAGSGDSA

>dreCCL38af ENSDART00000061437 Chr 20

mrtscifiaslvlvafcamarsEWSQSPDKCCFSFSNARIPVKQVVSYHTTHLQCNMNGVIFITRAQKEICTNPTEKWVQRLMKMVDNQNMKQMTEAGSADSP

>dreCCL39aa blast search Chr 25

mrslmfllvlvlfcslqdtssaMEATISANSVCCEGFTHKKIPLSKIVSHHLTTSNCAKKFIVFTTKAGKKICVDPENTFVKRQVAELDSRTRV

>dreCCL39ab (ENSDART00000129113) Chr 25

mrslmfllvlvlfccvqetscSPIPINSAKSVCCEATTHRNIPLKQIMSYQWTTSMSYKSHCVGTIAGREICVDPQNTLVKKQVAKLDKRRSTTALSPKSTSTTAETPAATSGTTSSSESTSAKSSPASTSATALSHEYTSASTVTFTTAESHSTSV

>dreCCL39ac blast search Chr 25

mrslmfllvlvlfcslqdtssaMDAIISANSVCCEGITHKKITLKQIVSYHWTTSSCAKKAIVFTTKAGKKICVDPENTFVKRQVVILDSRAKV

>dreCCL39ad blast search Chr 25

mrsllfllvlmlfcylqaTSSSMEAINSEKSVCCEGFTHKKIPLKQIVSYLWTSSNCAVKVIVYDKSRKKICVHPENNFVKRQVVILDSRAKV

>dreCCL39ae XP_002666849.1 Chr 25

mrslmfllvlvlfccvqetscAPLAMNSAKSVCCEATTDKNIPLKQIMSYQWTTSTCPIKAIVFKTIAGRKICVDPQNTLVKNQVAKLDKRTSSTTALSPESTSTTAETPAATSAAASSSEFTSATSSQASTSATALSHESTSATSSPESTSVSKSSPESTSAKSSPASTSATALSPEYTSASTVTFTTESHSTSDQSAVI

>dreCCL39af XP_001338140.1 Chr 25

mkiimktallfavlccallpqpsdgQESADAANSMCCFGKGSNIKIPLRRLEYFYWTSSRCPLKHVVFVTIAKKHLCMNPDNEWVQKVINMKSGSGSSV

>dreCCL39ag blast search Chr 25

mktpllllvcvvmlcslpdsssgQESIDAGNSICCFGNSNSRIPLKRLKYFYWTSSHCPFKHIVFVTIAKRHICMNLDNEWVQKVISMKSVSGSPV

>dreCCL39ah blast search Chr 25

mktpllllvcvvmlyslpdsssgQQSIRKGICCFGKGSNRRIKLNRLNSYYWTSNFCTLKRLVFVTTTKRNICMNPENEWVQKIIKEKLILDLSI

>dreCCL39ai XP_002666850.1 Chr 25

mkiimktallfavlccallpqssdgQPYSGDASTSICCFGKGSNSKIPPNRLKSYYWTSSICPFNHIVFVTAKRHICMNPENKWVQKTMKAIDKKPGSNSPV

>dreCCL39aj blast search Chr 25

mripvfllflvftmcsiqlvpaMPATPIAINSAKSVCCEGITHKVIPLKQIMSYQWTTSTCPIKAIVFKTIAGREICVDPQNTLVKKHIANLDKRTSSTTALSPESTFTTAETPASTSATASSSESTSAKSSPASTSATALSHEYTSASTVTFTTAESHSTSV

>dreCCL44 ENSDART00000109972 Chr 11

mflqtvsilflsavlfgclegKGVQMQRDVQCCMQYSHGKVRTKDVLRYERQTEGPDCSIRAIILYTKKAVKCADPRDRKVKRLLRKLNQRLGAKARRTMWLHPHLNLPVMSEVAVVNSQKMNKTK

>tniCXCL8a ENSTNIT00000021199 Chr 18

mcsrifltslvvllaflaisngMSLRSLGVEQHCRCIETESRRIGRHIGKVELILPNSHCERMEIIATLKKTGEEVCLDPEAAWVKKVIERFLSRMALTICTRFTDEYQLFEELGK

>tniCXCL12a CAG09382.1 Chr 17

mdvklvtlvaalmvvlyappsqaKPISLVERCYCRSTVSNLPRAYIRELRFIHTPNCPFQVIAKLKSNKEVCVNPQIQWLQQYLKNAINKMKKFKQGN

>tniCXCL12b ENSTNIT00000002721 Chr 18

ielkiipliaiiaiaakspvwpaKPISLVERCWCRSTLNTVPQRSIKELKFLHTPNCPFQVIAKLKNNREVCINPETKWLQQYLKNAINKVKKNRRRNK

>tniCXCL13 ENSTNIT00000020058 Chr 12

maklltlllvlmlcchqapadaFSGCHCLRIFRRPIPFRIIKQVEMIPISGQCRRPETILTRRNGSKDCIDPNQQWFKDVLRKITVPNSRNVTKNATKPGNF

>tniCXCL14 CAF90863.1 Chr Un_random

mhrctalllllvvslyvlgaeaYKCRCTRKGPKIRYKDVQKLEIKPKHPYCQEKMIFVTMENVARFKGQEYCLHPKLQSTKNLVKWVYEA

>tniCXCL18a ENSTNIT00000000555 Chr 17

melhlpsarqlvllslgcvlitvrqsdgTFVPGRCLCPQTQATVRGPLRALSIYHRNPTCNKVTVIVTLRNNDEVCLDPKAPLGKRLIHCWRRTQKKGRDVRHCLRRRRRNVKQGGRPGQGSPQSSRGQGRKSSSS

>tniCXCL19 ENSTNIT00000005982 Chr Un_random

mkicilfvfatllaastgMAPISRDYVTRCQCLQVESRIIPPDSLRSIKLIPEGPHCPTAEVIAGLASGAKVCLNPKSTWVKKLVQFVLEKQLNRKQATLPKTKA

>truCXCL20 blast search Scaffold 421

mmrttvalciflaciavctsSPACRCLNTVAAVNPSHVVDVVEYGPRPYCRRQEVIVILKNKRPRCLDPKGQFAQGLLWAKR

>tniCCL19aa ENSTNIT00000018653 Chr 12

malfgdaklvlclffaiccymtvtqaEVPADCCLSVTNAEVIKHAIVDYRRQVAGQGCTLNATILVTRRQKQLCAPASERWVEDVVAHVKQLRKCCNKAKCRQANKKKRCLGVKAE

>tniCCL19ab CAG07793.1 Chr 12

masraaallllglvcvqfaaaQVVLDCCRTKTSKLLPLQLIRSYSVQDAGAGCDISATVFVTKTGRQLCVSHPSEEKWVQKHIDALLRRKEKHAKTRVE

>tniCCL19ac ENSTNIT00000018654 Chr 12

masraaallllglvcvqfaaaQVVLDCCRTKTSKLLPLQLIRSYSVQDAGAGCDISATVFVTKTGRQLCVSHPSEEKWVQKHIDALLRRKEKHAKTRVE

>tniCCL20aa blast search Chr 15

mitvttvvlcfflvltpahyAPGSHASSGCCTRYSRRPVPFQLIKGYREQTTMENCHIEAIIFYTVQRKMICASRKDEWVRKLLKLLRWRCRLVKNGPAGLKRHR

>tniCCL20ab blast search Chr 15

mmklmlglsvlvlllaltesRYFCCTQYHEKPVPVKMLKYYIIQEDTGYCNIKAVIFKTKTKPLCANPESWWVKIAMETVPL

>tniCCL25a CAG07167.1 Chr 15

mrlnalffltitacvclalaQITYEDCCLKYIKKVKPRIQKYAVSYRLQVLDGGCNLPAVIFVMKKGRVVCTDPKEQWVTELMRQIDGRRLRTHSNKSTKHNSRG

>tniCCL25b ENSTNIT00000018776 Chr 1

mklqtlfvlllftcmymslaQGSYGNCCLGYVPAMRRNAKNIERYWRQETDGDCNIRAVVFVMKKKQGQKKPRTVCANPEQTWVQSLMAHVDGREGKIN

>truCCL27 CAG09577.1 Chr 12

mdlkvvavliclsafaisstqaAIPGCCINTRKIIPINVLRKVSRWTIQSSGGACDIDAVILHVRDKRICVDQTVFKDIWWRMKQWKQRVKKRAAKYNV

>tniCCL33a CAF98923.1 Chr 13

mktlclavtlllltvcccnaMPSALPLPENVRCCAQFTEQPVPKRNVRKIYKTSHQCGQKAFIVETLRRELCYRQSFPWALKVYKEFSDTADIQ

>tniCCL36a (ENSTNIT00000021107) Chr 18

mrtapalllcllaagllsfascRNEIGPDDCCFRFYPHRVKRTLVRSYYATDQRCSKTAVILVTQRSRHICVDPNLSWVETLLKNLEESSF

>tniCCL40a CAF90799.1 Chr 5

mktqvtfallvlacvlhhhadaQVVHDVLLQSLCCKSVRRMCVQETMVTKVVKNRCGAQKAILVVRKNNQTLCLDSEWKWAQNLLEKFSSTDAETKNVTLDYSMCLYKKRQGARI

>truCCL44 blast search Scaffold 135

mvmlqmltvisltvillasvegKGVQMQKDVQCCMLYSQGKVRTKDVLRFEVQTEGPDCSIQAIILYTKKAVKCADPRDRKVKRLLRKLLQRQRTKAHRTMWLQPYDNLPVMTEVRENL

>cmiCXCL8a blast search AAVX01024088.1

KMKTYLRCRCIKTQSTFIHPKHITNVDLITNGPHCSVDEIIITLIKGNKICLDPNEKWVQMVINIIQRYCFTSSI

>cmiCXCL8b blast search AAVX01202460.1

ASIGSAGGSLRCQCVKTMSEFINPKFMKNIEIVPSGPHCSNAEIIVTLKSTNRVCLDPQAPWVKRIINRVMNG

>cmiCXCL8c blast search AAVX01003327.1

ASLRRTEVNLRCNCIRTNSNFIHPKFMDHIDIFPSGPHCPVVEIIATLKSGNRVCLYPEASWVKKIIEKMMTR

>cmiCXCL8d blast search AAVX01039483.1

ASLRGKGANLQCQCIKPSSDFIRPTRMKEIDIIPSGPHCGNVEIIMSPLQIQNKSVCTLKHPGYKESLTR

>cmiCXCL12a blast search AAVX01074010.1

KPSSISYRCKCRGGTTRLHPGMIRKLLFLPIPNCPPQI

>cmiCXCL12b blast search AAVX01466911.1

KPAAVLNRCICRGGTLHINVNNIKALQIIPIPNCPLQL

>cmiCXCL13 blast search AAVX01063766.1 AC238883.1

SLGPGIESKCKCIRTTSAFIHPRKYQHVDIFPQSTLCRRVEIIIRLINKRVVCINPETAWVKKVVSIITERYGSFDITFCIFLPVLSVSESI

>cmiCXCL14 blast search AAVX01357766.1

YKCKCIRKGPKIHYKKVKKVEIKPRYPYCQEKMIFVTMQKAARFKGHQYCLHPKLQSTKNLIQWYNKWKRDHR

>cmiCXCL22a blast search AAVX01259558.1 AC238883.1

IPIVGTSAHCLCIQTTSRFIKLQNIQSLEYIHRRSGCESTEIIVTLKSNRKVCVNPDAKWVKVVIARRGRFLIDSLLHCVLLFSCPPKTEHFIIPYSLNR

>cmiCXCL22b blast search AAVX01252418.1 AC238883.1

IPIVGTRARCLCTQTTSRFIKLQNIQSLKYIPRGTSCESTEIIVTLKSNRKVCVNPDAKWVKVVIARRGRFLIDSLLACDSNENTTSSL

>cmiCXCL22c blast search AAVX01252418.1 AC238883.1

IPIVGTRARCLCTQTTSRFIKLQNIQSLKYIPRGTSCESTEIIVTLKSNRKVCVNPDAKWVKVVIARRGRFLIDSLLACDSNENTTSSL

>cmiCXCL22d blast search AAVX01255080.1 AC238883.1

IPIVGTSAHCLCIQTTSRFIKLQNIQSLEYIPRRSGCESTEIIVTLKSNRKVCVNPDAKWVKVVIARRGRFLIDSLLACDSNENTTCSL

>cmiCXCL23 blast search AAVX01045995.1

QPLLHKGRRCKCINTINRLHPSMRINNVKILVQQDYCPNVEIIVNLQNGNKICLNPTSVIGKKIINLMEL

>cmiCCL19 blast search AAVX01270433.1

YTENGGIMDCCLSVSRKRIPGRIVANYLRQEPADGCRIRAV

>cmiCCL20 blast search AAVX01221985.1

AQIPVDCCLSYARIMIPVKSIIGYIRQHSNELCRIDAI

>cmiCCL25 blast search AAVX01160627.1

MSYEDCCLSYNVVKHPKKLSRKIIHHRVQGTGGGCNLPAIVLTLKKSRIVCVDPEEKWLQMFLKKSRKWQKETRTRSKKLSDSVGEWEQLNDLVSVTGLQLNMVMGG

>cmiCCL27 blast search AAVX01160772.1

PGQIKKSCCLTASAQPPHYKKLTNYEIQENDGRCNIKAV

>pmaCXCL8 blast search Scaffold GL479477 Pma.5155

mtmnakllfvllalalcvghsqaMSVFGGGRCQCVHVISKFIHPKHFQTMEVIPQSSNCKNVEIIVTMKSTKNQICLNPDAPWVRKVISHILDGAQTPKPTP

>pmaCXCL12 blast search Scaffold GL481915 EE279694

mrallfigflvcclavfdlsqgITIAMQSRCPCRHSRNVQPSTIKKLDIFKQVNCPIQV

>pmaCXCL15L blast search Scaffold GL476971 Pma.7216

mklavssllvclmlcmkysqaAPNPRCLCITSSSNFIPVKMLRNIEVIPKSSRCNKVEVIATLKTNIDQKICLSPAAPWVKALVSKLLNRSQTSPHRK

>pmaCXCL21 blast search Scaffold GL491430 EC382525.1

mgspripsppllllllllatllstshgVIVHGLDTRCQCADFYRKPLRLKSIRGLVDHRFTSSCHRDVIASLPNGRKVCLDPDKAWVKHILRRFKKEFGLNRSG

**(b) Chemokine domain sequences** (smart00199; http://0-www.ncbi.nlm.nih.gov.elis.tmu.edu.tw/Structure/cdd/

cddsrv.cgi?uid=197570) used for the construction of phylogenetic trees. Incomplete sequences were omitted from this list.

>hsaCXCL1

ELRCQCLQTLQGIHPKNIQSVNVKSPGPHCAQTEVIATLKNGRKACLNPASPIVKKIIEKM

>hsaCXCL2

ELRCQCLQTLQGIHLKNIQSVKVKSPGPHCAQTEVIATLKNGQKACLNPASPMVKKIIEKM

>hsaCXCL3

ELRCQCLQTLQGIHLKNIQSVNVRSPGPHCAQTEVIATLKNGKKACLNPASPMVQKIIEKI

>hsaCXCL4

DLQCLCVKTTSQVRPRHITSLEVIKAGPHCPTAQLIATLKNGRKICLDLQAPLYKKIIKKL

>hsaCXCL4L1

DLQCLCVKTTSQVRPRHITSLEVIKAGPHCPTAQLIATLKNGRKICLDLQALLYKKIIKEH

>hsaCXCL5

ELRCVCLQTTQGVHPKMISNLQVFAIGPQCSKVEVVASLKNGKEICLDPEAPFLKKVIQKI

>hsaCXCL6

ELRCTCLRVTLRVNPKTIGKLQVFPAGPQCSKVEVVASLKNGKQVCLDPEAPFLKKVIQKI

>hsaCXCL7

ELRCMCIKTTSGIHPKNIQSLEVIGKGTHCNQVEVIATLKDGRKICLDPDAPRIKKIVQKK

>hsaCXCL8

ELRCQCIKTYSKPFHPKFIKELRVIESGPHCANTEIIVKLSDGRELCLDPKENWVQRVVEKF

>hsaCXCL9

KGRCSCISTNQGTIHLQSLKDLKQFAPSPSCEKIEIIATLKNGVQTCLNPDSADVKELIKKW

>hsaCXCL10

TVRCTCISISNQPVNPRSLEKLEIIPASQFCPRVEIIATMKKKGEKRCLNPESKAIKNLLKAV

>hsaCXCL11

RGRCLCIGPGVKAVKVADIEKASIMYPSNNCDKIEVIITLKENKGQRCLNPKSKQARLIIKKV

>hsaCXCL12

SYRCPCRFFESHVARANVKHLKILNTPNCALQIVARLKNNNRQVCIDPKLKWIQEYLEKA

>hsaCXCL13

SLRCRCVQESSVFIPRRFIDRIQILPRGNGCPRKEIIVWKKNKSIVCVDPQAEWIQRMMEVL

>hsaCXCL14

GSKCKCSRKGPKIRYSDVKKLEMKPKYPHCEEKMVIITTKSVSRYRGQEHCLHPKLQSTKRFIKWY

>hsaCXCL16

TGSCYCGKRISSDSPPSVQFMNRLRKHLRAYHRCLYYTRFQLLSWSVCGGNKDPWVQELMSCL

>hsaCXCL17

GQECECKDWFLRAPRRKFMTVSGLPKKQCPCDHFKGNVKKTRHQRHHRKPNKHSRACQQF

>hsaCCL1

FSRCCFSFAEQEIPLRAILCYRNTSSICSNEGLIFKLKRGKEACALDTVGWVQRHRKML

>hsaCCL2

PVTCCYNFTNRKISVQRLASYRRITSSKCPKEAVIFKTIVAKEICADPKQKWVQDSMDHL

>hsaCCL3

PTACCFSYTSRQIPQNFIADYFETSSQCSKPGVIFLTKRSRQVCADPSEEWVQKYVSDL

>hsaCCL3L1

PTACCFSYTSRQIPQNFIADYFETSSQCSKPSVIFLTKRGRQVCADPSEEWVQKYVSDL

>hsaCCL3L3

PTACCFSYTSRQIPQNFIADYFETSSQCSKPSVIFLTKRGRQVCADPSEEWVQKYVSDL

>hsaCCL4

PTACCFSYTARKLPRNFVVDYYETSSLCSQPAVVFQTKRSKQVCADPSESWVQEYVYDL

>hsaCCL4L1

PTACCFSYTARKLPRNFVVDYYETSSLCSQPAVVFQTKRGKQVCADPSESWVQEYVYDL

>hsaCCL4L2

PTACCFSYTARKLPRNFVVDYYETSSLCSQPAVVFQTKRGKQVCADPSESWVQEYVYDL

>hsaCCL5

TTPCCFAYIARPLPRAHIKEYFYTSGKCSNPAVVFVTRKNRQVCANPEKKWVREYINSL

>hsaCCL7

STTCCYRFINKKIPKQRLESYRRTTSSHCPREAVIFKTKLDKEICADPTQKWVQDFMKHL

>hsaCCL8

PITCCFNVINRKIPIQRLESYTRITNIQCPKEAVIFKTKRGKEVCADPKERWVRDSMKHL

>hsaCCL11

PTTCCFNLANRKIPLQRLESYRRITSGKCPQKAVIFKTKLAKDICADPKKKWVQDSMKYL

>hsaCCL13

PSTCCFTFSSKKISLQRLKSYVITTSRCPQKAVIFRTKLGKEICADPKEKWVQNYMKHL

>hsaCCL14

PSECCFTYTTYKIPRQRIMDYYETNSQCSKPGIVFITKRGHSVCTNPSDKWVQDYIKDM

>hsaCCL15

AADCCTSYISQSIPCSLMKSYFETSSECSKPGVIFLTKKGRQVCAKPSGPGVQDCMKKL

>hsaCCL16

PSTCCLKYYEKVLPRRLVVGYRKALNCHLPAIIFVTKRNREVCTNPNDDWVQEYIKDP

>hsaCCL17

GRECCLEYFKGAIPLRKLKTWYQTSEDCSRDAIVFVTVQGRAICSDPNNKRVKNAVKYL

>hsaCCL18

KELCCLVYTSWQIPQKFIVDYSETSPQCPKPGVILLTKRGRQICADPNKKWVQKYISDL

>hsaCCL19

AEDCCLSVTQKPIPGYIVRNFHYLLIKDGCRVPAVVFTTLRGRQLCAPPDQPWVERIIQRL

>hsaCCL20

NFDCCLGYTDRILHPKFIVGFTRQLANEGCDINAIIFHTKKKLSVCANPKQTWVKYIVRLL

>hsaCCL21

AQDCCLKYSQRKIPAKVVRSYRKQEPSLGCSIPAILFLPRKRSQAELCADPKELWVQQLMQHL

>hsaCCL22

DSVCCRDYVRYRLPLRVVKHFYWTSDSCPRPGVVLLTFRDKEICADPRVPWVKMILNKL

>hsaCCL23

SADCCISYTPRSIPCSLLESYFETNSECSKPGVIFLTKKGRRFCANPSDKQVQVCVRML

>hsaCCL24

PSPCCMFFVSKRIPENRVVSYQLSSRSTCLKAGVIFTTKKGQQFCGDPKQEWVQRYMKNL

>hsaCCL25

FEDCCLAYHYPIGWAVLRRAWTYRIQEVSGSCNLPAAIFYLPKRHRKVCGNPKSREVQRAMKLL

>hsaCCL26

SKTCCFQYSHKPLPWTWVRSYEFTSNSCSQRAVIFTTKRGKKVCTHPRKKWVQKYISLL

>hsaCCL27

STACCTQLYRKPLSDKLLRKVIQVELQEADGDCHLQAFVLHLAQRSICIHPQNPSLSQWFEHQ

>hsaCCL28

ASSCCTEVSHHISRRLLERVNMCRIQRADGDCDLAAVILHVKRRRICVSPHNHTVKQWMKVQ

>hsaCX3CL1

CNITCSKMTSKIPVALLIHYQQNQASCGKRAIILETRQHRLFCADPKEQWVKDAMQHL

>hsaXCL1

DKRTCVSLTTQRLPVSRIKTYTITEGSLRAVIFITKRGLKVCADPQATWVRDVVRSM

>hsaXCL2

HRRTCVSLTTQRLPVSRIKTYTITEGSLRAVIFITKRGLKVCADPQATWVRDVVRSM

>musCXCL1

ELRCQCLQTMAGIHLKNIQSLKVLPSGPHCTQTEVIATLKNGREACLDPEAPLVQKIVQKM

>musCXCL2

ELRCQCLKTLPRVDFKNIQSLSVTPPGPHCAQTEVIATLKGGQKVCLDPEAPLVQKIIQKI

>musCXCL3

ELRCQCLNTLPRVDFETIQSLTVTPPGPHCTQTEVIATLKDGQEVCLNPQGPRLQIIIKKI

>musCXCL4L1

DLSCVCVKTISSGIHLKHITSLEVIKAGRHCAVPQLIATLKNGRKICLDRQAPLYKKVIKKI

>musCXCL6

ELRCVCLTVTPKINPKLIANLEVIPAGPQCPTVEVIAKLKNQKEVCLDPEAPVIKKIIQKI

>musCXCL7b

ELRCRCTNTISGIPFNSISLVNVYRPGVHCADVEVIATLKNGQKTCLDPNAPGVKRIVMKI

>musCXCL9

NARCSCISTSRGTIHYKSLKDLKQFAPSPNCNKTEIIATLKNGDQTCLDPDSANVKKLMKEW

>musCXCL10

TVRCNCIHIDDGPVRMRAIGKLEIIPASLSCPRVEIIATMKKNDEQRCLNPESKTIKNLMKAF

>musCXCL11

QGRCLCIGPGMKAVKMAEIEKASVIYPSNGCDKVEVIVTMKAHKRQRCLDPRSKQARLIMQAI

>musCXCL12

SYRCPCRFFESHIARANVKHLKILNTPNCALQIVARLKNNNRQVCIDPKLKWIQEYLEKA

>musCXCL13

NLKCRCSGVISTVVGLNIIDRIQVTPPGNGCPKTEVVIWTKMKKVICVNPRAKWLQRLLRHV

>musCXCL14

GSKCKCSRKGPKIRYSDVKKLEMKPKYPHCEEKMVIVTTKSMSRYRGQEHCLHPKLQSTKRFIKWY

>musCXCL15

ELRCLCIQEHSEFIPLKLIKNIMVIFETIYCNRKEVIAVPKNGSMICLDPDAPWVKATVGPI

>musCXCL16

AGSCSCDRTISSGTQIPQGTLDHIRKYLKAFHRCPFFIRFQLQSKSVCGGSQDQWVRELVDCF

>musCXCL17

GQECECKDWFLQAPKRKATAVLGPPRKQCPCDHVKGREKKNRHQKHHRKSQRPSRACQQF

>musCCL1

SNSCCLNTLKKELPLKFIQCYRKMGSSCPDPPAVVFRLNKGRESCASTNKTWVQNHLKKV

>musCCL2

PLTCCYSFTSKMIPMSRLESYKRITSSRCPKEAVVFVTKLKREVCADPKKEWVQTYIKNL

>musCCL3

PTACCFSYSRKIPRQFIVDYFETSSLCSQPGVIFLTKRNRQICADSKETWVQEYITDL

>musCCL4

PTSCCFSYTSRQLHRSFVMDYYETSSLCSKPAVVFLTKRGRQICANPSEPWVTEYMSDL

>musCCL5

TTPCCFAYLSLALPRAHVKEYFYTSSKCSNLAVVFVTRRNRQVCANPEKKWVQEYINYL

>musCCL6

SSDCCFSYATQIPCKRFIYYFPTSGGCIKPGIIFISRRGTQVCADPSDRRVQRCLSTL

>musCCL7

ASTCCYVKKQKIPKRNLKSYRRITSSRCPWEAVIFKTKKGMEVCAEAHQKWVEEAIAYL

>musCCL8

PVTCCFHVLKLKIPLRVLKSYERINNIQCPMEAVVFQTKQGMSLCVDPTQKWVSEYMEIL

>musCCL9

SSDCCLSYNSRIQCSRFIGYFPTSGGCTRPGIIFISKRGFQVCANPSDRRVQRCIERL

>musCCL11

PTSCCFIMTSKKIPNTLLKSYKRITNNRCTLKAIVFKTRLGKEICADPKKKWVQDATKHL

>musCCL12

PVTCCYNVVKQKIHVRKLKSYRRITSSQCPREAVIFRTILDKEICADPKEKWVKNSINHL

>musCCL17

GRECCLDYFKGAIPIRKLVSWYKTSVECSRDAIVFLTVQGKLICADPKDKHVKKAIRLV

>musCCL19a

AEDCCLSVTQRPIPGNIVKAFRYLLNEDGCRVPAVVFTTLRGYQLCAPPDQPWVDRIIRRL

>musCCL19b

AEDCCLSVTQRPIPGNIVKAFRYLLNEDGCRVPAVVFTTLRGYQLCAPPDQPWVDRIIRRL

>musCCL20

NYDCCLSYIQTPLPSRAIVGFTRQMADEACDINAIIFHTKKRKSVCADPKQNWVKRAVNLL

>musCCL21a

GQDCCLKYSQKKIPYSIVRGYRKQEPSLGCPIPAILFSPRKHSKPELCANPEEGWVQNLMRRL

>musCCL21b

GQDCCLKYSQKKIPYSIVRGYRKQEPSLGCPIPAILFLPRKHSKPELCANPEEGWVQNLMRRL

>musCCL21c

GQDCCLKYSQKKIPYSIVRGYRKQEPSLGCPIPAILFLPRKHSKPELCANPEEGWVQNLMRRL

>musCCL21d

GQDCCLKYSQKKIPYSIVRGYRKQEPSLGCPIPAILFLPRKHSKPELCANPEEGWVQNLMRRL

>musCCL21e

GQDCCLKYSQKKIPYSIVRGYRKQEPSLGCPIPAILFLPRKHSKPELCANPEEGWVQNLMRRL

>musCCL22

DSICCQDYIRHPLPSRLVKEFFWTSKSCRKPGVVLITVKNRDICADPRQVWVKKLLHKL

>musCCL24

PSSCCTSFISKKIPENRVVSYQLANGSICPKAGVIFITKKGHKICTDPKLLWVQRHIQKL

>musCCL25

FEDCCLGYQHRIKWNVLRHARNYHQQEVSGSCNLRAVRFYFRQKVVCGNPEDMNVKRAMRIL

>musCCL26

AMSCCPNFSYYVIPWSWVYSYKFTDKSCTSDGVIFFTKTGKQFCVQPGAKWVQRFISLV

>musCCL27a

STSCCTQLYRQPLPSRLLRRIVHMELQEADGDCHLQAVVLHLARRSVCVHPQNRSLARWLERQ

>musCCL27b

STSCCTQLYRQPLPSRLLRRIVHMELQEADGDCHLQAVVLHLARRSVCVHPQNRSLARWLERQ

>musCCL27c

STSCCTQLYRQPLPSRLLRRIVHMELQEADGDCHLQAVVLHLARRSVCVHPQNRSLARWLERQ

>musCCL27d

STSCCTQLYRQPLPSRLLRRIVHMELQEADGDCHLQAVVLHLARRSVCVHPQNRSLARWLERQ

>musCCL28

ASSCCTEVSHHVSGRLLERVSSCSIQRADGDCDLAAVILHVKRRRICISPHNRTLKQWMRAS

>musCX3CL1

CEIMCDKMTSRIPVALLIRYQLNQESCGKRAIVLETTQHRRFCADPKEKWVQDAMKHL

>musXCL1

EESSCVNLQTQRLPVQKIKTYIIWEGAMRAVIFVTKRGLKICADPEAKWVKAAIKTV

>btaCXCL1

ELRCQCLQTLQGIHLKNIQSVKVTTPGPHCDQTEVIATLKTGQEVCLNPAAPMVKKIIDKM

>btaCXCL2

ELRCQCLQTLQGIHLKNIQSVKVTTPGPHCDQTEVIASLKTGQEVCLNPTAPMVKKIIDKM

>btaCXCL3

ELRCHCLQTLQGIHLKNIQSVKVTPPGPHCGQTEVIATLKNGQEACLNPEAPMVKKIINKM

>btaCXCL4L1

DLQCVCLKTTSGINPRHISSLEVIGAGLHCPSPQLIATLKTGRKICLDQQNPLYKKIIKRL

>btaCXCL6

ELRCVCLTTTPGIHPKTVSDLQVIAAGPQCSKVEVIATLKNGREVCLDPEAPLIKKIVQKI

>btaCXCL7b

ELRCLCVKTISGIPSSNIQSLEVNRAGPHCNKVEVIAELKNGKKICLNPEGPRIKKIVQKI

>btaCXCL8

ELRCQCIKTHSTPFHPKFIKELRVIESGPHCENSEIIVKLTNGNEVCLNPKEKWVQKVVQVF

>btaCXCL9

NGRCSCINTSQGMIHPKSLKDLKQFAPSPSCEKTEIIATMKNGNEACLNPDLPEVKELIKEW

>btaCXCL10

NTRCSCIEISNGSVNPRSLEKLEVIPASQSCPRVEIIATMKKNGEKRCLNPESKTIKNLLKAI

>btaCXCL11

GGRCLCIGPGVKAVKVADIEKVSIIYPTNNCDKTEVIITLKTHKGQRCLNPKAKQAKAIIKKV

>btaCXCL12

SYRCPCRFFESHVAKANVKHLKILNTPNCSLQIVARLKNNNRQVCIDPKLKWIQEYLDKA

>btaCXCL13

NLKCKCIRKTVSFFPVNLIERLNIIPRGRGCPNTEIIVWMKNKLVICLNPQAKWTQTLIKVL

>btaCXCL14

GSKCKCSRKGPKIRYSDVKKLEMKPKYPHCEEKMVIITTKSMSRYRGQEHCLHPKLQSTKRFIKWY

>btaCXCL15

ELRCQCIQTQSDFISPKFIAKVQIIPEGAHCNRKEIIVTLKDGQLICLDPEAEWVMNIIKKI

>btaCXCL16

VGSCPCDHTVSSHSPPNENIMRHLRKYLKAYQRCFSYVRFQLPLKNVCGGSTDGWVQELMHCF

>btaCXCL17

GQECECQDWFLRAPRRTLMAAPRLTKPCPCDHFKGRMKKTRHQRHHRKSNKPSRACQQF

>btaCCL1a

SSNCCFRTVKGKISPKKIQCYKNISSTCSYNDRLIFKLTGGLQSCVLQKDLWVQAYLKRI

>btaCCL1b

SSNCCFRTVKGKISPKKIQCYKNISSTCSYNDRLIFKLTGGLQSCVLQKDLWVQAYLKRI

>btaCCL2

QVACCYTFNSKKISMQRLMNYRRVTSSKCPKEAVIFKTILGKELCADPKQKWVQDSINYL

>btaCCL3a

PTACCFSFVSRQIPRKFVDDYYETSSQCSKPGIIFKTKRGRQVCADPSEDWVQEYITDL

>btaCCL3b

PTACCFSYVARQLSRKIVADYFETSSQCSKPGVIFQTKKGRQVCANPTEDWVQEYITDL

>btaCCL3c

PTDCCFSFVSRQIPRKLVDDYYETSSQCSKPGIIFKTKRGRQVCADPSEDWVQEYIIDL

>btaCCL4

PTACCFSYTLRKIPRNFVNDYFETSSLCSQPAVVFQTKKGRQVCANPSEPWVQEYVDDL

>btaCCL5

TTPCCFAYISRPLPRTHVQEYFYTSSKCSMAAVVFITRKKRQVCANPEKKWVREYINAL

>btaCCL8a

PITCCFSVINGKIPFKKLDSYTRITNSQCPQEAVIFKTKADRDVCADPKQKWVQTSIRLL

>btaCCL8b

PITCCFSVINGKIPFKKLDSYTRITNSQCPQEAVIFKTKADRDVCADPKQKWVQTSIRLL

>btaCCL11

PTICCFNMSKKKISIQRLQSYRKITSSKCPQKAVIFNTKQNKKICVDPQEKWVQNAMEYL

>btaCCL14a

PAECCLTYVSRPVPRQRVSSYYETSSQCPKPGIIFITKKGHYICANPRDGWVQDYIKEL

>btaCCL14b

PPTCCFTYTSGKIPRGNVVNYFKTSSNCPKSGIIFLTRRGLSVCVNPADSWVQEYIRDL

>btaCCL16

PPNCCLKYHEKVLPRKLVVGYRQALNCHLPAIVFITKRKREVCTNPNNDWVQEYIKDP

>btaCCL17

GRECCLQFYKGSIPQKVLVGWYQTSDDCPNKAIVLVTRSGRTICANPKDKTVKKAMKYL

>btaCCL19

AEDCCLSVTQRPIPVFLVRAYRYLLLKDGCRLPAVVFTTQRGHELCAPPDQPWVGRIIRRL

>btaCCL20

NFDCCLRYTERILHPSILVGFTQQLANEACDINAVVFYTRKKLAVCADPKKKWVKQVVHML

>btaCCL21

AQDCCLRYSRKKIPANIVRSYRKQDPTLSCAMPAILFSPRKRSQPELCADPKEAWVQKLMQHL

>btaCCL22

DSVCCRDYIRYPLPLRLVKYYYWTSNSCRRPGVVLLTVKDREICADPKQHWVKKILQKL

>btaCCL23

PTDCCMSYTPRNIRCVFMENYIETSSACSRPAVIFITKKGQSVCADPNNEGVQKCKSEL

>btaCCL24

PSSCCITFISKKIPESRVISYQLTNRSICPQAGVIFTTQKGQKFCGNPKLPWVQKYVKNL

>btaCCL25

FEDCCLAYHRRARLSLLRHAQSYHRQDVSGSCNLPAVIFFLPQKNKMVCGRPGDRWVRAWMKIL

>btaCCL26

AKFCCFQYSQKTLPWKQVHSYEFTRNICSLKAVIFTTKRGRKVCAQPKEEWVQRHISKL

>btaCCL27

STACCTQLYRQPLPNKLLRRVIRVELQEADGDCHLQAFVLHLSQRRVCIHPQNRSLIRWFERQ

>btaCCL28

ASSCCTEVSHHISRRLLERVTTCRIQRADGDCDLAAVILHVKRRRVCVSPHNHVIKQWMNEQ

>btaCCL29

MRVCCFSSVTRKIPLSLVKNYERTGDKCPQEAVIFQTRSGRSICANPGQAWVQKYIEYL

>btaCX3CL1

CNLFCNKMTQKIPESRLVGYQRNRESCNDGAVILKTVKGKSFCADPKEEWVQKAMKHL

>btaXCL1

EKSICVSLTTQRLPIKNIKTYTIKEGSVKAVIFITRRGFKICADPQAAWVKKAVQKI

>btaXCL2

EKSICVSLTTQRLPIKNIKTYTIKEGSVKAVIFITRRGFKICADPQAAWVKKAVQKI

>lafCXCL1

ELRCQCVKTVQGIHPKNIASVKVTPPGPHCADTEVIATLKNGQEVCLNPTAPMVIRLIEKI

>lafCXCL4L1

DLRCLCVSTTSTVHPKHVISLEVIKAGLHCPKAQLIATLKNGRKICLDQQARLYKKIIKKL

>lafCXCL6

ELRCMCLVITPGIHPKMISNLQVIAAGPQCPKVEAIGYLKSGKEVCLDPEAPLIKKIIQRI

>lafCXCL7b

ELRCMCVKTTSGVRPSYINNLQVIRAGAHCDKVEVIVTLKDGKKICLDPEAPVIKKIVQKI

>lafCXCL8

ELRCQCIKTHSTPFHPKYIKELRVIESGPHCPNSEIIVKIVTKEGEEGKEVCLDPTAKWVQKVVQVF

>lafCXCL9

NGRCFCININQEKIQLQSLKDLQQFAPSSSCGKAEIIATLKNGDQTCLNPDLKDVKKLVKAW

>lafCXCL10

TTRCTCIKISNQPVNPKFLEKLEMIPASLSCPHVEIIVTMKKSGEKRCLNPESKNVKNILKAI

>lafCXCL11

AGRCLCIGPGVKAVRVADIEKASIIYPSYNCDKLEVIIILKANKGQRCLNPRSKQANLIIKVE

>lafCXCL12

SYRCPCRFFESHVARANVKHLKILSTPNCALQIVARLKNNNRQVCIDPRLKWIQEYLDKT

>lafCXCL13

NLKCKCLQQTSVYIPAQNIQRLQIFPAGSGCPNVEVIVWMKNNRVVCSNPYTKWFQKLLKVL

>lafCXCL14

GSKCKCSRKGPKIRYSDVKKLEMKPKYPHCEEKMVIITTKSVSRYRGQEHCLHPKLQSTKRFIKWY

>lafCXCL16

TGSCYCDRLISTPTMEQKEHFRKHLKGYTCCRLFVRFELHSRTVCGGSTKPWVLHLMSCF

>lafCXCL17

SQECECKDWLLGAPKRKSMTVTVPGLPKKPCPCDHFKGNVKKIRHQRHHKKPNKHSRACQQF

>lafCCL1

SSNCCYKFMNKSISLKNIQCYKNTSSTCPYDALILKLKKGRESCVLKTVKWVQDNFSKM

>lafCCL2

PVTCCYTFVDKKIVVQRLASYRKVTNARCPKEAVIFKTKLAKELCADPKQKWVRDSTAYL

>lafCCL3

PTACCFSYVSRQMKFKSITDYYETNSQCSKPGIIFITKKNQQVCAKPSEAWVQEYITNL

>lafCCL4a

PTACCFSYTVRQLPRNFVIDYYETSSLCSNPAVVFQTKRGRQVCANPSESWVQEYMDDL

>lafCCL4b

PTACCFSYALRKLPRNFVIDYYETSSLCAKPAVVFQTKRGREVCANPSEPWVQEYMDDL

>lafCCL4c

PTACCFSYTSRQIQRKFVDSYYETSSQCSKPAVVFLTKRGRQVCANPSDAWVQEYVTDL

>lafCCL5

TTPCCFAYTPRTLPHAHVKEYFYTSGKCSMPAVVFVTRRNRQVCADPEKKWVKEYINTL

>lafCCL8

PITCCFNVVSRKIPIQNLVSYTRITNSECPRKAVIFKTKLAKEVCADSEERWVQNSMKRL

>lafCCL11a

SNMCCFKVASKKIPIQRLESYTQVASSKCPLTAVVFKTKLAKKICTDPKKKWVRDSMKYL

>lafCCL11b

PPVCCFKVVSKKILIQKLVSYTRITSSQCPREDVIFKAKLDKEICAEPRKKWVQDAMKHL

>lafCCL14

PTECCFTYITRRVPRTLVSGYYETNSQCTKPGVVFITKKGYALCANPSDDWVQDYIKGL

>lafCCL16

QVTCCLKYHEKVLPRRLVMGYRKALSCHLPAIMNREVCANPNDDWVQEYIKNP

>lafCCL17

GRECCLEYFKGAIPLKKLTSWYRTSEECPRDAIVFLTVQGRAICSDPKDTRVRKAVGHL

>lafCCL19

AEDCCLSVTQRPIPGNIVRAFHYLLLKDGCRVPAVVFTTMRGHQLCAPPDQPWVGRIIRRL

>lafCCL20

SFDCCLRYTEHVIHPKLITGFTQQLSSEACDIDAVIFHTKRRLAVCADPKKVWVKQAVWIL

>lafCCL21

AQDCCLKYSRRTIPARVVQSYRKQEPSLGCPIPAILFKPRKQSQPELCADPKEDWVQQLMQQL

>lafCCL22

DSVCCREYIRYPLPLRLVKYFFWTSDSCRRPGVVLLTIKDREICADPRLPWVKKVLQKL

>lafCCL23

PSDCCITYTARNIRCAFVDHYFETSSGCSQPGVIFITKKGQRVCANPLIKHVQDCVNNL

>lafCCL24

PSSCCMSFISKKIPEGRVVGYQVSNGSVCPKAGVIFTTKKGQKFCGDPKQLWVQRYMKNL

>lafCCL25

FEDCCLAYHRHAGRAIVQRAQGYLRQEVSGSCNLPAVIFFFPRRNRKVCGNPQDRWVQNGMKLL

>lafCCL27

SITCCTQLYQQPLPNKLLRKVIRVDLQKADGDCHLQAFVLHLAQHSVCIHPQNHSLAQWFKRQ

>lafCCL28

ASSCCTEVSHHISRRLLERVTTCRIQRADGDCDLAAVILHVRRRRICASPHSHTVKQWMKAQ

>lafCX3CL1

CEITCNKMTRPIPVKLLLRYEGNPESCGKPAIILVTKKHRLFCANPDEKWVQEAMEHL

>lafXCL1

DRSVCVSLHTRPLPVQKIKSYTIKEGSMKAVIFITKRGLKICADPQAKWVKSAVRSM

>mdoCXCL1a

ELRCKCVRTTQGIHPKHIARVEVIVAGPHCHSNEVIATLKTGNVICLNPKAPWVKKFIQKT

>mdoCXCL1c

ELRCQCLQTVQGVPFKAIGSLKVIPAGPHCSNVEVIATLKNGNQRCLNPEAPQVKKLVEKA

>mdoCXCL8

ELRCKCIKTNSKPFHPKHIKELRVIESGPHCLNTEIIVTLQDDRELCLDPHANWVQRVLQAF

>mdoCXCL9

SRRCSCIDVSADIHRKNILHLEQFPPGSSCSNTEIIATLENGIKKCLDPDSPLVKKAVKAW

>mdoCXCL10

NIRCRCIKSYDAVPNVKVLQKLEVIPQSSSCSHTEIIATIKRTQEQRCLNPDSKQIQNLIKLI

>mdoCXCL11

GGWCLCRSSTVNSVYIANIKKVSIFFPSGSCNKIEIIITLKEGMGQICLNPESKQAKLILKRA

>mdoCXCL12

SYRCPCRFTESNVAKANIKHLKILNTPNCALQIVARLKNSSRQVCIDPKLKWIQEYLEKA

>mdoCXCL14

gSKCKCSRKGPKIRYSDVKKLEMKPKYPHCEEKMVIITTKSVSRYRGQEHCLHPKLQSTKRFIKWY

>mdoCCL1

ETTCCYEFQLRKCAYSHIKSYELTSSSCSYKAVIFTLKKGQKICVHLEEKWAQNYLNKL

>mdoCCL2a

PTTCCFEFTSKKIPSKFVVSYGITGSRCSKEGVIFVTRRNFEICADPNEQWVQNIINDL

>mdoCCL2b

PAICCFEFTTKKIPPKLVVNYEATSSTCANNGVIFFTKRGFEICANPEEKWVQNIVKLL

>mdoCCL2c

PKTCCFEFTNKKIPLKLLAYYQRTSNMCSKEAVILVTKSGYNLCANPKVHWVNEVMKHL

>mdoCCL3a

PTSCCHSYTHHRLPYHLIVDFYETSSLCLKPAIVFLTSKGRQICANPKYEWVQRYILLL

>mdoCCL3b

PTACCFSYTSWKIPQTRVVDYYETSSKCSKPAIIFITKKGLQACANPRDPWVQELIKSV

>mdoCCL4a

PTSCCFSYVSQQIPRIFVTDYFETSSLCSRPAVVFQTKKGRQVCANPSDPWVQTYVEDL

>mdoCCL4b

PTSCCFSYVSQQIHRKFVTDYYETSSLCSQPAVVFQTKRGRQLCANPSDAWVQTYMEEL

>mdoCCL4c

PTSCCFSYVSQQIHRKFVIDYYETSSLCSQPAVVFQTKRGREVCANPSDAWVQTYMEEL

>mdoCCL4d

PTSCCFSYVSQQIHRKFVIDYYETSSLCSQPAVVFQTKRGRQVCANPSDDWVQTYMEEL

>mdoCCL4e

PMSCCFSYVSKQIPRKFVTDYYETSILCSQPAVVFQTKKGRQVCANPSDDWVQKYKDDP

>mdoCCL4f

PTTCCFSYIPQPIPRTFVTGYYRTDSMCPKPAVVFKTIKNQQQCANPNEDWVKTYIEDL

>mdoCCL5

TTPCCFTFSTRPPPLVHIREYFYTSSRCGNLGVVFITRKNRQLCANPEKKWVRSYINSL

>mdoCCL17a

GHDCCITYTKAAIPFSKLVAWFKTPTDCRKEAIVFVTVLKKSICANPNEKWVKKAISFL

>mdoCCL17b

GHDCCITYTKAAIPFSKLVAWFKTPTDCRKEAIVFVTVLKKSICANPNEKWVKKAISFL

>mdoCCL19

AEDCCLSVTKSVIPRHIVCAYRRLTPENGCRLPAVVFTTLKGHQLCAPPDQHWVKRLIKRL

>mdoCCL20

SFDCCLQYIEHPINSKFIKGYAEQRSYEACDIDAIIFYTRKHVVCANPKETWVKNVLRIL

>mdoCCL21

ALDCCLKYSQKKIPASIVRTYRRQELNQGCSIPAIIFSPWKKSQADLCADPTVQWVQDLVKRL

>mdoCCL22

NSICCKDFVRFPVPLKFLTNFHFTSKTCRRQGVILTTVKGREICADPQKLWVKNVLKHL

>mdoCCL24

TSSCCTNYMRKTIPRNLVISYRVTNRSACSIPGVIFITKVKREVCADPTKQWVKDYMKMI

>mdoCCL26

ARYCCETYSPRPIAWKLVQSYELTKSSCSLSAVIFTTKKGQKVCADPKAKWTQRYVASL

>mdoCCL28

ASSCCTEVSHHISRTLLPRVKRCHIQRADGVCDLPAVILHVHHRKFCVSPHNHTLKKWMKMQ

>mdoCCL37a

NTVCCFSYIGRIPRTVLINYKYTSQSCPTPGVIFFTRRGHHQICANPEEQWVQNMVSSL

>mdoCCL37b

NHTCCFSYTKLKIPKRHVVDYTYTSEMCSQPGVIFITRKGLQICADPSSRWVQTYVASL

>mdoCCL43

PLACCKDFVRKRIPQSFMLGYTRSNPRCSKPAIMFETNKGVKVCANPHTPWVRNYVRNL

>mdoCX3CL1

CTHECPPLNRDMPQIPNHRLKNYTATSPSCRPKAIIFFTARNKIICADPDVKWVQEAVRFL

>mdoXCL1

KRRFCVSLTSKRIPVNSVKSYIIEEGSMRAVIFVTRKGIKICADPEVSWAKGVIRAV

>meuCXCL1a

ELRCRCVKTMQGIHPKNIVSLEVIRAGPHCPNHEVIATLKKGSEICLKAEAPWVKKFIQRY

>meuCXCL1c

ELRCQCLQTVQGIPYKNIANLKVIPAGPHCSNLEVIVTLKNGNERCLNPAAPQVKKIVEKA

>meuCXCL14

gSKCKCSRKGPKIRYSDVKKLEMKPKYPHCEEKVVIITTKTMSRYRGQEHCLHPKLQSTKRFIKWY

>meuCCL1

KPTCCFAFLKRSLPHNLIKSYAITSRSCSYKAVMFTLKKDKKYCAHLEDKWVQDYLKKN

>meuCCL2a

PTTCCFEYTAKKIPPKYVVSYEVTSSRCAKEGVIFVTKRGFQICTNPKEQWVQDIRNHL

>meuCCL2b

TPTCCFDFTSKRIPSKLVVSYGTTSSRCAKEGVIFITRQGHNICANPKEQWVQHVKKQL

>meuCCL2c

PENCCFEFTNKKIPLKLLVSYKNTSSMCPKEAVIFVTKRGFNICANLKDLWVQDLMKNL

>meuCCL3a

PNACCHSYTRRRIQYSLLVDFYETSSLCLKPGIIFLTNKGHQICANPKSEWVQEYIFQA

>meuCCL3b

PTACCFSYTSRKIPRAVVVDFYETSGHCSQPAIIFLTKKGYQACANPSEPWVQKYTKEL

>meuCCL4a

PTSCCFSYVSQQIPRKFVTDYFETSSLCSQPAVVFQTKRGRQVCANPSDAWVQSYVEDL

>meuCCL4b

PTSCCFSYASQQIPRKFVTDYFETSSLCSQPAVVFQTKRGRQVCANPSDAWVQSYVEDL

>meuCCL4c

PTSCCFSYVSQQIPRKFVTDYFETSSLCSQPAVVFQTKRGRQVCANPSDAWVQSYVEDP

>meuCCL4d

PTSCCFSYVRQQIPRKFVIEYYETSSLCSQPGVVFMTKKGRKMCANPSDNWVQKYMADP

>meuCCL4e

PTSCCFFYARQQIPRIFVTGYYRTSGLCSKPGVVFNTTIGKESCANPSDDWVKEYMADP

>meuCCL4f

PTSCCFSYVSQQIPRKFVTDYFETSSLCSQPAVVQKEVGRCVPTPSDIWVQSYVED

>meuCCL4g

PTSCCFSYVSQQIPRKFVTDYFETSSLCSQPAVVFQTKRGREVCANPSDAWVQ

>meuCCL5

TTPCCFSFTSLPPPLTHVREYFYTSSRCGNQGVVFITRKNRQLCASPEKKWVRSYINSL

>meuCCL19

AEDCCLSVTNLIIPRHIVCAYRRLNPENGCGLPAVVFTTQKGHQLCAPPDRFWVKRLIKRL

>meuCCL20

SFDCCLRYTERPVNTKLIKGYAEQWSYEACDIDAIIFYTRRYAVCADPKEAWVKHALKIL

>meuCCL22

SSVCCRNFVQFPLPLKVLTTFYYTSKNCRKRGVILKTVKKLEICADPQKVWVQRALATL

>meuCCL26

AKYCCEKYSSRPIPRKLVQTFELTRSNCGLSAVIFITKKGQKVCANPKAEWTQKYVASL

>meuCCL28

ASNCCTEVSHQVSRRLLAKVKRCHFQRADGVCDLPAVILHVHHRKFCVSPHNHTLKTWMETQ

>meuCCL37

PASCCFSYVTRLIPRKFVVDYDYTSQECFNPAVIFTTSRGFKICADPQKQWVKQYVANL

>meuCCL43

STPCCNNFVNHRIPQSLVIGFVRTSLRCPKPGVLFETKQGLKVCANPAVHWVQRYMKNL

>meuCX3CL1

CTHECPPLSRNLPQIPRQFLKRYDLTSPSCRIQAILFTTIKNRIICADPNEKWVQDAIQFL

>oanCXCL1a

ELRCQCVKTTQGIHHSNMAKVEVIPAGPSCANVEVIVTLKNGKNVCLNPDAPRVKKLIEKM

>oanCXCL1b

ELRCKCVRVTQGIHHSKIQNVEVIPAGPHCSEVEVIATLKNSQIVCLNPQAPLVKKLIKKL

>oanCXCL8

QLRCRCISTHPKRIARKHIKSVEVIFKGPHCSLDEVIVTLQDNKEVCLDTTKDWVQELIEKY

>oanCXCL10

PGRCRCLENSSKFIHPNALAKLEFLPKSSSCSNNEVIATLKKSKEEICLNPDSRNVKKLLDTI

>oanCXCL11

GSRCLCIGPRVNSVTPMQIKSISVFLPTSTCDRKEVIVTLKKGKGQRCLNLDSKQAQLILKVG

>oanCXCL12

SYRCPCRFSESNVAKANIKQLKILNTPNCALQIVARLKNSRQVCIDPKLKWIQEYLEKA

>oanCXCL14

gSKCKCSRKGPKIRYSDVKKLEVKPKYPHCEEKMVIITTKSVSRYRGQEHCLHPKLQSTKKFIKWY

>oanCXCL15a

QLRCQCISTHSDPILRHLIVNVRHIPKGAHCSTTEIIAELINGQLVCLNPEAKWVKILIERI

>oanCXCL15b

QLRCQCIRTHSDFIHPKFFANIQYIPAGPLCDTPEVIAEMKQGNEICLDPNANWVKIIIQKI

>oanCCL2a

PQSCCYSWSKKPIPLHLLSGYFVTSSKCSLEAVIFKTVKGVEVCADPKEKWVQDRMRRL

>oanCCL2b

PQSCCFSWSKKPIPLHLLSGYFVTSSKCSLESVIFRTVKGVEICTDPKEKWVQDRIKRL

>oanCCL3a

PTICCFSYTSRPLPIRLLSYYEETSSRCSKPAIIFTTKKGREVCADPSEEWVQDRIQDL

>oanCCL3b

PVTCCTNYLHRPLPQKLVKSYFQNRSQCPKPGIIFITVKGRKVCADPSEAWVQKIQARI

>oanCCL17

GKECCSDYIKKPIKFCKLVSYQKTSVDCLKDAIVLNTIQNNTICANPNQPWVKSAVKYL

>oanCCL20a

DFDCCLRYTERHYGSRIIKGYTEQFSNEVCDIDAIIFHTKRGALVCANPQEQWVKHVLHDL

>oanCCL20b

DLDCCLSYIQKPLPYQLIKDFIEQLPSETCDISAVILITRKKRLLCANPKDKWVKELILRY

>oanCCL21

APDCCLAYTQQIVRLNIVRSYRQQDTNGGCTIPAVIFSPKNPKRKDICADPSMPWVRELLRKL

>oanCCL22

DSICCTEFVKWPVRFRYLTEFYFTSLSCRRRGVVLKTVKNLEICADPQIPWVKKAIDLL

>oanCCL24a

EWCCFTFTRKMLSWSLIMSYFRANKDVCPEPAILFVTKKGLRICANPKEDWVQRYVKAL

>oanCCL27

AASCCTELSRHLLKPQLLKKVTGVKLQETGGGCHLRAIVLHLGRRFLCIHPKNRSLTRWFEQL

>ggaCXCL8a

ELRCQCIETHSKFIHPKFIQNVNLTPSGPHCKNVEVIATLKDGREVCLDPTAPWVKLIIKAI

>ggaCXCL8b

ELRCQCISTHSKFIHPKSIQDVKLTPSGPHCKNVEIIATLKDGREVCLDPTAPWVQLIVKAL

>ggaCXCL8c

ELRCRCVRAVAERIPPRHLVQVELVPEGPHCAAPEVIATTKQGHTLCLSPSVPWVKLLVARF

>ggaCXCL12

TYRCPCRFFESNVARANIKHLKILSTPNCSLQIVARLKSNSKQVCIDPKLKWIQEYLEKA

>ggaCXCL13a

NLNCRCVKTTSDYISPKRYDSIELRPVGSTCRRIEIIIKLKSSAKVCVNPDAPWVKKLLKRI

>ggaCXCL13b

NLNCRCAKTTTAFIPLRKYESVEVRPVGSSCRRLEVLIKLKTLERICVDPNTPWVRKLLQDL

>ggaCXCL13c

NKRCKCVKVTAQIISLGLILAIDVMPPGIHCRRKEIILTLKRNKKVCVAPEAPWIQLLIHKL

>ggaCXCL14

GVKCKCSRKGPKIRFSNVRKLEIKPRYPFCVEEMIIVTLWTKVRGEQQHCLNPKRQNTVRLLKWY

>ggaCCL1

YSSCCYKNMFIQKEINTSLIRRYRETPPNCSRRAIIVELKKGKKFCVDPAEGWFQQYLQGK

>ggaCCL4a

PTSCCFTYISRQLPFSFVADYYETNSQCPHAGVVFITRKGREVCANPENDWVQDYMNKM

>ggaCCL4b

NGRCCYKFLNRALPSSKVMMYEYTGSRCPYHGVIFTTFEGKKCCANPEEKWVQDILNVE

>ggaCCL5

TTVCCFNYSVRKLPQNHVKDYFYTSSKCPQAAVVFITRKGRQVCANPDARWVKEYINFL

>ggaCCL17

PSECCYEHTKFALRLEALKSFYETSHDCLLQAIVFVTKNGTKVCSKPNAPWVKKAVKYL

>ggaCCL19

VLDCCLRTSEKPIPWRIVQDYRMQLVQDGCDIPATVFITAKGKRLCAPPQAPWVLRLREKL

>ggaCCL20

NQDCCLSYSKVRLPRKVIKGFTEQLSGEVCDIDAIIFHTVRGLKACVNPKEDWVKKHLLFL

>ggaCCL21

ASDCCLKTSQKAISMKWVKSYSIQGPESGCVLRAVVFTTKKNKKICSSPTDPIVQKLIKSL

>ggaCCL28

AFNCCTKISEEIPKGILRRVERFEIQKYDGLCHLHAVILHIEGRKFCVSPRIKRLKKWMKKN

>ggaCCL30a

PTTCCFSYVQRPVPRNLIASAYITSSKCRLPAVILVTKKGREICVNPEESWVQKRLELL

>ggaCCL30b

PSTCCLSYVQRPVPRNLIASAYITSSKCRLPAVILVTKKGREICANPEESWVQKRLELL

>ggaCCL30c

PSTCCFSYVQRPVPRNLIASAYITSSKCRLPAVILVTKKGREICANPKESWVQKRLELL

>ggaCCL30d

PTTCCLSYVQRPVPRNLIASAYITSSKCRLPAVILVTKKGKEICVNPEESWVQKRLELL

>ggaCCL31

PDKCCFNFHTRRIKMDNIVACYATSPQCPHRAVVFKVKNGKEICTPADRMWVKRYQQRF

>ggaCCL41

VPTCCTTYITHKIPRNLIQRHYSTSTSCSKPAIIFITKKEREVCANPSDPWVQRYLQSV

>ggaCX3CL1

CSKWCISFHRAIDQRQIKSYRETEPQCTKKAIIFTTKRNREICANPYEPWVEKIVKKL

>ggaXCL1

RKLSCVNLSTQKVDIRSIVNYEKQKVPVEAVMFITANGIRICVHPEQKWVQSAMKRI

>tguCXCL8a

ELRCQCINTHSKFIHPKFIQNVNLTPSGPHCKNVEVIATLRDGREVCLEPSAPWVKLIIKTI

>tguCXCL8b

ELRCQCIATHSRFIPPKSIQDVKLTQSGPHCKNVEVIATLKDGREVCLEPTAPWVQLIVKAI

>tguCXCL12

TYRCPCRFYESNVARANIKHLKILSTPNCSLQIVARLKSNSKQVCIDPKLKWIQEYLEKA

>tguCXCL13a

NLSCRCAKTTSEYISPKKYESIEIRPVGSSCRRVEIIIKLRTSGKVCVNPEAPWVKKLLKRI

>tguCXCL13b

NLSCRCLKSTRAFIPPERYSSIEVWPVGSSCRRPEVVLTLKKNKRVCVTPEAPWIQLLIHKL

>tguCXCL14

gVKCKCSRKGPKIRFSNVRKLEIKPRYPFCVEEMIIVTLWTRVRGEQQHCLNPKRQNTVRLLKWY

>tguCCL1

KAHCCSKEMSSRRKIPEFKIQGYLETPSTCTYRAVLVQLQKGMVCVDPEKKWFQEYLRKQ

>tguCCL4a

PTSCCFSYISRQLPRSFVKDYYETNSQCSQPAVVFITRKGREVCANPAEEWVQQYVNEL

>tguCCL4b

YGPCCVEYITRPLPLSRVVKYEHTGSHCSPPAVIFTTIKDKLVCANPNDKWVQDIMNEL

>tguCCL5

MALCCFSYISRKLPQNHVQEYFYTSSKCSQPAVVFVTRKKREVCANPDARWVKEYVNSL

>tguCCL17

PAECCFDYVKGVLRLEILVGFYSTSKECFLPAIVFDTKKKAKICANPEEKWVKRAVRVL

>tguCCL20

NQDCCLSYTKARLPRKALKGYTEQLPSEVCDIPAIIFHTASGRNACVNPKEGWVKKHLLFL

>tguCCL21

ALDCCLKHSTLKKDIPSGVVIAYRQQGPETGCYLRAVVLITKKNKKICVSPTDDTVLKLMQQL

>tguCCL28

AFNCCMKISDEISKGILRRVERFEIQKAGGLCHLEAVILHMKGRKFCVNPWNKKVKKMMKKM

>tguCCL30a

KTMCCFSYISRRIPRSVISSAYITSNTCSMPAVVLITRQGKKICADPKADWVQKHLKHL

>tguCCL30d

PSTCCFSYQRQPIPLRRVSSVFVTSSSCSLPGVIVVTQKKKQVCADPRAAWVQQLQKHF

>tguCCL31

PDKCCFNFQTRRIKRDNVVACYPTSPECPHQAVIFKVRNGKEICTQASRPWVKRYQQSF

>tguCCL41

IPICCFTYSQHKLPWKLIQHHYITSSSCPQPAVVFVTKEGRQVCANPENAWVRRYLKIL

>tguCX3CL1

CSNECRNFTRRLPEKLIRSYRETEPGCGRSATILITLKFKEICADSTEDWVKKIKDKL

>tguXCL1

RKFKCVNLSTRQLNIRNLVNYEKQQVPTDAIMFLTAGGIKICVRPNQEWVQAAIKRI

>aplCXCL8a

ELRCQCIETHSKFIHPKFIQNVNLTPSGPHCKNVEVIATLKDGREVCLDPTAPWVKLIIKAI

>aplCXCL8b

ELRCQCISTHSKFIHPKSIQDVKLTQSGPHCKNVEIIATLKDNREVCLDPTAPWVQRIVKAI

>aplCXCL12

TYRCPCRFYESNVARANIKHLKILSTPNCSLQIVARLKSNSKQVCIDPKLKWIQEYLEKA

>aplCXCL13a

NLSCRCVKTTSDYISPKRYESIEIRPVGSTCRRTEIIIKLRTSSKVCVNPEAPWVKKLLKRI

>aplCXCL13b

NLSCRCAKTTAAFIHPSKYESVEVRPVGSSCRRLEVVIKLKSLERVCVDPNAPWVKKLLQDL

>aplCXCL14

gVKCKCSRKGPKIRFSNVRKLEIKPRYPFCVEEMIIVTLWTRVRGEQQHCLNPKRQNTVRLLKWY

>aplCCL1

YSKCCYKFVKNISASSIRSFKYTLPNCSRRAELLQVMELLDGTKVCADPRKEWFQKYLNRQ

>aplCCL4a

PTSCCFTYVQRELPRSFVTDYYETNSLCSQPGVVFITRKGREICANPEHDWVKKYVIEL

>aplCCL4b

SGPCCVKYSSKSFSSRHVTMYQNTSSHCPQPGVIFTTFKGKSFCGNPKDEWVQNILKQH

>aplCCL5

TTICCFSYTSQKLPQSHVKDYFYTSSKCPQPAVVFITRRTRQVCAKPDARWVKEYVNFL

>aplCCL17a

PSECCFEFLKPALRYEVLKDFYETPKECFSPGIVFETKNGNKVCAKPNTPWVKKAMEKL

>aplCCL17b

PTECCFDYAQKPVRIANIKSFYTTSNDCALPAVVFVTAAGFKVCNNPQKPWVKKTLNKL

>aplCCL19

VLDCCLRTSENPIPRRIVQRYQIQLVQDGCEIPANVFITVRGKRLCAPLEAPWAVRLREKL

>aplCCL20

NQDCCLSYTKARLPRWAIKGYTEQLSSEVCDIDAIIFHTFSGLKACVNPKDVWVKKHLLFL

>aplCCL21

ASQCCERTSLEVIPSFLVKSYRIQGPESGCRLHAVVFTTRKEKKICSSLTSRAVQKLIRRL

>aplCCL28

AFNCCTKISDEIPKGILPRVERFEIQKADGLCHLEAVILYIGDRKFCVSPQIKRVKKWMKKK

>aplCCL30a

PTTCCFSYQQRPVPRSLIASAYITSTSCSQPGVILVTTKGRDICADPQATWVKAHLKHF

>aplCCL30d

LTKCCFRYLKRPVPRSVIASAYITSTSCSQPGVILVTTKGRDICADPQATWVKAHLKHF

>aplCCL31

PNKCCFNFQTRRIKRDNVVSCYATSPECPHQAVIFKVKSGKEICAQAGRAWVKRYQQSF

>aplCCL41

HPTCCFSYTSHKLPKKLILRYYVTSTSCSLPALVFITKKGREVCANPSDTWVQRYLQNM

>aplCX3CL1

CSKVCNRFTRILPEKRIKSYHKTEPQCAKQAIIFITLASLEICADPKNDQVQKIMRKL

>aplXCL1

RKFSCVTLSTQKLDIRQLVNYEKQQVPLNAVMFITAKGIKICVHPDQKWVQVAMKRI

>acaCXCL8a

ELRCQCISTHSRFIPPRNIQDVKLTQSGPHCTNVEVIATLKDGREVCLEPTANWVKVIIKAI

>acaCXCL8c

ELRCRCIQKVSEVIAPKHFANIELTPEGPHCPVSEVIATLKSGKEVCLDPTSRWVKMIISKI

>acaCXCL12

TYRCPCRYVESSVAKSHIKHLKILTVPGCSLQIIARLKNSSKQICIDPKLKWIQEYLEKY

>acaCXCL13a

NESCKCLRVRSEFINPTKFARVEILPAGILCQRMEIIITLKGNRKVCVNPESKWVQVLVKLI

>acaCXCL13b

VGRCRCLKQTSSPFSPRQLKSIQVFPHGMQCQNTEIILTLKNKWKVCVDPSAPWVQELLKVV

>acaCXCL14

SKCKCLRKGPKIRFSDIQKLEERPKYPYCKERMIIVTMKSRFRGGHQYCLHPKLPSTKRLLKWY

>acaCCL20

NQDCCLSYTKRPLPRRVIKGFAEQLSSEVCDINAVIFITKNGLRACANPKDQWVKKHLQWL

>acaCCL28

IVDCCTEVAHHVPEKWLRRHVLRFDVQKGDLCKIPAVILYTKRKKLCASPHNKNVKRWVRRM

>acaXCL1

ALSSCIPLQSTEINIRRIIDYIEQTRPVKAVILITRRGVKICVPHNLPWVKETIKKL

>xtrCXCL8a

GLRCQCIHTHSAFIPPRLYKSVELIPSGPHCKNVEVIITITSGERVCVDPSQRWVQRIINSI

>xtrCXCL8b

ELRCLCIKTESKPIHPKHIKNIEVIPNGPHCKNVEVIVTLTNMEEVCLEPSAPWVKKIIDKI

>xtrCXCL8c

ELRCLCITTEKKPIHPKHIKNIEMIPKGPHCKNVEVIATLTSGDDVCLEPTAPWVKRIIEKI

>xtrCXCL8d

ELRCLCITTEKKPIHPKHIKNIEMIPKGPHCKNVEVIATLTSGDDVCLEPTAPWVKRIIEKI

>xtrCXCL8e

ELRCQCISTVSTPFHKKHIRSLEVTPNGPHCPRVEVIVTLRNGVQHCLNPKAKWLTTVVKKI

>xtrCXCL10a

KQRCMCKGRGAERISTQHLKKLEVFPMSFGCDEIEVIVTMKSGSKICLNPQSKFANKLLSAL

>xtrCXCL10b

NRRCLCKRHVAKRFDLRSLKKVAVFPISPGCENVEIIATLKSGQHICIDPESKSINKLIFAL

>xtrCXCL10c

GRRCLCKKMSKKLSPKRLIKIEIYPAGYRCENIEYVATMKGSKKTKCFSPNSKLLKEIMSPK

>xtrCXCL12

VYRCPCRYFESNVPKSNIKHLKILSTSNCSLQIVARLKHNGKQICLDPKTKWIQEYLEKA

>xtrCXCL13a

GGRCKCFKQTNSFIKPSKLTRVEFFPPGRSCPQLECLVTLKNGDIVCVNPQAAWLQRLIAYL

>xtrCXCL13b

GRKCRCLKQTKKRPSSIHRIQVFPESYKCRTQEVLVFLKNKQIVCVDPEARWIQVLIFNS

>xtrCXCL14

gSKCKCSRKGPKIRFTDVQKLEIKPKYPYCEERMIIVTMQNVSRFRGQQYCLHPKLHSTKKFLKWY

>xtrC(X)CL16

AGACCFDMKPRDPPTDALFRLYKEKVKGFEECPHYRVQFKFDKGKICASKHDAWVEKLICHL

>xtrCXCL18

VPRCQCHQTINEIHQKHIKSFKINIPYEYCKFTEIILTLKDSRDVCLNRQAKMGRNLIRCW

>xtrCCL5

VVSCCFDYIKKPIPPKHVADYFYTSSRCSRFAVVLVTRKNRKICANPEDEWVNNIINVL

>xtrCCL19

PVDCCLRTNNKQIRWQNIRSYFRQDESSGCKIEAVVLITRRNKHLCTPPHEAWVQAITDRL

>xtrCCL20a

DYDCCTSYTQKKIPQKIIKGFYIQRSSEVCDIDAIVFEVAYKSPGNRNVLKSKLCADPKQKWVESRIEEL

>xtrCCL20b

MYDCCYTYTRKQLPLKIIKGFTIQNSFEVCDIDAVIFITRKFRVCANPKEQWVINATDAI

>xtrCCL20c

DYDCCTSYTQKKIPQKIIKGFYIQRSSEVCDLDAIVFEVAYKSPGNRNVLIKSRLCADPKQKWVESCIEEL

>xtrCCL25

FENCCLSYAKVTQYSGLYKHIRYYQLQEISESCNMRAVIFYLKKRIICANPMEQWVGLAIKQF

>xtrCCL28

SISCCTEHSDQISRGLLQRVRKFEIQQNNGLCNIRAVVLYTKHKVLCANPDNVLLNQWIEKR

>xtrCCL34

IVSCCTRVSSRKPKDVLVNFLIQKEDLPCVEAILFTTNEGKFICSRPKAPWVSKKMKEI

>xtrCCL42a

STSCCFKFAKKLLLGRIQSYYPTSGSCPNPAIVFVTKNGKVCAKPNDSWVIDYQNRL

>xtrCCL42b

PTSCCFKFIKKWLSPKHVKSYYNTSSFCPHSAVVFTTKKGIKMCAKSSDKWVTDLIIQL

>xtrCCL42c

PTSCCFHYAKKRMSVGLFKDYYITSSFCSNPGVIFRTNNGNKICAKPSDKWVTDYMTIL

>xtrCCL42d

PTSCCFNYAKKRMPIGLFNDYYNTSSSCSSPGVIFRTIKGHEICAKRSDKWVIDYMTRL

>xtrXCL1

FGKICLETKLMNKIKCKMLKSYVQQTSPVAAIMFTTQKNITICANPEQPWVKQAVQCL

>xtrXCL2

RGPVCLETSSPKPLSFILIKDYIEQTNPIKAIMFTTKKDKLICANPEENWVKKAVQYL

>olaCXCL8aa

ELHCRCIQTESKRIGRHIQKIEMIAPNSHCEETEIIATLKTGQEVCLDPEAPWVKKVIQKI

>olaCXCL8ab

NLRCQCITKEKTPIGRLIGAVEVNPASSHCKEVEIIATFAKNGKKICLDPEAPWVKKVVNAM

>olaCXCL12a

AERCSCRSTVNNVPRSFIRELKFIHTPNCPFQVIAKLKTNRELCINPEARWLQQYLKNA

>olaCXCL12b

VERCWCRSTLNTVPQRSIKELKFLHTPNCPFQVIAKLKNNREVCINPATKWLQQYLKNA

>olaCXCL13

RGGCICLRVSRSAVPAKAVSKVEMIPPNGRCRKTEIIITRKNNVRVCAVPQAKWVQDLLRTL

>olaCXCL14

aYKCRCTRKGPKIRYKDVQKLEIKPKHPFCQERMIFVTMENVSRFKGQEYCLHPKLQSTKNLVKWF

>olaCXCLC18a

PGRCLCPQTIPGVRGRLKELLVLPRSASCNTITVIVTMMNNARVCLNPEQPMAKQLIRCW

>olaCXCL18b

VGRCRCYEEVRLMAIKRNITDFQVKEKSAACSKIQLIVTFMEANSTAVERCVKPQGYKAKQLLKCW

>olaCXCL19

NTRCQCLKVESRIIPPDSLKSIKLVTEGPHCPETEVIAGLVTGEKVCLNPRSAWVKKLVQFV

>olaCXCL20aa

SECHCLRTSKTVQPSLIKKVQEFLPRPYCSKLEIIVTLKNNVKVCLDPTHKFAKAVLQSI

>olaCXCL20ab

iLKCHCLRTSKTVQPSLIKKVQEFLPRPYCSKLEIIVTLKNNVKVCLDPTHKFAKAVLQSI

>olaCCL19aa

PVDCCLSVKNQTIDKIVVADYYPQAKGCALDAT**I**LVTRRKKTLCVPHDEQWVQDVKKHV

>olaCCL19ab

PKDCCLSIRQEKLNPKNIVSYSIQEEGDGCQISAS**V**FVNKHGKKLCVAHPKDFPWAQKIMN

>olaCCL20aa

NLSCCTAYNKMQLPFQRIRGYREQTSNYCHIEAI**I**FYTINNRQFCANAKETWVKHSLELL

>olaCCL20ab

AKLCCTRYQSTGNHFPVQRLKSYRVQEDTGNCNIRAI**I**FLTVKGRVFCANPNQQWVIEAVETL

>olaCCL25a

YDDCCLKYVRKLSRGTQKHAIAYKIQKADGGCNISAL**V**FIMKRGRMICANPNETWAVTLKENL

>olaCCL25b

YDNCCLGYVRELGPRKRNNIVSYRIQETDGDCNIRAV**V**FLMKKRRGLAKQRTVCANPEDPWVQEKMMDL

>olaCCL27

TH**G**CCYNIGQFQPKKTQRITSIGIQHASGRCQINAV**V**VHLDGRETPICVHLKREKQIRKLKRK

>olaCXCL32aa

HDCKCVRPRDTQIPKTKLVSYTIQEESICNVRVV**I**FTTVKGKILCSDPNSSWTKEGMRTL

>olaCXCL32ab

KGCECLRWYDTKVNISLIKSYTIQEETKCSVRVI**K**FKTVRGKTICSDPNNKWTKNTMKKL

>olaCCL33aa

PGQCCFDFKNIPPQKIVKDIIQTHRLCSKNAF**I**VIPVKGKPICMKDDVEWVKELFNLK

>olaCCL33ab

PEECCFSFSTKDNFSENRVTAIIRTHPHCSNKAF**I**VSTVAGKKICFRDNSKWAVDTFNKF

>olaCCL33ad

PEKCCFKFSERQLPLKRVTGIIRTHHSCQHQAF**V**VSTVTGREFCFRDNFKWAVDTFNGI

>olaCCL33ae

STLCCFKFSKIPIPFVLVVNVKKTSASCPIQGF**I**VETRKRKICVSKTSIWGHNFFNRV

>olaCCL33af

LEKCCFEFSEEPLELRRVTGIIRTPQRCQHQAF**I**VFAIGREEFCFRDNFDWAMDVCKYF

>olaCCL33ag

PEKCCFVFSENPVPPKKVTAIIKTHHSCQHEAF**I**VSTEAKKEFCFKDNFQWAVDTFKKL

>olaCCL33ah

STLCCFKFSKIPIPFVLVVNVKKTSASCPIQGF**I**LEKAKICVSKTSIWGHNFFNRV

>olaCCL33ai

HGPCCFEFREKQLPPKRVTGIIRTHPRCQHKAF**I**VSTVRKEFCFRDNFQWAVDTFNKF

>olaCCL34a

VKSCCTKLSIANISAPIIGYRIQKKNLPCIRAV**V**FETTEGEICSHWKQDWVYEKIMEL

>olaCCL35a

PKKCCFRFNETPMEKENVVAYMKTSQRCSRPAV**L**LKTAAGRQLCVRPSAPWVKEVISYL

>olaCCL36aa

PDNCCFKFFPGTLSASRVRSFTLTDDRCPKPGV**I**LETKKKINICVKQNAAWVQEILQML

>olaCCL36ab

PDNCCFKFFQGTLSPSRVRSFILTDDRCPKPGV**I**LVTKRNLNICVEQSATWVQDVLQTL

>olaCCL40aa

KDGCCHQPQKINIPKTKVKHVQMTPSGCSTKAI**I**VTSKLERKFCLDPDWAPAKKLLQKF

>olaCCL40ab

KDGCCHQPQKINIPKTKVKHVQMTPSGCSTKAI**I**VTSKLERKFCLDPDWARAKKLLQKF

>olaCCL44

DVQCCMLYSQGKVRTKDVLRFEVQTEGPDCSIQAI**I**FYTKKAVKCADPRDRKVKRLLRKL

>gacCXCL8a

EQHCRCIQTESKIILRYIEKVELITANSHCDEAEIIATLKKTGQQVCLNPEAPWVKKVIQRI

>gacCXCL12a

VERCYCRSTVNSVPKSFIRELRFIHTPNCPFQVIAKLKSNKEVCVNPEIRWLQQYLKNV

>gacCXCL12b

VERCWCRSTLNTVPQRSIKELKFLHTPNCPFQVISAKLKSNREVCINPETKWLQQYLKNA

>gacCXCL13

TRGCRCIRTTSDPVPTRVIRRIEVVPVSGLCRRTEIIVTRRNGSKLCVNPDEAWLHVLLSKP

>gacCXCL14

aYKCRCTRKGPKIRYKDVQKLEIKPKHPFCHEKMIFVTMENVARFKGQEYCLHPKLQSTKNLVKWF

>gacCXCL18a

PGRCLCPHSQPGLRGKLKALTVYPKSPSCDRLTVIVTLQSTNESVCLNPEAPMGKQLIHCW

>gacCXCL18b

LGRCSCLNPIMFIKGSISDFEVLESRPGCDKTELIVTMNNPNNSTEKLCMNTVGKRAKAFLRCW

>gacCXCL19

NTHCRCLQVESRIIPPDSLRSIKLVPEGPHCPDIEVIAGLADGRKVCLNPRAAWVKKLVHFV

>gacCXCL20a

ILNCRCVKNSDAVSRHLIARIKQLPPRWYCNREELIAVLKDGREKCLAPNGRFAQAIKRYR

>gacCCL19aa

PMDCCLRVGNNTIAKQRVVNYFLQIGGQGCAMDAM**V**LMTRRKKNLCVPADEPWVKEVVKHV

>gacCCL19ab

VVDCCLTVAEKPLPLQILRSYTIQEAGKGCSISAT**G**FVTKLGRTLCVSHPNNKLWVRNYIK

>gacCCL20ab

NYGCCRSYMTSRIPFSRIKGYSVQTMKEMCSINAI**I**FHTMKGKGCTDPALNWVMQYVNRL

>gacCCL25a

FEDCCFKYVKKHIRRTQKLAVDYRRQVVDGGCNIPAT**I**GGRLLCADPTRKMVVHLMKRL

>gacCCL25b

YGNCCLGHHTRIAERVKKNIESYRMQETDGDCNIRAV**V**FLIKKKPSHAKQLTRCANPDDLWVQKLMKTV

>gacCCL27

IPKCCIRTKNHIPLQMLRKVQRWSMQSGGRACDISAL**L**LHVKDMRTPVCAHPKVLGILKKVRQQ

>gacCCL32a

VSSCCLGWSTRKVPPRCVVNYTVQTDAACSINAI**V**FRHINGGRICSDPNSDWAKQVILKV

>gacCCL33aa

HGVCCFTFSTVTVPKNNIVSVTKTHHSCPVKAF**V**VTTVKGRQICVGHYVNWAQKAFKQQ

>gacCCL33ab

HGVCCFTFSTVTVPKNNIVSVTKTHRSCPEKAF**V**VTTVKGRQFCVGHYVNWAQKAFKQQ

>gacCCL33ac

PVSCCFEFFTGRVPSKQIVSVMETHSSCGAKAF**V**VTTAKRRKICVGHYVNWAQEAFKKQ

>gacCCL34a

IIKCCTEVSVANVTAAILGYRVQRKKPPCVKAV**I**FETTDGEVCSHWKQEWVFAKVKEL

>gacCCL35a

PKRCCFRFNENKVPSNNVVGYTKTSQRCSNPAI**L**LQTVTGRKLCVRPSAAWVKELINAK

>gacCCL36a

PDHCCFTPYPRRLNKKLIRSYYMTDHRCPKSGV**I**LITQKGRHICVDPDLSWVGNIMSSV

>gacCCL40a

NNGCCVSTSHVHVPRGKVIHIGMSSRECPVTAI**I**ITTEKRQFCIDPDLKWAKTQLAHF

>gacCCL44

DVQCCMLYSQGKVRTKDVLRFEVQTEGPDCSIQAIXLYTKKAVKCADPTDRKVKRLLRKL

>dreCXCL8a

DPRCRCIETESRRIGKHIKSVELFPPSPHCKDLEIIATLMTTGQEICLDPSAPWVKKIIDRI

>dreCXCL8ba

PLRCQCVKIYSQPPIPRRQVLALKVNSAGPHCRNEEIIATLKNGQTCLNPTENWVMSLKTQG

>dreCXCL8bb

PQRCQCIKTHSKPPIPKRQVLGLKVTPAGSHCRNEEIIATLKKGQICLNPTETWVISLKEKF

>dreCXCL8bc

QLRCQCVKTYKGKPINPKLIQSLQTIPAGARCKNMEVIATVKNGKTCLNPKDEWVTKIIEGR

>dreCXCL11aa

SQRCVCLGAGLNMVKPVLIEKIEILPSSPSCGHMEVIATLKNGAGKRCLNPKSKFTKKIIDKI

>dreCXCL11ab

INRCSCVGKGLDRVVLRNIEKFEIIHPSPSCGKQDVTMKSSLQKCLNPEFKFTKPLIRRA

>dreCXCL11ac

RARCFCADKGINMVLLKNIEKVEIFPPSPSCNKNEIVVTLKNGAGQKCLNPDSKFTQNVVLKA

>dreCXCL11ad

RSRCLCADKGVNMVSPKLIEKVDIIPPTPSCGNLEIVVTLKNGAEPKCLSPDSKFTQKYLMKA

>dreCXCL11ae

FNRCSCVGKGLDRVALRNIEKFEIIHPSPSCGKQEIIVTMKSSEQKCLNPESKFTQELIRRA

>dreCXCL11af

RARCFCVDKGLNMVLLKNLDKVEIFPPSPSCNKHEIVVTLKNGAGQKCLNPDSKFTKNVVLKA

>dreCXCL11ag

RARCFCVDKGLNMVLLKNLEKVEIFPPSPSCNKHEIVVTLKNGAGQKCLNPDSKFTKNVVLKA

>dreCXCL11ah

KNRCLCADKGANNVNLKTIEKIQIIHPSPSCKRLEIVVTLMKGAGKKCLNPESNLGKNILKAL

>dreCXCL12a

VERCWCRSTVNTVPQRSIRELKFLHTPNCPFQVIAKLKNNKEVCINPETKWLQQYLKNA

>dreCXCL12b

VERCWCRSTLNTVPQRSIREIKFLHTPSCPFQVIAKLKNNREVCINPKTKWLQQYLKNA

>dreCXCL13

ENKCKCQTTTSSRIPPRLFQKIEILPAGAHCRKAEIIITKKDNQAVCLHPEARWVKEMVSKI

>dreCXCL14

aYKCRCTRKGPKIRYIDVQKLEIKPKHPYCQEKMIFVTMENVSRFKGQEYCLHPRLQSTRNLVKWF

>dreCXCL18aa

REKCECVKEAGAVQWRKITDYTITPKNPLCNKVQIKLQLSNKEVCLNPESKQGKKLQKCW

>dreCXCL18ab

PKTCQCPQVQKRVRGPFSDLRITPKGPSCLQNEIIVTPKKTNKPVCLSPEGPQGKSLMKCW

>dreCXCL18b

PDRCQCEESSLVNRARRDTIKEFYITPKRPNCDKVEIILTQKPENKTTASGQLCLNPQKQQGQLLQNCW

>dreCXCL19

NSRCVCLKLESRVIPQDNLRRVVILPRGPHCKTTEVIAGLTSGERICLNPRTHWVKKLIMFI

>dreCXCL20b

SQRCRCIGKPYKTVNPRSIQAVDVFQPSPSCSNKEIILTVVEGRGKTKGKGSRKRSKVCLDPNGKQGQRLLKGR

>dreCCL19aa

ALDCCLTVSPRVIPKHVILAYQKQSRGDGCPRDAV**I**FITRKGLNLCAPPASEESWVRDTMT

>dreCCL19ab

AVDCCLTTKDTRIPLQIVASYFHQTTESGCPIAAT**V**FITKKDKKLCAPPEKNTWISRIISH

>dreCCL19b

AVDCCLTTSDRRIPQKVVTTFTLQTGEGGCRVPAT**I**FVTKKGLKLCAPFPSQNNWVSRLID

>dreCCL20aa

NYACCVKYTRTPLPFGVIAGFIEQSSLEVCRIDAI**I**FITQKNKKICASIEDQWVRAALAHL

>dreCCL20ab

NYACCVKYTRTPLPFGVIAGFIEQSSLEVCRIDAI**I**FITQKNKKICASIEDQWVRAALARL

>dreCCL20ac

aV**S**CCRKYTKGMIPMSLIKGYSIQTITRSCHINAV**I**FHTNGGKNICTDPSKGWVMESIRKL

>dreCCL20ad

PMRCCTMYSIHSLPLNRLMDYTLQDTTKACNIKAV**I**FTTIKNRQICANPDDPWVQKAISHI

>dreCCL20b

sAICCLRYVKNPRRCGFLKGYDIQIMTEGCDLPAIIFHTVTGRSICANPSQNWTQERVLCL

>dreCCL25a

YEDCCLKYVRKINHSMKNRVIRYRKQEVDGGCNIPAI**V**FTLKQGRMFCTDPREKWVHELMQRV

>dreCCL25b

YENCCLKYVTGIKKNMRRNIMSYRVQLTDGGCNIPAV**V**FKMRLKKQLKPKSVCADPRSDWVQAIIKEL

>dreCCL27a

IPTCCLSVLRRIPKRVLRSVRTYEVQDTSGHCEIKAL**I**LHFKGKKICAHPKLERFLKKMLKH

>dreCCL27b

TPRCCVETTKRFPLDLLKKVNRYEVQTSSGACTIDAL**V**LHVGDMRYCATPKMEQFLQKLMKR

>dreXCL32aa

HKTGCLSTTDTKTPHTNLRSYTIQQKPLFPVHAV**R**FLTLKGITICSDPTSPWAIKAMKHL

>dreCCL32ab

PMSCCLRLRDRKLHLDKILNYRIQTEDLCPIRAV**L**FQTVAGKTLCSDPESSWTKSAMWKV

>dreCCL32ac

PPGCCLTVKNIRIPAFNIVGYSIQEIPLCSIKAV**R**FYTKKNRVICSDPNSDWAKKVIQQL

>dreCCL32ad

PANCCSLKDTKIPAENIVDFNIQEAPPCHIKAI**R**FYTRKNKVICSDPNSHWAKKMIEKL

>dreCCL32ae

PMSCCLRLKDRKLHLDKILNYRIQTEELCPIRAV**L**FQTVAGKTLCSDPESSWTKSAMWKV

>dreCXCL32ba

SNCPCLKLSDGVLRKANIKSYIRQRAGVCHIDAIVFTTVRGITFCADPKLTWVIDAMKFL

>dreCXCL32bb

SYCPCLKTSDIVLRKANIKSYTRQRADVCYIDAIVFKTVKGKTFCADPKKTWVKDAMESL

>dreCCL32bc

RLNCCLRTSKSSIPIKRVVDYRVQQPGICPIEAVILVTVKGKRICCDPNTEWIKKTMRKV

>dreCCL32ca

PVSCCLRHGDRRPHLDKILNYRIQTEELCPIRAV**L**FQTVAGKTLCSKPESSWTKSAMWKV

>dreCCL32cb

PVHCCTKVKNVRISVEKIVSYSIDEPPLCPIKAV**S**QAKQSYCSDPNSDWAKRVIYNL

>dreCCL32d

PFLCCVTVKNIRIPACNIERYSIQKPPLCPIKAV**R**FHTKKNKVICSDPNSDWAKKVIQQL

>dreCCL33aa

PEFCCINFIDFPIPANKIVSAVITPSRCSSKGIMVTTPRTQFCVKPDEDWIKPIMEKQ

>dreCCL33ab

PITCCFAFIDFPIPYNKIVSALRTSPRCATKAIVVTTPRTQFCVKPNEDWIRPIMERK

>dreCCL33ac

PEPCCFNFIDFPIPANKVVSAVRTVSRCAVKGIVVTTPRTQFCVKPDEDWIKPIMEKQ

>dreCCL34aa

FVQCCTSVSTQKIILPITGFKLQIRNPPCVKAV**I**FFTTEGPRCCHWKEGWVKEKIQEL

>dreCCL34ab

FVQCCTSVSTKEITLPITGFKYQKRNPPCVKAV**I**FFTKEGEQCIHWNQSWVREKIQEL

>dreCCL34ac

FVQCCTSVSTQKIILPITGFKLQIRNPPCVKAV**I**FFTTEGPRCSHWKEGWVKEKIQEL

>dreCCL34ad

FVQCCTSVSTKEITLPITGFKYQKRNPPCVKAV**I**FFTKEGEQCIHWNQSWVREKIQEL

>dreCCL34ba

VESCCTPVSTPELTDPIMSVRIQFESLECETAI**V**FKTEERELCSDPRQLWVRRKVMQF

>dreCCL34bb

VMPCCTSVSTAEVTDPIISVGIQRESPPCGTPIIFETKEGKICSDPRHEWVLRKVVQF

>dreCCL34bc

MMLCCTSVSRLEVTDPIIGFRIQRESRPCVNAILFETERGAFCSSPRQPWVRSKVMQF

>dreCCL34bd

GVTCCKEVSRGRIPPDIKLTAYKHQPALSPCVDAIIFYAEKERYCTDPKARWIQNRLQGL

>dreCCL34be

VINCCKSVSTVKVTDPIIGVRIQRKSLPCVNAI**I**FETDRGHFCSDPRQPWVQRKAQQF

>dreCCL34bf

GTNCCKSVSTVEVTDPIIGVRMQHQSLPCVNAIIFETDRGDFCSDPRQPWVRRKVKQF

>dreCCL34bg

EPVCCKTLTTNEPQIKINSCYFLQETSDCLKCVLFVDDMNRMYCIDLTAPWLSERIKCL

>dreCCL34bh

DTVCCRTLTTNEPQIKINSCYFLQEISKCLKSVLFIDVKNKMHCIDPTAPWLEARIKRL

>dreCCL34bi

GMVCCKKFTRKEPQIKINSCFFVQEISNCLKSVVLIDEMNKMHCIHPKAPWLNARIKRL

>dreCXL34bj

GKICFRNPTTEEPQIEIRACFNLRKTRHCPACVLFIDKKNRMCCINLKAPWLSAKIEHL

>dreCXL34bk

PGVCFKVLTTKEPKANIKRCYNLPKTNNCLKCVLFVDASNRMKCIDPNASWLAERLYRL

>dreCCL34bl

PSTCCEKHETNLPKIQLKKCLILPATDKCLESVLFVDIRNRRHCFSTTAPWINERIAIF

>dreCCL34bm

PKTCCRKYEKNAYFKIQRCYILPETDKCLNSIVFTDPNNRNHCYDPTAPWMTIRMNLL

>dreCXL34c

PSLCFQVLTTVEPRKNITSCYNLSKKGNCLQCV**L**FVDAENRMMCMDPNASWLPARLNRL

>dreCXL34d

PKLCFQVLTTEQPKANITSCYNLPKTSNCLECV**L**FVDATNRMMCIDPNASWLSERLNRL

>dreCCL34ea

GTNCCKSVSTVVVTDPIIGVRMQNQSLPCVKAI**I**FETDRGDFCSDPRQPWVRRKVMQF

>dreCCL34eb

VINCCRSVSTVKVTDPIIGVRIQRKSLPCVNAI**I**FETDRGDFCSDPRQPWVRRKAQQF

>dreCCL35aa

PKRCCFQYAERQVPFKQVMEYSMTSQQCPKEAVLFKTARGRYVCARPSDPWVQEYMQAI

>dreCCL35ab

PKRCCFQYAERQVPFKQVMEYSMTSQQCPKEAVLFKTARGRYVCARPSDPWVQEYMQAI

>dreCCL36aa

GTDCCYNFFKRKIPLSKINSYSLTRVDCTMPGV**I**FVTQKGLRLCVEPKLNWVKKTIQII

>dreCCL36ab

PDACCFTFFKRKIHPSKINSYNPTRVDCTLPGV**I**FVTQKGLRLCVEPKLNWVKKTIQII

>dreCCL38aa

PEKCCFSFTNARIPLKQIESYYTTHLQCNMNAVIFIIRAQREICTNPTEKWVRRLMKMV

>dreCCL38ab

QIECCFSFSTVRIPVNQVQSYQTTHFECHKKGIVFITKIQKEICTDPTEEWVQRLMGLV

>dreCCL38ac

PVKCCYSFFNARIPVKEVGGYHATHLQCNINAVIFITKAQREICTNPAEKWVQRLMRLV

>dreCCL38ad

PDKCCFSFSTIKIPVKQVQSYHTTHFQCQKNGIIFVTEQKEICADPTERWVQRLMNLV

>dreCCL38ae

PDKCCFSFSNTRIPVKQVESYHTTHLLCSGNGVIFITKAQREICTNPTEKWVQRLMKLV

>dreCCL38af

PDKCCFSFSNARIPVKQVVSYHTTHLQCNMNGVIFITRAQKEICTNPTEKWVQRLMKMV

>dreCCL39aa

NSVCCEGFTHKKIPLSKIVSHHLTTSNCAKKFI**V**FTTKAGKKICVDPENTFVKRQVAEL

>dreCCL39ab

KSVCCEATTHRNIPLKQIMSYQWTTSMSYKSHCV**GT**IAGREICVDPQNTLVKKQVAKL

>dreCCL39ac

NSVCCEGITHKKITLKQIVSYHWTTSSCAKKAI**V**FTTKAGKKICVDPENTFVKRQVVIL

>dreCCL39ad

KSVCCEGFTHKKIPLKQIVSYLWTSSNCAVKV**IV**YDKSRKKICVHPENNFVKRQVVIL

>dreCCL39ae

KSVCCEATTDKNIPLKQIMSYQWTTSTCPIKAIVFKTIAGRKICVDPQNTLVKNQVAKL

>dreCCL39af

NSMCCFGKGSNIKIPLRRLEYFYWTSSRCPLKHVVFVTIAKKHLCMNPDNEWVQKVINMK

>dreCCL39ag

NSICCFGNSNSRIPLKRLKYFYWTSSHCPFKHIVFVTIAKRHICMNLDNEWVQKVISMK

>dreCCL39ah

KGICCFGKGSNRRIKLNRLNSYYWTSNFCTLKRLVFVTTTKRNICMNPENEWVQKIIKEK

>dreCCL39ai

TSICCFGKGSNSKIPPNRLKSYYWTSSICPFNHIVFVTAKRHICMNPENKWVQKTMKAI

>dreCCL39aj

KSVCCEGITHKVIPLKQIMSYQWTTSTCPIKAIVFKTIAGREICVDPQNTLVKKHIANL

>dreCCL44

DVQCCMQYSHGKVRTKDVLRYERQTEGPDCSIRAIILYTKKAVKCADPRDRKVKRLLRKL

>tniCXCL8a

EQHCRCIETESRRIGRHIGKVELILPNSHCERMEIIATLKKTGEEVCLDPEAAWVKKVIERF

>tniCXCL12a

VERCYCRSTVSNLPRAYIRELRFIHTPNCPFQVIAKLKSNKEVCVNPQIQWLQQYLKNA

>tniCXCL12b

VERCWCRSTLNTVPQRSIKELKFLHTPNCPFQVIAKLKNNREVCINPETKWLQQYLKNA

>tniCXCL13

FSGCHCLRIFRRPIPFRIIKQVEMIPISGQCRRPETILTRRNGSKDCIDPNQQWFKDVLRKI

>tniCXCL14

aYKCRCTRKGPKIRYKDVQKLEIKPKHPYCQEKMIFVTMENVARFKGQEYCLHPKLQSTKNLVKWV

>tniCXCL18a

PGRCLCPQTQATVRGPLRALSIYHRNPTCNKVTVIVTLRNNDEVCLDPKAPLGKRLIHCW

>tniCXCL19

VTRCQCLQVESRIIPPDSLRSIKLIPEGPHCPTAEVIAGLASGAKVCLNPKSTWVKKLVQFV

>truCXCL20

S**P**ACRCLNTVAAVNPSHVVDVVEYGPRPYCRRQEV**I**VILKNKRPRCLDPKGQFAQGLLWAK

>tniCCL19aa

PADCCLSVTNAEVIKHAIVDYRRQVAGQGCTLNAT**I**LVTRRQKQLCAPASERWVEDVVAHV

>tniCCL19ab

VLDCCRTKTSKLLPLQLIRSYSVQDAGAGCDISAT**V**FVTKTGRQLCVSHPSEEKWVQKHID

>tniCCL19ac

VLDCCRTKTSKLLPLQLIRSYSVQDAGAGCDISAT**V**FVTKTGRQLCVSHPSEEKWVQKHID

>tniCCL20aa

SSGCCTRYSRRPVPFQLIKGYREQTTMENCHIEAI**I**FYTVQRKMICASRKDEWVRKLLKLL

>tniCCL20ab

**R**YFCCTQYHEKPVPVKMLKYYIIQEDTGYCNIKAV**I**FKTKTKPLCANPESWWVKIAMETV

>tniCCL25a

YEDCCLKYIKKVKPRIQKYAVSYRLQVLDGGCNLPAV**I**FVMKKGRVVCTDPKEQWVTELMRQI

>tniCCL25b

YGNCCLGYVPAMRRNAKNIERYWRQETDGDCNIRAV**V**FVMKKKQGQKKPRTVCANPEQTWVQSLMAHV

>truCCL27

IPGCCINTRKIIPINVLRKVSRWTIQSSGGACDIDAV**I**LHVRDKRICVDQTVFKDIWWRMKQ

>tniCCL33a

NVRCCAQFTEQPVPKRNVRKIYKTSHQCGQKAF**I**VETLRRELCYRQSFPWALKVYKEF

>tniCCL36a

PDDCCFRFYPHRVKRTLVRSYYATDQRCSKTAV**I**LVTQRSRHICVDPNLSWVETLLKNL

>tniCCL40a

QSLCCKSVRRMCVQETMVTKVVKNRCGAQKAI**L**VVRKNNQTLCLDSEWKWAQNLLEKF

>truCCL44

DVQCCMLYSQGKVRTKDVLRFEVQTEGPDCSIQAI**I**LYTKKAVKCADPRDRKVKRLLRKL

>cmiCXCL8a

YLRCRCIKTQSTFIHPKHITNVDLITNGPHCSVDEIIITLIKGNKICLDPNEKWVQMVINII

>cmiCXCL8b

SLRCQCVKTMSEFINPKFMKNIEIVPSGPHCSNAEIIVTLKSTNRVCLDPQAPWVKRIINRV

>cmiCXCL8c

NLRCNCIRTNSNFIHPKFMDHIDIFPSGPHCPVVEIIATLKSGNRVCLYPEASWVKKIIEKM

>cmiCXCL8d

NLQCQCIKPSSDFIRPTRMKEIDIIPSGPHCGNVEIIMSPLQIQNKSVCTLKHPGYKESLTR

>cmiCXCL13

ESKCKCIRTTSAFIHPRKYQHVDIFPQSTLCRRVEIIIRLINKRVVCINPETAWVKKVVSII

>cmiCXCL14

YKCKCIRKGPKIHYKKVKKVEIKPRYPYCQEKMIFVTMQKAARFKGHQYCLHPKLQSTKNLIQWY

>cmiCXCL22a

SAHCLCIQTTSRFIKLQNIQSLEYIPRRSGCESTEIIVTLKSNRKVCVNPDAKWVKVVIARR

>cmiCXCL22b

RARCLCTQTTSRFIKLQNIQSLKYIPRGTSCESTEIIVTLKSNRKVCVNPDAKWVKVVIARR

>cmiCXCL22c

RARCLCTQTTSRFIKLQNIQSLKYIPRGTSCESTEIIVTLKSNRKVCVNPDAKWVKVVIARR

>cmiCXCL22d

SAHCLCIQTTSRFIKLQNIQSLEYIPRRSGCESTEIIVTLKSNRKVCVNPDAKWVKVVIARR

>cmiCXCL23

GRRCKCINTINRLHPSMRINNVKILVQQDYCPNVEIIVNLQNGNKICLNPTSVIGKKIINLM

>cmiCCL25

YEDCCLSYNVVKHPKKLSRKIIHHRVQGTGGGCNLPAIVLTLKKSRIVCVDPEEKWLQMFLKKS

>pmaCXCL8

GGRCQCVHVISKFIHPKHFQTMEVIPQSSNCKNVEIIVTMKSTKNQICLNPDAPWVRKVISHI

>pmaCXCL15L

NPRCLCITSSSNFIPVKMLRNIEVIPKSSRCNKVEVIATLKTNIDQKICLSPAAPWVKALVSKL

>pmaCXCL21

DTRCQCADFYRKPLRLKSIRGLVDHRFTSSCHRDVIASLPNGRKVCLDPDKAWVKHILRRF

**B**. Amino acid sequences of vertebrate chemokine receptors in FASTA format.

Conserved DRY, CWLP, NPxxY(x)5,6F and TXP motifs (Nomiyama, H. et al. Dev. Comp. Immunol. 35, 705-715 (2011)) are indicated in red letters. Chemokine receptor domain pfam00001 (http://pfam.sanger.ac.uk/ family?PF00001) is indicated with blue letters. Transmembrane regions of human receptors are underlined. GenBank or Ensembl accession numbers and the chromosomal numbers are written in the comment lines. ‘Blast search’ indicates novel chemokine receptor genes identified in our study.

Species:

hsa, *Homo sapiens* (human); mus, *Mus musculus* (mouse); bta, *Bos taurus* (cow); laf, *Loxodonta africana* (elephant); mdo, *Monodelphis domestica* (opossum) ; meu, *Macropus eugenii* (wallaby); oan, *Ornithorhynchus anatinus* (platypus)

gga, *Gallus gallus* (chicken); tgu, *Taeniopygia guttata* (zebra finch); apl, *Anas platyrhynchos* (duck); aca, *Anolis carolinensis* (anole lizard); xtr, *Xenopus tropicalis* (Xenopus); xla, *Xenopus laevis*

dre, *Danio rerio* (zebrafish); ola, *Oryzias latipes* (medaka); tni, *Tetraodon nigroviridis* (Tetraodon); tru, *Takifugu rubripes* (Fugu); gac, *Gasterosteus aculeatus* (stickleback); cmi, *Callorhinchus milii* (elephant shark); pma, *Petromyzon marinus* (sea lamprey)

Genome sequence versions:

Human, GRCh37 Feb 2009; Mouse, NCBIM37, Apr 2007; Cow, Btau_4.0 Oct 2007, 7x coverage; Elephant, loxAfr3 Jul 2009, 7x coverage; Opossum, monDom5 Oct 2006, 7.33X coverage; Wallaby, Meug_1.0 Dec 2008, 2x coverage; Platypus, Ornithorhynchus_anatinus-5.0 Dec 2005, 6X coverage

Chicken, WASHUC2 May 2006; Zebra finch, Taeniopygia_guttata-3.2.4 Aug 2008, 58.1d; Duck, duck1 Aug 2009; Anole lizard, AnoCar2.0 May 2010; Xenopus, JGI 4.2 Nov 2009, 62.42

Medaka, HdrR Oct 2005; Stickleback, BROAD S1, Feb 2006; Zebrafish, Zv9 Apr 2010; Tetraodon, TETRAODON 8.0 Mar 2007; Elephant shark, whole genome shotgun sequences9; Sea lamprey, Petromyzon_marinus_7.0, Jan 2011

**(a) Complete sequences**

>hsaCXCR1 NP_000625.1 Chr 2

MSNITDPQMWDFDDLNFTGMPPADEDYSPCMLETETLNKYVVIIAYALVFLLSLLGNSLVMLVILYSRVGRSVTDVYLLNLALADLLFALTLPIWAASKVNGWIFGTFLCKVVSLLKEVNFYSGILLLACISVDRYLAIVHATRTLTQKRHLVKFVCLGCWGLSMNLSLPFFLFRQAYHPNNSSPVCYEVLGNDTAKWRMVLRILPHTFGFIVPLFVMLFCYGFTLRTLFKAHMGQKHRAMRVIFAVVLIFLLCWLPYNLVLLADTLMRTQVIQETCERRNNIGRALDATEILGFLHSCLNPIIYAFIGQNFRHGFLKILAMHGLVSKEFLARHRVTSYTSSSVNVSSNL

>hsaCXCR2 NP_001548.1 Chr 2

MEDFNMESDSFEDFWKGEDLSNYSYSSTLPPFLLDAAPCEPESLEINKYFVVIIYALVFLLSLLGNSLVMLVILYSRVGRSVTDVYLLNLALADLLFALTLPIWAASKVNGWIFGTFLCKVVSLLKEVNFYSGILLLACISVDRYLAIVHATRTLTQKRYLVKFICLSIWGLSLLLALPVLLFRRTVYSSNVSPACYEDMGNNTANWRMLLRILPQSFGFIVPLLIMLFCYGFTLRTLFKAHMGQKHRAMRVIFAVVLIFLLCWLPYNLVLLADTLMRTQVIQETCERRNHIDRALDATEILGILHSCLNPLIYAFIGQKFRHGLLKILAIHGLISKDSLPKDSRPSFVGSSSGHTSTTL

>hsaCXCR3 NP_001495.1 Chr X

MVLEVSDHQVLNDAEVAALLENFSSSYDYGENESDSCCTSPPCPQDFSLNFDRAFLPALYSLLFLLGLLGNGAVAAVLLSRRTALSSTDTFLLHLAVADTLLVLTLPLWAVDAAVQWVFGSGLCKVAGALFNINFYAGALLLACISFDRYLNIVHATQLYRRGPPARVTLTCLAVWGLCLLFALPDFIFLSAHHDERLNATHCQYNFPQVGRTALRVLQLVAGFLLPLLVMAYCYAHILAVLLVSRGQRRLRAMRLVVVVVVAFALCWTPYHLVVLVDILMDLGALARNCGRESRVDVAKSVTSGLGYMHCCLNPLLYAFVGVKFRERMWMLLLRLGCPNQRGLQRQPSSSRRDSSWSETSEASYSGL

>hsaCXCR4 NP_003458.1 Chr 2

MEGISIYTSDNYTEEMGSGDYDSMKEPCFREENANFNKIFLPTIYSIIFLTGIVGNGLVILVMGYQKKLRSMTDKYRLHLSVADLLFVITLPFWAVDAVANWYFGNFLCKAVHVIYTVNLYSSVLILAFISLDRYLAIVHATNSQRPRKLLAEKVVYVGVWIPALLLTIPDFIFANVSEADDRYICDRFYPNDLWVVVFQFQHIMVGLILPGIVILSCYCIIISKLSHSKGHQKRKALKTTVILILAFFACWLPYYIGISIDSFILLEIIKQGCEFENTVHKWISITEALAFFHCCLNPILYAFLGAKFKTSAQHALTSVSRGSSLKILSKGKRGGHSSVSTESESSSFHSS

>hsaCXCR5 NP_001707.1 Chr 11

MNYPLTLEMDLENLEDLFWELDRLDNYNDTSLVENHLCPATEGPLMASFKAVFVPVAYSLIFLLGVIGNVLVLVILERHRQTRSSTETFLFHLAVADLLLVFILPFAVAEGSVGWVLGTFLCKTVIALHKVNFYCSSLLLACIAVDRYLAIVHAVHAYRHRRLLSIHITCGTIWLVGFLLALPEILFAKVSQGHHNNSLPRCTFSQENQAETHAWFTSRFLYHVAGFLLPMLVMGWCYVGVVHRLRQAQRRPQRQKAVRVAILVTSIFFLCWSPYHIVIFLDTLARLKAVDNTCKLNGSLPVAITMCEFLGLAHCCLNPMLYTFAGVKFRSDLSRLLTKLGCTGPASLCQLFPSWRRSSLSESENATSLTTF

>hsaCXCR6 NP_006555.1 Chr 3

MAEHDYHEDYGFSSFNDSSQEEHQDFLQFSKVFLPCMYLVVFVCGLVGNSLVLVISIFYHKLQSLTDVFLVNLPLADLVFVCTLPFWAYAGIHEWVFGQVMCKSLLGIYTINFYTSMLILTCITVDRFIVVVKATKAYNQQAKRMTWGKVTSLLIWVISLLVSLPQIIYGNVFNLDKLICGYHDEAISTVVLATQMTLGFFLPLLTMIVCYSVIIKTLLHAGGFQKHRSLKIIFLVMAVFLLTQMPFNLMKFIRSTHWEYYAMTSFHYTIMVTEAIAYLRACLNPVLYAFVSLKFRKNFWKLVKDIGCLPYLGVSHQWKSSEDNSKTFSASHNVEATSMFQL

>hsaCCR1 NP_001286.1 Chr 3

METPNTTEDYDTTTEFDYGDATPCQKVNERAFGAQLLPPLYSLVFVIGLVGNILVVLVLVQYKRLKNMTSIYLLNLAISDLLFLFTLPFWIDYKLKDDWVFGDAMCKILSGFYYTGLYSEIFFIILLTIDRYLAIVHAVFALRARTVTFGVITSIIIWALAILASMPGLYFSKTQWEFTHHTCSLHFPHESLREWKLFQALKLNLFGLVLPLLVMIICYTGIIKILLRRPNEKKSKAVRLIFVIMIIFFLFWTPYNLTILISVFQDFLFTHECEQSRHLDLAVQVTEVIAYTHCCVNPVIYAFVGERFRKYLRQLFHRRVAVHLVKWLPFLSVDRLERVSSTSPSTGEHELSAGF

>hsaCCR2 NP_001116868.1 Chr 3

MLSTSRSRFIRNTNESGEEVTTFFDYDYGAPCHKFDVKQIGAQLLPPLYSLVFIFGFVGNMLVVLILINCKKLKCLTDIYLLNLAISDLLFLITLPLWAHSAANEWVFGNAMCKLFTGLYHIGYFGGIFFIILLTIDRYLAIVHAVFALKARTVTFGVVTSVITWLVAVFASVPGIIFTKCQKEDSVYVCGPYFPRGWNNFHTIMRNILGLVLPLLIMVICYSGILKTLLRCRNEKKRHRAVRVIFTIMIVYFLFWTPYNIVILLNTFQEFFGLSNCESTSQLDQATQVTETLGMTHCCINPIIYAFVGEKFRRYLSVFFRKHITKRFCKQCPVFYRETVDGVTSTNTPSTGEQEVSAGL

>hsaCCR3 NP_001828.1 Chr 3

MTTSLDTVETFGTTSYYDDVGLLCEKADTRALMAQFVPPLYSLVFTVGLLGNVVVVMILIKYRRLRIMTNIYLLNLAISDLLFLVTLPFWIHYVRGHNWVFGHGMCKLLSGFYHTGLYSEIFFIILLTIDRYLAIVHAVFALRARTVTFGVITSIVTWGLAVLAALPEFIFYETEELFEETLCSALYPEDTVYSWRHFHTLRMTIFCLVLPLLVMAICYTGIIKTLLRCPSKKKYKAIRLIFVIMAVFFIFWTPYNVAILLSSYQSILFGNDCERSKHLDLVMLVTEVIAYSHCCMNPVIYAFVGERFRKYLRHFFHRHLLMHLGRYIPFLPSEKLERTSSVSPSTAEPELSIVF

>hsaCCR4 NP_005499.1 Chr 3

MNPTDIADTTLDESIYSNYYLYESIPKPCTKEGIKAFGELFLPPLYSLVFVFGLLGNSVVVLVLFKYKRLRSMTDVYLLNLAISDLLFVFSLPFWGYYAADQWVFGLGLCKMISWMYLVGFYSGIFFVMLMSIDRYLAIVHAVFSLRARTLTYGVITSLATWSVAVFASLPGFLFSTCYTERNHTYCKTKYSLNSTTWKVLSSLEINILGLVIPLGIMLFCYSMIIRTLQHCKNEKKNKAVKMIFAVVVLFLGFWTPYNIVLFLETLVELEVLQDCTFERYLDYAIQATETLAFVHCCLNPIIYFFLGEKFRKYILQLFKTCRGLFVLCQYCGLLQIYSADTPSSSYTQSTMDHDLHDAL

>hsaCCR5 NP_000570.1 Chr 3

MDYQVSSPIYDINYYTSEPCQKINVKQIAARLLPPLYSLVFIFGFVGNMLVILILINCKRLKSMTDIYLLNLAISDLFFLLTVPFWAHYAAAQWDFGNTMCQLLTGLYFIGFFSGIFFIILLTIDRYLAVVHAVFALKARTVTFGVVTSVITWVVAVFASLPGIIFTRSQKEGLHYTCSSHFPYSQYQFWKNFQTLKIVILGLVLPLLVMVICYSGILKTLLRCRNEKKRHRAVRLIFTIMIVYFLFWAPYNIVLLLNTFQEFFGLNNCSSSNRLDQAMQVTETLGMTHCCINPIIYAFVGEKFRNYLLVFFQKHIAKRFCKCCSIFQQEAPERASSVYTRSTGEQEISVGL

>hsaCCR6 NP_004358.2 Chr 6

MSGESMNFSDVFDSSEDYFVSVNTSYYSVDSEMLLCSLQEVRQFSRLFVPIAYSLICVFGLLGNILVVITFAFYKKARSMTDVYLLNMAIADILFVLTLPFWAVSHATGAWVFSNATCKLLKGIYAINFNCGMLLLTCISMDRYIAIVQATKSFRLRSRTLPRSKIICLVVWGLSVIISSSTFVFNQKYNTQGSDVCEPKYQTVSEPIRWKLLMLGLELLFGFFIPLMFMIFCYTFIVKTLVQAQNSKRHKAIRVIIAVVLVFLACQIPHNMVLLVTAANLGKMNRSCQSEKLIGYTKTVTEVLAFLHCCLNPVLYAFIGQKFRNYFLKILKDLWCVRRKYKSSGFSCAGRYSENISRQTSETADNDNASSFTM

>hsaCCR7 NP_001829.1 Chr 17

MDLGKPMKSVLVVALLVIFQVCLCQDEVTDDYIGDNTTVDYTLFESLCSKKDVRNFKAWFLPIMYSIICFVGLLGNGLVVLTYIYFKRLKTMTDTYLLNLAVADILFLLTLPFWAYSAAKSWVFGVHFCKLIFAIYKMSFFSGMLLLLCISIDRYVAIVQAVSAHRHRARVLLISKLSCVGIWILATVLSIPELLYSDLQRSSSEQAMRCSLITEHVEAFITIQVAQMVIGFLVPLLAMSFCYLVIIRTLLQARNFERNKAIKVIIAVVVVFIVFQLPYNGVVLAQTVANFNITSSTCELSKQLNIAYDVTYSLACVRCCVNPFLYAFIGVKFRNDLFKLFKDLGCLSQEQLRQWSSCRHIRRSSMSVEAETTTTFSP

>hsaCCR8 NP_005192.1 Chr 3

MDYTLDLSVTTVTDYYYPDIFSSPCDAELIQTNGKLLLAVFYCLLFVFSLLGNSLVILVLVVCKKLRSITDVYLLNLALSDLLFVFSFPFQTYYLLDQWVFGTVMCKVVSGFYYIGFYSSMFFITLMSVDRYLAVVHAVYALKVRTIRMGTTLCLAVWLTAIMATIPLLVFYQVASEDGVLQCYSFYNQQTLKWKIFTNFKMNILGLLIPFTIFMFCYIKILHQLKRCQNHNKTKAIRLVLIVVIASLLFWVPFNVVLFLTSLHSMHILDGCSISQQLTYATHVTEIISFTHCCVNPVIYAFVGEKFKKHLSEIFQKSCSQIFNYLGRQMPRESCEKSSSCQQHSSRSSSVDYIL

>hsaCCR9 NP_112477.1 Chr 3

MTPTDFTSPIPNMADDYGSESTSSMEDYVNFNFTDFYCEKNNVRQFASHFLPPLYWLVFIVGALGNSLVILVYWYCTRVKTMTDMFLLNLAIADLLFLVTLPFWAIAAADQWKFQTFMCKVVNSMYKMNFYSCVLLIMCISVDRYIAIAQAMRAHTWREKRLLYSKMVCFTIWVLAAALCIPEILYSQIKEESGIAICTMVYPSDESTKLKSAVLTLKVILGFFLPFVVMACCYTIIIHTLIQAKKSSKHKALKVTITVLTVFVLSQFPYNCILLVQTIDAYAMFISNCAVSTNIDICFQVTQTIAFFHSCLNPVLYVFVGERFRRDLVKTLKNLGCISQAQWVSFTRREGSLKLSSMLLETTSGALSL

>hsaCCR10 NP_057686.2 Chr 17

MGTEATEQVSWGHYSGDEEDAYSAEPLPELCYKADVQAFSRAFQPSVSLTVAALGLAGNGLVLATHLAARRAARSPTSAHLLQLALADLLLALTLPFAAAGALQGWSLGSATCRTISGLYSASFHAGFLFLACISADRYVAIARALPAGPRPSTPGRAHLVSVIVWLLSLLLALPALLFSQDGQREGQRRCRLIFPEGLTQTVKGASAVAQVALGFALPLGVMVACYALLGRTLLAARGPERRRALRVVVALVAAFVVLQLPYSLALLLDTADLLAARERSCPASKRKDVALLVTSGLALARCGLNPVLYAFLGLRFRQDLRRLLRGGSSPSGPQPRRGCPRRPRLSSCSAPTETHSLSWDN

>hsaCX3CR1 NP_001328.1 Chr 3

MDQFPESVTENFEYDDLAEACYIGDIVVFGTVFLSIFYSVIFAIGLVGNLLVVFALTNSKKPKSVTDIYLLNLALSDLLFVATLPFWTHYLINEKGLHNAMCKFTTAFFFIGFFGSIFFITVISIDRYLAIVLAANSMNNRTVQHGVTISLGVWAAAILVAAPQFMFTKQKENECLGDYPEVLQEIWPVLRNVETNFLGFLLPLLIMSYCYFRIIQTLFSCKNHKKAKAIKLILLVVIVFFLFWTPYNVMIFLETLKLYDFFPSCDMRKDLRLALSVTETVAFSHCCLNPLIYAFAGEKFRRYLYHLYGKCLAVLCGRSVHVDFSSSESQRSRHGSVLSSNFTYHTSDGDALLLL

>hsaXCR1 NP_005274.1 Chr 3

MESSGNPESTTFFYYDLQSQPCENQAWVFATLATTVLYCLVFLLSLVGNSLVLWVLVKYESLESLTNIFILNLCLSDLVFACLLPVWISPYHWGWVLGDFLCKLLNMIFSISLYSSIFFLTIMTIHRYLSVVSPLSTLRVPTLRCRVLVTMAVWVASILSSILDTIFHKVLSSGCDYSELTWYLTSVYQHNLFFLLSLGIILFCYVEILRTLFRSRSKRRHRTVKLIFAIVVAYFLSWGPYNFTLFLQTLFRTQIIRSCEAKQQLEYALLICRNLAFSHCCFNPVLYVFVGVKFRTHLKHVLRQFWFCRLQAPSPASIPHSPGAFAYEGASFY

>hsaCXCR7 NP_064707.1 Chr 2

MDLHLFDYSEPGNFSDISWPCNSSDCIVVDTVMCPNMPNKSVLLYTLSFIYIFIFVIGMIANSVVVWVNIQAKTTGYDTHCYILNLAIADLWVVLTIPVWVVSLVQHNQWPMGELTCKVTHLIFSINLFGSIFFLTCMSVDRYLSITYFTNTPSSRKKMVRRVVCILVWLLAFCVSLPDTYYLKTVTSASNNETYCRSFYPEHSIKEWLIGMELVSVVLGFAVPFSIIAVFYFLLARAISASSDQEKHSSRKIIFSYVVVFLVCWLPYHVAVLLDIFSILHYIPFTCRLEHALFTALHVTQCLSLVHCCVNPVLYSFINRNYRYELMKAFIFKYSAKTGLTKLIDASRVSETEYSALEQSTK

>hsaCCBP2 NP_001287.2 Chr 3

MAATASPQPLATEDADSENSSFYYYDYLDEVAFMLCRKDAVVSFGKVFLPVFYSLIFVLGLSGNLLLLMVLLRYVPRRRMVEIYLLNLAISNLLFLVTLPFWGISVAWHWVFGSFLCKMVSTLYTINFYSGIFFISCMSLDKYLEIVHAQPYHRLRTRAKSLLLATIVWAVSLAVSIPDMVFVQTHENPKGVWNCHADFGGHGTIWKLFLRFQQNLLGFLLPLLAMIFFYSRIGCVLVRLRPAGQGRALKIAAALVVAFFVLWFPYNLTLFLHTLLDLQVFGNCEVSQHLDYALQVTESIAFLHCCFSPILYAFSSHRFRQYLKAFLAAVLGWHLAPGTAQASLSSCSESSILTAQEEMTGMNDLGERQSENYPNKEDVGNKSA

>hsaCCRL1 NP_057641.1 Chr 3

MALEQNQSTDYYYEENEMNGTYDYSQYELICIKEDVREFAKVFLPVFLTIVFVIGLAGNSMVVAIYAYYKKQRTKTDVYILNLAVADLLLLFTLPFWAVNAVHGWVLGKIMCKITSALYTLNFVSGMQFLACISIDRYVAVTKVPSQSGVGKPCWIICFCVWMAAILLSIPQLVFYTVNDNARCIPIFPRYLGTSMKALIQMLEICIGFVVPFLIMGVCYFITARTLMKMPNIKISRPLKVLLTVVIVFIVTQLPYNIVKFCRAIDIIYSLITSCNMSKRMDIAIQVTESIALFHSCLNPILYVFMGASFKNYVMKVAKKYGSWRRQRQSVEEFPFDSEGPTEPTSTFSI

>hsaCCRL2 NP_003956.2 Chr 3

MANYTLAPEDEYDVLIEGELESDEAEQCDKYDAQALSAQLVPSLCSAVFVIGVLDNLLVVLILVKYKGLKRVENIYLLNLAVSNLCFLLTLPFWAHAGGDPMCKILIGLYFVGLYSETFFNCLLTVQRYLVFLHKGNFFSARRRVPCGIITSVLAWVTAILATLPEYVVYKPQMEDQKYKCAFSRTPFLPADETFWKHFLTLKMNISVLVLPLFIFTFLYVQMRKTLRFREQRYSLFKLVFAIMVVFLLMWAPYNIAFFLSTFKEHFSLSDCKSSYNLDKSVHITKLIATTHCCINPLLYAFLDGTFSKYLCRCFHLRSNTPLQPRGQSAQGTSREEPDHSTEV

>hsaDARC NP_001116423.1 Chr 1

MASSGYVLQAELSPSTENSSQLDFEDVWNSSYGVNDSFPDGDYGANLEAAAPCHSCNLLDDSALPFFILTSVLGILASSTVLFMLFRPLFRWQLCPGWPVLAQLAVGSALFSIVVPVLAPGLGSTRSSALCSLGYCVWYGSAFAQALLLGCHASLGHRLGAGQVPGLTLGLTVGIWGVAALLTLPVTLASGASGGLCTLIYSTELKALQATHTVACLAIFVLLPLGLFGAKGLKKALGMGPGPWMNILWAWFIFWWPHGVVLGLDFLVRSKLLLLSTCLAQQALDLLLNLAEALAILHCVATPLLLALFCHQATRTLLPSLPLPEGWSSHLDTLGSKS

>musCXCR1 NP_839972.1 Chr 1

MAEAEYFIWTNPEGDFEKEFGNITGMLPTGDYFIPCKRVPITNRQALVVFYALVSLLSLLGNSLVMLVILYRRRTRSVMDVYVLNLAIADLLFSLTLPFLAVSKLKGWIFGTPLCKMVSLLKEFNFFSGILLLACISVDRYLAIVHATRTLARKRYLVKFVCVGIWGLSLILSLPFAIFRQAYKPFRSGTVCYEVLGEATTDFRMTLRGLSHIFGFLLPLLTMLVCYGLTLRMLFKTHMRQKHRAMGVIFAVVLVFLLCCLPYNLVLLSDTLLGAHLIEDTCERRNDIDQALYITEILGFSHSCLNPIIYAFVGQNFRHEFLKILANHGLVRKEVLTHRRVAFHTSLTAIY

>musCXCR2 NP_034039.1 Chr 1

MGEFKVDKFNIEDFFSGDLDIFNYSSGMPSILPDAVPCHSENLEINSYAVVVIYVLVTLLSLVGNSLVMLVILYNRSTCSVTDVYLLNLAIADLFFALTLPVWAASKVNGWTFGSTLCKIFSYVKEVTFYSSVLLLACISMDRYLAIVHATSTLIQKRHLVKFVCIAMWLLSVILALPILILRNPVKVNLSTLVCYEDVGNNTSRLRVVLRILPQTFGFLVPLLIMLFCYGFTLRTLFKAHMGQKHRAMRVIFAVVLVFLLCWLPYNLVLFTDTLMRTKLIKETCERRDDIDKALNATEILGFLHSCLNPIIYAFIGQKFRHGLLKIMATYGLVSKEFLAKEGRPSFVSSSSANTSTTL

>musCXCR3 NP_034040.1 Chr X

MYLEVSERQVLDASDFAFLLENSTSPYDYGENESDFSDSPPCPQDFSLNFDRTFLPALYSLLFLLGLLGNGAVAAVLLSQRTALSSTDTFLLHLAVADVLLVLTLPLWAVDAAVQWVFGPGLCKVAGALFNINFYAGAFLLACISFDRYLSIVHATQIYRRDPRVRVALTCIVVWGLCLLFALPDFIYLSANYDQRLNATHCQYNFPQVGRTALRVLQLVAGFLLPLLVMAYCYAHILAVLLVSRGQRRFRAMRLVVVVVAAFAVCWTPYHLVVLVDILMDVGVLARNCGRESHVDVAKSVTSGMGYMHCCLNPLLYAFVGVKFREQMWMLFTRLGRSDQRGPQRQPSSSRRESSWSETTEASYLGL

>musCXCR4 NP_034041.2 Chr 1

MEPISVSIYTSDNYSEEVGSGDYDSNKEPCFRDENVHFNRIFLPTIYFIIFLTGIVGNGLVILVMGYQKKLRSMTDKYRLHLSVADLLFVITLPFWAVDAMADWYFGKFLCKAVHIIYTVNLYSSVLILAFISLDRYLAIVHATNSQRPRKLLAEKAVYVGVWIPALLLTIPDFIFADVSQGDISQGDDRYICDRLYPDSLWMVVFQFQHIMVGLVLPGIVILSCYCIIISKLSHSKGHQKRKALKTTVILILAFFACWLPYYVGISIDSFILLGVIKQGCDFESIVHKWISITEALAFFHCCLNPILYAFLGAKFKSSAQHALNSMSRGSSLKILSKGKRGGHSSVSTESESSSFHSS

>musCXCR5 NP_031577.2 Chr 9

MNYPLTLDMGSITYNMDDLYKELAFYSNSTEIPLQDSNFCSTVEGPLLTSFKAVFMPVAYSLIFLLGMMGNILVLVILERHRHTRSSTETFLFHLAVADLLLVFILPFAVAEGSVGWVLGTFLCKTVIALHKINFYCSSLLLACIAVDRYLAIVHAVHAYRRRRLLSIHITCTAIWLAGFLFALPELLFAKVGQPHNNDSLPQCTFSQENEAETRAWFTSRFLYHIGGFLLPMLVMGWCYVGVVHRLLQAQRRPQRQKAVRVAILVTSIFFLCWSPYHIVIFLDTLERLKAVNSSCELSGYLSVAITLCEFLGLAHCCLNPMLYTFAGVKFRSDLSRLLTKLGCAGPASLCQLFPNWRKSSLSESENATSLTTF

>musCXCR6 NP_109637.3 Chr 9

MDDGHQESALYDGHYEGDFWLFNNSSDNSQENKRFLKFKEVFLPCVYLVVFVFGLLGNSLVLIIYIFYQKLRTLTDVFLLNLPLADLVFVCTLPFWAYAGTYEWVFGTVMCKTLRGMYTMNFYVSMLTLTCITVDRFIVVVQATKAFNRQAKWKIWGQVICLLIWVVSLLVSLPQIIYGHVQDIDKLICQYHSEEISTMVLVIQMTLGFFLPLLTMILCYSGIIKTLLHARNFQKHKSLKIIFLVVAVFLLTQTPFNLAMLIQSTSWEYYTITSFKYAIVVTEAIAYFRACLNPVLYAFVGLKFRKNVWKLMKDIGCLSHLGVSSQWKSSEDSSKTCSASHNVETTSMFQL

>musCCR1 NP_034042.3 Chr 9

MEISDFTEAYPTTTEFDYGDSTPCQKTAVRAFGAGLLPPLYSLVFIIGVVGNVLVILVLMQHRRLQSMTSIYLFNLAVSDLVFLFTLPFWIDYKLKDDWIFGDAMCKLLSGFYYLGLYSEIFFIILLTIDRYLAIVHAVFALRARTVTFGIITSIITWALAILASMPALYFFKAQWEFTHRTCSPHFPYKSLKQWKRFQALKLNLLGLILPLLVMIICYAGIIRILLRRPSEKKVKAVRLIFAITLLFFLLWTPYNLSVFVSAFQDVLFTNQCEQSKQLDLAMQVTEVIAYTHCCVNPIIYVFVGERFWKYLRQLFQRHVAIPLAKWLPFLSVDQLERTSSISPSTGEHELSAGF

>musCCR1L1 NP_031744.3 Chr 9

MEIPAVTEPSYNTVAKNDFMSGFLCFSINVRAFGITVPTPLYSLVFIIGVIGHVLVVLVLIQHKRLRNMTSIYLFNLAISDLVFLSTLPFWVDYIMKGDWIFGNAMCKFVSGFYYLGLYSDMFFITLLTIDRYLAVVHVVFALRARTVTFGIISSIITWVLAALVSIPCLYVFKSQMEFTYHTCRAILPRKSLIRFLRFQALTMNILGLILPLLAMIICYTRIINVLHRRPNKKKAKVMRLIFVITLLFFLLLAPYYLAAFVSAFEDVLFTPSCLRSQQVDLSLMITEALAYTHCCVNPVIYVFVGKRFRKYLWQLFRRHTAITLPQWLPFLSEDRAQRASATPPSTVEIETSADL

>musCCR2 NP_034045.1 Chr 9

MEDNNMLPQFIHGILSTSHSLFTRSIQELDEGATTPYDYDDGEPCHKTSVKQIGAWILPPLYSLVFIFGFVGNMLVIIILIGCKKLKSMTDIYLLNLAISDLLFLLTLPFWAHYAANEWVFGNIMCKVFTGLYHIGYFGGIFFIILLTIDRYLAIVHAVFALKARTVTFGVITSVVTWVVAVFASLPGIIFTKSKQDDHHYTCGPYFTQLWKNFQTIMRNILSLILPLLVMVICYSGILHTLFRCRNEKKRHRAVRLIFAIMIVYFLFWTPYNIVLFLTTFQESLGMSNCVIDKHLDQAMQVTETLGMTHCCINPVIYAFVGEKFRRYLSIFFRKHIAKRLCKQCPVFYRETADRVSSTFTPSTGEQEVSVGL

>musCCR3 NP_034044.3 Chr 9

MAFNTDEIKTVVESFETTPYEYEWAPPCEKVRIKELGSWLLPPLYSLVFIIGLLGNMMVVLILIKYRKLQIMTNIYLFNLAISDLLFLFTVPFWIHYVLWNEWGFGHYMCKMLSGFYYLALYSEIFFIILLTIDRYLAIVHAVFALRARTVTFATITSIITWGLAGLAALPEFIFHESQDSFGEFSCSPRYPEGEEDSWKRFHALRMNIFGLALPLLIMVICYSGIIKTLLRCPNKKKHKAIRLIFVVMIVFFIFWTPYNLVLLFSAFHSTFLETSCQQSKHLDLAMQVTEVIAYTHCCINPVIYAFVGERFRKHLRLFFHRNVAVYLGKYIPFLPGEKMERTSSVSPSTGEQEISVVF

>musCCR4 NP_034046.2 Chr 9

MNATEVTDTTQDETVYNSYYFYESMPKPCTKEGIKAFGEVFLPPLYSLVFLLGLFGNSVVVLVLFKYKRLKSMTDVYLLNLAISDLLFVLSLPFWGYYAADQWVFGLGLCKIVSWMYLVGFYSGIFFIMLMSIDRYLAIVHAVFSLKARTLTYGVITSLITWSVAVFASLPGLLFSTCYTEHNHTYCKTQYSVNSTTWKVLSSLEINVLGLLIPLGIMLFCYSMIIRTLQHCKNEKKNRAVRMIFAVVVLFLGFWTPYNVVLFLETLVELEVLQDCTLERYLDYAIQATETLAFIHCCLNPVIYFFLGEKFRKYITQLFRTCRGPLVLCKHCDFLQVYSADMSSSSYTQSTVDHDFRDAL

>musCCR5 NP_034047.2 Chr 9

MDFQGSVPTYSYDIDYGMSAPCQKINVKQIAAQLLPPLYSLVFIFGFVGNMMVFLILISCKKLKSVTDIYLLNLAISDLLFLLTLPFWAHYAANEWVFGNIMCKVFTGLYHIGYFGGIFFIILLTIDRYLAIVHAVFALKVRTVNFGVITSVVTWAVAVFASLPEIIFTRSQKEGFHYTCSPHFPHTQYHFWKSFQTLKMVILSLILPLLVMVICYSGILHTLFRCRNEKKRHRAVRLIFAIMIVYFLFWTPYNIVLLLTTFQEFFGLNNCSSSNRLDQAMQATETLGMTHCCLNPVIYAFVGEKFRSYLSVFFRKHMVKRFCKRCSIFQQDNPDRASSVYTRSTGEHEVSTGL

>musCCR6 NP_033965.1 Chr 17

MNSTESYFGTDDYDNTEYYSIPPDHGPCSLEEVRNFTKVFVPIAYSLICVFGLLGNIMVVMTFAFYKKARSMTDVYLLNMAITDILFVLTLPFWAVTHATNTWVFSDALCKLMKGTYAVNFNCGMLLLACISMDRYIAIVQATKSFRVRSRTLTHSKVICVAVWFISIIISSPTFIFNKKYELQDRDVCEPRYRSVSEPITWKLLGMGLELFFGFFTPLLFMVFCYLFIIKTLVQAQNSKRHRAIRVVIAVVLVFLACQIPHNMVLLVTAVNTGKVGRSCSTEKVLAYTRNVAEVLAFLHCCLNPVLYAFIGQKFRNYFMKIMKDVWCMRRKNKMPGFLCARVYSESYISRQTSETVENDNASSFTM

>musCCR7 NP_031745.2 Chr 11

MDPGKPRKNVLVVALLVIFQVCFCQDEVTDDYIGENTTVDYTLYESVCFKKDVRNFKAWFLPLMYSVICFVGLLGNGLVILTYIYFKRLKTMTDTYLLNLAVADILFLLILPFWAYSEAKSWIFGVYLCKGIFGIYKLSFFSGMLLLLCISIDRYVAIVQAVSAHRHRARVLLISKLSCVGIWMLALFLSIPELLYSGLQKNSGEDTLRCSLVSAQVEALITIQVAQMVFGFLVPMLAMSFCYLIIIRTLLQARNFERNKAIKVIIAVVVVFIVFQLPYNGVVLAQTVANFNITNSSCETSKQLNIAYDVTYSLASVRCCVNPFLYAFIGVKFRSDLFKLFKDLGCLSQERLRHWSSCRHVRNASVSMEAETTTTFSP

>musCCR8 NP_031746.1 Chr 9

MDYTMEPNVTMTDYYPDFFTAPCDAEFLLRGSMLYLAILYCVLFVLGLLGNSLVILVLVGCKKLRSITDIYLLNLAASDLLFVLSIPFQTHNLLDQWVFGTAMCKVVSGLYYIGFFSSMFFITLMSVDRYLAIVHAVYAIKVRTASVGTALSLTVWLAAVTATIPLMVFYQVASEDGMLQCFQFYEEQSLRWKLFTHFEINALGLLLPFAILLFCYVRILQQLRGCLNHNRTRAIKLVLTVVIVSLLFWVPFNVALFLTSLHDLHILDGCATRQRLALAIHVTEVISFTHCCVNPVIYAFIGEKFKKHLMDVFQKSCSHIFLYLGRQMPVGALERQLSSNQRSSHSSTLDDIL

>musCCR9 NP_034043.1 Chr 9

MMPTELTSLIPGMFDDFSYDSTASTDDYMNLNFSSFFCKKNNVRQFASHFLPPLYWLVFIVGTLGNSLVILVYWYCTRVKTMTDMFLLNLAIADLLFLATLPFWAIAAAGQWMFQTFMCKVVNSMYKMNFYSCVLLIMCISVDRYIAIVQAMKAQVWRQKRLLYSKMVCITIWVMAAVLCTPEILYSQVSGESGIATCTMVYPKDKNAKLKSAVLILKVTLGFFLPFMVMAFCYTIIIHTLVQAKKSSKHKALKVTITVLTVFIMSQFPYNSILVVQAVDAYAMFISNCTISTNIDICFQVTQTIAFFHSCLNPVLYVFVGERFRRDLVKTLKNLGCISQAQWVSFTRREGSLKLSSMLLETTSGALSL

>musCCR10 NP_031747.2 Chr 11

MGTKPTEQVSWGLYSGYDEEAYSVGPLPELCYKADVQAFSRAFQPSVSLMVAVLGLAGNGLVLATHLAARRTTRSPTSVHLLQLALADLLLALTLPFAAAGALQGWNLGSTTCRAISGLYSASFHAGFLFLACISADRYVAIARALPAGQRPSTPSRAHLVSVFVWLLSLFLALPALLFSRDGPREGQRRCRLIFPESLTQTVKGASAVAQVVLGFALPLGVMAACYALLGRTLLAARGPERRRALRVVVALVVAFVVLQLPYSLALLLDTADLLAARERSCSSSKRKDLALLVTGGLTLVRCSLNPVLYAFLGLRFRRDLRRLLQGGGCSPKPNPRGRCPRRLRLSSCSAPTETHSLSWDN

>musCX3CR1 NP_034117.3 Chr 9

MSTSFPELDLENFEYDDSAEACYLGDIVAFGTIFLSVFYALVFTFGLVGNLLVVLALTNSRKPKSITDIYLLNLALSDLLFVATLPFWTHYLISHEGLHNAMCKLTTAFFFIGFFGGIFFITVISIDRYLAIVLAANSMNNRTVQHGVTISLGVWAAAILVASPQFMFTKRKDNECLGDYPEVLQEMWPVLRNSEVNILGFALPLLIMSFCYFRIIQTLFSCKNRKKARAVRLILLVVFAFFLFWTPYNIMIFLETLKFYNFFPSCDMKRDLRLALSVTETVAFSHCCLNPFIYAFAGEKFRRYLGHLYRKCLAVLCGHPVHTGFSPESQRSRQDSILSSFTHYTSEGDGSLLL

>musXCR1 NP_035928.1 Chr 9

MESSTAFYDYHDKLSLLCENNVIFFSTISTIVLYSLVFLLSLVGNSLVLWVLVKYENLESLTNIFILNLCLSDLMFSCLLPVLISAQWSWFLGDFFCKFFNMIFGISLYSSIFFLTIMTIHRYLSVVSPISTLGIHTLRCRVLVTSCVWAASILFSIPDAVFHKVISLNCKYSEHHGFLASVYQHNIFFLLSMGIILFCYVQILRTLFRTRSRQRHRTVRLIFTVVVAYFLSWAPYNLTLFLKTGIIQQSCESLQQLDIAMIICRHLAFSHCCFNPVLYVFVGIKFRRHLKHLFQQVWLCRKTSSTVPCSPGTFTYEGPSFY

>musCXCR7 NP_031748.2 Chr 1

MDVHLFDYAEPGNYSDINWPCNSSDCIVVDTVQCPTMPNKNVLLYTLSFIYIFIFVIGMIANSVVVWVNIQAKTTGYDTHCYILNLAIADLWVVITIPVWVVSLVQHNQWPMGELTCKITHLIFSINLFGSIFFLACMSVDRYLSITYFTGTSSYKKKMVRRVVCILVWLLAFFVSLPDTYYLKTVTSASNNETYCRSFYPEHSIKEWLIGMELVSVILGFAVPFTIIAIFYFLLARAMSASGDQEKHSSRKIIFSYVVVFLVCWLPYHFVVLLDIFSILHYIPFTCQLENVLFTALHVTQCLSLVHCCVNPVLYSFINRNYRYELMKAFIFKYSAKTGLTKLIDASRVSETEYSALEQNTK

>musCCBP2 NP_067622.2 Chr 9

MPTVASPLPLTTVGSENSSSIYDYDYLDDMTILVCRKDEVLSFGRVFLPVVYSLIFVLGLAGNLLLLVVLLHSAPRRRTMELYLLNLAVSNLLFVVTMPFWAISVAWHWVFGSFLCKVISTLYSINFYCGIFFITCMSLDKYLEIVHAQPLHRPKAQFRNLLLIVMVWITSLAISVPEMVFVQIHQTLDGVWHCYADFGGHATIWKLYLRFQLNLLGFLLPLLAMIFFYSRIGCVLVRLRPPGQGRALRMAAALVIVFFMLWFPYNLTLFLHSLLDLHVFGNCEISHRLDYTLQVTESLAFSHCCFTPVLYAFCSHRFRRYLKAFLSVMLRWHQAPGTPSSNHSESSRVTAQEDVVSMNDLGERQSEDSLNKGEMGNT

>musCCRL1 NP_663746.2 Chr 9

MALELNQSAEYYYEENEMNYTHDYSQYEVICIKEEVRQFAKVFLPAFFTVAFVTGLAGNSVVVAIYAYYKKQRTKTDVYILNLAVADLLLLITLPFWAVNAVHGWILGKMMCKVTSALYTVNFVSGMQFLACISIDRYWAITKAPSQSGAGRPCWIICCCVWMAAILLSIPQLVFYTVNQNARCTPIFPHHLGTSLKASIQMLEIGIGFVVPFLIMGVCYASTARALIKMPNIKKSRPLRVLLAVVVVFIVTQLPYNVVKFCQAIDAIYLLITSCDMSKRMDVAIQVTESIALFHSCLNPILYVFMGASFKNYIMKVAKKYGSWRRQRQNVEEIPFDSEGPTEPTSSFTI

>musCCRL2 NP_059494.2 Chr 9

MDNYTVAPDDEYDVLILDDYLDNSGPDQVPAPEFLSPQQVLQFCCAVFAVGLLDNVLAVFILVKYKGLKNLGNIYFLNLALSNLCFLLPLPFWAHTAAHGESPGNGTCKVLVGLHSSGLYSEVFSNILLLVQGYRVFSQGRLASIFTTVSCGIVACILAWAMATALSLPESVFYEPRMERQKHKCAFGKPHFLPIEAPLWKYVLTSKMIILVLAFPLLVFIICCRQLRRRQSFRERQYDLHKPALVITGVFLLMWAPYNTVLFLSAFQEHLSLQDEKSSYHLDASVQVTQLVATTHCCVNPLLYLLLDRKAFMRYLRSLFPRCNDIPYQSSGGYQQAPPREGHGRPIELYSNLHQRQDII

>musDARC NP_034175.2 Chr 1

MGNCLYPVENLSLDKNGTQFTFDSWNYSFEDNYSYELSSDYSLTPAAPCYSCNLLGRSSLPFFMLTSVLGMLASGGILFAILRPFFHWQICPSWPILAELAVGSALFSIAVPILAPGLHSAHSTALCNLGYWVWYTSAFAQALLIGCYACLNPRLNIGQLRGFTLGLSVGLWGAAALLGLPVALASDAYNGFCAFPSSRDMEALKYMHYAICFTIFTVLPPTLLAAKGLKIALSKGPGPWVSVLWIWFIFWWPHGMVLIFDALVRSKIVLLYTCQSQKILDAMLNVTEALSMLHCVATPLLLALFCHQTTRRSLSSLSLPTRQASQMDALAGKS

>btaCXCR1 NP_776785.1 Chr 2

MTIILKDLSNSSILWEGFEDEFGNYSGTPPTEDYDYSPCEISTETLNKYAVVVIDALVFLLSLLGNSLVMLVILYSRIGRSVTDVYLLNLAMADLLFAMTLPIWTASKAKGWVFGTPLCKVVSLLKEVNFYSGILLLACISMDRYLAIVHATRTLTQKWHWVKFICLGIWALSVILALPIFIFREAYQPPYSDLVCYEDLGANTTKWRMIMRVLPQTFGFLLPLLVMLFCYGFTLRTLFSAQMGHKHRAMRVIFAVVLVFLLCWLPYNLVLIADTLMRAHVIAETCQRRNDIGRALDATEILGFLHSCLNPLIYVFIGQKFRHGLLKIMAIHGLISKEFLAKDGRPSFVGSSSGNTSTTL

>btaCXCR2 NP_001094755.1 Chr 2

MAETKFTSNIEGFNWENYSDEDFGNYSYNTDLPSILTDSAPCRPEILDINKHAVVVIYALVFLLSLLGNSLVMLVILYSRIGRSVTDVYLLNLAMADLLFAMTLPIWAASKAKGWVFGTPLCKVVSLLKEVNFYSGILLLACISMDRYLAIVHATRTLTQKRHWVKFICLGIWALSVILALPVFIFRRAIHPPYSSAVCYEDMGANTTKWRMVMRVLPQTFGFLLPLLVMLFCYGFTLRTLFSAQMGQKHRAMRVIFAVVLVFLLCWLPYNLVLIVDTLMRAHVIAETCQRRNDIGRALDATEILGFLHSCLNPLIYVFIGQKFRHGLLKIMAIHGLISKEFLAKDGRPSFVGSSSGNTSTTL

>btaCXCR3 NP_001011673.1 Chr X

MVPEMSERQEFQASDFAYLLENSSYDYGENETYFCCTSPPCPQDFSLNFDRTFLPVLYSLLFVLGLLGNGIVAVVLLSQRAALSSTDTFLLHLAVADALLVLTLPLWAVDAAIQWVFGSGLCKVAGALFNINFYAGALLLACISFDRYLSIVHATQLYRRGPPTRVALTCVAVWGLCLLFALPDFIFLSSHHDNRLNATHCQYNFPQEGHTALRILQLVAGFLLPLLVMAYCYARILAVLLVSRGQRRLRAMRLVVVVVVAFALCWTPYHLVVLVDTLMDLGALARNCGRESSVDIAKSVTSGMGYMHCCLNPLLYAFVGVKFRERMWVLLVRLGCPDQRCHQRQPSASRRESSWSETTEASYSGL

>btaCXCR4 NP_776726.1 Chr 2

MEGIRIFTSDNYTEDDLGSGDYDSMKEPCFREENAHFNRIFLPTVYSIIFLTGIVGNGLVILVMGYQKKLRSMTDKYRLHLSVADLLFVLTLPFWAVDAVANWYFGKFLCKAVHVIYTVNLYSSVLILAFISLDRYLAIVHATNSQKPRKLLAEKVVYVGVWLPAVLLTIPDLIFADIKEVDERYICDRFYPSDLWLVVFQFQHIVVGLLLPGIVILSCYCIIISKLSHSKGYQKRKALKTTVILILTFFACWLPYYIGISIDSFILLEIIQQGCEFESTVHKWISITEALAFFHCCLNPILYAFLGAKFKTSAQHALTSVSRGSSLKILSKGKRGGHSSVSTESESSSFHSS

>btaCXCR5 NP_001011675.1 Chr 15

MNYPLTLDMDLMNYNLEDLYKELGSYNDSTETPIKENHLCLTVEGPLLTSFKAVFMPVAYGLIFLLGVMGNILVLVILERHRQTRSSTETFLFHLAVADLLLVFILPFAVAESSVGWVLGTFLCKTVISLHKINFYCSSLLLACIAVDRYLAIVHAVHAYRHRRLLSIHITCATIWLAGFFFALPEILFAKVSEPHYNDSLPHCTFSQENQAETNAWFTSRFLYHIGGFLLPMLVMAWCYVGVVHRLCQAQRRPQRQKAVRVAILVTSVFFLCWSPYQVVIFLDTLARLKMLGSSCELDGYLSMAITMSEFLGLAHCCLNPMLYTFAGVKFRSYLSRLLTKLGCPGPASLCQFFPSWRKSSLSESENATSLTTF

>btaCXCR6 NP_001014859.1 Chr 22

MAEYNYEDLGFFNGSNDSSQGHQDFLRFSKFFLPCMYVVVFTCGLVGNSLVLVIYVFYQKLKSLTDVFLMNLPLADLVFVCTLPFWAYAGIHEWVFGNVMCKALLGIYTLNFYTSMLVLTCITVDRFVAVVRATKAYNQQAKRMAWGKAICSSIWVVSLLVSLPQIIYGNVLYHDKPFCGYHEAISTMVLAIQMTLGFFLPLLAMILCYSVIIKTLLQARGFRKHKSLKIIFLVVAVFLLTQTPFNLVKLIRSTSWEYHTMTSFDYAITVTEAIAYLRACLNPVLYAFVGLKFRKNFWKLVKDAGCLPYLGVSGQHMYSEDTSRSASASHNVEATSMFHL

>btaCCR1 NP_001071307.1 Chr 22

METSTTAKDYDMTTEYDYGDTTPCQKAQERAFGAQLLPPLYSVVFVIGLVGNILVVLVLMQYKRLKSMTSIYLLNLAISDLIFLFTLPFWIDYKVKDDWIFGDAMCKLLSGFYFMGLYSEIFFIILLTIDRYLAIVHAVFALRARTITFGIITSIVVWVLAVLASVPGLYFSKTQWEFTHHTCSIHFPPESFTKWKQFQALKLNIMGLVLPLLVMIVCYTGIIKILLRRPNEKKAKAVRLIFVIMIIFFLFWTPYNLSVFVAAFQDSLFTRKCEQSRQLDLAIQVTEVIAYTHCCINPVIYVFVGERFRKYLRQLFYRLVAVHLAKWFPFLSTERLERVSSMSPSTGEHELSAGF

>btaCCR1L1 NP_001069389.1 Chr 22

METSTTAKDYDMTTEYDYGDTTPCQKAQERAFGAQLLPLLYSVVFVIGLVGNILVVLVLYKRIKNIINICLLNLAISDLIFLFTLPFWIDYKVKDDWIFGDAMCKLLFGFFFLGLYSKIFFIILLTIDRYLTNVHPQFKRQCWNITSGTVTSIVVWVLAILASVPGLYFSKTQWWLTHYTCSLHFPPESHGRKWKQFLALKLNILGLILPLLVMTVCYGHIKIIKIMLRTWNKKANIIRLIFVIMITFFLFWTPCNLTVFVSAFQDSLCEHCSQLDLAIEVTEAIAYAHCCMNPIVYVLFCRRYRRVLWYLLYQLTKCRCFLYEEAGEGQLHVHYTGKEERCSVHRLRPLRSIRDKETISC

>btaCCR2 XP_584158.2 Chr 22

MDGNDTFSHNVLPTSHSLFTTNVKGNDEEPTTSYDYDYSEPCRKTSVGQIEAQLLPPLYSLVFIFGFVGNLLVVLILINCKKLKSMTDIYLLNLAISDLLFLLTMPFWAHYAADQWVFGNVMCKFFTGLYHIGYFGGIFFIILLTIDRYLAIVHAVFALKARTVTFGVVTSGVTWVVAVFASLPGIIFIKSLEEHSGYACAPYFPLGWKNFHTIMRSILGLVLPLLVMIICYSGIIKTLLRCRNEKKKHKAVRLIFVIMIVYFLFWAPYNIVLLLSTFQEFFGLSNCKSSSQLDQAMQVTETLGLTHCCINPIIYAFVGEKFRRYLSTFFRKHIAKHLCKQCPVFYGETGDRVSSTYTHSTGEQEVSAAL

>btaCCR3 XP_874241.1 Chr 22

MATSADGIETVGEVAGTTPYDYEAALPCEKSNVKELAAQFLPPLYSLVFVTGLLGNVVVVVILTKYKRLRIMTNIYLLNLAISDVLFLFTLPFWIHYVRWNEWVFGHRMCKLLSGLYYMGLYSEIFFIILLTIDRYLAIVHAVFALRARTVTFGIVTSIFTWVLAGLAALPEFFFHETQEEAGQTFCSPLYPEDNENAWKRFHALRMNILGLALPLLVMAICYSGIIKTLLRCPSKKKYKAIRLIFVIMVVFFIFWTPYNLVVLLFAFQMHLKADCEQSRQLDLAMLVTEVIAYTHCCVNPVIYAFVGERFRKHLRHFFHRYMATYLGKFMPFLPSEKLERTSSVSPSTGEQELSAVF

>btaCCR4 NP_001093763.1 Chr 22

MNPTDIADTTVDESIYNSYYLYENLPKPCNKDGIRAFGGLFLPPLYSLVFLFGLLGNSVVVLVLFKYKRLKSMTDVYLLNLAISDLLFVLSLPFWGYYAADQWVFGLGLCKLISWIYLVGFYSGIFFITLMSIDRYLAIVHAIFSLRARTLTYGVITSVATWSVAVLVSLPGLLFSTCYTERNHTYCKTKYSFNSTRWKVLSSLEINILGLVIPLGIMLFCYSMIIRTLQHCKNEKKNKAVKMIFAVVVLFLGFWTPYNVVLFLETLVELEVLQDCTFERHLDYAIQTTETLAFVHCCLNPVIYFFLGEKFRKYIVQLFKTCRGTLVLCQYCRLLPMYTDTPSSSYTQSTVDHDLRDAL

>btaCCR5 NP_001011672.2 Chr 22

MDYQTSTPLYDIDYGMSEPCQKLNVRQIAARLLPPLYSLVFIFGFVGNMLVVLILINCKKLKSMTDIYLLNLAISDLLFIITIPFWAHYAADQWVFGNTMCQLFTGFYFIGYFGGIFFIILLTIDRYLAIVHAVFALKARTVTFGAATSVVTWVVAVFASLPGIIFTKSQKEGSRHTCSPHFPSSQYHFWKNFQTLKIVILGLVLPLLVMIVCYSGIIKTLLRCRNEKKKHKAVRLIFVIMIVYFLFWAPYNIVLLLSTFQEFFGLNNCSGSNRLDQAMQVTETLGMTHCCINPIIYAFVGEKFRNYLLRFFRKYFASRFCKGCPVFQGEAPERVSSVYTRSTGEQEISVGL

>btaCCR6 XP_597941.2 Chr 9

MNSTNIYDSNEDYFGLANSSDYLLDDDSFLCSLQEVRSFSGLFVPVAYSLICVCGLLGNILVVVTFAFYKKAKSMTDVYLLNMAVADILFVLTLPFWAVNHATGEWIFSNAMCKLTRGIYAINFNCGMLLLTCISLDRYIAIVQATKSFRLRSRTLAHHKLICLAVWAVSILISSSTFTFNQKYKLQGGDVCEPRYHAVSEPIRWKLLMLGLQLLFGFFIPLVFMIFCYAFIVKTLVQAQNSKRHRAIRVIIAVVLVFLACQIPHNMVLLVTAVNLGRTGRSCGSEKLLGYAKNVTEVLAFLHCCLNPALYAFIGQKFRSYFLKIMKDLWCVRKKQKAPGFSCSRLHSDTFTSRQNSETADNDNPSSFTM

>btaCCR7 NP_001020101.2 Chr 19

MDLGKPMKNVLVVALLVIFQVCLCQDEVTDDYIGDNTTVDYTLYESVCFKKDVRNFKAWFLPIMYSIICFVGLLGNGLVMLTYIYFKRLKTMTDTYLLNLALADILFLLTLPFWAYSAAKSWVFGVHVCKLIFGIYKISFFSGMLLLLCISIDRYVAIVQAVSAHRHRARVLLISKLSCLGIWMLAIVLSTPEVMYSGIQKSSSEQALRCSLVTEHVEALITIQVAQMVVGFLIPLMAMSFCYLVIIRTLLQARNFERNKAIKVIIAVVVVFVAFQLPYNGVVLAQTVANFNITSGTSCELSKQLNIAYDVTYSLACVRCCVNPFLYAFIGVKFRSDLFKLFKDLGCLSQEQLRQWSSCRHTRRSSMSVEAETTTTFSP

>btaCCR8 XP_876202.2 Chr 22

MDYTPEPNLTTATDFYYPDIYSSPCDGEGRESKLLLAVFYCILFVFGLLGNSLVILVLVACKKLRSVTDVYLLNLALSDLLFVFSFPFQTHYQLDQWVFGTVMCKVVSGFYYIGFFSSMFFITLMSMDRYLAVVHAVYALKVRTISMGTALSLVVWLTALVATSPLLVFYQVASENGILQCYSYYNQQTLKWKIFIHFEVNILGLLIPFSILMFCYIRILHQLKSCQNHNKTKAIKLVLIVVVASLLFWVPFNTVLFLTSLHDMHVLDGCVMSQQLTYATHVTETISFTHCCVNPIIYAFMGEKFKKHLSELFRKSCSHTLVYIGRQVSREVLEKSSSNQQSSRSSTVDYIL

>btaCCR9 NP_001091537.1 Chr 22

MVPTEATSLIPNLSDDYGYDGTLPMEYDGNFTDYFCEKSHVRQFAGHFLPPLYWLVFIVGGVGNSLVILVYWYCTRVKTMTDMFLLNLAIADLLFLATLPFWAIAAADQWKFQTFMCKVVNSMYKMNFYSCVLLIMCISVDRYIAIAQAMRAQMWRQKRLLYSKMVCFTIWVTAAALCLPELLYSQVKEEHGIAICTMVYSSDDSTKLKSAVLTLKVILGFFLPFVVMACCYTIIIHTLIQAKKSSKHKALKVTITVLTVFVLSQFPHNCVLLVQTIDAYAMFISSCALSIKIDICFQVTQTVAFFHSCLNPVLYVFVGERFRRDLVKTLKNLGCISQAQWVSFTRREGSLKLSSMLLETTSGALSF

>btaCCR10 XP_584874.4 Chr 19

MGTEPAEQVSWGPYSGEDEAYSVEPLPELCYKADVQAFSRAFQPSVSLTVAALGLAGNGLVLATHLAARRAARSPTSAHLLQLALADLLLALTLPFAAAGALQGWSLGSATCRAISGLYSASFHAGFLFLACISADRYVAIARALPAGPRPSAPGRAHLVSVIVWLLSLLLALPALLFSQDGHREGQRRCRLIFPEGLTQTVKGASAVAQVVLGFALPLGVMAACYALLGRTLLATRGPERRRALRVVVALVAAFVVLQLPYSLALLLDTADLLAARERSCPASKRKDLALLVTGGLALARCGLNPVLYAFLGLRFRQDLRRLLRGGGCSPGPHPGGGGGRCPRRPRLSSCSAPTETNSFSSWDH

>btaCX3CR1 NP_001096028.1 Chr 22

MHTTLPESTSENFEYYDLAEACDMGDIVALGTVFVVILYSLVFAFGLVGNLLVVFALINSQRSKSITDIYLLNLALSDLLFVATLPFWTHYVINEQGLHHATCKLITAFFFIGFFGGIFFITVISVDRFLAIVLAANSMSNRTVQHGVTTSLGVWAAAILVATPQFMFTREKENECFGDYPEILQEIWPVILNTEINFLGFLLPLLIMSYCYFRIMQTLFSCKNHKKAKAIRLIFLVVVVFFLFWTPYNVMIFLQTLNLYDFFPKCDVKRDLKLAISVTETIAFSHCCLNPLIYAFAGEKFRRYLYRLYRKCLAVLCCHPDHLSFSSSLSESQRSRRESVLSSNFTHYTSDGDASILL

>btaXCR1 XP_875303.1 Chr 22

MEPSDIPESTTFYEYDPQSFLCEKRTFVFATVSTTILYCLVFFLSLVGNSLVLWVLVKYESLESLTNVFILNLCLSDLVFSCLLPVWILGYHWGWVLGDLLCKLLNMVFSISLYSSISFLTIMTIHRYLSVVSPISSLRVHTLQRRVLVTAAVWAASILSSIPDAIFHKVFPSGCDYSELEGFLASVYQHNVIFLLSVGVILFCYVEILRTLFRSRSKRRHRTVRLIFTIVAAYFLSWAPYNLILFLQTLLKLGVIQSCEVSQQLDYALLICRNVAFSHCCFNPVLYVFVGVKFRRHLKSLLRRFWLCRQQVPSLPPSPHPPGAFTYEGISFY

>btaCXCR7 NP_001091851.1 Chr 3

MDLHLLDYSEPGNFSDISWPCNGSDCIAVDTLQCGHIPNKSVLLYTLSFVYIFIFVIGMIANSVVVWVNIQAKTTGYDTHCYILNLAIADLWVVVTIPVWVVSLVQHNQWPMGELTCKVTHLIFSINLFGSIFFLTCMSVDRYLSVAYFASTSGRKKRLVRRAVCVLVWLLAFGVSLPDTYYLKTVTSASNNETYCRAFYPEHSVKEWLISMELVSVILGFAIPFCIIAVFYFLLARAIASSSDQEKQSSRKIILSYVVVFLVCWLPYHLVVLLDIFSILHYIPFTCQLEAFLFTALHVTQCLSLVHCCVNPVLYSFINRNYRYELMKAFIFKYSAKTGLTKLIDASHVSETEYSALEQNAK

>btaCCBP2 NP_001015581.1 Chr 22

MATTASPLPPTTKVASSENSSSFYDYEYYLEDMIFMLCRKDEVLSFGRVFLPVFYSLIFVLGLVGNLLLLAVLLRFVPRRRMTETYLLNLAISNLLFVVTLPFWGISVAWHWVFGSVLCKVVSTLYTVNFYSGIFFISCMSLDKYLEIVCARPYHRLRTRAKSLLLAASVWAMALAVSIPDMVFVRTHENSPGVWECYADFGGHGTIWKLFLRFQQNLLGFLLPLLAMIFFYSRIGSVLVSLRPPGQRRALRMAVALVVAFFVLWFPYNLTLFLHSLLDLQVFGDCRVSQHLDYALQVTESIAFLHCCFTPVLYAFSSHRFRQYLKAFLATVLRRQQALPSSYSESSGLTVQEDVMGMSDLGERPAESSPTRLSISSGLLRLDS

>btaCCRL1 NP_776690.1 Chr 1

MAVEYNQSTDYYYEENEMNDTHDYSQYEVICIKEEVRKFAKVFLPAFFTIAFIIGLAGNSTVVAIYAYYKKRRTKTDVYILNLAVADLFLLFTLPFWAVNAVHGWVLGKIMCKVTSALYTVNFVSGMQFLACISTDRYWAVTKAPSQSGVGKPCWVICFCVWVAAILLSIPQLVFYTVNHKARCVPIFPYHLGTSMKASIQILEICIGFIIPFLIMAVCYFITAKTLIKMPNIKKSQPLKVLFTVVIVFIVTQLPYNIVKFCQAIDIIYSLITDCDMSKRMDVAIQITESIALFHSCLNPVLYVFMGTSFKNYIMKVAKKYGSWRRQRQNVEEIPFESEDATEPTSTFSI

>btaCCRL2 NP_001069200.1 Chr 22

MANYTPAPEDDYDVFIEDDLSDDEIEPCTPYDPKILSAQLVPYLYTTVFMVGLLDNILVVFILVKYKGLRQAENMSFLNLALSNLGFLLTLPFWAYAASHGEGFDDPLCKILLLLYSIGLYSEAFFNVLLTVQRYKEFFHVRRRFSACRTVAGSIFISVLVWVTATLVTLPELVSYKPQMQSQKYKCFFTGLHFLPADETFWKHFLTLKMNILGFLLPLFAFVYCYVRMRKTLQFRERNYGLFKLVFTIMAVFLLMWGPYNIVLFLSAFNEHFSLHGCGSSYNLNKSVQITRIIAATHCCVNPLLYVFLDKAFRKHLCHLFYLCSDTAPQPTEEPAQGASGEEYHLSS

>btaDARC NP_001015634.1 Chr 3

MGNCLYPVADDNSTKLAIKEDFLIDFPEDYYPDYNETDVEAAAPCHSCSLLNYSSLPFFILVSILGILASGTILYALLRPLFRWQLYQDRSTLVQLAVGSALFSIVVPILARGLSGALITSLCHLAHLVAYGSAFAQALLIGYHACLGPRLGAGQVPGLRLGVTVGLWGVAALLSLPVVLGSDTSQGLCTVTFSGEWETLRYIHAAACFAIFVLLPLGLLGTKGLKTVLGRAPCPWVDVLWVWFIFWWPQGMTLGLDSLVRSKAIVVSTCPAQQALDMLLDVAEALAILHCVATPLLLAWVCYQATHTSPPSLPLPTTQTSHLDTLGSKS

>lafCXCR1 ENSLAFT00000026816 Scaffold 3

MADIIDNIWNVTDLWKWFDDISNFTGMPPTEEDVGPCNIETETLNKYIVVTIYALVFLLSLLGNSLVMLVILYNRVSSSVTDVYLLNLAMADLLFALTLPIWAASKEHGWIFGTPLCKVVSLLKEVNFYSGILLLACISVDRYLAIVHATRTLIQKRHLVKFICLGIWGLSLILSLPFFLFRQAYRLPYSSLIVCYEDIGNNTAKWRLVLRILPQTFGFVLPLLVMVFCYGFTLRTLLEARMKQKYRAMRVIFVVVFVFLLCWLPYNLVLVTDTLMRTGLIEETCERRNDIGRALDATEILGFFHSCLNPVIYAFIGQNFRNGFLKILATHGLVSKDFLARHHITSYTSSSTNVSANF

>lafCXCR2 ENSLAFT00000033749 Scaffold 3

MEEFNFDNLSLEDFFGDDSNYSYSTGIPSILSDSAPCRPESLEINKNVVITIYALVFLLSLLGNTLVMLVILYNRVSCSVTDVYLLNLAMADLLFALSLPIWAASKKNGWIFGTTLCKVVSLLKEVNFYSGILLLACISVDRYLAIVHATRTLIQKRHLVKFICLGIWGLSLILALPIVLFRKAIYPPYSSPVCYEDIGNNTANWRLVLRILPQTFGFILPLLVMVFCYGFTLRTLFEAHTGQKHKAMRIIFAVVLVFLLCWLPYNLVLVTDTLMRIGVIKETCERRNDIGWALDVTEILGFLHSCLNPLIYAFIGQKFRHGLLRILATHGLISKDFLAKDGRPSFVGSSSANTSTTL

>lafCXCR3 ENSLAFT00000034594 Scaffold 24

LPPQMSEHQVLDAPDFAFLENCSSSYDYEDNETDSCCASPPCPQDFSLNFDRAFLPALYSFLFLLGLLGNGAVAAVLLSQRAALSSTDTFLFHLAVADALLVLTLPLWAVDAAVQWVFGSGLCKVAGALFNINFYAGALLLACISFDRYLSIVHATQLYRRRPPARVALTCVVVWGLCLLFALPELIFLSAHHDDRLNATHCQYNFPQVGRTALRMLQLVAGFLLPLLVMAYCYARILAVLLVSRGQRRLRAMRLVVMVVVAFALCWTPYHLVVLVDTLMDLGALARDCGRESRVDVAKSVTSGLGYMHCCLNPLLYAFVGVKFRERMWMLLMRLGCPGQRRHQRQAAPSYRDSSWSETTEATYSGL

>lafCXCR4 ENSLAFT00000006282 Scaffold 3

MDNETEEVGSGDYDSIKEPCFREENAHFNRFFLPTIYSIIFLTGIVGNGLVILVMGYQKKLRSMTDKYRLHLSVADLLFVLTLPFWAVDAVAGWYFGKFLCQAVHVIYTVNLYSSVLILAFISLDRYLAIVHATNSQKPRKLLAEKVVYVGVWIPALLLTIPDFIFANVTESEEKYICDRFYPSDLWMVVFQFQHIMVGLILPGIVILSCYCIIISKLSHSKGHQKRKALKTTVILILAFFACWLPYYVGISIDAFILLEIIKQGCEFENTVHKWISITEALAFFHCCLNPILYAFLGAKFKTSAQHALTSVSRGSSLKILSKGKRAGHSSVSTESESSSFHSS

>lafCXCR6 ENSLAFT00000005164 Scaffold 12

MEEYDYTDPEFFNTSSDSRQEHNRFLQFSKFFLPCMYMVVFVCGLVGNSLVLVIYIFYQKLKSMTDLFLMNLPLADLVFVCTLPFWTYASIHEWVFGTSMCKILLGTYTLNFYTSMLILTCITVDRFLAVVQATKAYNQQAKRMIWAKVICLSMWVISLLVSLPQIIYGNVSIHDKLICDYKDEKISTVVLATQMALGFFLPLFTMIICYSVIIKTLLHARGFQKHKSLKIIFLVVAVFLLTQTPFNLMKLIRSTSWEYHAMTSFHYAIIVTEAIAYLRACLNPVLYVFVGLKFRKNFWKLVKDIGCLPYLGVSSQWKFSEDTSKISSASHNVEATSMLQM

>lafCCR1 ENSLAFT00000011887 Scaffold 12

MEITTTPEAYDMTTEYDYGGSTPCQKREVRAFGAQLLPPLYSLVFVIGLVGNILVVLVLMQYKRLRSMTSIYLLNLAISDLLFLFTLPFWIDYKLKDNWVFGDVMCKFLSGFYYTGLYSEIFFIILLTFDRYLAIVHAVFALRARTITFGIISSIVSWALAILASIPGWQFSKTQWEVSHYTCSLFFPYESLKMWKQFQALKLNILGLVLPLLVMIVCYTGIIKILLRRPNEKKAKAVRLIFVIMIIFFLFWTPYNLAVLVSAFQDSLFSNECEQSKQLDLAIQVTEVIAFTHCCLNPIIYVFIGERFRKYLRQLFHRLIEVHLAKWLPFLATERLERASSVSPSTGEHELSMGF

>lafCCR2 ENSLAFT00000032634 Scaffold 12

MLSTSQSFFTRSIAGNDKEATISYDYDCGNHASEPCRKTDLKQIAAQILPPLYSLVFIFGFVGNMLVVLTLINCKKLKSMTDIYLLNLAISDLLFLLTLPFWAHHAANGWVFGDSGCKIFTGLYHIGYFGGIFFIILLTVDRYLAIVHAVFALKARTVTFGVVTSGATWVVVVLVSLPGIIFTKSQEEDSHYVCGPAFPIIWKNLHTIMRNILSLVLPLLVMVICYSGILKTLLRCRNEKKHKAVRLIFAIMIVYFLFWAPYNIVLDLITFQEFFGLDNCDSSNRLDQAMQVTETLGMTHCCINPIIYAFVGEKFRRYIWVFFRKHIAKRLCKQCPVFFGETADRVSSGYTPSTGEQEVSVGL

>lafCCR3 ENSLAFT00000014611 Scaffold 12

METSIDESETEVEVFGTTPYDYAGLWPCEKVNIKDLGAQFLPPLYSLVFIVGLLGNVVVVVILTKYRRLRIMTNIYLLNLAISDLLFLVTLPFWIHYTGWNKWVFGQCMCKFLSGFYYMGLYSEIFFIVLLTIDRYLAIVHAVFALRARTVTFGVITSIFTWVLAGLAALPEFAFHEFQEEDEYSACSPRYPENEEDTWKRFHALRMSILGLALPLLIMAICYTGIIKTLLRCPSRKKYKAIRLIFVIMVVFFIFWTPYNLVLLLSAFQVILPENNCEQSKQLDLAMAVTEVVAYTHCCVNPIIYAFVGERFRKYLHHFFYRHVGIYLSKYLPFFPREKLERASSISPSTGEQKLLSEEF

>lafCCR4 ENSLAFT00000001079 Scaffold 36

MNPTDITDTTLDESMYNNYYLFENIPKPCTKEGIKAFGELFLPPLYFLVFLFGLLGNSVVVLVLIKFKRLKSMTDVYLLNLAISDLLFVFSLPFWGYYAADQWVFGLGLCKVISWMYLVGFYSGIFFIMLMSIDRYLAIVHAVFSLRARTLTYGVITSLATWSVAILASLPGLIFSTCYTERNHTYCKTKYSFNSTTWKVLSSLEINILGLAVPLGIMLFCYSMIIRTLQHCKNEKKNRAVKMIFAVVVLFLGFWTPYNVVLFLETLVELDILQDCAFERHLDYAIQATETLAFVHCCLNPVIYFFLGEKFRKYIIQLLKTCRGPFVLCQYCGLLQIYSAETPSSSYTQSTVDHDLHDAL

>lafCCR5 EF524204.1 Scaffold 12

MDYETTSPMDYGIDYSISDPCQKIDVRQIAAQLLPPLYSLVFIFGFVGNMLVVLTLINCKKLRSMTDIYLLNLTISDLLFLLTLPFWAHYAANGWVFGNVVCKLFTGLYHIGYFGGIFFIILLTIDRYLAIVHAVFAVKARTVTFGVVTSGVTWVVAVLVSLPGIIFTRSQKEGSRYTCSPHFPSSQYHFWKNFQTLKITILGLVLPLLVMVICYSGILKTLLRCRNEKKKHKAVRLIFAIMIVYFLFWAPYNIVLDLSTFQGFFGLDNCDSSNRLDQAMQVTETLGMTHCCINPIIYAFVGEKFRNYLLVFFRKHVAKRLCKHCSIFQREAPERVTSVYTRSTGEQEISTGL

>lafCCR6 ENSLAFT00000020703 Scaffold 69

SNINDDYFGSTDMDYSEDGWAFPCSLWEVRKFSSLFVPIAYALICIFGLLGNILVVITFAFYKKAKSMTDVYLLNMAIADILFVLTLPFWAVNHARGTWYFSNVLCKLIKGIYAVNFNCGMLLLTCISLDRYVAIVQATKSFRLRSKTLAHSKVICLVVWVASIIISSSTFIFSQKYNIQGVDVCEPKYHNVLEPVKWKLLMLVLQLLFGFFIPLMFMIFCYMFIVKTLVQAQNSKRHKAIRVIIAVVLVFLVCQTPHNMVLLVTAATMGSMGRSCSSEKLIAYTKNVTEVLAFLHCCLNPVLYAFIGQKFRNYFLKIMKDLWCVRRKPQSAGFSCSRMYSEGAVSKQTSETVDNENASSFTM

>lafCCR7 ENSLAFT00000022724 Scaffold 31

LSPAGKPMKSVLVVALLVVFQVCLCQDEVTDDYIGDNTTVDYTLYESVCFKKDVRNFKAWFLPVMYSVICFVGLLGNGLVMLTYIYFKRLKTMTDTYLLNLAMADILFLLTLPFWAYSAAKSWDFGVHFCKFIFGTYKVSFFSGMLLLLCISIDRYVAIVQAVSAHRHRARVLLISKLSCVGIWILAVVLSIPELLYSGIQKSSSEQALRCSLITEHVEALITIQVAQMVVGFLIPLMAMSFCYLVIIRTLLQARNFERNKAIKVIIAVVVVFIVFQLPYNGVVLAQTVANFNFTSSSCELSKQLNIAYDVTYSLACVRCCVNPFLYAFIGVKFRSDLFKLFKDLGCLSQERLRQWSSCRHSRRSSMSADVETTTTFSP

>lafCCR8 ENSLAFT00000006343 Scaffold 36

MDYTLEPNVTEVTDYYYPDVFSSPCDGELIQRGSSLLLAIFYCLLFVCSLLGNSLVILVLITCKKLRSITDVYLLNLALSDLLFVFSFPFLTHYHLDQWVFGTVMCKVVSGIYYIGFFSSMFFVTLMSVDRYVAVVHAVYALKVRTVSMGTVLSLAVWLIAIIATSPLLVFYQVASEDGILQCYSSYHQQTLKWKIFTYFEINILGLLIPFTILFFCYISILHQLRGCQKHKTKAIRLVLIVVAASLLFWVPFNVVLFLTSLHSMHVLDGCVISQRLIYATHVTETISFTHCCVNPVIYAFMGEKFKKHLSEIFQKSCNYIFLCLGKRDSKEGWERSSSFHQRSSGSSSIDYIL

>lafCCR9 ENSLAFT00000007500 Scaffold 12

MLSCVPLQSLNLNLSDEYSYDSASSEAYEYMHDDFTDFFCKKSNVRQFASYFIPPLYWLVFIVGTLGNSLVILVYWYCTRVKTMTDMFLLNLAIADLLFLLTLPFWAIAAADHWKFHSVTCKVVNSMYKMNFYSCVLLIMCISVDRYIAIAQAMKAQNWRQKRLLYSKMVCITIWVIAAVLCIPEILYSQVRGESDVTVCTMVYPSKDSTNVKSAVLTLEVIVGFFLPLVVMACCYTIIIHTLLQAKKSSKHKALKVTITVLTAFLLSQFPYSCILLVQTINAYTMFISNCAISTNIDICFQVTQTIAFFHSCLNPVLYVFVGERFRRDLMKTLRNLGCISWAQWVSFTRREGSLKLSSVLLETTSGALSL

>lafCCR10 ENSLAFT00000032640 edited Scaffold 31

MGTEPAEQVSWGPYSGDDDEAYSSEPLPELCYKADVQAFSRAFQPSVSLTVAALGLAGNGLVLATHLAARRVARSPTSAHLLQLALADLLLALTLPFAAVGTLQGWSLGSATCRAISGLYSASFHAGFLFLACISADRYVAIARALPAGPRPSAPGQAHLVSAVVWLLSLLLALPALLFSRDGHREGQRRCRLIFPEGLTQTVKGASAVAQVALGFALPLSVMAACYALLGRTLLAARGLERRRALRVVVALVAAFVVLQLPYSLALLLDTADLLAARELSCPASKRKDLALLVTGALALARCGLNPVLYAFLGLRFRQDLRRLLRGGGCSPGPHPRGRCPRRPRLSSCSAPTETHSLSF

>lafCX3CR1 ENSLAFT00000004804 Scaffold 36

MSTPFTNSYTEDHVYDEGAEACQEGDIVDFGTVFLPIFYSCVFAFGLVGNLLVVFVLTNSQKPKSITDIYLLNLAFSDLLFVATLPFWIHYVISDQGFSNAVCKLITAFFFIGFFGGIFFITIISIDRYLAVVLATHSMRNRTVQHGVTISLSVWAVAILVAAPQFMFTKQNGSECLGDYPEILQEVWPVLQNVGANLLGFLLPLLTMSYCYLRIIWTLFSCKNHKKSKAIKLIFLVVIMFFLFWTPYNVMIFLETLKAFHFFPSCDVKKKLRLALSVTETIAFSHCCLNPLIYVFAGQKFRRHLYHLYRKYLAILCRHPVEVSFYPSESQRSRRESVLSSNFTECTSHGDVSILL

>lafCCBP2 ENSLAFT00000036410 Scaffold 12

MAASTSPPLLTTEDTNSENSSYYYYDYFVDIPFMLCRKDAVMSFGKIFLPVFYSLIFVLGLGGNLLLLTVLLRYVPRRQMAEIYLMNLAISNLLFVVTLPFWGISAAWHWVFGNFLCKMVSTLYTINFYSGIFFISCMSLDKYLEIVHAQPHHRLRTRAKSLILSAVVWGVALAISIPDMVFVQTHENPKGMWRCYPDFGGHGTFWKLFLRFQQNLLGFLLPLLAMIFFYSRIGCVLVRLKPPGQSRALRMAAALVVAFFVLWFPYNLALFLHSLLDLQVFGDCNISHRLDYALQVTESIAFLHCCFTPVLYAFSSRRFRQYLKTFLATVLGWHLAPSTAQASLSTCSGSSCLTAQEEMTSMNELGKKHADSSPNKGDVGKN

>lafCCRL1 ENSLAFT00000014704 Scaffold 103

MALGHNQSTDYYYEENEVNGTHDYSQYEMICVKEEVRRFAKAFLPAFFTIAFVIGLAGNSVVVAIYAYYKKQRTKTDVYILNLAVADLLLLFTLPFWAVSAVHGWVLGKMMCKVTSALYTVNFVSGMQFLACISMDRYLAVTKAPSQSRVGRPGWIICSCVWTAAVLLSIPQLVFNTVNDNARCIPIFPHHLGTSVKAAIQMLEICIGFVVPFLIMGVCYFITARILIKTPNIKKSRPLKVLLTVVVVFIVTQLPYNIVKFCQAIDIIYSLITDCSMSKRMDVAIQVTESIALFHSCLNPILYVFMGASFKSYITKVAKTYGHWRRQRQNVEEIPFDSEGPTEPTSSFSI

>lafCCRL2 ENSLAFT00000007852 Scaffold 12

YPRGSLRMAGYTAAPDEDYDVLIEGDLNGSDTEPCDKYDARVLSAQLLPNLYSIVFVVCLLGNLLVVLILAKYKGLRHVKNIYFLNLAFSNLLFSLTLPFWAYTASAGGSLGDTMHTILIGVSAVGLYSEVCFNVLLTVHRYLVQSPSPTPCTASCGILTSILVWTVAVLISLPECMAYTLQMEGQENKYSFSIPHFLPAAEKSWKHFLTLKMNILGLLFPLFIFIFCYVRMRRRLTCRDNENGLFKLVFAIMAVFLLMWAPYNVVLFLSTFKQDFSLDDCKSTYGLDQGIQITEIVAATHCFVNPLLHMFFDQDFRRHLGHLLHLSNNTSPQPGKESGRDALREQPESSTQV

>lafDARC ENSLAFT00000029558 Scaffold 33

MGNCLHPVTSSVDDNSSKFSFENYEWNMTYDSLDYNDPSLQAAAPCHSCNLLDDSSLPFFIMASILGILASGTALLAFLRPLFHWELCRGRPILVQLAVGSALFSVVVPILAPGISSSHSTALCRLGHSVWYGSAFAQGSADRMPGLPGPQAGCSQVSSHTLVLTVGLWGTAALLALPITLARDTSQGLCTLAYSRGLGTLQCIHSAVCFAIFFLLPLGLVGAKGLKKVFGRGPGPWVDILWVWFSFWWPHGVILGFDTLVRSGTLPLQTCLAQQILDLLLQLAEALATLHCVATPLLLAFFCHRTVHTSFLSLPLHGRQSSHVGTLGSKS

>mdoCXCR1 XP_001366526.1 Chr 7

MSSRYILNEYNWGSAFDNYTDFIASDAMPCDVGSWRINKYFVMVIYSLVFLLSLLGNSLVILVILYNRISRSVTDIYLLNLAIADLLFALSLPIWAASKIKGWLYGTALCKIVSLLKEVNFYSGILLLACISVDRYLAIVHATRSLTQKRHWVKFVCLGIWGLSLLLSMPIILSREAFKSEDYGFVCYEDLGKKTEIWRLVLRILPQVFGFVLPLLVMLFCYGFTLRTLFEARMGQKHRAMKVIFAVVLIFLLCWLPYNLVLVADTLMRTHIIEETCERREEIDQAISVTEVLGFLHSCLNPIIYAFIGQKFRYSFLKILAAHGVVSKEFLSRHQISSSSFASSSVSAAV

>mdoCXCR2 XP_001366576.1 Chr 7

MSIEEIYNLTFGDDDFLYSGYGTGLPPLSEDAAPCHSESSGLNKYFVIIIYSLVFLLSLLGNSLVILVILYNRISRSVTDIYLLNLAIADLLFALSLPIWAASKIKGWLYGTPLCKIVSLLKEVNFYSGILLLACISVDRYLAIVHATRTLTQKRHWVKFVCLGIWGLSLLLSMPIILSREAFKSDDYGFVCYEDLGKNTTTWRLVLRILPQVFGFVLPLLIMLFCYGFTLRTLFEAHMGQKHRAMKVIFAVVLIFLLCWLPYNLVLVADTLMRTHIIEETCGRREEIDQAISVTEVLGFLHSCLNPIIYAFIGQKFRYSFLKILAAHGVVSKEFLARHSKPSVHGSSSGNTSTTL

>mdoCXCR4 XP_001370420.1 Chr 4

MALRAQSFPFTGLIIEFHDNNTVEGFGSGDYEGKEPCYPNENEDFNRIFLPTVFSIIFVTGIVGNGLVIIVMGYQKKLRSMTDKYRLHLSVADLLFVLTLPFWAVDAAANWYFGNFLCKAVHVIYTVNLYGSVLILAFISLDRYLAIVHATNSQRPRKLLAEKVVYLGVWLPAVLLTVPDIIFASTSEAGGRYVCDRMYPHENWRISFRFQHILVGLVLPGLIILTCYCIIISKLSHSKGHQKRKALKTTVILILAFFACWLPYYIGISIDTFILLEVIKQDCDFDKAVHKWISITEAVAFFHCCLNPILYAFLGAKFKTSAQNALTSVSRGSSLKILSKGKRGGHSSVSTESESSSFHSS

>mdoCXCR5 XP_001380659.1 Chr 4

MLPGGGAACLWREAPPLLGGRAATLPASSPEVPAPHPPPAHPGAAERPFPDFSNYSNHSNELLEPDEYLCPEVGGWADQLGLKAVFVLLAYSAIFLLGMMGNTLVLVILKSHHMSRSSTETFLLHLAVADLLLVLTLPFAVVEGAVGWVLGAFFCKAVSALHKINFYCSSLLLACIAVDRYLAIVHAVHTYRHRRLLSVHATCAAVWLASFLCALPELLFVKVSNSGANNSATCSFSGQGLAGSNAWLTSRFLYHVGGFLIPLLVMSWCYAGVVRRLCQAQRRHQRQKAIKVAILVTGVFFFCWSPYHVVIFLDTLVMLDAVSKSCQLNDHLATAITTCEFMGLAHCCLNPVLYTFVGVKFRSDLIHLLGKLGCMVPAAHHRFLSSWRKGSSSESENATSITTF

>mdoCXCR6 ENSMODT00000034208 Chr 8

MEDGNNRLTMEEYAYDPDVWKTLENVSKEGHENFQKFGKIFLPCTYLLVFSCGLVGNCLVLAVYIFFQKAKSLTDQFLMNLPIADLLFLCTLPFWVYATIHEWVFGQVMCKVILGMYTLNFYTSMLFLTSITIDRLLAVVQATKTYNYQAKRMTVGKNLCATIWLISLTVTIPQFMYARVFPNDKRVCHNEEEGISTVVLSTQMTIGFFLPLIAMIVCYSVIVKTLVQAKRFQKHKSLKIIFLVVVVFIATQLPFNLMKLIRTTNWEYDTDPRFLYGLMVTEAIAYLRVCLNPVLYAFVGLKFRRNFWKLMKALKCPQAAGKAGQWKSSDDTSRSCSASNHAQATSLYQL

>mdoCCR1 XP_001379588.1 Chr 8

MGTSAPLLDFTVPEYNSSFPEYYYFGEDAIPCFKYNIKELASKFLPPLYSLVFIVGLLGNAAVVLILTKYKRLTSMTNIYLFNLAISDLLFLVTIPFWIHYEKQNDWVFGNAMCKLLTGLHYLGLYSEIFFIILLTVDRYVAIVHAVFAIRVRRVVLSVTTSIITWVIALLVSLPDIIFTKTQWEFTHYTCSLHFPHETARIWRKFQALKLNFLGLILPMWIMIACYTGIIRILLKRRNEKKWKAVKLIFAIMIIFFLFWTPYSITILISAFDEFIFTLDCEKSKQLDLAIQVTEVIAFTHCCVNPVIYAFIGERFQKYLSHFARNHIAVHLCRRVPSFLKDRLERASSISPPTGEHEISDEF

>mdoCCR2 XP_001369334.1 Chr 8

MSFSSIGQIKMEEDAITTTFYYDFKVPCQNFDVKQTASLILPPLYSLVFIFGFVGNALVFLILIRCKKLKSMTDIYLLNLAISDLLFIVTLPFWAHYAADQWIFGDALCKVLTGFYHMGFFGGVFFIILLTMDRYLAIVHAVFALKARTVTFGILTSVITWVVAGFASLPAIIFTKSQKEGIQHTCSPHFPSEQSTLWKNFQTLKMNLLGLVVPLLVMIVCYSGIIKTLLRCRNEKKKHKAVRLIFVIMVVYFLFWAPYNLALLLNTFQDFFGLDNCESSKRLGRAIQLTETLGMTHCCINPVIYAFVGEKFRRYLSTFFRKYIVRHFCKRCPFFHGEALDRVSSTYTPSTGEQEVSAGL

>mdoCCR3 XP_001379609.1 Chr 8

MATYSMDINEEATTFDYEFSTPCQKMNIKELGAKFLPPLYSFVLIVGLLGNILVVLILTKYKRFKSMTNIYLFNLAISDLLFLFTLPFWIDYARKNDWVFGHTMCKILSGLYYMGLYSEIFFIVLLTIDRYLAVVYAVFALKARTVTFGIFTSIVTWGLSALAALPEVIFHESQEDLENHICSPRYPEDNEATWKRFQALRMNILGLAIPLAIMIICYTGIIKKLLSCRNEKKYKAVKLIFVIMIVFFLFWAPYNLTLLLNAFQSSFFTADCERSKQLDLAMQITEVIAYTHCCVNPVIYAFVGERFRKYLCLFVRRHIAVHLCKYIPFLPGEKLERVSSISPSTGEQELSAAF

>mdoCCR4 XP_001378619.1 Chr 8

MEPLETTMDYSGYYIYDLPSPCSKGGVRDFGQLFLPPLYSLVFLFGLLGNFMVVLVLLKYKRLKSMTDVYLFNLAISDLLFVFSLPFWVYYVAAHQWVFGTAFCKIISYMYLVGFYSGIFFIMLMSVDRYLAIVHAIFALRARTLTYGVITSLVIWLVAILASLPVLLFSTSYTENNHTYCKEKYPGNSTTWKVLRSLEVNILGLLIPLGIMLFCYTMIIKTLHRCKNDKKNKAVKMIFAVMIVFLVFWIPYNIVLFLEILVELEVLQDCTFDMQLDYALQATETLAFVHCCLNPVIYFFLGEKFRKYIKQLFKTCRGPLKLCKHCSFLQVYASDTPSSSHTQSTMDQELHDAL

>mdoCCR5 XP_001379621.2 Chr 8

MDNESTTPFYYPDYPIEEPCQKLDVKHTASRLLPPLYSLVFIFGFVGNALVFLVLIRCKKLKSMTDIYLL

NLAISDLLFIVTLPFWAHYAADQWIFGDALCKVLTGFYHMGFFGGVFFIILLTMDRYLAIVHAVFALKAR

TVTFGILTSVITWVVAGLASLPAIIFTKSQKEGIQHTCSPHFPSEQFSAWKNFQTLKMNLLGLVVPLLVM

IICYSGILKTLLRCRNEKKKHKAVRLIFVIMIVYFLFWAPYNLVLLLNTFQAFFGLDNCESSKRLDRAIQ

ITETLGMTHCCINPVIYAFVGEKFRRYLSTFFHKYIVRRFCKRCPIFQGEALDRVSSMYTRSTGEQDIST

GL

>mdoCCR6 XP_001381581.1 Chr 2

MDTKRPVTAKPQLCVSAAGKGAIRSGTGGAERADTVGRRGEEDDTMNAVGEPMNSSSFYDDFTEVTSSTDYFIGDAVLCVMEEVRKFTTLFVPVAYSLICVFGLLGNILVVITFAFYKKAKSMTDVYLLNMAIADILFILTLPFWAVNHATGSWKFSNIMCKLTTGIYAINFNCGMLLLTCISLDRYIAIVQATKSFRLRTWTLAYSKMICLMVWLFSIIISISTFVFNQKYTIQGRDVCEAKYHTTSEAVKWKILILVLQLLFGFFIPLLFMIFCYMFIVKTLVQAHNSKRHKAIRVIIVVVLVFLVCQVPHNMVLLVVASNIGRLRSCSDEKLISYTRSVTEVLAFLHCCLNPVLYAFIGQKFRNYFLKIMKDLWCMGKKHKLTGFSCSKMHSETYISRQTSETYENENASSFTM

>mdoCCR7 XP_001369798.1 Chr 2

MTDVLTENKVIAYYGYYKFPIFWNKNNAHHPLQRKRMQNVMVVTLLVICQVCLCFDGVTDDYGDENTTVDYSQFETLCYKKEVRDFKAWFLPTMYSVICFVGLLGNGLVMLTYIYFKRLKTMTDIYLLNLALADILFLLTLPFWAASAAKSWMFGPFLCKAVYCIYKMSFFSGMLLLLCISVDRYFAIVQAVSAHRHRNRIILISQISCCVVWVLAFLFSIPELIYSNILKNGRCSLVTEDLETFTTIIQVSQMVIGFLIPLLVMFSCYLVIIRTLLQARNFERNKAIKVIIAVVIVFIVFQLPYNGVVLAKTVAALNKTATDCEHSKQLDIASDVTYSLACFRCCLNPFLYAFIGVKFRQDLFKLLKTLGCLSQERLLNLSSCRQNRRFSMSMEAETTTTFSP

>mdoCCR8a XP_001378660.1 Chr 8

MDYTPEPNVSVTTEDYYPEILASPCHTAFTQKSSSLLLALFYCLLFGFGLLGNTLVILVLVACKKLRSMTDVYLLNLAISDLLFVFSFPFLTHYTLDQWVFGNIMCKTISGIYYIGFFSSIFFITIMSMDRYLAIVHAVYALKVRTTRKGMAVSLLVWMVATLASVPLLVFYQVSSEEGTLKCYSFYDDRTIEWKLITHFEINILGLVIPLSILVFCYANILRHLKGCQNRHKIKAIRLVLVVVVAFFLFWVPFNMMLFLNSLHNLHILDGCDLSQKLTQATQITEVISFTHCCVNPVIYAFAGEKFKNHLLEIFQKYKWYSWLGKEHMARTSGHLFSSRSFSTDYIL

>mdoCCR8b XP_001378647.1 Chr 8

MDYTPEPNVSVTTEDYYPEILASPCHTAFTQKSSSLLLALFYCLLFGFGLLGNTLVILVLVACKKLRSMTDVYLLNLAISDLLFVFSFPFLTHYTLDQWVFGNIMCKTISGIYYIGFFSSIFFITIMSMDRYLAIVHAVYALKVRTTRKGMAVSLLVWMVATLASVPLLVFYQVSSEEGTLKCYSFYDDRTIEWKLITHFEINILGLVIPLSILVFCYANILRHLKGCQNRHKIKAIRLVLVVVVAFFLFWVPFNMMLFLNSLHNLHILDGCDLSQKLTQATQITEVISFTHCCVNPVIYAFAGEKFKNHLSEIFWKYKGYFWVCKERFTSNEYVDRSSVNQPSSRSSITDYIL

>mdoCCR9 XP_001379563.1 Chr 8

MTPTDFMESNYESTPEYYYNETESFCVKTSVREFASYFLPPLYWLVFVVGALGNSLVITVYWYCTRVKTMTDMFLLNLAIADLLFLLTLPFWAIAASDQWKFETVMCKLVNAIYKMNFYSSMLLIMCISIDRYIVIAQAMKAHHWRQKRLLYSKMVCFAIWVMAITLCIPEILYSQSETKSDITICTMVYNGSAMLKSTVLMLKVILGFFLPLIVMACCYAIIIYTLLQAKKSSKHKALKVTISVLTVFVLSQFPYNVILFVQAIDAYIVLISDCALSTRIDICFQVTQTIAFFHSCLNPVLYVFVGERFRRDLVKTLKKLGCISQAQWVSFTRREGSLKLSSMLETTSGALSL

>mdoCCR10 XP_001365440.1 Chr 2

MGTEPLEQVTWTPYSGESDDVYPSVSLPELCYKADVQSFSRTFQPSISLTVAAFGLAGNGLVLATHLAAHRAARSPTSAHLLQLALADLLLALTLPFGAAGALQGWTLGSTLCRTVSGLYSASFHAGFLFLACISADRYVAIAQASPGSHRPPAPGRTHVISAAIWLLSLLLALPSLLYGQDGQRDGQRRCRLVFPEGLTQAVKGASAVAQVILGFALPLSVMVVCYALLGRTLLAARGPERRRALRVVVALVVAFVVLQLPYSLALLLDTTDLLTARERSCSASKRKDMALLVTGGLALARCGLNPILYAFLGLRFRQDLKRLFRGGCFYHSSSQRGRCRRRPRLSSCSAPTETHSLSY

>mdoCX3CR1 XP_001378633.1 Chr 8

MTETILKATTLDYYEGITFGCDQKDILDFGMMFLPSFYSLVFAVGLVGNLLVIFALTNSSKHKSITDIYLFNLALSDLLFVTTLPFWSHYLLHEEGFNNALCKLITAFYFIGYYSGIFFITIISIDRYVAIVLAANSLNSRTVQHGVVVSLGVWAAAILVATPQFMFTEKVDKECLSNYPDSLQHIWPILKNVEINSIGFVIPLLIMCFCYFGIIRTIFSCKNHRKTRTIKLISVVVGVFFLFWTPYNVMIFVDTLKFYGFFESCEVKNNLRLVINVTETFAFVHCCLNPFIYAFAGEKFRKYLHHLYRKCLSILCGHDAHTQLLPPVAHRRESTFISSSITNYTSDESILL

>mdoXCR1 XP_001379577.1 Chr 8

MTSVGYSDDQLSSTPFTDYDTEGILCENHENFESMTLSHTVFYSLVFFLSLVGNSLVLWVLVKYESLESLTNVFILNLCLSDLVFSCLLPFWVVVHYYDWIFGEFFCKLLNMLFSISLYSSIIFLMVMTTHRYISVVHPLSNLGGHSCWSRVGIILGIWMASIMVSVPDTIFHTVLTDKSCDFSEPKWFLLSTYLHNLFFIFSLVVTLFCYVQILRTLFQSHTRRRHRTVRLIFTIVVAYFLSWAPYNMVLFLQTLVKLGYIQNCEVIKKLNYWEHICREFAFSHCCFNPVLYVFVGIKFRRHLKALCRQIWPSQWSLTSSPRSPRAFLYEDASFF

>mdoCXCR7 XP_001372691.1 Chr 2

MDLFSFDYSEPGNFTDLNWTCPNGDCITVDTVMCPSTTSRNVLLYTLSIFYIFIFVVGMIANSVVVWVNLQAKTTGYETHLYIFNLAIADLCVVVTIPIWVVSLVQHNQWPMGELTCKIAHLIFSINLFGSIFFLTCMSVDRYLSVTYFSNTSSHKKKVVRRLICILVWLLAICVSLPDSYYLKTVTLAANNETYCRSIYPEHSIKEWLLGMELLSVILGFIIPFSIIAIFYFLLARAITASGDQEKHSSQKIIFSYVIVFLICWLPYHAVVLLDIFSFLHFIPFNCQLENFLYTALHVTQCLSLVHCCVNPVLYSFINRNYRYELMKAFIFKYSAKTGLTKLIDASRVSEAEYSALEQNAK

>mdoCCBP2 XP_001377171.1 Chr 6

MAATFPPASSAPTSLENTSLYDYYYFENFPDTVCRKEAILSFGQVFLPIFYSLVFVLGLGGNLFFLIVLLYSARSRRVTEIYLLNLVVSNLLFTITLPFWGVSAAWHWGFGEVLCKIICTLYTTSLYGSIFFLGCMSLDKYLDVVHAQTHHRQWTSAKSRLLTGGVWTVALVLSIPDLVFARLQEGPSGRQNCHLDFGENGPVWKLVLRFQQSALGFILPLFTMAFFYTRIVCVLTVLRPRGRSRALWRAALLVVTFFALWGPYNITLFLHSLQDLQVLESCEVSKHLDYALQVTESIAFLHSCLSPFLYLFVHHRLKKHLKKLFHRPRRNDKYWQSGTKLTGS

>mdoCCRL1 ENSMODT00000034182 Chr 8

MDLEHNQSTDYYYEENEVNGTQDYSQYEILCVKEEVRNFAKVFLPTFYTVAFIAGIAGNSTVVAIYAYYKKQKTKTDVYIMNLAVADLLLLITLPFWAVNAVHGWVLGIPMCKLTSALFTINFVSGMQFLACISIDRYSAITKDPGHQRLGRPCWIICFSVWLIAILLSIPQLVFNTVNDKKRCLPIFSHYLGTTIQASIQILEICIGFVLPFLIMGTCYSITARKLIKMPNVKKSRPLQVLLAVVAVFIVTQLPYNIVKFWQAIDIIYSLIIDCEMSKRMDVAIQITKSLALFHSCLNPILYAFMGSSFKMHITKMVKKYGYWRRQRSNPEDIPFDSEGLTEPTSTFSI

>mdoCCRL2 ENSMODT00000014598 Chr 8

MDNSTLPDDYDVYIEDDFTHDLTEECHKYDDKVFAAQFLPTFYSLMFILGLVGNAFLVLILVKYKGLKVVVNIYFLNIAISNFLFLVTFPFSIHTAIHSWDLGGAMCKIISGFYSVGYYGYTCFLLLLIIHRYLAIVHTGRFHLATKKTTYGIIISSWGTAMLVTLPELVLSQVQMEDKDYICYFVQTYQYPPGDEKFWKYFLTLKMNILGILIPLFVFVFCFGGIKKTSRYKGRKHELLRLIFVITLVFIGLWTPYNLVLFLKTFQEHMNLSDCNSNYHLDKAIQVTKIIANIHCFISPIVYGFLDETFRKQLCYFFKPRNKTENHSSEGSEQSLTQMDHHANP

>mdoDARC XP_001379721.1 Chr 1

MGNCLHRAESGPQNSENWTFSSSEDYDWSLNYTYNYTDDDLNMAAPCRSCVLLDDTSLPFFAITSALGILGGGILLFALLKPLACWNLCPDRTILAQLAGGSTLFSIVLPILAPGWNPALDTVVCCMAHGLWYASAFAQALLVCAYSCLGPELNQSWLLKLSLAVWSVALLMALPVTLASDVVSGFCILPISNDEWVWPWIHIVACIVIFLILPLGLVQGWAIQRVKGRGSGPWANVLWVWFLFWWLHAIMIGLDALVRTENLSMHTCFAQQTLDFLLQSAEILGILHCLATPLLLALFCHRAT

>meuCXCR4 ENSMEUT00000006643 Scaffold 357344

SLFLQIQASDNNSSDGFGSGDYDGKEPCFRHENEDFNRIFLPTVYTFIFLTGIVGNGLVIIVMGYQKKLRSMTDKYRLHLSVADLLFVLTLPFWAVDAAASWYFGKFLCKAVHVIYTVNLYGSVLILAFISLDRYLAIVHATNSQRPRKLLAEKVVYLGVWLPALLLTVPDIIFANTGEANGRYFCDRVYPHDNWLISFRFQHILVGLVLPGLIILTCYCIIISKLSHSKGHQKRKALKTTVILILAFFACWLPYYIGISIDTFVLLEVIKQDCDFDTAVHKWISITEALAFFHCCLNPILYAFLGAKFKTSAQNALTSVSRGSSLKILSKGKRGGHSSVSTESESSSFHSS

>meuCXCR5 ENSMEUT00000004982 Scaffold 70457

FTILDFSNYSNYSNVFLPESWDDYLCPARAGKILNFKSVFVLVAYSAIFLLGMTGNALVLVILKSHRTARSSTETFLLHLAVADLLLVLTLPFAMTEGAVGWVLGAFLCKAVSALHKINFYCSSLLLACIAVDRYLAIVHAVHTYRHRRLLSVRIACGAVWLASVLYALPELLFVTVSKIGTNDLLCSFAGQGLAGSDARLTSRFLYHIGGFLIPLLVMAWCYTGVVRRLCQAQRRHQRQKAVKVAILVTGVFFFCWSPYNVVIFLDTLVMLGTIQKSCQLDDHLATAIITCEFLGLAHCCLNPVLYTFVGVKFRSDLIRLLGKLGCVGPAALQRFVPSWRKGSSSESENASSITTF

>meuCCR3 ENSMEUT00000013461 Scaffold 17826

MTSFMDTSEEMTTFDYDSAMPCQKMSIKTLGAKFLPPLYSFVFIVGLLGNIMVVLILTKYKRFKSMTNIYLLNLSISDLLFLFTLPFWIHYARQNDWVFGHTMCKILSGLYYLGLYSEIFFIVLLTIDRYLAVVYAVFALKARTVTFGIFTSIVTWGLSALAALPEFIFHESQQDMENHICSPRYPEYDEDKWKRFQALRMNILGLAIPLAIMIICYTGIIKKLLGSRNDKKYKAVRLIFVIMIVFFLFWTPYNLTLLLSAFQASFFKGDCERSKQLDIAMQVTEVIAYTHCCVNPVIYAFVGERFRKYLCHFVRRHIVAHLFYYNPFLPGEKLERVSSISPSTGVQELSAAF

>meuCCR4 ENSMEUT00000009969 GeneScaffold 8860

LETMETTLDYGNYYSSDLPSPCSKGGVRDFGKLFLPPLYSLVFLFGLLGNFMVVLVLLKYKRLKSMTDVYLFNLAISDLLFVFSLPFWVYYVADQWVLGTAFCKIISYMYLVGFYSGIFFIMLMSVDRYLAIVHAIFALRARTLTYGVITSLVIWSVAMLASLPVLLFSTSYTENNNTYCKEKYPGNSTTWKIMSSLEINILGLLIPLGIMLFCYTRIIKTLHRCKNDKKNKAVKMIFAVMVVFLLFWIPYNIVLFLEILVELEVFQDCTFGMQLDYALQATETLAFVHCCLNPVIYFFLGEKFRKYIKQLFKXXXXXXXXXXXXXXXXXXXXXXXSSSHTQSTMDHEIHDAL

>meuCCR7 ENSMEUT00000015024 GeneScaffold 3780

LVMLTYIYFKRLKTMTDIYLLNLALADILFLLTLPFWAVSAAKSWIFGPILCKVVYFTYKTSFFSGMLLLLCISVDRYFAIVQAVSAHRHRTRIVLISQISCGVVWVLALLFSTPELIYSNILKNGRCSLVTEDLVTFTTIIQVSQMVIGFLIPLLVMFSCYLIIIRTLLQARNFERNKAIKVIIAVVIVFIVFQLPYNGVVLVKTVAALNKTSTDCEHSKQLDIASDITYSLACLRCCLNPFLYAFIGVKFRQDLFKLLKTLGCLSQERLLHLSSCRQNRRFSMSMEAETTTTFSP

>meuCCR9 ENSMEUT00000014566 Scaffold 266820

SNFDYNFTISAEDYTVNDICMKTSVRQLARYFLPPLYWLVFVVGTLGNSLVIVMYWYCTRVKTMTDMFLLNLAIADLLFLFTLPFWAIDASDQWKFGTNMCKVINATYKINFYSYVLLIMCISIDRYIVIVQAMKAHRWRQKRLLYSKMVCSAIWVIAIILCIPEYLYSQSKVQYDTTVCTMVYPRDNNSLKAILSMLKFIMGFFIPVTVMASCYTIVTHTLMQAKKSSKNKALKVTILVLTVFVLSQFPYNIIILVQAIDAYRMFIIDCDISTYIDISFQVTQTIAYFHSCLNPVIYVFVGEKFRQDLVKTLKKLGCISQAQWVSFTRKEGTLRLSSMMQETTSGPLSF

>meuCCR10 ENSMEUT00000015886 GeneScaffold 8919

VTWGIHYSGESDDVYPSVTLPELCHKADVQSFSRTFQPSISLTVAAFGLAGNGLVLATHLAAHRTARSPTSAHLLQLALADLLLALTLPFAAAGALQGWTLGSILCRTVSGLYSASFHAGFLFLACISADRYVAIAQASPGGHRPPAPGKTHVVSAAIWLLSLLLALPSLLYGQDGQRDGQRRCRLVFPEGLTQAVKGASAVAQVILGFAVPLSVMVACYTLLGRTLLAARGPERRRALRVVVALVVAFVVLQLPYSLALLVDTTDLLTARERSCSSSKRKDMALLVTGGLALARCGLNPVLYAFLGLRFRQDLRRLLRGGCFRYRSSQRGRCPRRPRLSSCSAPTETHSLSLDNW

>meuCXCR7 ENSMEUT00000008252 Scaffold 129652

MDPFPFDYSEPGNFTDLNWTCTNGDCITVDTIMCPSATGRNILSYILSIFYIFIFVVGMIANSVVVWVNLQAKTTGYETHLYILNLAIADLCVVVTIPIWVVSLVQHNQWPMGELTCKVAHLIFSINLFGSIFFLTCMSVDRYLSVTYFANTSSYKKKVVRRLICILVWLLAICVSLPDAYYLKTITLAANNETYCRSIYPEHSIKEWLLGMELLSVVLGFIIPFSIIAVFYFLLARAISASGDQEKHSNQKIIFSYVIVFLICWLPYHAVLLLDIFSFLHFIPFSCQLENFLYTALHITQCLSLVHCCVNPVLYSFINRNYRYELMKAFIFKYSAKTGLTKLIDASRVSEAEYSALEQNAK

>meuCCRL1 ENSMEUT00000003569 Scaffold 45630

MDLEHNQSTDYYYEENEANGTQDYSQYEILCVKEDVRNFAKVFLPAFYTVAFIAGIAGNSTVVAIYAYYKKQKTKTDVYIMNLAVADLLLLVTLPFWAVNAVHGWVLGIPMCKVTSALFTINFASGMQFLACISMDRYSAITKAPGHQRVGRPCWIICVSVWLIAILLSIPQLVFNTVNDKKRCLPIFSHRLGTTIKASIQILEICIGFVLPFLIMGVCYSLTARTLIKIPNVKKSQALRVLLAVVAVFIVTQLPYNIVKFWQAIDIIYSLIIDCEMSKRMDVAIQITKSLALFHSCLNPILYAFMGSSFKMHITKMVKKYGYWRRQRQNPEDIPFDSEERTESMSTFSI

>oanCXCR1a XP_001514199.1 Supercontig 25198

MNHLVDLDELFSYSNVSDYSSYSPSLDDFLSTVGMSAPCRREPGAVMRPALAALYGLVFLFNVVGNSLVALVVLHNRPHRTVTDVYLLNLAGADLLFALTLPFWAAYQVTGWLFGTVLCKLVSVLQDLNFYSGVLLLACISVDRYLAIVHATRSAAHQRRWVVFVCLGLWILSFLLALPALVLRDVFTPPNSTPVCYENMGKDTARWRVILRLLQQTFGFLLPLLVMLFCYGRTLAVLFRARMGQKHRAMRVVLAVVLVFLLCWLPYNVILVIDSLMWVKVLTDSCGLRDHVDRALVVTPVLGYLHSCLNPIVYAFVGQKFRHSLLRILAARGLVSKDFLVRHRIPSSQSSSVANITTV

>oanCXCR1b XP_001520717.1 Supercontig 38449

MRPALAALYGLVFLFNVVGNSLVALVVLHNRPHRTVTDVYLLNLAGADLLFALTLPFWAAYQVTGWLFGTVLCKLVSVLQDLNFYSGVLLLACISVDRYLAIVHATRSAAHQRRWVVFVCLGLWILSFLLALPALVLRDVFTPHNSTPVCYENMGKDTARWRVILRLLQQTFGFLLPLLVMLFCYGRTLAVLFRARMGQKHRAMRVVLAVVLVFLLCWLPYNVILVIDSLMWIQVLADSCGLRDHVDRALVVTPVLGYLHSCLNPIVYAFVGQKFRHSLLRILAARGLVSKDFLVRHRVPSSHSSSSANTTTM

>oanCXCR4 XP_001510648.1 Ultracontig 387

MAHWKEHGLSSQRSWVPILALPPVSCVTLGKSLPSGFIDIHDNSSDGDWSGSGSGDYDNYKEPCYRQENADFNRIFLPTIYSIIFLTGIVGNLLVIIVMGYQKKLRSMTDKYRLHLSVADLLFVLTLPFWAVDAAVSWCFGIFLCKAIHVIYTVNLYGSVLILAFISLDRYLAIVHATNSQRPRKLLAEKVVYVGVWLPAVLLTIPDLIFAETSQLDERYICERFYPHDTWLISFRFQHIVVGLILPGLIILTCYCIIISKLSHSKGHQKRKALKTTVILILAFFACWLPYYIGISIDTFILLGVIKQGCAFQNTVHKWVSITEALAFFHCCLNPILYAFLGAKFKTSAQNALTSVSRGSSLKILSKGKRAGHSSVSTESESSSFHSS

>oanCXCR6a blast search Ultracontig 117

VVDSNLYTSMLLLTCITVDRFISVAQATKAHAYQSRRLTWGKITCICMWLISLAVTTPQFIYSTVSNQDKQVCLEFGNSDSRQVFLVALAAQMTIGFFLPLLAMIVCYSVIIKTLIRAKGFQKHKSLKIIFLVVAVFVLTQLPYNLVKLIVRAHWEYYTYPSFHYALVVTESIAYLRACLNPVLYAFVGLKFRNNFWKLLRDLRCPHQLQITSNWRTTDEDASKTFTASNLAESPPHISCKSGLLACWVFI

>oanCXCL6b blast search Ultracontig 117

CGGFNLYTSMLLLTCITVDRFISVAQATKAHAYQSRRLTWGKITCICMWLISLAVTTPQFIYSTVSNQDKQVCLEFGNSDSRQVFLVALAAQMTIGFFLPLLAMIVCYSVIIKTLIRAKGFQKHKSLKIIFLVVAVFVLTQLPYNLVKLIVRAHWEYYTYPSFHYALVVTESIAYLRACLNPVLYAFVGLKFRNNFWKLLRDLRCPHQLQITSNWRTTDEDASKTFTASNLAEVTTTYQL

>oanCCR1 XP_001513774.1 Ultracontig 117

MNISTVETDYDSATELIYDLAEPCKKTDVKALATRFLPPLYSLVFIVGVVGNFMVVLILVKYKRLITMTNIYLLSLAFSDLLFLFTFPFWIHSELKGDWIFGNGMCKLLSCLHSVGLFSGIFIIILLTIDRYLAVVHAVFALKVRTVTVSFVSSAVAWILAGFTALPDFIFQKTQTKNLRRTCSLYFPHDTAETWKYLQALKQNILGFALPLLVMVVCYTGIIKRLLRQRGERKVVVVRLIFVIMLIFFIFWTPYNLVHLIFAFQEFIFENENECERSNQLDIAFQVTEVIAFTHCCANPVIYAFVGERFRKYLRHFFQRHIAANLCKLVPFSPGKKRSGXXXX

>oanCCR2 XP_001513811.1 Ultracontig 117

MDGPLTTTQFFYDYGGEPCQKIDVQLTGAQLLPPLYSLVFIFGFVGNGLVVLILKKYKKLKTMTDIYLLNLAISDLLFLFTLPFWAYYAADQWLLGDAACKIFSGLYYLGFFSGIFFIILLTVDRYLAIVHAVFALKARTVSCGVVTSVCTWAVALLASVPGFLFNRSQKAEDRYVCSTYFPMGQDLAWKIFQTLQMNILGLAVPLLVMIVCYTGIIKTLLRCRNEKKKHKAVRLIFLIMLIYFLFWAPYNLVLLLNTFQGFFGLNNCDSSNRLDRAMQVTETLGMTHCCINPVIYAFVGEKFRRYVSLFFQKHIAKCFFKSCPVFYSETADRTSSTYTASTGEPEFSAGL

>oanCCR3 XP_001513794.1 Ultracontig 117

MENSTVDMDYDATTEFDYGTSAPCEKYNVKALATQLLPPLYSLVFIVGLVGNVVVALILIKYKRLRIVTNIYLLNLAFSDLLFLFTLPFWIHTEVKSEWVFGNSMCKFVSGLYYMGLYSEIFFIVLLTVDRYLAIVHAVFALQVRTVNFGIMTSAVTWGLAGLAALPSFVFSTSQRSLENHVCSPVYPDAKAENWKHFRTLKMNLLGLIFPMIVMVVCYAGIIKKLLKCRNEKKYKAVRLIFVIMIVFFLFWTPYNLVLLLNNFQMYFFEGECEKSKQLDIAMQVTEVITFIHCCANPVIYAFVGERFQKYLCHFFRRRMAVKLGKHIPLFPCEKLERVSSVTPSTGEQDFSFIFKETEGILVAATFCYAGEEGQNTS

>oanCCR4 XP_001514912.1 Supercontig 1521

MSTVDPILSTTEEEPYSSSDYSYDNAPRPCSKGGVKAFGGLFLPPIYSQVFLLGLLGNSTVVLVLFKYKRLRSMTDVYLLNLAISDLLFVFSLPFWAYYAADQWIFGLGLCKIISWIYLVGFYSGILFIMLMSIDRYLAIVHAVFSLKARTFSYGVIASSVIWLVAILASFPTLLFSESFWEEGQISCKSKFPNNSTTWKVLSSLEINVLGLLIPLGVMLFCYSLIIKTLQHCKNDKKNKAVRMIFVVMIVFLVFWIPYNVVLFLEALVELELLKDCTFEKHLDYALQATETLAFVHCCLNPVIYFFLGEKFRKYIKQLFKTCKIPLALCKYCGLLQAYYPETPSSSHTQSTMDHEINNAL

>oanCCR5 XP_001517287.1 Ultracontig 117

METTTSFMYDNDGDPCTKNEVKFTGAQLLPPLYSLVFISGVVGNGLVVLILKKYKKLKTMTDIYLLNLAISDLLFLFTLPFWAYYAADQWLLGDAACKIFSGLYYLGFFSGIFFIILLTVDRYLAIVHAVFALKARTVSCGVVTSVCTWAVALLASVPGFLFNRSQKAEDRYVCSTYFPMGQDLAWKTFQTLQMNILGLAVPLLVMIVCYTGIIKTLLRCRNEKKKHKAVRLIFLIMIIYFLFWAPYNLVLLLNTFQSSFGLNNCDSSNRLDQAMQVTETLGMTHCCINPVIYAFVGEKFRRYVSLFFQKHIAKHFCKSCPMFHSETADRTNSTCTRSSGEQEFSAGL

>oanCCR6 XP_001507022.1 Ultracontig 413

MKENQLEARGRIDGHSRLKREEWEEAGREQGQVNLSIFEEYSDYTEATSTLDYEDALPCSMQEVRNFTKTFLPVAYSLISIFGLVGNVLVVVTFAFYKKGKSMTDVYLLNMAITDILFVLTLPFWAVNHATESWIFDNFICKLTSGIYAINFNCGMLFLACISMDRYIAIVQATKSFRLRAKSFKFRMRTLAYSKVICSVVWMFSILISSATFIVSQKYQMQGKYICEPKYQVVSEAITWKLLILGLQLLFGFFIPLLFMIFCYTFIVKALVKAQNSKRRKVIRVIIVVVVIFLIFQVPYNMVLLINAINLGKLNRTCKSEQQRAYARNITQVLAFLHSCLNPVLYAFIGQNFRNYFMKIMKDLWCVDRKHKPGTPSCPRKYSDTCISRQTSEIAESDNPSSFTM

>oanCCR9 XP_001513641.1 Ultracontig 117

MYSSVAISCKDGYDTGVIRVATPKPPTIGEVGGHPFFTVNGPFAVLWGIVSLTSMNGSELGKSMDSMIIMIMMVMADSTKLISWSSFLSAIDGDDSEFTTSLDDYYYNITENVFCEKSNVRLFAKNFIPPLYWTVFTAGILGNSLVILVYWYCSQMKTMTDMFLLNLAIADLLFLFTLPFWAVAAADQWKFQNTTCKLVNSMYQMNFYSSMLLLTCIGMDRHIVIVQATRAFSWKQKRVVYSKVICLCIWMVATALCIPELTFSQATETNDVMTCTVVYPDDGTTMLKASFMMLRFILGFFLPLLVMVCCYATIIHTLLQTKRSCKHRAFKVAIAVLIVFILSQFPFNSLLLVKTIATFKPSEDRAFISSCALSTKLDIGFQITQTLAFFHSCLNPVVYVFVGEKFRQDLRKVLKKLGCIRKTSTWREGKGLSSTLLETTSGALSL

>oanCX3CR1 XP_001518678.1 Supercontig 20158

MTEKTLEMTTSYEYDDLAEMCELGDILDFGRVFLPVLYALVFAFGLVGNLLVVFAVTNGGKKKSITDVYLLNLAFSDLLLVISLPFWIHYVVGEQVLGNILCKSASALFFIGFFGGMFFVTVISIDRFLAIVQAAGSIHSRTVQHGVSTSFSVWTVAILAAVPQFMYTEQVGDECVGNYPEVLQHMWPVLRNLEANLFGFLFPFLIMSYCYLRILKTLLFCNNRKKVRAAKLILMVVAVFFLFWTPYNILVFLETLNHFKFFPSCTLKRHLRFGLSVTETIALIHCCLNPFIYAFAGKKFRRYLYVLYVKCRAVFCGLDPRLRIPKEEVSLSKRESVISSNFTSFSFDHEPSGLL

>oanXCR1 XP_001513749.1 Ultracontig 117

MAGVLLYAKRNGEPSKFFEEEIYLQCGRQADIAYSRSSTAWCLDLGSGKWEQGSNPKSGTYLFYNLDPESYLCDSEEVFGFVSINISVLYCLVFFLSLIGNSLVLCVLIRYENLESITNTFIFNLCLSDLVFSCLLPFWTTAHHLGWIFGDVLCKILNLLFSLSLYSSIIFLTVMTIHRYLSVVNPLSTLRTHTLRYRLLVSLAIWVTSLAAAIPDAIFHKVMTGSEKAVNPEFCDYFEPKWLLVSVCQHNVFFLFSMVIILFCYIEILRTLVRSRSRRRHRTVRLIFAIVMAYFLSWGPYNVLVFLETLVRFHIIPSCSLYKQLEYALHISREIAFSHCCFNPVLYVFVGIKFRRHLKSLCYRPHLSVPTNPSLPLLSLPIKERLLKVGFGDLHQSPHLNQFSLYPLSPIGFVPLQLTI

>oanCXCR7 XP_001508356.1 Ultracontig 197

MDFASIFDYPEPGNFNDTNFTCTSGDCILVDTVLCPNTLNKSALLYTLSIFYIFIFVIGLIANSVVVWVNLQAKTTGYETHRYILNLAIADLCVVITLPIWVVSLIQHNQWPLGELTCKITHLIFSINLFGSIFFLTCMSVDRYLSITYFTNSSSRTKKVTRCVICTFVWLLAICVSLPDTYYLKTITSATSNETYCRSFYPEDSVKEWLIGMELVSVILGFAIPFAVIAVFYFLLARTITTSNDQEKHSNRKIIFSYVVVFLVCWLPYHAVVLLDIFSILHFIPFSCQMENFLYAALHVTQCLSLVHCCVNPVLYSFINRNYRYELMKAFIFKYSAKTGLTKLIDASRVSEAEYSALEQNTK

>oanCCRL1 ENSOANT00000010820 Ultracontig 117

RSLFYSPNILAMDLAHNQSSEYYYEEGEMNATYDYGLFETICLKEEVRNFAKSFLPAFYTVAFIVGLAGNSAVLVVYSFYKKQKSKTDVYIMNLAVADLLLLFTLPFWAVNAVQGWVLGQTMCKVTSALYTINFVSGMQFLACISVDRYSAVTNAPRLEKIGKTCWVICFCVWIAAVLLSVPELIFNTVRKNKDRYRCLPIFPQHLGISMKASIQLLEISLGFAVPFLIMGVCYFITARTLIRTPNIKKSQPLKVLLAVVVVFIVTQLPYNIVKFWRAMDIIYLLITDCGMSKRIDVAIQITESVALFHSCLNPILYAFMGASFKTHI

>oanCCRL2 XP_001513845.1 Ultracontig 117

MDNNSSEYIYDVFIEEDFIKDEMEQCNKYDGQVLATQFMPRLYLLVFILGLVGNTLILVILIKYKRLKLTVHLCLLNLAISNLFFLLTLGFGALTSRPGWALGYAMCQLISGIYSVGLFSQSFFIMFLAVQKYLTVVHRLTFHLKPKAGHWGIITSALIWGLATLASLPDFLLRQSPLKDESDTCRFSKPHYALSDEKSWMHFLTLRMSILGLLFPLLVAIFCSVGLIKVLCRHRIEQKVTLTLTIVMVFFLFWTPYNLVLFLSTFSKYFSLSDCESSYQLDRAIQVTKIIADTHCCVNPVICVSLDESFRKYLCRLFIRSNNTPGHPPEGPEQAHSTSREQLEHTSTC

>ggaCXCR2 CAJ00345.1 Chr Un_random

MGTFYADELLDILYNYTSDYGNYSLVLPDIDVSSSPCRNEGSVANKYLVAFIYCLVFLLSMVGNGLVVLVVTSGHINRSVTDVYLLNLAVADLLFALSLPLWAVYWAHEWVFGTVMCKAISVLQESNFYSGILLLACISVDRYLAIVYATRAATEKRHWVKFVCVGIWVFSVLLSLPVLLFREAFVSDRNGTVCYERIGNENTTKWRVVLRVLPQTFGFALPLLVMLFCYGVTVHTLLQTKNVQKQRAMKVILAVVLVFLVCWLPYNITLVSDTLMRTRAITETCERRKHIDTALSITQVLGFAHSCINPIIYAFIGQKFRNSFLKILAQRGFISKDAVARYGRTSYTSTSGNTSTTL

>ggaCXCR4 NP_989948.1 Chr 7

MDGLDLSSGILIEFADNGSEEIGSADYGDYGEPCFQHENADFNRIFLPTIYSIIFLTGIIGNGLVIIVMGYQKKQRSMTDKYRLHLSVADLLFVITLPFWSVDAAISWYFGNVLCKAVHVIYTVNLYSSVLILAFISLDRYLAIVHATNSQRPRKLLAEKIVYVGVWLPAVLLTVPDIIFASTSEVEGRYLCDRMYPHDNWLISFRFQHILVGLVLPGLIILTCYCIIISKLSHSKGHQKRKALKTTVILILTFFACWLPYYIGISIDTFILLGVIRHRCSLDTIVHKWISITEALAFFHCCLNPILYAFLGAKFKTSAQNALTSVSRGSSLKILSKSKRGGHSSVSTESESSSFHSS

>ggaCXCR5 NP_001026083.1 Chr 24

MIRECAAPQWGGFGGDWVPLFRSCPPVTRLSVPQSLVELSGYYEAENTTPSLEGYFCFNPSSLWLANQRDPFRKVFIPLAYLLMFVLGTVQNALVLVILERFKRSRTTTENFLFHLTLANLALLLTFPFSVVESLAGWVFGTFLCKILSAVHKINFYLHEHAAGLHRVDRYLAIVYAIHTYRKARARSIHLTCTAIWLSSLLLTLPDLIFMEVWTDESNRSICYFPEAGIHGNNVWLATRFLYHSVGFFMPLLVMCYCYMAIVRTLCQSQRLQRQKAVRVAILVTGVFLLCWSPYHIVIFLNTLTKLEAFAKDCLLEDHLDTAIMVTEAIGFTHCCLNPIIYAFIGVKFRNDFFRILHELGCISQETLQEILEVTRKGCGIESDNTTSISTF

>ggaCCR2 NP_001039300.1 Chr 2

MENYTDLGDMPTTTFDYGDTAPCMGTEEKHFAANFLPPLYSLVVIFGFIGNILVVLILVKYKKLKSMTDIYLLNLAISDLLFVFSLPFWAYYAAHDWIFGDALCRILSGVYLLGFYSGIFFIILLTIDRYLAIVHAVFALKARTVTYGILTSIVTWAVALFASVPGIVFHKTQQENTQCTCSFHYPSDALINWQHSYILKMNILGLIIPMIIMIFCYSQILRTLFGCRNEKKQKAVRLIFVIMIFYFIFWTPFHVASFVHTFQTSFFSPDCDSQSRLEKTIQVTETISMVHCCINPVIYAFVGEKFRKYLHMFFRKHVATHLCKKCPSLYREKLERVSSTFTASTAEHDISTGL

>ggaCCR4 XP_426017.2 Chr 2

MSSSSTESLEADTTTFYDFIDNYNDAPQPCSKENFKRFAASFFPVLYTLVFLIGLIGNTLVIVVLFKYKRLKSMTDVYLLNLAISDLLFVLSLPFWSYFMIDQWVFGTPWCKIISWIYLVGFYSGIFFIMLMSIDRYLAIVRAVFSMKARTAFHGLIASLTVWLVALLASVPELVFRESFVEQNYTTCKLRYPSNYLTWKLFYTLEINILGLLLPLIVMAFCYSMIIKTLLHCRNEKKNKAVRMIFAVMIVFFFFWTPYNIVILLQLLEATGVIRNCQASRNLDYASQITESLGLFHCCLNPVIYFFMGEKFKKYLKMLFKNWQLPGDICKWCGLHITYHTESTGSFHTQSTGDQEAL

>ggaCCR5 NP_001039299.1 Chr 2

QHTMENYTDLGDMDVTTTFDYGDTAPCMGTEEKHFAANFLPPLYSLVVIFGFIGNILVVLILVKYKKLKSMTDIYLLNLAISDLLFIFSLPFWAYYAAHDWIFGDALCRILSGVYLLGFYSGIFFIILLTVDRYLAIVHAVFALKARTVTYGILTSIVTWAVALFASVPGIVFHKTQQEHTRYTCSAHYPQEQRDEWKQFLALKMNILGLVIPMIIMICSYTQIIKTLLQCRNEKKNKAVRLIFIIMIVYFFFWAPYNICILLRDFQDSFSITSCEISGQLQKATQVTETISMIHCCINPVIYAFAGEKFRKYLRSFFRKQIASHFSKYCPVFYADTVERASSTYTQSTGEQEVSAAL

>ggaCCR6 NP_001107553.1 Chr 3

MSTTVFGTTEFFDTDYASLISTVCSKSEVRSFTKVFLPVAYSLICIVGLVGNIFVVMTFALYERTKSMTDVYLFNMAIADILFVLTLPLWAVNYAADKWIFGNFICKMAKGIYAINFSCGMLLLAFISVDRYIAIVQATKSFKLRARTLAYSKLICLAVWASAILISSSSFLYSESYDFATNETQICDHRFDKTSDSIVLKSLLLCLQVGFGFFIPFVFMIFCYAFIVKSLQQAQNSKRNKAINVIVLIVVVFLVCQVPYNTVLLMAVANMGKAEKSCDSDNIMAYAKYTTETIAFLHCCLNPVLYAFIGVKFRSYFVKIMKDLWCRRYKKYNKRSSRINSDIYLSRQTSEILTDNASSFTI

>ggaCCR7 XP_425875.2 Chr 27

MRQDWRRVGAGGARGGELQGEVVLPPKNAQCGRSSAAPGGTAAVRPAWDGRESDPGGVLTMWAGGCSPPPFHTASSSSLSLRLHLPNNGGQQSCRELCRVQNATAKHAAAAVLQAARGNPNLTTNTVPSSSPGKQLRVTIAFSLPLIFQFCAGNNVTDDYDANTTIDYNMFEMMCEKKEVRDFRAAFLPAMYSLICFTGLLGNGLVMLTYIYFKRLKTMTDIYLLNLALADILFLLTLPFWATSAATFWCFGEFACKAVYCICKMSFFSGMLLLLSISIDRYFAIVQAASAHRFRPRMIFISKVTCILIWLLAFVLSIPELVHSGVNNYDSHPRCSIIASDLQTFSTGIKVSQMVFGFLVPLVVMSVCYLIIIKTLLQARNFEKNKAIKVIIAVVIVFVVFQLPYNGVMLAKTISVFNNTSSCDESKKLDMADDVTYTLACFRCCLNPFLYAFIGVKFRNDLFKLLKELGCLSQQRLWQLSSCRESKRFSFAMETETTTTFSP

>ggaCCR8a NP_001026162.1 Chr 2

MDGDLRSLLAGGKQEDLLADFHFSPTVNSSASYDNMYYPELATDCEFESIPAFASSFFPVLYSILFVIGLMGNALVVWVLTAFKKIRAMTDVYLLNLAISDLVFVFSLPFLAQYSLVSQWTFGNAMCKIVSSAYFIGFYSSAFFITIMSIDRYLAIVHSVYALKVRTTKHGIIASLALWAVAILASVPGLVFFREVDEDNRTQCIPHYPGSGNSWKVFSNSEVNILGWLFPVSILIFCYHNILRNLQRCHTQNKYKAMKLVFIVVIVFFLFWTPINIMLLLDSLRSLHIIDDCQNSQRLDLALELAETLSLVHCCLNPIIYAFVGEKFKKYLCEAFGKYAHFLLICKGHSAFNRRNTDRRTSMYTASSQSSFVGSVL

>ggaCCR8b NP_001074363.1 Chr 2

MEQRKKPTGRHTRALYLWFFPSQESKMNPTDLFLSTTEYDYGYDENTAPCNEGNSFPRFKSLFLPILYCLVFVFCLLGNSLVLWILLTRKRLMTMTDICLLNLAASDLLFIVPLPFQAYYASDQWVFGNALCKIMAGIYYTGFYSSIFFITLMSIDRYIAIVHAVYAMKIRTASCGTMISLVLWLVAGLASVPNIVFNQQLEIEQSVQCVPVYPPGNNIWKVTTQFAANILGLLIPFSILIHCYAQILRNLRKCKNQNKIKAIKMIFIIVIVFFLFWTPFNVVLFLDSLQSLLIIDNCQASSQITLALQLTETISFIHCCLNPVIYAFAGVTFKAHLKRLLQPCARILWSPTRGSGVTQSSLVLSQISGCSDSAGVL

>ggaCCR9 NP_001039305.1 Chr 2

MTSLDYYRNDSAGLSVIGNPINDTELMCDRRQVWQFARAFLPVFFWLIFFVGTVGNALVVLIYCKYRFRRSMMDRYLLHLAVADLLLLFTLPFWATAASSGWIFRNFMCKVVNSMYKINFYGCILFLTCISFDRYLTIVQATKAKSSKQRRILRSKVVCFAVWLASVSLCLPEIMYSQSKQIGAVTVCKMTYPPNIGMAFRVAVLVLKVTIGFFLPLLVMVICYTLIIHTLLQAKRCQKHKSLKIITMIITAFLLSQFPYNIVLLIKTINMYTGAVYSCQTINGLDIGLQVTQSIAFLHSCLNPFLYVFAGERFRMALARMVQSTGRYWLGGQDQCSSLGDSQEHSSNWSFAMLGRRRVRNSLTLSTNLASSVVPASCQVFV

>ggaCX3CR1 XP_418820.1 Chr 2

MTEAPPEVTTEYVFYESALACDESDIQAFGKIFLPLFYIAVFALGLAGNVMVVLAIVKEGSKKSITDIYLMNLAVSDLLFVISLPFWASNTVRGWTLGTIPCKVVSSLYYIGFFGGMFFITVISIDRYLAIVRATYSMRSRTIKHSLLITCGVWATAVLVSVPHFVFSQMFENDCIPVLPQELMNIWPVFCNVELNTAGFFIPVCIICYCYCGIIKTLLYCKNQKKARAIKLTLAVVIVFFLFWTPYNVLIFLETLRHYELFISCNQIKSLDYAMHLTETIAFSHCCLNPLIYAFAGEKFRKYLHRVCFKYCPCLCFCGPCNHYDVRPSVSYAESMVNSNITLNTSDQDGTVFL

>ggaXCR1 NP_001039303.1 Chr 2

MDEEQYPSGWDDNYSFEYVLNESNVCEMGNYFIFYTHFTTVLYTLAFLLSLLGNTLVLWILFKYENLTSLTNIFIMNLCISDLVFSCMLPFWAVDQTFGWIFGEFLCKAVNAIFSIGYYSGVFFLTLMTILRYLSVVSPLSTLRSQTQYCGSLVCLLVWTCSILIVVPEMIHTTVVETLEEVSTCDYDDWKWKKVDIYQRNILFLISFGIIIFCYINILIILLRTRSRRKHRTVKLILVIVVAFFLSWAPYNILSFLITFPPPTCQYEKDTLLAFHISRKIAFSHCCLNPVLYVFAGVKFKSHLFRLCGQYLPCCDEVSRIGSQAKFHYEDASIY

>ggaCXCR7 NP_001076831.1 Chr 7

MSALDLTSIVDFLEKANLTEINWMCNNSDCITVDATTCPGTLNKSALLYTLSFFCIFIFVIGLVANSVVVWVNLQAKMTGYETHLYIFNLAVADLCVVITLPVWVVSLVQHNQWHMGEITCKITHLIFSINLYGSIFFLACMSVDRYLSVAYFTNSSNRKKKIVRRCICILVWLLAFSASLPDTYFLKTVSTSNETYCRPAYPEESFKEWLIGMELISVVLGFLIPFPVIALFYFLLAKTISASSDQERKSSGKIIFSYVVVFLVCWLPYHTTVLLDIFYSLHFIPFSCQMENFLYATLHITQCFSLVHCCVNPILYSFINRNYRYELMKAFIFKYSAKTGLTKLIDASRVSEAEYSALEQNTK

>ggaCCBP2 XP_418499.1 Chr 2

MGTARRGGATLPATIAWDGHLGSLRRGITTGSHLPYTASKLATTAAALLDGQDYTANSSDYPYEYLNEEDYILYGVCTKEEVVSFSKAFLPAFYTVVFLIGFTGNVLLFTVLMYISKKKKMAEVYLLNLVVSDFLLLLTLPFWALFISQWVTWDLLCPVLNAMYIMNFYSGIFFVSCMSLDTYLQIVHACSPHSSVTRKKSFLLLLMLWVLSILLSIPDALFSSTKEMHNKTIVCTHDYGQKHLFWKVVFQVTQNILGFLLPFFFMVFCYSRSMCVLTTSRVPGSRTALRFVFILVAVFFVLWFPYNVVLILHSLQFVGLIQSCERSRQLDYAIQITESLSFVHCCLNPVLYAFVKKRFRLYLQKIPQAFCRKSTFDIQLSETSCSCSRYIAQIEMLSITNT

>ggaCCRL1 ENSGALT00000019133 Chr 2

MGWVVNNSTDYWIEDDEHYLNSDVDYNTYEFLCEKEDVRKFMKVFLPVFYALTFTVGVAGNSLVVAIYAYCKKPKTKTDVYIMHLAIADLLLLFTLPFWAANAVQGWELGNSMCKLTSSLYTMNFSSSMLFLACISLDRYRATSESQRHRRAGKHCSVTCICVWLSAVLLSIPELIFNQVITHNNRNECLPIFPTNMETLLKATIQILEVILEFLLPFLVMVTCYSVTARAIFRSANAKKSRPFMVLLAVVAAFIITQLPYNIIKFWRAIDSIYLLITDCEASKTIDVALQVTKSIALFHTCLNPILYAFLGASFKMRVMKIAKNYGYWRRQQQHEVPEEISMNSEARTEETISFTI

>tguCXCR4 XP_002198314.1 Chr 7

MALSMDSSLDSLDLSSGLLIELSENGTDEIGSGDYGDYKEPCYQNENADFNRIFLPTIYSIIFLTGIIGNGLVIIVMGYQKKQRSMTDKYRLHLSVADLLFVITLPFWSVDAAIGWYFGNVLCKAVHVIYTVNLYSSVLILAFISLDRYLAIVHATNSQRPRKLLAEKVVYVGVWLPALLLTVPDLIFASTSEIERKYLCDRMYPHENWLISFRFQHILVGLVLPGLIILTCYCIIISKLSHSKGHQKRKALKTTVILILAFFACWLPYYIGISIDTFILLGVIRRRCSLETIVHKWISITEALAFFHCCLNPILYAFLGAKFKTSAQNALTSVSRGSSLKILSKGKRAGHSSVSTESESSSFHSS

>tguCXCR5 ENSTGUT00000000021 Chr 24_random

FRKIFMPLIYLLMFVLGTLGNALVLVILERFKRSRTTTENFLFHLTLANLALLLTFPFSVVESLAGWVFGKFLCKILSAVHKINFYCSSMLLGCIAVDRYLAIVYAIHTYRKRRAHSIHLTCMAVWLCSLLLTLPDLIFMEVWTDDGNRSICYFPEVGIDGNNAWLATCFLYHTVGFFVPLLVMCYCYTAIIRALCQSQRLQRQKAVRVAILVTGIFLLCWSPYHIVIFLNTLTKLEAFTKNCLLEDQLDTAIMVTEAIGFTHCCLNPILYAFIGVKFRNDFFRILQELGCISQETLQEILEVTRKGSGIESDNTTSISTF

>tguCCR2 XP_002196634.1 Chr 2

MGNETTDYTDPSVTTEFDYGDSTPCMGREEKHFAANFLPPLYSLVVIFGLTGNMLVVLILVKYKRLKSMTDIYLLNLAISDLLFVFSLPFWAYYAVHDWIFGEALCRILSGVYFLGFYSGIFFIILLTLDRYLAIVHAVFALKARTVTYGVLTSVVTWALAVLISVPGVVFHKTQKESSGYTCSTHYPSDSTINWKYSFILKMNILGLIVPMLIMIFSYSQILKILLRSKNEKKQKAVRLIFVIMIFYFIFWTPFHISSFLHTFQDSFFITDCELKGQLEKAIQVTETISMIHCCINPVIYVFVGEKFRAYLRSFFRKHVAPHLCKKCPSLYHEKLERASSTFTQSTAEHDISTGL

>tguCCR4 XP_002196746.1 Chr 2

MSSSSTESLEVELSTFYDYYDSYYDAPKLCSKEGVRRFAASFLPVLYSLVFLVGLAGNILVIVVLFKYKRLKSMTDVYLLNLAISDLLFVLSLPFWSYFTVDQWVFGTPWCKIISWIYLVGFYSGIFFIMLMSIDRYLAIVRAVLSLKARTTFHGLITSLVVWLVALSASVPELVFRESFNEHNFTTCKPRFPGNFTTWKLFSTLEVNILGLLIPFIIMTFCYSMIIKTLVHCRNDKKNKAVKMIFVVMIVFFFFWTPYNIVIFLQLLEFMGVIKDCQVSRSLDYAFQVTEILGLFHCCLNPVIYFFMGEKFKKYLKMLFKNWQLPGYFCKWCGVHTTYPAESTSSFHTQSTGDQDAL

>tguCCR5 XP_002198821.1 Chr 2

MGNETTDYTDWPLTTEFDYSDSTPCPATEEKHFAANFLPPLYSLVVIFGLTGNMLVVLILVKYKRLKSMTDIYLLNLAISDLLFVFSLPFWAYYAVHDWIFGEALCRILSGVYLLGFYSGIFFIILLTLDRYLAIVHAVFALKARTVTYGVLTSVVTWALAVLISVPGVVFHKTQKESSGYTCGAHYPSEQRNTWKQFLTLNMNILGLLIPMLIMICSYTQIIKTLLQCRNEKKHKAVRLIFIIMIIYFFFWAPYNICILLRDFQGAFSISTCEGNGQLHKAIQVTETISMIHCCINPVIYAFAGEKFRKYLRSFFRKQIAVHLSKCCPVFYADTAERASSTYTQSTGEQEVSAAL

>tguCCR6 XP_002189252.1 Chr 3

MTQILEVADPNTTDLHSTDYPYYSDYVNLITSPCSKQEVRNFTKAFLPVAYSLICIIGLFGNIFVVMTFALYERAKSMTDVYLFNMAIADILFVLTLPLWAMNYATDEWIFGDFICKMTRGIYAINFSCGMLLLAFISVDRYIAIVQATKSFKLRARTLAHSKLICLAVWISSILISSPSFLYSESYSFSINETKEICDHRFGRISESTMLKSLLLWLQVAFGFFIPFIFMIFCYTFIVKSLQQAQNSKRNKAIRVIVLIVAVFLICQVPYNIVLLITAVNMGKIDKSCDNDKIMAYAKYTTEAIAFLHCCVNPVLYAFIGVKFRSYFVKLMKDLWCKRHKKDNKRNSRTNSDTYHSRQTSEILTDNGSSFTI

>tguCCR7 XP_002194005.1 Chr 27

MPGLNDMSGYQRSNVKGPVEVQGKQLKVTLVFSLPLVFQLCAGDNVTDYYDSNSTIDYSMFESLCEKEEVRNFRAAFLPAMYSLICFLGLLGNGLVMLTYIYFKRLKTMTDVYLLNLALADILFLLTLPFWATSATTHWLFGSFACKAVYCICKMSFFSGMLLLLSISIDRYFAIVQAASAHRLRPRMIFISKVTCILIWLLAFILSTPELVHSGVNNMDSYPRCSIIANDLQTFSTGIKVSQMVFGFLIPLLVMSFCYLIIIKTLLQARNFEKNKAIKVIIAVVIVFVVFQLPYNSVMLAKTISAFNQTSSCEESKKLDVADDVTYTLACFRCCLNPFLYAFIGVKFRTDLFKLLKELGCLSQERLWQLTSCRDSKRTSFAMETETTTTFSP

>tguCCR8a XP_002196778.1 Chr 2

MEQNLTDLLGSSGSEDMLMSYTSPTPNSSAAYEYAFDYSELHIICHPEGIPEFASTLFPVLYSILFVAGLVGNALVVWILTVFMKIKTMTDVYLLNLTLSDLLLVFSLPFLVQYSVVSQWTFGNALCKIISSVYFIGFYSNVFFITIMSIDRYLAIVHSLHVQGIRTAAIGFITSLVVWVVAILASLPDLLFFQEVNDNNQIKCLPHYPSGSNGWKTFSNFEVNILGWLIPVFVLIFCYHSILKNLQKCHTKNKYKAIKLVFIVVILFFLSWTPVNIVLFLDSLRNMHIINDCQTSQRLDLAVELTEALSYVHCCLNPVIYAFVGEKFKKHLCDALKKSACFLSNCKGYGAFSGHSLDKHSSLHTKSSQLSSVGTVL

>tguCCR8b XP_002196658.1 Chr 2

MPAFSSIWASLQPQEPAAAEHSTALKAAPARAPAQVLLEHTLSAEEEEEEEEEEGTEAKPEVSEMNPTSQFLGTTEYDYGYDENTAPCNEGNNFLRFKSFFLPILYCLVFVFCLLGNSLVLWVLLTRKKLTTMTDICLLNLAASDLLFVLPLPFQAHYASDQWVFGNAMCKIMAGIYYTGFYSSIFFITLMSVDRYIAIVHAVYAMRIRTATCGIIISLILWLVAGLASVPNILFSQELEIEQALQCVPKYPPGDNTWKVASQFAANILGLLIPFSILFCCYTQILKNLQKCKNRNKVKAIKMIFIIVIVFFLFWTPFNIALFLDSLQSLHIINDCKASSQIALALQLTETISFIHCCLNPIIYAFAGVTFKAHLKGLLQSCGRVLSSPAGGAGAGQSISVPTQLSGWSDSAGVM

>tguCCR8c XP_002200128.1 Chr Un

IPEFASTYYLLGNFLFPVLYSILFVAGLVGNALVVWILTVFMKIKTMTDVYLLNLTLSDLLLVFSLPFLVQYSVVSQWTFGNALCKIISSVYFIGFYSNVFFITIMSIDRYLAIVHSLHVQGIRTAAIGFITSLVVWVVAILASLPDLLFSQEVNDNNQIKCLPHYPSGSNGWKTFSNFEVNILGWLIPVFVLIFCYHSILKNLQKCHTKNKYKAIKLVFIVVILFFLSWTPVNIVLFLDSLRNMHIINDCQTSQRLDLAVELTEALSYVHCCLNPVIYAFVGEKFKKHLCDAFKKSACFLSNCKGYGAFSGHSLDKHSSLHTKSSQLSSVGTVL

>tguCCR9 XP_002196709.1 Chr 2

MNMGHFPMEEVWLHPLLLWFLQGDLSVTHPGKSSLDYYRNGSIVLSLCEDPANSTDFMCDKRQVRQFAQAFLPVFFWLIFAVGTVGNTLVVLVYCKYHFRRSMMDLYLLHLAIADLLLLFTFPFWAKAASDGWIFKDFMCKVVNSMYKINFYGCSLLLTCISFDRYITIVQAMKARTCKRRWLLRSRLMCLAVWLTSVSLCIPELIYSQSTEVGDVTVCKIMYPPNISVIFRVTVLALKVIIGFFLPLLVMVICYALITNTLLQAKRFQKQKSLKIITMILTAFLLSQFPYNIVLLVKAIDTYTGVVHSCQAANQLDIGLQVTQIIAFLHSCLNPFLYVFAGERFRMALGRMMQSCGCCWSRGQEHFSACDSQEHSSNWSFAMLGRRRVRNSLILNTHWTSSVMSPPCKVIL

>tguCX3CR1 XP_002196764.1 Chr 2

MTEAYAETTAEYAYDEHAFSCNKTDIQEFGKIFLPLFYILVFALGLTGNLMVVFAIVKGNKKSITDIYLLNLAVSDLLFVISLPFWASNTVRGWTLGTIACKAVSSLYYIGFFGGMFFITVISVDRYLAIVRATYSLKSRTIRHGFLVTCGVWAIAVLVSVPHFVFSQLIENDCIAVYPEKLENIWPVFRNVELNTIGFFIPVCIIFYCYCGIIKTLLSCKNQKKARAIKLILIVVVVFFLFWSPYNVLIFLDTLNHYELFTNCNQIKTLDYAMHLTETIAFSHCCLNPLIYAFAGEKFRKYLYHVCFKYCPFLCFCGPCSHYQVSHSVSYAESVVNSNITQNTSDQDGSVFV

>tguXCR1 XP_002196670.1 Chr 2

MDEDYYLDENYSYEYPNETNVCEMGDYFTFNIYLTAVLYILVFFLSLLGNTLVLWILLKYENLTSLTNIFIMNLCISDLVFSCMLPFWVVDQSFGWIFGEFLCKASNAIFSIGYYSGVFFLTLMTILRYLFVVNPLSTLRSQTQCCGVLVSLAVWTVSILIVVPEVIHTTVQKDLEEHRYCDYADGNWKKVDIYVRNVLFLFSFGVIIFCYFKILIILLRARSRRKHRTVRLILIIVVAFFLCWAPYNILSFLTTFPPPTCQYVKDSSLAFHISRKIAFSHCCLNPVLYVFVGVKFKRHLAQLCSLCLHCSNGQASSPRTCYEVKFQHEGTSVY

>tguCXCR7 XP_002191329.1 Chr 7

MSALDLTSILDFLETANLTEINWTCNNGECITVDATTCPGTLNKSALLYTLSFFYIFIFVIGLVANSVVVWVNLQAKMTGYETHLYIFNLAIADLCVVITLPVWVVSLVQHNQWHMGEITCKITHLIFSINLYSSIFFLACMSVDRYLSVAYFTNSSSRKKKIIRRCICILVWLLAFSASLPDTYYLKTVSSNNETYCRPVYPEESFKEWLIGMELISVVLGFLIPFPIIAVFYFLLAKSISTSSDQERKSNGKIIFSYVVVFLVCWLPYHVAVLLDIFYSLHFIPFSCQMENFLYATLHITQCFSLVHCCVNPILYSFINRNYRYELMKAFIFKYSAKTGLTKLIDASRETEAEYSALKQTAIGLQLQEKEFLDSQT

>tguCCBP2a XP_002193020.1 Chr 2

MGLSVEKGHWSNTTSGVTATTTGTWLDAANSSEYLYEYLDEEDYGLYGLCTKEEVLSFSRVFLPSFYTVIFLVGMAGNALLFTVLLMHIKKKKKMTELYLMNLVVSDFFLLLTLPFWALYISQWVTWDILCPFLSAMYTLNFYSGIFFVSCMSLDMYLQIVHAWSPHSSTVWRNSILILLVMWILSIALSIPDGLFTSTRQTHNKTIMCAQDYGQEHLFWKVVFRVTQNILGFLFPFLFMTFCYSRIACVLNTSQIPGSRRALCLVLTLVGVFFVLWCPYNVVLILHSLQDVGVIRSCESSRKLDYALQITESLSFVHCCLNPLLYAFVKKRFRAYLWKIPQAIFRRGAFFSIQDSQTSLSCSRRYNRKDWTPLS

>tguCCBP2b XP_002190074.1 Chr 2_random

MSGVTATTTGTWLDAANSSEYPYEYLDEEDYGLYGLCTKEEVLSFSRVFLPSFYTVIFLVGMAGNALLFTVLLMHIKKKKKMTELYLMNLVVSDFFLLLTLPFWALYISQWVTWDVLCPFLSAMYTLNFYSGIFFVSCMSLDMYLQIVHAWSPHSSTVWRNSILILLVMWILSIALSIPDGLFTSTRQTHNKTIMCAQDYGQEHLFWKVVFRVTQNILGFLFPFLFMTFCYSRIACVLNTSQIPGSRRALCLVLTLVGVFFVLWCPYNVVLILHSLQDVGVIRSCESSRKLDYALQITESLSFVHCCLNPLLYAFVKKRFRAYLWKIPQAIFRRGAFFSIQDSQTSLSCSRYAAEIEMLSITNAS

>tguCCRL1a ENSTGUT00000019226 Chr 2

MNNSTDYWIEDEEDDLNPLIDYNTYELLCEKGDVRNFRKLFLPVFYALAFTVGVAGNSLVVAIYAYCKKPKTKTDVYIMHLAIADLLLLFTLPFWAANAVQGWELGNPMCKLTSSLYTMNFSSSMLFLACISVDRYRATSDSQGHRRVGKHCSVTCICVWLAATFLSIPELIFNQVKKHNERNECLPVFPMNMETLLKSTIQILEIILEFLLPFLVMLICYSATAWAIFRSANVKKSRPFKVLLAVVATFIVTQLPYNIVKLWRAIDIIYILVTDCHTSKIMDVALQVTKSIALSHACLNPLLYTFLGASFKMHIMKIAKNYGYWRRQQHNGRPEEISMNYEDPTEETTSFTI

>tguCCRL1b ENSTGUT00000004599 Chr 2

MNNSTDYWIEDEEDDLNPLIDYNTYELLCEKGDVRNFRKLFLPVFYALAFTVGVAGNSLVVAIYAYCKKPKTKTDVYIMHLAIADLLLLFTLPFWAANAVQGWELGNPMCKLTSSLYTMNFSSSMLFLACISVDRYRATSDSQGHRRVGKHCSVTCICVWLAATFLSIPELIFNQVKKHNERNECLPVFPMNMETLLKSTIQILEIILEFLLPFLVMLICYSATAWAIFRSANVKKSRPFKVLLAVVATFIVTQLPYNIVKLWRAIDIIYILVTDCHTSKIMDVALQVTKSIALSHACLNPLLYTFLGASFKMHIMKIAKNYGYWRRQQHNGRPEEISMNYEDPTEETTSFTI

>aplCXCR2 ENSAPLT00000004348 Scaffold 6660

GMDSISFIGDLSDLLSNYTYDYSTALPDAAISSSPCRPDGSVLNKYLVVFIYCLVFVLSLLGNGLVVLVVTSSHANRSVTDVYLLNLAVALTLPLWAAYRAHEWVFGTVLCKAISVLQEANFYSGILLLACISVDRYLAIVYATRAATEKRHWVKFVCLAIWLFSVLLSLPVLLFREAFVSPSNGTVCYERIRGEDTAKWRVVLRVLPQTFGFALPLLVMLFCYGVTVRTLLRTKNAQRQRAMKVILAVVLVFLVCWLPYNITLVSDTLMRTRAIAETCERRNHIDTALSVTQVLGFSHSCLNPIIYAFIGQKFRNSFLKILAQRGLISKDAVARYGRASYASTSGNTSTTL

>aplCXCR4 ENSAPLT00000001873 Scaffold 767

SSGLIIEFSDNGTDEIGSGDYGDYGEPCFQHENADFNRIFLPTIYSIIFLTGIIGNGLVIVVMGYQKKQRSMTDKYRLHLSVADLLFVITLPFWSVDAAISWYFGNVLCKAVHVIYTVNLYSSVLILAFISLDRYLAIVHATNSQRPRKLLAEKVVYVGVWLPAVLLTVPDIIFASTSEVEGKYLCDRIYPHENWLISFRFQHILVGLVLPGLIILTCYCIIISKLSHSKGHQKRKALKTTVILILAFFACWLPYYIGISIDTFILLGVIRHRCSLETIVHKWISITEALAFFHCCLNPILYAFLGAKFKTSAQNALTSVSRGSSLKILSKSKRGGHSSVSTESESSSFHSS

>aplCXCR5 ENSAPLT00000000743 Scaffold 1650

MNLPPCNQILSVPQSQAELSGYYEADNTTPSLEGYFCFNPASSVVGNQRDPFRKVFMPLIYLLMFVLGTVGNALVLVILERFKRSRTTTENFLFHLTLANLALLLTFPFSVVESLAGWVFGTFLCKILSAVHKINFYCSSMLLGCIAVDRYLAIVYAIHTYRKRRARSIHLTCTAVWLCSLLLTLPDLIFMEVWTDESNRSICYFPEIGIHGNNAWLATRFLYHTVGFFVPLLVMCYCYMAIVRTLCQSQRLQRQKAVRVAILVTGVFLLCWSPYHIVIFLNTLTKLEAFTKNCLLEDQLDTAIMVTEAIGFTHCCLNPILYAFIGVKFRNDFFRILQELGCISQETLQEILEVTRKGSGIESDNTTSISTF

>aplCXCR6 ENSAPLT00000001961 Scaffold 953

MATADAVTFYYNFSAIDPNENGIENFYTFITIFLPCVYSFVFIFGLAGNALVFIILVFYEKLKTLTDIFLLNLAIADWIFLWTLPFWAYSAAQEWIFGTVTCRIIRGLYNLNLYTSMLTLTSITFDRLISITFATKAHMSQTKRLKWGKLICGLIWVISVAFATPQLIFSDVFTIDKTICLEKYPDHHIELVLEVIQVTLGYFIPMLTMIICYSLIIKTLLHARNFQKNKSLKKIFSVVAIFILTQSPYTFLRLMKIIDWSFNLDSNFDYAIVITEALAYFHGCLNPVMYFFMGVKFRKNFQKIIKSSRCFKQQVAVRQWHTTEDEGSKTYTVSNNADVTSMYPL

>aplCCR2 ENSAPLT00000001933 Scaffold 953

MENDTIGFLDMATTTEFDYGDSAPCTGTEEKHFAANLLPPLYSLVVIFGLIGNVLVVLILIKYKRLKSMTDIYLLNLAISDLLFIFSLPFWAYYAVHDWIFGEALCRILSGVYLLGFYSGIFFIILLTIDRYLAIVHAVFALKARTVTYGILASIVTWAVAMLASVPGIVFHKTQKENSRYTCSAHFPSDSSINWKYSYILKMNILGLVIPMFIMIFSYSQILKTLLGCRNEKKQKAVRLIFVIMIFYFIFWTPFHIASFLHTFQSSFFDPDCETQSKLEKAIQVTETISMIHCCINPVIYAFVGEKFRKYLYNFFRKYVAAHLCKKCPSVYREKLERVNSTFTPSTAEHDISTGL

>aplCCR4 ENSAPLT00000001191 Scaffold 340

EEKVTDIFIFFHSQESGKKNMSSSSTESFEVDTSTLYDYYDNYNDAPKPCSKESVKRFAASFLPVLYTLVFLVGLMGNILVIVVLFKYKRLKSMTDVYLLNLAVSDLLFVFSLPFWSYFAIDQWVFGTPWCKIISWIYLVGFYSGIFFIMLMSIDRYLAIVRAVFSLKARTTFHGLITSVIVWLVALSASVPELVFRESFHEQNYTTCKHRYPGNFTTWKLFSTLEINVLGLLIPFIVMAFCYSMIIKTLVHCRNEKKNKAVRMIFAVMIVFFFFWTPYNIVIFLQFLELIGVIRDCQASRNLDYAFQITEIFGLFHCCLNPVIYFLMGEKFKKYLKMLFKNWWLPGDICKWCGLHISYHTESTSSFHTQSTGDQDAL

>aplCCR5 blast search Scaffold 953

VLGNVLVVLILIKYKRLKSMTDIYLLNLAISDLLFIFSLPFWAYYAVHDWIFGEALCRILSGVYLLGFYSGIFFIILLTIDRYLAIVHAVFALKARTVTYGILASIVTWAVAMLASVPGIVFHKTQKENSQYTCSAHYPSDQRNVWKQFLTLKMNILGLVIPMLIMICSYTQIIKTLLQCRNEKKHKAVRLIFIIMIVYFFFWAPYNICILLRDFQGSFSISSCEGSGQLHKATQVTETISMIHCCINPVIYAFAGEKFRKYLHSFFRKQIASHFSKYCPVFYVDPSERASSTYTQSTGEQEVSAAL

>aplCCR6 ENSAPLT00000010912 Scaffold 696

FKSAKPLLYRICIVLLPIIFQLYKTESSVTEYMYDSDYPSQIAQPCSKGEVRTFTKAFLPVAYSLICIIGLVGNIFVVMTFALYERTKSMTDVYLFNMAIADILFVLTLPLWAVNYAADKWIFGNFICKMARGIYAINFSCGMLLLAFISVDRYIAIVQATKSFKLRARTLAYSKLICLAVWVSSILISSSSFLYSESYNFSVNETKNICDHRFDKMAESTMLKSLLLCLQVGFGFFLPFIFMFFCYTFIIKSLQQAQNSKRNKAIRVIVLIVAVFLVCQVPYNIVLLVAAVNMGKMDKSCDSEKAMAYAKYTTEAIAFLHCCLNPVLYAFIGVKFRSYFVKIMKDLWCMRHKKYNKLHSCEFYRVMAEETEKEVTTSFSG

>aplCCR7 ENSAPLT00000008323 Scaffold 3143

SSIGKQLKVTLVFSLPLIFQVRLCQWNNVTDDYDSNTTIDYTMFETVCEKEEVRNFRAAFLPAMYSLICFTGLLGNGLVMVTYIYFKRLKTMTDIYLLNLALADILFLLTLPFWATSAATYWCFGETACKAVYCICKMSFFSGMLLLLSISIDRYFAIVQAASAHRFRPQMILISKITCVVIWVLAFILSIPELVHSGVNNPDNHPRCSIIANDLQTFNTGIKVSQMVFGFLFPLVVMSVCYLIIIKTLLQARNFEKNKAIKVIIAVVIVFIVFQLPYNSVMLAKTISAFNHTTSCEESKKLDMADDVTYTLACFRCCLNPFLYAFIGVKFRNDLFKLLKELGCLSQKRLWQLSACRESKRFSFAMETETTTTFSP

>aplCCR8a ENSAPLT00000000942 Scaffold 488

MDDSLGSLLSSGDLEDLLVDYQSTVNSSASYDDIFLYSELNIDCELETIPAFAQIFFPVLYTVLFVTGLVGNALVVWVLLAFKKVRAMTDIYLLNLAISDLLFVFSLPFLVQYSLVSQWTFGNAMCKIVSSAYFIGFYSSSFFITIMSIDRYLAIVRSVYALRVRTSAHGVIASLALWAVAILASAPDLIFFQEMDDSNRTVCLPHYPGSDNSWKIFSNFEVNVLGWLIPVGILIFCYHNILKNLQRCHTRNKYKAMKLVFIVVTVFFLFWTPVNVVLFLDSMRSMHIIDDCQASQKLDLALELAEALSFVHCCLNPIIYAFVGEKFKKYLCEAFGKYARFLLTCNNYSVFHRHKLDRQSSVHMGSSQSSFVGSVL

>aplCCR8b ENSAPLT00000001945 Scaffold 953

MNPTSHFVETTEYAYGYDENTAPCNEGNSFHRFKSLLLPILYCLVFVFCLLGNSLVLWVLLTRKKLMTMTDVCLLNLAASDLLFVVPLPFQAHYAAEQWVFGNAMCKIMAGIYYTGFYSSIFFITLMSIDRYIAIVHAVYAMKIRTTSCGIIISLILWLVAGLASVPNIVFNQQLEIEQSMQCVPTYPPGSNTWKVASQFAANILGLLIPLSILICCYAQILKNLQKCKNRNKIKAIKMIFIIVIVFFLFWTPFNVVLFLDTLQSLHIINDCQASNRIALALQLTETISFIHCCLNPVIYAFAGVMFKAHLKGLLQSCVRVF

>aplCCR8c ENSAPLT00000001368 Scaffold 488

MSPAPNSSSNYYEDYYYPEMASTCTTEHSKYFTSMFFPVLYTVLFVTGLVGNALVVWVLLAFKKVRAMTDIYLLNLAISDLLFVFSLPFLVQYSLVSQWTFGNAMCKIVSSAYFIGFYSSSFFITIMSIDRYLAIVRSVYALRVRTSAHGVIASLALWAVAILASAPDLIFFQEMDDSNRTVCLPHYPGSDNSWKIFSNFEVNVLGWLIPVGILIFCYHNILKNLQRCHTRNKYKAMKLVFIVVTVFFLFWTPVNVVLFLDSMRSMHIIDDCQASQKLDLALELAEALSFVHCCLNPIIYAFVGEKFKKYLCEAFGKYARFLLTCNNYSVFHRHKLDRQSSVHMGSSQSSFVGSVL

>aplCCR9 ENSAPLT00000001979 Scaffold 953

KTSLDYYRNSTVMSLYANPANDTELMCDRRQVWQFARAFLPVFFWLIFSVGTVGNALVVLIYCKYRFRRSMMDRYLLHLAVADLLLLFTLPFWAKAASDGWVFRNFLCKVVNSMYKINFYGCILFLTCISFDRYITIVQATKAKTSKRRRLLHNKLVCLAVWLTSIGLCIPEIMYSQSKQVGDMTVCKMMYPPNVSMVFRVAVLALKVTIGFFLPLLVMVICYTLIINTLLQAKRCQKQKSLKIITMIITAFLLSQFPYNIVLLVKTINTYTGVVYSCWATNGLDIGLQVTQSIAFLHSCLNPFLYVFAGERFRTALARLVRRPRGQEQCSSVCDSQEHSSNWSFAMLGRRRVRSSLTLSTHLTSSIVPASCQVFV

>aplCX3CR1 ENSAPLT00000001385 Scaffold 488

MAEAFPEVTTEYAYDEYAFTCDKTDIQEFGKIFLLIFYIVVFALGLMGNLMVVFAILRAGSKKSITDIYLLNLAVSDLLFVVSLPFWASNTVRGWTLGTIPCTVVSSLYYIGFFGGMFFITVISIDRYLAIVRATYSLKSRTMKQGFLITCGVWATAVLFSVPHFVFSQLLENDCIPVFPQELENIWPVFCNVELNTIGFLIPVCIICYCYCGIIKTLLSCKNQKKTRAIKLTLVVVVVFFLFWSPYNVLIFLETLKHYELFVNCQQIKSLDYAMHLTETVAFSHCCLNPLIYAFAGEKFRKYLYSVCLKYCPCLCFCGPCSRYQVSSSASYAESAVNSNITLNTSDQDGTVFL

>aplXCR1 ENSAPLT00000001951 Scaffold 953

MDEEEYPAYSDNNYSYGYSINESNVCEMGNYFVFYTHLTTVIYSLAFFLSLLGNTLVLWILFKYENLVSLTNIFIMNLCVSDLIFSCMLPFWVVDQTFGWIFGEFLCKAMNAIFSIGYYSGVFFLTLMTILRYLSVVNPLSTLRSPTQCCGSVVSLVVWTGSILIVVPEVMHTTVHEDVYGYKTCDYNDWKMKKVDVYQRNVLFLFSLGIIIFCYLRILIILLGTRSRRKHRTVKLILIIVMAFFLSWAPYNILSFLLTFPPSTCQYEKDINLAFHISRKIAFSHCCLNPVLYVFVGVKFKSHLIRLCSQCLPCGNSQVSSPRICSQGKFHYEDASIY

>aplCXCR7 ENSAPLT00000002309 Scaffold 1555

LDLTSILDFLEKANLTEINWMCNNSDCITVDATTCPGTLNKSALLYTLSFFCIFIFVIGLVANSVVVWVNLQAKMTGYETHLYIFNLAVADLCVVITLPVWVVSLVQHNQWHMGEITCKITHLIFSINLYGSIFFLACMSVDRYLSVAYFTSSSNRKQKIVRRCICILVWLLAFSASLPDTYFLKTVSSNNETYCRPVYPEESFKEWLIGMELISVVLGFLIPFPVIALFYFLLAKAISASSDQERKSSGKIIFSYVVVFLVCWLPYHATVLLDIFYSLHFIPFSCQMENFLYATLHITQCFSLVHCCVNPILYSFINRNYRYELMKAFIFKYSAKTGLTKLIDASRVSEAEYSALEQNAK

>aplCCBP2 ENSAPLT00000000679 Scaffold 1087

SKAATTAAALMDAQDYAANTSDYPYEYLDEVDYVQYGLCTKEEVLSFSRVFLPAFYTVVFLVGLAGNLLLFVVLVLYIRKKKKMTEVYLLNLVVSDFFLLLTLPFWAMYISQRVTWDMLCPFLNAMYTMNFYSGVFLVSCMSLDMYLQIIDACSPRSFTTQRKSILVLVAVWVLSILLSVPDGLFTTTKHTNNQTIVCTRDYGQEHLFWKVVFQVIQNVLGFLFPLLLMVFCYSRIACVLTTSRMPGLRRALCFVFTLVGVFFVLWFPYNIVLILHSLQDVGVIRSCERSRQLDYAIQITESLAFVHCCLNPLLYAFVKKRFRLYLWKICRRSAFVDIQTSETSPSCSR

>aplCCRL1 ENSAPLT00000001901 Scaffold 953

MDWDMNNSTDYWTEDEEEDLNSVIDYNMYELLCEKDDVRKFRKLFLPVFYALTFTVGVAGNSLVVAIYAYCKKLKTKTDMYIMHLAIADLLLLFTLPFWAANAVQGWELGTSMCKLTSSLYTMNFSSSMLFLACISVDRYRATAGTQGHRRGGKRCSMTCCCVWLAAVLLSIPELIFNQVKKHNNRNECLPVFPVNMETLLKATIQILEIILEFLLPFLVMLTCYSVTARAIFRSANAKKSRPFMVLLAVVAAFIITQLPYNIVKFWRAIDIIYLLITDCDASKTIDIALQVTKSIALFHACLNPLLYAFLGASFKMHIMKIAKNYGHWRRQQQNGVTEEISMNSEDRTEQTVSFTI

>acaCXCR1 ENSACAT00000000998 Chr 1

RYFVAFLYCLVCLLSLIGNALVVLVVAYNKRNRSVTDVYLLNLAIADLLFALTLPIWAVFRAHEWIFGTGMCKFTSVLKEVNFYSGVLLLAFISVDRYLAIVYATRHATEKRHWVKFVCVGIWVFSLLLSLPMVTYREVFHAPNSSLRVCYENIGGNETSKWRVVLRILPQTFGFLVPLAIMLFCYGVTVHRLFQMKNNQKKKAMKVILVVVLVFLFCWLPYNITLFADTLMRTGVITEDCKRRGIIDAGLSGTEILGFSHSCMNPIIYAFIGQKFRNNFLKILVERGIISKEVLIRYRKGSSFSSTSGNTSTTL

>acaCXCR2 ENSACAT00000000984 Chr 1

DYLNYSYESTLNPDSAPGPCRPKSASALIRYFVAFLYCLVCLFSLVGNALVVLVVTYNKGNRPVTDVYLLNLAIADLIFALTLPIWAIFRAHEWIFGTAMCKIISAMKEVNFYSGILLLAFISIDRYLAIVYATHHVTEKRHWVKFVCVGIWVFSLLLSLPMITYREVVHAPNSAQMVCYENIGGNETTKWRVVLRILPQTFGFLVPLAVMLFCYGVTVHRLFQMKNNQKKKAMKVILVVVLVFLFCWLPYNISLLIDTLMRTRVIAETCGLQDAIDAALLGTEILGFSHSCMNPIIYAFIGQKFRNNFLKILVTRGIISKEVLIRYRKGSTFSSTSGNTSTTL

>acaCXCR3 ENSACAT00000025783 Scaffold GL343868.1

GDDAMVLTSGDLFDLVGNSSDGYYDYDNWTDACCSSSSVCNSNATQGFSGSFLPAFYSLICLLGLWGNGMVIAVLLRAKEALAGTDVFLFNLAVADILLVLTLPFWAVQEARGWVFGTFLCRVVGGAFKINFFASIFFLVCISLDRYLSIVCVVRMYRRSKASAVHLTAVAVWVACLLLTVPDFVYLSAEYDSRQKSTSCSLVFPPDSATQWKVGLSLFNQVGTFFLPLLAMGYCYAHIVFTLLLSKGFRKHKAMRVILAVVGAFFLCWLPYHSIQFATTFLKLDCAWQERLEVAEVVATALGFFHCCLNPLLYAFMGVKF

>acaCXCR3L XP_003229345.1 Scaffold GL343868.1

MVYLYIYLTCFTAVSVPFRLDPFALSPSQFPSDLPDINPGAFPCTQSEVGSFARSFGPAVFSVSFLLGLVGNGLVLAVLSSRRCPWLLADRFLFQLAVADLLLVLVLPFRATQFSQSWAFGEPFCKLVGALSAMSSYSTAFLLACVTLERYLAIVHSLQPRWTPHGALLASTLLWAASIALSVVELHFRTVSYVSQAGAVVCHLGFDARDANTWRLSLRLVSFLLGFLFPVAVMVYCYVRMLVKLRQLFFRVMALRLLSVILLLFVLCWGPFHGFVLVDSLQRLGHVGRDCAKEKILDFGLLFTESVGLVHSCLNPLVYAFVGAKFRKELSGLFRDWRQCRRQQQIAPSPVGSGRETEFSVAQMADYSVMM

>acaCXCR4 ENSACAT00000000709 Chr 1

PGIFIFMTENSTDEPGSGDDGDYQEVCLQQENADFNRIFLPTIFSIIFLTGIIGNGLVIVVMGYQKKLRSMTDKYRLHLSVADLLFVITLPFWSVDAVISWYFGNFLCKAVHFIYTVNLYSSVLILAFISLDRYLAIVHATNSQRPRKLLAERIVYVGVWLPALLLTVPDIIFASTSEVGGKYVCQRFYPHETWLISFRFQHILVGLVLPGLIILTCYCIIISKLSHSKGHQKRKALKTTVILIVAFFACWLPYHIGISIDTFVLLGFIENGCTFEAILQKWISITEALAFFHCCLNPILYAFLGAKFKTSAQNALTSVSRGSSLKILSKGKRGGHSSVSTESESSSFHSS

>acaCXCR5 ENSACAT00000017184 Scaffold GL344088.1

MTLGRLRTTDLTPSLLSPFPPQYNYSEPDGEWNYSDYICVDEPEAAEHFGKFLVPAVHLLIFLLGGLGNLLVMVTLWRYRRARTPTEVFLFHFALANLLLVAMFPFGAAESLAGWVFGTVLCKGLSAATRVSFYSSSLLLAGISVDRYLAVVHALRTFQRPRSLSVHLTCLAVWLLSVLLAMPDLLFTEVWPDSGNLSICYFKKYGEQGVRSWLATRFLYHVVGFFLPAAVMCFCYLAIVRLLCRSQRLQRQKAVKVAILVTCVFLLCWTPFHVVTFWDTLTRLAPNSCAHEYSLAAAIALTELLGYSHCGLNPFLYAFVGERFRHDACRVLHDLGCLSQGALQNVVGSRGVESTTETTGSNSRHQASP

>acaCXCR6 ENSACAT00000014162 Scaffold GL343324.1

MFLQFNKIFLVCMYSVTCIFGISGNALVLIILIFYEKVKVLSDIFLVSLAIADLCFLCTLPFWAYMAADEWIFHTLPCKLIRGLYTMNLYGSMLTLTCITIDRYFAVVQATKAHVSQAKRRTWGIAACILVWVISLAFAMPQFIFSTEASNGKKVCHSSYPSEDTHKFTEVIQMVLGFFFPIVVMVVFYSIIVNTLFKAKGFHKHKSLQIIFAIVVAFILTQTPYNILKIIRAVDKHVAMHFNFDYGLVITEAIAYFHGCLNPILYFFIGVKFRKNLAKILKKFGCIKHQHITKQGQTTEEDISKTCTDTHNMEETSMYPL

>acaCCR2 XP_003225805.1 Scaffold GL343324.1

MNTPEMADGFTTISDYGNLPGPIYNPYVNIFSSYVVPPLYSLVFIFGLLGNALVVLILIKYKKLKNMSDIYLLNLAISDLVFIISLPFWAYYAANEWVFGNAVCKILSGVFRAGFYSGSFFITLLTIDRYLAIVHAIFALRARTVFYGTFSSAITWVVATLASVPALLFSHVQKEGESCKCNLFYPPGKEEEWKQVVTLMMFILGLAIPLAIMIFCYYQIIWVLIKGQNERKRKVVRLIFAIMIVYFILWMPYTITSLLHTYQNAFFSCGLDADCDGNFALALEVTEVIAMIHCCLNPLIYAFVGENFRKYLSVFFQKHVAVYLCRLCPGQPRPKLEQPSSSYRSTTVHNIHFSL

>acaCCR4 ENSACAT00000014042 Scaffold GL343324.1

EPCSKDGVKRFSSWFLPTFYSLVFFLGLAGNTLVLLVLFKYKRLRSMTDIYLLNLAISDLLFVFALPFWSYFVADEWVFGDGLCKFISWVYRTGFYSGIFFIMLMSIDRYFAVVHVVFALKARTVSYGTLASLVVWLVAITASFPELIFSEAKSDYNHTECKSVYGKNDTMWKLFTALETNILGLLIPFMVMLFCYTHIVKTLMHCRNEKKKRAVKMIFAVMIVFFVFWTPYNIVLFLQYLLDVDILTGCSISKNLDYADQVTQTLAFFHCCLNPVIYFFMGQKFKKYIKLFFKNCILTKRLCKPCGLPDTLSFESSSSFRTQSTSEEE

>acaCCR5a ENSACAT00000014112 Scaffold GL343324.1

PCHSVAVQEFASHVLPTLYSLVLVFGMLGNALVVLILIRYKKLKSMTDIYLLNLAISDLLFVVSLPFWAYSAAHEWIFEDAMCKILSGIYVVGFYSGSFFIILLTIDRYLAIVHAVFALKARTVTYGIVTSAVTWCVAILASIPWLIFNKLQRENNHCRCTLSFPPETHVNWNQFLTLKINLIGLIFPMIVMIFCYTQIIITLMRCRNDKKNKAVRLIFIIMIIYFLFWAPYNIVLLLQTFQTSFRLDNCYSYSNLGVALQVTETLAMAHCCINPVIYAFAGEKFRKYTCTFFRKHIALHLSKHCRFLYTEPLERASSTYSHSTGEQDLSAAL

>acaCCR5b ENSACAT00000021030 Scaffold GL343324.1

YGAAPCKDIKGQKFASHVLPTLYSLVLIFGMLGNALVVLILIRYKKLRSMTDIYLLNLAISDLLFVVSLPFWAYSAAHEWIFEDAMCKILSGIYVVGFYSGSFFIILLTIDRYLAIVHAVFALKARTVVYGIATSAVTWCVAILASIPWLIFNKLQQENNHCRCTLHFPPETHVNWNQFLTLKINLIGLIFPMMVMFFCYTRIIVTLMRRRNDKKNKAVRLIFIIMIIYFLFWAPYNIVLLLQTFQTSYHLDNCYSYSNLGVALQVTETLAMAHCCINPVIYAFAGEKFRKYTCTFFRKHIALHLSKHCRFLYTEPLERASSTYSHSTGEQDLSVPL

>acaCCR6 ENSACAT00000010463 Chr 1

CDKNEVRNFTKTLLPVAYSLICMFGLVGNIFVVMTFALYKKTESMTDLYLCNMAIVDILFVLTLPFWAVNYALNRWIFGDFMCKLIKGIYALNFVCGMLLLACISMDRYISIVQATRSFKFRSRTLAYRKVICLTVWVASILISCPTFIFSGSYQSTNVSNDICEHKSSTEFDVTLKLLIINIQLFFGFFIPMLFMVFCYTFIVKKLVQAHNSKRSKAIRVVVSIVIVFLICQVPYNMVLLVTAATMKTLDKTCQSEKQMAYAKYITETFAFLHCCMNPVLYAFIGVKFRNYFVKVMAGLCCV

>acaCCR7 ENSACAT00000016157 Chr 6

DNSTLDYSNFEELCKKEEIRRFRATFLPTIYSIVCFVGLAGNGLVMLTYIYFKRLKTMTDIYLLNLALADILFLLTLPFWAVSAAKYWVFKEFACKAVHCICQMSFFSGMLLLLSISIDRYFAIVQAPSAHRHRSQRVLASKVTCLSIWILGFILSLPEAINRGVYDYESPTPRCTIVTANLLAFSTSIRISQMVFGFFIPLLVMTFCYWIIIRTLLQARSFEKNRAIKVLIAVMVVFVLFQMPYNSVMLAETITAFNNTTGQCDAIKRIDVASDVTYSLACFRCCLNPFLYAFIGVKFRNDVLRLLKDLGCINQAQLWKWSTYRENNRCSIATETDTTTTYSP

>acaCCR9 ENSACAT00000026110 Scaffold GL343324.1

MAVNQYISSNDIAYLAHTQKQTNNGVHLDLSCDMSQVKLLAKTFLPTFFWCVFFVGTIGNAFVVLVYWKYKGKKNLTDKYLIHLAIADLLFLFTLPFWAIAAHDGWYFNTFMCMYKINLYSCMLFLMLISFDRYTVVVRSTRARHSKQKRLTHHKLICFGVWLMAVSLCIPEIIYSQTEQSSNITICKMIYPPNVNRSIKVINLSLKIAIGFLLPLVVIVVCYTCIIHTLLRAKKTPKHKLFKIMTIIILVFLLSQVPYNSILMVKTMVLYAPVIKDCKMLDRIDIGFQLTQSIAFLHSCLNPFLYVFAGQRFRKTLFETLK

>acaCCR10 ENSACAT00000013858 Chr 6

LTPWTNEFPVSTEWDLWYYDASPPALPELCEKEAIRVVAHICLPVMAILFCVLGVLGNGILLLVRIRYHIQTIGDALLLHLAFSDLLLLLTLPMGVAAMMGRWHLGTATCQGLQGLHALNFYSGFLFLTGLTLDRYVAIRAPIAHRLRPATTCWARLGLGLIWLLSSSLALPHFLYARMEDHEGFQLCRVATVAAAISLVQVALGFVLPFVVMVVSYMAIARTLLSSPCAQSQRALWLILSLVFLFLALQLPYALLTLLDTADLMSQQVSSCKVIFHRDLALLITSGLAFARCCLNPVLHSFLGVRFRKDLRRLSRDIGCLGE

>acaCX3CR1 ENSACAT00000014047 Scaffold GL343324.1

MTSTEFDYSQLVSPCDKVDIHITMKVFLCVLYVVVFAIGLTGNFLVVLTILKAGGQRSITDIFLLNLAISDLLFVLSLPFWAFYFIHGWTLGNLLCQIVSSLYSVALFGGMFFITVISIDRYLAIVHATYAMKARTIHRGYITSAAIWTLAVLFAAPHFVFVQESEKQCTSLYPPHLQTLWPVFSYLEMNIIGFLLPVCIMSFCYLGIIKTLFSCKNTRKKRAVKLILTVVIVFLLFWAPYHVLLFLQMLRTYNYFETCVSLRVLDYIVQVTETIAFSHCCLNPIIYAFASEKLRKFLCHLVLKCFS

>acaXCR1 ENSACAT00000014122 Scaffold GL343324.1

DISELSALLISILYSVIFLFSLLGNSLVLRIVLKYESLMSLTNLFIVNLCISDLIFSCTLPFLIVYHSYGWIMGEFLCKAVSGIFSISYFCGVIFLTIMTILRYLAVVDPLSTLRTQKKRSGILVSLAVWVTSLLFVIPEILSIQVTTDIDGRYGCYYQAFYPWEMVELCLKVLFFLISFMIIAICYTGMLDILLRSRSQSRHRTVRLIFAIVLVFFLSWAPYNVLGFVYALSEQNVIESKCQTKKDIYFAFDISRTVAYCHCCLNPVLYVFVGVKFRRHL

>acaCXCR7 ENSACAT00000010495 Chr 1

VDLPPIFEFVEGGNLTETNLTCSNGDCITVDSLSCPNTLNKTALLYTLSVFYIFIFMIGLVANFVVVWMNFQAKTTGYETHLYIFNLAVADLCVLITLPVWVVSLVQQSQWHLGEITCKITHLVFSINLYGSIFFLACMSVDRYLSVAYFTTSSNAKKKRIRRCICIFVWLFAFFVSLPDTYYLKTISTNNETYCRPVYPEESAREWLAGMELSSVLLGFIIPFPVIAVFYCLLAKAISASNDQERKSNGKVIFAYVVVFLVCWLPYHVAVFLDLLLAFHFIPFSCQMENFLYTALHVTQCFSLVHCCINPILYSFINRNYKYELMKAFIFKYSAKTGLTKLIDTSRVSEMEYSALDQNAK

>acaCCRL1 ENSACAT00000000757 Chr 6

MESATNPSELYYDSYYDESNATVDYALHEMPCLKEEVRTFNKSFLPAFYSIAFLIGLPGNSLVIAIYAYIKKLKTRTDVYIMHLAIADLLLLFTLPFWATNAVHGWVFGNPLCKITTAIYTMTFSVSMQFLAWISVDRYNAIVKSPSQQRTTKLCSKICFFVWMAGTFLCLPDLIFNQVKEFHGKIACVSTFPESLSKIIKVTIEVGEMALCFVLPFFIMLTCYSAVARALFKSPSVKKTQPLKVLAAVVSVFIVTQLPYNVIKLWRAIDIIYPLITNCKASKAMDVAFEVTNSIALFHSCLNPLLYFFMGASLKMHMVKLAKRYGYWRRQQNIPPEEIPMDYEESAEQTSSLII

>acaDARC ENSACAT00000028578 Chr 846

YTYDGYPSYDDNLPEPCQFTFCLRFLSSILALLMVISILGSMSSLALGISLAKRPILWKQRHPGKYGFFLTSAATGLFAATLPFFAVGLKHGWVFGPHFCQLARALRYGCIFAQGLMVAGSTWRMLPDVPRSLFLTGLFFLGFVCATPAIVISSPGDDCFSSLEPNLQPWSLAHILVSLAIFVILPLAMLVAKAALKWHGKNERLQLNLSWVFYIFWSPYGVALLLDKLLEDGLLTSTCHFREHLDYFLGISEGFGILHCFLLPLFI

>xlaCXCR1 NP_001082234.1

MSFNFDGSFFNDIDFSDIPTGFPTVISAPCKSTWVINKYFVVVVYALVFFLNVVGNSLVVLVIYNNKLKRSSTDVYLLHLAIADLLFATTLPFWAAYKASQWVFGIFMCKAVSVLQEVNFYSGILLLACISVDRYLAIVHATEAVTQKRHWVKFICLGIWIFSLVVSLPTLLFRTVFKSPRDAYVCHDSIGNENTEDWMIILRIGRHLVGFFIPLLIMLFCYGFTIKTLYQTKSSQKHRAMKVIFAVVLAFLICWLPYNLTVIVDSLMRTRFINETCEKREHLDAALSTTEIFGYTHSCINPILYAFIGQKFWNSFLRILASKGIVNKSFLARYARGSTFSFGSTSGNTS

NTL

>xtrCXCR3 ENSXETT00000000415 Scaffold GL173018.1

RGETSEPLQIRSETEDLLANMANSGHGIYDDLDVETSSIFNYDYSTSSESNDVAPCDLQTTIIFDRSFLPAFYSILFLLGILGNVLVMVVLLQNRKRLQSTDIFLLHLALADILLVVTLPFWATQAVSGWLFGNVLCKTVASIFKINFYACTFLLVCISCDRYLAIVYAVQVYKKHRTNLVHWSCLFVWCLCVGLSVPDMVHFQVAYEPRTNVTECQPVFGSSNFKTWRVSMAFLYHIVGFLLPLCFMLYCYTHIIHTLFQTHGFKKQRALRVIITVVVAFFLCWTPYNIVALLDTLNLLHVLADNCTIDSNIDIALSVTSGLCYFHSCLNPLLYAFVGAKFKKKLVELLSKLSCICPQIVKNYIKHSPSAKSSTWSESGETSISRM

>xtrCXCR3L NP_001011067.1 Scaffold GL173018.1

MAENQDDYYSSDDIFTGLPIIPPGASPCNYEVTSRFNKWFIPATFLLVFLLGLVGNGLVLYVLKSRRCSWHLSDHYLFHLTLSDLFLGLTLPFWATQYAYGWVFGSVPCKLVGALFSINMYSSIFFLACIGLNRYFAIVHAVELHRKQRPIHTFLICAVVWATSCLLSLQEFYFRDVDFIKQLKSHSCHYKFDPETADTWRTTIRLINLSLGFLLPLFLMFFFYCRIFCTLRKSRHGHSYRSQVVIVVLLFVFVLCWGPYNTLLLIDSLQRLDVIAPSCPLFQKLDIGLTVTETLGLSHVCLNPFIYAFVGVKFKSELSRLSKRVSGKVISSGVTGSKEETIVIETNNSYTKVF

>xtrCXCR4 NP_001090831.1 Scaffold GL172713.1

MDGFSGGIDINIFDSNSTENGSGDFEDFSEPCFMHDNSDFNRIFLPTIYSFIFLLGIIGNGLVVVVMGYQKKSRTMTDKYRLHLSVADLLFVFTLPFWSVDAAIGWYFKEFLCKAVHVIYTVNLYSSVLILAFISLDRYLAIVHATNSQGSRKMLADKVVYAGVWLPALLLTVPDLVFARVSDENGQFVCDRIYPIDNRETWTVGFRFLHITVGLILPGLIILICYCVIISKLSHSKGHQKRKALKTTVILILAFFACWLPYYVCLTTDTFMLLGLLKADCIWENTLHKAISITEALAFFHCCLNPILYAFLGAKFKTSAQNAFTSVSRGSSLKILSKKRAGLSSVSTESESSSFHSS

>xlaCXCR4b NP_001080681.1

MDGFSGGIDINIFDGNSTENGSGDFEDFIEPCFMQENSDFNRIFLPTIYSFIFLLGIIGNGLVVVVMGYQKKSRTMTDKYRLHLSVADLLFVFTLPFWSVDAAIGWYFKEFLCKAVHVIYTVNLYSSVLILAFISLDRYLAIVHATNSQGSRKMLADKVVYAGVWLPALLLTVPDLVFASVSNENGQFVCDRIYPIDNRETWTVGFRFLHITVGLILPGLIILVCYCVIISKLSHSKGHQKRKALKTTVILILAFFACWLPYYVCLTTDTFMMLGLVKADCIWENTLHKAISITEALAFFHCCLNPILYAFLGAKFKKSAQNAFTSVSRGSSLKILSKKRAGLSSVSTESESSSFHSS

>xtrCXCR5 ENSXETT00000022187 Scaffold GL172675.1

VHFQKFFIPLVYTLVFILGCLGNSLVLLILIKFRRSRSTTENFLLHLALADLLMLVTFPFAITESVAGWVFGSFLCKFVGVINRINFFCSSLLLGCISVDRYIAIIHAIHTFRSRRLVAVHLPCFGVWALCFLLSMPNLFVLGIQENGNVTTCTYHQSHFPSNGWWQTGRFLNHIVGFLLPLSIMGFCYAHIVAALCRSPRLEKKKAVRLAIVVTGVFLLCWTPYNVTVFIDTLDQLGLVHNCQVREELPIAITVTEFLGYVHCCLNPILYAFVGVKFRNDALRILRRAGCLSSLMPEVSLNFDRKSSATDSENGTEYLHI

>xtrCXCR6 ENSXETT00000034054 Scaffold GL172693.1

HVDMLRHYFLPILYSVTCITGLVGNLLIIIIYAFYEKMKTLTDTFMVNLAMADILFLCTLPFLAYQAAEGWIFGNLMCKIIRGGYRINLYSSMLILTCITFDRFISITQAKKLKISHSKKHRWGKLVCVIVWTVSLILAVPQFMFSKSNDKMECFETYLEGHLHLIVNSFQMTVGFFVPLAAMIFCYTFIIKTLIFSSNFQKHKSLKIIFLVVIAFIVTQLPYNIAILCHVLYKTINAKVLVITEAIAYLHACINPILYFFVGIKFRKNFCKILVDLHLAKPNLELSDNLKTTDRDSRSISAFNNTETITMHQL

>xtrCCR6 blast search Scaffold GL172809.1

MDDLFTTTEFYYTDYTDFAEGKVCSLEDVRQFKKKFAPVVLSLIFAVGLVGNLLVVITFRYYKRTKSMTDVYLLNMAVADILFVLTLPFWTVNYHKGEWIFKDFMCKFIRSIYAINFTCSMLLLACVGIDRYVAIVQVTKSFRFRTTTMAYKRVICFSVWIMSACLSGLTYYFSKCYKYNERFVCEASYPEDATALKWKLAVIIVQISLGFCIPFFVMFFCYLCIIKTLLQAHNSQRHKAIRVIVAVVAVFLVCQVPYNVVLVIKATQLGRTDGICSKNINYAYAFFITETVAFFHCCLNPVIYAFVGVKFRNYFMKIMQDLWCISKQYMVGSRISRVPSETSRRTSEVYVTEGGSSFTM

>xtrCCR7 ENSXETT00000046189 Scaffold GL173670.1

LVVDQLSAGDNTFSPEENVEYSTMDYSDFQTVCEKVEVRNFRSAFLPAMYAIVGLVGLAGNGLVMVRYLYFNRLKTGTDYYMLNLAIADIVFLLTLPFWAVSVAKTWIFGNEMCKIIYCLYKMSFFSGMFLLMCVSMERYFAIVQAPSAHRHRSKTVLISKLSSLGIWVFAFLLSIPELLYSGVKENAKVDMCIIFSDSIQSLTAKLKISQMFFGFFLPLLIMVSCYCMIIRKLLQARNFEKYKAIKVIIAIVIVFVAFQLPYNSVMLIRAFSNSTECDTSKNLDIADDVTYSLACFRCCLNPFLYAIIGIKFRNDLYKLFKDIGCLSQEKCSEWSSAKPSKRTSLAMDTETTTTFSP

>xtrCCR8 blast search Scaffold GL172672.1

MNVTDSNILDTTNDYIYSESTETFENSRTFTFHFLPVLYCLAFTFGLVGNILVIFVLVYCKKLRTTTDVYLFNMAVSDLLFVVSLPFIAYTIINEWIFGNIMCKILSTIYFVGFFSSIFFITVMSVDRYFAIVHVVFALRVRNVRWGLIVSIVVWVLALSISTPNFKFHEIVMTGNYTECVLSYPEINRQNWKIFCSLLINIFGLVIPLFILLFSYLHIIKTLQNSKCKQKRCAIRLILIVGIVFFIFWTPYNIVVFLNILKTSGAVDLETDQLQTAADVTHTLSLVHCCLNPIIYTFAGEKFRGYLRLIINRPLNFVHSSRIVGSYKTSVSDYASSTSRISKGSFSSESIL

>xtrCCR9 blast search Scaffold GL173310.1

VTEFFTGEEEYEDQYTLSTLDYIIFDPGVFCEKNSVREFASYVLPPIYWCVFLFGLVGNSLVLAVYVYNRKLKTMTDTFLINLAIADILFLITLPFWAIAASHDWVFKTALCKAVNSMYSVNVYSGMLLLACISIDRYIAIVQATKAQKYQTKKLLISKLTCFIVWALSTGLSLPEILFSVVKEEFNSTTCTMSYPAELSKTFKVSVLSLKVTVAFCLPFLVMVFCYAMIIPILVQARGFQRHKALKVIFAVLSVFILSQLPYNSILVLRVLNAANINDFECATTQNIDIAYKITQSVAFLHCCLNPFIYVFVGVKFRSDLLKILQKCIGEQQWAKGLWGDNNKKPLSETRESKMGTLSL

>xtrCCR10 blast search Scaffold GL172940.1

NCEIKIHCFDKLSQQLVLFVLQSENYNEDYSALPTDDYHSKESYISVPVLCNKKEIQNFTQIYQPIVYSLLFVTGMVGNGLVLLTYWFCRKIKSMTDVYLISLALADLLLVLSFPFLGINAVQGWIFGNIMCKVVQGLYSVNFFSGLLFLTCISVDRYIEIVQAVQAHKCRHKSIYYSKLTCIVVWVFSLLLTLPQFIYSHSESIGGFYHCKMIFPEEVTATVKGISNVAQIIFGFIIPSLVMVFCYSVIVKTLLSSKTLRRHKTLKVIISLVVVFVMFQLPHSVVIFLETADILQSKQMPCEVSKKKDVALIVTSSLAFTRCCLNPILYAFVGVTFRRDILLLLKNLGCISRASTTYNGSRRLHASSVHIDTSSFSL

>xtrCCR12 NP_001107334.1 Scaffold GL173310.1

MSSNSVEIHLSNSTMGFSTTTYEDETIVYMCSNDESIKFGAAAVPFFYYTVFTLSLLGNGLILFLLLKYEKIKTVTNLFILNLVISDLLFTITLPFWAFYHSNEWVFGNGMCKVVSSVFFIGFFSCILFLTVMTMDRYLAVVHAVSAARTRKLIYVYVASIAIWVISFVSTVPKFVLYGTRKHDSAGILCEETGFSADKIDTWRRLGYYQQLTMFFLFPLIVILYCYTLIVVKLFNTKMHNKDKAVKLISVIVLAFFICWTPYNVVIFLRLSPGDPCNDYLNNAFYICRNIAYFHCCINPFFYTFVGTKFRRHLSALLGTNCLSMFRRSSSSSRTSEYSPQTIYE

>xtrXCR1 blast search Scaffold GL172693.1

MEETYYSGFNETDYGGYSEPCDKNDIYEFATLFNTILNSVLFSFSIIGNCLVLWILIKYESLVSLTNVFIFNLSIADLILSSWLPLFIVYHRQGWVFGEVACKILNAFFSIGFYSGIIFLTFMTFHRYLSVVDPLSALKAKNPLFGVAASLLSWLISICASIPVIIYKAQVDRNGFIICEYRDNLPHLVSNYQQNIVFLIAFVVIIVCYFSIIKTLRRSRSQRNHKPVKLIFIIVVVYFVSWAPYNIVMLLQSFEKQQLFKSFRDCDFSKNLDYAKSVSEKLAISHCCLNPILYAFVGIKFRQHLKRLLHYRPCKHEEQTTSIRTNSHDHYHNNDGSLY

>xtrCXCR7 NP_001025605.1 Scaffold GL172651.1

MDYMESMNSTEFNLSCSNGDCIILESLSCPNTLNKSALLYTLAIVYIFIFVMGLLANSMVIWLNLQAKTTGYETHLYIFNLAIADLCVLLTLPVWVVSLVQHNQWPMGEMTCKITHLVFSINLYSSIFFLTCMSVDRYLCVSLNGTAGQRRRKIIRRLVCVLVWLVAFVVSLPDTYYLKTVSSPVTNETYCRSMYPEETFKEWLLGMEIVSIMLGFVIPFPIIAIFYCLLAWTLSSSSSSSGDQERRISGRLIVSYVVVFMVCWLPYHAMVILDVMSFLQLLPFSCFLDNFLYAALHITQCYSLLHCCINPILYSFIHRNYRYEIMKAFIFRYSSKTGLTKLIESSKVSEAEYSAVDQIPK

>xtrCCRL1 ENSXETT00000018546 Scaffold GL172950.1

MEEKINTTQRPTTENYDEYGDTTFDYSNYEELCEKKGVRQFAQIFLPAFYAVAFVIGVAGNSLVVAIYAYYKKMKSKTDVYLLNLAVADLLLLFTLPFWATDAAVGWQFGIFMCKITSAMYTINFSSGMQFLACISLDRYFAVTKAPNPQPIRKICWVTCLFVWSTSMLLSIPDLYFSTVKEHNNKHACLPVYPKDTIKQTTVLIQILEIVFCFLLPFLVMLFCYASMAKIVLQTPNIKRSRSLKVLLAVVGVFLITQLPYNVIKFWRAIDIIYALITSCSMSRTIDIMIQVTESLALFHSCLNPILYAFMGTTFKSYISKIAKRYGALRRQRIQSTEEYAMHSENHVEETSSFSI

>olaCXCR1a ENSORLT00000010920 Chr 21

MDDFSSIYEELNFTYNYTEFILDPDTQPCDTFTFPDAVMITVCVFYVLIFLLAIPGNMVVGVVISMNRRTMTPSDFYLLNLAVADILLALTLPFWAASVTVGWVFGDALCKIVTVLQELSFYSSILFLTCICVDRYMVIVRALEARRANRLMASWGVCAAVWALGALLSLPGLFSSSFSSKNFTLYVCAEQYDPRSADEWRLSTRILRHTLGFLLPLAIMVPCYGITIQRLLHIRGGFQRQRAMRVIVFVVGAFLLCWMPYHVTVMTDTFFRAKIVPYKCPERMAVDQAMFGTQSLGLLHSCINPVLYAFVGKKFRRRLGQVVRKMGLKERGSASRSSRSSLSSEITSTVM

>olaCXCR1bb ENSORLT00000022641 Chr 21

LKTCDTQLTLEEAESNFDLTLFNDSYNFTYSDETVTDDSLPCNVTVPGFNSVALMVVYILVSLFSLIGNSLVVFVVCTMKKGRGSTDIYLMHLAIADLLFCITLPFWGTYVHFGWSYGNFLCKVLSGFQEASVYSGVFLLACISVDRFFAVVRATRVLSSNHHLVKVVCSVVWLMAGLLSLPVVIKRESMFAEELNQSICYENVTGDSSDLWQFSLRILRHTLGFFLPLVVMTFCYGRTGVTLLQIRNQQKHKAMRVIMAVVLGFVLCWLPYNVAVLTDTLIRAESLKVTSCDTRYRVEVTLNVTQVLAYMHCAINPVLYAFIGQKFRNQLLLSLYKHGVISKR

>olaCXCR1ba ENSORLT00000008135 Chr 2

MSSVTFTLDSDYFENDTDLPDLNTQPCLLVSLGPAVSLVLSVLLVATSALAIPGNLLVGWVIGTNQRALTPSDVYLFHLTAADGLLALTLPFFAVAFVRGWIFGDFLCKFLNLIMEANFYTSIIFLACISVDRYLAIVHTRGLQQSRRGSCSRLLCAVVWAVGWALALPALFNATIAVTLKDGSETWLCTESFHVGRPTAWRLATRVLRHVFGFVLPLVVMVACYGVTVAKLLHTRGFQKHRAMRVIMAVVVAFLLCWTPYHITLMVDTLLRAELMAPGCGVRGAVNVALGVTNSLALLHSCINPFLYAFVGRKFRTKMNLLLQRKFRRDKMSASRVSRSTSQTSEGAGTVL

>olaCXCR3aa AM305942.1 Chr 16

MSYLLNPQPTFKSSNSTPEAAPVKALNMDVLLDGIFNSTDSFDYGDYTDYISEDNDAAAAASVPVLILIVLSVVLILGLFGNTVLLAALALRRRFWRVSDIFILHLAGADLLLLLTLPIRVAQVAGSSGSFGAFCKICGAVFHINFYCGVFGLLCISLDHYLCTNHAAKWRSLRRPRFAAFCCLFVWISSVLLSVPDCMFLASSKNEDQKLRCDYSYSQTATGSMLASRLFHHTVGFSLPVVFLMLLCSYFLSSLLSKDKDLQRRRRRAVIVILSLVLAFLLFWLPYNITLIRDTYLRKISHRHPKKLYPQDSMASALLITSMFGYIHACLRPPIYLLCKKFRDQVKNLSFSNAEMSGSLWELSVEEPCVQRASVEEMKPMAAQQSPVQAH

>olaCXCR3ab ENSORLT00000016896 Chr 16

TSEYPWENYSYYQDDCCEGGYNCDLNEVWKFEALFMPIVLSLTFAVGIPGNGLLVGVLLRRWRTWSVADIFILHLGIADILLMVTLPLWAVQYADEWKFGLLTCKMAGSLYTVNFYSGSGIFLLVCISLDRYFSLVHATQMYSRRKPWVVHVSCLVVWLFSLLLSIPDWIFLDVAQDRQGRQQCFRNYTIQDMDEWIVTSRSIFHTVGFLIPSVVLIFCYASIFHRLRSGNQNLQKQRACKVIMAVVAVFFVCWTPFNISLFVETIHRRNQNNTCQSNAALSKALKVTQSFGYIHCSLNPILYAFLGEKFQLQLRDIFLGGKFLMHSSLQEPLCGIWNKCVYKKQTNIVCS

>olaCXCR3ac blast search Chr 16

MEDGNDSSEGAYWWHDAFEEYTSEYPLENYSLYHNECCEGGYFCDLNEVRKFEALFMPIVLSLTFAVGIPGNGLLVGVLLRRWRTWSVADIFILHLGIADFLLMMTLPLWTVQYADEWKFGLPICKMAGSLYTVNFYSGIFLLVCISLDRYFSVVHTQMYSRRKPWVVHVSCLVVCLFSLLLSIPDWIFLDVAQDELDRQQCFRNYGIEDMVHWIVVSRSIFHTVGFLIPSVVLIFCYASIFHRLRCGNQNLQKQRACKVIMAVVAVFFVCLTPFNITLFIDTIYSRNPNETCQSSAAPLVKAIRVTQSLVYIRCSLIPILYAFLGGKFRRQLQVM

>olaCXCR3ad ENSORLT00000016916 Chr 16

MEDGNDSSEGDYWWQDAFKEYTSEYPWENYSHYQDNCCEGGDICDLNEVWKFEALFMPIVCSLTFAVGIPGNGLLVSILFGRKRTWSVADIFILHLGIADFLLMMTLPLWAAQYADEWKFGLLTCKMAGSLYTVNFYSGIFLLACISLDRYFSVVHATQMYSRRKPWVVHVSCLVVWLFSLLLSIPDWIFLDVVQDRLERQQCFRNYSLQDMDDWIVTSRSIFHTVVGFLIPSVVLIFCYASIFHRLRSGNQNLQKQRACKVIMAVVAVFFVCWTPFNISLFVETIHRRNQNNTCQSNAALSKALKVTQSFGYIHCSLNPILYAFLGVKFRRQLQDIFRSLGCKVKMRTKLHSISSRRTTMWSESADTSNSIA

>olaCXCR3L ENSORLT00000016881 Chr 16

MGSHTSTTDDYDYGDDYVPGDPMKIGAPCSEEDIYGFAKKYTPTVYSLVFVLAVVGNVLVLCVIRRYKNRNSGACAFSLTDTFLLHLAISDLLLAFTLPLFAVQYNHQWVFGLALCKISSALFSLNRYSGILFLACISFDRYLAIVHAVSSGWKRHTCHAQLACAVIWVICLGLSGVDIAFKQVDEQISPNKETVVLCRLWFAQNDVEWRVGLHLVSVVLGFGLPLLVMLYCYIQIFRSLCNATRRQRRKSLSLIFTLVSVFVICWAPYNCFQLVDSLEKLKVVAGGCHFNKVMDIGILITESIGVSHCALNPLLYGFVGEKFRKELLRMCKDLLGKRSWMEANEWKGKRLRKTTGSFSSAESENTSYSVMV

>olaCXCR4a ENSORLT00000014201 Chr 17

MSYYEHIVLDYDLNDTGSGSGLGSGEVGVDLEEPCGMEHLMTAELQQVFLPVVYSLIFILGITGNGLVVMVLGCQRRSKCSLTDRYRLHLSAADLLFVLALPFWAVDAALADWRFGAVTCVGVHVIYTVNLYGSVLILAFISLDRYLAVVRATDTNSGGLRQLLAHRLVYVGAWMPAGLLAVPDLIFARTQEGGEGSTLCQRFYPDDNAPLWVAVFHLQLVLVGLVIPGLVLSVCYCVIVTRLTRGPLGGQRQKRRAVRTTIALVLCFFICWLPYGAGISVDALLRLEVLPRSCRLEAALGVWLSVAEPMAFAHCCLNPLLYAFLGAGFKSSARRALTLSRASSLKVLPRRRPGASTTTESESSSLHSS

>olaCXCR4b ENSORLT00000025393 Ultracontig 257

MEYFYESIVFDNSSEGILDGSGDFEFPEEAYKEALSRDFKKIFLPTVYGVIFVLGIVGNGLVVVVMGYQKKVKNMTDKYRLHLSVADLLLVLTLPFWAVDAVKTWYFGGFVCVSAHVIYTVNLYSSVLILAFISLDRYLAIVRATNSQATRKLLASRVIYVGVWLPAAFLTVPDLVFARVKSVSSPSFSFRNDSVEMEDSRTICERFYPVESRVVWTVIFRFQHILVGFILPGLVILVCYCIIIAKLSKGTKGQTLKKRALKTTVILILCFFCCWLPYCIGIFLDTLMMLNVVRTTYELQQALDKWISITEALAYFHCCLNPILYAFLGVRFKKTARSTLTMSSKSSQKVNLMTKKRGQVSSVSTESESSSVLSS

>olaCXCR5 ENSORLT00000003082 Chr 13

TRKTRIEATTFNEMYTAFMGEYDNFTDPSMPFDAICDDEELGLQSFYSIVQPVLYSIIFLLGLAGNGLMTTVLLRRRRRLRITEIYLLHLAVTDLLLLLTFPFEMIGVAVGWVFGDFLCMTKGVLENLNLLCGSFLLACIGFDRYLAIVHAIPSMQSRRPGKVHQVCAVLWFICLLLSTPNAVFLSVAKHRNSSLDCYYYRYDIHAHNWVLANRVLHHISFFFSLVVMSYCYTVLVFTLWKSPKREAKKSAVRLALLVTLVFCVCWLPYNVALLIRTMVDLDVLTYDSCRFLTLLNQTAAVTKSLGLSHCCLNPFLYAFVGAQFRSELVNLLSKMGCGRGGVRGQGHSLASISERTTTNSTTF

>olaCCR4Laa ENSORLT00000006580 Chr 16

NNNFPSFVFLFKSSSGHPTTTFSDDATTEYDYDYFLQFETCYYEKLGARFIPAMYSMFFLLGLLGNSLVIWVVVCGARLRSMTDMCLLNLAIADLLLVCSLPFLAYQARDQWLFGDAMCKIVLGVYHVVFYSGIFFICLMSIDRYLAIVHAVYAMKARTLFFGRIAAAVTWTAGFLASFPELIFIKQQTTTNKTEKTDSSSHFWTIFSIFKMNIMGLFIPLCIMTFCYSRIIWKLLDSHSSRKQPIRLVLLVIAVFFCCWVPYNISSLFKGLELLQIYMGCESSNSIRLALQVTEVIAYSHSCLNPILYVFVGEKFRRQLLRLISKTPCLLCQMIKVYIPQNRIFGSTYSQNTSMEERSTAV

>olaCCR4Lab ENSORLT00000006570 Chr 16

ISFTVDDNVLFYLPKLPFSANSQIAFKLNLSVMVDPGLYIMFFLLGLLGNSLVIWVVVCGARLRSMTDMCLLNLAIADLLLVCSLPFLAYQARDQWLFGDAMCKIVLGVYNVVFYSGIFFICLMSIDRYLAIVHAVYAMKARTLFFGRIAAAVTWTAGFLASFPELIFIKQQEEGDRHHCLSVYPDSGAGEDDSSHFWRIFGIFKMNIMGLFVPLCIMVYCYSQIIWKLLDSHSSRKQTIRVVILVIVVFFCCWVPYNITSMVKGLELLQIYTGCESSKAITLALQVTEVIAYSHSCLNPILYVFVGEKFRRQLLRLISKTPCLICQMIKVYIPQERIFGSTYSQSTRMNERSTAKNISETLDQIT

>olaCCR4Lac ENSORLT00000006550 Chr 16

MYDYTNNCDNSSADLQDGSKFFLVLYCIMFGFGLIANCTVLWVLIKHIKLRMMTDVLLLNLVLSDLLLAVSLPLWIVKSHNIGLCKLVTGIYQLGFYSGTFFVTVMSVDRYLAIVHAVAAIRARALRYGIIVSVVIWIVSVIMAAPQVVFASLEKEDFDTSHCHPVYPEETVEFWKKLRNFSENTVGIFVCLPIMIFCYVKILLVLSKSRNSKKDKVVKLIFTVVCVFVACWVPYNILVFLQTLEQLEILDDCQLSNNINKAMHFTEIMALSHCCLNPIIYAFIGEKFRKSLGNALKNHFC

>olaCCR6a ENSORLT00000025241 edited Scaffold 1003

MDYLEEDLAGNFTFDYEEPCVYSLNSSMDRKVLPFVHSIICALGLVGNSLVIITYALYKRAKSMTDLYLLNVAIADLLFVLSLPLIIYNEITSWSMGWLSCKLLRGSYSVNLYSGMLLLGCISIDRYLAIVQARRSFRLRSLSYSRLICALIWVFAVLVSIPTFYFYQRYEPFENSTFFLPNEEENEPDDHHYVCDFKHDDRNLAPYFKVMIPSIQLGVGFFLPLLIMIFCYTSIIVTLMKAKNFQRHKAVRVVLAVVVVFVICHLPYNISLLYHTINMFDVVKCQVADTLKVTQNALQAVAYLHCCLNPVLYAFVGVKFRNHFRRIFRDLFCLSKKYMTQRRFSRVTSDMYMSSIRRSVEGSGDSNLSFTM

>olaCCR6b ENSORLT00000022324 Chr 22

KNKFMNLLNSVTTSAVNMENETFYFESNDSDELCNLGTVQAEVVTKTALHAIICASGLVGNLLVMLTYFFYKRSKTMTDVYLFNLAVADLIFVVALPLIILNEQAGFSLGVVACKLLESAYSVNLFSGTLLLACISADRYVAIVHARRSFGSRSRALTYSRLICSTIWVSALVLTLPTLIFTELFEEKDPITGTSSRKCQLSFNQPDTAKLMKVLVPGFQMAIGFLLPMLVMGFCYSWIAYTLLRAQTQRHKAIRVIVAVVVAFFVCHLPYNMALLIHTTSLFKERSCEAEQIKLYVLNVSKSVAYLHCCLNPILYAFVGVKFRSHFMEI

>olaCCR7 ENSORLT00000003263 Chr 1

IYKGKPPSNALIPSDTVLPQASLCQQRKKGNFSGYFPSVDYSNFPTPCVKDVNRNFRRWFMPTLFSIICLLGVAGNLLVILTVFHFKRLKTMTDVYLLNLSFADLLFALSLPFWAASSMAEWVLGQMVCKSMYTIYKVSFYSSMLFLCLISIDRYFAIAKAVSSHRHRTEAAFYSKVSSTVVWVMALIFSIPEMAYTNVSNNTCSLSNQNSTELKVGIQASQIALAFLLPLLVMGFCYASIIKTLCQARNFERNKAIKVILAVVTVFLVSQVPYNIVLFLSTSVTANGGTEDCEYDNTLMLATDATQGLAFFRCCLNPFVYAFIGVKFRNDLFKLLKEYGCMSQEQFYRYTYARRKSSGANETETSTTFSP

>olaCCR9a ENSORLT00000009611 Chr 17

MAMDYTNFINSTSSPDIFTAMTSKDLLSSDLSFTTVDDYDDYEDNDTDLMCDRSSVRTFRGQYEPPLFLIIAIVGGIGNLAVVWIYLNVRRRLKTMTDMYLLNLAAADLLFLATLPLWAAEASYSWTFGSAFCKLNSALYKVNLFSSMLLLTCISVDRYIVIVQSTKAQNSKMERRRLSQLVCAGVWLLALLLATPELVFAKPAPVESKYYCRMVFPSNIGNRTKILVLSLQVSMGFCLPFIAMVFCYSVIVAKLLKTRSFQKHKAMRVILSVVVVFVVSQLPYNITLVMEAMQATNMTVTNCERVKALDKAGQVLKSLAYMHACLNPFLYVFVGVRFRHDFVRLLHPCFGRPPAKVSSLGKTRSPIPSTRASVMSDSETSQALSL

>olaCCR9b ENSORLT00000015575 Chr 20

MDESTTFMSTSEYPYESETTPGDYDYTGDTGMCQKSWVRDFRGHYEPPLFWIIFILGAVGNIMVVWIYTTVRNRLKTMTDVYLLNLAVADLLFLCMLPFWAVDAIKGWHFGLPLCKIVSAVYKINFFSSMLLLTCISIDRYIAIVQVTKAQNLKKKRLFYSKLACIGVWLFSTLLALPEFIFAQVKTDGNLSNCALVYWDNAFNRTKILVLSMQICMGFCFPLLIMFSCYSVIIRNLLQARNFEKHKALRVIFAVVFVFILSQLPYNTTLIMEAVQAANPNITDCATVTHFDIAGQVTRSLAFTHACLNPFLYVFIGVRFREDLFKMVRARVGGLGKSGISKTPAVQSRPSGMSDTDTTPALSI

>olaCCR10 ENSORLT00000017611 Chr 19

ISLNYIYGFNQIYSCWKSKICPINLFAQLLDRTRYMSFMTCAFCLIFLLGVTGNSLVIATFALYRRLRLRSTTDIFLFHLALADLLLLLTLPLKVADTNLSWSFSESFQKSARAFHAVNTYSGLLLLACISVDRYMVVVRAQEMLRLRHQMHTVGSLAAVGVWFVAVLLSLPEILFAWHYNHDCHSCNEVKMASMGLLIAVFCLTLLTMLACYSVIARVLCESPRHRRGKQWQRQRTLKLMVALVLVFLAFQLPYTVVLFWKMARPICELMLEYSTCTLAYTRCCLNPVLYALIGVRFRRDVWHLLQDAGCACAPGGLLQTLSSVSSPNRSTVAGFSPVSPSNSSSNAVPSNFSFQEPHKCSR

>olaCCR11aa blast search Chr 16

SFFASTTTDYSSYYDGDEGGAPCDMNDIKTFSRGFLITLYSLVFVLGFLGNGLVVCVLVKHWKQSNLTDICLFNLALSDLLFVITLPFYANLSMMGYWTFGNFMCHILSGFHRTGFFSSIFFMIIMTLDRYIVILYSHKVARYRTMRLTIALTLTSWILSACVSLPSFIFTKVSNYSGKQDECYFFPENEDWYHYDLFATNMLGLILPLLVMVACYSRIIPVLVKMKTAKKHRVVKLIISIVGVFFLFWAPYNISLFLNFLLLQQIIPSTCNGDKNLRLAVSVTEAFAYTHCCLNPIIYAFVGQKFMRRALQMLKKFVPI

>olaCCR11ab ENSORLT00000006548 Chr 16

TSLPTGIYNFCDYDSVDVTNTGIVVILYNLVFALGLLGNGLVVCVLVKHWKQSNLTDICLFNLALSDLLFVITLPFYSHVLVKGYWTFGNFMCSILSGFHCTGFFSSIFFMIILTLDRYIVILHSHKVAQYRTMRLTIALTLTSWILSACVSLPSFIFTKVTNDECHLLPENEDWYHYDLFAKNILGLILPLLLMVACYSRIIPVLVKMKTAKKHRVVKLIISIVGVFFLFWAPYNISLFLNFLLSHNIIPQTCDSDKNLRLAESVTEAFAYTHCCLNPIIYAFVGQKFMRRTLQMLKKWLPI

>olaCCR12a ENSORLT00000005485 Chr 17

MDDMDDMDDMNYNLFLDLLNGTDDPTEYLQNELVRLCSKNKVNQFGAKFNPIFYSVNFLLSFFGNWLVVVIICKYEKLNTVTNIFLLNLVFSNLLFASSLPFWATYHFSEWIFGTAMCKIVSSTYFIGFYSSILFLTLMTFDRYLAVVHAVSAAKHRKKLYAMVSSVIVWCISVAASLKELVLRKSWKDPSDGYLCEDSGFPISTIRTWSLVTYYQQFMLFFLVPLIMVLYCYISITVRILSTRMKEKCRAIKLIFVIIVTFFICWTPYNIVILLRAIQSSNPELQPPSCSDAESLDYALYVTRNIAYLYCCISPVFYTFVGKKFQSHFKRLMLTRVPCLKRHISLTSHSTRSTSQGTPHSVYEY

>olaCCR12b ENSORLT00000003345 Chr 20

CDRYISENDTINYNNYDYTDSPEGSNQPCSSQSSNDLGAQLSVLYYLMFIFSLVGNSLVLVIIHRFERLTTVTNILLLNLVISSLIFISSLPFIAAYLKLKKWIFGSAMCKIMGSVYYLGLYTSVLFLTLLTFDRHLAVVYPLNASHIRNRKYAFFSCAVVWIVSAVACIIPMITHNTVNSVGTTLCEQDYGNIPSAIGMKLRTTWFYLQLIMFLIFPVIVILYCYFRIAITVMSSKIVSKFKTVRLILVIVLLFFMSWAPFSILELMEDGTTNCVQKQRIEYGIVVSRNLAYFYFCISPFFYTFVGRKFQNYFRQLLVKRFPALKKYISVNEVSRTNLSTKST

>olaXCR1a ENSORLT00000020838 Chr 4

MVPLASTELSERNDFNFSYDDDYESEVCEKGGVVKFGSFVTPVFFSVVITLSVTGNVLVLVILALYESLKSLTNIFILNLAISDLVFTLGLPFWAYYHVWGWQLPEILCKIVNFVFYTGFYSSILCLTAMTIYRYMVVVCTLSDRCRPKLSTGIFLSFLMWTISVGGAMPSLLHTSIIKIHHKDRESWGCEYSSEWWKHMSTYQQNLFFLFAFAVMAVCYIQILQKVRRTRSRTKSRAVKLVFCIVTVFFVGWMPYNMVIFLRALFPSLGFEDCEQSNHLDYAFFVCRLIAFSHCCLNPVFYAFVGVKFRRHLKSMLQAGLKRERPVEEHFPKEERTSSSLKALRHLVGPGDGGVLPKFKGRTKLFTFNAR

>olaXCR1ba blast search Chr 17

WSFFFLLSRPSMEENRTVYENDDYPYLCELNDVSTVKGTVFALIFIISILSNALLIVALLLYENWKNVTTIFIMNLAVADLIFTTTLPFWAVYHLHHWVFGDFLCQCMTALYFISVYSSVLILTALSVDRLVLVIKKPTDSFRRKYVLGTCAAAWLIGIIASSTNAIKVKVTEYEGSYYCEPSHYVDLGYYAEISLLFLLPFIITVFCYTGIIVAVLRTTVRRKFRSVSVMFCIVTVFFFCWGPYNIALIIGHVYEPTECWKKERKEVILGVCQILAFSHCCMNPPLYMLSQKMR

>olaXCR1bb blast search Chr 17

SRLTMEENRTVYENDDYPYLCELNDVSTVKGTVFALIFIISILSNALLIVALLLYENWKNVTTIFIMNLAVADLIFTTTLPFWAVYHLHHWVFGDFLCQCMTALYFISVYSSVLILTALSVDRLVLVIKKPTDSFRRKYVLGTCAAAWLIGIIASSTNAIKVKVTEYEGSYYCEPSHYVDLGYYAEISLLFLLPFIITVFCYTGIIVAVLRTTVRRKFRSVSVMFCIVTVFFFCWGPYNIALIVGHVYEPTECWKQERQEVILGVCQILAFSHCCMNPLLYMLSQKMR

>olaXCR1bc ENSORLT00000022265 Chr 17

TETMTTFDYVYDYPSEECNRTEVFEFAAISTSVFFSIVVVLSLLGNILVLVILVNYENIRSVTNTLILNLAVSDLLFTASLPFWIYYHLHGWTFGKPTCKLVNWVFFTGFCSSSILLVLMTVHRYVAVMNPLSNIVSAAGFPSVVVTVIVWVVSILIASPSFVYSNVTDHENFKICFYENQDAKLWIFYQQNAFFVVCSLVFIFCYSKILWKLKGSTVQKRNNRTLKLIFILVVVFFLEWAPYNYVIFQMSLDVINKNSGHECDSRTRLDYAFFISRMLAFSHCCLNPVFYVLVGIKFKSHLKKMVKICSSDRNSIPNRRSRLTIMSVTSE

>olaCXCR7a ENSORLT00000022368 Chr 21

MSLSEFEDLWENFGELNFSETFSNITSVEAMVCATAFNRSALLYSMCVLYTFIFVIGLAANALVLWVNIRAQSDSTPRHETHTYIAHLAVADLCVCATLPIWVSSLAQHGHWPFGEVACKLTHLLFSVNLFGSIFFLACMSVDRYFSLLQPREEGAQRRKLIRRGVCLGVWLLALVASLPDTHFLHTVKSTHSDTMLCRPVYPEENHTEWMVGVQLSFILLGFLLPFPVIAVFYALLARAFAHSSSSSSSSPVEQERRVSRRVILAYIVVFLACWGPYHSVLLADSLSQLGLVPLTCGLENVLYVALHLTQCLSLLHCCFNPILYNFINRNYRYDLMKAFIFKYSTRTGLARLIEAPNMSETEYSAVAVENPP

>olaCCRL1a ENSORLT00000022426 Chr 17

PDPSRRRKNYSEMPFLIFFHLLGSVDMDVAEDEDYFYHENISFNFSYDDFPTVCEKEDLRAFVALFLPAAYALCLVAGLAGNALVVGVYAYRKRLKTMTDSFLAHMAAADLMLIFTLPFWAAGAARGWELGAVLCKMASTSYTVNFHCCMLLLACVSLDRYLALARLQGGQQRRGLQGAFSRKHCWKVCLAVWTTAFLLGLPDLIFSEVRQASSRNACLLIYPAWMAPGGTAVLEGAEVLLGFLLPLLVMLVCYWNVCKVLTGLPAERKGKKWRAVRVLLLVAGVFLVTQLPYNLVKAFRAVDSVYLLVTHCGTSKALDQAAQVTESLALTHCCFNPILYAAAGSNFRQDLTRMAKTLGALDVENLSPNTSC

>olaCCRL1b ENSORLT00000012853 Chr 20

MEDYEYDEGNETYDYNDEHSVCDKATVRSFGGVFLPIIYALALVVGLTGNALVVVVYTSRLRLRTLTDVCILNLAIADLLLLFTLPFWAADAVHGWTLGSAACKLMSFLYSTNFSCGMLLLACISVDRYRAVTHSSTGRAETGQRARRQWLLVCVALWALASFLGLPELVFSGVKTSHHRTSCTAFYPRNMARPAKAALELLEVILRFLLPFAVMIVCYTLMGRALTRVPGVRRERKWRALRVLFAVVAVFLLTQLPYNVVKLCRALDIIYVLVTVCDVSKGLDHALQVTESLALTHACINPLLYAFMGSSFRSHVLRAAKHLGERLGKHSRPESKEPAVEIALRTQSQNNSQSDSDDRDTSTFTI

>gacCXCR1a ENSGACT00000003009 Group XVI

NTSHLFEDLGSFYDELNYTYNNDTEFDPNPDTVACDFSIPDAGAVVIGVFYVLIFLLAIPGNLLVGLVIGLSRHPLPPSDLYLLHLAVADLLLAVTLPFWATSLTRGWVFGDAACKLVTVLQELSFYSSILFLTCISMDRYMVIVRAMEARRANRRRVSWALCAAVWAVGALLATPGLLHSAQPSKTSNLTTCGEKYEQGNADRWRLATRMLRHTLGFLVPLAVMLPCYGVTVRRLLRVRGGFQRQRAMRVIVTVVVAFLLCWTPYHVAVMADTFFRAKIVPYKCPARMAVDLAMLGTQSLGLLHSCVNPVLYAFVGEKFRLRLGQIVRKTGLLGRTSVARGSRSSMSSEITSTVMGSGRL

>gacCXCR1ba ENSGACT00000019166 Group I

VLLIIIFLLAIPGNLLVGWVIGTSKQALTPSDVYLFHLTMSDFLMALTIPFSAVHLIQGWVFGDFLCIFLSLVFEANFYTSILFLTCISVDRYLMIVRASESHRTRQAMRRRLLCATVWALGWALALPALFNDVSKLTAERMTCGESFDLGSASSWRIATRGFRHIFGFFLPLVVMVICYSITITRLLRTRGFHKHRAMKVIIAVVMVFLLCWTPYHVTMMVDTLLRADLIPPGCALRRSLSTALFSTHGLALFHSCVNPVLYAFVGEKFRSKMMQVVQSVTNSLLLNCVLLIKESWSAFIGPGGIHAFTRV

>gacCXCR1bb ENSGACT00000003112 Group XVI

LMVTYIGVFVLSILGNGVVVYVIYSMEKGRGTTDIYLMHLAMADLLFCVTLPFWAINAQSGWIFGNFLCKLLSGFQEASVYGGVFLLACISVDRHFVIVRATRLRPFHRLLVKVTCGVVWLVAAVLSLPVAILKESMHDEDLGRTICYENITNESSDRWRVIMLVMRHSIGFFVPLVVMTVCYGWIVVALIHTRNSQKHKAIRVILAVVFAFVVCWLPYNIAVLIDTLTRKKTIAMETCETRYMVEVMLAVTQVLAFMHCAVNPVLYAFIGEKFRKHLLSALYRHRLISRKGSASSVGSIRSRNTSIMIFQQNLK

>gacCXCR3aa ENSGACT00000002330 scaffold_122

LSLLSPPQQMSMEVDFDGLFNPNYTFDEDEDYQYKEDPEPGAGGAAYAPALYSLLLVLGLLGNGLLLALLAKGRRPWSTSDAFVFHLSVADVLLLATLPLWAAQAALRGGWGFEGLLCKISGAAFNVNFYFGIFLLACIGLDLYLSIVRGTRLFARERPRLGHISCLSIWLASLLLTVPGWAFPAAKKDPARATGLCARGHSWPLTDWQLASRVLHHALGFLLPAAALFFCCSRVLLRRRVASGLQERRPVAVFLAPVAAFCLCWVPYNVALVADTARGRAAEPAERDGSLKTALTVTSALGCVHACLRPLLYFGLCRNARKRTLAML

>gacCXCR3ab ENSGACT00000002346 scaffold_122

VSVQSSLMDSAVLIQGDDLAALLDGYDDIFANVSHEESDLCDTNEAGPFEAVFIPALYSVAFVVGILGNGVLLGVLVQSRRSWSVTDTFIVHLGVSDVLLLATLPVWAAQSAQADGWTFGSPLCKITGAVFTINFYCGIFLLACISLDRYLSIVHATQMYSRRKPRVVQVSCLAVWLFSLLLSIPDWIFLEAVEDSGRGQKTQCVRNYLMNASGKVGDWRLASRLLFHTAGFLLPSVVLIFCYSCILRQLRCGSRAPQKQKAFRVIVTVVVVFFLCWTPYNITLMVDTLHSADHNDTCGTRTSLGTAKMITSSVGYLHCSLNPVLYAFVGVKFRRQLLGILRSLGCELKTRAHPQSVTSRRSSIWSESGDTSNSLAI

>gacCXCR3L ENSGACT00000002324 scaffold_122

VTISLTQSAIPTAAVFASVLNEYDYDIYDNATWPTATGRSRAAPCEPEDSYGFAQRFSPVVYTLVFLLAFVGNVLVLCVIRRYRNSQSGGACAFSLTDTFLLHLAISDLLLAFTLPLFATQWAHQWVFGLAACKLSGALFSLNRYSGILFLACISFDRYLAIVHAVKRNAWHAQVACALIWACCLALSGADVAFKQVEEVKTGANRTALLCVVWYTEHPTRWRVGLQLVGMLLGFGLPLLVMLYCYIRIFKSLCNATRRQKRKSLRLIVSLVSVFVVCWAPYNGFQLADSLHRLGAVTGGCRFGHVMDVGTVVAEGLGLSHCALNPLLYGFVGVKFRRELGRMCGGLLGGRGARGMEGWKQRRLRKSTASFSSAESEHTSYSVM

>gacCXCR4a ENSGACT00000016396 Group III

HEGIHIFVNDFNDTGSGSGSGDLPDLEEPCDVEHVMTADLQQVFLPVVYALIFTLGITGNGLVVFVLGCQRRSKCSLTDRYRLHLSAADLLFVLALPFWAVDSALGDWRFGEVTCVGVHVIYTVNLYGSVLILAFISLDRYLAVVRATDTNTGGLRQLLAHRLVYVGAWLPAGLLAVPDLIFARTQEGGEGATLCQRFYPAESAPLWVAVFNLQLVVVGLVIPGLVLLVCYCVIVTRLTRGPLGGQRQKRRAVRTTIALVLCFFVCWLPYGVGISVDTLLRLEVLPRGCRLEAVLGVWLAVAEPMAFAHCCLNPLLYAFLGAGFKSSARRALTLSRASSLKILPRRRPGASTTTESESSSLHSS

>gacCXCR4b ENSGACT00000009661 Group XVI

MNVLNMEIDMYSDYFENSTDNLSDDSVDFDIDFQEPCGRSLSNNFNQIFLPTVYGIIFILGMIGNGLVVVVMGYQKKVKTMTDKYRLHLSVADLLFVLTLPFWAVDAAKSWYFGSFLCVSVHVIYTLNLYSSVLILAFISLDRYLAIVRATNSQDTRKLLATKVIYVGVWLPAVLLTVPDLVFARVQEAYPLNSPFPNQSMEAVDSRIMCQRTYPQKHSVEWTVGFRFQQILVGFILPGLVILNCYCIIIGKLSQGARSHAQKRKALKTTIILIVCFFSCWLPYCVGIFLDNLVMLGVISLSCDLEQAVEKWISISEALAYFHCCLNPILYAFLGVKFKKSAKSALTASSRSSQKVTLMAKKRGPISSVSIESESSSALTS

>gacCXCR5 ENSGACT00000016264 Group I

NSASDIWKFSEVSFYNYGNDMAPSVCDDEGISLRTFQPVLYSLIFLLGVSGNGLMTTVLLRRRHHLRITEIYLLHLALADLLLLFTFPFDVVNAAAGWLFGEFLCKLTGLVQNLNLHCGSFLLACIGFDRYLAVVHAIPRMQSRCPKTVHRTCIALWLVCLGLSVPNAVFLSVKEVGGQVSCFYHDYGIHANNWVLTNTVLNHVCFFIPLAVMSYCYAAVVVTLCRSQKSQAKQGAIRLSLLVTLVFFFCWLPYNVTILIKTMVDLSVISQQACEHYILLLSVLDVTKSIGLSHCCLNPFLYAFVGVRFRQELIHLLCEEGCSNVCSPFLRAQGHSRPSISDRATTTSTM

>gacCCR4Laa ENSGACT00000008555 Group XX

AKQKEKGYHGHNRVCYCFFQWSKSTTLSYTDTVSVTSPEYDYPEEGFDSCAYGRHGANFLPTLYAIFFLLGFLGNSLVIWVITCGVRLRSMTDVCLLNLAGADLLLVCTLPFLAHQAWDQWVFGDAMCKVVLGIYHIVFYCGIFFISLMSIDRYLAIVHAVYAIRARTRSFGMIAAAVTWVAGFLASFPDLIYLKQQPGPNMSQSCFPVYPEDVGHSWMIFSVFKRNVLCLFVPVVIISFCYSQIVWRLLHSQSSKKRAIRLVLTVVAVFFCCWVPYNVASFFNALELLHIYTECESSKAIRLALQVTEVIGYSHSCLNPILYVFIGQKFRRHLLRLINRIPC

>gacCCR4Lac (ENSGACT00000008552) Group XX

MNKSENESYLDYDYNDTCDEVQGPELSDGSTAFLVLYYLLFFFSLLGNITVLWVLLRYIKLRTMTDVCLLNLAVSDLMQATTLPLWTCNDTNLASCKLMTGGYQLGFYSGTLFVTLMSVDRYLAIVHAVAAMRARTLRYGIIASTAIWVISVTMALPGVTFASLEIDVNDNSSQCQPLYPDDSHRFWKLLRNFSENTVGLFLCLPIMIFCYVKILIVLSKSRNAKKDKAVKLIFTVVGVFVVCWVPYNVTVFLQTLQLFLENLDTCEASKSINSAMHFAEIIALSHCCVNPVIYAFIGEKFRKSLGNVLTRYCWSHQSRRAFSHRETTEKETSNTPVKSDYQE

>gacCCR6a ENSGACT00000018265 Group XVIII

MFSSQQNKKTMNDSGDQMFYNDSDDDYWTKLVEPCEDQKNTNMELVVGPYVHSIICILGLVGNSLVIVTYAFYKRTKSMTDVYLLNVAVADFLFVASLPLIVYNELSSWSMGPVACKLLRGSYSMNLYGGMLLLACVGADRYVAIVQARRSFRLRSLRYSRLICGTVWSAALLLSLPTFYFYHRYEPLHIGASVEANRTAEPPHYVCELWFADNFTARTTKVAVPGTQLAVGFFLPLLVMVLCYGAIIVKLTKARNFQRHKAVRVVLVVVLVFVVCHLPYNVTLLYETAGMFQEGSCEGSDVLQAAKTLTQTLAYLHCCLNPVLYAFVGVKFRNHFRRLVMDLWCLGKRYIGPRRFSRVTSEIYVSTRRSVDECSDNGSSFTM

>gacCCR6b ENSGACT00000017544 Group XV

QNMTSQALDYSYYSEEPGGEEPCNLDPNPMEIIAQTYIHSIICALGLIGNTLVIITYIRYKKSKTMTDVYLYNVAVADLIFVVALPFLIYNEQHGWLMGSVACKMLRSAYSINLYSGMLLLACVSGDRYVAIVQARKSFGARPRTLLYSRLICSAVWAFAVALTVPTLLYSERSEDPQATCQLSFKETGTAKLMKVAVPSLQMAIGFLLPLLVMMFCYTSIVCTLLRARSSQRHKAIRVVMAVVAVFVVCHLPYNATLLNHTLSLFKVRTCASEKIRLRVLAVSKSVAYFHCCLNPVLYAFVGVKFRTHFQQIILDLWCFSKKHTSTARSSRATSDTCISGRVSSDGSNNMRSFSA

>gacCCR7 ENSGACT00000023977 Group IX

MAVCTRQCSMCYPEFNTTDYNYSASLDYSDFPELCVTTSNGQFRSCFMLPFYIIICLLGLAGNLLVILTFFYFKRLKTMTDVYLLNLSFADLLFALSLPFWAYNSTTKWRLGLTMCKAMYTIYKVSLYGSMFVLAFISVERYFVIAKAISAHRYRSRALFLSKVSSVGIWLMALIFSIPEMTYTAIINNTCTPYSSYSDMLHVKIEASQIALAFALPLLVMSICYSRIVQTLFQARGFERNKAIKVILAVAAVFLVCQMPYNLLLLLNTVVIAQGRSEDCYYQNTFLYVSDVAQCVAFLRCCLNPFVYAFIGVKFRHDLLKLMKDLG

>gacCCR9a ENSGACT00000003203 scaffold_161

VQLSMNKVASYFNLSSTPDDDYDYTDDLFCDRSSVREFRSRFEPPFFWIIALVGGAGNLAVVWIYLNLRRRLKTMTDVYLLNLAVADLLFLVTLPLWAAEASHGSWSFGSALCKLNSALYKVNLFSGTLLLACISVDRYVVIVQTVRAQNSQAERRSCSRLVCSGVWLLALLLATPELVFAATTEPSGREYCRMVFPAHLGNRTKILVLSLQVSMGFCLPFVVMAFCYSVIAAKLLRTRSFQKHKAMRVILAVAAAFASTQLPYAGVLVMEAAQATTVTLTECEDLKQFDKAGQMLKSLAYLHACLNPFLYAFVSVRFRRDLLKL

>gacCCR9b ENSGACT00000006389 Group XXI

AGTDFDGADFNGTDSSEYDYGSDPTESSGMCDRGWVRDFRGQWEPPLFWIIFLLGAVGNLLVVWIYTTVRHRLKTMTDVYLLNLAVADLLFLCMLPFLAVDSIKGWNFGISLCKIVSAVYKINFFSGMLLLTCISVDRYIAIVQVTKAQNLKKKRLFYSKLACLGVWTFSALLALPEFIFAQVTDRNGQVLCTLVYWNNAFNRTKILVLSLQICVGFFLPLLVMLFCYSVIIRTLLQAKSFEKHKALRVVFVVVFVFVLSQLPHNALLVVEATQAAKTTITKCDVIAAVDIAGQIAKSLAFTHACLNPFLYVFIGVRFRQDLVRIVKDTG

>gacCCR10 ENSGACT00000005828 Group V

CPPQPAMDLSIDYDNDFDSYDFNNSLGNTSNVSYSDDWSSDFCEAKADQEVAIKTFQVCVFVLIFLLGVTGNGLVIATFALYRRLRLRSVTDVFLFHLALADLLLLLTLPLQAADTLGWSPSMALDFVVRTCYAINTYSGLLLLACISVDRYLLVAWAQEMLRLRRRMLTGGRAAAAGVWLVAALLSLPQVLYSGLAGRDSEAYCGLVKCEQVKVATNGAVIAVFCLSLAVMATCYSRIACVLWDGRASRRGKQWHRQRTLKLMVALVLVFLAFQLPYTVLLSRKMAGPFCGLLLEYVTCTLAYARCGLNPVLYALVGERFRNDVTRLVHNSGCRRGLRRAPQSLATNSISVSSPASALLA

>gacCCR11aa ENSGACT00000008548 Group XX

MADYAENVSEEYDYSDYYEDNTNNNSPYSITALRDFGKVFLPTFYSLVFVLGVIGNGLVVCVLVKHRHKTNLTDMCLFNLAVSDLVFVFTLPFYSHYSVVGEWPFGDFLCRFASGSHTTGFLSSIFFMVVMTLDRYMVIMHAHKVAQYRTFRAGIALTAFVWTMSLCFSLPAVFFTKVTNESTGVACSYEPENDAWRLYDIIMKNVLGLGIPLLVMIVCYSRIIPILLNMRSTKKHRVVKLIISIVIAFFMFWAPYNISILLEFLNLSTDCNSYQSLKLSIIVTETIAYSHCCLNPIIYAFVGQKFVRRVLQMLKKC

>gacCCR11ab blast search Group XX

YAENVSDYSDYYDKDNTNNNSPCSTTALMDFGKVFLPTFYSLHRHKTNLTDMCLFNLAVSDLVFVFTLPFYSHYSVVGEWPFGDFLCRFASGSHTTGFLSSIFFMVAMTLDRYMVIMHAHKVAQYRTFRAGIALTAFVWTMSLCFSLPAVFFTKVTNESTGVACTYEPENDAWRLYDILMKNVLGLGIPLLVMIVCYSRIIPILLNMRSTKKHRVVKLIISIVIAFFMFWAPYNISILLEFQKLSTDCNSLQSLKLSIIVTETIAYSHCCLNPIIYAFVGQKFMRRVLQMLKKCGSGFLPSTRENSSHRKSSVMSRSSDATTNFIM

>gacCCR11ac (ENSGACT00000008552) Group XX

SADYAENFSQEYNYSDYYDKDNTNNNSPCSTTALRDFGKVFLPTFYSLVFVLGVIGNGLVVCVLVKHRHKTNLTDMCLFNLAVSDLVFVFTLPFYSHYSVVGEWPFGDFLCRFASGSHTTGFLSSIFFMVVMTLDRYMVIMHAHKVAQYRTFRAGIALTAFVWTMSLCFSLPAVFFTKVTNESTGVACSYEPENDAWRLHDILMKNVLGLGIPLLVMIVCYSRIIPILLNMRSTKKHRVVKLIISIVIAFFMFWAPYNISILLEFLNLSTNCNSHQSLKLSIIVTETIAYSHCCLNPIIYAFVGQKFMRRVLQMLKKCGSGFLPSTRENSSHRKSSVRSRSSDATTNF

>gacCCR11ad ENSGACT00000001738 scaffold_797

LSLSADDAENVSDYSDYYEANNSSPCSTTALMDFGKVFLPTFYSLVFVLGVIGNGLVVCVLVKHRHKTNLTDMCLFNLAVSDLVFVFTLPFYSHYSVVGEWPFGDFLCRFASGSHTTGFLSSIFFMVVMTLDRYMVIMHAHKVAQYRTFRAGIALTAFVWTMSLCFSLPGLFFTKVTNESTGVACSYKPENDAWRLYDILMKNVLGLGIPLLVMIVCYSRIIPILLNMRSTKKHRVVKLIISIVIAFFMFWAPYNISILLEFLKLSTDCNSHQSLKLSIIVTETIAYSHCCLNPIIYAFVGQKFMRRVLQMLKKCGSGFLPSTRENSSHRKSSVRSRSSDATTNFIM

>gacCCR12a ENSGACT00000019274 Group III

FYDEANDTTDPSYVVSETVQLCGKQAVNQFGARLIPAFYFSNFLLSYVGNGLVLLIIYKYEKLCTVTNIFLLNLVLSNLLFAGSLPFWASYHLSEWIFGLALCKLVSSAYFIGFYSSVLFLTLMTFDRYLAVVHAVAAAKSRKKAYAIIASVVVWCISIVASLKELVLQNVSKSPFNGLICEESGYPKITMERWRLVSYYQQFLIFFLLPLCMVMYCYISITVRILSTRMKEKCRAIKLIFVIIFTFFACWTPYNIVILLRAIQITHPSEGDDPCADEDRLDYAMYVTRNIAYLYCCVSPMFYTFLGKKFQSHFRKLIGLYMRACTAHSTPQIETPKGNKVSSVSTFAVVSTVSIVH

>gacCCR12ba ENSGACT00000023896 scaffold_164

IIFLITIWFEKLTTVTNILLVNLVMSSLIFMSSLPFMGLSNWIFGNVMCKIHGTVYYLGSYSSVLFLTLLTFDRHLAVVHSLTASRLRSQRYAAVSCVVVWLVSCLACIKPMILHKAFVDFENTTYCQEYPNEIPGIEGKLLSDFGFYIQLILFLIVPLAVTIYCHVRIAITVVSSKIVTKFKTVRLIFVIVLLFFTSWTPFNILMLMNDEDADCETRQKMDYALEVTRVMAYAYFCISPVFYTFVGKKFQKYFRQLLKKHMQKCTQKNVLQQDDDQRGYDLFGGSGQVHSYRVTF

>gacCCR12bb ENSGACT00000001439 scaffold_223

DEEAKLQAKLYEIHNLITTPSQVFNPECLTLSVLPVPGSRLRLALFGIIFKLQIYLILVQTFEKLTTVTNILLVNLVMSSLIFRSSLPFMGVYLQLSNWIFGNVMCKIHGTVYYLGSYSSVLFLTLLTFDRHLAVVHSLTASRLRSQRYAAVSCAVVWLVSCLACIKPMILHKAFVDFENTTYCQEYPNEIPGIEGKLLSDFGFYIQLILFLIVPLAVTIYCHVRISITVVSSKIVTKFKTLRLIFVIVLLFFTSWTPFNILMLMNDEDADCETRQKMDYALEVTRVMAYAYFCISPVFYTFVGKKFQKYFRQF

>gacCCR12bc ENSGACT00000001129 scaffold_37

CSVESVNLLGAQLSILFYFMFVFSVFGNGLVLLIIHRFEKLTTVTNILLVNLVMSSLIFMSSLPFMGVYLQLSNWIFGNVMCKIHGTVYYLGSYSSVLFLTLLTFDRHLAVVHSLTASRLRSQRYAAVSCVLVCLVSCLCMLQAHVFYTKALVDFENTTYCQEYLNEIPGIEGKLLSDFGFYIQLILFLIVPLAVTIYCYVRIAITVVSSKIVTKFKTVRLIFVIVLLFFTCWILFNILMLMNDEDADCETRQKMDYALEVTRVMAYAYFCISPVFYTFVGKKFQKYFRQLLVKTFPRFKRDVFVCLQNRKITNMKTFYKAFNY

>gacXCR1a ENSGACT00000015563 Group VIII

NTFLAFLKKNLFPRSTNVLSMNDSFNDSQYDRNYDDEVCEKEEVVKFGSIAVPVFFSVVITLSLIGNILVLVILALYENLKSLTNIFILNLAISDLVFTAGLPFWSIYHIWGWLFSKVLCKIVTFIFFTGFYSSVLFLTIMTIYRYLVVVHPQSILIPQRPSTSIYISVVMWIISVGAALPSLLYTTIVSIPHKDNHSLGCEYQDRLWKTISVFQQNIFFLVAFAVMAFCYIQLLWKITRTRSHTRRRAVKLVFSIVAVFFLGWVPYNVVIFLQVLADVGASSDNCGASIDLDYTFHVCRLVAFSHCCLNPVFYVFVGVKFRSHLKSLLQRLVIQTRAEEQPV

>gacXCR1bc ENSGACT00000022730 Group III

YDYESSSYRYDSFSYDNDSSGGDQMCDKTSVIKFGATFTVVLFSVVVILSLFGNVMVIVVLAKYENLKALTNAFILNLAVSDLFFTAALPFWAYNHVHEWTLGEHACKMATFVFYVGFYSSGIFLILMTAHRYVAVMSPLSNVVSTTGSVSVAACVITWAVSTLGAGSSFVATKVDQERCVLASSYWKLWVIYQQNVLFLLSSVVFIFCYSQIMCRLLRPTAQRRKNKTLKLIFALTVVFFVGWAPYNAVIFLQSLNMRPQQEVDSSVLVEMCEASRRLDYAFYVSRLLAFSHCCLNPVFYVFVGVKFKSHLKTFTFKYCQKMFCKDKSFTCCSAFRPQS

>gacCCRL1a ENSGACT00000022518 Group III

TSPLPLSLLSSFPEEEQSTGRYDDYPVLCEKGDVRSFAAAFLPAVYAACLLAGLAGNGLVVAVYAYHKRLRTMTDAFLTHLAVADLLLLFTLPFWAADAARGWELGGVLCKVVSACYSVNFTCCMLLLACISLDRYLAVARVRGREGGRWLRRVVTRRHCWKLCSAVWATAFVLGLPDLILSEVRWLSDRSVCLAVYPASMAQGGQAALEVAEVLLGFLLPLLVMVICYGSVGRALRGLPADVGGRKRRALRVLLIVVGVFVVTQLPYNVVKLYRAMDSVYALVTHCVTSKVLDKAAQVTESLALTHCCLNPILYAFVGSSFR

>gacCCRL1b ENSGACT00000005018 Group XXI

DSYDYTTEHSVCDKEAVRSFGGVFLPVVYAVTLVVGLAGNALVVVVYASKLRLRTLTDMCILNLAISDLLLLFTLPFWAADAVHGWKLGVAACKLNSFLYGTNFSCGMLLLACVSVDRYRAVAQSPAGRPGSAPRVRTQWTLVCVLLWAVAGVLGLPELFFCTVKHSHHRMACTAVYPPGMGRSAKATLELMEVALRFLLPFLVMTVCYCSVWRILSRAAGVRRERKWRALRVLLAVVAVFLLTQLPYTLVKLIRAMDVIYILVTDCEVSKGLDRALQVTESLALVHACINPLLYAFMGSSFRGHVLKAAKHLGQRLGRHPRHAGAEPAVEMALRTPAEPQSQSGSD

>dreCXCR1ba CU855890.1-201 (ENSDART00000073870) Chr 9

MMTDPNSSNHLVDFHEFYYEEFNDTDFSNFTFVPDEKTIPCSSITMASAVNISFSVFYVFIFLLAIPGNVIVGWVIGSNRRLLSASDVYLFNLMLADTLLALILPFSAVNVIHGWVFGNVACKLVSLVKEVNFYTSILFLVCISVDRYMVIVRAMESQKAQRRLCSGVACGLVWVLGLVLSLPSFYNEAFFDKRMFNQTICAERFETDHADEWRLATRIMRHVLGFALPLVVMLSCYSVTVVRLLRTRCFQKQRAMKVIVAVVVAFLVCWTPFHVSTIIDTILRAKVVQFGCTMRTSVEVAMFATQNLGLLHCCVNPVLYAFVGEKFRRRFLQLLHRKGVLERFSLSKSSKSSSLTSEVPSSFL

>dreCXCR1bb CU855890.2-201 (ENSDART00000077260) Chr 9

METATTEFPFTLMTPCPETVKNLNSTVLVVIYIIVFCLSLLGNTVVIFVVFFMDNRRTSTDLYLMHLAVADLLFSLTLPFWVAYLHAGHWPFGTIMCKMISGVQEVTFYCSVFMLACISIDRYMAIVKATQFLNRKLHLIGFVCALVWLCAALLSLPVMVHREAITYDGVEYICEDNVTAESTDSWRMSLRIIRHTLGFFLPLTVMMFCYGFTMFTLCHTRNSQKQKAMRVILSVVLAFIICWLPHNIIEFTDILMRAGQVEETCQLRDNIDVALYATQVMAFAHCAINPILYAFIGKKFRNQLLISLFKKGLFGRNMLSRYGAGSFQSSGSTRQMSVTL

>dreCXCR3aa si:dkey-269d20.3-201 (ENSDART00000103981) Chr 16

MAAPSNMEVELHGLFEKNNSFDYDNYENKELDCQSKAVSDALGVFIPMLYSLGILLGLLGHGLVLAVLWHKWLNCSVMDIFIFHLSLIDSLLLLSMPLWAVDAVKGWIMGSGLCKLAGVLFKMNFYCSMLMLAFISVDCYLSIVHGVQKLSRKKPMVVHGCCLIIWLVCLLLSIPEWIFLKSISDSTDQVKDECIYFYPDDSWHRSSRFPHHVIFGVGTLVLLFCCTSIMLKLQRESMCQQKKMGRKTAIIAVLVLVFLICWTPYSIAFIVNTGARPVHIDPLTGESECEWRQWTATKITAIFGLLHCTINPVIYFCFSKEFRRRSLAVIKFNACESNNNDGSLWDSTAVNVNTTVQEEQGPLQQVNELKPKVQTQQQDT

>dreCXCR3ab cxcr3.1-201 (ENSDART00000028141) Chr 16

MKDFSDYTDLYNYSDYNDNESYGAGAVCTQDSSMYFDSIFKPILYSLAAVVGLLGNGLVLIVLWKKRAGLNVTDIFILHLSLADILLLLTLPFWAVEAVKEWIFGTPLCKLTGAMFRINFYCGIYMLSCISLDRYLSIVHAVQMYSRKKPMAVHCCCMIVWFFCFLLSIPDWILLGANKDSRRQDRTECVNSEALSDFWVLVNRLIYHFLGFIIPAIMMVFCYTSILLRLLLGSKCMQKKRAIHVIVALVLAFFISWTPYNIALMADTIHTNRTDNNQTSCETRTTLDVAITATSTFAYMHCCVNPILYAFVGVKFRQHLLDMLRPLGFKLKGRAGLVSRKSSGWSESVDTSHTSAF

>dreCXCR3L cxcr3.2-201 (ENSDART00000060137) Chr 16

MDNSTTAAEVSAPTDYDYNSTSYDDDNPYAAPCSLTETWNFLGRFAPVAYILVFILALVGNILVLCVIRRYRQSRHSPCSFSLTDTFLLHLAVSDLLLAATLPFFAVEWISEWVFGKVMCKITGALFSLNVYCGVLFLACISFDRYLAIVHAINISWRRKTCHAQLACAFIWVICLGLSMVDMHFRDLVEIPGMNRMVCQIVYSEQYSKQWQIGMQLVSMVLGFILPLLVMLYCYLHIFKALCHATRRQKRRSLRLIISLVIVFVISWAPYNALRMTDSLQMLGVIVKSCALNNVLDVGILVTESLGLAHCALNPLLYGLVGVKFRRELAQMCKAALGPQGCLGLVGWANGRGSSTRRPTGSFSSVETENTSYFSVMA

>dreCXCR4a cxcr4a-201 (ENSDART00000080350) Chr 6

MAYYEHIVFEDDLSADNSSEFGSGDIGANFEVPCDVEVSHDFQRIFLPTVYGIIFVLGLIGNGLVVLVMGCQKKSRTMTDKYRLHLSVADLLFVLTLPFWAVDAAKDWYFGGFMCVAVHMIYTVNLYSSVLILAFISLDRYLAVVRATNSQGPRKLLANRIIYVGVWLPAALLTVPDLVFAKAESSAIRTFCERIYPQDSFVTWVVAFRFQHILVGFVLPGLVILICYCIIISKLSRGSKGTQKRKALKTTVVLIVCFFVCWLPYCGGILLDTLMMLEVIPHSCELEQGLQKWIFVTEALAYFHCCLNPILYAFLGVKFKKSARSALSPSRGSSLKILSKKRTGMSSVSTESESSSFHSS

>dreCXCR4b cxcr4b-001 (ENSDART00000061499) Chr 9

MEFYDSIILDNSSDSGSGDYDGEELCDLSVSNDFQKIFLPTVYGIIFVLGIIGNGLVVLVMGFQKKSKNMTDKYRLHLSIADLLFVLTLPFWAVDAVSGWHFGGFLCVTVNMIYTLNLYSSVLILAFISLDRYLAVVRATNSQNLRKLLAGRVIYIGVWLPATFFTIPDLVFAKIHNSSMGTICELTYPQEANVIWKAVFRFQHIIIGFLLPGLIILTCYCIIISKLSKNSKGQTLKRKALKTTVILILCFFICWLPYCAGILVDALTMLNVISHSCFLEQGLEKWIFFTEALAYFHCCLNPILYAFLGVRFSKSARNALSISSRSSHKMLTKKRGPISSVSTESESSSALTS

>dreCXCR5 CR759774.1-201 (ENSDART00000024213) Chr 18

YICEEKKDPLLLFHTVFQPLIFGVVFLIGLTGNGLLLIVLLKRRHNLRITEIYLLHLAVADLLLLFTFPFAVTQSIAGWLFGNFLCKLVGLANRLNLVCGCLLLACISFDRYLAIVHAIPSLQTRRPRTVHLTCGLLWLLCLLVTIPNLVFLSVEKDNNSTRLSCYYNSHGIHGSNWKLTSHFITHLCFFLPLVIMGYCYTFVVITLRQSHRSLEKQGAIRLALLVTVVFCLCWLPYNLTILMNTLVDLEAMPKLSCPAQDTLDRAVIVTESIGFSHCCLNPILYAFIGVRFRRDLLQLLAKTKCLRVCFSDVRINNLNRVSVTDAVTTTTSSQY

>dreCCR4Laa ccr8.1-201 (ENSDART00000060382) Chr 16

MSSTIALLSIAAQLLLTDMEDSSIPDLNDHTLYISNVNGPVTDQPTTPVLMITDYSYDDYYNSVDPDSLPCVYPAHGASILPVLYSLFFVLGFLGNTLVIRLVLKSLRSMTDICLLNLAIADLLLVSSLPFLAHYARDQWIFGGPMCTIVLSVYHIGFYSGIFFIVLMSVDQYLAVVHAVFALKVRTRTYGFLASLVIWVAAVAASFPELIYIDTTDINNQTLCTSYPTTDQSSYHDSKTNGIFKMNIIGLIIPLSVIGFCYSMILIKLLNVRSSRKQAIRLVVVVMVVFFCCWVPYNIAAFFKALELKRVIPHSCESSKAITLSLQITEAVAYSHSCINPFLYVFVGEKFRKHLFRLLNRTPFSRLQFMKSYVQTTASVYSQTTSLDARSSASV

>dreCCR4Lab CABZ01093075.1-201 (ENSDART00000124693) Chr 16

MSSTAGLLSQNSSRSLIMSSTAGLLSITAQSLWTEMENSSIPDLNIHTSYIPNMDEPVSDQPTTPAHYSYDDYYNSVDPDSAPCVYPAHGASILPVLYSLFFVVGFLGNALVIWVVLMGVKLRSMTDICLLNLAIADLLLVSSLPFLAHYARDQWIFGDHMCTMVLSVYHIGFYSGIFFIVMMSVDRYLAVVHAVFALKVRTKTYGILASLVIWVAAVTASFPELIHLKTTVTNNQTLCASYPTTDQWSYHDSKTAGIFKMNVIGLILPLSVIGFCYSMILIKLLTVRSSRRQAMRLVVVVMVVFFCCWAPYNIAAFFKALELKKVLTHSCESSKAITLSLQITEAVAYSHSCVNPFLYVFVGEKFRKHLFRLLNRTPFSRLQFMKSYIIQATGSVYSQTTSMDERSSVAV

>dreCCR4Lac XP_002664844.1 Chr 16

MCNTEEGSLNPHIKAAIFYIVFVLGLVGNIIVLWVLLKSMHVKNMTNLCLLNLAMSDLLMVLSLPFWALYAQGHYLKTDAMCKAMAGAYQVGFYSGIFFVTLMSVDRYLVIVHAVAVLGAKMLRYGIVASVIIWMVSIGAALPEVIFAEVVKDSESNSCQRHYPDESARKWKLFRNFGENAVGLFISLPIIAYCYLRVLMVVKKTKNSKKNRAIKLILGIVIMFVVFWVPYNVVVFLKTLHEFDMLTSCEPYKIINMAMDVTETIALTHCCVNPFIYAFVGEKFRKYLASAFSKYLRCLKTYQSTPSQSRISENDTSNTAIFSTSPMKILSPKHPYTEVYNHRSGKTPNNNNMPPRMGKRGWDGDVCLEDSMGGHKLINHSGER

>dreCCR6a ccr6a-001 (ENSDART00000122256) Chr 20

MINDSSENYNYGDYYEGAVEPCSVESRQKLENFLRLFIHPIICVAGFIGNSLVIVTYALYKRTKSMTDVYLLNVAIADILFVVALPLIIYSEQHSWAMGNMSCKLLRGIYSVNLYSGMLLLACISGDRYLAIVQARRSFRLRSSTLLYSHLVCAAVWLLALLLSLPTFIFYERYENGLTESTFLFNNNTIMEEIQYVCSFKFESNETARMMKTIVPSSQVAVGFFLPLLIMGFCYSSVIVTLLRAKNFQRHKAVRVVLTVVLVFVVCHMPYNLVLLYHTINLFEQQECSHEEAVALTMTITESLAYLHSCLNPLLYAFIGVNFRNHFRKILRDIWCLGKNYMSARRSSRVTTEMYLSTRRSMDYSNNENGTSFTM

>dreCCR6b ccr6b-201 (ENSDART00000056884) Chr 17

MDLEYSDDYAEPCPLMWYRDHEVTVQTYVYSLICALGLVGNVLVLLTYAFYKKAKSMTDVYLVNVALADLLFVVALPLIIHNERSRWSMGTWACKLLRGAYSMNVYTSTLLLACISGDRYIAIVQARRSLRTRSQAKAYSRIICLAIWFLAFVLSLPTVIYHQEKNQECITIFSEMETARLVKILIPSMQMVFGFLVPLMVMVFCYSWTMVTLLKAQNFQKHKAVRVVLAVFFVFVLCHLPYNVALLVYTSKLFVERSCGEEQVTLMTLSVSRTAAYLHCCLNPILYAFIGVKFRSHFCQILRDLRCLGKRYIYSGRSSQQTSDLYVSATKTVAEQNGINDNPSSFTL

>dreCCR7 ccr7-201 (ENSDART00000065441) Chr 6

MHAYTVFCPVLLIWSCHIKKSWSNMTEHQMGEKATTEYDYTTGTVDYDIYEQSCNKTNNRTFRAWFLPTIYTIICLLALMGNFLVILTYLYFKRLKTMTDVYLFNLAMADLLFAISLPFWAASFMSTWHLGLYPCKAMFTIYKVSFFSGMFLLTCISIDRYFSITKAVSAHRCRSSAVYYGQVSSLVTWVIAIVFSVPDMVFAEINSRGTCSANVNYQDYYVKMVISQMVLGFIIPAVVMGFCYICIIKTLMQAKNFERNKAFKVIIAVVVVFVFSQLPYNVVMGVSVQQTTDCAKDNTRLFALDVTMAVAFMRCCVNPFLYAFIGVKFRNDLLKLLKDLGCLRTSHLMYTHCGRRRSSVGLDTETTTTFSP

>dreCCR9a ccr9a-001 (ENSDART00000077511) Chr 2

MMNVTELITSLTPQTSEYEEYGDFSTTSDYGDTDDGFCPKSKVREFRMYYEPMLYWMIVILGAIGNTLVVWIYTHFKNRLKAMTDVYLLNLALADLFFLCTLPFWAADSIYGWAFGSGLCKVVSAVYKINFFSSMFLLTCISVDRYIVIVQTTKAQNSKRSRLLYSKLICVLVWLLAALMSIPECLFARSKEDDESNTFCTMVYWNNENNRTKILVLALQICMGFCIPLVVMIFCYTNIIRTLLKTRNFQKHKALRVILAVVAVFVLSQLPYNGMLVFEATQAANMTVTNCTESIRFDIAGQVMKSIAYMHSCLNPFLYAFVGVRFRKDVIRIFQNIGCVSNLKNSKLAGIPHRSSVMSDTDTTNALSL

>dreCCR9b ccr9b-001 (ENSDART00000098747) Chr 24

ADCCESQREQSMDISTTSEYESGAFNFTDFTNLITDTDDYEPELDGLCSKELVRQFSKNFEPPLYWIIFVVGALGNLLVICIFTTVRNRLKTMTDVYLLNLAVADLIFLGTLPFWATNAAQGWVFHQVICKGVSAAYKINFFASMLLLTCISVDRYIAIVHVTEAHNYKNKRMLHSKITCAFVWLASCILALPEFIFAKVKNIEPQHNSCVMVYSIMDNNRTKVLVLALQICVGFLVPFMVIVLCYSVIIRKLMQARSFEKHKALRVIIAVVAVFVFSQLPLNGYLIIEAGQANNATITDCEVMQRLDMVGQIVKSLAYTHCCLNPILYVFIGVRFQKDLLSLLQCMSCGLGSVENCKLQDTNKKPSVMSDTDTTPVFSL

>dreCCR10 ccr10-001 (ENSDART00000059507) Chr 24

MGSFDLTTEDYSLNYDDDDDYNFTTDPSAFFGKELCEASPEQERNITIVQTTAFLVVFVLGVIGNGLVIATFALYRRLRLRCMTDVFLFFLALSDMLLLLTLPLQTVETLIGSWEFGEPMCKLNRGMYAINTYSGLLLLACISIDRYLVVVCTRSMRKRSSGTLFYSVLSALSIAVISIMFSLPDLSFSSVDNVLNSNLSSCDMKVSEVKWKLWAQIAKIAGFCIPCITMIVCYGAIGHVLIHTGGKGWRRQRTLWLMALLVVLFLLFQLPYTVVLLIKISTPTPTLCSDWTTLHIMEIVTRNLAYVRCCLNPLLYALVGVRFRNDIIRLLTDAGCVCKCVSQKAPHLGNGSSITPSSPAPTTLTHIPSTYQPGKNTSAPDTPAANTAQTFFFPTPVSGKVTSVISWCHQII

>dreCCR11aa BX511173.3-201 (ENSDART00000104176) Chr 16

LKNKNILSSIKHTYIISLHLFYGLKLGFLCTGFNKYYNYNETEHLAPPCNDAKTKAFSEVFLPILYSIVFIIGIIGNGLVVWVFIRCRQKSNMTDVCLLNLALSDLLFLVSLPFWAHNAMNQRTFGKFMCHTITGLFMIGLYASIFFMVLLTLDRYAIIIHPNCMFFRNRSAKLGLALLVWMLSLLASLPNIIFANEKFDLNHIKSCQPDFPDNTSWMSFTYINMNLLSLIFPLIILIFCYSRIISTLFRMKSEKKPKLVKLILAVVTVYFLFFTPYNIVIFLLFLQRMEYFFSCEWHIDLSLAMQWVETIALSHCCLNPIIYAFASQQFR

>dreCCR11ab BX537113.2-201 (ENSDART00000104183) Chr 16

CTGFDDYYNYNETGHVAPPCNNGNAKAFSEVFLPTLYSIVFIVGFIGNGLVVWVLIRHRQKSNMTDVCLFNLALADLIFLVSLPFWAHNAMDEWILGRFMCHTITGLFMIGLYASIFFMVLMTLDRYAIIVHAHSVFSRNRSTKMGLALASLVWMLSLFASLPNIIFANANNGTNSKSSCRPDFPDNTSWMSFTYINMNLLSLIFPLIIMSFCYSRIIPTLLSIKSQKRHKVVRLILAVVAVYFLFWTPYNIVMFLMFLQRMEYMFSCEWHNGLSLAMQWVETIALSHCCLNPIIYAFAGEKFRRAVLKVLKDQFPMCFKQCASFSQQLSERRSSIFSRSSEISSTQ

>dreCCR11ac BX537113.1-201 (ENSDART00000104162) Chr 16

MTEEPSTVAATKTDYSDYYNEEGDFEQPCNNGQTKAFSEVFLPTLYSIVFIIGFIGNGLVVWVLVRYRHKSNMTDVCLFNLALADLLFLVSLPFWAHNAMDEWIFGRFMCHTITGLFMIGLYASIFFMVLMTLDRYAIIVHAHSVFSRNRSTKMGLALASLVWMLSLLVSLPNIIFAKDKNETNSKISCGSDFPKDSSWMPFTYLKMNLLSLVFPLIIMIFCYSRIIPTLLSMKSQKKHKVVRLILAVVAVYFIFWTPYNIVMFLMFLQKMEYMLTCEWHNGLSLAMQWVETIALSHCCLNPIIYAFAGEKFRGAVIKVLNDQFPMCFKQCASFTHQLSERRSSMFSRSSEISSTQIA

>dreCCR12a ccr12.3-001 (ENSDART00000145888) Chr 2

MVDTNWSDFESFFNETYSEETYEGSIVTAEVILCKKADVIRFSAAFLPAFYYINFLLSLLGNGLVLCIIYKFEKLSTVTNIFLLNLVISDLIFASSLPFWAVYHKSEWIFGKNLCKFVGSCYSVGFNSSILFLTLMTFDRYLAVVHSIAAAQSRRMAYAFGSSAAVWVVSIVASIKDIVLYDVMKTEDGLLCEMTGYNQTFLTKWELIGYYQQFFLFFMVPLIIVLYCYVRITIRIMYTRLMEKCRAVKLIFIIVFTFFICWTPYNVVILLKAIKTYFKVQNDCSNALDYALYVTRNFAYLYCCISPVFYTFLGKKFQSHFLKLLSKRIPCLKIDAMWSTQSSKNTSFRSPNTDF

>dreCCR12ba CU467964.1-201 (ENSDART00000066867) Chr 24

FATTDAKISTKMEASNSTPLPYEDYSPGVWQCSPIGTNDFGRTFLPPFYYIIFIISILGNGVVLLVVHKFEKMNTITNIFLINLVASNIIFTLTLPFYAVYHSSEWIFGEPMCKLVTSAYYLGFYSSILFLTLMTFDRYLAVVHCIMANSQRRSIYAASLSVAVWIISLLASLEYLIYFTVEESQVGGLSCNDPRVGEWKTFALYKQFVLFFLFPLVVFVYCYSRITLTVMHTRMVKKHHTIRVIFVTVLMFFVFWSPYNIVLVMREYKDPNDCNSNLPHAVYVTNNVARLYFCINPVFYIFLGRKFQNRVR

>dreCCR12bb ccr12.2-001 (ENSDART00000066872) Chr 24

QKHCQSCSQVVEITTKMETSSTTPDYYENFSPETSESGTCSSIDINEFGRAFLPMFYYIIFTVSILGNGVVLLVVYKFEKMNTVTNIFLINLVASNIIFTLTLPFQAVYHSSEWIFGEPLCKLVTSAYYLGFYSSILFLTLMTFDRYLAVVHCVVANKNRRSVYAASLSVAVWIISLLESLEYLIRFTVEESQMDGLLCEDSNENQWRTFSFYKQFVLFFLFPLVVFVYCYSRITLTIMRTRMIGKHRTVRLIFVIVLMFFLFWSPYNIILMITEHKDLEHEDCNSVLPYAVYITNNIARLYFCINPVFYTFLGRKFQNHVRGVLMSHTSCLTSQMSVSGSSRSLA

>dreXCR1aa xcr1a-201 (ENSDART00000077124) Chr 23

MTLNNTTYDYDDDYEDQLCRKDDVVKFGSIIIPLFFSIVVVMSCIGNVLVLIILALYESLKFLTNVFILNLALSDLLFTFGLPFWASYFIHGWTFGEIGCKAVKFLFYVGFYSSVLFLTLMTIQRYMAVVHPLSDWEKCRCFSVAPIIIWMMSGTAALVGAHYSKILKDLNNTYCEYESIKVKSGIAYFQNAFFFAAFLIMGFCYCRMLQTITNARTSKRHKTVRLIFSIALVFFIGWAPYNIAMFLRSLTDQNIPPFTICEVSKGVDYAYYLCRLLAFSHCCLNPVFYVFVGVKFRNHLYMILEKIIPVRAEWQKISHKVQCTERKLEHFNRIQPTVSSSESNVAF

>dreXCR1ab CU657980.1-201 (ENSDART00000077122) Chr 23

MTDENTTYDYDDYYEDQLCRKDDVVKFGSIIIPLFFSIVVVMSCIGNILVLIILALYENLKSLTNVFIIHLSVSNLLFTFGLPFWASYFIHGWTFGEIGCKAVKFLFYVGFYSSVMFLTLMTIQRYMAVVHPLSDWEKCRCFSVAPIIIWMMSGTAALVGAHYSKILKDLNNTYCEYESIKVKSGIAYYQNAFFFIAFIFVWFCYGRMCWAITKYHNRETHRTVRLTFLIALLLFFGWTPYNIAMFLRSLTDQNIYPFTLCEVSKTVDYAYYICRLIAFSHCSVNPALFIFADANFCKHL

>dreXCR1ac CU657980.2-201 (ENSDART00000130476) Chr 23

MTEEYTTLDDEPEDELCRKDAVIKFGSIVIPLFFALLVVFSCVGNTLVLVILVLYEKLKSLINLLILNLALSDLLFTFGLPFWASYFIHGWTFGEIGCKAVKFLFYVGFYSSVLFLTLMTIQRYMAVVHPLSDWKKHRCFSVAPFIIWILSGTAALSVSLRSKVLIHDDNLYCEFDSIKVKHVAVYSQNIFFLMAFCIMGFCHARMFQVITQSQSRRRRKTINLIFCIGIAFFIGWVPYNIVMLLKTLQDYALPPFLICSVSIHLDYAFYACRLLAFSHCCLNPVLYTLIGERFQKHLRTLPKKICLK

>dreXCR1ba CABZ01053221.1-201 (ENSDART00000081711) Chr 2

MDPVTTNNYVGNSTTPDYYNYYEPLIPMCQADDYKTTTGICYAIIFFISILGNGFLIGALTCYEDLKRATNLFMFCLALFDLVFTLTLPFWSTEFLHHWVFGDVACKIMTGAYFVGIYGSLILLTAMTLDRFVVVVVRSYWLTRSRRLKCAKGACIGAWIISLIACLRDSVSAKVQNIHIENYSCQSISTTDETFGYYAQLILLFFVPFAVIVFCYAKILMTFMSTSTKQKSRTVILVLCIVVAFFICWGPYHIIVVLMSIYDFDACKHYELHIAFIVCRILAFSHCCINPALYIVRGKYRKLLCSLLFCSPDLRHSRSYRGPTDPSGFREH

>dreXCR1bc CABZ01053222.1-201 (ENSDART00000074943) Chr 2

MDLQTVTPKQNENSTYDYNDDYTDEACNKMNVIQFGTIVSPIIFTIVVMFSCVGNILVLCVLVKYENLRSLTNTFLLNLAISDLIFTVGLPFWAYYYVNGWTLGDHACKAVNYVFYTGYYSSIIFMTVLTIHRYVAVVHPLSVVMSRKSIHCYATSIVVWIISLSAAIPQAMFKTVVRNPIDTQSEASDVIKLCDFDGQINWKLWSTYLQNGFFIVAFLIIAFCYTVILTRLLRPTSHTRKKTVHLIFFIVLFFFLGWGPFNVAIFLDSLISWGISPFNECEVSKSIDYLMYVSQMVAYSHCCLNPVVYVFMGIKFRNHLKKMLWTLCKNNVEPPNRNSRIIYSNGEEISMY

>dreXCR1bd CABZ01053219.1-201 (ENSDART00000123781) Chr 2

MISSFLSDGQRRFLKEQFTTPNECTDDMMHEDMSYEVFNFSYDDYYLTYEYPLIVLEEGNSLFGKISAICYSLIVCMGLPGNLFLLWLVLKKVGLSSSADCLLLHLTISDLVFTLTLIPWTIYHIRGWIFGFAACRLFSWFIFLGLYSYMLFLTVMTVHRYIAVQHPVFASSVGNRGRLYAHVSSAVVWMISLGFSLPEMIFSETLDRYDGVQCVSYSRSEFWILFGYFSQIILFFLLPFLVIALCYARMGFTIHQSRIRSRNRHHAVCLILSIAIGFFICWAPYNIFLFIHSLEFLGVVELRETLSDTVYCVTHILAYFHCCLNPLVHIFGGKKYRNFLPWSRRVRWLPQSFSNEMFSSQSSFSGQFHL

>dreCXCR7a si:dkeyp-74a11.11-201 (ENSDART00000090414) Chr 9

MRTKPWINIAHSTSIKGKMTEIQSSSPNEKLGLSASELTEFFAMWEELNFTDSNNNSSRVEAQMCPASFNRSALLNAMCTLYAFIFVVGLAANALVVWVNMRSQRHYHETHMYILNLAVADLCVVATLPVWVSSLAQGGHWAFGQAACKLTHLLFSVNLFASIFFLTCMSVDRYLSVVRFREISHRRGRQVRRLVCAITWLLALFASVPDTYFLRSVKSQYSHVTLCLPVYPEDNPLQWMVGIQLSFVVLGFAIPFPIIAVSYALLANTLASTSTHSPSNGDQDRSVSKKVILMYIVVFIACWAPYHAVLLADALAMLGVLPLGCSSENGLFVALHLTQCLSLLHCCVNPVVYSFAHRHYRYDLMKAFIFKYSTRTGLARLIDGSQGIETEYAMVENNPPTVT

>dreCXCR7b cxcr7b-001 (ENSDART00000063665) Chr 6

MSVNVNDFNDILDALGELNFSTLDDNVSHVEVCHSTFSQRALLYALSVLYIFLFIIGLAANALVVWVNVRAERTRYETHLYILNLAIADLCVVATLPVSISSLLQLGHWPFGGAMCKITHLIFSVNLFSSIFFLTCMSVDRYLSVKLFGDTPSQRKRRTRQIICVGVWLLALIAALPEIYFLQAEKSDHSDAIVCKAVYPVESMKEWTVGIQMSFFMLGFAIPFPVIAVFYVLLANTIHPSVDQERRISRHLIFTYIVVFLVCWLPYHGALLLDTLAFLNVLPFNCTLENALYAALHLTQCFSLFHCCANPIIYNFINKNYRYDLMKAFIFKYSTKTGLARLIDASHVSETEYSAVENQG

PL

>dreCCRL1a ccrl1a-201 (ENSDART00000111070) Chr 2

MSQSKQTVLQEEQTETIHSAFRQSFNPFERMENSEEHFYDYPEYENSSSNFSYDDYQTICEKGDVRSFARIFLPAVFGLSLVIGLAGNALIVAVYAYCKQLKTMTDTFILHLAVADLLLLLTLPFWAVDAVHGWQLGITICKLVSGLYTINFTCSMMLLAYISMDRYLALSVGSRNQGLGRVFQKQHCGKLCVVVWMAAFLLGIPDLVFSTVRELPHKKSCLAMYPSDMALRAKASLEMVEVVIGFLLPLLVMLFCYTRVGRALLKLSEDRKWKKWQYIRVLLAMVGVFLVTQLPYNVVKFCRALDIMYTFVTHCGVSKKLDWATQITESLALTHCCLNPVVYTFVGSSFRQHVLKCAKDFGDRGRRLAHAREQQEVNISLNSHSHSRDTSTFSI

>dreCCRL1b ccrl1b-001 (ENSDART00000058703) Chr 24

LLWTADSSFLNGVSHHKQITMGVLLEYEHDYQYHDHDNDSNDSAYDEDYFGDLHTVCDKQEVRSFAGVFLPVIYTLALVLGLAGNSLVVFIYLSHKRLRTLTDVFILNLAFADLLLLFTLPFWAADAVNGWQIGTAACKITSALYTTNFSCSMLLLSCISIDRYRALAKGSAHTPARNNSRKHRIIMCLVVWGIAIVLGLPDMVFYTVRVQHSSERYTCRAVYPHSMARAAKATLEILEVSLSFILPFLVMMFCYCRVGVALSQAATAGVHGGRRWRAFRVLIAVVGVFLLTQLPYNVVKLIKTLDVIYILVTECDVSKNLDLANQITESLALTHCCLNPVLYVFIGSSFKMHIIKLAKHWSQTGRGYRHGNEQPAIEISLKSATQTHTNSSSGNEDTSTFTI

>tniCXCR1a ENSTNIT00000003044 Chr 2

SSDPSPSSFSIDFGSLYEELNFTYNDSGYDLNPDTQPCGSFSIPDVAMIAVCVFYILIFLLAIPGNMVVGLVISLSKQALPPSDLYLLHLALADLLLAITLPFWATSVTKGWVFGDAMCKIVTIIQELSFYSSILFLACISVDRYMVIVRALEVRRANRQKVSWCVCLAVWVVGGLLSLPGFFSSSFITNNSSQIVCAEQYDPGSADMWRLVTRILRHTLGFLVPLAIMLPCYGITIKRLLRIRGSIQRRRAMKVIVFVVVAFLLCWTPYHLAVMTDTFFRAKIVPYRCPARMAVDQAVFATQSLGLLHSCVNPVLYAFAGEKFRKRLLQLIKKVKFNERVSVSRSSRSSLSSEITSAFM

>tniCXCR1ba ENSTNIT00000011332 Chr 3

TLSCEVQPMDPTAALITCFFLIIIFILAIPGNLIVGWVIFTRRQMLTPSDVYLFHLTIADGLMALTIPFLAAALVKGWIFGSFMCKFLPFVVEANFYTSILFLACISIDRYLVIVRANDNVRSRRRLCSWFLCLAVWALGSTLALPALFNDTAKLESDPQIMICSKNFDLGSATSWRLATRLFLHIFGFFLPLVVMIACYSIVIVRLLRTRGFQKHRAMKVIISVVVAFLLCWMPYHVTLMVDTALRTDLIPFDCSVRMAVTTALDVTNSLALVHSCINP>tniCXCR1bb ENSTNIT00000011927 Chr 2

LQVPMAPAYLDENPENFTSYPYDDDGSGPCNLTVRGFNPLGLTITYMLVFVFSTVGNGVVVWVVCWIAKRRTSTDIYLTHLAAANLLFGLTLPFWAVDARSGWIFGTALCKLLSGLQEASEYGGVFLLACISVDRHLAIVKATRVKSSHGPVVKATCAAVWLVAAVLSIPTAVQRRHMGTEDPDRDICYEDVMDESSNRWYVLMHIARHVLGFFLPLAVMAVCYGSTLVTLYHTHNRQKQKAIRVIVAVVLAFIVCWLPYNVVLLIQLLIQSSLVEVESCGTRDRVWVALDVTKVLAFVHCAVNPVLYAFIGVKFRNQLLSVAHKWGLVGSRLVATYRGSSASSGGSARSRNT

>truCXCR3aa ENSTRUT00000009389 Chr 8

MGIKVVADGIFKENDTQDYDYDYKDESELGADTAVWIPVLHLVVLVAGLLVNLLLLTVLFRKRRSWSITDIFFLHLCLADVLLLLTLPLLAAQAFQPCGWCFELGLIPCKISRALFNVSFYCGIFLLVCIVLDRYLFYSRSTRIYSKSRPGLAHGTCLCVWICSLVLVIPIWIFVVTLNDPSGEKVLCVENFSASSGVGHLASRVLHHTLGFLLPAAILIICCCCVTLRPQRGSKQPHKRRAFTVVLWLVVVFLLCWTPYNITLIVDTVKSRAGKMGHSLETPLKMTFLLGYIHTCLRPLLYLSLSAGFRAQALALLRCAPPRPVGSLWGLGLGEDGQTEQSHKDEEQEQEQMTRDHHMRVSAHC

>tniCXCR3ab ENSTNIT00000011213 Chr 8

IPQMGNVQKITGDELKEIWDDLADYFENATTNESCCAAGYVCDPTNGQEFKATFILVLYLLAFVVGVVGNGLLLLVLVQNRRTWSVTDTFILHLAMADVLLLVTLPTWAAQAAQDEGWTFGTPLCKITGAVFMVNFYCGIFLLGCISFDRYLSIVHATHMYSHRKPWAIRISCMAAWLFSLLLSITDLVFLEAVSNDRLNRTMCVRVNSTQVNWQLSSRLIYHIVGFLLPSAVMIFCYTCILRRLRCSSQGLQKQRAIRVIIAVVAVFFLCWTPYNIVLLVDTFYSPSGSDKCGMHTSMEKALTVTSSVGYLHCSLNPILYAFVGVKFRRQLLAVLRSLGCKLKTTTRLHSTASSRRSSFWSESAETSKSVA

>tniCXCR3L ENSTNIT00000011214 Chr 8

EDYENVTPATEVLHAAPCKQTDIYSFAQKYSPVVYCLLFILAVVGNLLVICVIRHYRSSQRGRSCASSLADTFLLHLAVSDLLLAFTLPLFAVQWAHEWVFGLTVCKISGALLSLSRYSGIFFLACISVDRYLAIVHAIGSGCKRNTFHAQAVCAIIWVVCLALSGADIAFKQVGDAATFGHYGLLCQAWIPRDTPHWQVVLQMISAFVGFGLPVFIMLYCYVCIFRSLCTATRRQKRKSLHLIVSLVSVFVICWAPYNCFQLADSLQALGVVAGGCLFGRVLDIGTLVTESLGLSHCALNPFLYGFVGVKFRRELAKMCKEMLGRRGLLGMEEWRERRSRKATSSLGSGTSENTLNSVM

>tniCXCR4a ENSTNIT00000010907 Chr 15

MSYYEHIFLEYDYNDTGSGSGSASGELEGDLDEPCGVEHVMTADLQRVFLPVVYALIFIVGITGNGLVVLVLGCQHRSKCSLTDRYRLHLSAADLLFVLALPFWAVDAALADWRFGAATCVGVHVIYTVNLYGSVLILAFISLDRYLAVVRATDTATGGLRQLLAHRLVYMAGAWLPAGLLAIPDLVFARTQEGGEGATLCQRFYPEENAPLWVAVFHLQLVLVGLVIPGLVLLVCYCVIVLRLTQGPLGAQRQKRRAVRTTIVLVLCFFLCWLPYGAGISVDALLRLELLPRSCRLEAVLGVWLAVAEPMAFAHCCLNPLLYAFLGASFKNSARRALTTSRASSLKILPRRRPGTSITTESESSSLHSS

>tniCXCR4b ENSTNIT00000001795 Chr 2

LPFFQISFSVGLDNVTDNSSEMEPGDFGDLQLEERCDLALGNNFNKIFLPTVYGIIFILGIVGNGLVIAVMGYQKKGRNMTDKYRLHLSVADLLFVLTLPFWAVDAASNWYFGSFLCVSVHMIYSVNLYSSVLILAFISLDRYLAVVRATNSQATRKLLASRVIYVGVWLPAAVLTVPDLVFARENMETAQSRLICQRIYPEETSLIWIAVFRFQHILVGFILPGLVILICYCIIISKLSQGAKGQALKKKALKTTVILILCFFSCWLPYCVGIFLDTLMMLNVVSSPCGLQHAVEKWISITEALAYFHCCLNPILYAFLGVKFKKSAKNALTSRSSQKATLMTKKRGPISSVSTESESSSVLSS

>tniCXCR5 ENSTNIT00000019618 Chr 16

YCGDEDGTLRSFMATYEALFYSCLFLLGVVGNGLMVTVLLSRWRLLRVSEIYLLHLAVSDLLLLATFPFSILESITGWLFGDFSCKLTGLARQLNFLCGSFLLACIAIDRYLAIVHAISSLQHRRQRSVHLTCLSLWLVCFSMSVPNLVFLTATDSTHASRTSCSFYNYGIHAHNWVLATRALEHAVFFLSLVIMAYCYSTVVVTLVKSPRGPTQQGAIRLALLITLVFCVCWLPYNIASVLRTVDDLRTVDDLNRGVRNCESNLLLQAALGVTKSLGFSHCCLNPFLYAFVGVRFRNDLLHLLSKLGCGCGCPQRLMKTQFNKAALDSHRSSSCTAF

>tniCCR4Laa ENSTNIT00000022558 edited Chr 8

FLPALYSLFFLFGLLGNLLVIWVIVFGTRLRSMTDVCLLNLALADLLLVCSFPFMAPQPSDQWAFGDAMCKMVLGVFNIFYCGIFFISLMSIDRYLAVVHAVYTIRARTRSLGITAAVVVWIAGFLASFPDLLFLKLQKVSSDFYCFPEYPKDTVNTSSHYWSVFSLFKMNILGLFLPLVIMLFCYSQIIWRLYHSHSSKKQAIRLVLIVVTVFLFCWIPYNITSFFKALELLGIYGTCDFSKGITLSLEITESIAYSHSCLNPILYVFVGEKFRRHLFRMINKIPCRICQVVRVCLPQDRIHGSVYSQTTSLD

>tniCCR4Lac ENSTNIT00000022557 Chr 8

MNTSGVNFSLYPDIYDYDYNSTCDQDPNPVLSDTVLRLFYCVVFGFGLIGNSTVIWVLLQFIKLKTMADVCLLNLALSDLIFAVSLPLWAFNFQILALCKVMTAIYQVGFYSGTLFVTLMSLDRYVAIVHAVSSMRARTLHRGIIASISIWAVSIIIAAPQVKYASLEIDPENNLSQCQPLYPEDSMEFWKMRRNLSENIVALFVCLPIMIFCYVKILIVLSKSPNSKKDKAIRLIFAIVCVFVMCWVPYNVTVFLQTLQIFEILVSCSASRSISLTMSFAEIIALSHCCLNPIIYAFAGEKFRKSL

>tniCCR6 ENSTNIT00000007608 Chr 14

PCSHQLTQEAELLLGPYVHSLICLLGFLGNSLVIATYAFYKRTKSMTDVFLLNVAVADLLFVLALPLLVYNQLSSWSMGTAACKLLRGSYSVNLYSGTLLLACISADRYVAIVQVRRSFRLRSLSHSRLICVLVWTAALLLSVPTFYFYHRYQPSHSQDEFLDGDNTSQSYVCEFQFLDNSTAWRTKVAVPSTQLAVGFVLPLLVMASCYSAVLLTLLRARNFQRHGCHLPYNLALLYETTTMFQLQSCERSDTLQLAKALTQTLAYLHCCLNPLLYAFIGVKFRNHFRRVLWDLWCRRRGSAPPRPFSRATSEVYVSGRRSQDGSSDNGSSFTM

>tniCCR7 ENSTNIT00000017377 Chr 18

TADYSTFPTVCVKELNRQFRRWFMPTFYSVIFFLGLAGNLLVILTFFYFKRLKTMTDVYLLNLSFADLLFALSLPFWAANTMTKWVLGEEMCIAMYTVYKVSFYSSMFLLCCISVDRYFAISKATSAYRYRSQTMFLSKVSSAVVWVAALIFSMPEMRYTSVNNNTCTPYTGSKDQLRVIIQVGQIVLAFALPLVIMSICYSSIIKTLCQAQNFERNKAIKVILAVVAVFLVSQVPYNLVLFWSTLVTAKGGTTSCSYDNNLLYATDVTQCLAFFRCCLNPIVYAFIGVKFRNDLLKLLKDWGCMSHESFFKYTSRRRRSSGFTETETTTTLTISSPTMMPATEGMTHMIPNHVLRVGQTIRLDPATEASDRHDDSDRDHDISLEQLNRIILELDPTFEPLHLGRSPTQSTRPAAGNCCPDEDLHSMLIPRGCSTPSVMPSTSPSIPIPTRSGPSCSPPGTLVFSSSPGCSLPPLPFGSAPRRNLSPKSEAALFQGSLRLSQSNRNSVCSLLSMSTCSDTSYILGSNLSLASEEADSPDSILVRTCGSFSDGSRTRRCVPAHFTSTQPTMKFVMDTSKFWFRPHITRAEAEALVKDKEAGTFVVRDSTSYRGSFGLAMKVEPGSPEYFSFLTLTEPNSSELVRHFLIESSAKGVRVKGSSQEPYFGSLSALVYQHTISAYALPCKLVLHSKDASAAEWKANDKPASEDKSKLGGDFVSFFLEACNFVYLNAVPTEMLTGPCAVQKAVSFTLEAPASFRATTVNLKVSSKGVTLTDINRKLFFRRHYPAHLLSHSGEDPDNRLWVKGSCVGARMFGFVAKGVEAGVENVCHVFAEYDPLQPCDKVIEAVQ

>tniCCR9a ENSTNIT00000005792 Chr Un_random

TEGDYNYPDLMCDREPVRLFRSNYEPPLFWIIFLVGGAGNTAVVWTYLHLRRQLKTMTEVYLLNLAVADLLFLVTLPLWAAEALSGWTFGPALCKVIAAVYKVNLFSSMLLLTCISVDRYVVIVMATKARSSQQERRRRSLLVCLGVWLLALLLAIPELAFATTKGVGSLQYCRMVFPDGQGNRTKILALSLQVSMGFCLPFAIMASCYAVIIARLLRTRNFQKHKAMRVVLAVVLAFVLSQLPYNGVLVAEALQASDMPVTDCEELKRFDRVKQVLMSLAYVHACLNPFLYAFVGVRFRRDMKKLLCVRCQRAANKMQPSKSCRSPPSSTRATVMSDSDTSQALSL

>tniCCR9b ENSTNIT00000016883 Chr 6

FPLISNLQEPSDYEDDFDTGPTEDGGMCNTYDTVMSFRSQYEPPLFWIIFILGAVGNLLVVWIYSTVRNRLKTMTDVYLLNLAVADLLFLCTLPFLAVEAIKYWNFGLALCKTVLAVYRINFFSGMLLLTCISVDRYISIVQVTKAQNTKKQRLFWSKLICLIVWVVSTLLALPEFIYARVKTKQRDQSLCTLIYWDNSENQIKILVLSIQICMGFWLPLMVMIFCYSVVIRTLLQARNFQKHKALRVIFAVVLVFVLSQLPYNSLLIFETTQAANTTMSSCETRINLELAKQITKSLAYAHACLNPFLYAFIGVRFRQDLLGIARTCAGLGLIKLPAIPKRSSVASDMDTIPALSI

>tniCCR10 ENSTNIT00000015017 Chr 2

YCSLCFRIKIKNSKSRHVPFLCVFCVVFVLGVLGNGLVIATFASRCEGLRSMTDVFLLHLALADLLLLLTLPLQAVDTQLGWILPVSLCKATRACYAVNTYSGLLLLACISVDRYLMVARAQLRQWLRRWTFKAATLVALAVWASALTLSLPEILFSGVSGSGSKAYCGVLNSPEAKMATRVAIIAVFCLSFLVMLSSYSLIAMVLWGGQAQRRGKAWQRQRTLKLMLALVLLFLAFQLPYTLVLLRKLAGEFCGLLLEYVTCTLVSTRCCLNPILYALVGVRFRNDVLRLMHRAGCGCGGQARPQLSRSASPSSPAPTMLSFGT

>tniCCR11a ENSTNIT00000001852 edited Chr 8

VMIFMTLDRYMVIMHAITVARYRTLRAGIFVTMVLWLLSFSVSLPTFIFTEVTNESYGSSCYYAPEKDSWQIYDLFVINVLGLMLPLVVMIVCYSRIIPRLVNMRSTKRHRVIKLIISIMLTFFLFWAPYNFYFFLKFLHRKGKLVGDPCQIEEHLGLTGILTETFAYTHCCLNPIIYAFMGEKFMKRVLIFLRHCFPSLHFVSFLSRDLSMTSRRRSSVMSRSSEVSSTFIS

>tniCCR12a ENSTNIT00000021538 Chr Un_random

NDTPNVTNESFEVIGHVKFCTNEAVNNFGAKIIPVFYYINFLLSYLGNGLVLFIICKYEKLDTVTNIFLLNLVISNILFASSLPFLAIYHQSEWIFGNVLCKTVSSAYFIGFYSSILFLTLMTFDRYLAVVHAIAAAKCRKRVYAIIATVAVWCISILASMKELVLRNVWESPSNGLVCKESGYMESTMKVWRLVSYYQQFFGFFLIPLFTLMYCYITITIRILSTRMREKCRTIKLIFIIIFTFFICWTPYNVVIFLQAIQDSRGDEEESCSETLDYALYISQNIAYTYCCISPVFYTFVGKKFQSHFRRLVHSRVSAHLCRMPSLSELLQEVRSRAEKKSQSSISFLF

>tniCCR12b ENSTNIT00000008715 edited Chr Un_random

MCKRHVVKSSVVCLIFYISRFEKLTTVTNILLLNLILSSMIFMSSLPFMAVYMQLSNWIFGTVICKIVGGMYYLGIYSSVLFLTLLTFDRHLAVVYSLSAVRMRNVSYARISCGVVWLVSILACAKQMILRTTFEHSFENRIFCDEYPLISPLYSQLRSAGFYLQLFLFLLFPLVVIIYCYVRIAITVISSKLASKFKTVRLILFIVVLFFMCWTPYNITLLLDADICEESQKLGYVLQITQNLVYLYFCICPILYTFVGRKFQNYFRQMLVKQFPALKNHVSVSQSGTNGSTRTTRNDL

>truXCR1ba blast search Scaffold 80

MSVEDTDFNNDLAILCDISDLESITGAIFILIFVFSVTGNLLVLAILLFGEKLKNITSLFILNLACSDLVFTLTLPFWAYYQLHHWVFGEYACKLLTAAYIVGVNSSVILLTALTVDRFVTVVLQWPNDPSRRKRFAVVSCTAAWLISAAASVNDAITVKVETQWNNLSSCEDTSPESHVNLGYYLHVSLLFFLPFTIIVFCYSFILKTVLQASKRRTYCTVVMILCIVAAFFICWGPYNILLIVKIFYKPQSCYAEDTLYVAYSVCRIIAYSHCCMNPLLYMIPKTSRKHIWSILCCRNSKKKERDNVAGQSTTTMHNAAFTVQNSTVFFQQGVTAQTGSLAAMPEDTNTLSAVNGYRRMHSPN

>truXCR1bc ENSTRUT00000031511 Scaffold 80

MATNITIGLLKNLSFDNTSDYDMNYEDERCDTSGPRQVGTYVTVVVFSIVILLSLFGNILVMVILVKYENIKSLSNVLIMNLAVSDLFFTIGLPLWIHSHMNEWTLGEPACKMVMFVFFVGYYSSSILLVLMTAHRYIAVMRPLSSIMSNKGFCSALASPVIWVVSLIFAVPALIFTSVLQNNRCVTVRSIWNLFGIYQQNFFFFLNSVVFLFCYPQIICRLLRPTAQRRKSKTLKLIFILMVVFLVTWAPYNIVIFLKSFQFYPNSHDASTLQEKCNFTKRLEYAFYISRLFAFSQCCLNPVFYVFVGVKFKKHLKKMLKSWGRKSSSNSLQGRHSRLTVTSVTSGEESTNLGF

>tniCXCR7a ENSTNIT00000017165 Chr 2

MSLSTSDLEDLFKLFGDPNVSDTLSNISSVETMVCATAFNRSALLYSMCVLYTFIFIIGLAANALVLWVNVRAQRDSTPRHETHMYIAHLAVADLCVCATLPVWVSSLAQHGHWPFGQVACKLTHLLFSVNLFGSIFFLACMSVDRYLSVTRRRNNEEGTRRKLIRRGVCVGVWLLALVASLPDTYFLQTVKATHGDTMLCRPVYPEENPREWMVGVQLSFILLGFVLPFPIIAVFYILLARAFTGCSSSSSSTVEQERRVSRRVILAYIVVFLGCWGPYHGVLLVDSLSQLGLVPLTCTLENVIYVALHLTQCLSLLHCCFNPILYNFINRNYRYDLMKAFIFKYSTRTGLARLIEASNISEAEYSAVALDNP

>tniCCRL1a ENSTNIT00000004790 Chr 15

TFSYEDYPTLCEKEEVRSFAALFLPVMYAVCLVVGLAGNTVVVAVYAYLKRLRTMTDVFMTHLAVADLLLLLTLPFWAADAAQGWELGTATCKMVSSMYTVNFTCCMMLLACISVDRRLALAAARGEGRGRLLQRVFTKKHCWKVCFAVWALAFGLGLPDLVLSEVRWLSNRSVCLVVYPPSMVGGGKAGLEMMEVLLGFLLPLLVMIFSYWRVGQALKGLPVESRSRKWRALRVLLTVVAVFVVTQLPYNVVKVYRAVDSVYTLVTHCASSKALDRAAQVTESLALCHCCLNPVLYAFVGTSFKQHMVKLAKQFGQKRRKRRPNPAEEGGMEMSFNSHDASQETNTFSV

>tniCCRL1b ENSTNIT00000000588 Chr 6

MDYFHDDEDSSFNDSYDYNFEHSVCDKEAVRSFAGVFLPVIYALTLVVGLAGNVVVVAVYASRVRLRTLTDVCILNLAVSDLLLLFTLPFWAADAVHGWRLGSTACKLTSFLYSTNFSCGMLLLACISVDRCCAVVRSFSGKTSASPRVRRRWLLVCLVLWAVASFLGLPELIFSTVKHSHHRMACTEIYPPSMARAAKAALMLLEVLLRFLVPFLVMVVCYSWMGRVLSRAAGVQRERKWRALRVLLAVVAVFLLTQLPYNVVKLCRAMDTIYVVVTDCEVSKGLDRALQVTEGLALAHACINPLLYAFMGSSFRGHVLRVAKHVGQRLGGLRRHAPGEPAVEIALNSPSQPQSQSGSEEQDTSTFTI

>cmiCXCR1a AAVX01477245

PCARSVNTESVNTAMAVIYSLVCLLAMAGNVLVMIVILHNRRTMSSTDIYLLHLAIADVLFAITLPFSAADVINGWLFGDAMCKIVSVLKEVNFYSGILLLACISIDRFLAIVYSARANKQRSQFLTNVVCGGVWLFAIILSFPILVKGVFRSPDSERILCYEVLDGKSSATWRVATRFLRHIAGFLIPLSIMLFCYSVTINRVLKTKGFQKQKAMKVIIAVVLAFLICWLPYNITVFIDTLIRSKIINETCEMRNPLDKALFATESLGFLHSCINPILYAFIGVKF

>cmiCXCR1b AAVX01375965

YWSGTHVMIVILHNRRTMSSTDIYLLHLAIADVLFAITLPFSAADVINGWLFGDAMCKIVSVLKEVNFYSGILLLACISIDRFLAIVYSARTNKLKNQFLTNVVCGGVWLFAILLSLPILVKGVFRPPDSERILCYEVLDGKSSAKWRVATRFLRHIAGFLIPLSIMLFCYSVTINRVLKTKGFQKQKAMKVIIAVVLAFLICWLPYNITVFIDTLIRSKIINETCEMRNPLDKALFATESLGFLHSCINPILYAFIGVKFTRNLIKILVTKGIIKQGPIVKYGRSVSTTSESGLTSTTI

>cmiCXCR4 AAVX01302553

MCEEQNLYIKKILIPLFYSLIFILGIVGNGLVVLVMGYHKRYRSMTDKYRLHLSVADLLFVLTLPFWAIDTVNWYFGDITCKIVHVIYTMNLYSTVLIMAFISLDRYYAVVHATNSTRQRKMLANRFVYVGVWLPSILLTVPDMVFAKTTQLMDRVVCDRIYPTETFQTWLVAFRMNGVLVGFVLPALVILTCYCIIISKLSQSTGLQKRKALKS

>cmiCXCR5 AAVX01026304

LVSNKSVFLQNISIFLFLFFLPQEFPGNENDTSYYYGDGYVCNPRDEFSLESQTIAISVVSLLVSVMGVLGNGLVLTVLICTKHTRTPTDSYLLHLTLIDLLLSLSLPFTAIQGIFQWYFGQVVCKMVGTMYKLNFFCSSLLLGFISFDRYLAIVYAVQTYKKRKQVVIHCICAGVWALCLLLQLPNTIYLRVETQENKSMCTYPWASVERWLLTEQILYHVLGFALPLLVMCYCYTMVGKTLWRCQNFKRRKAVRVVLLVTAVFFLCWTPFNLVIFINTLSKLELINSQSCHFEHDLGVALRVTECIGSVRCCLNPILYAFIGVKFRNDVLKLLREMGCISQATLDKHFQLKQTTKNRSSTLTENQFVE

>cmiCXCR6 AAVX01097432

MVTIQPIYEYYDFNSTDDLNQQWELPCNKKEVKQFARFFIPLFYSIMCVTGLLGNSLVIVIYVFYEKLKTVTNIYMVNLAVADLLFLCTLPFWAVNACHGWIFDTFMCKVMNGAYTVNFYSCMLILTCVSINRYNVIVQATKMLNCKYRRCHSVVCTAVWLLAIILSLPQFIFSEARTDSSSKICSMVYPTNLSVAIKVQVNIIQMTVGFLIPFAAMVICYSIIAKTLLHGKGFQKHKSLRIIFVIVIVFVFCQLPFNIVKLMETLSIINNVSIKCTDSIKVDYAIIVTKCIAYVHCCLNPILYVFYVE

>cmiCCR4 AAVX01061874

MYTTKDSTSTADYDYYHGFLPEDIRSDNTFQAVLYSLVFVFGLPGNILVLWVLIQYKGLKSMTDIYLLNLTISDLLFVVSIPFWLHYMLHEWVFGNVLCKVINAGYLIGFYSGILFIMLMSIERYLAIVHHVFAFKVRKVRYGIISSAIIWFVAICASLPELIFYNIKTVSGKTECSIFYPNTTISTWKVFGFFQVNIFGFLIPFSVMTFCYSRIIITLLGNKASKKHRAVKIIFTVMIVFFVFWIPYNIVLFLNSLLEMEILNKIGNEGRLQMALQITQSLAFTHCCVNPFIYAFMGEKFRMYVGRFFRNWFSPILVCMGWSNVHFNFQNNNSSVQSHSSGRHDLSTVM

>cmiCCR6 AAVX01068499

MDYSKENGNSDEDLDDEYFYNTSDYSDYYNNSGTWSTLCKMQDIREFMSVFIPILYIIIFVMGIIGNGLVVITYVFYKKMRSMTDLYLLNLAVADIMFIATLPFWAVYESNQWIFGTFVCKFLKGVYSINFYSGVLLLAVISVDRYIAIVHATKSFNYRGKALLYGKIICAVTWLLVITASLPDFIYCEVYDFTTSGQKLCEIRYPKEWSKIGKLIAPSIQLSLGFFGPLLVMFFCYSMIIHTLLRVRSLQRHKAVRVVMAVVAAFVVCQVPYNITTLIETSVKNLHCEALKRLSLAKTVTKCLAFFHCCLNPILYVFIGVKFRFYFSKIIRDVWCLSKKYITFRYIQSRRTSEVFTSRRTSEFEIDTPSSFTM

>cmiCCR7 AAVX01024218

MTELLGFEFTTEDYNDVGTFDPNVDYRDFVSQCEMQEVRSFRESFLPVMYAVICVLGVLGNGLVVLTFIYFKRLKTMTDIYLLNLALADLLFLFTLPFWAVSVVKQWIFGQPMCRTVCVLYKLSFFSGMLLLMCISIDRYFSIVRAASAHRHRSRAVYYSKIISGGVWILAFILSMPELLYSEEIQRGGILVCKLRSNNNTMMFTTSAQLAMGFFIPFLVMIFCYSVIVKTLLISRSFEKNKAIKVIFSVVVVFILFQLPYNSVMLLDTISSIKANITNCDVNKRLNMALDVTKSLAYVRCCLNPFLYAFIGVKFRKDLLRLLKGMGCLSSQRLLAYINVKSSLSSKRGSIATDTDSTTTYSP

>cmiCCR9 AAVX01140752

DVYSPYYFPTMANYDDSISSSVDYDYSSGLCDRSEVRQFSQYFQPAFYWCVCLLGAIGNILVVVLYAFYKRIKTMTDVYLLNLSIADLLFLCTLPFWALNASIGWRFGSSLCKIVSGVYKINLFSCMFLLTCISFDRYIAIVLATKAHYSKNKRLLQSKLVCLFVWIIATILSFPEFAFSSTNESQMTCSSNYPSKNMKVVAFALQVTFGFLLPLVIMMICYSLITWTLLQTKRFQKHKAIKVIVAVVSTFVLTQLPYNSFLIVKLLDADNITITDCNTLKNVDIATQITQSIAFLHCCLNPFLYAFIGVKFRQHLLKFLKDIGCFNQRQFLKLFKPQPGTSKRSSVVSESETFGTFVL

>cmiCCR10 AAVX01326265

FFQFSLMMATYEVSTDYPEETNNVDYSRFPAPCENVYVIDLIRVLQPCVYSVVFILGLVGNGLVLTIYICYRKLKSMTDMYLLNLAIADLLFIFTLPYMAASSVHGWIFGNAMCKIVQSLYSMTFYSGFLFLTCISVDRYIVIVRATLAHRLRGKTIYYSKVTCLIVWFVSILISLPQFIYSHVELEEQVCWMTYPDVANGWMKVGTLITQLVIGFLIPLLVMLFCYSIIIRTLLQARNSEKHKAFKVIMAIVVTFVVFQTPYNIISILETADTLNHSSSVCQQRSQRDITIQVTSCLAYSRCCLNPLLYAFVGVRFRSDILHLLQASGCISRTRCLKHFRSRRYTKRGSASNIETSSSFAL

>cmiXCR1 AAVX01263959

MTMMSTEMADYSDEYNTSYDYPEDSGICNKDSINNFAKTFMPAFYVLLLLFSLLGNGLVLCVLVKYEYLRSITNIFILNLAVSDLLSAFCLPFWTMHHLTGWIFGDIMCKVVCAMFYTGFYSGIMFLTLMTFDRYLAVVHAVSALRSRKVRYAAITSVIVWGSSILATLPHAIFSTLTEESTCDYIYPKETALPWKLLRYFLQNVLFFVIPFAIIVYCYLRIIQTLIKCRTMQKYRTVKLIFIIVVVFFLCWAPYNVVIFLTALKDLRIFKTCEVINQIEYADFFTLNIAYFHCCLNPLFYAFIGAKFRGHLINLTHKCFPRVGNYKQQTPVIKAHNMDYSDGSTGNDITNNW

>cmiXCR1ba AAVX01174039

MTTALPTEDYYNYDNYNYNYSDEDFVVLCENYNSNMFGATLTVTLYSLIFILSVVGNSLVLWIMLRYEKLKTITDIFIVNLAISDLLFAASLPFWAKDHVSGWVFGNAMCKLLSGVFFVGYYSGIMFLTLMTADRYFAVVHAVYAARSRKTCYAVTASLVVWAISLSASVPEFIYSTEITWNQTEFCMANYPEDSEHIWLLFGYYQQIILFFLIPSVVIVYCYYKIMNIVLRCKARKKYKAVKVILCIVVIFFLCWAPYNMMIFLVSLKQLNVSPFTTCEMSKHIDYAYFISRNLAYFHCCLNPVFYAFVGTNFRIHLNKLLRNLFPCMRTQNLWIYKVRSLNRSSSHEYSEAFVMNTSIL

>cmiXCR1bb AAVX01286375

MTTALPTEDYYNYDNYNYNYSDEDFVVLCENYNSNMFGATLTVTLYSLIFILSVVGNSLVLWIMLRYEKLKTITDIFIVNLAISDLLFAASLPFWAKDHVSGWVFGNAMCKLLSGVFFVGYYSGIMFLTLMTADRYFAVVHAVYAARSRKTCYAVTASLVVWAISLSASVPEFIYSTEITWNQTEFCMANYPEDSEHIWLLFGYYQQIILFFLIPSVVIVYCYYKIMNIVLRCKARKKYKAVKVILCIVVIFFLCWAPYNMMIFLVSLKQLNVSPFTTCEMSKHINYAYFISRNLAYFHCCLNPMFYAFAGTNFRIHLNKLSRNLFPCMRTRNLWIYKVRNLNRSSSHEYSEAFVMNTSIL

>cmiCXCR7 AAVX01259911

SSECVSWEIFHCPYAFDKNAILYTMSFFYALIFIVGLVANVVVVYVNLKTKRTQYETHLYILNLAIADLCMVATLPIWLPSLVQHGSWPFGEFMCRLTHLVFSVNLFGSIFFLTCMSVDRYISVVRVGESVDRRKGVIRKIIIVYVWIFALVVSFPDILFLKTVTLPSNGETYCYPSYPEENFRKWMAGMEMVYIIIGFVIPFPIITVSYFLLARAISSTSDQENKNSRNIIFVYVIIFLICWLPYHTVLFIDVLWFMNLISFSCELESFMYISLHLTQCFSMVHCCANPILYSFINKNYRYRLMNAFTVKYTAKISITKLKETTETECSLVEAVSEMTAFE

>pmaCXCR4 AY178969.1 ENSPMAT00000007712 edited GL482293

MAELMHSISLDEADLLPMGLNDTSELEDNPPRPAATAPTCLAPSQSFHRVFLPVVYGLVCLLGFAGNGLILVILTCFTKKRTSSDLYLMHLAAADLLFVLTMPFWAVGSATEWVFGNVLCCLVNFTFTVNLASSILLLACISIERYLAIVRATKTDKVRRKFATKVTCGAVWALSLLLAMPDLVFSHVYIAPLSGHQLCEHVYPESASELWRTSLRALHHVLAFALPGIVIVFCYVMVIRTLSQLHNHEKRKALKVVVAIVAAFFVCWLPYNVVTLLDTLMRLDAVVNSDCEMEQRLGVAVAVTEGVGFSHCCFIPVLYAFVGKKFKENLARLRGCKACVGTPVASYREGKRQSSNRPHPISSDSDFSTSTIPA

>pmaCCR13a ENSPMAT00000011206 edited Scaffold GL480624

MDVSSGFTTANPLDSSSVWGPTDDYEDDSGALCSDSAQRLGQTLLPVLYSLVFILGLVGNGLAFLVLVRRQRRGNGSGTWDTTQVFLLNLAAADLLLVATLPLWARHAAREWPFGEWACKLATALYSVSFYCGVFVLTCLSVDRYLVVVRATRCRGVRWRRRRAWVACAGAWSAAMLLSLLDVLSSRVQLQADTPDSAPRTVCQSLHAGPGGHVRKAALAVFQTAACFLLPFAIMLACYIRIAVTLAHTKSRGSRRKAVRVIVALVLLFFLCWLPYNVVLLVVALADLRGTWLGCRTHEALLYARQTTETLAFTHCCLNPLLYAFVGVRFRADLYRMLGDVRSTLCSLLTGSGTARQAAEEASTGTQSMPTNSGDARSQGHPQLENAGVPIDTLQDLPYCHAHSPGLHVESSHIFSGVSTHSWVYF

>pmaCCR13b ENSPMAT00000011248 Scaffold GL476832

MLTETTEYMSTEDNGSDYASGRNGMSCDFDAEELSKTLLPIIYSIVFILAFIGNALVILILVKFKRHKEVTNFYLLNLSIADLIFAATMPFWAHESANGTWIFGNIMCKLVTACYSVNFYSGIFLLVCMSIDRFNAVVLATKFNKIRRLQNVKYICAFVWGFASSLSIFDIVYVKAQTFDDGITITCGHNIESKILDWKLGLAIVQNIFGFLIPFIIMLSCYSSITITLMHTKNYKKLKAFYVILAVVLAFFFCWLPFNVIMFLEAYLGYNDAMTCKAQTSLMYAKQAAEGIAFSHCCLNPFLYAFIGSKFKNDFMRLLSEVRLISQKQRRRTRQPSFTRSSQASDAESNTSVTNIKYGA

>pmaCXCR7a ENSPMAT00000011233 Scaffold GL476634

MSSIDFTSLLESLEEFNSSDLPEEWTRLCNASDLECLWREVFHCVHPFDKSSLFVPIAVLNCFVFFAGLLGNAAVLWLLWRPGRGGRPEVRCYVLNLAVADLFVVLTLPVWTVSLLGHGAWSLGDFMCKFTHFVYSVNLYGSIFFLVALSADRYVTLVLSPDLLGRLAERRARSRALACAGVWLLALAVSMPDIVYIESGTAPHTNQTYCVAAYPLGAFSQWMAGMQLMCNAVGFALPFPIIAVCYALVARALRDSARAGSPREARGARRLLVAYVAVFVLCWLPYHAALFVDALALLEVVELSCGAERFLYSALHVTQCFALVHCCANPALYSLLSRGARGALRRALVVRFSKGERLQEEADAATGDTDCTEIRPSERAIGD

>pmaCXCR7b ENSPMAT00000011447 Scaffold GL478568

MLAAKRLPRVNVPCVYSLCRLRTHRDNSNMDLTLDFDSEAFLNVTDYWDLEPTCNESTDGNCSSFYEHFDCPHVLNKGALLIGISIVYLVIFVVGVAGNVAVFCFNVFFHKNRFETHLYVVNLAISNLCIVLIMPVMVASFLHDDQWLYGNFLCKLTNVTFSVNLFAGVFFVTAMSVDRYITFVHFHEASSRRKERARLLICAIAWLLAVVASLPEIIYIRAVTTPSGETHCRADLPPDHFFRWMASINLLYNTLGFVVPFPMIVVSSVLLYRAIAWHGLSERNNGRAIVLVYATVFAACWTPYHALLLVDALALLRVLRLGCAMDEALYAGLHVAQCLAVGQCCVNPVLYCFVDKRRRQEFIRSVVVKFKASTASHDLLAEDCTVITQDTECAVILPNRS

**(b) Chemokine receptor domain sequences** used for the construction of phylogenetic trees. DARC sequences were removed from this list.

>hsaCXCR1

LVILYSRVGRSVTDVYLLNLALADLLFALTLPIWAASKVNGWIFGTFLCKVVSLLKEVNFYSGILLLACISVDRYLAIVHATRTLTQKRHLVKFVCLGCWGLSMNLSLPFFLFRQAYHPNNSSPVCYEVLGNDTAKWRMVLRILPHTFGFIVPLFVMLFCYGFTLRTLFKAHMGQKHRAMRVIFAVVLIFLLCWLPYNLVLLADTLMRTQVIQETCERRNNIGRALDATEILGFLHSCLNPIIY

>hsaCXCR2

LVILYSRVGRSVTDVYLLNLALADLLFALTLPIWAASKVNGWIFGTFLCKVVSLLKEVNFYSGILLLACISVDRYLAIVHATRTLTQKRYLVKFICLSIWGLSLLLALPVLLFRRTVYSSNVSPACYEDMGNNTANWRMLLRILPQSFGFIVPLLIMLFCYGFTLRTLFKAHMGQKHRAMRVIFAVVLIFLLCWLPYNLVLLADTLMRTQVIQETCERRNHIDRALDATEILGILHSCLNPLIY

>hsaCXCR3

AVLLSRRTALSSTDTFLLHLAVADTLLVLTLPLWAVDAAVQWVFGSGLCKVAGALFNINFYAGALLLACISFDRYLNIVHATQLYRRGPPARVTLTCLAVWGLCLLFALPDFIFLSAHHDERLNATHCQYNFPQVGRTALRVLQLVAGFLLPLLVMAYCYAHILAVLLVSRGQRRLRAMRLVVVVVVAFALCWTPYHLVVLVDILMDLGALARNCGRESRVDVAKSVTSGLGYMHCCLNPLLY

>hsaCXCR4

LVMGYQKKLRSMTDKYRLHLSVADLLFVITLPFWAVDAVANWYFGNFLCKAVHVIYTVNLYSSVLILAFISLDRYLAIVHATNSQRPRKLLAEKVVYVGVWIPALLLTIPDFIFANVSEADDRYICDRFYPNDLWVVVFQFQHIMVGLILPGIVILSCYCIIISKLSHSKGHQKRKALKTTVILILAFFACWLPYYIGISIDSFILLEIIKQGCEFENTVHKWISITEALAFFHCCLNPILY

>hsaCXCR5

VILERHRQTRSSTETFLFHLAVADLLLVFILPFAVAEGSVGWVLGTFLCKTVIALHKVNFYCSSLLLACIAVDRYLAIVHAVHAYRHRRLLSIHITCGTIWLVGFLLALPEILFAKVSQGHHNNSLPRCTFSQENQAETHAWFTSRFLYHVAGFLLPMLVMGWCYVGVVHRLRQAQRRPQRQKAVRVAILVTSIFFLCWSPYHIVIFLDTLARLKAVDNTCKLNGSLPVAITMCEFLGLAHCCLNPMLY

>hsaCXCR6

VISIFYHKLQSLTDVFLVNLPLADLVFVCTLPFWAYAGIHEWVFGQVMCKSLLGIYTINFYTSMLILTCITVDRFIVVVKATKAYNQQAKRMTWGKVTSLLIWVISLLVSLPQIIYGNVFNLDKLICGYHDEAISTVVLATQMTLGFFLPLLTMIVCYSVIIKTLLHAGGFQKHRSLKIIFLVMAVFLLTQMPFNLMKFIRSTHWEYYAMTSFHYTIMVTEAIAYLRACLNPVLY

>hsaCCR1

LVLVQYKRLKNMTSIYLLNLAISDLLFLFTLPFWIDYKLKDDWVFGDAMCKILSGFYYTGLYSEIFFIILLTIDRYLAIVHAVFALRARTVTFGVITSIIIWALAILASMPGLYFSKTQWEFTHHTCSLHFPHESLREWKLFQALKLNLFGLVLPLLVMIICYTGIIKILLRRPNEKKSKAVRLIFVIMIIFFLFWTPYNLTILISVFQDFLFTHECEQSRHLDLAVQVTEVIAYTHCCVNPVIY

>hsaCCR2

LILINCKKLKCLTDIYLLNLAISDLLFLITLPLWAHSAANEWVFGNAMCKLFTGLYHIGYFGGIFFIILLTIDRYLAIVHAVFALKARTVTFGVVTSVITWLVAVFASVPGIIFTKCQKEDSVYVCGPYFPRGWNNFHTIMRNILGLVLPLLIMVICYSGILKTLLRCRNEKKRHRAVRVIFTIMIVYFLFWTPYNIVILLNTFQEFFGLSNCESTSQLDQATQVTETLGMTHCCINPIIY

>hsaCCR3

MILIKYRRLRIMTNIYLLNLAISDLLFLVTLPFWIHYVRGHNWVFGHGMCKLLSGFYHTGLYSEIFFIILLTIDRYLAIVHAVFALRARTVTFGVITSIVTWGLAVLAALPEFIFYETEELFEETLCSALYPEDTVYSWRHFHTLRMTIFCLVLPLLVMAICYTGIIKTLLRCPSKKKYKAIRLIFVIMAVFFIFWTPYNVAILLSSYQSILFGNDCERSKHLDLVMLVTEVIAYSHCCMNPVIY

>hsaCCR4

LVLFKYKRLRSMTDVYLLNLAISDLLFVFSLPFWGYYAADQWVFGLGLCKMISWMYLVGFYSGIFFVMLMSIDRYLAIVHAVFSLRARTLTYGVITSLATWSVAVFASLPGFLFSTCYTERNHTYCKTKYSLNSTTWKVLSSLEINILGLVIPLGIMLFCYSMIIRTLQHCKNEKKNKAVKMIFAVVVLFLGFWTPYNIVLFLETLVELEVLQDCTFERYLDYAIQATETLAFVHCCLNPIIY

>hsaCCR5

LILINCKRLKSMTDIYLLNLAISDLFFLLTVPFWAHYAAAQWDFGNTMCQLLTGLYFIGFFSGIFFIILLTIDRYLAVVHAVFALKARTVTFGVVTSVITWVVAVFASLPGIIFTRSQKEGLHYTCSSHFPYSQYQFWKNFQTLKIVILGLVLPLLVMVICYSGILKTLLRCRNEKKRHRAVRLIFTIMIVYFLFWAPYNIVLLLNTFQEFFGLNNCSSSNRLDQAMQVTETLGMTHCCINPIIY

>hsaCCR6

ITFAFYKKARSMTDVYLLNMAIADILFVLTLPFWAVSHATGAWVFSNATCKLLKGIYAINFNCGMLLLTCISMDRYIAIVQATKSFRLRSRTLPRSKIICLVVWGLSVIISSSTFVFNQKYNTQGSDVCEPKYQTVSEPIRWKLLMLGLELLFGFFIPLMFMIFCYTFIVKTLVQAQNSKRHKAIRVIIAVVLVFLACQIPHNMVLLVTAANLGKMNRSCQSEKLIGYTKTVTEVLAFLHCCLNPVLY

>hsaCCR7

LTYIYFKRLKTMTDTYLLNLAVADILFLLTLPFWAYSAAKSWVFGVHFCKLIFAIYKMSFFSGMLLLLCISIDRYVAIVQAVSAHRHRARVLLISKLSCVGIWILATVLSIPELLYSDLQRSSSEQAMRCSLITEHVEAFITIQVAQMVIGFLVPLLAMSFCYLVIIRTLLQARNFERNKAIKVIIAVVVVFIVFQLPYNGVVLAQTVANFNITSSTCELSKQLNIAYDVTYSLACVRCCVNPFLY

>hsaCCR8

LVLVVCKKLRSITDVYLLNLALSDLLFVFSFPFQTYYLLDQWVFGTVMCKVVSGFYYIGFYSSMFFITLMSVDRYLAVVHAVYALKVRTIRMGTTLCLAVWLTAIMATIPLLVFYQVASEDGVLQCYSFYNQQTLKWKIFTNFKMNILGLLIPFTIFMFCYIKILHQLKRCQNHNKTKAIRLVLIVVIASLLFWVPFNVVLFLTSLHSMHILDGCSISQQLTYATHVTEIISFTHCCVNPVIY

>hsaCCR9

LVYWYCTRVKTMTDMFLLNLAIADLLFLVTLPFWAIAAADQWKFQTFMCKVVNSMYKMNFYSCVLLIMCISVDRYIAIAQAMRAHTWREKRLLYSKMVCFTIWVLAAALCIPEILYSQIKEESGIAICTMVYPSDESTKLKSAVLTLKVILGFFLPFVVMACCYTIIIHTLIQAKKSSKHKALKVTITVLTVFVLSQFPYNCILLVQTIDAYAMFISNCAVSTNIDICFQVTQTIAFFHSCLNPVLY

>hsaCCR10

THLAARRAARSPTSAHLLQLALADLLLALTLPFAAAGALQGWSLGSATCRTISGLYSASFHAGFLFLACISADRYVAIARALPAGPRPSTPGRAHLVSVIVWLLSLLLALPALLFSQDGQREGQRRCRLIFPEGLTQTVKGASAVAQVALGFALPLGVMVACYALLGRTLLAARGPERRRALRVVVALVAAFVVLQLPYSLALLLDTADLLAARERSCPASKRKDVALLVTSGLALARCGLNPVLY

>hsaCX3CR1

FALTNSKKPKSVTDIYLLNLALSDLLFVATLPFWTHYLINEKGLHNAMCKFTTAFFFIGFFGSIFFITVISIDRYLAIVLAANSMNNRTVQHGVTISLGVWAAAILVAAPQFMFTKQKENECLGDYPEVLQEIWPVLRNVETNFLGFLLPLLIMSYCYFRIIQTLFSCKNHKKAKAIKLILLVVIVFFLFWTPYNVMIFLETLKLYDFFPSCDMRKDLRLALSVTETVAFSHCCLNPLIY

>hsaXCR1

WVLVKYESLESLTNIFILNLCLSDLVFACLLPVWISPYHWGWVLGDFLCKLLNMIFSISLYSSIFFLTIMTIHRYLSVVSPLSTLRVPTLRCRVLVTMAVWVASILSSILDTIFHKVLSSGCDYSELTWYLTSVYQHNLFFLLSLGIILFCYVEILRTLFRSRSKRRHRTVKLIFAIVVAYFLSWGPYNFTLFLQTLFRTQIIRSCEAKQQLEYALLICRNLAFSHCCFNPVLY

>hsaCXCR7

WVNIQAKTTGYDTHCYILNLAIADLWVVLTIPVWVVSLVQHNQWPMGELTCKVTHLIFSINLFGSIFFLTCMSVDRYLSITYFTNTPSSRKKMVRRVVCILVWLLAFCVSLPDTYYLKTVTSASNNETYCRSFYPEHSIKEWLIGMELVSVVLGFAVPFSIIAVFYFLLARAISASSDQEKHSSRKIIFSYVVVFLVCWLPYHVAVLLDIFSILHYIPFTCRLEHALFTALHVTQCLSLVHCCVNPVLY

>hsaCCBP2

MVLLRYVPRRRMVEIYLLNLAISNLLFLVTLPFWGISVAWHWVFGSFLCKMVSTLYTINFYSGIFFISCMSLDKYLEIVHAQPYHRLRTRAKSLLLATIVWAVSLAVSIPDMVFVQTHENPKGVWNCHADFGGHGTIWKLFLRFQQNLLGFLLPLLAMIFFYSRIGCVLVRLRPAGQGRALKIAAALVVAFFVLWFPYNLTLFLHTLLDLQVFGNCEVSQHLDYALQVTESIAFLHCCFSPILY

>hsaCCRL1

AIYAYYKKQRTKTDVYILNLAVADLLLLFTLPFWAVNAVHGWVLGKIMCKITSALYTLNFVSGMQFLACISIDRYVAVTKVPSQSGVGKPCWIICFCVWMAAILLSIPQLVFYTVNDNARCIPIFPRYLGTSMKALIQMLEICIGFVVPFLIMGVCYFITARTLMKMPNIKISRPLKVLLTVVIVFIVTQLPYNIVKFCRAIDIIYSLITSCNMSKRMDIAIQVTESIALFHSCLNPILY

>hsaCCRL2

LILVKYKGLKRVENIYLLNLAVSNLCFLLTLPFWAHAGGDPMCKILIGLYFVGLYSETFFNCLLTVQRYLVFLHKGNFFSARRRVPCGIITSVLAWVTAILATLPEYVVYKPQMEDQKYKCAFSRTPFLPADETFWKHFLTLKMNISVLVLPLFIFTFLYVQMRKTLRFREQRYSLFKLVFAIMVVFLLMWAPYNIAFFLSTFKEHFSLSDCKSSYNLDKSVHITKLIATTHCCINPLLY

>musCXCR1

LVILYRRRTRSVMDVYVLNLAIADLLFSLTLPFLAVSKLKGWIFGTPLCKMVSLLKEFNFFSGILLLACISVDRYLAIVHATRTLARKRYLVKFVCVGIWGLSLILSLPFAIFRQAYKPFRSGTVCYEVLGEATTDFRMTLRGLSHIFGFLLPLLTMLVCYGLTLRMLFKTHMRQKHRAMGVIFAVVLVFLLCCLPYNLVLLSDTLLGAHLIEDTCERRNDIDQALYITEILGFSHSCLNPIIY

>musCXCR2

LVILYNRSTCSVTDVYLLNLAIADLFFALTLPVWAASKVNGWTFGSTLCKIFSYVKEVTFYSSVLLLACISMDRYLAIVHATSTLIQKRHLVKFVCIAMWLLSVILALPILILRNPVKVNLSTLVCYEDVGNNTSRLRVVLRILPQTFGFLVPLLIMLFCYGFTLRTLFKAHMGQKHRAMRVIFAVVLVFLLCWLPYNLVLFTDTLMRTKLIKETCERRDDIDKALNATEILGFLHSCLNPIIY

>musCXCR3

AVLLSQRTALSSTDTFLLHLAVADVLLVLTLPLWAVDAAVQWVFGPGLCKVAGALFNINFYAGAFLLACISFDRYLSIVHATQIYRRDPRVRVALTCIVVWGLCLLFALPDFIYLSANYDQRLNATHCQYNFPQVGRTALRVLQLVAGFLLPLLVMAYCYAHILAVLLVSRGQRRFRAMRLVVVVVAAFAVCWTPYHLVVLVDILMDVGVLARNCGRESHVDVAKSVTSGMGYMHCCLNPLLY

>musCXCR4

LVMGYQKKLRSMTDKYRLHLSVADLLFVITLPFWAVDAMADWYFGKFLCKAVHIIYTVNLYSSVLILAFISLDRYLAIVHATNSQRPRKLLAEKAVYVGVWIPALLLTIPDFIFADVSQGDISQGDDRYICDRLYPDSLWMVVFQFQHIMVGLVLPGIVILSCYCIIISKLSHSKGHQKRKALKTTVILILAFFACWLPYYVGISIDSFILLGVIKQGCDFESIVHKWISITEALAFFHCCLNPILY

>musCXCR5

VILERHRHTRSSTETFLFHLAVADLLLVFILPFAVAEGSVGWVLGTFLCKTVIALHKINFYCSSLLLACIAVDRYLAIVHAVHAYRRRRLLSIHITCTAIWLAGFLFALPELLFAKVGQPHNNDSLPQCTFSQENEAETRAWFTSRFLYHIGGFLLPMLVMGWCYVGVVHRLLQAQRRPQRQKAVRVAILVTSIFFLCWSPYHIVIFLDTLERLKAVNSSCELSGYLSVAITLCEFLGLAHCCLNPMLY

>musCXCR6

IIYIFYQKLRTLTDVFLLNLPLADLVFVCTLPFWAYAGTYEWVFGTVMCKTLRGMYTMNFYVSMLTLTCITVDRFIVVVQATKAFNRQAKWKIWGQVICLLIWVVSLLVSLPQIIYGHVQDIDKLICQYHSEEISTMVLVIQMTLGFFLPLLTMILCYSGIIKTLLHARNFQKHKSLKIIFLVVAVFLLTQTPFNLAMLIQSTSWEYYTITSFKYAIVVTEAIAYFRACLNPVLY

>musCCR1

LVLMQHRRLQSMTSIYLFNLAVSDLVFLFTLPFWIDYKLKDDWIFGDAMCKLLSGFYYLGLYSEIFFIILLTIDRYLAIVHAVFALRARTVTFGIITSIITWALAILASMPALYFFKAQWEFTHRTCSPHFPYKSLKQWKRFQALKLNLLGLILPLLVMIICYAGIIRILLRRPSEKKVKAVRLIFAITLLFFLLWTPYNLSVFVSAFQDVLFTNQCEQSKQLDLAMQVTEVIAYTHCCVNPIIY

>musCCR1L1

LVLIQHKRLRNMTSIYLFNLAISDLVFLSTLPFWVDYIMKGDWIFGNAMCKFVSGFYYLGLYSDMFFITLLTIDRYLAVVHVVFALRARTVTFGIISSIITWVLAALVSIPCLYVFKSQMEFTYHTCRAILPRKSLIRFLRFQALTMNILGLILPLLAMIICYTRIINVLHRRPNKKKAKVMRLIFVITLLFFLLLAPYYLAAFVSAFEDVLFTPSCLRSQQVDLSLMITEALAYTHCCVNPVIY

>musCCR2

IILIGCKKLKSMTDIYLLNLAISDLLFLLTLPFWAHYAANEWVFGNIMCKVFTGLYHIGYFGGIFFIILLTIDRYLAIVHAVFALKARTVTFGVITSVVTWVVAVFASLPGIIFTKSKQDDHHYTCGPYFTQLWKNFQTIMRNILSLILPLLVMVICYSGILHTLFRCRNEKKRHRAVRLIFAIMIVYFLFWTPYNIVLFLTTFQESLGMSNCVIDKHLDQAMQVTETLGMTHCCINPVIY

>musCCR3

LILIKYRKLQIMTNIYLFNLAISDLLFLFTVPFWIHYVLWNEWGFGHYMCKMLSGFYYLALYSEIFFIILLTIDRYLAIVHAVFALRARTVTFATITSIITWGLAGLAALPEFIFHESQDSFGEFSCSPRYPEGEEDSWKRFHALRMNIFGLALPLLIMVICYSGIIKTLLRCPNKKKHKAIRLIFVVMIVFFIFWTPYNLVLLFSAFHSTFLETSCQQSKHLDLAMQVTEVIAYTHCCINPVIY

>musCCR4

LVLFKYKRLKSMTDVYLLNLAISDLLFVLSLPFWGYYAADQWVFGLGLCKIVSWMYLVGFYSGIFFIMLMSIDRYLAIVHAVFSLKARTLTYGVITSLITWSVAVFASLPGLLFSTCYTEHNHTYCKTQYSVNSTTWKVLSSLEINVLGLLIPLGIMLFCYSMIIRTLQHCKNEKKNRAVRMIFAVVVLFLGFWTPYNVVLFLETLVELEVLQDCTLERYLDYAIQATETLAFIHCCLNPVIY

>musCCR5

LILISCKKLKSVTDIYLLNLAISDLLFLLTLPFWAHYAANEWVFGNIMCKVFTGLYHIGYFGGIFFIILLTIDRYLAIVHAVFALKVRTVNFGVITSVVTWAVAVFASLPEIIFTRSQKEGFHYTCSPHFPHTQYHFWKSFQTLKMVILSLILPLLVMVICYSGILHTLFRCRNEKKRHRAVRLIFAIMIVYFLFWTPYNIVLLLTTFQEFFGLNNCSSSNRLDQAMQATETLGMTHCCLNPVIY

>musCCR6

MTFAFYKKARSMTDVYLLNMAITDILFVLTLPFWAVTHATNTWVFSDALCKLMKGTYAVNFNCGMLLLACISMDRYIAIVQATKSFRVRSRTLTHSKVICVAVWFISIIISSPTFIFNKKYELQDRDVCEPRYRSVSEPITWKLLGMGLELFFGFFTPLLFMVFCYLFIIKTLVQAQNSKRHRAIRVVIAVVLVFLACQIPHNMVLLVTAVNTGKVGRSCSTEKVLAYTRNVAEVLAFLHCCLNPVLY

>musCCR7

LTYIYFKRLKTMTDTYLLNLAVADILFLLILPFWAYSEAKSWIFGVYLCKGIFGIYKLSFFSGMLLLLCISIDRYVAIVQAVSAHRHRARVLLISKLSCVGIWMLALFLSIPELLYSGLQKNSGEDTLRCSLVSAQVEALITIQVAQMVFGFLVPMLAMSFCYLIIIRTLLQARNFERNKAIKVIIAVVVVFIVFQLPYNGVVLAQTVANFNITNSSCETSKQLNIAYDVTYSLASVRCCVNPFLY

>musCCR8

LVLVGCKKLRSITDIYLLNLAASDLLFVLSIPFQTHNLLDQWVFGTAMCKVVSGLYYIGFFSSMFFITLMSVDRYLAIVHAVYAIKVRTASVGTALSLTVWLAAVTATIPLMVFYQVASEDGMLQCFQFYEEQSLRWKLFTHFEINALGLLLPFAILLFCYVRILQQLRGCLNHNRTRAIKLVLTVVIVSLLFWVPFNVALFLTSLHDLHILDGCATRQRLALAIHVTEVISFTHCCVNPVIY

>musCCR9

LVYWYCTRVKTMTDMFLLNLAIADLLFLATLPFWAIAAAGQWMFQTFMCKVVNSMYKMNFYSCVLLIMCISVDRYIAIVQAMKAQVWRQKRLLYSKMVCITIWVMAAVLCTPEILYSQVSGESGIATCTMVYPKDKNAKLKSAVLILKVTLGFFLPFMVMAFCYTIIIHTLVQAKKSSKHKALKVTITVLTVFIMSQFPYNSILVVQAVDAYAMFISNCTISTNIDICFQVTQTIAFFHSCLNPVLY

>musCCR10

THLAARRTTRSPTSVHLLQLALADLLLALTLPFAAAGALQGWNLGSTTCRAISGLYSASFHAGFLFLACISADRYVAIARALPAGQRPSTPSRAHLVSVFVWLLSLFLALPALLFSRDGPREGQRRCRLIFPESLTQTVKGASAVAQVVLGFALPLGVMAACYALLGRTLLAARGPERRRALRVVVALVVAFVVLQLPYSLALLLDTADLLAARERSCSSSKRKDLALLVTGGLTLVRCSLNPVLY

>musCX3CR1

LALTNSRKPKSITDIYLLNLALSDLLFVATLPFWTHYLISHEGLHNAMCKLTTAFFFIGFFGGIFFITVISIDRYLAIVLAANSMNNRTVQHGVTISLGVWAAAILVASPQFMFTKRKDNECLGDYPEVLQEMWPVLRNSEVNILGFALPLLIMSFCYFRIIQTLFSCKNRKKARAVRLILLVVFAFFLFWTPYNIMIFLETLKFYNFFPSCDMKRDLRLALSVTETVAFSHCCLNPFIY

>musXCR1

WVLVKYENLESLTNIFILNLCLSDLMFSCLLPVLISAQWSWFLGDFFCKFFNMIFGISLYSSIFFLTIMTIHRYLSVVSPISTLGIHTLRCRVLVTSCVWAASILFSIPDAVFHKVISLNCKYSEHHGFLASVYQHNIFFLLSMGIILFCYVQILRTLFRTRSRQRHRTVRLIFTVVVAYFLSWAPYNLTLFLKTGIIQQSCESLQQLDIAMIICRHLAFSHCCFNPVLY

>musCXCR7

WVNIQAKTTGYDTHCYILNLAIADLWVVITIPVWVVSLVQHNQWPMGELTCKITHLIFSINLFGSIFFLACMSVDRYLSITYFTGTSSYKKKMVRRVVCILVWLLAFFVSLPDTYYLKTVTSASNNETYCRSFYPEHSIKEWLIGMELVSVILGFAVPFTIIAIFYFLLARAMSASGDQEKHSSRKIIFSYVVVFLVCWLPYHFVVLLDIFSILHYIPFTCQLENVLFTALHVTQCLSLVHCCVNPVLY

>musCCBP2

VVLLHSAPRRRTMELYLLNLAVSNLLFVVTMPFWAISVAWHWVFGSFLCKVISTLYSINFYCGIFFITCMSLDKYLEIVHAQPLHRPKAQFRNLLLIVMVWITSLAISVPEMVFVQIHQTLDGVWHCYADFGGHATIWKLYLRFQLNLLGFLLPLLAMIFFYSRIGCVLVRLRPPGQGRALRMAAALVIVFFMLWFPYNLTLFLHSLLDLHVFGNCEISHRLDYTLQVTESLAFSHCCFTPVLY

>musCCRL1

AIYAYYKKQRTKTDVYILNLAVADLLLLITLPFWAVNAVHGWILGKMMCKVTSALYTVNFVSGMQFLACISIDRYWAITKAPSQSGAGRPCWIICCCVWMAAILLSIPQLVFYTVNQNARCTPIFPHHLGTSLKASIQMLEIGIGFVVPFLIMGVCYASTARALIKMPNIKKSRPLRVLLAVVVVFIVTQLPYNVVKFCQAIDAIYLLITSCDMSKRMDVAIQVTESIALFHSCLNPILY

>musCCRL2

FILVKYKGLKNLGNIYFLNLALSNLCFLLPLPFWAHTAAHGESPGNGTCKVLVGLHSSGLYSEVFSNILLLVQGYRVFSQGRLASIFTTVSCGIVACILAWAMATALSLPESVFYEPRMERQKHKCAFGKPHFLPIEAPLWKYVLTSKMIILVLAFPLLVFIICCRQLRRRQSFRERQYDLHKPALVITGVFLLMWAPYNTVLFLSAFQEHLSLQDEKSSYHLDASVQVTQLVATTHCCVNPLLY

>btaCXCR1

LVILYSRIGRSVTDVYLLNLAMADLLFAMTLPIWTASKAKGWVFGTPLCKVVSLLKEVNFYSGILLLACISMDRYLAIVHATRTLTQKWHWVKFICLGIWALSVILALPIFIFREAYQPPYSDLVCYEDLGANTTKWRMIMRVLPQTFGFLLPLLVMLFCYGFTLRTLFSAQMGHKHRAMRVIFAVVLVFLLCWLPYNLVLIADTLMRAHVIAETCQRRNDIGRALDATEILGFLHSCLNPLIY

>btaCXCR2

LVILYSRIGRSVTDVYLLNLAMADLLFAMTLPIWAASKAKGWVFGTPLCKVVSLLKEVNFYSGILLLACISMDRYLAIVHATRTLTQKRHWVKFICLGIWALSVILALPVFIFRRAIHPPYSSAVCYEDMGANTTKWRMVMRVLPQTFGFLLPLLVMLFCYGFTLRTLFSAQMGQKHRAMRVIFAVVLVFLLCWLPYNLVLIVDTLMRAHVIAETCQRRNDIGRALDATEILGFLHSCLNPLIY

>btaCXCR3

VVLLSQRAALSSTDTFLLHLAVADALLVLTLPLWAVDAAIQWVFGSGLCKVAGALFNINFYAGALLLACISFDRYLSIVHATQLYRRGPPTRVALTCVAVWGLCLLFALPDFIFLSSHHDNRLNATHCQYNFPQEGHTALRILQLVAGFLLPLLVMAYCYARILAVLLVSRGQRRLRAMRLVVVVVVAFALCWTPYHLVVLVDTLMDLGALARNCGRESSVDIAKSVTSGMGYMHCCLNPLLY

>btaCXCR4

LVMGYQKKLRSMTDKYRLHLSVADLLFVLTLPFWAVDAVANWYFGKFLCKAVHVIYTVNLYSSVLILAFISLDRYLAIVHATNSQKPRKLLAEKVVYVGVWLPAVLLTIPDLIFADIKEVDERYICDRFYPSDLWLVVFQFQHIVVGLLLPGIVILSCYCIIISKLSHSKGYQKRKALKTTVILILTFFACWLPYYIGISIDSFILLEIIQQGCEFESTVHKWISITEALAFFHCCLNPILY

>btaCXCR5

VILERHRQTRSSTETFLFHLAVADLLLVFILPFAVAESSVGWVLGTFLCKTVISLHKINFYCSSLLLACIAVDRYLAIVHAVHAYRHRRLLSIHITCATIWLAGFFFALPEILFAKVSEPHYNDSLPHCTFSQENQAETNAWFTSRFLYHIGGFLLPMLVMAWCYVGVVHRLCQAQRRPQRQKAVRVAILVTSVFFLCWSPYQVVIFLDTLARLKMLGSSCELDGYLSMAITMSEFLGLAHCCLNPMLY

>btaCXCR6

VIYVFYQKLKSLTDVFLMNLPLADLVFVCTLPFWAYAGIHEWVFGNVMCKALLGIYTLNFYTSMLVLTCITVDRFVAVVRATKAYNQQAKRMAWGKAICSSIWVVSLLVSLPQIIYGNVLYHDKPFCGYHEAISTMVLAIQMTLGFFLPLLAMILCYSVIIKTLLQARGFRKHKSLKIIFLVVAVFLLTQTPFNLVKLIRSTSWEYHTMTSFDYAITVTEAIAYLRACLNPVLY

>btaCCR1

LVLMQYKRLKSMTSIYLLNLAISDLIFLFTLPFWIDYKVKDDWIFGDAMCKLLSGFYFMGLYSEIFFIILLTIDRYLAIVHAVFALRARTITFGIITSIVVWVLAVLASVPGLYFSKTQWEFTHHTCSIHFPPESFTKWKQFQALKLNIMGLVLPLLVMIVCYTGIIKILLRRPNEKKAKAVRLIFVIMIIFFLFWTPYNLSVFVAAFQDSLFTRKCEQSRQLDLAIQVTEVIAYTHCCINPVIY

>btaCCR1L1

VVLVLYKRIKNIINICLLNLAISDLIFLFTLPFWIDYKVKDDWIFGDAMCKLLFGFFFLGLYSKIFFIILLTIDRYLTNVHPQFKRQCWNITSGTVTSIVVWVLAILASVPGLYFSKTQWWLTHYTCSLHFPPESHGRKWKQFLALKLNILGLILPLLVMTVCYGHIKIIKIMLRTWNKKANIIRLIFVIMITFFLFWTPCNLTVFVSAFQDSLCEHCSQLDLAIEVTEAIAYAHCCMNPIVY

>btaCCR2

LILINCKKLKSMTDIYLLNLAISDLLFLLTMPFWAHYAADQWVFGNVMCKFFTGLYHIGYFGGIFFIILLTIDRYLAIVHAVFALKARTVTFGVVTSGVTWVVAVFASLPGIIFIKSLEEHSGYACAPYFPLGWKNFHTIMRSILGLVLPLLVMIICYSGIIKTLLRCRNEKKKHKAVRLIFVIMIVYFLFWAPYNIVLLLSTFQEFFGLSNCKSSSQLDQAMQVTETLGLTHCCINPIIY

>btaCCR3

VILTKYKRLRIMTNIYLLNLAISDVLFLFTLPFWIHYVRWNEWVFGHRMCKLLSGLYYMGLYSEIFFIILLTIDRYLAIVHAVFALRARTVTFGIVTSIFTWVLAGLAALPEFFFHETQEEAGQTFCSPLYPEDNENAWKRFHALRMNILGLALPLLVMAICYSGIIKTLLRCPSKKKYKAIRLIFVIMVVFFIFWTPYNLVVLLFAFQMHLKADCEQSRQLDLAMLVTEVIAYTHCCVNPVIY

>btaCCR4

LVLFKYKRLKSMTDVYLLNLAISDLLFVLSLPFWGYYAADQWVFGLGLCKLISWIYLVGFYSGIFFITLMSIDRYLAIVHAIFSLRARTLTYGVITSVATWSVAVLVSLPGLLFSTCYTERNHTYCKTKYSFNSTRWKVLSSLEINILGLVIPLGIMLFCYSMIIRTLQHCKNEKKNKAVKMIFAVVVLFLGFWTPYNVVLFLETLVELEVLQDCTFERHLDYAIQTTETLAFVHCCLNPVIY

>btaCCR5

LILINCKKLKSMTDIYLLNLAISDLLFIITIPFWAHYAADQWVFGNTMCQLFTGFYFIGYFGGIFFIILLTIDRYLAIVHAVFALKARTVTFGAATSVVTWVVAVFASLPGIIFTKSQKEGSRHTCSPHFPSSQYHFWKNFQTLKIVILGLVLPLLVMIVCYSGIIKTLLRCRNEKKKHKAVRLIFVIMIVYFLFWAPYNIVLLLSTFQEFFGLNNCSGSNRLDQAMQVTETLGMTHCCINPIIY

>btaCCR6

VTFAFYKKAKSMTDVYLLNMAVADILFVLTLPFWAVNHATGEWIFSNAMCKLTRGIYAINFNCGMLLLTCISLDRYIAIVQATKSFRLRSRTLAHHKLICLAVWAVSILISSSTFTFNQKYKLQGGDVCEPRYHAVSEPIRWKLLMLGLQLLFGFFIPLVFMIFCYAFIVKTLVQAQNSKRHRAIRVIIAVVLVFLACQIPHNMVLLVTAVNLGRTGRSCGSEKLLGYAKNVTEVLAFLHCCLNPALY

>btaCCR7

LTYIYFKRLKTMTDTYLLNLALADILFLLTLPFWAYSAAKSWVFGVHVCKLIFGIYKISFFSGMLLLLCISIDRYVAIVQAVSAHRHRARVLLISKLSCLGIWMLAIVLSTPEVMYSGIQKSSSEQALRCSLVTEHVEALITIQVAQMVVGFLIPLMAMSFCYLVIIRTLLQARNFERNKAIKVIIAVVVVFVAFQLPYNGVVLAQTVANFNITSGTSCELSKQLNIAYDVTYSLACVRCCVNPFLY

>btaCCR8

LVLVACKKLRSVTDVYLLNLALSDLLFVFSFPFQTHYQLDQWVFGTVMCKVVSGFYYIGFFSSMFFITLMSMDRYLAVVHAVYALKVRTISMGTALSLVVWLTALVATSPLLVFYQVASENGILQCYSYYNQQTLKWKIFIHFEVNILGLLIPFSILMFCYIRILHQLKSCQNHNKTKAIKLVLIVVVASLLFWVPFNTVLFLTSLHDMHVLDGCVMSQQLTYATHVTETISFTHCCVNPIIY

>btaCCR9

LVYWYCTRVKTMTDMFLLNLAIADLLFLATLPFWAIAAADQWKFQTFMCKVVNSMYKMNFYSCVLLIMCISVDRYIAIAQAMRAQMWRQKRLLYSKMVCFTIWVTAAALCLPELLYSQVKEEHGIAICTMVYSSDDSTKLKSAVLTLKVILGFFLPFVVMACCYTIIIHTLIQAKKSSKHKALKVTITVLTVFVLSQFPHNCVLLVQTIDAYAMFISSCALSIKIDICFQVTQTVAFFHSCLNPVLY

>btaCCR10

THLAARRAARSPTSAHLLQLALADLLLALTLPFAAAGALQGWSLGSATCRAISGLYSASFHAGFLFLACISADRYVAIARALPAGPRPSAPGRAHLVSVIVWLLSLLLALPALLFSQDGHREGQRRCRLIFPEGLTQTVKGASAVAQVVLGFALPLGVMAACYALLGRTLLATRGPERRRALRVVVALVAAFVVLQLPYSLALLLDTADLLAARERSCPASKRKDLALLVTGGLALARCGLNPVLY

>btaCX3CR1

FALINSQRSKSITDIYLLNLALSDLLFVATLPFWTHYVINEQGLHHATCKLITAFFFIGFFGGIFFITVISVDRFLAIVLAANSMSNRTVQHGVTTSLGVWAAAILVATPQFMFTREKENECFGDYPEILQEIWPVILNTEINFLGFLLPLLIMSYCYFRIMQTLFSCKNHKKAKAIRLIFLVVVVFFLFWTPYNVMIFLQTLNLYDFFPKCDVKRDLKLAISVTE

TIAFSHCCLNPLIY

>btaXCR1

WVLVKYESLESLTNVFILNLCLSDLVFSCLLPVWILGYHWGWVLGDLLCKLLNMVFSISLYSSISFLTIMTIHRYLSVVSPISSLRVHTLQRRVLVTAAVWAASILSSIPDAIFHKVFPSGCDYSELEGFLASVYQHNVIFLLSVGVILFCYVEILRTLFRSRSKRRHRTVRLIFTIVAAYFLSWAPYNLILFLQTLLKLGVIQSCEVSQQLDYALLICRNVAFSHCCFNPVLY

>btaCXCR7

WVNIQAKTTGYDTHCYILNLAIADLWVVVTIPVWVVSLVQHNQWPMGELTCKVTHLIFSINLFGSIFFLTCMSVDRYLSVAYFASTSGRKKRLVRRAVCVLVWLLAFGVSLPDTYYLKTVTSASNNETYCRAFYPEHSVKEWLISMELVSVILGFAIPFCIIAVFYFLLARAIASSSDQEKQSSRKIILSYVVVFLVCWLPYHLVVLLDIFSILHYIPFTCQLEAFLFTALHVTQCLSLVHCCVNPVLY

>btaCCBP2

AVLLRFVPRRRMTETYLLNLAISNLLFVVTLPFWGISVAWHWVFGSVLCKVVSTLYTVNFYSGIFFISCMSLDKYLEIVCARPYHRLRTRAKSLLLAASVWAMALAVSIPDMVFVRTHENSPGVWECYADFGGHGTIWKLFLRFQQNLLGFLLPLLAMIFFYSRIGSVLVSLRPPGQRRALRMAVALVVAFFVLWFPYNLTLFLHSLLDLQVFGDCRVSQHLDYALQVTESIAFLHCCFTPVLY

>btaCCRL1

AIYAYYKKRRTKTDVYILNLAVADLFLLFTLPFWAVNAVHGWVLGKIMCKVTSALYTVNFVSGMQFLACISTDRYWAVTKAPSQSGVGKPCWVICFCVWVAAILLSIPQLVFYTVNHKARCVPIFPYHLGTSMKASIQILEICIGFIIPFLIMAVCYFITAKTLIKMPNIKKSQPLKVLFTVVIVFIVTQLPYNIVKFCQAIDIIYSLITDCDMSKRMDVAIQITESIALFHSCLNPVLY

>btaCCRL2

FILVKYKGLRQAENMSFLNLALSNLGFLLTLPFWAYAASHGEGFDDPLCKILLLLYSIGLYSEAFFNVLLTVQRYKEFFHVRRRFSACRTVAGSIFISVLVWVTATLVTLPELVSYKPQMQSQKYKCFFTGLHFLPADETFWKHFLTLKMNILGFLLPLFAFVYCYVRMRKTLQFRERNYGLFKLVFTIMAVFLLMWGPYNIVLFLSAFNEHFSLHGCGSSYNLNKSVQITRIIAATHCCVNPLLY

>lafCXCR1

LVILYNRVSSSVTDVYLLNLAMADLLFALTLPIWAASKEHGWIFGTPLCKVVSLLKEVNFYSGILLLACISVDRYLAIVHATRTLIQKRHLVKFICLGIWGLSLILSLPFFLFRQAYRLPYSSLIVCYEDIGNNTAKWRLVLRILPQTFGFVLPLLVMVFCYGFTLRTLLEARMKQKYRAMRVIFVVVFVFLLCWLPYNLVLVTDTLMRTGLIEETCERRNDIGRALDATEILGFFHSCLNPVIY

>lafCXCR2

LVILYNRVSCSVTDVYLLNLAMADLLFALSLPIWAASKKNGWIFGTTLCKVVSLLKEVNFYSGILLLACISVDRYLAIVHATRTLIQKRHLVKFICLGIWGLSLILALPIVLFRKAIYPPYSSPVCYEDIGNNTANWRLVLRILPQTFGFILPLLVMVFCYGFTLRTLFEAHTGQKHKAMRIIFAVVLVFLLCWLPYNLVLVTDTLMRIGVIKETCERRNDIGWALDVTEILGFLHSCLNPLIY

>lafCXCR3

AVLLSQRAALSSTDTFLFHLAVADALLVLTLPLWAVDAAVQWVFGSGLCKVAGALFNINFYAGALLLACISFDRYLSIVHATQLYRRRPPARVALTCVVVWGLCLLFALPELIFLSAHHDDRLNATHCQYNFPQVGRTALRMLQLVAGFLLPLLVMAYCYARILAVLLVSRGQRRLRAMRLVVMVVVAFALCWTPYHLVVLVDTLMDLGALARDCGRESRVDVAKSVTSGLGYMHCCLNPLLY

>lafCXCR4

LVMGYQKKLRSMTDKYRLHLSVADLLFVLTLPFWAVDAVAGWYFGKFLCQAVHVIYTVNLYSSVLILAFISLDRYLAIVHATNSQKPRKLLAEKVVYVGVWIPALLLTIPDFIFANVTESEEKYICDRFYPSDLWMVVFQFQHIMVGLILPGIVILSCYCIIISKLSHSKGHQKRKALKTTVILILAFFACWLPYYVGISIDAFILLEIIKQGCEFENTVHKWISITEALAFFHCCLNPILY

>lafCXCR6

VIYIFYQKLKSMTDLFLMNLPLADLVFVCTLPFWTYASIHEWVFGTSMCKILLGTYTLNFYTSMLILTCITVDRFLAVVQATKAYNQQAKRMIWAKVICLSMWVISLLVSLPQIIYGNVSIHDKLICDYKDEKISTVVLATQMALGFFLPLFTMIICYSVIIKTLLHARGFQKHKSLKIIFLVVAVFLLTQTPFNLMKLIRSTSWEYHAMTSFHYAIIVTEAIAYLRACLNPVLY

>lafCCR1

LVLMQYKRLRSMTSIYLLNLAISDLLFLFTLPFWIDYKLKDNWVFGDVMCKFLSGFYYTGLYSEIFFIILLTFDRYLAIVHAVFALRARTITFGIISSIVSWALAILASIPGWQFSKTQWEVSHYTCSLFFPYESLKMWKQFQALKLNILGLVLPLLVMIVCYTGIIKILLRRPNEKKAKAVRLIFVIMIIFFLFWTPYNLAVLVSAFQDSLFSNECEQSKQLDLAIQVTEVIAFTHCCLNPIIY

>lafCCR2

LTLINCKKLKSMTDIYLLNLAISDLLFLLTLPFWAHHAANGWVFGDSGCKIFTGLYHIGYFGGIFFIILLTVDRYLAIVHAVFALKARTVTFGVVTSGATWVVVVLVSLPGIIFTKSQEEDSHYVCGPAFPIIWKNLHTIMRNILSLVLPLLVMVICYSGILKTLLRCRNEKKHKAVRLIFAIMIVYFLFWAPYNIVLDLITFQEFFGLDNCDSSNRLDQAMQVTETLGMTHCCINPIIY

>lafCCR3

VILTKYRRLRIMTNIYLLNLAISDLLFLVTLPFWIHYTGWNKWVFGQCMCKFLSGFYYMGLYSEIFFIVLLTIDRYLAIVHAVFALRARTVTFGVITSIFTWVLAGLAALPEFAFHEFQEEDEYSACSPRYPENEEDTWKRFHALRMSILGLALPLLIMAICYTGIIKTLLRCPSRKKYKAIRLIFVIMVVFFIFWTPYNLVLLLSAFQVILPENNCEQSKQLDLAMAVTEVVAYTHCCVNPIIY

>lafCCR4

LVLIKFKRLKSMTDVYLLNLAISDLLFVFSLPFWGYYAADQWVFGLGLCKVISWMYLVGFYSGIFFIMLMSIDRYLAIVHAVFSLRARTLTYGVITSLATWSVAILASLPGLIFSTCYTERNHTYCKTKYSFNSTTWKVLSSLEINILGLAVPLGIMLFCYSMIIRTLQHCKNEKKNRAVKMIFAVVVLFLGFWTPYNVVLFLETLVELDILQDCAFERHLDYAIQATETLAFVHCCLNPVIY

>lafCCR5

LTLINCKKLRSMTDIYLLNLTISDLLFLLTLPFWAHYAANGWVFGNVVCKLFTGLYHIGYFGGIFFIILLTIDRYLAIVHAVFAVKARTVTFGVVTSGVTWVVAVLVSLPGIIFTRSQKEGSRYTCSPHFPSSQYHFWKNFQTLKITILGLVLPLLVMVICYSGILKTLLRCRNEKKKHKAVRLIFAIMIVYFLFWAPYNIVLDLSTFQGFFGLDNCDSSNRLDQAMQVTETLGMTHCCINPIIY

>lafCCR6

ITFAFYKKAKSMTDVYLLNMAIADILFVLTLPFWAVNHARGTWYFSNVLCKLIKGIYAVNFNCGMLLLTCISLDRYVAIVQATKSFRLRSKTLAHSKVICLVVWVASIIISSSTFIFSQKYNIQGVDVCEPKYHNVLEPVKWKLLMLVLQLLFGFFIPLMFMIFCYMFIVKTLVQAQNSKRHKAIRVIIAVVLVFLVCQTPHNMVLLVTAATMGSMGRSCSSEKLIAYTKNVTEVLAFLHCCLNPVLY

>lafCCR7

LTYIYFKRLKTMTDTYLLNLAMADILFLLTLPFWAYSAAKSWDFGVHFCKFIFGTYKVSFFSGMLLLLCISIDRYVAIVQAVSAHRHRARVLLISKLSCVGIWILAVVLSIPELLYSGIQKSSSEQALRCSLITEHVEALITIQVAQMVVGFLIPLMAMSFCYLVIIRTLLQARNFERNKAIKVIIAVVVVFIVFQLPYNGVVLAQTVANFNFTSSSCELSKQLNIAYDVTYSLACVRCCVNPFLY

>lafCCR8

LVLITCKKLRSITDVYLLNLALSDLLFVFSFPFLTHYHLDQWVFGTVMCKVVSGIYYIGFFSSMFFVTLMSVDRYVAVVHAVYALKVRTVSMGTVLSLAVWLIAIIATSPLLVFYQVASEDGILQCYSSYHQQTLKWKIFTYFEINILGLLIPFTILFFCYISILHQLRGCQKHKTKAIRLVLIVVAASLLFWVPFNVVLFLTSLHSMHVLDGCVISQRLIYATHVTETISFTHCCVNPVIY

>lafCCR9

LVYWYCTRVKTMTDMFLLNLAIADLLFLLTLPFWAIAAADHWKFHSVTCKVVNSMYKMNFYSCVLLIMCISVDRYIAIAQAMKAQNWRQKRLLYSKMVCITIWVIAAVLCIPEILYSQVRGESDVTVCTMVYPSKDSTNVKSAVLTLEVIVGFFLPLVVMACCYTIIIHTLLQAKKSSKHKALKVTITVLTAFLLSQFPYSCILLVQTINAYTMFISNCAISTNIDICFQVTQTIAFFHSCLNPVLY

>lafCCR10

THLAARRVARSPTSAHLLQLALADLLLALTLPFAAVGTLQGWSLGSATCRAISGLYSASFHAGFLFLACISADRYVAIARALPAGPRPSAPGQAHLVSAVVWLLSLLLALPALLFSRDGHREGQRRCRLIFPEGLTQTVKGASAVAQVALGFALPLSVMAACYALLGRTLLAARGLERRRALRVVVALVAAFVVLQLPYSLALLLDTADLLAARELSCPASKRKDLALLVTGALALARCGLNPVLY

>lafCX3CR1

FVLTNSQKPKSITDIYLLNLAFSDLLFVATLPFWIHYVISDQGFSNAVCKLITAFFFIGFFGGIFFITIISIDRYLAVVLATHSMRNRTVQHGVTISLSVWAVAILVAAPQFMFTKQNGSECLGDYPEILQEVWPVLQNVGANLLGFLLPLLTMSYCYLRIIWTLFSCKNHKKSKAIKLIFLVVIMFFLFWTPYNVMIFLETLKAFHFFPSCDVKKKLRLALSVTETIAFSHCCLNPLIY

>lafCCBP2

TVLLRYVPRRQMAEIYLMNLAISNLLFVVTLPFWGISAAWHWVFGNFLCKMVSTLYTINFYSGIFFISCMSLDKYLEIVHAQPHHRLRTRAKSLILSAVVWGVALAISIPDMVFVQTHENPKGMWRCYPDFGGHGTFWKLFLRFQQNLLGFLLPLLAMIFFYSRIGCVLVRLKPPGQSRALRMAAALVVAFFVLWFPYNLALFLHSLLDLQVFGDCNISHRLDYALQVTESIAFLHCCFTPVLY

>lafCCRL1

AIYAYYKKQRTKTDVYILNLAVADLLLLFTLPFWAVSAVHGWVLGKMMCKVTSALYTVNFVSGMQFLACISMDRYLAVTKAPSQSRVGRPGWIICSCVWTAAVLLSIPQLVFNTVNDNARCIPIFPHHLGTSVKAAIQMLEICIGFVVPFLIMGVCYFITARILIKTPNIKKSRPLKVLLTVVVVFIVTQLPYNIVKFCQAIDIIYSLITDCSMSKRMDVAIQVTESIALFHSCLNPILY

>lafCCRL2

LILAKYKGLRHVKNIYFLNLAFSNLLFSLTLPFWAYTASAGGSLGDTMHTILIGVSAVGLYSEVCFNVLLTVHRYLVQSPSPTPCTASCGILTSILVWTVAVLISLPECMAYTLQMEGQENKYSFSIPHFLPAAEKSWKHFLTLKMNILGLLFPLFIFIFCYVRMRRRLTCRDNENGLFKLVFAIMAVFLLMWAPYNVVLFLSTFKQDFSLDDCKSTYGLDQGIQITEIVAATHCFVNPLLH

>mdoCXCR1

LVILYNRISRSVTDIYLLNLAIADLLFALSLPIWAASKIKGWLYGTALCKIVSLLKEVNFYSGILLLACISVDRYLAIVHATRSLTQKRHWVKFVCLGIWGLSLLLSMPIILSREAFKSEDYGFVCYEDLGKKTEIWRLVLRILPQVFGFVLPLLVMLFCYGFTLRTLFEARMGQKHRAMKVIFAVVLIFLLCWLPYNLVLVADTLMRTHIIEETCERREEIDQAISVTEVLGFLHSCLNPIIY

>mdoCXCR2

LVILYNRISRSVTDIYLLNLAIADLLFALSLPIWAASKIKGWLYGTPLCKIVSLLKEVNFYSGILLLACISVDRYLAIVHATRTLTQKRHWVKFVCLGIWGLSLLLSMPIILSREAFKSDDYGFVCYEDLGKNTTTWRLVLRILPQVFGFVLPLLIMLFCYGFTLRTLFEAHMGQKHRAMKVIFAVVLIFLLCWLPYNLVLVADTLMRTHIIEETCGRREEIDQAISVTEVLGFLHSCLNPIIY

>mdoCXCR4

IVMGYQKKLRSMTDKYRLHLSVADLLFVLTLPFWAVDAAANWYFGNFLCKAVHVIYTVNLYGSVLILAFISLDRYLAIVHATNSQRPRKLLAEKVVYLGVWLPAVLLTVPDIIFASTSEAGGRYVCDRMYPHENWRISFRFQHILVGLVLPGLIILTCYCIIISKLSHSKGHQKRKALKTTVILILAFFACWLPYYIGISIDTFILLEVIKQDCDFDKAVHKWISITEAVAFFHCCLNPILY

>mdoCXCR5

VILKSHHMSRSSTETFLLHLAVADLLLVLTLPFAVVEGAVGWVLGAFFCKAVSALHKINFYCSSLLLACIAVDRYLAIVHAVHTYRHRRLLSVHATCAAVWLASFLCALPELLFVKVSNSGANNSATCSFSGQGLAGSNAWLTSRFLYHVGGFLIPLLVMSWCYAGVVRRLCQAQRRHQRQKAIKVAILVTGVFFFCWSPYHVVIFLDTLVMLDAVSKSCQLNDHLATAITTCEFMGLAHCCLNPVLY

>mdoCXCR6

AVYIFFQKAKSLTDQFLMNLPIADLLFLCTLPFWVYATIHEWVFGQVMCKVILGMYTLNFYTSMLFLTSITIDRLLAVVQATKTYNYQAKRMTVGKNLCATIWLISLTVTIPQFMYARVFPNDKRVCHNEEEGISTVVLSTQMTIGFFLPLIAMIVCYSVIVKTLVQAKRFQKHKSLKIIFLVVVVFIATQLPFNLMKLIRTTNWEYDTDPRFLYGLMVTEAIAYLRVCLNPVLY

>mdoCCR1

LILTKYKRLTSMTNIYLFNLAISDLLFLVTIPFWIHYEKQNDWVFGNAMCKLLTGLHYLGLYSEIFFIILLTVDRYVAIVHAVFAIRVRRVVLSVTTSIITWVIALLVSLPDIIFTKTQWEFTHYTCSLHFPHETARIWRKFQALKLNFLGLILPMWIMIACYTGIIRILLKRRNEKKWKAVKLIFAIMIIFFLFWTPYSITILISAFDEFIFTLDCEKSKQLDLAIQVTEVIAFTHCCVNPVIY

>mdoCCR2

LILIRCKKLKSMTDIYLLNLAISDLLFIVTLPFWAHYAADQWIFGDALCKVLTGFYHMGFFGGVFFIILLTMDRYLAIVHAVFALKARTVTFGILTSVITWVVAGFASLPAIIFTKSQKEGIQHTCSPHFPSEQSTLWKNFQTLKMNLLGLVVPLLVMIVCYSGIIKTLLRCRNEKKKHKAVRLIFVIMVVYFLFWAPYNLALLLNTFQDFFGLDNCESSKRLGRAIQLTETLGMTHCCINPVIY

>mdoCCR3

LILTKYKRFKSMTNIYLFNLAISDLLFLFTLPFWIDYARKNDWVFGHTMCKILSGLYYMGLYSEIFFIVLLTIDRYLAVVYAVFALKARTVTFGIFTSIVTWGLSALAALPEVIFHESQEDLENHICSPRYPEDNEATWKRFQALRMNILGLAIPLAIMIICYTGIIKKLLSCRNEKKYKAVKLIFVIMIVFFLFWAPYNLTLLLNAFQSSFFTADCERSKQLDLAMQITEVIAYTHCCVNPVIY

>mdoCCR4

LVLLKYKRLKSMTDVY

LFNLAISDLLFVFSLPFWVYYVAAHQWVFGTAFCKIISYMYLVGFYSGIFFIMLMSVDRYLAIVHAIFALRARTLTYGVITSLVIWLVAILASLPVLLFSTSYTENNHTYCKEKYPGNSTTWKVLRSLEVNILGLLIPLGIMLFCYTMIIKTLHRCKNDKKNKAVKMIFAVMIVFLVFWIPYNIVLFLEILVELEVLQDCTFDMQLDYALQATETLAFVHCCLNPVIY

>mdoCCR5

LVLIRCKKLKSMTDIYLLNLAISDLLFIVTLPFWAHYAADQWIFGDALCKVLTGFYHMGFFGGVFFIILLTMDRYLAIVHAVFALKARTVTFGILTSVITWVVAGLASLPAIIFTKSQKEGIQHTCSPHFPSEQFSAWKNFQTLKMNLLGLVVPLLVMIICYSGILKTLLRCRNEKKKHKAVRLIFVIMIVYFLFWAPYNLVLLLNTFQAFFGLDNCESSKRLDRAIQITETLGMTHCCINPVIY

>mdoCCR6

ITFAFYKKAKSMTDVYLLNMAIADILFILTLPFWAVNHATGSWKFSNIMCKLTTGIYAINFNCGMLLLTCISLDRYIAIVQATKSFRLRTWTLAYSKMICLMVWLFSIIISISTFVFNQKYTIQGRDVCEAKYHTTSEAVKWKILILVLQLLFGFFIPLLFMIFCYMFIVKTLVQAHNSKRHKAIRVIIVVVLVFLVCQVPHNMVLLVVASNIGRLRSCSDEKLISYTRSVTEVLAFLHCCLNPVLY

>mdoCCR7

LTYIYFKRLKTMTDIYLLNLALADILFLLTLPFWAASAAKSWMFGPFLCKAVYCIYKMSFFSGMLLLLCISVDRYFAIVQAVSAHRHRNRIILISQISCCVVWVLAFLFSIPELIYSNILKNGRCSLVTEDLETFTTIIQVSQMVIGFLIPLLVMFSCYLVIIRTLLQARNFERNKAIKVIIAVVIVFIVFQLPYNGVVLAKTVAALNKTATDCEHSKQLDIASDVTYSLACFRCCLNPFLY

>mdoCCR8a

LVLVACKKLRSMTDVYLLNLAISDLLFVFSFPFLTHYTLDQWVFGNIMCKTISGIYYIGFFSSIFFITIMSMDRYLAIVHAVYALKVRTTRKGMAVSLLVWMVATLASVPLLVFYQVSSEEGTLKCYSFYDDRTIEWKLITHFEINILGLVIPLSILVFCYANILRHLKGCQNRHKIKAIRLVLVVVVAFFLFWVPFNMMLFLNSLHNLHILDGCDLSQKLTQATQITEVISFTHCCVNPVIY

>mdoCCR8b

LVLVACKKLRSMTDVYLLNLAISDLLFVFSFPFLTHYTLDQWVFGNIMCKTISGIYYIGFFSSIFFITIMSMDRYLAIVHAVYALKVRTTRKGMAVSLLVWMVATLASVPLLVFYQVSSEEGTLKCYSFYDDRTIEWKLITHFEINILGLVIPLSILVFCYANILRHLKGCQNRHKIKAIRLVLVVVVAFFLFWVPFNMMLFLNSLHNLHILDGCDLSQKLTQATQITEVISFTHCCVNPVIY

>mdoCCR9

TVYWYCTRVKTMTDMFLLNLAIADLLFLLTLPFWAIAASDQWKFETVMCKLVNAIYKMNFYSSMLLIMCISIDRYIVIAQAMKAHHWRQKRLLYSKMVCFAIWVMAITLCIPEILYSQSETKSDITICTMVYNGSAMLKSTVLMLKVILGFFLPLIVMACCYAIIIYTLLQAKKSSKHKALKVTISVLTVFVLSQFPYNVILFVQAIDAYIVLISDCALSTRIDICFQVTQTIAFFHSCLNPVLY

>mdoCCR10

THLAAHRAARSPTSAHLLQLALADLLLALTLPFGAAGALQGWTLGSTLCRTVSGLYSASFHAGFLFLACISADRYVAIAQASPGSHRPPAPGRTHVISAAIWLLSLLLALPSLLYGQDGQRDGQRRCRLVFPEGLTQAVKGASAVAQVILGFALPLSVMVVCYALLGRTLLAARGPERRRALRVVVALVVAFVVLQLPYSLALLLDTTDLLTARERSCSASKRKDMALLVTGGLALARCGLNPILY

>mdoCX3CR1

FALTNSSKHKSITDIYLFNLALSDLLFVTTLPFWSHYLLHEEGFNNALCKLITAFYFIGYYSGIFFITIISIDRYVAIVLAANSLNSRTVQHGVVVSLGVWAAAILVATPQFMFTEKVDKECLSNYPDSLQHIWPILKNVEINSIGFVIPLLIMCFCYFGIIRTIFSCKNHRKTRTIKLISVVVGVFFLFWTPYNVMIFVDTLKFYGFFESCEVKNNLRLVINVTETFAFVHCCLNPFIY

>mdoXCR1

WVLVKYESLESLTNVFILNLCLSDLVFSCLLPFWVVVHYYDWIFGEFFCKLLNMLFSISLYSSIIFLMVMTTHRYISVVHPLSNLGGHSCWSRVGIILGIWMASIMVSVPDTIFHTVLTDKSCDFSEPKWFLLSTYLHNLFFIFSLVVTLFCYVQILRTLFQSHTRRRHRTVRLIFTIVVAYFLSWAPYNMVLFLQTLVKLGYIQNCEVIKKLNYWEHICREFAFSHCCFNPVLY

>mdoCXCR7

WVNLQAKTTGYETHLYIFNLAIADLCVVVTIPIWVVSLVQHNQWPMGELTCKIAHLIFSINLFGSIFFLTCMSVDRYLSVTYFSNTSSHKKKVVRRLICILVWLLAICVSLPDSYYLKTVTLAANNETYCRSIYPEHSIKEWLLGMELLSVILGFIIPFSIIAIFYFLLARAITASGDQEKHSSQKIIFSYVIVFLICWLPYHAVVLLDIFSFLHFIPFNCQLENFLYTALHVTQCLSLVHCCVNPVLY

>mdoCCBP2

IVLLYSARSRRVTEIYLLNLVVSNLLFTITLPFWGVSAAWHWGFGEVLCKIICTLYTTSLYGSIFFLGCMSLDK

YLDVVHAQTHHRQWTSAKSRLLTGGVWTVALVLSIPDLVFARLQEGPSGRQNCHLDFGENGPVWKLVLRFQQSALGFILPLFTMAFFYTRIVCVLTVLRPRGRSRALWRAALLVVTFFALWGPYNITLFLHSLQDLQVLESCEVSKHLDYALQVTESIAFLHSCLSPFLY

>mdoCCRL1

AIYAYYKKQKTKTDVYIMNLAVADLLLLITLPFWAVNAVHGWVLGIPMCKLTSALFTINFVSGMQFLACISIDRYSAITKDPGHQRLGRPCWIICFSVWLIAILLSIPQLVFNTVNDKKRCLPIFSHYLGTTIQASIQILEICIGFVLPFLIMGTCYSITARKLIKMPNVKKSRPLQVLLAVVAVFIVTQLPYNIVKFWQAIDIIYSLIIDCEMSKRMDVAIQITKSLALFHSCLNPILY

>mdoCCRL2

LILVKYKGLKVVVNIYFLNIAISNFLFLVTFPFSIHTAIHSWDLGGAMCKIISGFYSVGYYGYTCFLLLLIIHRYLAIVHTGRFHLATKKTTYGIIISSWGTAMLVTLPELVLSQVQMEDKDYICYFVQTYQYPPGDEKFWKYFLTLKMNILGILIPLFVFVFCFGGIKKTSRYKGRKHELLRLIFVITLVFIGLWTPYNLVLFLKTFQEHMNLSDCNSNYHLDKAIQVTKIIANIHCFISPIVY

>meuCXCR4

IVMGYQKKLRSMTDKYRLHLSVADLLFVLTLPFWAVDAAASWYFGKFLCKAVHVIYTVNLYGSVLILAFISLDRYLAIVHATNSQRPRKLLAEKVVYLGVWLPALLLTVPDIIFANTGEANGRYFCDRVYPHDNWLISFRFQHILVGLVLPGLIILTCYCIIISKLSHSKGHQKRKALKTTVILILAFFACWLPYYIGISIDTFVLLEVIKQDCDFDTAVHKWISITEALAFFHCCLNPILY

>meuCXCR5

VILKSHRTARSSTETFLLHLAVADLLLVLTLPFAMTEGAVGWVLGAFLCKAVSALHKINFYCSSLLLACIAVDRYLAIVHAVHTYRHRRLLSVRIACGAVWLASVLYALPELLFVTVSKIGTNDLLCSFAGQGLAGSDARLTSRFLYHIGGFLIPLLVMAWCYTGVVRRLCQAQRRHQRQKAVKVAILVTGVFFFCWSPYNVVIFLDTLVMLGTIQKSCQLDDHLATAIITCEFLGLAHCCLNPVLY

>meuCCR3

LILTKYKRFKSMTNIYLLNLSISDLLFLFTLPFWIHYARQNDWVFGHTMCKILSGLYYLGLYSEIFFIVLLTIDRYLAVVYAVFALKARTVTFGIFTSIVTWGLSALAALPEFIFHESQQDMENHICSPRYPEYDEDKWKRFQALRMNILGLAIPLAIMIICYTGIIKKLLGSRNDKKYKAVRLIFVIMIVFFLFWTPYNLTLLLSAFQASFFKGDCERSKQLDIAMQVTEVIAYTHCCVNPVIY

>meuCCR4

LVLLKYKRLKSMTDVYLFNLAISDLLFVFSLPFWVYYVADQWVLGTAFCKIISYMYLVGFYSGIFFIMLMSVDRYLAIVHAIFALRARTLTYGVITSLVIWSVAMLASLPVLLFSTSYTENNNTYCKEKYPGNSTTWKIMSSLEINILGLLIPLGIMLFCYTRIIKTLHRCKNDKKNKAVKMIFAVMVVFLLFWIPYNIVLFLEILVELEVFQDCTFGMQLDYALQATETLAFVHCCLNPVIY

>meuCCR7

LTYIYFKRLKTMTDIYLLNLALADILFLLTLPFWAVSAAKSWIFGPILCKVVYFTYKTSFFSGMLLLLCISVDRYFAIVQAVSAHRHRTRIVLISQISCGVVWVLALLFSTPELIYSNILKNGRCSLVTEDLVTFTTIIQVSQMVIGFLIPLLVMFSCYLIIIRTLLQARNFERNKAIKVIIAVVIVFIVFQLPYNGVVLVKTVAALNKTSTDCEHSKQLDIASDITYSLACLRCCLNPFLY

>meuCCR9

VMYWYCTRVKTMTDMFLLNLAIADLLFLFTLPFWAIDASDQWKFGTNMCKVINATYKINFYSYVLLIMCISIDRYIVIVQAMKAHRWRQKRLLYSKMVCSAIWVIAIILCIPEYLYSQSKVQYDTTVCTMVYPRDNNSLKAILSMLKFIMGFFIPVTVMASCYTIVTHTLMQAKKSSKNKALKVTILVLTVFVLSQFPYNIIILVQAIDAYRMFIIDCDISTYIDISFQVTQTIAYFHSCLNPVIY

>meuCCR10

THLAAHRTARSPTSAHLLQLALADLLLALTLPFAAAGALQGWTLGSILCRTVSGLYSASFHAGFLFLACISADRYVAIAQASPGGHRPPAPGKTHVVSAAIWLLSLLLALPSLLYGQDGQRDGQRRCRLVFPEGLTQAVKGASAVAQVILGFAVPLSVMVACYTLLGRTLLAARGPERRRALRVVVALVVAFVVLQLPYSLALLVDTTDLLTARERSCSSSKRKDMALLVTGGLALARCGLNPVLY

>meuCXCR7

WVNLQAKTTGYETHLYILNLAIADLCVVVTIPIWVVSLVQHNQWPMGELTCKVAHLIFSINLFGSIFFLTCMSVDRYLSVTYFANTSSYKKKVVRRLICILVWLLAICVSLPDAYYLKTITLAANNETYCRSIYPEHSIKEWLLGMELLSVVLGFIIPFSIIAVFYFLLARAISASGDQEKHSNQKIIFSYVIVFLICWLPYHAVLLLDIFSFLHFIPFSCQLENFLYTALHITQCLSLVHCCVNPVLY

>meuCCRL1

AIYAYYKKQKTKTDVYIMNLAVADLLLLVTLPFWAVNAVHGWVLGIPMCKVTSALFTINFASGMQFLACISMDRYSAITKAPGHQRVGRPCWIICVSVWLIAILLSIPQLVFNTVNDKKRCLPIFSHRLGTTIKASIQILEICIGFVLPFLIMGVCYSLTARTLIKIPNVKKSQALRVLLAVVAVFIVTQLPYNIVKFWQAIDIIYSLIIDCEMSKRMDVAIQITKSLALFHSCLNPILY

>oanCXCR1a

LVVLHNRPHRTVTDVYLLNLAGADLLFALTLPFWAAYQVTGWLFGTVLCKLVSVLQDLNFYSGVLLLACISVDRYLAIVHATRSAAHQRRWVVFVCLGLWILSFLLALPALVLRDVFTPPNSTPVCYENMGKDTARWRVILRLLQQTFGFLLPLLVMLFCYGRTLAVLFRARMGQKHRAMRVVLAVVLVFLLCWLPYNVILVIDSLMWVKVLTDSCGLRDHVDRALVVTPVLGYLHSCLNPIVY

>oanCXCR1b

LVVLHNRPHRTVTDVYLLNLAGADLLFALTLPFWAAYQVTGWLFGTVLCKLVSVLQDLNFYSGVLLLACISVDRYLAIVHATRSAAHQRRWVVFVCLGLWILSFLLALPALVLRDVFTPHNSTPVCYENMGKDTARWRVILRLLQQTFGFLLPLLVMLFCYGRTLAVLFRARMGQKHRAMRVVLAVVLVFLLCWLPYNVILVIDSLMWIQVLADSCGLRDHVDRALVVTPVLGYLHSCLNPIVY

>oanCXCR4

IVMGYQKKLRSMTDKYRLHLSVADLLFVLTLPFWAVDAAVSWCFGIFLCKAIHVIYTVNLYGSVLILAFISLDRYLAIVHATNSQRPRKLLAEKVVYVGVWLPAVLLTIPDLIFAETSQLDERYICERFYPHDTWLISFRFQHIVVGLILPGLIILTCYCIIISKLSHSKGHQKRKALKTTVILILAFFACWLPYYIGISIDTFILLGVIKQGCAFQNTVHKWVSITEALAFFHCCLNPILY

>oanCXCR6a

VVDSNLYTSMLLLTCITVDRFISVAQATKAHAYQSRRLTWGKITCICMWLISLAVTTPQFIYSTVSNQDKQVCLEFGNSDSRQVFLVALAAQMTIGFFLPLLAMIVCYSVIIKTLIRAKGFQKHKSLKIIFLVVAVFVLTQLPYNLVKLIVRAHWEYYTYPSFHYALVVTESIAYLRACLNPVLY

>oanCXCL6b

CGGFNLYTSMLLLTCITVDRFISVAQATKAHAYQSRRLTWGKITCICMWLISLAVTTPQFIYSTVSNQDKQVCLEFGNSDSRQVFLVALAAQMTIGFFLPLLAMIVCYSVIIKTLIRAKGFQKHKSLKIIFLVVAVFVLTQLPYNLVKLIVRAHWEYYTYPSFHYALVVTESIAYLRACLNPVLY

>oanCCR1

LILVKYKRLITMTNIYLLSLAFSDLLFLFTFPFWIHSELKGDWIFGNGMCKLLSCLHSVGLFSGIFIIILLTIDRYLAVVHAVFALKVRTVTVSFVSSAVAWILAGFTALPDFIFQKTQTKNLRRTCSLYFPHDTAETWKYLQALKQNILGFALPLLVMVVCYTGIIKRLLRQRGERKVVVVRLIFVIMLIFFIFWTPYNLVHLIFAFQEFIFENENECERSNQLDIAFQVTEVIAFTHCCANPVIY

>oanCCR2

LILKKYKKLKTMTDIYLLNLAISDLLFLFTLPFWAYYAADQWLLGDAACKIFSGLYYLGFFSGIFFIILLTVDRYLAIVHAVFALKARTVSCGVVTSVCTWAVALLASVPGFLFNRSQKAEDRYVCSTYFPMGQDLAWKIFQTLQMNILGLAVPLLVMIVCYTGIIKTLLRCRNEKKKHKAVRLIFLIMLIYFLFWAPYNLVLLLNTFQGFFGLNNCDSSNRLDRAMQVTETLGMTHCCINPVIY

>oanCCR3

LILIKYKRLRIVTNIYLLNLAFSDLLFLFTLPFWIHTEVKSEWVFGNSMCKFVSGLYYMGLYSEIFFIVLLTVDRYLAIVHAVFALQVRTVNFGIMTSAVTWGLAGLAALPSFVFSTSQRSLENHVCSPVYPDAKAENWKHFRTLKMNLLGLIFPMIVMVVCYAGIIKKLLKCRNEKKYKAVRLIFVIMIVFFLFWTPYNLVLLLNNFQMYFFEGECEKSKQLDIAMQVTEVITFIHCCANPVIY

>oanCCR4

LVLFKYKRLRSMTDVYLLNLAISDLLFVFSLPFWAYYAADQWIFGLGLCKIISWIYLVGFYSGILFIMLMSIDRYLAIVHAVFSLKARTFSYGVIASSVIWLVAILASFPTLLFSESFWEEGQISCKSKFPNNSTTWKVLSSLEINVLGLLIPLGVMLFCYSLIIKTLQHCKNDKKNKAVRMIFVVMIVFLVFWIPYNVVLFLEALVELELLKDCTFEKHLDYALQATETLAFVHCCLNPVIY

>oanCCR5

LILKKYKKLKTMTDIYLLNLAISDLLFLFTLPFWAYYAADQWLLGDAACKIFSGLYYLGFFSGIFFIILLTVDRYLAIVHAVFALKARTVSCGVVTSVCTWAVALLASVPGFLFNRSQKAEDRYVCSTYFPMGQDLAWKTFQTLQMNILGLAVPLLVMIVCYTGIIKTLLRCRNEKKKHKAVRLIFLIMIIYFLFWAPYNLVLLLNTFQSSFGLNNCDSSNRLDQAMQVTETLGMTHCCINPVIY

>oanCCR6

VTFAFYKKGKSMTDVYLLNMAITDILFVLTLPFWAVNHATESWIFDNFICKLTSGIYAINFNCGMLFLACISMDRYIAIVQATKSFRLRAKSFKFRMRTLAYSKVICSVVWMFSILISSATFIVSQKYQMQGKYICEPKYQVVSEAITWKLLILGLQLLFGFFIPLLFMIFCYTFIVKALVKAQNSKRRKVIRVIIVVVVIFLIFQVPYNMVLLINAINLGKLNRTCKSEQQRAYARNITQVLAFLHSCLNPVLY

>oanCCR9

LVYWYCSQMKTMTDMFLLNLAIADLLFLFTLPFWAVAAADQWKFQNTTCKLVNSMYQMNFYSSMLLLTCIGMDRHIVIVQATRAFSWKQKRVVYSKVICLCIWMVATALCIPELTFSQATETNDVMTCTVVYPDDGTTMLKASFMMLRFILGFFLPLLVMVCCYATIIHTLLQTKRSCKHRAFKVAIAVLIVFILSQFPFNSLLLVKTIATFKPSEDRAFISSCALSTKLDIGFQITQTLAFFHSCLNPVVY

>oanCX3CR1

FAVTNGGKKKSITDVYLLNLAFSDLLLVISLPFWIHYVVGEQVLGNILCKSASALFFIGFFGGMFFVTVISIDRFLAIVQAAGSIHSRTVQHGVSTSFSVWTVAILAAVPQFMYTEQVGDECVGNYPEVLQHMWPVLRNLEANLFGFLFPFLIMSYCYLRILKTLLFCNNRKKVRAAKLILMVVAVFFLFWTPYNILVFLETLNHFKFFPSCTLKRHLRFGLSVTETIALIHCCLNPFIY

>oanXCR1

CVLIRYENLESITNTFIFNLCLSDLVFSCLLPFWTTAHHLGWIFGDVLCKILNLLFSLSLYSSIIFLTVMTIHRYLSVVNPLSTLRTHTLRYRLLVSLAIWVTSLAAAIPDAIFHKVMTGSEKAVNPEFCDYFEPKWLLVSVCQHNVFFLFSMVIILFCYIEILRTLVRSRSRRRHRTVRLIFAIVMAYFLSWGPYNVLVFLETLVRFHIIPSCSLYKQLEYALHISREIAFSHCCFNPVLY

>oanCXCR7

WVNLQAKTTGYETHRYILNLAIADLCVVITLPIWVVSLIQHNQWPLGELTCKITHLIFSINLFGSIFFLTCMSVDRYLSITYFTNSSSRTKKVTRCVICTFVWLLAICVSLPDTYYLKTITSATSNETYCRSFYPEDSVKEWLIGMELVSVILGFAIPFAVIAVFYFLLARTITTSNDQEKHSNRKIIFSYVVVFLVCWLPYHAVVLLDIFSILHFIPFSCQMENFLYAALHVTQCLSLVHCCVNPVLY

>oanCCRL1

VVYSFYKKQKSKTDVYIMNLAVADLLLLFTLPFWAVNAVQGWVLGQTMCKVTSALYTINFVSGMQFLACISVDRYSAVTNAPRLEKIGKTCWVICFCVWIAAVLLSVPELIFNTVRKNKDRYRCLPIFPQHLGISMKASIQLLEISLGFAVPFLIMGVCYFITARTLIRTPNIKKSQPLKVLLAVVVVFIVTQLPYNIVKFWRAMDIIYLLITDCGMSKRIDVAIQITESVALFHSCLNPILY

>oanCCRL2

VILIKYKRLKLTVHLCLLNLAISNLFFLLTLGFGALTSRPGWALGYAMCQLISGIYSVGLFSQSFFIMFLAVQKYLTVVHRLTFHLKPKAGHWGIITSALIWGLATLASLPDFLLRQSPLKDESDTCRFSKPHYALSDEKSWMHFLTLRMSILGLLFPLLVAIFCSVGLIKVLCRHRIEQKVTLTLTIVMVFFLFWTPYNLVLFLSTFSKYFSLSDCESSYQLDRAIQVTKIIADTHCCVNPVIC

>ggaCXCR2

LVVTSGHINRSVTDVYLLNLAVADLLFALSLPLWAVYWAHEWVFGTVMCKAISVLQESNFYSGILLLACISVDRYLAIVYATRAATEKRHWVKFVCVGIWVFSVLLSLPVLLFREAFVSDRNGTVCYERIGNENTTKWRVVLRVLPQTFGFALPLLVMLFCYGVTVHTLLQTKNVQKQRAMKVILAVVLVFLVCWLPYNITLVSDTLMRTRAITETCERRKHIDTALSITQVLGFAHSCINPIIY

>ggaCXCR4

IVMGYQKKQRSMTDKYRLHLSVADLLFVITLPFWSVDAAISWYFGNVLCKAVHVIYTVNLYSSVLILAFISLDRYLAIVHATNSQRPRKLLAEKIVYVGVWLPAVLLTVPDIIFASTSEVEGRYLCDRMYPHDNWLISFRFQHILVGLVLPGLIILTCYCIIISKLSHSKGHQKRKALKTTVILILTFFACWLPYYIGISIDTFILLGVIRHRCSLDTIVHKWISITEALAFFHCCLNPILY

>ggaCXCR5

VILERFKRSRTTTENFLFHLTLANLALLLTFPFSVVESLAGWVFGTFLCKILSAVHKINFYLHEHAAGLHRVDRYLAIVYAIHTYRKARARSIHLTCTAIWLSSLLLTLPDLIFMEVWTDESNRSICYFPEAGIHGNNVWLATRFLYHSVGFFMPLLVMCYCYMAIVRTLCQSQRLQRQKAVRVAILVTGVFLLCWSPYHIVIFLNTLTKLEAFAKDCLLEDHLDTAIMVTEAIGFTHCCLNPIIY

>ggaCCR2

LILVKYKKLKSMTDIYLLNLAISDLLFVFSLPFWAYYAAHDWIFGDALCRILSGVYLLGFYSGIFFIILLTIDRYLAIVHAVFALKARTVTYGILTSIVTWAVALFASVPGIVFHKTQQENTQCTCSFHYPSDALINWQHSYILKMNILGLIIPMIIMIFCYSQILRTLFGCRNEKKQKAVRLIFVIMIFYFIFWTPFHVASFVHTFQTSFFSPDCDSQSRLEKTIQVTETISMVHCCINPVIY

>ggaCCR4

VVLFKYKR

LKSMTDVYLLNLAISDLLFVLSLPFWSYFMIDQWVFGTPWCKIISWIYLVGFYSGIFFIMLMSIDRYLAIVRAVFSMKARTAFHGLIASLTVWLVALLASVPELVFRESFVEQNYTTCKLRYPSNYLTWKLFYTLEINILGLLLPLIVMAFCYSMIIKTLLHCRNEKKNKAVRMIFAVMIVFFFFWTPYNIVILLQLLEATGVIRNCQASRNLDYASQITESLGLFHCCLNPVIY

>ggaCCR5

LILVKYKKLKSMTDIYLLNLAISDLLFIFSLPFWAYYAAHDWIFGDALCRILSGVYLLGFYSGIFFIILLTVDRYLAIVHAVFALKARTVTYGILTSIVTWAVALFASVPGIVFHKTQQEHTRYTCSAHYPQEQRDEWKQFLALKMNILGLVIPMIIMICSYTQIIKTLLQCRNEKKNKAVRLIFIIMIVYFFFWAPYNICILLRDFQDSFSITSCEISGQLQKATQVTETISMIHCCINPVIY

>ggaCCR6

MTFALYERTKSMTDVYLFNMAIADILFVLTLPLWAVNYAADKWIFGNFICKMAKGIYAINFSCGMLLLAFISVDRYIAIVQATKSFKLRARTLAYSKLICLAVWASAILISSSSFLYSESYDFATNETQICDHRFDKTSDSIVLKSLLLCLQVGFGFFIPFVFMIFCYAFIVKSLQQAQNSKRNKAINVIVLIVVVFLVCQVPYNTVLLMAVANMGKAEKSCDSDNIMAYAKYTTETIAFLHCCLNPVLY

>ggaCCR7

LTYIYFKRLKTMTDIYLLNLALADILFLLTLPFWATSAATFWCFGEFACKAVYCICKMSFFSGMLLLLSISIDRYFAIVQAASAHRFRPRMIFISKVTCILIWLLAFVLSIPELVHSGVNNYDSHPRCSIIASDLQTFSTGIKVSQMVFGFLVPLVVMSVCYLIIIKTLLQARNFEKNKAIKVIIAVVIVFVVFQLPYNGVMLAKTISVFNNTSSCDESKKLDMADDVTYTLACFRCCLNPFLY

>ggaCCR8a

WVLTAFKKIRAMTDVYLLNLAISDLVFVFSLPFLAQYSLVSQWTFGNAMCKIVSSAYFIGFYSSAFFITIMSIDRYLAIVHSVYALKVRTTKHGIIASLALWAVAILASVPGLVFFREVDEDNRTQCIPHYPGSGNSWKVFSNSEVNILGWLFPVSILIFCYHNILRNLQRCHTQNKYKAMKLVFIVVIVFFLFWTPINIMLLLDSLRSLHIIDDCQNSQRLDLALELAETLSLVHCCLNPIIY

>ggaCCR8b

WILLTRKRLMTMTDICLLNLAASDLLFIVPLPFQAYYASDQWVFGNALCKIMAGIYYTGFYSSIFFITLMSIDRYIAIVHAVYAMKIRTASCGTMISLVLWLVAGLASVPNIVFNQQLEIEQSVQCVPVYPPGNNIWKVTTQFAANILGLLIPFSILIHCYAQILRNLRKCKNQNKIKAIKMIFIIVIVFFLFWTPFNVVLFLDSLQSLLIIDNCQASSQITLALQLTETISFIHCCLNPVIY

>ggaCCR9

LIYCKYRFRRSMMDRYLLHLAVADLLLLFTLPFWATAASSGWIFRNFMCKVVNSMYKINFYGCILFLTCISFDRYLTIVQATKAKSSKQRRILRSKVVCFAVWLASVSLCLPEIMYSQSKQIGAVTVCKMTYPPNIGMAFRVAVLVLKVTIGFFLPLLVMVICYTLIIHTLLQAKRCQKHKSLKIITMIITAFLLSQFPYNIVLLIKTINMYTGAVYSCQTINGLDIGLQVTQSIAFLHSCLNPFLY

>ggaCX3CR1

LAIVKEGSKKSITDIYLMNLAVSDLLFVISLPFWASNTVRGWTLGTIPCKVVSSLYYIGFFGGMFFITVISIDRYLAIVRATYSMRSRTIKHSLLITCGVWATAVLVSVPHFVFSQMFENDCIPVLPQELMNIWPVFCNVELNTAGFFIPVCIICYCYCGIIKTLLYCKNQKKARAIKLTLAVVIVFFLFWTPYNVLIFLETLRHYELFISCNQIKSLDYAMHLTETIAFSHCCLNPLIY

>ggaXCR1

WILFKYENLTSLTNIFIMNLCISDLVFSCMLPFWAVDQTFGWIFGEFLCKAVNAIFSIGYYSGVFFLTLMTILRYLSVVSPLSTLRSQTQYCGSLVCLLVWTCSILIVVPEMIHTTVVETLEEVSTCDYDDWKWKKVDIYQRNILFLISFGIIIFCYINILIILLRTRSRRKHRTVKLILVIVVAFFLSWAPYNILSFLITFPPPTCQYEKDTLLAFHISRKIAFSHCCLNPVLY

>ggaCXCR7

WVNLQAKMTGYETHLYIFNLAVADLCVVITLPVWVVSLVQHNQWHMGEITCKITHLIFSINLYGSIFFLACMSVDRYLSVAYFTNSSNRKKKIVRRCICILVWLLAFSASLPDTYFLKTVSTSNETYCRPAYPEESFKEWLIGMELISVVLGFLIPFPVIALFYFLLAKTISASSDQERKSSGKIIFSYVVVFLVCWLPYHTTVLLDIFYSLHFIPFSCQMENFLYATLHITQCFSLVHCCVNPILY

>ggaCCBP2

TVLMYISKKKKMAEVYLLNLVVSDFLLLLTLPFWALFISQWVTWDLLCPVLNAMYIMNFYSGIFFVSCMSLDTYLQIVHACSPHSSVTRKKSFLLLLMLWVLSILLSIPDALFSSTKEMHNKTIVCTHDYGQKHLFWKVVFQVTQNILGFLLPFFFMVFCYSRSMCVLTTSRVPGSRTALRFVFILVAVFFVLWFPYNVVLILHSLQFVGLIQSCERSRQLDYAIQITESLSFVHCCLNPVLY

>ggaCCRL1

AIYAYCKKPKTKTDVYIMHLAIADLLLLFTLPFWAANAVQGWELGNSMCKLTSSLYTMNFSSSMLFLACISLDRYRATSESQRHRRAGKHCSVTCICVWLSAVLLSIPELIFNQVITHNNRNECLPIFPTNMETLLKATIQILEVILEFLLPFLVMVTCYSVTARAIFRSANAKKSRPFMVLLAVVAAFIITQLPYNIIKFWRAIDSIYLLITDCEASKTIDVALQVTKSIALFHTCLNPILY

>tguCXCR4

IVMGYQKKQRSMTDKYRLHLSVADLLFVITLPFWSVDAAIGWYFGNVLCKAVHVIYTVNLYSSVLILAFISLDRYLAIVHATNSQRPRKLLAEKVVYVGVWLPALLLTVPDLIFASTSEIERKYLCDRMYPHENWLISFRFQHILVGLVLPGLIILTCYCIIISKLSHSKGHQKRKALKTTVILILAFFACWLPYYIGISIDTFILLGVIRRRCSLETIVHKWISITEALAFFHCCLNPILY

>tguCXCR5

VILERFKRSRTTTENFLFHLTLANLALLLTFPFSVVESLAGWVFGKFLCKILSAVHKINFYCSSMLLGCIAVDRYLAIVYAIHTYRKRRAHSIHLTCMAVWLCSLLLTLPDLIFMEVWTDDGNRSICYFPEVGIDGNNAWLATCFLYHTVGFFVPLLVMCYCYTAIIRALCQSQRLQRQKAVRVAILVTGIFLLCWSPYHIVIFLNTLTKLEAFTKNCLLEDQLDTAIMVTEAIGFTHCCLNPILY

>tguCCR2

LILVKYKRLKSMTDIYLLNLAISDLLFVFSLPFWAYYAVHDWIFGEALCRILSGVYFLGFYSGIFFIILLTLDRYLAIVHAVFALKARTVTYGVLTSVVTWALAVLISVPGVVFHKTQKESSGYTCSTHYPSDSTINWKYSFILKMNILGLIVPMLIMIFSYSQILKILLRSKNEKKQKAVRLIFVIMIFYFIFWTPFHISSFLHTFQDSFFITDCELKGQLEKAIQVTETISMIHCCINPVIY

>tguCCR4

VVLFKYKRLKSMTDVYLLNLAISDLLFVLSLPFWSYFTVDQWVFGTPWCKIISWIYLVGFYSGIFFIMLMSIDRYLAIVRAVLSLKARTTFHGLITSLVVWLVALSASVPELVFRESFNEHNFTTCKPRFPGNFTTWKLFSTLEVNILGLLIPFIIMTFCYSMIIKTLVHCRNDKKNKAVKMIFVVMIVFFFFWTPYNIVIFLQLLEFMGVIKDCQVSRSLDYAFQVTEILGLFHCCLNPVIY

>tguCCR5

LILVKYKRLKSMTDIYLLNLAISDLLFVFSLPFWAYYAVHDWIFGEALCRILSGVYLLGFYSGIFFIILLTLDRYLAIVHAVFALKARTVTYGVLTSVVTWALAVLISVPGVVFHKTQKESSGYTCGAHYPSEQRNTWKQFLTLNMNILGLLIPMLIMICSYTQIIKTLLQCRNEKKHKAVRLIFIIMIIYFFFWAPYNICILLRDFQGAFSISTCEGNGQLHKAIQVTETISMIHCCINPVIY

>tguCCR6

MTFA

LYERAKSMTDVYLFNMAIADILFVLTLPLWAMNYATDEWIFGDFICKMTRGIYAINFSCGMLLLAFISVDRYIAIVQATKSFKLRARTLAHSKLICLAVWISSILISSPSFLYSESYSFSINETKEICDHRFGRISESTMLKSLLLWLQVAFGFFIPFIFMIFCYTFIVKSLQQAQNSKRNKAIRVIVLIVAVFLICQVPYNIVLLITAVNMGKIDKSCDNDKIMAYAKYTTEAIAFLHCCVNPVLY

>tguCCR7

LTYIYFKRLKTMTDVYLLNLALADILFLLTLPFWATSATTHWLFGSFACKAVYCICKMSFFSGMLLLLSISIDRYFAIVQAASAHRLRPRMIFISKVTCILIWLLAFILSTPELVHSGVNNMDSYPRCSIIANDLQTFSTGIKVSQMVFGFLIPLLVMSFCYLIIIKTLLQARNFEKNKAIKVIIAVVIVFVVFQLPYNSVMLAKTISAFNQTSSCEESKKLDVADDVTYTLACFRCCLNPFLY

>tguCCR8a

WILTVFMKIKTMTDVYLLNLTLSDLLLVFSLPFLVQYSVVSQWTFGNALCKIISSVYFIGFYSNVFFITIMSIDRYLAIVHSLHVQGIRTAAIGFITSLVVWVVAILASLPDLLFFQEVNDNNQIKCLPHYPSGSNGWKTFSNFEVNILGWLIPVFVLIFCYHSILKNLQKCHTKNKYKAIKLVFIVVILFFLSWTPVNIVLFLDSLRNMHIINDCQTSQRLDLAVELTEALSYVHCCLNPVIY

>tguCCR8b

WVLLTRKKLTTMTDICLLNLAASDLLFVLPLPFQAHYASDQWVFGNAMCKIMAGIYYTGFYSSIFFITLMSVDRYIAIVHAVYAMRIRTATCGIIISLILWLVAGLASVPNILFSQELEIEQALQCVPKYPPGDNTWKVASQFAANILGLLIPFSILFCCYTQILKNLQKCKNRNKVKAIKMIFIIVIVFFLFWTPFNIALFLDSLQSLHIINDCKASSQIALALQLTETISFIHCCLNPIIY

>tguCCR8c

WILTVFMKIKTMTDVYLLNLTLSDLLLVFSLPFLVQYSVVSQWTFGNALCKIISSVYFIGFYSNVFFITIMSIDRYLAIVHSLHVQGIRTAAIGFITSLVVWVVAILASLPDLLFSQEVNDNNQIKCLPHYPSGSNGWKTFSNFEVNILGWLIPVFVLIFCYHSILKNLQKCHTKNKYKAIKLVFIVVILFFLSWTPVNIVLFLDSLRNMHIINDCQTSQRLDLAVELTEALSYVHCCLNPVIY

>tguCCR9

LVYCKYHFRRSMMDLYLLHLAIADLLLLFTFPFWAKAASDGWIFKDFMCKVVNSMYKINFYGCSLLLTCISFDRYITIVQAMKARTCKRRWLLRSRLMCLAVWLTSVSLCIPELIYSQSTEVGDVTVCKIMYPPNISVIFRVTVLALKVIIGFFLPLLVMVICYALITNTLLQAKRFQKQKSLKIITMILTAFLLSQFPYNIVLLVKAIDTYTGVVHSCQAANQLDIGLQVTQIIAFLHSCLNPFLY

>tguCX3CR1

FAIVKGNKKSITDIYLLNLAVSDLLFVISLPFWASNTVRGWTLGTIACKAVSSLYYIGFFGGMFFITVISVDRYLAIVRATYSLKSRTIRHGFLVTCGVWAIAVLVSVPHFVFSQLIENDCIAVYPEKLENIWPVFRNVELNTIGFFIPVCIIFYCYCGIIKTLLSCKNQKKARAIKLILIVVVVFFLFWSPYNVLIFLDTLNHYELFTNCNQIKTLDYAMHLTETIAFSHCCLNPLIY

>tguXCR1

WILLKYENLTSLTNIFIMNLCISDLVFSCMLPFWVVDQSFGWIFGEFLCKASNAIFSIGYYSGVFFLTLMTILRYLFVVNPLSTLRSQTQCCGVLVSLAVWTVSILIVVPEVIHTTVQKDLEEHRYCDYADGNWKKVDIYVRNVLFLFSFGVIIFCYFKILIILLRARSRRKHRTVRLILIIVVAFFLCWAPYNILSFLTTFPPPTCQYVKDSSLAFHISRKIAFSHCCLNPVLY

>tguCXCR7

WVNLQAKMTGYETHLYIFNLAIADLCVVITLPVWVVSLVQHNQWHMGEITCKITHLIFSINLYSSIFFLACMSVDRYLSVAYFTNSSSRKKKIIRRCICILVWLLAFSASLPDTYYLKTVSSNNETYCRPVYPEESFKEWLIGMELISVVLGFLIPFPIIAVFYFLLAKSISTSSDQERKSNGKIIFSYVVVFLVCWLPYHVAVLLDIFYSLHFIPFSCQMENFLYATLHITQCFSLVHCCVNPILY

>tguCCBP2a

VLLMHIKKKKKMTELYLMNLVVSDFFLLLTLPFWALYISQWVTWDILCPFLSAMYTLNFYSGIFFVSCMSLDMYLQIVHAWSPHSSTVWRNSILILLVMWILSIALSIPDGLFTSTRQTHNKTIMCAQDYGQEHLFWKVVFRVTQNILGFLFPFLFMTFCYSRIACVLNTSQIPGSRRALCLVLTLVGVFFVLWCPYNVVLILHSLQDVGVIRSCESSRKLDYALQITESLSFVHCCLNPLLY

>tguCCBP2b

VLLMHIKKKKKMTELYLMNLVVSDFFLLLTLPFWALYISQWVTWDVLCPFLSAMYTLNFYSGIFFVSCMSLDMYLQIVHAWSPHSSTVWRNSILILLVMWILSIALSIPDGLFTSTRQTHNKTIMCAQDYGQEHLFWKVVFRVTQNILGFLFPFLFMTFCYSRIACVLNTSQIPGSRRALCLVLTLVGVFFVLWCPYNVVLILHSLQDVGVIRSCESSRKLDYALQITESLSFVHCCLNPLLY

>tguCCRL1a

AIYAYCKKPKTKTDVYIMHLAIADLLLLFTLPFWAANAVQGWELGNPMCKLTSSLYTMNFSSSMLFLACISVDRYRATSDSQGHRRVGKHCSVTCICVWLAATFLSIPELIFNQVKKHNERNECLPVFPMNMETLLKSTIQILEIILEFLLPFLVMLICYSATAWAIFRSANVKKSRPFKVLLAVVATFIVTQLPYNIVKLWRAIDIIYILVTDCHTSKIMDVALQVTKSIALSHACLNPLLY

>tguCCRL1b

AIYAYCKKPKTKTDVYIMHLAIADLLLLFTLPFWAANAVQGWELGNPMCKLTSSLYTMNFSSSMLFLACISVDRYRATSDSQGHRRVGKHCSVTCICVWLAATFLSIPELIFNQVKKHNERNECLPVFPMNMETLLKSTIQILEIILEFLLPFLVMLICYSATAWAIFRSANVKKSRPFKVLLAVVATFIVTQLPYNIVKLWRAIDIIYILVTDCHTSKIMDVALQVTKSIALSHACLNPLLY

>aplCXCR2

LVVTSSHANRSVTDVYLLNLAVALTLPLWAAYRAHEWVFGTVLCKAISVLQEANFYSGILLLACISVDRYLAIVYATRAATEKRHWVKFVCLAIWLFSVLLSLPVLLFREAFVSPSNGTVCYERIRGEDTAKWRVVLRVLPQTFGFALPLLVMLFCYGVTVRTLLRTKNAQRQRAMKVILAVVLVFLVCWLPYNITLVSDTLMRTRAIAETCERRNHIDTALSVTQVLGFSHSCLNPIIY

>aplCXCR4

VVMGYQKKQRSMTDKYRLHLSVADLLFVITLPFWSVDAAISWYFGNVLCKAVHVIYTVNLYSSVLILAFISLDRYLAIVHATNSQRPRKLLAEKVVYVGVWLPAVLLTVPDIIFASTSEVEGKYLCDRIYPHENWLISFRFQHILVGLVLPGLIILTCYCIIISKLSHSKGHQKRKALKTTVILILAFFACWLPYYIGISIDTFILLGVIRHRCSLETIVHKWISITEALAFFHCCLNPILY

>aplCXCR5

VILERFKRSRTTTENFLFHLTLANLALLLTFPFSVVESLAGWVFGTFLCKILSAVHKINFYCSSMLLGCIAVDRYLAIVYAIHTYRKRRARSIHLTCTAVWLCSLLLTLPDLIFMEVWTDESNRSICYFPEIGIHGNNAWLATRFLYHTVGFFVPLLVMCYCYMAIVRTLCQSQRLQRQKAVRVAILVTGVFLLCWSPYHIVIFLNTLTKLEAFTKNCLLEDQLDTAIMVTEAIGFTHCCLNPILY

>aplCXCR6

IILVFYEKLKTLTDIFLLNLAIADWIFLWTLPFWAYSAAQEWIFGTVTCRIIRGLYNLNLYTSMLTLTSITFDRLISITFATKAHMSQTKRLKWGKLICGLIWVISVAFATPQLIFSDVFTIDKTICLEKYPDHHIELVLEVIQVTLGYFIPMLTMIICYSLIIKTLLHARNFQKNKSLKKIFSVVAIFILTQSPYTFLRLMKIIDWSFNLDSNFDYAIVITEALAYFHGCLNPVMY

>aplCCR2

LILIKYKRLKSMTDIYLLNLAISDLLFIFSLPFWAYYAVHDWIFGEALCRILSGVYLLGFYSGIFFIILLTIDRYLAIVHAVFALKARTVTYGILASIVTWAVAMLASVPGIVFHKTQKENSRYTCSAHFPSDSSINWKYSYILKMNILGLVIPMFIMIFSYSQILKTLLGCRNEKKQKAVRLIFVIMIFYFIFWTPFHIASFLHTFQSSFFDPDCETQSKLEKAIQVTETISMIHCCINPVIY

>aplCCR4

VVLFKYKRLKSMTDVYLLNLAVSDLLFVFSLPFWSYFAIDQWVFGTPWCKIISWIYLVGFYSGIFFIMLMSIDRYLAIVRAVFSLKARTTFHGLITSVIVWLVALSASVPELVFRESFHEQNYTTCKHRYPGNFTTWKLFSTLEINVLGLLIPFIVMAFCYSMIIKTLVHCRNEKKNKAVRMIFAVMIVFFFFWTPYNIVIFLQFLELIGVIRDCQASRNLDYAFQITEIFGLFHCCLNPVIY

>aplCCR5

LILIKYKRLKSMTDIYLLNLAISDLLFIFSLPFWAYYAVHDWIFGEALCRILSGVYLLGFYSGIFFIILLTIDRYLAIVHAVFALKARTVTYGILASIVTWAVAMLASVPGIVFHKTQKENSQYTCSAHYPSDQRNVWKQFLTLKMNILGLVIPMLIMICSYTQIIKTLLQCRNEKKHKAVRLIFIIMIVYFFFWAPYNICILLRDFQGSFSISSCEGSGQLHKATQVTETISMIHCCINPVIY

>aplCCR6

MTFALYERTKSMTDVYLFNMAIADILFVLTLPLWAVNYAADKWIFGNFICKMARGIYAINFSCGMLLLAFISVDRYIAIVQATKSFKLRARTLAYSKLICLAVWVSSILISSSSFLYSESYNFSVNETKNICDHRFDKMAESTMLKSLLLCLQVGFGFFLPFIFMFFCYTFIIKSLQQAQNSKRNKAIRVIVLIVAVFLVCQVPYNIVLLVAAVNMGKMDKSCDSEKAMAYAKYTTEAIAFLHCCLNPVLY

>aplCCR7

VTYIYFKRLKTMTDIYLLNLALADILFLLTLPFWATSAATYWCFGETACKAVYCICKMSFFSGMLLLLSISIDRYFAIVQAASAHRFRPQMILISKITCVVIWVLAFILSIPELVHSGVNNPDNHPRCSIIANDLQTFNTGIKVSQMVFGFLFPLVVMSVCYLIIIKTLLQARNFEKNKAIKVIIAVVIVFIVFQLPYNSVMLAKTISAFNHTTSCEESKKLDMADDVTYTLACFRCCLNPFLY

>aplCCR8a

WVLLAFKKVRAMTDIYLLNLAISDLLFVFSLPFLVQYSLVSQWTFGNAMCKIVSSAYFIGFYSSSFFITIMSIDRYLAIVRSVYALRVRTSAHGVIASLALWAVAILASAPDLIFFQEMDDSNRTVCLPHYPGSDNSWKIFSNFEVNVLGWLIPVGILIFCYHNILKNLQRCHTRNKYKAMKLVFIVVTVFFLFWTPVNVVLFLDSMRSMHIIDDCQASQKLDLALELAEALSFVHCCLNPIIY

>aplCCR8b

WVLLTRKKLMTMTDVCLLNLAASDLLFVVPLPFQAHYAAEQWVFGNAMCKIMAGIYYTGFYSSIFFITLMSIDRYIAIVHAVYAMKIRTTSCGIIISLILWLVAGLASVPNIVFNQQLEIEQSMQCVPTYPPGSNTWKVASQFAANILGLLIPLSILICCYAQILKNLQKCKNRNKIKAIKMIFIIVIVFFLFWTPFNVVLFLDTLQSLHIINDCQASNRIALALQLTETISFIHCCLNPVIY

>aplCCR8c

WVLLAFKKVRAMTDIYLLNLAISDLLFVFSLPFLVQYSLVSQWTFGNAMCKIVSSAYFIGFYSSSFFITIMSIDRYLAIVRSVYALRVRTSAHGVIASLALWAVAILASAPDLIFFQEMDDSNRTVCLPHYPGSDNSWKIFSNFEVNVLGWLIPVGILIFCYHNILKNLQRCHTRNKYKAMKLVFIVVTVFFLFWTPVNVVLFLDSMRSMHIIDDCQASQKLDLALELAEALSFVHCCLNPIIY

>aplCCR9

LIYCKYRFRRSMMDRYLLHLAVADLLLLFTLPFWAKAASDGWVFRNFLCKVVNSMYKINFYGCILFLTCISFDRYITIVQATKAKTSKRRRLLHNKLVCLAVWLTSIGLCIPEIMYSQSKQVGDMTVCKMMYPPNVSMVFRVAVLALKVTIGFFLPLLVMVICYTLIINTLLQAKRCQKQKSLKIITMIITAFLLSQFPYNIVLLVKTINTYTGVVYSCWATNGLDIGLQVTQSIAFLHSCLNPFLY

>aplCX3CR1

FAILRAGSKKSITDIYLLNLAVSDLLFVVSLPFWASNTVRGWTLGTIPCTVVSSLYYIGFFGGMFFITVISIDRYLAIVRATYSLKSRTMKQGFLITCGVWATAVLFSVPHFVFSQLLENDCIPVFPQELENIWPVFCNVELNTIGFLIPVCIICYCYCGIIKTLLSCKNQKKTRAIKLTLVVVVVFFLFWSPYNVLIFLETLKHYELFVNCQQIKSLDYAMHLTETVAFSHCCLNPLIY

>aplXCR1

WILFKYENLVSLTNIFIMNLCVSDLIFSCMLPFWVVDQTFGWIFGEFLCKAMNAIFSIGYYSGVFFLTLMTILRYLSVVNPLSTLRSPTQCCGSVVSLVVWTGSILIVVPEVMHTTVHEDVYGYKTCDYNDWKMKKVDVYQRNVLFLFSLGIIIFCYLRILIILLGTRSRRKHRTVKLILIIVMAFFLSWAPYNILSFLLTFPPSTCQYEKDINLAFHISRKIAFSHCCLNPVLY

>aplCXCR7

WVNLQAKMTGYETHLYIFNLAVADLCVVITLPVWVVSLVQHNQWHMGEITCKITHLIFSINLYGSIFFLACMSVDRYLSVAYFTSSSNRKQKIVRRCICILVWLLAFSASLPDTYFLKTVSSNNETYCRPVYPEESFKEWLIGMELISVVLGFLIPFPVIALFYFLLAKAISASSDQERKSSGKIIFSYVVVFLVCWLPYHATVLLDIFYSLHFIPFSCQMENFLYATLHITQCFSLVHCCVNPILY

>aplCCBP2

VLVLYIRKKKKMTEVYLLNLVVSDFFLLLTLPFWAMYISQRVTWDMLCPFLNAMYTMNFYSGVFLVSCMSLDMYLQIIDACSPRSFTTQRKSILVLVAVWVLSILLSVPDGLFTTTKHTNNQTIVCTRDYGQEHLFWKVVFQVIQNVLGFLFPLLLMVFCYSRIACVLTTSRMPGLRRALCFVFTLVGVFFVLWFPYNIVLILHSLQDVGVIRSCERSRQLDYAIQITESLAFVHCCLNPLLY

>aplCCRL1

AIYAYCKKLKTKTDMYIMHLAIADLLLLFTLPFWAANAVQGWELGTSMCKLTSSLYTMNFSSSMLFLACISVDRYRATAGTQGHRRGGKRCSMTCCCVWLAAVLLSIPELIFNQVKKHNNRNECLPVFPVNMETLLKATIQILEIILEFLLPFLVMLTCYSVTARAIFRSANAKKSRPFMVLLAVVAAFIITQLPYNIVKFWRAIDIIYLLITDCDASKTIDIALQVTKSIALFHACLNPLLY

>acaCXCR1

LVVAYNKRNRSVTDVYLLNLAIADLLFALTLPIWAVFRAHEWIFGTGMCKFTSVLKEVNFYSGVLLLAFISVDRYLAIVYATRHATEKRHWVKFVCVGIWVFSLLLSLPMVTYREVFHAPNSSLRVCYENIGGNETSKWRVVLRILPQTFGFLVPLAIMLFCYGVTVHRLFQMKNNQKKKAMKVILVVVLVFLFCWLPYNITLFADTLMRTGVITEDCKRRGIIDAGLSGTEILGFSHSCMNPIIY

>acaCXCR2

LVVTYNKGNRPVTDVYLLNLAIADLIFALTLPIWAIFRAHEWIFGTAMCKIISAMKEVNFYSGILLLAFISIDRYLAIVYATHHVTEKRHWVKFVCVGIWVFSLLLSLPMITYREVVHAPNSAQMVCYENIGGNETTKWRVVLRILPQTFGFLVPLAVMLFCYGVTVHRLFQMKNNQKKKAMKVILVVVLVFLFCWLPYNISLLIDTLMRTRVIAETCGLQDAIDAALLGTEILGFSHSCMNPIIY

>acaCXCR3

AVLLRAKEALAGTDVFLFNLAVADILLVLTLPFWAVQEARGWVFGTFLCRVVGGAFKINFFASIFFLVCISLDRYLSIVCVVRMYRRSKASAVHLTAVAVWVACLLLTVPDFVYLSAEYDSRQKSTSCSLVFPPDSATQWKVGLSLFNQVGTFFLPLLAMGYCYAHIVFTLLLSKGFRKHKAMRVILAVVGAFFLCWLPYHSIQFATTFLKLDCAWQERLEVAEVVATALGFFHCCLNPLLY

>acaCXCR3L

AVLSSRRCPWLLADRFLFQLAVADLLLVLVLPFRATQFSQSWAFGEPFCKLVGALSAMSSYSTAFLLACVTLERYLAIVHSLQPRWTPHGALLASTLLWAASIALSVVELHFRTVSYVSQAGAVVCHLGFDARDANTWRLSLRLVSFLLGFLFPVAVMVYCYVRMLVKLRQLFFRVMALRLLSVILLLFVLCWGPFHGFVLVDSLQRLGHVGRDCAKEKILDFGLLFTESVGLVHSCLNPLVY

>acaCXCR4

VVMGYQKKLRSMTDKYRLHLSVADLLFVITLPFWSVDAVISWYFGNFLCKAVHFIYTVNLYSSVLILAFISLDRYLAIVHATNSQRPRKLLAERIVYVGVWLPALLLTVPDIIFASTSEVGGKYVCQRFYPHETWLISFRFQHILVGLVLPGLIILTCYCIIISKLSHSKGHQKRKALKTTVILIVAFFACWLPYHIGISIDTFVLLGFIENGCTFEAILQKWISITEALAFFHCCLNPILY

>acaCXCR5

VTLWRYRRARTPTEVFLFHFALANLLLVAMFPFGAAESLAGWVFGTVLCKGLSAATRVSFYSSSLLLAGISVDRYLAVVHALRTFQRPRSLSVHLTCLAVWLLSVLLAMPDLLFTEVWPDSGNLSICYFKKYGEQGVRSWLATRFLYHVVGFFLPAAVMCFCYLAIVRLLCRSQRLQRQKAVKVAILVTCVFLLCWTPFHVVTFWDTLTRLAPNSCAHEYSLAAAIALTELLGYSHCGLNPFLY

>acaCXCR6

IILIFYEKVKVLSDIFLVSLAIADLCFLCTLPFWAYMAADEWIFHTLPCKLIRGLYTMNLYGSMLTLTCITIDRYFAVVQATKAHVSQAKRRTWGIAACILVWVISLAFAMPQFIFSTEASNGKKVCHSSYPSEDTHKFTEVIQMVLGFFFPIVVMVVFYSIIVNTLFKAKGFHKHKSLQIIFAIVVAFILTQTPYNILKIIRAVDKHVAMHFNFDYGLVITEAIAYFHGCLNPILY

>acaCCR2

LILIKYKKLKNMSDIYLLNLAISDLVFIISLPFWAYYAANEWVFGNAVCKILSGVFRAGFYSGSFFITLLTIDRYLAIVHAIFALRARTVFYGTFSSAITWVVATLASVPALLFSHVQKEGESCKCNLFYPPGKEEEWKQVVTLMMFILGLAIPLAIMIFCYYQIIWVLIKGQNERKRKVVRLIFAIMIVYFILWMPYTITSLLHTYQNAFFSCGLDADCDGNFALALEVTEVIAMIHCCLNPLIY

>acaCCR4

LVLFKYKRLRSMTDIYLLNLAISDLLFVFALPFWSYFVADEWVFGDGLCKFISWVYRTGFYSGIFFIMLMSIDRYFAVVHVVFALKARTVSYGTLASLVVWLVAITASFPELIFSEAKSDYNHTECKSVYGKNDTMWKLFTALETNILGLLIPFMVMLFCYTHIVKTLMHCRNEKKKRAVKMIFAVMIVFFVFWTPYNIVLFLQYLLDVDILTGCSISKNLDYADQVTQTLAFFHCCLNPVIY

>acaCCR5a

LILIRYKKLKSMTDIYLLNLAISDLLFVVSLPFWAYSAAHEWIFEDAMCKILSGIYVVGFYSGSFFIILLTIDRYLAIVHAVFALKARTVTYGIVTSAVTWCVAILASIPWLIFNKLQRENNHCRCTLSFPPETHVNWNQFLTLKINLIGLIFPMIVMIFCYTQIIITLMRCRNDKKNKAVRLIFIIMIIYFLFWAPYNIVLLLQTFQTSFRLDNCYSYSNLGVALQVTETLAMAHCCINPVIY

>acaCCR5b

LILIRYKKLRSMTDIYLLNLAISDLLFVVSLPFWAYSAAHEWIFEDAMCKILSGIYVVGFYSGSFFIILLTIDRYLAIVHAVFALKARTVVYGIATSAVTWCVAILASIPWLIFNKLQQENNHCRCTLHFPPETHVNWNQFLTLKINLIGLIFPMMVMFFCYTRIIVTLMRRRNDKKNKAVRLIFIIMIIYFLFWAPYNIVLLLQTFQTSYHLDNCYSYSNLGVALQVTETLAMAHCCINPVIY

>acaCCR6

MTFALYKKTESMTDLYLCNMAIVDILFVLTLPFWAVNYALNRWIFGDFMCKLIKGIYALNFVCGMLLLACISMDRYISIVQATRSFKFRSRTLAYRKVICLTVWVASILISCPTFIFSGSYQSTNVSNDICEHKSSTEFDVTLKLLIINIQLFFGFFIPMLFMVFCYTFIVKKLVQAHNSKRSKAIRVVVSIVIVFLICQVPYNMVLLVTAATMKTLDKTCQSEKQMAYAKYITETFAFLHCCMNPVLY

>acaCCR7

LTYIYFKRLKTMTDIYLLNLALADILFLLTLPFWAVSAAKYWVFKEFACKAVHCICQMSFFSGMLLLLSISIDRYFAIVQAPSAHRHRSQRVLASKVTCLSIWILGFILSLPEAINRGVYDYESPTPRCTIVTANLLAFSTSIRISQMVFGFFIPLLVMTFCYWIIIRTLLQARSFEKNRAIKVLIAVMVVFVLFQMPYNSVMLAETITAFNNTTGQCDAIKRIDVASDVTYSLACFRCCLNPFLY

>acaCCR9

LVYWKYKGKKNLTDKYLIHLAIADLLFLFTLPFWAIAAHDGWYFNTFMCMYKINLYSCMLFLMLISFDRYTVVVRSTRARHSKQKRLTHHKLICFGVWLMAVSLCIPEIIYSQTEQSSNITICKMIYPPNVNRSIKVINLSLKIAIGFLLPLVVIVVCYTCIIHTLLRAKKTPKHKLFKIMTIIILVFLLSQVPYNSILMVKTMVLYAPVIKDCKMLDRIDIGFQLTQSIAFLHSCLNPFLY

>acaCCR10

LVRIRYHIQTIGDALLLHLAFSDLLLLLTLPMGVAAMMGRWHLGTATCQGLQGLHALNFYSGFLFLTGLTLDRYVAIRAPIAHRLRPATTCWARLGLGLIWLLSSSLALPHFLYARMEDHEGFQLCRVATVAAAISLVQVALGFVLPFVVMVVSYMAIARTLLSSPCAQSQRALWLILSLVFLFLALQLPYALLTLLDTADLMSQQVSSCKVIFHRDLALLITSGLAFARCCLNPVLH

>acaCX3CR1

LTILKAGGQRSITDIFLLNLAISDLLFVLSLPFWAFYFIHGWTLGNLLCQIVSSLYSVALFGGMFFITVISIDRYLAIVHATYAMKARTIHRGYITSAAIWTLAVLFAAPHFVFVQESEKQCTSLYPPHLQTLWPVFSYLEMNIIGFLLPVCIMSFCYLGIIKTLFSCKNTRKKRAVKLILTVVIVFLLFWAPYHVLLFLQMLRTYNYFETCVSLRVLDYIVQVTETIAFSHCCLNPIIY

>acaXCR1

RIVLKYESLMSLTNLFIVNLCISDLIFSCTLPFLIVYHSYGWIMGEFLCKAVSGIFSISYFCGVIFLTIMTILRYLAVVDPLSTLRTQKKRSGILVSLAVWVTSLLFVIPEILSIQVTTDIDGRYGCYYQAFYPWEMVELCLKVLFFLISFMIIAICYTGMLDILLRSRSQSRHRTVRLIFAIVLVFFLSWAPYNVLGFVYALSEQNVIESKCQTKKDIYFAFDISRTVAYCHCCLNPVLY

>acaCXCR7

WMNFQAKTTGYETHLYIFNLAVADLCVLITLPVWVVSLVQQSQWHLGEITCKITHLVFSINLYGSIFFLACMSVDRYLSVAYFTTSSNAKKKRIRRCICIFVWLFAFFVSLPDTYYLKTISTNNETYCRPVYPEESAREWLAGMELSSVLLGFIIPFPVIAVFYCLLAKAISASNDQERKSNGKVIFAYVVVFLVCWLPYHVAVFLDLLLAFHFIPFSCQMENFLYTALHVTQCFSLVHCCINPILY

>acaCCRL1

AIYAYIKKLKTRTDVYIMHLAIADLLLLFTLPFWATNAVHGWVFGNPLCKITTAIYTMTFSVSMQFLAWISVDRYNAIVKSPSQQRTTKLCSKICFFVWMAGTFLCLPDLIFNQVKEFHGKIACVSTFPESLSKIIKVTIEVGEMALCFVLPFFIMLTCYSAVARALFKSPSVKKTQPLKVLAAVVSVFIVTQLPYNVIKLWRAIDIIYPLITNCKASKAMDVAFEVTNSIALFHSCLNPLLY

>xlaCXCR1

LVIYNNKLKRSSTDVYLLHLAIADLLFATTLPFWAAYKASQWVFGIFMCKAVSVLQEVNFYSGILLLACISVDRYLAIVHATEAVTQKRHWVKFICLGIWIFSLVVSLPTLLFRTVFKSPRDAYVCHDSIGNENTEDWMIILRIGRHLVGFFIPLLIMLFCYGFTIKTLYQTKSSQKHRAMKVIFAVVLAFLICWLPYNLTVIVDSLMRTRFINETCEKREHLDAALSTTEIFGYTHSCINPILY

>xtrCXCR3

VVLLQNRKRLQSTDIFLLHLALADILLVVTLPFWATQAVSGWLFGNVLCKTVASIFKINFYACTFLLVCISCDRYLAIVYAVQVYKKHRTNLVHWSCLFVWCLCVGLSVPDMVHFQVAYEPRTNVTECQPVFGSSNFKTWRVSMAFLYHIVGFLLPLCFMLYCYTHIIHTLFQTHGFKKQRALRVIITVVVAFFLCWTPYNIVALLDTLNLLHVLADNCTIDSNIDIALSVTSGLCYFHSCLNPLLY

>xtrCXCR3L

YVLKSRRCSWHLSDHYLFHLTLSDLFLGLTLPFWATQYAYGWVFGSVPCKLVGALFSINMYSSIFFLACIGLNRYFAIVHAVELHRKQRPIHTFLICAVVWATSCLLSLQEFYFRDVDFIKQLKSHSCHYKFDPETADTWRTTIRLINLSLGFLLPLFLMFFFYCRIFCTLRKSRHGHSYRSQVVIVVLLFVFVLCWGPYNTLLLIDSLQRLDVIAPSCPLFQKLDIGLTVTETLGLSHVCLNPFIY

>xtrCXCR4

VVMGYQKKSRTMTDKYRLHLSVADLLFVFTLPFWSVDAAIGWYFKEFLCKAVHVIYTVNLYSSVLILAFISLDRYLAIVHATNSQGSRKMLADKVVYAGVWLPALLLTVPDLVFARVSDENGQFVCDRIYPIDNRETWTVGFRFLHITVGLILPGLIILICYCVIISKLSHSKGHQKRKALKTTVILILAFFACWLPYYVCLTTDTFMLLGLLKADCIWENTLHKAISITEALAFFHCCLNPILY

>xlaCXCR4b

VVMGYQKKSRTMTDKYRLHLSVADLLFVFTLPFWSVDAAIGWYFKEFLCKAVHVIYTVNLYSSVLILAFISLDRYLAIVHATNSQGSRKMLADKVVYAGVWLPALLLTVPDLVFASVSNENGQFVCDRIYPIDNRETWTVGFRFLHITVGLILPGLIILVCYCVIISKLSHSKGHQKRKALKTTVILILAFFACWLPYYVCLTTDTFMMLGLVKADCIWENTLHKAISITEALAFFHCCLNPILY

>xtrCXCR5

LILIKFRRSRSTTENFLLHLALADLLMLVTFPFAITESVAGWVFGSFLCKFVGVINRINFFCSSLLLGCISVDRYIAIIHAIHTFRSRRLVAVHLPCFGVWALCFLLSMPNLFVLGIQENGNVTTCTYHQSHFPSNGWWQTGRFLNHIVGFLLPLSIMGFCYAHIVAALCRSPRLEKKKAVRLAIVVTGVFLLCWTPYNVTVFIDTLDQLGLVHNCQVREELPIAITVTEFLGYVHCCLNPILY

>xtrCXCR6

IIYAFYEKMKTLTDTFMVNLAMADILFLCTLPFLAYQAAEGWIFGNLMCKIIRGGYRINLYSSMLILTCITFDRFISITQAKKLKISHSKKHRWGKLVCVIVWTVSLILAVPQFMFSKSNDKMECFETYLEGHLHLIVNSFQMTVGFFVPLAAMIFCYTFIIKTLIFSSNFQKHKSLKIIFLVVIAFIVTQLPYNIAILCHVLYKTINAKVLVITEAIAYLHACINPILY

>xtrCCR6

ITFRYYKRTKSMTDVYLLNMAVADILFVLTLPFWTVNYHKGEWIFKDFMCKFIRSIYAINFTCSMLLLACVGIDRYVAIVQVTKSFRFRTTTMAYKRVICFSVWIMSACLSGLTYYFSKCYKYNERFVCEASYPEDATALKWKLAVIIVQISLGFCIPFFVMFFCYLCIIKTLLQAHNSQRHKAIRVIVAVVAVFLVCQVPYNVVLVIKATQLGRTDGICSKNINYAYAFFITETVAFFHCCLNPVIY

>xtrCCR7

VRYLYFNRLKTGTDYYMLNLAIADIVFLLTLPFWAVSVAKTWIFGNEMCKIIYCLYKMSFFSGMFLLMCVSMERYFAIVQAPSAHRHRSKTVLISKLSSLGIWVFAFLLSIPELLYSGVKENAKVDMCIIFSDSIQSLTAKLKISQMFFGFFLPLLIMVSCYCMIIRKLLQARNFEKYKAIKVIIAIVIVFVAFQLPYNSVMLIRAFSNSTECDTSKNLDIADDVTYSLACFRCCLNPFLY

>xtrCCR8

FVLVYCKKLRTTTDVYLFNMAVSDLLFVVSLPFIAYTIINEWIFGNIMCKILSTIYFVGFFSSIFFITVMSVDRYFAIVHVVFALRVRNVRWGLIVSIVVWVLALSISTPNFKFHEIVMTGNYTECVLSYPEINRQNWKIFCSLLINIFGLVIPLFILLFSYLHIIKTLQNSKCKQKRCAIRLILIVGIVFFIFWTPYNIVVFLNILKTSGAVDLETDQLQTAADVTHTLSLVHCCLNPIIY

>xtrCCR9

AVYVYNRKLKTMTDTFLINLAIADILFLITLPFWAIAASHDWVFKTALCKAVNSMYSVNVYSGMLLLACISIDRYIAIVQATKAQKYQTKKLLISKLTCFIVWALSTGLSLPEILFSVVKEEFNSTTCTMSYPAELSKTFKVSVLSLKVTVAFCLPFLVMVFCYAMIIPILVQARGFQRHKALKVIFAVLSVFILSQLPYNSILVLRVLNAANINDFECATTQNIDIAYKITQSVAFLHCCLNPFIY

>xtrCCR10

LTYWFCRKIKSMTDVYLISLALADLLLVLSFPFLGINAVQGWIFGNIMCKVVQGLYSVNFFSGLLFLTCISVDRYIEIVQAVQAHKCRHKSIYYSKLTCIVVWVFSLLLTLPQFIYSHSESIGGFYHCKMIFPEEVTATVKGISNVAQIIFGFIIPSLVMVFCYSVIVKTLLSSKTLRRHKTLKVIISLVVVFVMFQLPHSVVIFLETADILQSKQMPCEVSKKKDVALIVTSSLAFTRCCLNPILY

>xtrCCR12

FLLLKYEKIKTVTNLFILNLVISDLLFTITLPFWAFYHSNEWVFGNGMCKVVSSVFFIGFFSCILFLTVMTMDRYLAVVHAVSAARTRKLIYVYVASIAIWVISFVSTVPKFVLYGTRKHDSAGILCEETGFSADKIDTWRRLGYYQQLTMFFLFPLIVILYCYTLIVVKLFNTKMHNKDKAVKLISVIVLAFFICWTPYNVVIFLRLSPGDPCNDYLNNAFYICRNIAYFHCCINPFFY

>xtrXCR1

WILIKYESLVSLTNVFIFNLSIADLILSSWLPLFIVYHRQGWVFGEVACKILNAFFSIGFYSGIIFLTFMTFHRYLSVVDPLSALKAKNPLFGVAASLLSWLISICASIPVIIYKAQVDRNGFIICEYRDNLPHLVSNYQQNIVFLIAFVVIIVCYFSIIKTLRRSRSQRNHKPVKLIFIIVVVYFVSWAPYNIVMLLQSFEKQQLFKSFRDCDFSKNLDYAKSVSEKLAISHCCLNPILY

>xtrCXCR7

WLNLQAKTTGYETHLYIFNLAIADLCVLLTLPVWVVSLVQHNQWPMGEMTCKITHLVFSINLYSSIFFLTCMSVDRYLCVSLNGTAGQRRRKIIRRLVCVLVWLVAFVVSLPDTYYLKTVSSPVTNETYCRSMYPEETFKEWLLGMEIVSIMLGFVIPFPIIAIFYCLLAWTLSSSSSSSGDQERRISGRLIVSYVVVFMVCWLPYHAMVILDVMSFLQLLPFSCFLDNFLYAALHITQCYSLLHCCINPILY

>xtrCCRL1

AIYAYYKKMKSKTDVYLLNLAVADLLLLFTLPFWATDAAVGWQFGIFMCKITSAMYTINFSSGMQFLACISLDRYFAVTKAPNPQPIRKICWVTCLFVWSTSMLLSIPDLYFSTVKEHNNKHACLPVYPKDTIKQTTVLIQILEIVFCFLLPFLVMLFCYASMAKIVLQTPNIKRSRSLKVLLAVVGVFLITQLPYNVIKFWRAIDIIYALITSCSMSRTIDIMIQVTESLALFHSCLNPILY

>olaCXCR1a

VVISMNRRTMTPSDFYLLNLAVADILLALTLPFWAASVTVGWVFGDALCKIVTVLQELSFYSSILFLTCICVDRYMVIVRALEARRANRLMASWGVCAAVWALGALLSLPGLFSSSFSSKNFTLYVCAEQYDPRSADEWRLSTRILRHTLGFLLPLAIMVPCYGITIQRLLHIRGGFQRQRAMRVIVFVVGAFLLCWMPYHVTVMTDTFFRAKIVPYKCPERMAVDQAMFGTQSLGLLHSCINPVLY

>olaCXCR1bb

FVVCTMKKGRGSTDIYLMHLAIADLLFCITLPFWGTYVHFGWSYGNFLCKVLSGFQEASVYSGVFLLACISVDRFFAVVRATRVLSSNHHLVKVVCSVVWLMAGLLSLPVVIKRESMFAEELNQSICYENVTGDSSDLWQFSLRILRHTLGFFLPLVVMTFCYGRTGVTLLQIRNQQKHKAMRVIMAVVLGFVLCWLPYNVAVLTDTLIRAESLKVTSCDTRYRVEVTLNVTQVLAYMHCAINPVLY

>olaCXCR1ba

WVIGTNQRALTPSDVYLFHLTAADGLLALTLPFFAVAFVRGWIFGDFLCKFLNLIMEANFYTSIIFLACISVDRYLAIVHTRGLQQSRRGSCSRLLCAVVWAVGWALALPALFNATIAVTLKDGSETWLCTESFHVGRPTAWRLATRVLRHVFGFVLPLVVMVACYGVTVAKLLHTRGFQKHRAMRVIMAVVVAFLLCWTPYHITLMVDTLLRAELMAPGCGVRGAVNVALGVTNSLALLHSCINPFLY

>olaCXCR3aa

AALALRRRFWRVSDIFILHLAGADLLLLLTLPIRVAQVAGSSGSFGAFCKICGAVFHINFYCGVFGLLCISLDHYLCTNHAAKWRSLRRPRFAAFCCLFVWISSVLLSVPDCMFLASSKNEDQKLRCDYSYSQTATGSMLASRLFHHTVGFSLPVVFLMLLCSYFLSSLLSKDKDLQRRRRRAVIVILSLVLAFLLFWLPYNITLIRDTYLRKISHRHPKKLYPQDSMASALLITSMFGYIHACLRPPIY

>olaCXCR3ab

GVLLRRWRTWSVADIFILHLGIADILLMVTLPLWAVQYADEWKFGLLTCKMAGSLYTVNFYSGSGIFLLVCISLDRYFSLVHATQMYSRRKPWVVHVSCLVVWLFSLLLSIPDWIFLDVAQDRQGRQQCFRNYTIQDMDEWIVTSRSIFHTVGFLIPSVVLIFCYASIFHRLRSGNQNLQKQRACKVIMAVVAVFFVCWTPFNISLFVETIHRRNQNNTCQSNAALSKALKVTQSFGYIHCSLNPILY

>olaCXCR3ac

GVLLRRWRTWSVADIFILHLGIADFLLMMTLPLWTVQYADEWKFGLPICKMAGSLYTVNFYSGIFLLVCISLDRYFSVVHTQMYSRRKPWVVHVSCLVVCLFSLLLSIPDWIFLDVAQDELDRQQCFRNYGIEDMVHWIVVSRSIFHTVGFLIPSVVLIFCYASIFHRLRCGNQNLQKQRACKVIMAVVAVFFVCLTPFNITLFIDTIYSRNPNETCQSSAAPLVKAIRVTQSLVYIRCSLIPILY

>olaCXCR3ad

SILFGRKRTWSVADIFILHLGIADFLLMMTLPLWAAQYADEWKFGLLTCKMAGSLYTVNFYSGIFLLACISLDRYFSVVHATQMYSRRKPWVVHVSCLVVWLFSLLLSIPDWIFLDVVQDRLERQQCFRNYSLQDMDDWIVTSRSIFHTVVGFLIPSVVLIFCYASIFHRLRSGNQNLQKQRACKVIMAVVAVFFVCWTPFNISLFVETIHRRNQNNTCQSNAALSKALKVTQSFGYIHCSLNPILY

>olaCXCR3L

CVIRRYKNRNSGACAFSLTDTFLLHLAISDLLLAFTLPLFAVQYNHQWVFGLALCKISSALFSLNRYSGILFLACISFDRYLAIVHAVSSGWKRHTCHAQLACAVIWVICLGLSGVDIAFKQVDEQISPNKETVVLCRLWFAQNDVEWRVGLHLVSVVLGFGLPLLVMLYCYIQIFRSLCNATRRQRRKSLSLIFTLVSVFVICWAPYNCFQLVDSLEKLKVVAGGCHFNKVMDIGILITESIGVSHCALNPLLY

>olaCXCR4a

MVLGCQRRSKCSLTDRYRLHLSAADLLFVLALPFWAVDAALADWRFGAVTCVGVHVIYTVNLYGSVLILAFISLDRYLAVVRATDTNSGGLRQLLAHRLVYVGAWMPAGLLAVPDLIFARTQEGGEGSTLCQRFYPDDNAPLWVAVFHLQLVLVGLVIPGLVLSVCYCVIVTRLTRGPLGGQRQKRRAVRTTIALVLCFFICWLPYGAGISVDALLRLEVLPRSCRLEAALGVWLSVAEPMAFAHCCLNPLLY

>olaCXCR4b

VVMGYQKKVKNMTDKYRLHLSVADLLLVLTLPFWAVDAVKTWYFGGFVCVSAHVIYTVNLYSSVLILAFISLDRYLAIVRATNSQATRKLLASRVIYVGVWLPAAFLTVPDLVFARVKSVSSPSFSFRNDSVEMEDSRTICERFYPVESRVVWTVIFRFQHILVGFILPGLVILVCYCIIIAKLSKGTKGQTLKKRALKTTVILILCFFCCWLPYCIGIFLDTLMMLNVVRTTYELQQALDKWISITEALAYFHCCLNPILY

>olaCXCR5

TVLLRRRRRLRITEIYLLHLAVTDLLLLLTFPFEMIGVAVGWVFGDFLCMTKGVLENLNLLCGSFLLACIGFDRYLAIVHAIPSMQSRRPGKVHQVCAVLWFICLLLSTPNAVFLSVAKHRNSSLDCYYYRYDIHAHNWVLANRVLHHISFFFSLVVMSYCYTVLVFTLWKSPKREAKKSAVRLALLVTLVFCVCWLPYNVALLIRTMVDLDVLTYDSCRFLTLLNQTAAVTKSLGLSHCCLNPFLY

>olaCCR4Laa

WVVVCGARLRSMTDMCLLNLAIADLLLVCSLPFLAYQARDQWLFGDAMCKIVLGVYHVVFYSGIFFICLMSIDRYLAIVHAVYAMKARTLFFGRIAAAVTWTAGFLASFPELIFIKQQTTTNKTEKTDSSSHFWTIFSIFKMNIMGLFIPLCIMTFCYSRIIWKLLDSHSSRKQPIRLVLLVIAVFFCCWVPYNISSLFKGLELLQIYMGCESSNSIRLALQVTEVIAYSHSCLNPILY

>olaCCR4Lab

WVVVCGARLRSMTDMCLLNLAIADLLLVCSLPFLAYQARDQWLFGDAMCKIVLGVYNVVFYSGIFFICLMSIDRYLAIVHAVYAMKARTLFFGRIAAAVTWTAGFLASFPELIFIKQQEEGDRHHCLSVYPDSGAGEDDSSHFWRIFGIFKMNIMGLFVPLCIMVYCYSQIIWKLLDSHSSRKQTIRVVILVIVVFFCCWVPYNITSMVKGLELLQIYTGCESSKAITLALQVTEVIAYSHSCLNPILY

>olaCCR4Lac

WVLIKHIKLRMMTDVLLLNLVLSDLLLAVSLPLWIVKSHNIGLCKLVTGIYQLGFYSGTFFVTVMSVDRYLAIVHAVAAIRARALRYGIIVSVVIWIVSVIMAAPQVVFASLEKEDFDTSHCHPVYPEETVEFWKKLRNFSENTVGIFVCLPIMIFCYVKILLVLSKSRNSKKDKVVKLIFTVVCVFVACWVPYNILVFLQTLEQLEILDDCQLSNNINKAMHFTEIMALSHCCLNPIIY

>olaCCR6a

ITYALYKRAKSMTDLYLLNVAIADLLFVLSLPLIIYNEITSWSMGWLSCKLLRGSYSVNLYSGMLLLGCISIDRYLAIVQARRSFRLRSLSYSRLICALIWVFAVLVSIPTFYFYQRYEPFENSTFFLPNEEENEPDDHHYVCDFKHDDRNLAPYFKVMIPSIQLGVGFFLPLLIMIFCYTSIIVTLMKAKNFQRHKAVRVVLAVVVVFVICHLPYNISLLYHTINMFDVVKCQVADTLKVTQNALQAVAYLHCCLNPVLY

>olaCCR6b

LTYFFYKRSKTMTDVYLFNLAVADLIFVVALPLIILNEQAGFSLGVVACKLLESAYSVNLFSGTLLLACISADRYVAIVHARRSFGSRSRALTYSRLICSTIWVSALVLTLPTLIFTELFEEKDPITGTSSRKCQLSFNQPDTAKLMKVLVPGFQMAIGFLLPMLVMGFCYSWIAYTLLRAQTQRHKAIRVIVAVVVAFFVCHLPYNMALLIHTTSLFKERSCEAEQIKLYVLNVSKSVAYLHCCLNPILY

>olaCCR7

LTVFHFKRLKTMTDVYLLNLSFADLLFALSLPFWAASSMAEWVLGQMVCKSMYTIYKVSFYSSMLFLCLISIDRYFAIAKAVSSHRHRTEAAFYSKVSSTVVWVMALIFSIPEMAYTNVSNNTCSLSNQNSTELKVGIQASQIALAFLLPLLVMGFCYASIIKTLCQARNFERNKAIKVILAVVTVFLVSQVPYNIVLFLSTSVTANGGTEDCEYDNTLMLATDATQGLAFFRCCLNPFVY

>olaCCR9a

IYLNVRRRLKTMTDMYLLNLAAADLLFLATLPLWAAEASYSWTFGSAFCKLNSALYKVNLFSSMLLLTCISVDRYIVIVQSTKAQNSKMERRRLSQLVCAGVWLLALLLATPELVFAKPAPVESKYYCRMVFPSNIGNRTKILVLSLQVSMGFCLPFIAMVFCYSVIVAKLLKTRSFQKHKAMRVILSVVVVFVVSQLPYNITLVMEAMQATNMTVTNCERVKALDKAGQVLKSLAYMHACLNPFLY

>olaCCR9b

IYTTVRNRLKTMTDVYLLNLAVADLLFLCMLPFWAVDAIKGWHFGLPLCKIVSAVYKINFFSSMLLLTCISIDRYIAIVQVTKAQNLKKKRLFYSKLACIGVWLFSTLLALPEFIFAQVKTDGNLSNCALVYWDNAFNRTKILVLSMQICMGFCFPLLIMFSCYSVIIRNLLQARNFEKHKALRVIFAVVFVFILSQLPYNTTLIMEAVQAANPNITDCATVTHFDIAGQVTRSLAFTHACLNPFLY

>olaCCR10

FALYRRLRLRSTTDIFLFHLALADLLLLLTLPLKVADTNLSWSFSESFQKSARAFHAVNTYSGLLLLACISVDRYMVVVRAQEMLRLRHQMHTVGSLAAVGVWFVAVLLSLPEILFAWHYNHDCHSCNEVKMASMGLLIAVFCLTLLTMLACYSVIARVLCESPRHRRGKQWQRQRTLKLMVALVLVFLAFQLPYTVVLFWKMARPICELMLEYSTCTLAYTRCCLNPVLY

>olaCCR11aa

CVLVKHWKQSNLTDICLFNLALSDLLFVITLPFYANLSMMGYWTFGNFMCHILSGFHRTGFFSSIFFMIIMTLDRYIVILYSHKVARYRTMRLTIALTLTSWILSACVSLPSFIFTKVSNYSGKQDECYFFPENEDWYHYDLFATNMLGLILPLLVMVACYSRIIPVLVKMKTAKKHRVVKLIISIVGVFFLFWAPYNISLFLNFLLLQQIIPSTCNGDKNLRLAVSVTEAFAYTHCCLNPIIY

>olaCCR11ab

CVLVKHWKQSNLTDICLFNLALSDLLFVITLPFYSHVLVKGYWTFGNFMCSILSGFHCTGFFSSIFFMIILTLDRYIVILHSHKVAQYRTMRLTIALTLTSWILSACVSLPSFIFTKVTNDECHLLPENEDWYHYDLFAKNILGLILPLLLMVACYSRIIPVLVKMKTAKKHRVVKLIISIVGVFFLFWAPYNISLFLNFLLSHNIIPQTCDSDKNLRLAESVTEAFAYTHCCLNPIIY

>olaCCR12a

VIICKYEKLNTVTNIFLLNLVFSNLLFASSLPFWATYHFSEWIFGTAMCKIVSSTYFIGFYSSILFLTLMTFDRYLAVVHAVSAAKHRKKLYAMVSSVIVWCISVAASLKELVLRKSWKDPSDGYLCEDSGFPISTIRTWSLVTYYQQFMLFFLVPLIMVLYCYISITVRILSTRMKEKCRAIKLIFVIIVTFFICWTPYNIVILLRAIQSSNPELQPPSCSDAESLDYALYVTRNIAYLYCCISPVFY

>olaCCR12b

VIIHRFERLTTVTNILLLNLVISSLIFISSLPFIAAYLKLKKWIFGSAMCKIMGSVYYLGLYTSVLFLTLLTFDRHLAVVYPLNASHIRNRKYAFFSCAVVWIVSAVACIIPMITHNTVNSVGTTLCEQDYGNIPSAIGMKLRTTWFYLQLIMFLIFPVIVILYCYFRIAITVMSSKIVSKFKTVRLILVIVLLFFMSWAPFSILELMEDGTTNCVQKQRIEYGIVVSRNLAYFYFCISPFFY

>olaXCR1a

VILALYESLKSLTNIFILNLAISDLVFTLGLPFWAYYHVWGWQLPEILCKIVNFVFYTGFYSSILCLTAMTIYRYMVVVCTLSDRCRPKLSTGIFLSFLMWTISVGGAMPSLLHTSIIKIHHKDRESWGCEYSSEWWKHMSTYQQNLFFLFAFAVMAVCYIQILQKVRRTRSRTKSRAVKLVFCIVTVFFVGWMPYNMVIFLRALFPSLGFEDCEQSNHLDYAFFVCRLIAFSHCCLNPVFY

>olaXCR1ba

VALLLYENWKNVTTIFIMNLAVADLIFTTTLPFWAVYHLHHWVFGDFLCQCMTALYFISVYSSVLILTALSVDRLVLVIKKPTDSFRRKYVLGTCAAAWLIGIIASSTNAIKVKVTEYEGSYYCEPSHYVDLGYYAEISLLFLLPFIITVFCYTGIIVAVLRTTVRRKFRSVSVMFCIVTVFFFCWGPYNIALIIGHVYEPTECWKKERKEVILGVCQILAFSHCCMNPPLY

>olaXCR1bb

VALLLYENWKNVTTIFIMNLAVADLIFTTTLPFWAVYHLHHWVFGDFLCQCMTALYFISVYSSVLILTALSVDRLVLVIKKPTDSFRRKYVLGTCAAAWLIGIIASSTNAIKVKVTEYEGSYYCEPSHYVDLGYYAEISLLFLLPFIITVFCYTGIIVAVLRTTVRRKFRSVSVMFCIVTVFFFCWGPYNIALIVGHVYEPTECWKQERQEVILGVCQILAFSHCCMNPLLY

>olaXCR1bc

VILVNYENIRSVTNTLILNLAVSDLLFTASLPFWIYYHLHGWTFGKPTCKLVNWVFFTGFCSSSILLVLMTVHRYVAVMNPLSNIVSAAGFPSVVVTVIVWVVSILIASPSFVYSNVTDHENFKICFYENQDAKLWIFYQQNAFFVVCSLVFIFCYSKILWKLKGSTVQKRNNRTLKLIFILVVVFFLEWAPYNYVIFQMSLDVINKNSGHECDSRTRLDYAFFISRMLAFSHCCLNPVFY

>olaCXCR7a

WVNIRAQSDSTPRHETHTYIAHLAVADLCVCATLPIWVSSLAQHGHWPFGEVACKLTHLLFSVNLFGSIFFLACMSVDRYFSLLQPREEGAQRRKLIRRGVCLGVWLLALVASLPDTHFLHTVKSTHSDTMLCRPVYPEENHTEWMVGVQLSFILLGFLLPFPVIAVFYALLARAFAHSSSSSSSSPVEQERRVSRRVILAYIVVFLACWGPYHSVLLADSLSQLGLVPLTCGLENVLYVALHLTQCLSLLHCCFNPILY

>olaCCRL1a

GVYAYRKRLKTMTDSFLAHMAAADLMLIFTLPFWAAGAARGWELGAVLCKMASTSYTVNFHCCMLLLACVSLDRYLALARLQGGQQRRGLQGAFSRKHCWKVCLAVWTTAFLLGLPDLIFSEVRQASSRNACLLIYPAWMAPGGTAVLEGAEVLLGFLLPLLVMLVCYWNVCKVLTGLPAERKGKKWRAVRVLLLVAGVFLVTQLPYNLVKAFRAVDSVYLLVTHCGTSKALDQAAQVTESLALTHCCFNPILY

>olaCCRL1b

VVYTSRLRLRTLTDVCILNLAIADLLLLFTLPFWAADAVHGWTLGSAACKLMSFLYSTNFSCGMLLLACISVDRYRAVTHSSTGRAETGQRARRQWLLVCVALWALASFLGLPELVFSGVKTSHHRTSCTAFYPRNMARPAKAALELLEVILRFLLPFAVMIVCYTLMGRALTRVPGVRRERKWRALRVLFAVVAVFLLTQLPYNVVKLCRALDIIYVLVTVCDVSKGLDHALQVTESLALTHACINPLLY

>gacCXCR1a

LVIGLSRHPLPPSDLYLLHLAVADLLLAVTLPFWATSLTRGWVFGDAACKLVTVLQELSFYSSILFLTCISMDRYMVIVRAMEARRANRRRVSWALCAAVWAVGALLATPGLLHSAQPSKTSNLTTCGEKYEQGNADRWRLATRMLRHTLGFLVPLAVMLPCYGVTVRRLLRVRGGFQRQRAMRVIVTVVVAFLLCWTPYHVAVMADTFFRAKIVPYKCPARMAVDLAMLGTQSLGLLHSCVNPVLY

>gacCXCR1ba

WVIGTSKQALTPSDVYLFHLTMSDFLMALTIPFSAVHLIQGWVFGDFLCIFLSLVFEANFYTSILFLTCISVDRYLMIVRASESHRTRQAMRRRLLCATVWALGWALALPALFNDVSKLTAERMTCGESFDLGSASSWRIATRGFRHIFGFFLPLVVMVICYSITITRLLRTRGFHKHRAMKVIIAVVMVFLLCWTPYHVTMMVDTLLRADLIPPGCALRRSLSTALFSTHGLALFHSCVNPVLY

>gacCXCR1bb

YVIYSMEKGRGTTDIYLMHLAMADLLFCVTLPFWAINAQSGWIFGNFLCKLLSGFQEASVYGGVFLLACISVDRHFVIVRATRLRPFHRLLVKVTCGVVWLVAAVLSLPVAILKESMHDEDLGRTICYENITNESSDRWRVIMLVMRHSIGFFVPLVVMTVCYGWIVVALIHTRNSQKHKAIRVILAVVFAFVVCWLPYNIAVLIDTLTRKKTIAMETCETRYMVEVMLAVTQVLAFMHCAVNPVLY

>gacCXCR3aa

ALLAKGRRPWSTSDAFVFHLSVADVLLLATLPLWAAQAALRGGWGFEGLLCKISGAAFNVNFYFGIFLLACIGLDLYLSIVRGTRLFARERPRLGHISCLSIWLASLLLTVPGWAFPAAKKDPARATGLCARGHSWPLTDWQLASRVLHHALGFLLPAAALFFCCSRVLLRRRVASGLQERRPVAVFLAPVAAFCLCWVPYNVALVADTARGRAAEPAERDGSLKTALTVTSALGCVHACLRPLLY

>gacCXCR3ab

GVLVQSRRSWSVTDTFIVHLGVSDVLLLATLPVWAAQSAQADGWTFGSPLCKITGAVFTINFYCGIFLLACISLDRYLSIVHATQMYSRRKPRVVQVSCLAVWLFSLLLSIPDWIFLEAVEDSGRGQKTQCVRNYLMNASGKVGDWRLASRLLFHTAGFLLPSVVLIFCYSCILRQLRCGSRAPQKQKAFRVIVTVVVVFFLCWTPYNITLMVDTLHSADHNDTCGTRTSLGTAKMITSSVGYLHCSLNPVLY

>gacCXCR3L

CVIRRYRNSQSGGACAFSLTDTFLLHLAISDLLLAFTLPLFATQWAHQWVFGLAACKLSGALFSLNRYSGILFLACISFDRYLAIVHAVKRNAWHAQVACALIWACCLALSGADVAFKQVEEVKTGANRTALLCVVWYTEHPTRWRVGLQLVGMLLGFGLPLLVMLYCYIRIFKSLCNATRRQKRKSLRLIVSLVSVFVVCWAPYNGFQLADSLHRLGAVTGGCRFGHVMDVGTVVAEGLGLSHCALNPLLY

>gacCXCR4a

FVLGCQRRSKCSLTDRYRLHLSAADLLFVLALPFWAVDSALGDWRFGEVTCVGVHVIYTVNLYGSVLILAFISLDRYLAVVRATDTNTGGLRQLLAHRLVYVGAWLPAGLLAVPDLIFARTQEGGEGATLCQRFYPAESAPLWVAVFNLQLVVVGLVIPGLVLLVCYCVIVTRLTRGPLGGQRQKRRAVRTTIALVLCFFVCWLPYGVGISVDTLLRLEVLPRGCRLEAVLGVWLAVAEPMAFAHCCLNPLLY

>gacCXCR4b

VVMGYQKKVKTMTDKYRLHLSVADLLFVLTLPFWAVDAAKSWYFGSFLCVSVHVIYTLNLYSSVLILAFISLDRYLAIVRATNSQDTRKLLATKVIYVGVWLPAVLLTVPDLVFARVQEAYPLNSPFPNQSMEAVDSRIMCQRTYPQKHSVEWTVGFRFQQILVGFILPGLVILNCYCIIIGKLSQGARSHAQKRKALKTTIILIVCFFSCWLPYCVGIFLDNLVMLGVISLSCDLEQAVEKWISISEALAYFHCCLNPILY

>gacCXCR5

TVLLRRRHHLRITEIYLLHLALADLLLLFTFPFDVVNAAAGWLFGEFLCKLTGLVQNLNLHCGSFLLACIGFDRYLAVVHAIPRMQSRCPKTVHRTCIALWLVCLGLSVPNAVFLSVKEVGGQVSCFYHDYGIHANNWVLTNTVLNHVCFFIPLAVMSYCYAAVVVTLCRSQKSQAKQGAIRLSLLVTLVFFFCWLPYNVTILIKTMVDLSVISQQACEHYILLLSVLDVTKSIGLSHCCLNPFLY

>gacCCR4Laa

WVITCGVRLRSMTDVCLLNLAGADLLLVCTLPFLAHQAWDQWVFGDAMCKVVLGIYHIVFYCGIFFISLMSIDRYLAIVHAVYAIRARTRSFGMIAAAVTWVAGFLASFPDLIYLKQQPGPNMSQSCFPVYPEDVGHSWMIFSVFKRNVLCLFVPVVIISFCYSQIVWRLLHSQSSKKRAIRLVLTVVAVFFCCWVPYNVASFFNALELLHIYTECESSKAIRLALQVTEVIGYSHSCLNPILY

>gacCCR4Lac

WVLLRYIKLRTMTDVCLLNLAVSDLMQATTLPLWTCNDTNLASCKLMTGGYQLGFYSGTLFVTLMSVDRYLAIVHAVAAMRARTLRYGIIASTAIWVISVTMALPGVTFASLEIDVNDNSSQCQPLYPDDSHRFWKLLRNFSENTVGLFLCLPIMIFCYVKILIVLSKSRNAKKDKAVKLIFTVVGVFVVCWVPYNVTVFLQTLQLFLENLDTCEASKSINSAMHFAEIIALSHCCVNPVIY

>gacCCR6a

VTYAFYKRTKSMTDVYLLNVAVADFLFVASLPLIVYNELSSWSMGPVACKLLRGSYSMNLYGGMLLLACVGADRYVAIVQARRSFRLRSLRYSRLICGTVWSAALLLSLPTFYFYHRYEPLHIGASVEANRTAEPPHYVCELWFADNFTARTTKVAVPGTQLAVGFFLPLLVMVLCYGAIIVKLTKARNFQRHKAVRVVLVVVLVFVVCHLPYNVTLLYETAGMFQEGSCEGSDVLQAAKTLTQTLAYLHCCLNPVLY

>gacCCR6b

ITYIRYKKSKTMTDVYLYNVAVADLIFVVALPFLIYNEQHGWLMGSVACKMLRSAYSINLYSGMLLLACVSGDRYVAIVQARKSFGARPRTLLYSRLICSAVWAFAVALTVPTLLYSERSEDPQATCQLSFKETGTAKLMKVAVPSLQMAIGFLLPLLVMMFCYTSIVCTLLRARSSQRHKAIRVVMAVVAVFVVCHLPYNATLLNHTLSLFKVRTCASEKIRLRVLAVSKSVAYFHCCLNPVLY

>gacCCR7

LTFFYFKRLKTMTDVYLLNLSFADLLFALSLPFWAYNSTTKWRLGLTMCKAMYTIYKVSLYGSMFVLAFISVERYFVIAKAISAHRYRSRALFLSKVSSVGIWLMALIFSIPEMTYTAIINNTCTPYSSYSDMLHVKIEASQIALAFALPLLVMSICYSRIVQTLFQARGFERNKAIKVILAVAAVFLVCQMPYNLLLLLNTVVIAQGRSEDCYYQNTFLYVSDVAQCVAFLRCCLNPFVY

>gacCCR9a

IYLNLRRRLKTMTDVYLLNLAVADLLFLVTLPLWAAEASHGSWSFGSALCKLNSALYKVNLFSGTLLLACISVDRYVVIVQTVRAQNSQAERRSCSRLVCSGVWLLALLLATPELVFAATTEPSGREYCRMVFPAHLGNRTKILVLSLQVSMGFCLPFVVMAFCYSVIAAKLLRTRSFQKHKAMRVILAVAAAFASTQLPYAGVLVMEAAQATTVTLTECEDLKQFDKAGQMLKSLAYLHACLNPFLY

>gacCCR9b

IYTTVRHRLKTMTDVYLLNLAVADLLFLCMLPFLAVDSIKGWNFGISLCKIVSAVYKINFFSGMLLLTCISVDRYIAIVQVTKAQNLKKKRLFYSKLACLGVWTFSALLALPEFIFAQVTDRNGQVLCTLVYWNNAFNRTKILVLSLQICVGFFLPLLVMLFCYSVIIRTLLQAKSFEKHKALRVVFVVVFVFVLSQLPHNALLVVEATQAAKTTITKCDVIAAVDIAGQIAKSLAFTHACLNPFLY

>gacCCR10

FALYRRLRLRSVTDVFLFHLALADLLLLLTLPLQAADTLGWSPSMALDFVVRTCYAINTYSGLLLLACISVDRYLLVAWAQEMLRLRRRMLTGGRAAAAGVWLVAALLSLPQVLYSGLAGRDSEAYCGLVKCEQVKVATNGAVIAVFCLSLAVMATCYSRIACVLWDGRASRRGKQWHRQRTLKLMVALVLVFLAFQLPYTVLLSRKMAGPFCGLLLEYVTCTLAYARCGLNPVLY

>gacCCR11aa

CVLVKHRHKTNLTDMCLFNLAVSDLVFVFTLPFYSHYSVVGEWPFGDFLCRFASGSHTTGFLSSIFFMVVMTLDRYMVIMHAHKVAQYRTFRAGIALTAFVWTMSLCFSLPAVFFTKVTNESTGVACSYEPENDAWRLYDIIMKNVLGLGIPLLVMIVCYSRIIPILLNMRSTKKHRVVKLIISIVIAFFMFWAPYNISILLEFLNLSTDCNSYQSLKLSIIVTETIAYSHCCLNPIIY

>gacCCR11ab

TFYSLHRHKTNLTDMCLFNLAVSDLVFVFTLPFYSHYSVVGEWPFGDFLCRFASGSHTTGFLSSIFFMVAMTLDRYMVIMHAHKVAQYRTFRAGIALTAFVWTMSLCFSLPAVFFTKVTNESTGVACTYEPENDAWRLYDILMKNVLGLGIPLLVMIVCYSRIIPILLNMRSTKKHRVVKLIISIVIAFFMFWAPYNISILLEFQKLSTDCNSLQSLKLSIIVTETIAYSHCCLNPIIY

>gacCCR11ac

CVLVKHRHKTNLTDMCLFNLAVSDLVFVFTLPFYSHYSVVGEWPFGDFLCRFASGSHTTGFLSSIFFMVVMTLDRYMVIMHAHKVAQYRTFRAGIALTAFVWTMSLCFSLPAVFFTKVTNESTGVACSYEPENDAWRLHDILMKNVLGLGIPLLVMIVCYSRIIPILLNMRSTKKHRVVKLIISIVIAFFMFWAPYNISILLEFLNLSTNCNSHQSLKLSIIVTETIAYSHCCLNPIIY

>gacCCR11ad

CVLVKHRHKTNLTDMCLFNLAVSDLVFVFTLPFYSHYSVVGEWPFGDFLCRFASGSHTTGFLSSIFFMVVMTLDRYMVIMHAHKVAQYRTFRAGIALTAFVWTMSLCFSLPGLFFTKVTNESTGVACSYKPENDAWRLYDILMKNVLGLGIPLLVMIVCYSRIIPILLNMRSTKKHRVVKLIISIVIAFFMFWAPYNISILLEFLKLSTDCNSHQSLKLSIIVTETIAYSHCCLNPIIY

>gacCCR12a

LIIYKYEKLCTVTNIFLLNLVLSNLLFAGSLPFWASYHLSEWIFGLALCKLVSSAYFIGFYSSVLFLTLMTFDRYLAVVHAVAAAKSRKKAYAIIASVVVWCISIVASLKELVLQNVSKSPFNGLICEESGYPKITMERWRLVSYYQQFLIFFLLPLCMVMYCYISITVRILSTRMKEKCRAIKLIFVIIFTFFACWTPYNIVILLRAIQITHPSEGDDPCADEDRLDYAMYVTRNIAYLYCCVSPMFY

>gacCCR12ba

LITIWFEKLTTVTNILLVNLVMSSLIFMSSLPFMGLSNWIFGNVMCKIHGTVYYLGSYSSVLFLTLLTFDRHLAVVHSLTASRLRSQRYAAVSCVVVWLVSCLACIKPMILHKAFVDFENTTYCQEYPNEIPGIEGKLLSDFGFYIQLILFLIVPLAVTIYCHVRIAITVVSSKIVTKFKTVRLIFVIVLLFFTSWTPFNILMLMNDEDADCETRQKMDYALEVTRVMAYAYFCISPVFY

>gacCCR12bb

LILVQTFEKLTTVTNILLVNLVMSSLIFRSSLPFMGVYLQLSNWIFGNVMCKIHGTVYYLGSYSSVLFLTLLTFDRHLAVVHSLTASRLRSQRYAAVSCAVVWLVSCLACIKPMILHKAFVDFENTTYCQEYPNEIPGIEGKLLSDFGFYIQLILFLIVPLAVTIYCHVRISITVVSSKIVTKFKTLRLIFVIVLLFFTSWTPFNILMLMNDEDADCETRQKMDYALEVTRVMAYAYFCISPVFY

>gacCCR12bc

LIIHRFEKLTTVTNILLVNLVMSSLIFMSSLPFMGVYLQLSNWIFGNVMCKIHGTVYYLGSYSSVLFLTLLTFDRHLAVVHSLTASRLRSQRYAAVSCVLVCLVSCLCMLQAHVFYTKALVDFENTTYCQEYLNEIPGIEGKLLSDFGFYIQLILFLIVPLAVTIYCYVRIAITVVSSKIVTKFKTVRLIFVIVLLFFTCWILFNILMLMNDEDADCETRQKMDYALEVTRVMAYAYFCISPVFY

>gacXCR1a

VILALYENLKSLTNIFILNLAISDLVFTAGLPFWSIYHIWGWLFSKVLCKIVTFIFFTGFYSSVLFLTIMTIYRYLVVVHPQSILIPQRPSTSIYISVVMWIISVGAALPSLLYTTIVSIPHKDNHSLGCEYQDRLWKTISVFQQNIFFLVAFAVMAFCYIQLLWKITRTRSHTRRRAVKLVFSIVAVFFLGWVPYNVVIFLQVLADVGASSDNCGASIDLDYTFHVCRLVAFSHCCLNPVFY

>gacXCR1bc

VVLAKYENLKALTNAFILNLAVSDLFFTAALPFWAYNHVHEWTLGEHACKMATFVFYVGFYSSGIFLILMTAHRYVAVMSPLSNVVSTTGSVSVAACVITWAVSTLGAGSSFVATKVDQERCVLASSYWKLWVIYQQNVLFLLSSVVFIFCYSQIMCRLLRPTAQRRKNKTLKLIFALTVVFFVGWAPYNAVIFLQSLNMRPQQEVDSSVLVEMCEASRRLDYAFYVSRLLAFSHCCLNPVFY

>gacCCRL1a

AVYAYHKRLRTMTDAFLTHLAVADLLLLFTLPFWAADAARGWELGGVLCKVVSACYSVNFTCCMLLLACISLDRYLAVARVRGREGGRWLRRVVTRRHCWKLCSAVWATAFVLGLPDLILSEVRWLSDRSVCLAVYPASMAQGGQAALEVAEVLLGFLLPLLVMVICYGSVGRALRGLPADVGGRKRRALRVLLIVVGVFVVTQLPYNVVKLYRAMDSVYALVTHCVTSKVLDKAAQVTESLALTHCCLNPILY

>gacCCRL1b

VVVYASKLRLRTLTDMCILNLAISDLLLLFTLPFWAADAVHGWKLGVAACKLNSFLYGTNFSCGMLLLACVSVDRYRAVAQSPAGRPGSAPRVRTQWTLVCVLLWAVAGVLGLPELFFCTVKHSHHRMACTAVYPPGMGRSAKATLELMEVALRFLLPFLVMTVCYCSVWRILSRAAGVRRERKWRALRVLLAVVAVFLLTQLPYTLVKLIRAMDVIYILVTDCEVSKGLDRALQVTESLALVHACINPLLY

>dreCXCR1ba

WVIGSNRRLLSASDVYLFNLMLADTLLALILPFSAVNVIHGWVFGNVACKLVSLVKEVNFYTSILFLVCISVDRYMVIVRAMESQKAQRRLCSGVACGLVWVLGLVLSLPSFYNEAFFDKRMFNQTICAERFETDHADEWRLATRIMRHVLGFALPLVVMLSCYSVTVVRLLRTRCFQKQRAMKVIVAVVVAFLVCWTPFHVSTIIDTILRAKVVQFGCTMRTSVEVAMFATQNLGLLHCCVNPVLY

>dreCXCR1bb

FVVFFMDNRRTSTDLYLMHLAVADLLFSLTLPFWVAYLHAGHWPFGTIMCKMISGVQEVTFYCSVFMLACISIDRYMAIVKATQFLNRKLHLIGFVCALVWLCAALLSLPVMVHREAITYDGVEYICEDNVTAESTDSWRMSLRIIRHTLGFFLPLTVMMFCYGFTMFTLCHTRNSQKQKAMRVILSVVLAFIICWLPHNIIEFTDILMRAGQVEETCQLRDNIDVALYATQVMAFAHCAINPILY

>dreCXCR3aa

AVLWHKWLNCSVMDIFIFHLSLIDSLLLLSMPLWAVDAVKGWIMGSGLCKLAGVLFKMNFYCSMLMLAFISVDCYLSIVHGVQKLSRKKPMVVHGCCLIIWLVCLLLSIPEWIFLKSISDSTDQVKDECIYFYPDDSWHRSSRFPHHVIFGVGTLVLLFCCTSIMLKLQRESMCQQKKMGRKTAIIAVLVLVFLICWTPYSIAFIVNTGARPVHIDPLTGESECEWRQWTATKITAIFGLLHCTINPVIY

>dreCXCR3ab

IVLWKKRAGLNVTDIFILHLSLADILLLLTLPFWAVEAVKEWIFGTPLCKLTGAMFRINFYCGIYMLSCISLDRYLSIVHAVQMYSRKKPMAVHCCCMIVWFFCFLLSIPDWILLGANKDSRRQDRTECVNSEALSDFWVLVNRLIYHFLGFIIPAIMMVFCYTSILLRLLLGSKCMQKKRAIHVIVALVLAFFISWTPYNIALMADTIHTNRTDNNQTSCETRTTLDVAITATSTFAYMHCCVNPILY

>dreCXCR3L

CVIRRYRQSRHSPCSFSLTDTFLLHLAVSDLLLAATLPFFAVEWISEWVFGKVMCKITGALFSLNVYCGVLFLACISFDRYLAIVHAINISWRRKTCHAQLACAFIWVICLGLSMVDMHFRDLVEIPGMNRMVCQIVYSEQYSKQWQIGMQLVSMVLGFILPLLVMLYCYLHIFKALCHATRRQKRRSLRLIISLVIVFVISWAPYNALRMTDSLQMLGVIVKSCALNNVLDVGILVTESLGLAHCALNPLLY

>dreCXCR4a

LVMGCQKKSRTMTDKYRLHLSVADLLFVLTLPFWAVDAAKDWYFGGFMCVAVHMIYTVNLYSSVLILAFISLDRYLAVVRATNSQGPRKLLANRIIYVGVWLPAALLTVPDLVFAKAESSAIRTFCERIYPQDSFVTWVVAFRFQHILVGFVLPGLVILICYCIIISKLSRGSKGTQKRKALKTTVVLIVCFFVCWLPYCGGILLDTLMMLEVIPHSCELEQGLQKWIFVTEALAYFHCCLNPILY

>dreCXCR4b

LVMGFQKKSKNMTDKYRLHLSIADLLFVLTLPFWAVDAVSGWHFGGFLCVTVNMIYTLNLYSSVLILAFISLDRYLAVVRATNSQNLRKLLAGRVIYIGVWLPATFFTIPDLVFAKIHNSSMGTICELTYPQEANVIWKAVFRFQHIIIGFLLPGLIILTCYCIIISKLSKNSKGQTLKRKALKTTVILILCFFICWLPYCAGILVDALTMLNVISHSCFLEQGLEKWIFFTEALAYFHCCLNPILY

>dreCXCR5

IVLLKRRHNLRITEIYLLHLAVADLLLLFTFPFAVTQSIAGWLFGNFLCKLVGLANRLNLVCGCLLLACISFDRYLAIVHAIPSLQTRRPRTVHLTCGLLWLLCLLVTIPNLVFLSVEKDNNSTRLSCYYNSHGIHGSNWKLTSHFITHLCFFLPLVIMGYCYTFVVITLRQSHRSLEKQGAIRLALLVTVVFCLCWLPYNLTILMNTLVDLEAMPKLSCPAQDTLDRAVIVTESIGFSHCCLNPILY

>dreCCR4Laa

VIRLVLKSLRSMTDICLLNLAIADLLLVSSLPFLAHYARDQWIFGGPMCTIVLSVYHIGFYSGIFFIVLMSVDQYLAVVHAVFALKVRTRTYGFLASLVIWVAAVAASFPELIYIDTTDINNQTLCTSYPTTDQSSYHDSKTNGIFKMNIIGLIIPLSVIGFCYSMILIKLLNVRSSRKQAIRLVVVVMVVFFCCWVPYNIAAFFKALELKRVIPHSCESSKAITLSLQITEAVAYSHSCINPFLY

>dreCCR4Lab

WVVLMGVKLRSMTDICLLNLAIADLLLVSSLPFLAHYARDQWIFGDHMCTMVLSVYHIGFYSGIFFIVMMSVDRYLAVVHAVFALKVRTKTYGILASLVIWVAAVTASFPELIHLKTTVTNNQTLCASYPTTDQWSYHDSKTAGIFKMNVIGLILPLSVIGFCYSMILIKLLTVRSSRRQAMRLVVVVMVVFFCCWAPYNIAAFFKALELKKVLTHSCESSKAITLSLQITEAVAYSHSCVNPFLY

>dreCCR4Lac

WVLLKSMHVKNMTNLCLLNLAMSDLLMVLSLPFWALYAQGHYLKTDAMCKAMAGAYQVGFYSGIFFVTLMSVDRYLVIVHAVAVLGAKMLRYGIVASVIIWMVSIGAALPEVIFAEVVKDSESNSCQRHYPDESARKWKLFRNFGENAVGLFISLPIIAYCYLRVLMVVKKTKNSKKNRAIKLILGIVIMFVVFWVPYNVVVFLKTLHEFDMLTSCEPYKIINMAMDVTETIALTHCCVNPFIY

>dreCCR6a

VTYALYKRTKSMTDVYLLNVAIADILFVVALPLIIYSEQHSWAMGNMSCKLLRGIYSVNLYSGMLLLACISGDRYLAIVQARRSFRLRSSTLLYSHLVCAAVWLLALLLSLPTFIFYERYENGLTESTFLFNNNTIMEEIQYVCSFKFESNETARMMKTIVPSSQVAVGFFLPLLIMGFCYSSVIVTLLRAKNFQRHKAVRVVLTVVLVFVVCHMPYNLVLLYHTINLFEQQECSHEEAVALTMTITESLAYLHSCLNPLLY

>dreCCR6b

LTYAFYKKAKSMTDVYLVNVALADLLFVVALPLIIHNERSRWSMGTWACKLLRGAYSMNVYTSTLLLACISGDRYIAIVQARRSLRTRSQAKAYSRIICLAIWFLAFVLSLPTVIYHQEKNQECITIFSEMETARLVKILIPSMQMVFGFLVPLMVMVFCYSWTMVTLLKAQNFQKHKAVRVVLAVFFVFVLCHLPYNVALLVYTSKLFVERSCGEEQVTLMTLSVSRTAAYLHCCLNPILY

>dreCCR7

LTYLYFKRLKTMTDVYLFNLAMADLLFAISLPFWAASFMSTWHLGLYPCKAMFTIYKVSFFSGMFLLTCISIDRYFSITKAVSAHRCRSSAVYYGQVSSLVTWVIAIVFSVPDMVFAEINSRGTCSANVNYQDYYVKMVISQMVLGFIIPAVVMGFCYICIIKTLMQAKNFERNKAFKVIIAVVVVFVFSQLPYNVVMGVSVQQTTDCAKDNTRLFALDVTMAVAFMRCCVNPFLY

>dreCCR9a

IYTHFKNRLKAMTDVYLLNLALADLFFLCTLPFWAADSIYGWAFGSGLCKVVSAVYKINFFSSMFLLTCISVDRYIVIVQTTKAQNSKRSRLLYSKLICVLVWLLAALMSIPECLFARSKEDDESNTFCTMVYWNNENNRTKILVLALQICMGFCIPLVVMIFCYTNIIRTLLKTRNFQKHKALRVILAVVAVFVLSQLPYNGMLVFEATQAANMTVTNCTESIRFDIAGQVMKSIAYMHSCLNPFLY

>dreCCR9b

IFTTVRNRLKTMTDVYLLNLAVADLIFLGTLPFWATNAAQGWVFHQVICKGVSAAYKINFFASMLLLTCISVDRYIAIVHVTEAHNYKNKRMLHSKITCAFVWLASCILALPEFIFAKVKNIEPQHNSCVMVYSIMDNNRTKVLVLALQICVGFLVPFMVIVLCYSVIIRKLMQARSFEKHKALRVIIAVVAVFVFSQLPLNGYLIIEAGQANNATITDCEVMQRLDMVGQIVKSLAYTHCCLNPILY

>dreCCR10

FALYRRLRLRCMTDVFLFFLALSDMLLLLTLPLQTVETLIGSWEFGEPMCKLNRGMYAINTYSGLLLLACISIDRYLVVVCTRSMRKRSSGTLFYSVLSALSIAVISIMFSLPDLSFSSVDNVLNSNLSSCDMKVSEVKWKLWAQIAKIAGFCIPCITMIVCYGAIGHVLIHTGGKGWRRQRTLWLMALLVVLFLLFQLPYTVVLLIKISTPTPTLCSDWTTLHIMEIVTRNLAYVRCCLNPLLY

>dreCCR11aa

WVFIRCRQKSNMTDVCLLNLALSDLLFLVSLPFWAHNAMNQRTFGKFMCHTITGLFMIGLYASIFFMVLLTLDRYAIIIHPNCMFFRNRSAKLGLALLVWMLSLLASLPNIIFANEKFDLNHIKSCQPDFPDNTSWMSFTYINMNLLSLIFPLIILIFCYSRIISTLFRMKSEKKPKLVKLILAVVTVYFLFFTPYNIVIFLLFLQRMEYFFSCEWHIDLSLAMQWVETIALSHCCLNPIIY

>dreCCR11ab

WVLIRHRQKSNMTDVCLFNLALADLIFLVSLPFWAHNAMDEWILGRFMCHTITGLFMIGLYASIFFMVLMTLDRYAIIVHAHSVFSRNRSTKMGLALASLVWMLSLFASLPNIIFANANNGTNSKSSCRPDFPDNTSWMSFTYINMNLLSLIFPLIIMSFCYSRIIPTLLSIKSQKRHKVVRLILAVVAVYFLFWTPYNIVMFLMFLQRMEYMFSCEWHNGLSLAMQWVETIALSHCCLNPIIY

>dreCCR11ac

WVLVRYRHKSNMTDVCLFNLALADLLFLVSLPFWAHNAMDEWIFGRFMCHTITGLFMIGLYASIFFMVLMTLDRYAIIVHAHSVFSRNRSTKMGLALASLVWMLSLLVSLPNIIFAKDKNETNSKISCGSDFPKDSSWMPFTYLKMNLLSLVFPLIIMIFCYSRIIPTLLSMKSQKKHKVVRLILAVVAVYFIFWTPYNIVMFLMFLQKMEYMLTCEWHNGLSLAMQWVETIALSHCCLNPIIY

>dreCCR12a

CIIYKFEKLSTVTNIFLLNLVISDLIFASSLPFWAVYHKSEWIFGKNLCKFVGSCYSVGFNSSILFLTLMTFDRYLAVVHSIAAAQSRRMAYAFGSSAAVWVVSIVASIKDIVLYDVMKTEDGLLCEMTGYNQTFLTKWELIGYYQQFFLFFMVPLIIVLYCYVRITIRIMYTRLMEKCRAVKLIFIIVFTFFICWTPYNVVILLKAIKTYFKVQNDCSNALDYALYVTRNFAYLYCCISPVFY

>dreCCR12ba

LVVHKFEKMNTITNIFLINLVASNIIFTLTLPFYAVYHSSEWIFGEPMCKLVTSAYYLGFYSSILFLTLMTFDRYLAVVHCIMANSQRRSIYAASLSVAVWIISLLASLEYLIYFTVEESQVGGLSCNDPRVGEWKTFALYKQFVLFFLFPLVVFVYCYSRITLTVMHTRMVKKHHTIRVIFVTVLMFFVFWSPYNIVLVMREYKDPNDCNSNLPHAVYVTNNVARLYFCINPVFY

>dreCCR12bb

LVVYKFEKMNTVTNIFLINLVASNIIFTLTLPFQAVYHSSEWIFGEPLCKLVTSAYYLGFYSSILFLTLMTFDRYLAVVHCVVANKNRRSVYAASLSVAVWIISLLESLEYLIRFTVEESQMDGLLCEDSNENQWRTFSFYKQFVLFFLFPLVVFVYCYSRITLTIMRTRMIGKHRTVRLIFVIVLMFFLFWSPYNIILMITEHKDLEHEDCNSVLPYAVYITNNIARLYFCINPVFY

>dreXCR1aa

IILALYESLKFLTNVFILNLALSDLLFTFGLPFWASYFIHGWTFGEIGCKAVKFLFYVGFYSSVLFLTLMTIQRYMAVVHPLSDWEKCRCFSVAPIIIWMMSGTAALVGAHYSKILKDLNNTYCEYESIKVKSGIAYFQNAFFFAAFLIMGFCYCRMLQTITNARTSKRHKTVRLIFSIALVFFIGWAPYNIAMFLRSLTDQNIPPFTICEVSKGVDYAYYLCRLLAFSHCCLNPVFY

>dreXCR1ab

IILALYENLKSLTNVFIIHLSVSNLLFTFGLPFWASYFIHGWTFGEIGCKAVKFLFYVGFYSSVMFLTLMTIQRYMAVVHPLSDWEKCRCFSVAPIIIWMMSGTAALVGAHYSKILKDLNNTYCEYESIKVKSGIAYYQNAFFFIAFIFVWFCYGRMCWAITKYHNRETHRTVRLTFLIALLLFFGWTPYNIAMFLRSLTDQNIYPFTLCEVSKTVDYAYYICRLIAFSHCSVNPALF

>dreXCR1ac

VILVLYEKLKSLINLLILNLALSDLLFTFGLPFWASYFIHGWTFGEIGCKAVKFLFYVGFYSSVLFLTLMTIQRYMAVVHPLSDWKKHRCFSVAPFIIWILSGTAALSVSLRSKVLIHDDNLYCEFDSIKVKHVAVYSQNIFFLMAFCIMGFCHARMFQVITQSQSRRRRKTINLIFCIGIAFFIGWVPYNIVMLLKTLQDYALPPFLICSVSIHLDYAFYACRLLAFSHCCLNPVLY

>dreXCR1ba

GALTCYEDLKRATNLFMFCLALFDLVFTLTLPFWSTEFLHHWVFGDVACKIMTGAYFVGIYGSLILLTAMTLDRFVVVVVRSYWLTRSRRLKCAKGACIGAWIISLIACLRDSVSAKVQNIHIENYSCQSISTTDETFGYYAQLILLFFVPFAVIVFCYAKILMTFMSTSTKQKSRTVILVLCIVVAFFICWGPYHIIVVLMSIYDFDACKHYELHIAFIVCRILAFSHCCINPALY

>dreXCR1bc

CVLVKYENLRSLTNTFLLNLAISDLIFTVGLPFWAYYYVNGWTLGDHACKAVNYVFYTGYYSSIIFMTVLTIHRYVAVVHPLSVVMSRKSIHCYATSIVVWIISLSAAIPQAMFKTVVRNPIDTQSEASDVIKLCDFDGQINWKLWSTYLQNGFFIVAFLIIAFCYTVILTRLLRPTSHTRKKTVHLIFFIVLFFFLGWGPFNVAIFLDSLISWGISPFNECEVSKSIDYLMYVSQMVAYSHCCLNPVVY

>dreXCR1bd

WLVLKKVGLSSSADCLLLHLTISDLVFTLTLIPWTIYHIRGWIFGFAACRLFSWFIFLGLYSYMLFLTVMTVHRYIAVQHPVFASSVGNRGRLYAHVSSAVVWMISLGFSLPEMIFSETLDRYDGVQCVSYSRSEFWILFGYFSQIILFFLLPFLVIALCYARMGFTIHQSRIRSRNRHHAVCLILSIAIGFFICWAPYNIFLFIHSLEFLGVVELRETLSDTVYCVTHILAYFHCCLNPLVH

>dreCXCR7a

WVNMRSQRHYHETHMYILNLAVADLCVVATLPVWVSSLAQGGHWAFGQAACKLTHLLFSVNLFASIFFLTCMSVDRYLSVVRFREISHRRGRQVRRLVCAITWLLALFASVPDTYFLRSVKSQYSHVTLCLPVYPEDNPLQWMVGIQLSFVVLGFAIPFPIIAVSYALLANTLASTSTHSPSNGDQDRSVSKKVILMYIVVFIACWAPYHAVLLADALAMLGVLPLGCSSENGLFVALHLTQCLSLLHCCVNPVVY

>dreCXCR7b

WVNVRAERTRYETHLYILNLAIADLCVVATLPVSISSLLQLGHWPFGGAMCKITHLIFSVNLFSSIFFLTCMSVDRYLSVKLFGDTPSQRKRRTRQIICVGVWLLALIAALPEIYFLQAEKSDHSDAIVCKAVYPVESMKEWTVGIQMSFFMLGFAIPFPVIAVFYVLLANTIHPSVDQERRISRHLIFTYIVVFLVCWLPYHGALLLDTLAFLNVLPFNCTLENALYAALHLTQCFSLFHCCANPIIY

>dreCCRL1a

AVYAYCKQLKTMTDTFILHLAVADLLLLLTLPFWAVDAVHGWQLGITICKLVSGLYTINFTCSMMLLAYISMDRYLALSVGSRNQGLGRVFQKQHCGKLCVVVWMAAFLLGIPDLVFSTVRELPHKKSCLAMYPSDMALRAKASLEMVEVVIGFLLPLLVMLFCYTRVGRALLKLSEDRKWKKWQYIRVLLAMVGVFLVTQLPYNVVKFCRALDIMYTFVTHCGVSKKLDWATQITESLALTHCCLNPVVY

>dreCCRL1b

FIYLSHKRLRTLTDVFILNLAFADLLLLFTLPFWAADAVNGWQIGTAACKITSALYTTNFSCSMLLLSCISIDRYRALAKGSAHTPARNNSRKHRIIMCLVVWGIAIVLGLPDMVFYTVRVQHSSERYTCRAVYPHSMARAAKATLEILEVSLSFILPFLVMMFCYCRVGVALSQAATAGVHGGRRWRAFRVLIAVVGVFLLTQLPYNVVKLIKTLDVIYILVTECDVSKNLDLANQITESLALTHCCLNPVLY

>tniCXCR1a

LVISLSKQALPPSDLYLLHLALADLLLAITLPFWATSVTKGWVFGDAMCKIVTIIQELSFYSSILFLACISVDRYMVIVRALEVRRANRQKVSWCVCLAVWVVGGLLSLPGFFSSSFITNNSSQIVCAEQYDPGSADMWRLVTRILRHTLGFLVPLAIMLPCYGITIKRLLRIRGSIQRRRAMKVIVFVVVAFLLCWTPYHLAVMTDTFFRAKIVPYRCPARMAVDQAVFATQSLGLLHSCVNPVLY

>tniCXCR1ba

WVIFTRRQMLTPSDVYLFHLTIADGLMALTIPFLAAALVKGWIFGSFMCKFLPFVVEANFYTSILFLACISIDRYLVIVRANDNVRSRRRLCSWFLCLAVWALGSTLALPALFNDTAKLESDPQIMICSKNFDLGSATSWRLATRLFLHIFGFFLPLVVMIACYSIVIVRLLRTRGFQKHRAMKVIISVVVAFLLCWMPYHVTLMVDTALRTDLIPFDCSVRMAVTTALDVTNSLALVHSCINPVLY

>tniCXCR1bb

WVVCWIAKRRTSTDIYLTHLAAANLLFGLTLPFWAVDARSGWIFGTALCKLLSGLQEASEYGGVFLLACISVDRHLAIVKATRVKSSHGPVVKATCAAVWLVAAVLSIPTAVQRRHMGTEDPDRDICYEDVMDESSNRWYVLMHIARHVLGFFLPLAVMAVCYGSTLVTLYHTHNRQKQKAIRVIVAVVLAFIVCWLPYNVVLLIQLLIQSSLVEVESCGTRDRVWVALDVTKVLAFVHCAVNPVLY

>truCXCR3aa

TVLFRKRRSWSITDIFFLHLCLADVLLLLTLPLLAAQAFQPCGWCFELGLIPCKISRALFNVSFYCGIFLLVCIVLDRYLFYSRSTRIYSKSRPGLAHGTCLCVWICSLVLVIPIWIFVVTLNDPSGEKVLCVENFSASSGVGHLASRVLHHTLGFLLPAAILIICCCCVTLRPQRGSKQPHKRRAFTVVLWLVVVFLLCWTPYNITLIVDTVKSRAGKMGHSLETPLKMTFLLGYIHTCLRPLLY

>tniCXCR3ab

LVLVQNRRTWSVTDTFILHLAMADVLLLVTLPTWAAQAAQDEGWTFGTPLCKITGAVFMVNFYCGIFLLGCISFDRYLSIVHATHMYSHRKPWAIRISCMAAWLFSLLLSITDLVFLEAVSNDRLNRTMCVRVNSTQVNWQLSSRLIYHIVGFLLPSAVMIFCYTCILRRLRCSSQGLQKQRAIRVIIAVVAVFFLCWTPYNIVLLVDTFYSPSGSDKCGMHTSMEKALTVTSSVGYLHCSLNPILY

>tniCXCR3L

CVIRHYRSSQRGRSCASSLADTFLLHLAVSDLLLAFTLPLFAVQWAHEWVFGLTVCKISGALLSLSRYSGIFFLACISVDRYLAIVHAIGSGCKRNTFHAQAVCAIIWVVCLALSGADIAFKQVGDAATFGHYGLLCQAWIPRDTPHWQVVLQMISAFVGFGLPVFIMLYCYVCIFRSLCTATRRQKRKSLHLIVSLVSVFVICWAPYNCFQLADSLQALGVVAGGCLFGRVLDIGTLVTESLGLSHCALNPFLY

>tniCXCR4a

LVLGCQHRSKCSLTDRYRLHLSAADLLFVLALPFWAVDAALADWRFGAATCVGVHVIYTVNLYGSVLILAFISLDRYLAVVRATDTATGGLRQLLAHRLVYMAGAWLPAGLLAIPDLVFARTQEGGEGATLCQRFYPEENAPLWVAVFHLQLVLVGLVIPGLVLLVCYCVIVLRLTQGPLGAQRQKRRAVRTTIVLVLCFFLCWLPYGAGISVDALLRLELLPRSCRLEAVLGVWLAVAEPMAFAHCCLNPLLY

>tniCXCR4b

AVMGYQKKGRNMTDKYRLHLSVADLLFVLTLPFWAVDAASNWYFGSFLCVSVHMIYSVNLYSSVLILAFISLDRYLAVVRATNSQATRKLLASRVIYVGVWLPAAVLTVPDLVFARENMETAQSRLICQRIYPEETSLIWIAVFRFQHILVGFILPGLVILICYCIIISKLSQGAKGQALKKKALKTTVILILCFFSCWLPYCVGIFLDTLMMLNVVSSPCGLQHAVEKWISITEALAYFHCCLNPILY

>tniCXCR5

TVLLSRWRLLRVSEIYLLHLAVSDLLLLATFPFSILESITGWLFGDFSCKLTGLARQLNFLCGSFLLACIAIDRYLAIVHAISSLQHRRQRSVHLTCLSLWLVCFSMSVPNLVFLTATDSTHASRTSCSFYNYGIHAHNWVLATRALEHAVFFLSLVIMAYCYSTVVVTLVKSPRGPTQQGAIRLALLITLVFCVCWLPYNIASVLRTVDDLRTVDDLNRGVRNCESNLLLQAALGVTKSLGFSHCCLNPFLY

>tniCCR4Laa

WVIVFGTRLRSMTDVCLLNLALADLLLVCSFPFMAPQPSDQWAFGDAMCKMVLGVFNIFYCGIFFISLMSIDRYLAVVHAVYTIRARTRSLGITAAVVVWIAGFLASFPDLLFLKLQKVSSDFYCFPEYPKDTVNTSSHYWSVFSLFKMNILGLFLPLVIMLFCYSQIIWRLYHSHSSKKQAIRLVLIVVTVFLFCWIPYNITSFFKALELLGIYGTCDFSKGITLSLEITESIAYSHSCLNPILY

>tniCCR4Lac

WVLLQFIKLKTMADVCLLNLALSDLIFAVSLPLWAFNFQILALCKVMTAIYQVGFYSGTLFVTLMSLDRYVAIVHAVSSMRARTLHRGIIASISIWAVSIIIAAPQVKYASLEIDPENNLSQCQPLYPEDSMEFWKMRRNLSENIVALFVCLPIMIFCYVKILIVLSKSPNSKKDKAIRLIFAIVCVFVMCWVPYNVTVFLQTLQIFEILVSCSASRSISLTMSFAEIIALSHCCLNPIIY

>tniCCR6a

ATYAFYKRTKSMTDVFLLNVAVADLLFVLALPLLVYNQLSSWSMGTAACKLLRGSYSVNLYSGTLLLACISADRYVAIVQVRRSFRLRSLSHSRLICVLVWTAALLLSVPTFYFYHRYQPSHSQDEFLDGDNTSQSYVCEFQFLDNSTAWRTKVAVPSTQLAVGFVLPLLVMASCYSAVLLTLLRARNFQRHGCHLPYNLALLYETTTMFQLQSCERSDTLQLAKALTQTLAYLHCCLNPLLY

>tniCCR7

LTFFYFKRLKTMTDVYLLNLSFADLLFALSLPFWAANTMTKWVLGEEMCIAMYTVYKVSFYSSMFLLCCISVDRYFAISKATSAYRYRSQTMFLSKVSSAVVWVAALIFSMPEMRYTSVNNNTCTPYTGSKDQLRVIIQVGQIVLAFALPLVIMSICYSSIIKTLCQAQNFERNKAIKVILAVVAVFLVSQVPYNLVLFWSTLVTAKGGTTSCSYDNNLLYATDVTQCLAFFRCCLNPIVY

>tniCCR9a

TYLHLRRQLKTMTEVYLLNLAVADLLFLVTLPLWAAEALSGWTFGPALCKVIAAVYKVNLFSSMLLLTCISVDRYVVIVMATKARSSQQERRRRSLLVCLGVWLLALLLAIPELAFATTKGVGSLQYCRMVFPDGQGNRTKILALSLQVSMGFCLPFAIMASCYAVIIARLLRTRNFQKHKAMRVVLAVVLAFVLSQLPYNGVLVAEALQASDMPVTDCEELKRFDRVKQVLMSLAYVHACLNPFLY

>tniCCR9b

IYSTVRNRLKTMTDVYLLNLAVADLLFLCTLPFLAVEAIKYWNFGLALCKTVLAVYRINFFSGMLLLTCISVDRYISIVQVTKAQNTKKQRLFWSKLICLIVWVVSTLLALPEFIYARVKTKQRDQSLCTLIYWDNSENQIKILVLSIQICMGFWLPLMVMIFCYSVVIRTLLQARNFQKHKALRVIFAVVLVFVLSQLPYNSLLIFETTQAANTTMSSCETRINLELAKQITKSLAYAHACLNPFLY

>tniCCR10

FASRCEGLRSMTDVFLLHLALADLLLLLTLPLQAVDTQLGWILPVSLCKATRACYAVNTYSGLLLLACISVDRYLMVARAQLRQWLRRWTFKAATLVALAVWASALTLSLPEILFSGVSGSGSKAYCGVLNSPEAKMATRVAIIAVFCLSFLVMLSSYSLIAMVLWGGQAQRRGKAWQRQRTLKLMLALVLLFLAFQLPYTLVLLRKLAGEFCGLLLEYVTCTLVSTRCCLNPILY

>tniCCR11a

VMIFMTLDRYMVIMHAITVARYRTLRAGIFVTMVLWLLSFSVSLPTFIFTEVTNESYGSSCYYAPEKDSWQIYDLFVINVLGLMLPLVVMIVCYSRIIPRLVNMRSTKRHRVIKLIISIMLTFFLFWAPYNFYFFLKFLHRKGKLVGDPCQIEEHLGLTGILTETFAYTHCCLNPIIY

>tniCCR12a

FIICKYEKLDTVTNIFLLNLVISNILFASSLPFLAIYHQSEWIFGNVLCKTVSSAYFIGFYSSILFLTLMTFDRYLAVVHAIAAAKCRKRVYAIIATVAVWCISILASMKELVLRNVWESPSNGLVCKESGYMESTMKVWRLVSYYQQFFGFFLIPLFTLMYCYITITIRILSTRMREKCRTIKLIFIIIFTFFICWTPYNVVIFLQAIQDSRGDEEESCSETLDYALYISQNIAYTYCCISPVFY

>tniCCR12b

FYISRFEKLTTVTNILLLNLILSSMIFMSSLPFMAVYMQLSNWIFGTVICKIVGGMYYLGIYSSVLFLTLLTFDRHLAVVYSLSAVRMRNVSYARISCGVVWLVSILACAKQMILRTTFEHSFENRIFCDEYPLISPLYSQLRSAGFYLQLFLFLLFPLVVIIYCYVRIAITVISSKLASKFKTVRLILFIVVLFFMCWTPYNITLLLDADICEESQKLGYVLQITQNLVYLYFCICPILY

>truXCR1ba

AILLFGEKLKNITSLFILNLACSDLVFTLTLPFWAYYQLHHWVFGEYACKLLTAAYIVGVNSSVILLTALTVDRFVTVVLQWPNDPSRRKRFAVVSCTAAWLISAAASVNDAITVKVETQWNNLSSCEDTSPESHVNLGYYLHVSLLFFLPFTIIVFCYSFILKTVLQASKRRTYCTVVMILCIVAAFFICWGPYNILLIVKIFYKPQSCYAEDTLYVAYSVCRIIAYSHCCMNPLLY

>truXCR1bc

VILVKYENIKSLSNVLIMNLAVSDLFFTIGLPLWIHSHMNEWTLGEPACKMVMFVFFVGYYSSSILLVLMTAHRYIAVMRPLSSIMSNKGFCSALASPVIWVVSLIFAVPALIFTSVLQNNRCVTVRSIWNLFGIYQQNFFFFLNSVVFLFCYPQIICRLLRPTAQRRKSKTLKLIFILMVVFLVTWAPYNIVIFLKSFQFYPNSHDASTLQEKCNFTKRLEYAFYISRLFAFSQCCLNPVFY

>tniCXCR7a

WVNVRAQRDSTPRHETHMYIAHLAVADLCVCATLPVWVSSLAQHGHWPFGQVACKLTHLLFSVNLFGSIFFLACMSVDRYLSVTRRRNNEEGTRRKLIRRGVCVGVWLLALVASLPDTYFLQTVKATHGDTMLCRPVYPEENPREWMVGVQLSFILLGFVLPFPIIAVFYILLARAFTGCSSSSSSTVEQERRVSRRVILAYIVVFLGCWGPYHGVLLVDSLSQLGLVPLTCTLENVIYVALHLTQCLSLLHCCFNPILY

>tniCCRL1a

AVYAYLKRLRTMTDVFMTHLAVADLLLLLTLPFWAADAAQGWELGTATCKMVSSMYTVNFTCCMMLLACISVDRRLALAAARGEGRGRLLQRVFTKKHCWKVCFAVWALAFGLGLPDLVLSEVRWLSNRSVCLVVYPPSMVGGGKAGLEMMEVLLGFLLPLLVMIFSYWRVGQALKGLPVESRSRKWRALRVLLTVVAVFVVTQLPYNVVKVYRAVDSVYTLVTHCASSKALDRAAQVTESLALCHCCLNPVLY

>tniCCRL1b

AVYASRVRLRTLTDVCILNLAVSDLLLLFTLPFWAADAVHGWRLGSTACKLTSFLYSTNFSCGMLLLACISVDRCCAVVRSFSGKTSASPRVRRRWLLVCLVLWAVASFLGLPELIFSTVKHSHHRMACTEIYPPSMARAAKAALMLLEVLLRFLVPFLVMVVCYSWMGRVLSRAAGVQRERKWRALRVLLAVVAVFLLTQLPYNVVKLCRAMDTIYVVVTDCEVSKGLDRALQVTEGLALAHACINPLLY

>cmiCXCR1a

IVILHNRRTMSSTDIYLLHLAIADVLFAITLPFSAADVINGWLFGDAMCKIVSVLKEVNFYSGILLLACISIDRFLAIVYSARANKQRSQFLTNVVCGGVWLFAIILSFPILVKGVFRSPDSERILCYEVLDGKSSATWRVATRFLRHIAGFLIPLSIMLFCYSVTINRVLKTKGFQKQKAMKVIIAVVLAFLICWLPYNITVFIDTLIRSKIINETCEMRNPLDKALFATESLGFLHSCINPILY

>cmiCXCR1b

IVILHNRRTMSSTDIYLLHLAIADVLFAITLPFSAADVINGWLFGDAMCKIVSVLKEVNFYSGILLLACISIDRFLAIVYSARTNKLKNQFLTNVVCGGVWLFAILLSLPILVKGVFRPPDSERILCYEVLDGKSSAKWRVATRFLRHIAGFLIPLSIMLFCYSVTINRVLKTKGFQKQKAMKVIIAVVLAFLICWLPYNITVFIDTLIRSKIINETCEMRNPLDKALFATESLGFLHSCINPILY

>cmiCXCR4

LVMGYHKRYRSMTDKYRLHLSVADLLFVLTLPFWAIDTVNWYFGDITCKIVHVIYTMNLYSTVLIMAFISLDRYYAVVHATNSTRQRKMLANRFVYVGVWLPSILLTVPDMVFAKTTQLMDRVVCDRIYPTETFQTWLVAFRMNGVLVGFVLPALVILTCYCIIISKLSQSTGLQKRKALKS

>cmiCXCR5

TVLICTKHTRTPTDSYLLHLTLIDLLLSLSLPFTAIQGIFQWYFGQVVCKMVGTMYKLNFFCSSLLLGFISFDRYLAIVYAVQTYKKRKQVVIHCICAGVWALCLLLQLPNTIYLRVETQENKSMCTYPWASVERWLLTEQILYHVLGFALPLLVMCYCYTMVGKTLWRCQNFKRRKAVRVVLLVTAVFFLCWTPFNLVIFINTLSKLELINSQSCHFEHDLGVALRVTECIGSVRCCLNPILY

>cmiCXCR6

VIYVFYEKLKTVTNIYMVNLAVADLLFLCTLPFWAVNACHGWIFDTFMCKVMNGAYTVNFYSCMLILTCVSINRYNVIVQATKMLNCKYRRCHSVVCTAVWLLAIILSLPQFIFSEARTDSSSKICSMVYPTNLSVAIKVQVNIIQMTVGFLIPFAAMVICYSIIAKTLLHGKGFQKHKSLRIIFVIVIVFVFCQLPFNIVKLMETLSIINNVSIKCTDSIKVDYAIIVTKCIAYVHCCLNPILY

>cmiCCR4

WVLIQYKGLKSMTDIYLLNLTISDLLFVVSIPFWLHYMLHEWVFGNVLCKVINAGYLIGFYSGILFIMLMSIERYLAIVHHVFAFKVRKVRYGIISSAIIWFVAICASLPELIFYNIKTVSGKTECSIFYPNTTISTWKVFGFFQVNIFGFLIPFSVMTFCYSRIIITLLGNKASKKHRAVKIIFTVMIVFFVFWIPYNIVLFLNSLLEMEILNKIGNEGRLQMALQITQSLAFTHCCVNPFIY

>cmiCCR6

ITYVFYKKMRSMTDLYLLNLAVADIMFIATLPFWAVYESNQWIFGTFVCKFLKGVYSINFYSGVLLLAVISVDRYIAIVHATKSFNYRGKALLYGKIICAVTWLLVITASLPDFIYCEVYDFTTSGQKLCEIRYPKEWSKIGKLIAPSIQLSLGFFGPLLVMFFCYSMIIHTLLRVRSLQRHKAVRVVMAVVAAFVVCQVPYNITTLIETSVKNLHCEALKRLSLAKTVTKCLAFFHCCLNPILY

>cmiCCR7

LTFIYFKRLKTMTDIYLLNLALADLLFLFTLPFWAVSVVKQWIFGQPMCRTVCVLYKLSFFSGMLLLMCISIDRYFSIVRAASAHRHRSRAVYYSKIISGGVWILAFILSMPELLYSEEIQRGGILVCKLRSNNNTMMFTTSAQLAMGFFIPFLVMIFCYSVIVKTLLISRSFEKNKAIKVIFSVVVVFILFQLPYNSVMLLDTISSIKANITNCDVNKRLNMALDVTKSLAYVRCCLNPFLY

>cmiCCR9

VLYAFYKRIKTMTDVYLLNLSIADLLFLCTLPFWALNASIGWRFGSSLCKIVSGVYKINLFSCMFLLTCISFDRYIAIVLATKAHYSKNKRLLQSKLVCLFVWIIATILSFPEFAFSSTNESQMTCSSNYPSKNMKVVAFALQVTFGFLLPLVIMMICYSLITWTLLQTKRFQKHKAIKVIVAVVSTFVLTQLPYNSFLIVKLLDADNITITDCNTLKNVDIATQITQSIAFLHCCLNPFLY

>cmiCCR10

TIYICYRKLKSMTDMYLLNLAIADLLFIFTLPYMAASSVHGWIFGNAMCKIVQSLYSMTFYSGFLFLTCISVDRYIVIVRATLAHRLRGKTIYYSKVTCLIVWFVSILISLPQFIYSHVELEEQVCWMTYPDVANGWMKVGTLITQLVIGFLIPLLVMLFCYSIIIRTLLQARNSEKHKAFKVIMAIVVTFVVFQTPYNIISILETADTLNHSSSVCQQRSQRDITIQVTSCLAYSRCCLNPLLY

>cmiXCR1e

CVLVKYEYLRSITNIFILNLAVSDLLSAFCLPFWTMHHLTGWIFGDIMCKVVCAMFYTGFYSGIMFLTLMTFDRYLAVVHAVSALRSRKVRYAAITSVIVWGSSILATLPHAIFSTLTEESTCDYIYPKETALPWKLLRYFLQNVLFFVIPFAIIVYCYLRIIQTLIKCRTMQKYRTVKLIFIIVVVFFLCWAPYNVVIFLTALKDLRIFKTCEVINQIEYADFFTLNIAYFHCCLNPLFY

>cmiXCR1ba

WIMLRYEKLKTITDIFIVNLAISDLLFAASLPFWAKDHVSGWVFGNAMCKLLSGVFFVGYYSGIMFLTLMTADRYFAVVHAVYAARSRKTCYAVTASLVVWAISLSASVPEFIYSTEITWNQTEFCMANYPEDSEHIWLLFGYYQQIILFFLIPSVVIVYCYYKIMNIVLRCKARKKYKAVKVILCIVVIFFLCWAPYNMMIFLVSLKQLNVSPFTTCEMSKHIDYAYFISRNLAYFHCCLNPVFY

>cmiXCR1bb

WIMLRYEKLKTITDIFIVNLAISDLLFAASLPFWAKDHVSGWVFGNAMCKLLSGVFFVGYYSGIMFLTLMTADRYFAVVHAVYAARSRKTCYAVTASLVVWAISLSASVPEFIYSTEITWNQTEFCMANYPEDSEHIWLLFGYYQQIILFFLIPSVVIVYCYYKIMNIVLRCKARKKYKAVKVILCIVVIFFLCWAPYNMMIFLVSLKQLNVSPFTTCEMSKHINYAYFISRNLAYFHCCLNPMFY

>cmiCXCR7

YVNLKTKRTQYETHLYILNLAIADLCMVATLPIWLPSLVQHGSWPFGEFMCRLTHLVFSVNLFGSIFFLTCMSVDRYISVVRVGESVDRRKGVIRKIIIVYVWIFALVVSFPDILFLKTVTLPSNGETYCYPSYPEENFRKWMAGMEMVYIIIGFVIPFPIITVSYFLLARAISSTSDQENKNSRNIIFVYVIIFLICWLPYHTVLFIDVLWFMNLISFSCELESFMYISLHLTQCFSMVHCCANPILY

>pmaCXCR4

VILTCFTKKRTSSDLYLMHLAAADLLFVLTMPFWAVGSATEWVFGNVLCCLVNFTFTVNLASSILLLACISIERYLAIVRATKTDKVRRKFATKVTCGAVWALSLLLAMPDLVFSHVYIAPLSGHQLCEHVYPESASELWRTSLRALHHVLAFALPGIVIVFCYVMVIRTLSQLHNHEKRKALKVVVAIVAAFFVCWLPYNVVTLLDTLMRLDAVVNSDCEMEQRLGVAVAVTEGVGFSHCCFIPVLY

>pmaCCR13a

QRRGNGSGTWDTTQVFLLNLAAADLLLVATLPLWARHAAREWPFGEWACKLATALYSVSFYCGVFVLTCLSVDRYLVVVRATRCRGVRWRRRRAWVACAGAWSAAMLLSLLDVLSSRVQLQADTPDSAPRTVCQSLHAGPGGHVRKAALAVFQTAACFLLPFAIMLACYIRIAVTLAHTKSRGSRRKAVRVIVALVLLFFLCWLPYNVVLLVVALADLRGTWLGCRTHEALLYARQTTETLAFTHCCLNPLLY

>pmaCCR13b

LILVKFKRHKEVTNFYLLNLSIADLIFAATMPFWAHESANGTWIFGNIMCKLVTACYSVNFYSGIFLLVCMSIDRFNAVVLATKFNKIRRLQNVKYICAFVWGFASSLSIFDIVYVKAQTFDDGITITCGHNIESKILDWKLGLAIVQNIFGFLIPFIIMLSCYSSITITLMHTKNYKKLKAFYVILAVVLAFFFCWLPFNVIMFLEAYLGYNDAMTCKAQTSLMYAKQAAEGIAFSHCCLNPFLY

>pmaCXCR7a

WLLWRPGRGGRPEVRCYVLNLAVADLFVVLTLPVWTVSLLGHGAWSLGDFMCKFTHFVYSVNLYGSIFFLVALSADRYVTLVLSPDLLGRLAERRARSRALACAGVWLLALAVSMPDIVYIESGTAPHTNQTYCVAAYPLGAFSQWMAGMQLMCNAVGFALPFPIIAVCYALVARALRDSARAGSPREARGARRLLVAYVAVFVLCWLPYHAALFVDALALLEVVELSCGAERFLYSALHVTQCFALVHCCANPALY

>pmaCXCR7b

CFNVFFHKNRFETHLYVVNLAISNLCIVLIMPVMVASFLHDDQWLYGNFLCKLTNVTFSVNLFAGVFFVTAMSVDRYITFVHFHEASSRRKERARLLICAIAWLLAVVASLPEIIYIRAVTTPSGETHCRADLPPDHFFRWMASINLLYNTLGFVVPFPMIVVSSVLLYRAIAWHGLSERNNGRAIVLVYATVFAACWTPYHALLLVDALALLRVLRLGCAMDEALYAGLHVAQCLAVGQCCVNPVLY
