# Supplementary material for: Systematic classification of vertebrate chemokines based on conserved synteny and evolutionary history
Source: Genes Cells. 2012 Nov 12;18(1):1–16. doi: 10.1111/gtc.12013 (PMC3568907; doi:10.1111/gtc.12013)

Fig. S2

Chemokine and chemokine receptor gene clusters.

Human chemokine and chemokine receptor genes displaying a high level of promiscuity in ligand-receptor binding are boxed with a red line. Hsa, Gga and Ola indicate human, chicken and medaka chromosomes, respectively. 'n' indicates the gene copy number.

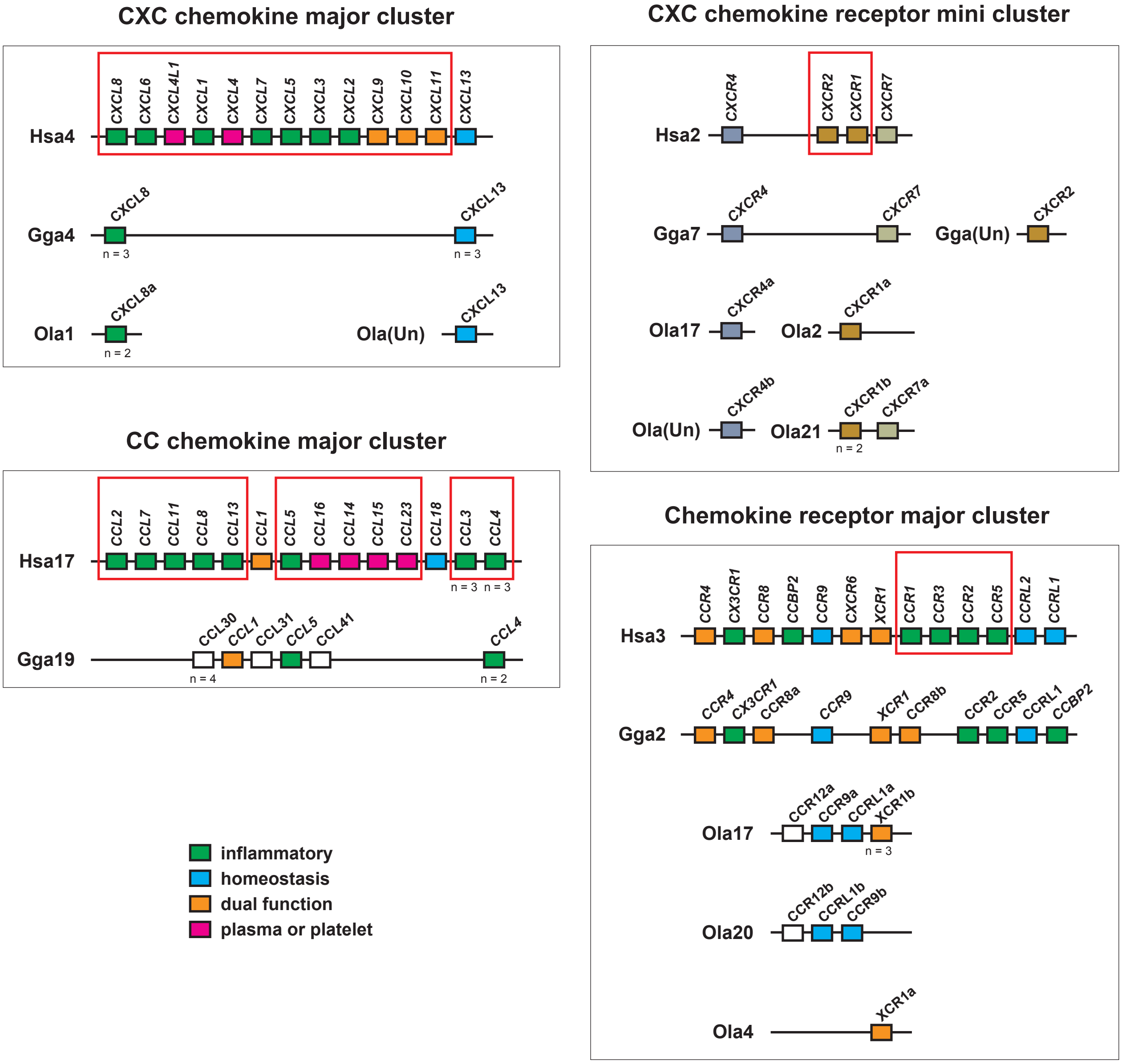

Supplement: Supplementary file 2 [file gtc0018-0001-SD2.pdf]
