# Supplementary figures and images for: Systematic classification of vertebrate chemokines based on conserved synteny and evolutionary history
Source: Genes Cells. 2012 Nov 12;18(1):1–16. doi: 10.1111/gtc.12013 (PMC3568907; doi:10.1111/gtc.12013)

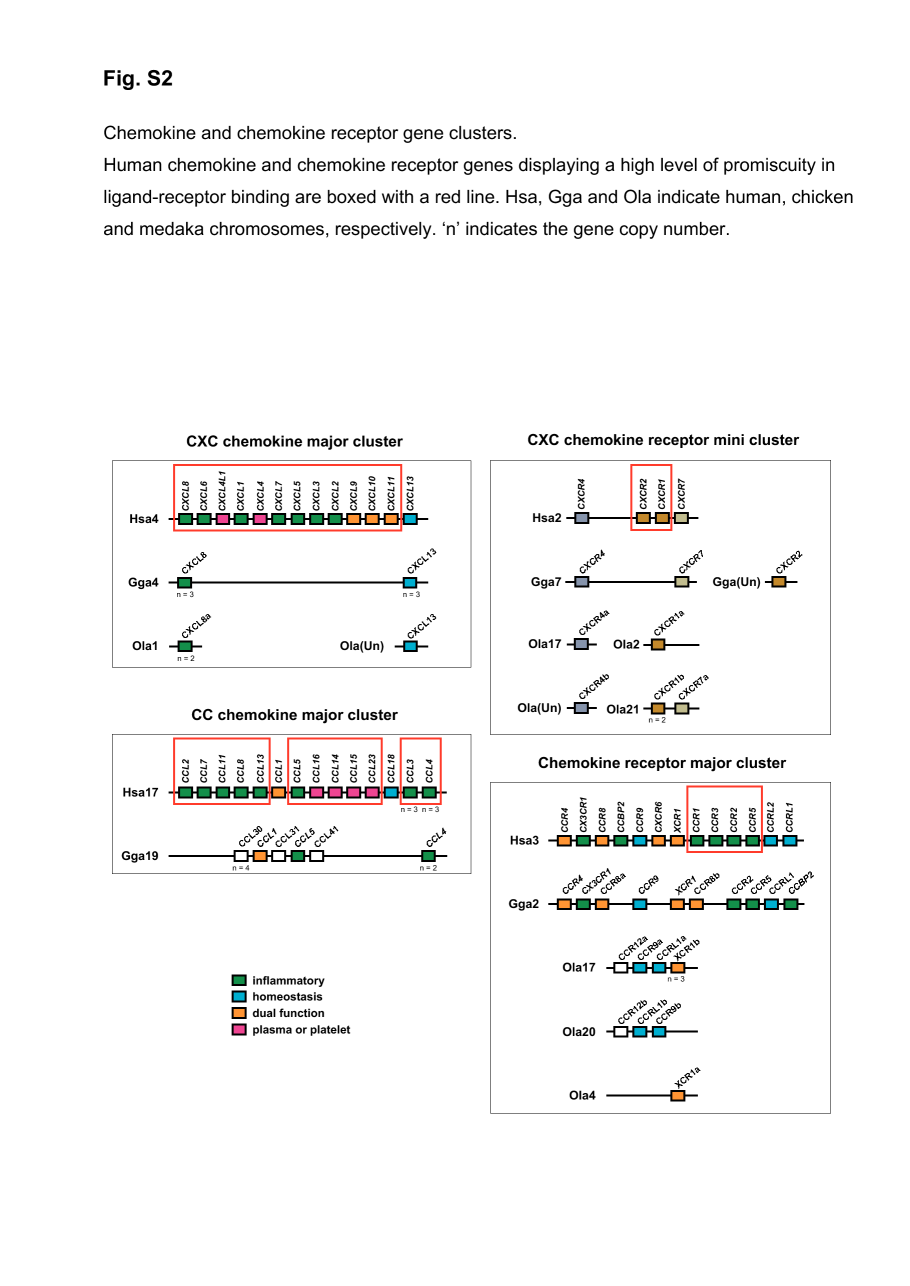

Supplement: Supplementary file 3 [file gtc0018-0001-SD12.png]

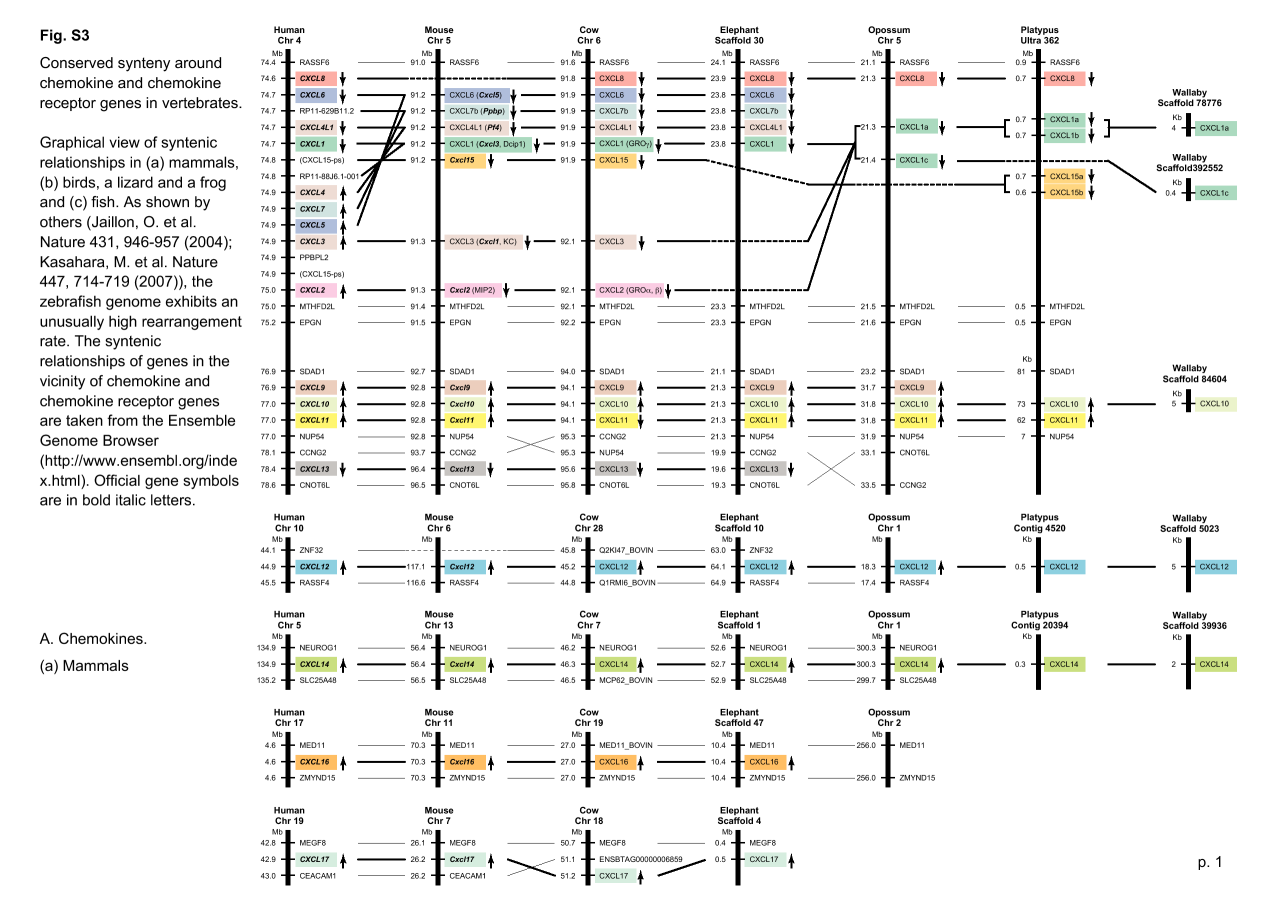

Supplement: Supplementary file 6 [file gtc0018-0001-SD13.png]

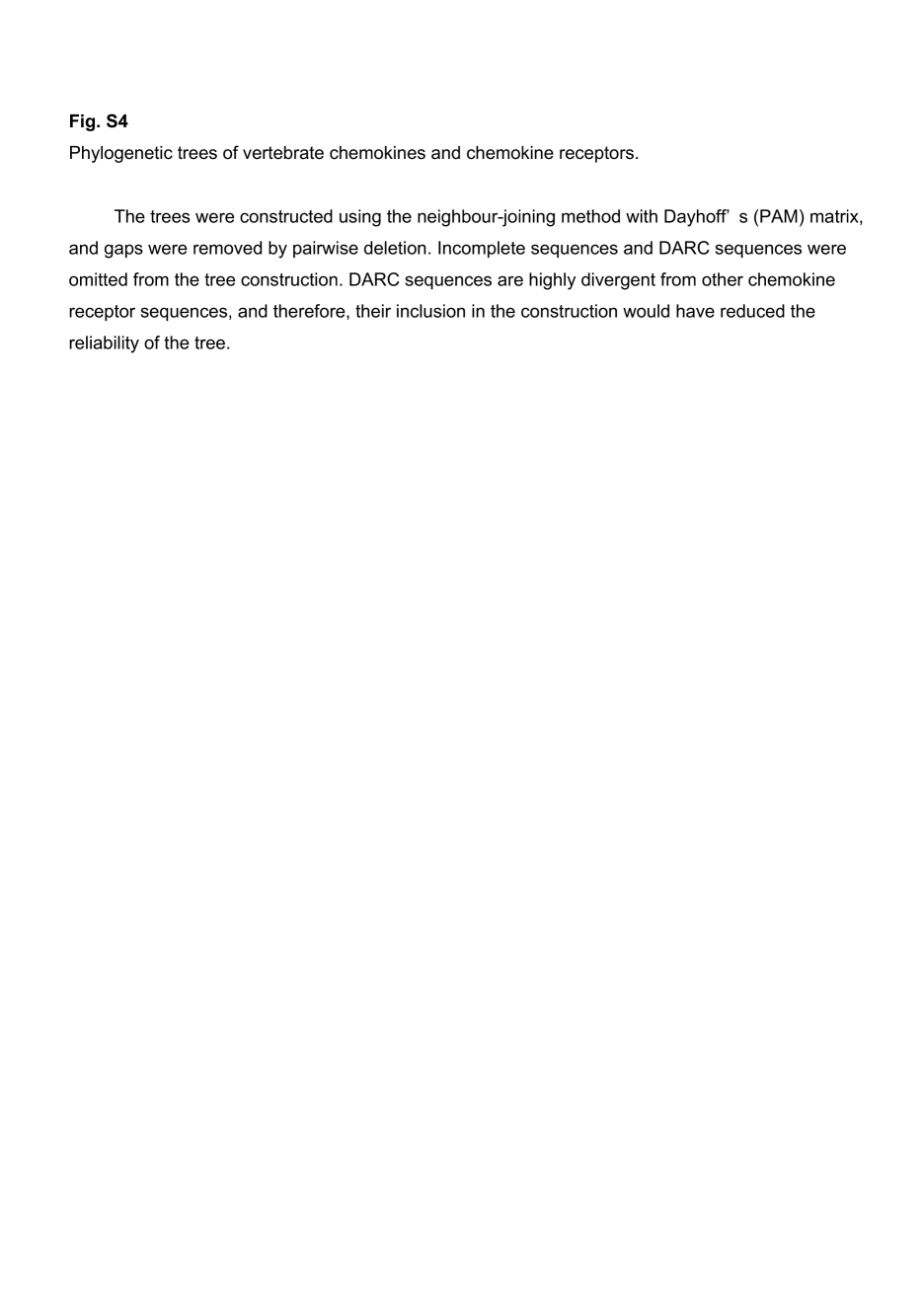

Supplement: Supplementary file 8 [file gtc0018-0001-SD14.png]

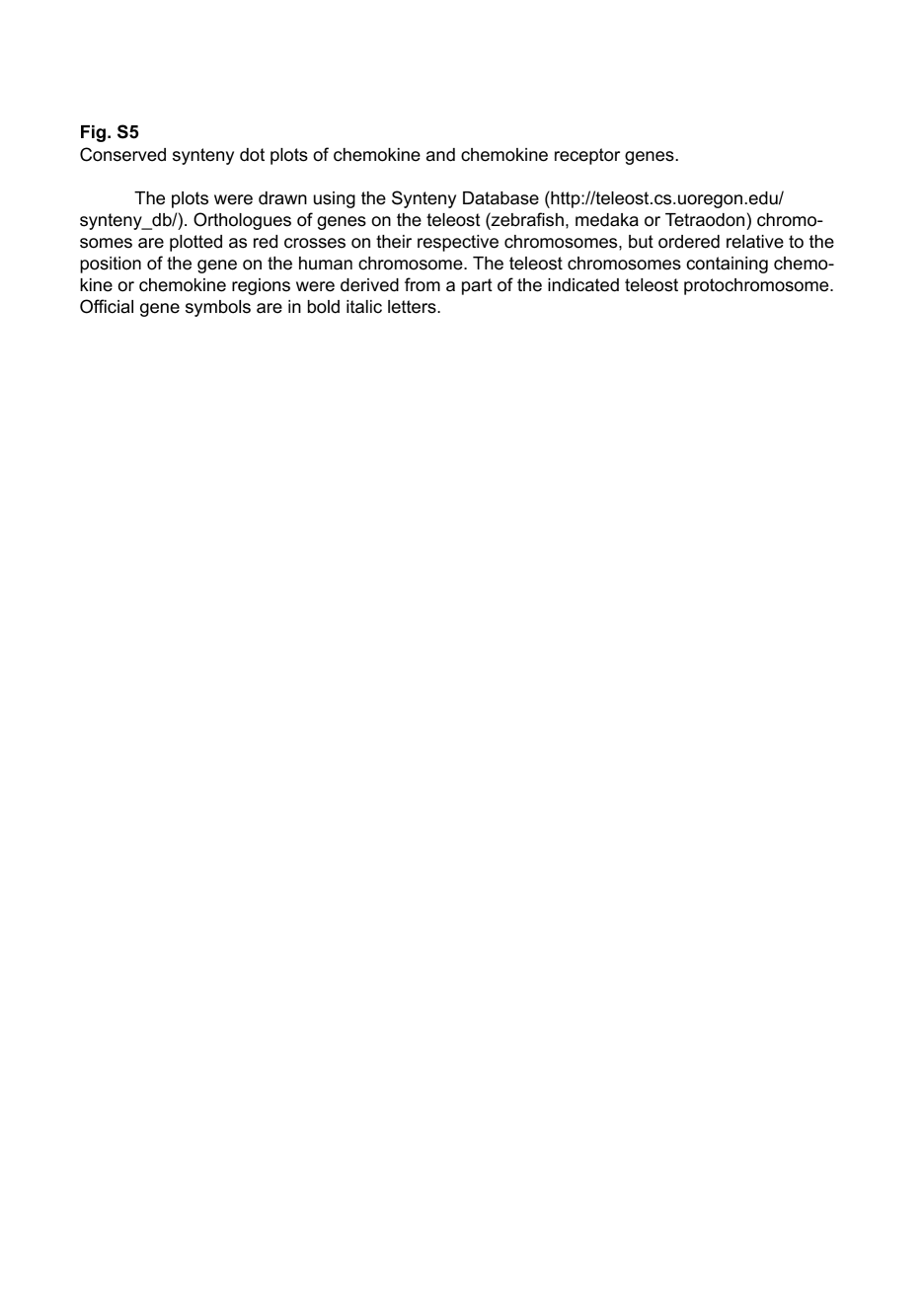

Supplement: Supplementary file 10 [file gtc0018-0001-SD15.png]

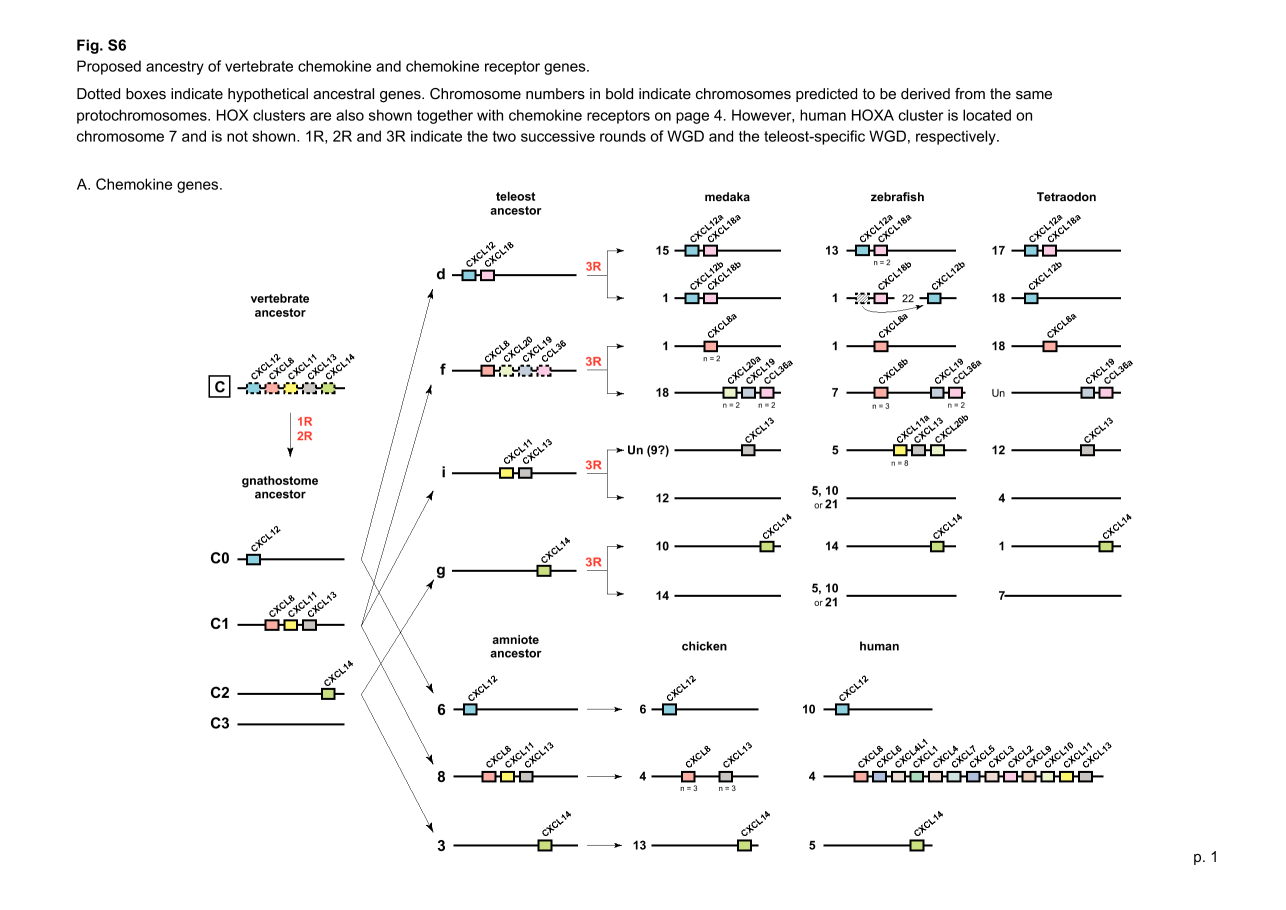

Supplement: Supplementary file 12 [file gtc0018-0001-SD16.png]

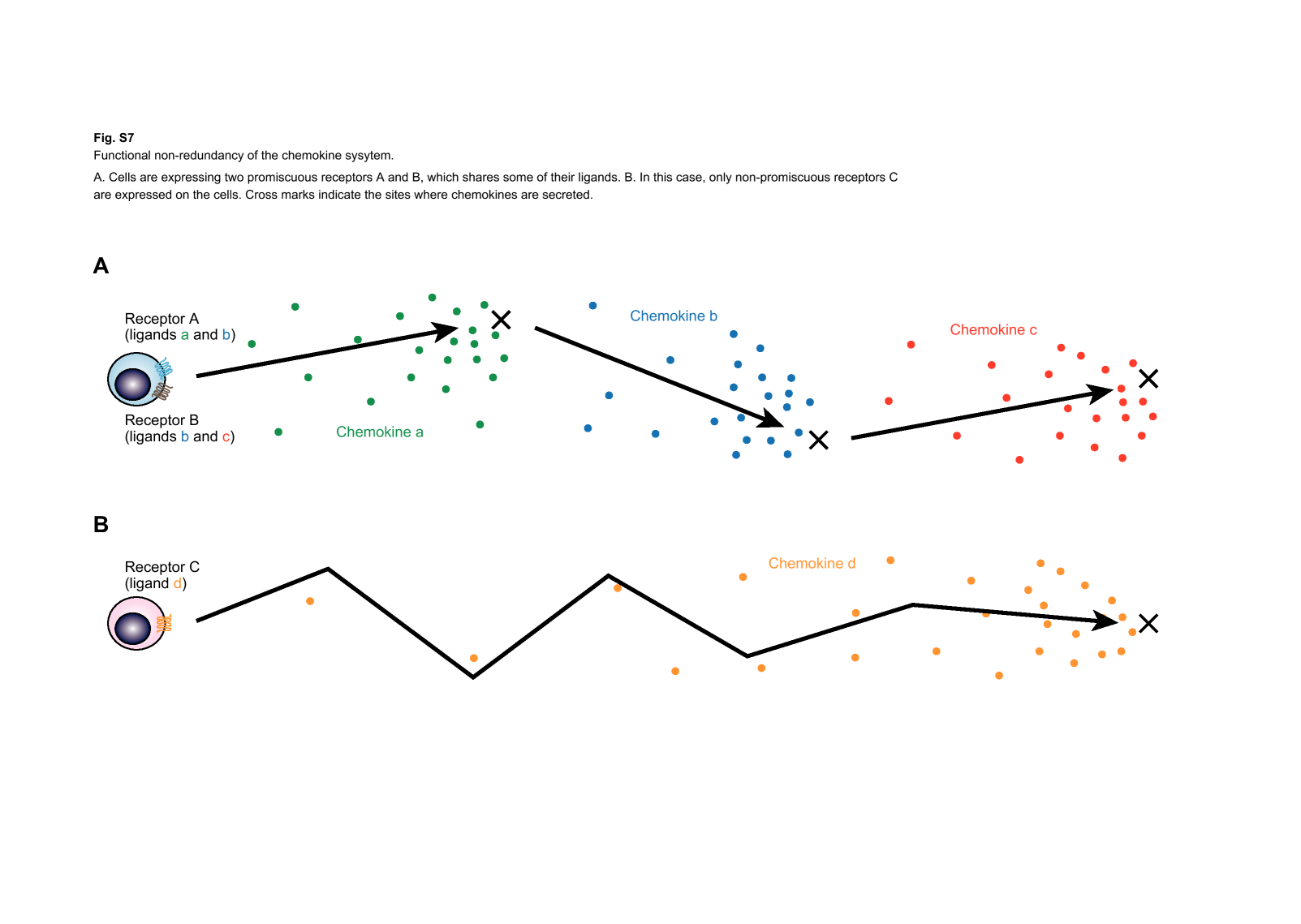

Supplement: Supplementary file 14 [file gtc0018-0001-SD17.png]
