# Supplementary material for: Systematic classification of vertebrate chemokines based on conserved synteny and evolutionary history
Source: Genes Cells. 2012 Nov 12;18(1):1–16. doi: 10.1111/gtc.12013 (PMC3568907; doi:10.1111/gtc.12013)
Supplement: Supplementary file 5 [file gtc0018-0001-SD3.pdf]

Fig. S3

Conserved synteny around chemokine and chemokine receptor genes in vertebrates.

Graphical view of syntenic relationships in (a) mammals, (b) birds, a lizard and a frog and (c) fish. As shown by others (Jaillon, O. et al. Nature 431, 946-957 (2004); Kasahara, M. et al. Nature 447, 714-719 (2007)), the zebrafish genome exhibits an unusually high rearrangement rate. The syntenic relationships of genes in the vicinity of chemokine and chemokine receptor genes are taken from the Ensemble Genome Browser (http://www.ensembl.org/index.html). Official gene symbols are in bold italic letters.

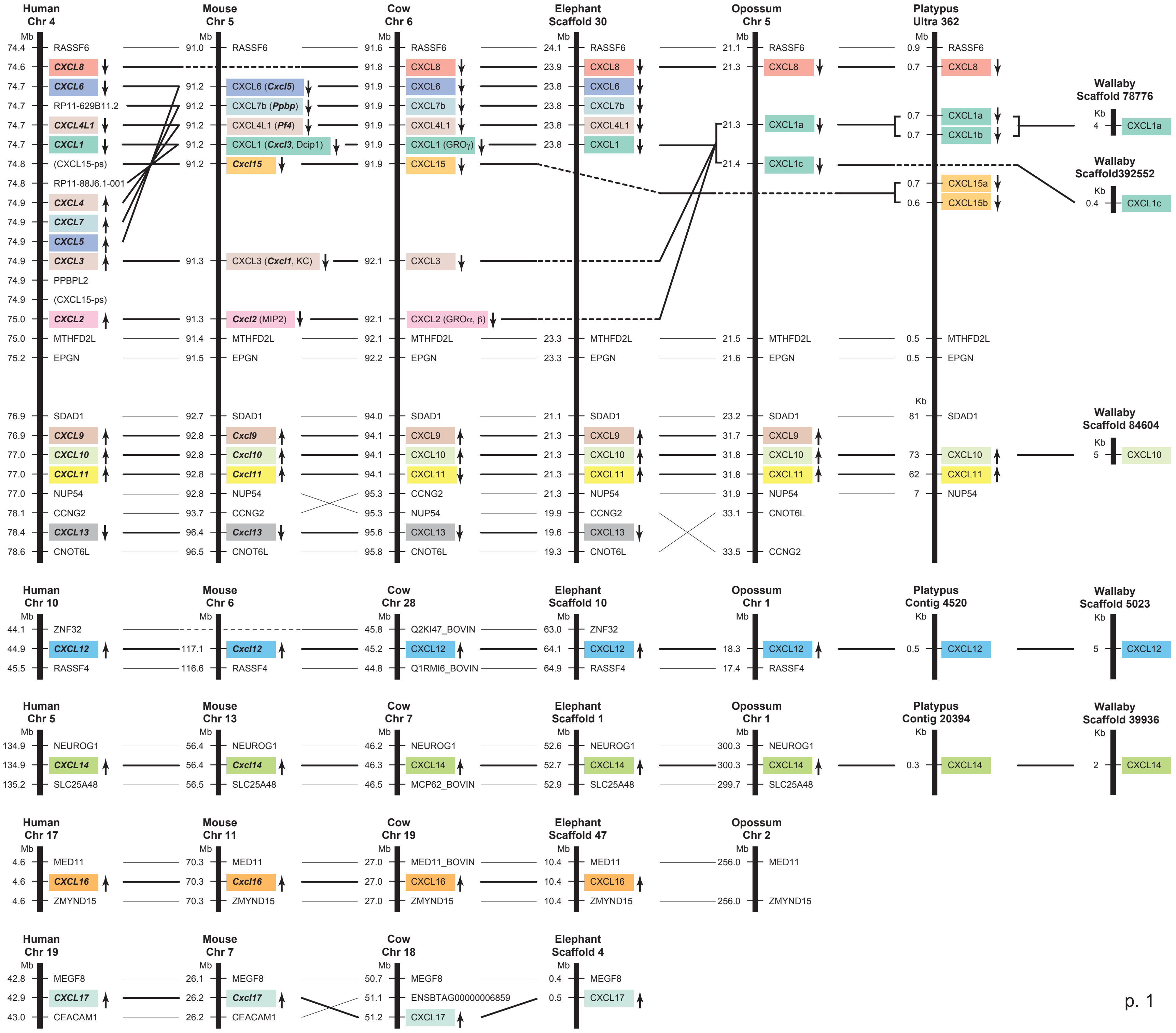

A. Chemokines.

(a) Mammals

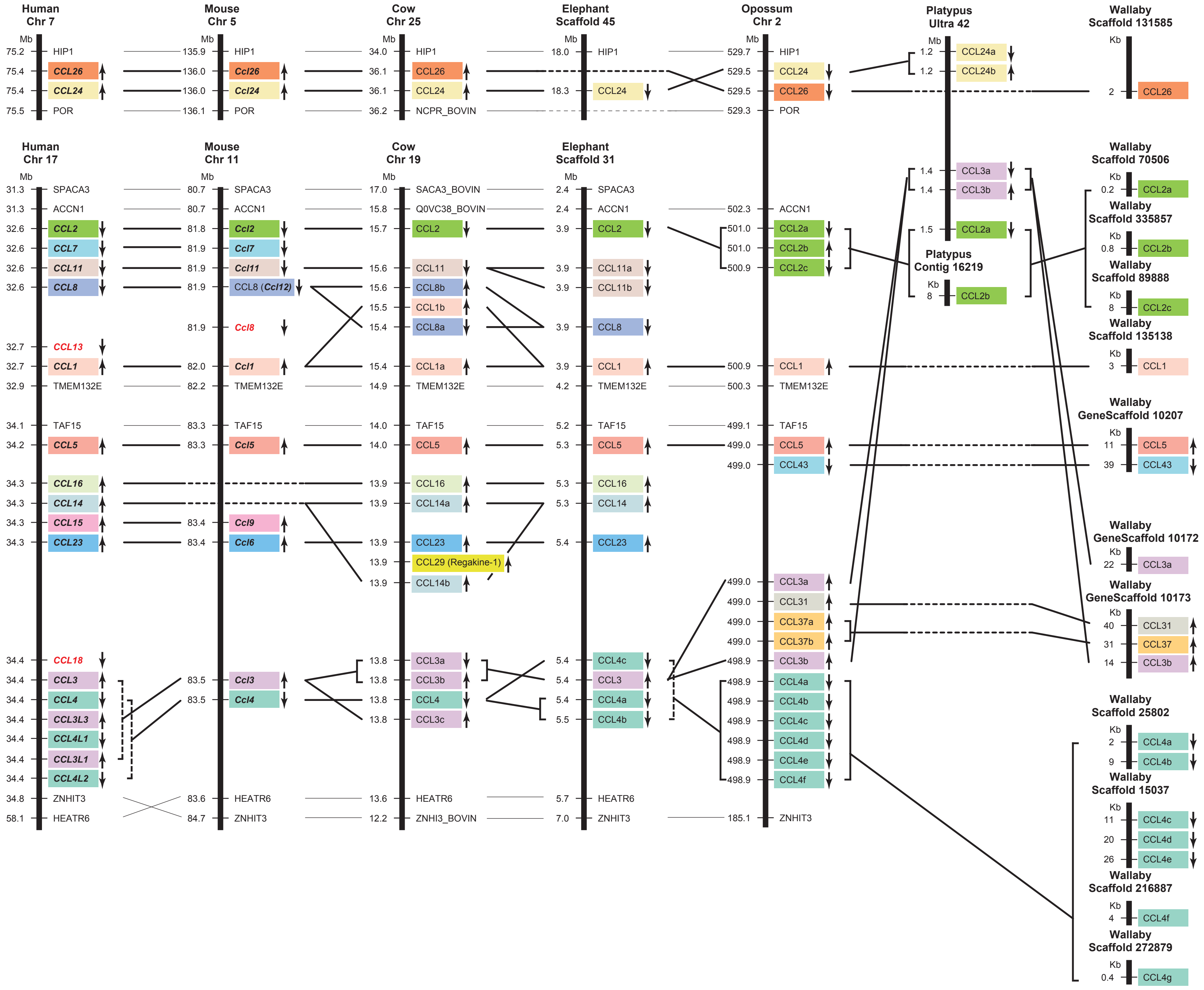

Human Chr 7
Mouse Chr 5
Cow Chr 25
Elephant Scaffold 45
Opossum Chr 2
Platypus Ultra 42
Wallaby Scaffold 131585
Human Chr 17
Mouse Chr 11
Cow Chr 19
Elephant Scaffold 31
Wallaby Scaffold 70506
Wallaby Scaffold 335857
Platypus Contig 16219
Wallaby Scaffold 89888
Wallaby Scaffold 135138
Wallaby GeneScaffold 10207
Wallaby GeneScaffold 10172
Wallaby GeneScaffold 10173
Wallaby Scaffold 25802
Wallaby Scaffold 15037
Wallaby Scaffold 216887
Wallaby Scaffold 272879
Mb
Kb
HIP1
CCL26
CCL24
POR
NCPR_BOVIN
CCL24a
CCL24b
SPACA3
SACA3_BOVIN
ACCN1
Q0VC38_BOVIN
CCL2
CCL7
CCL11
CCL8
CCL8 (Ccl12)
Ccl8
CCL13
CCL1
TMEM132E
TAF15
CCL5
CCL43
CCL16
CCL14
CCL15
Ccl9
CCL23
Ccl6
CCL29 (Regakine-1)
CCL18
CCL3
CCL4
CCL3L3
CCL4L1
CCL3L1
CCL4L2
CCL31
CCL37
ZNHIT3
HEATR6
ZNHI3_BOVIN

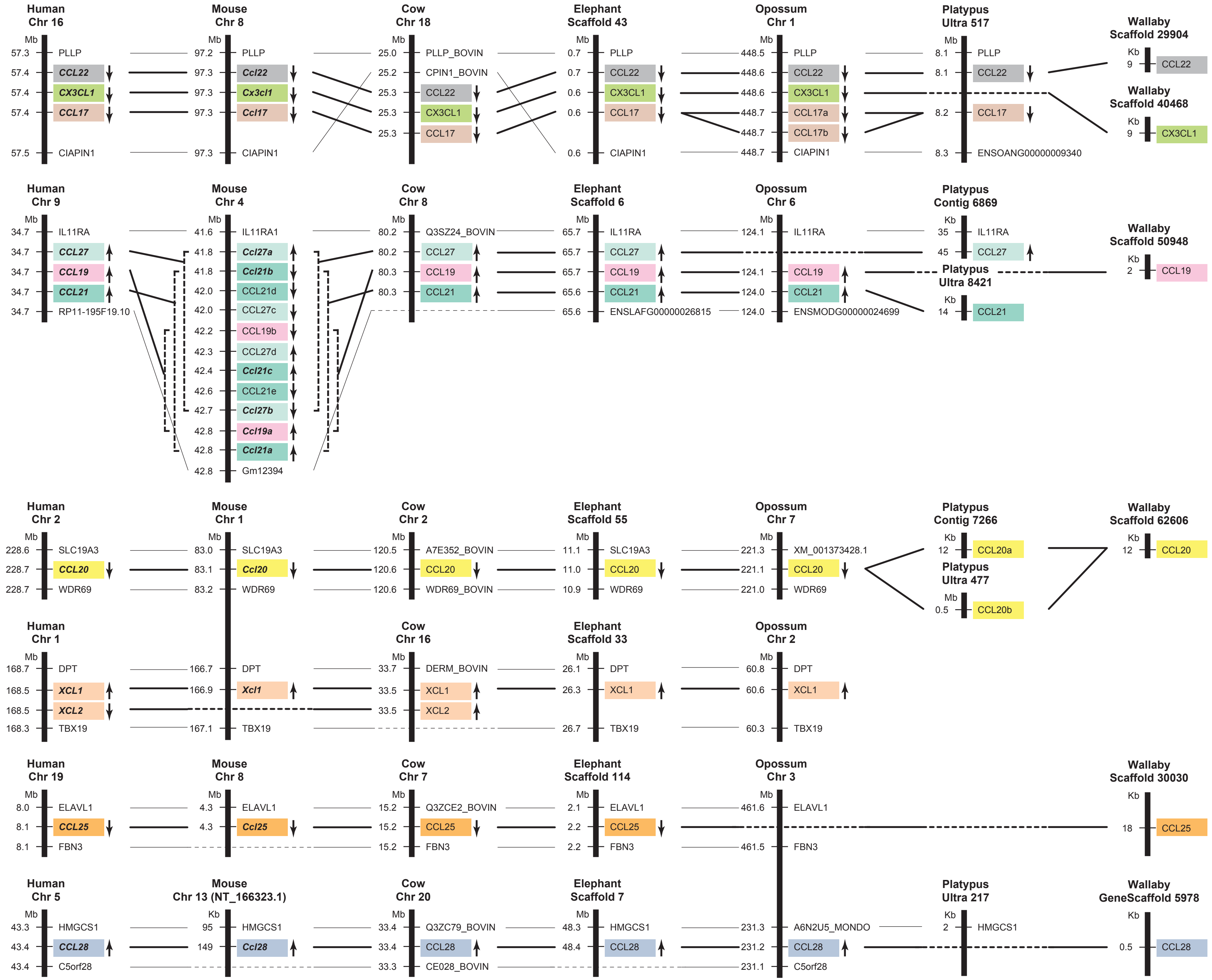
Human Chr 16
Mb
57.3 PLLP
57.4 CCL22
57.4 CX3CL1
57.4 CCL17
57.5 CIAPIN1
Mouse Chr 8
Mb
97.2 PLLP
97.3 Ccl22
97.3 Cx3cl1
97.3 Ccl17
97.3 CIAPIN1
Cow Chr 18
Mb
25.0 PLLP_BOVIN
25.2 CPIN1_BOVIN
25.3 CCL22
25.3 CX3CL1
25.3 CCL17
Elephant Scaffold 43
Mb
0.7 PLLP
0.7 CCL22
0.6 CX3CL1
0.6 CCL17
0.6 CIAPIN1
Opossum Chr 1
Mb
448.5 PLLP
448.6 CCL22
448.6 CX3CL1
448.7 CCL17a
448.7 CCL17b
448.7 CIAPIN1
Platypus Ultra 517
Mb
8.1 PLLP
8.1 CCL22
8.2 CCL17
8.3 ENSOANG00000009340
Wallaby Scaffold 29904
Kb
9 CCL22
Wallaby Scaffold 40468
Kb
9 CX3CL1
Human Chr 9
Mb
34.7 IL11RA
34.7 CCL27
34.7 CCL19
34.7 CCL21
34.7 RP11-195F19.10
Mouse Chr 4
Mb
41.6 IL11RA1
41.8 Ccl27a
41.8 Ccl21b
42.0 CCL21d
42.0 CCL27c
42.2 CCL19b
42.3 CCL27d
42.4 Ccl21c
42.6 CCL21e
42.7 Ccl27b
42.8 Ccl19a
42.8 Ccl21a
42.8 Gm12394
Cow Chr 8
Mb
80.2 Q3SZ24_BOVIN
80.2 CCL27
80.3 CCL19
80.3 CCL21
Elephant Scaffold 6
Mb
65.7 IL11RA
65.7 CCL27
65.7 CCL19
65.6 CCL21
65.6 ENSLAFG00000026815
Opossum Chr 6
Mb
124.1 IL11RA
124.1 CCL19
124.0 CCL21
124.0 ENSMODG00000024699
Platypus Contig 6869
Kb
35 IL11RA
45 CCL27
Platypus Ultra 8421
Kb
14 CCL21
Wallaby Scaffold 50948
Kb
2 CCL19
Human Chr 2
Mb
228.6 SLC19A3
228.7 CCL20
228.7 WDR69
Mouse Chr 1
Mb
83.0 SLC19A3
83.1 Ccl20
83.2 WDR69
Cow Chr 2
Mb
120.5 A7E352_BOVIN
120.6 CCL20
120.6 WDR69_BOVIN
Elephant Scaffold 55
Mb
11.1 SLC19A3
11.0 CCL20
10.9 WDR69
Opossum Chr 7
Mb
221.3 XM_001373428.1
221.1 CCL20
221.0 WDR69
Platypus Contig 7266
Kb
12 CCL20a
Platypus Ultra 477
Mb
0.5 CCL20b
Wallaby Scaffold 62606
Kb
12 CCL20
Human Chr 1
Mb
168.7 DPT
168.5 XCL1
168.5 XCL2
168.3 TBX19
166.7 DPT
166.9 Xcl1
167.1 TBX19
Cow Chr 16
Mb
33.7 DERM_BOVIN
33.5 XCL1
33.5 XCL2
Elephant Scaffold 33
Mb
26.1 DPT
26.3 XCL1
26.7 TBX19
Opossum Chr 2
Mb
60.8 DPT
60.6 XCL1
60.3 TBX19
Human Chr 19
Mb
8.0 ELAVL1
8.1 CCL25
8.1 FBN3
Mouse Chr 8
Mb
4.3 ELAVL1
4.3 Ccl25
Cow Chr 7
Mb
15.2 Q3ZCE2_BOVIN
15.2 CCL25
15.2 FBN3
Elephant Scaffold 114
Mb
2.1 ELAVL1
2.2 CCL25
2.2 FBN3
Opossum Chr 3
Mb
461.6 ELAVL1
461.5 FBN3
Wallaby Scaffold 30030
Kb
18 CCL25
Human Chr 5
Mb
43.3 HMGCS1
43.4 CCL28
43.4 C5orf28
Mouse Chr 13 (NT_166323.1)
Kb
95 HMGCS1
149 Ccl28
Cow Chr 20
Mb
33.4 Q3ZC79_BOVIN
33.4 CCL28
33.3 CE028_BOVIN
Elephant Scaffold 7
Mb
48.3 HMGCS1
48.4 CCL28
231.3 A6N2U5_MONDO
231.2 CCL28
231.1 C5orf28
Platypus Ultra 217
Kb
2 HMGCS1
Wallaby GeneScaffold 5978
Kb
0.5 CCL28

(b) Birds, lizard and frog

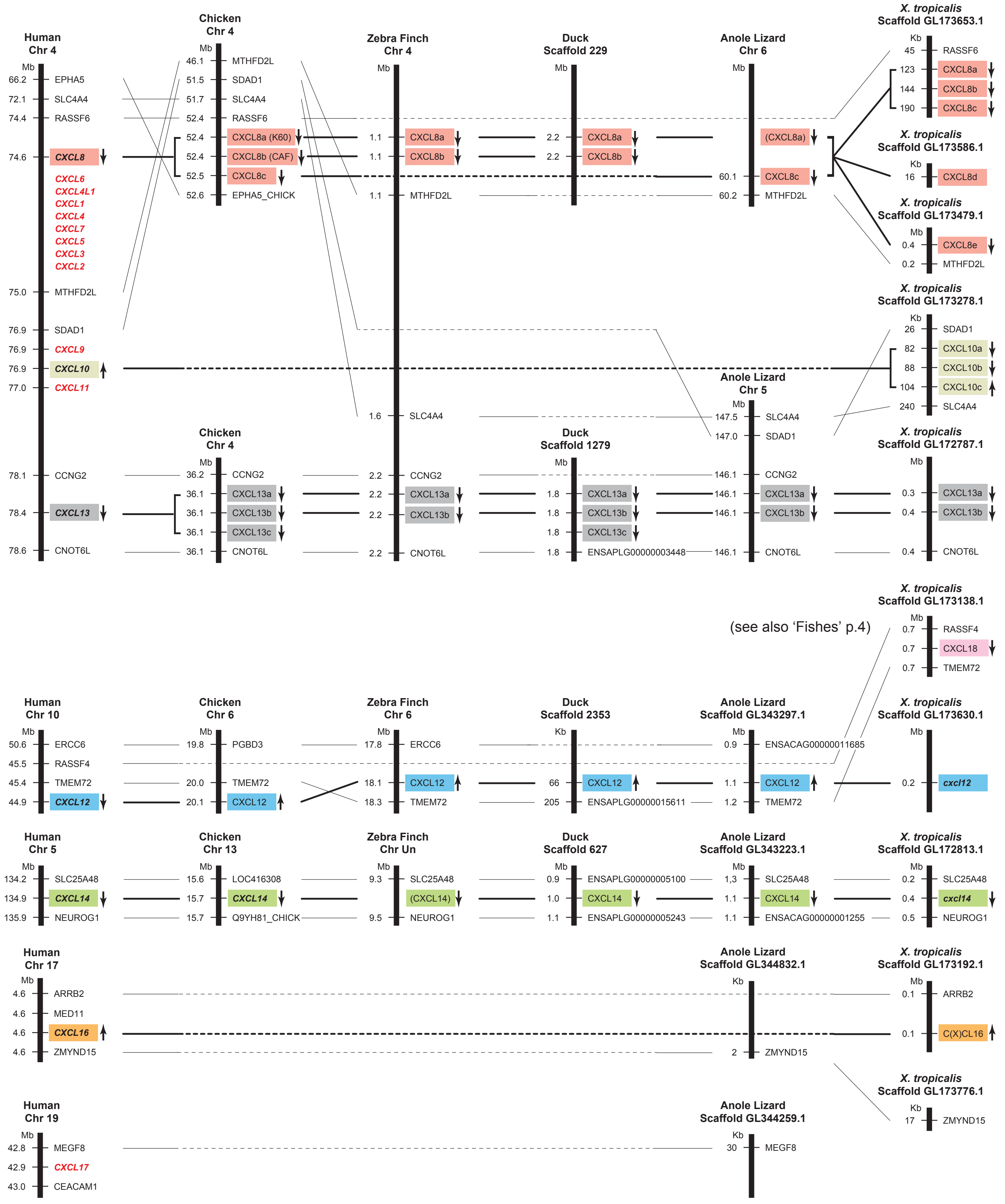

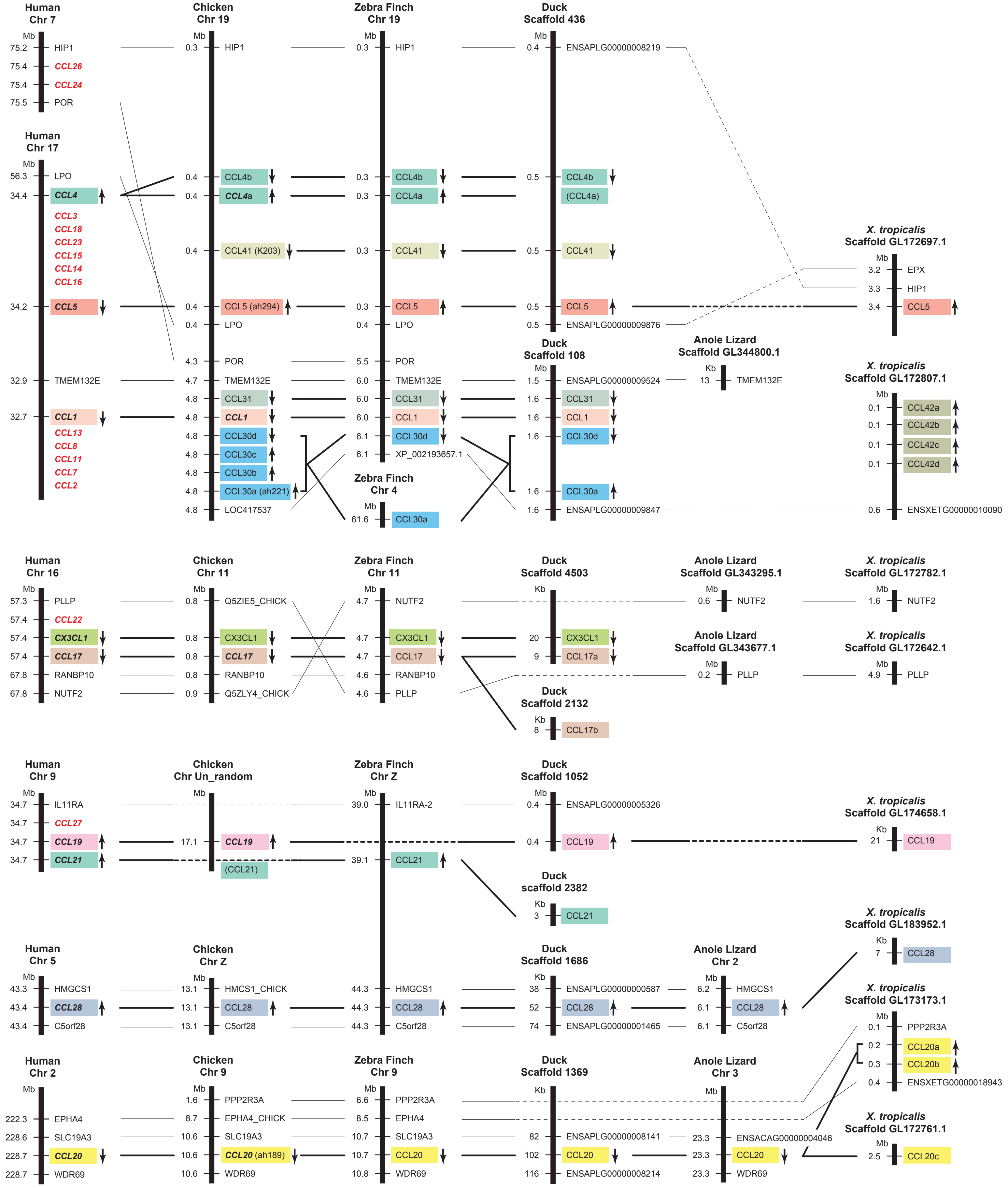

Human Chr 7
Mb
75.2 HIP1
75.4 CCL26
75.4 CCL24
75.5 POR
Human Chr 17
Mb
56.3 LPO
34.4 CCL4
CCL3
CCL18
CCL23
CCL15
CCL14
CCL16
34.2 CCL5
32.9 TMEM132E
32.7 CCL1
CCL13
CCL8
CCL11
CCL7
CCL2
Chicken Chr 19
Mb
0.3 HIP1
0.4 CCL4b
0.4 CCL4a
0.4 CCL41 (K203)
0.4 CCL5 (ah294)
0.4 LPO
4.3 POR
4.7 TMEM132E
4.8 CCL31
4.8 CCL1
4.8 CCL30d
4.8 CCL30c
4.8 CCL30b
4.8 CCL30a (ah221)
4.8 LOC417537
Zebra Finch Chr 19
Mb
0.3 HIP1
0.3 CCL4b
0.3 CCL4a
0.3 CCL41
0.3 CCL5
0.4 LPO
5.5 POR
6.0 TMEM132E
6.0 CCL31
6.0 CCL1
6.1 CCL30d
6.1 XP_002193657.1
Zebra Finch Chr 4
Mb
61.6 CCL30a
Duck Scaffold 436
Mb
0.4 ENSAPLG00000008219
0.5 CCL4b
(CCL4a)
0.5 CCL41
0.5 CCL5
0.5 ENSAPLG00000009876
Duck Scaffold 108
Mb
1.5 ENSAPLG00000009524
1.6 CCL31
1.6 CCL1
1.6 CCL30d
1.6 CCL30a
1.6 ENSAPLG00000009847
Anole Lizard Scaffold GL344800.1
Kb
13 TMEM132E
X. tropicalis Scaffold GL172697.1
Mb
3.2 EPX
3.3 HIP1
3.4 CCL5
X. tropicalis Scaffold GL172807.1
Mb
0.1 CCL42a
0.1 CCL42b
0.1 CCL42c
0.1 CCL42d
0.6 ENSXETG00000010090
Human Chr 16
Mb
57.3 PLLP
57.4 CCL22
57.4 CX3CL1
57.4 CCL17
67.8 RANBP10
67.8 NUTF2
Chicken Chr 11
Mb
0.8 Q5ZIE5_CHICK
0.8 CX3CL1
0.8 CCL17
0.8 RANBP10
0.9 Q5ZLY4_CHICK
Zebra Finch Chr 11
Mb
4.7 NUTF2
4.7 CX3CL1
4.7 CCL17
4.6 RANBP10
4.6 PLLP
Duck Scaffold 4503
Kb
20 CX3CL1
9 CCL17a
Duck Scaffold 2132
Kb
8 CCL17b
Anole Lizard Scaffold GL343295.1
Mb
0.6 NUTF2
Anole Lizard Scaffold GL343677.1
Mb
0.2 PLLP
X. tropicalis Scaffold GL172782.1
Mb
1.6 NUTF2
X. tropicalis Scaffold GL172642.1
Mb
4.9 PLLP
Human Chr 9
Mb
34.7 IL11RA
34.7 CCL27
34.7 CCL19
34.7 CCL21
Chicken Chr Un_random
Mb
17.1 CCL19
(CCL21)
Zebra Finch Chr Z
Mb
39.0 IL11RA-2
39.1 CCL21
Duck Scaffold 1052
Mb
0.4 ENSAPLG00000005326
0.4 CCL19
Duck scaffold 2382
Kb
3 CCL21
X. tropicalis Scaffold GL174658.1
Kb
21 CCL19
Human Chr 5
Mb
43.3 HMGCS1
43.4 CCL28
43.4 C5orf28
Chicken Chr Z
Mb
13.1 HMCS1_CHICK
13.1 CCL28
13.1 C5orf28
44.3 HMGCS1
44.3 CCL28
44.3 C5orf28
Duck Scaffold 1686
Kb
38 ENSAPLG00000000587
52 CCL28
74 ENSAPLG00000001465
Anole Lizard Chr 2
Mb
6.2 HMGCS1
6.1 CCL28
6.1 C5orf28
X. tropicalis Scaffold GL183952.1
Kb
7 CCL28
X. tropicalis Scaffold GL173173.1
Mb
0.1 PPP2R3A
0.2 CCL20a
0.3 CCL20b
0.4 ENSXETG00000018943
Human Chr 2
Mb
222.3 EPHA4
228.6 SLC19A3
228.7 CCL20
228.7 WDR69
Chicken Chr 9
Mb
1.6 PPP2R3A
8.7 EPHA4_CHICK
10.6 SLC19A3
10.6 CCL20 (ah189)
10.6 WDR69
Zebra Finch Chr 9
Mb
6.6 PPP2R3A
8.5 EPHA4
10.7 SLC19A3
10.7 CCL20
10.8 WDR69
Duck Scaffold 1369
Kb
82 ENSAPLG00000008141
102 CCL20
116 ENSAPLG00000008214
Anole Lizard Chr 3
Mb
23.3 ENSACAG00000004046
23.3 CCL20
23.3 WDR69
X. tropicalis Scaffold GL172761.1
Mb
2.5 CCL20c

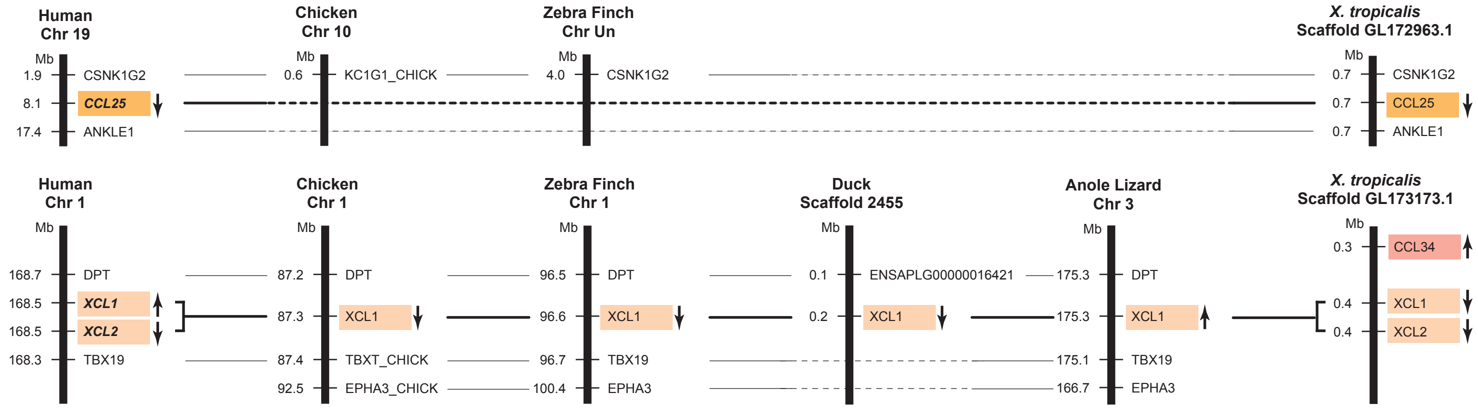

(see also (c) Fish p.9)

(c) Fish

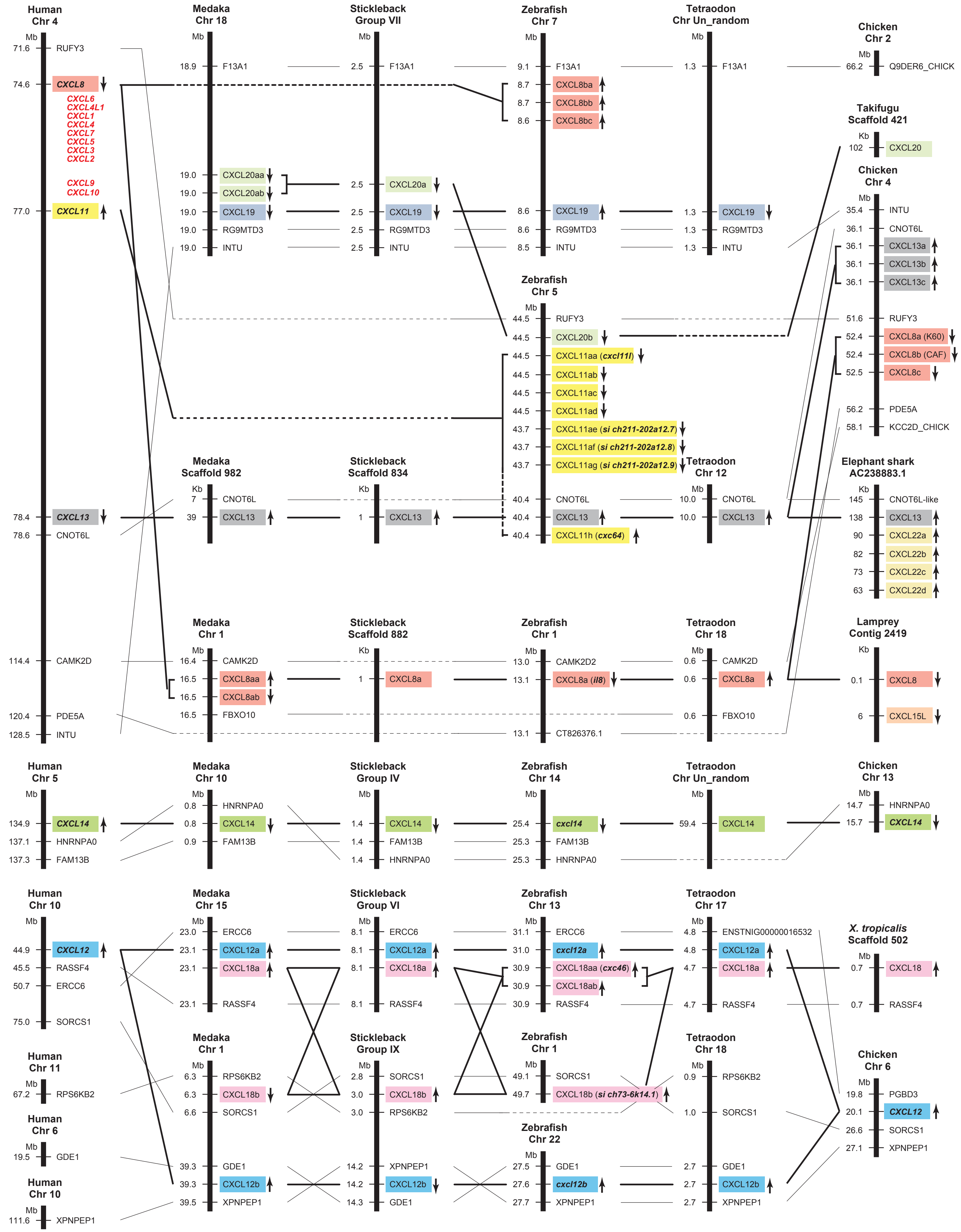
Human Chr 4
Mb
71.6 RUFY3
74.6 CXCL8
CXCL6
CXCL4L1
CXCL1
CXCL4
CXCL7
CXCL5
CXCL3
CXCL2
CXCL9
CXCL10
77.0 CXCL11
78.4 CXCL13
78.6 CNOT6L
114.4 CAMK2D
120.4 PDE5A
128.5 INTU
Medaka Chr 18
Mb
18.9 F13A1
19.0 CXCL20aa
19.0 CXCL20ab
19.0 CXCL19
19.0 RG9MTD3
19.0 INTU
Stickleback Group VII
Mb
2.5 F13A1
2.5 CXCL20a
2.5 CXCL19
2.5 RG9MTD3
2.5 INTU
Zebrafish Chr 7
Mb
9.1 F13A1
8.7 CXCL8ba
8.7 CXCL8bb
8.6 CXCL8bc
8.6 CXCL19
8.6 RG9MTD3
8.5 INTU
Tetraodon Chr Un_random
Mb
1.3 F13A1
1.3 CXCL19
1.3 RG9MTD3
1.3 INTU
Chicken Chr 2
Mb
66.2 Q9DER6_CHICK
Takifugu Scaffold 421
Kb
102 CXCL20
Chicken Chr 4
Mb
35.4 INTU
36.1 CNOT6L
36.1 CXCL13a
36.1 CXCL13b
36.1 CXCL13c
51.6 RUFY3
52.4 CXCL8a (K60)
52.4 CXCL8b (CAF)
52.5 CXCL8c
56.2 PDE5A
58.1 KCC2D_CHICK
Zebrafish Chr 5
Mb
44.5 RUFY3
44.5 CXCL20b
44.5 CXCL11aa (cxcl11l)
44.5 CXCL11ab
44.5 CXCL11ac
44.5 CXCL11ad
43.7 CXCL11ae (si ch211-202a12.7)
43.7 CXCL11af (si ch211-202a12.8)
43.7 CXCL11ag (si ch211-202a12.9)
40.4 CNOT6L
40.4 CXCL13
40.4 CXCL11h (cxc64)
Medaka Scaffold 982
Kb
7 CNOT6L
39 CXCL13
Stickleback Scaffold 834
Kb
1 CXCL13
Tetraodon Chr 12
Mb
10.0 CNOT6L
10.0 CXCL13
Elephant shark AC238883.1
Kb
145 CNOT6L-like
138 CXCL13
90 CXCL22a
82 CXCL22b
73 CXCL22c
63 CXCL22d
Medaka Chr 1
Mb
16.4 CAMK2D
16.5 CXCL8aa
16.5 CXCL8ab
16.5 FBXO10
Stickleback Scaffold 882
Kb
1 CXCL8a
Zebrafish Chr 1
Mb
13.0 CAMK2D2
13.1 CXCL8a (il8)
13.1 CT826376.1
Tetraodon Chr 18
Mb
0.6 CAMK2D
0.6 CXCL8a
0.6 FBXO10
Lamprey Contig 2419
Kb
0.1 CXCL8
6 CXCL15L
Human Chr 5
Mb
134.9 CXCL14
137.1 HNRNPA0
137.3 FAM13B
Medaka Chr 10
Mb
0.8 HNRNPA0
0.8 CXCL14
0.9 FAM13B
Stickleback Group IV
Mb
1.4 CXCL14
1.4 FAM13B
1.4 HNRNPA0
Zebrafish Chr 14
Mb
25.4 cxcl14
25.3 FAM13B
25.3 HNRNPA0
Tetraodon Chr Un_random
Mb
59.4 CXCL14
Chicken Chr 13
Mb
14.7 HNRNPA0
15.7 CXCL14
Human Chr 10
Mb
44.9 CXCL12
45.5 RASSF4
50.7 ERCC6
75.0 SORCS1
Human Chr 11
Mb
67.2 RPS6KB2
Human Chr 6
Mb
19.5 GDE1
Human Chr 10
Mb
111.6 XPNPEP1
Medaka Chr 15
Mb
23.0 ERCC6
23.1 CXCL12a
23.1 CXCL18a
23.1 RASSF4
Stickleback Group VI
Mb
8.1 ERCC6
8.1 CXCL12a
8.1 CXCL18a
8.1 RASSF4
Zebrafish Chr 13
Mb
31.1 ERCC6
31.0 cxcl12a
30.9 CXCL18aa (cxc46)
30.9 CXCL18ab
30.9 RASSF4
Tetraodon Chr 17
Mb
4.8 ENSTNIG00000016532
4.8 CXCL12a
4.7 CXCL18a
4.7 RASSF4
X. tropicalis Scaffold 502
Mb
0.7 CXCL18
0.7 RASSF4
Medaka Chr 1
Mb
6.3 RPS6KB2
6.3 CXCL18b
6.6 SORCS1
39.3 GDE1
39.3 CXCL12b
39.5 XPNPEP1
Stickleback Group IX
Mb
2.8 SORCS1
3.0 CXCL18b
3.0 RPS6KB2
14.2 XPNPEP1
14.2 CXCL12b
14.3 GDE1
Zebrafish Chr 1
Mb
49.1 SORCS1
49.7 CXCL18b (si ch73-6k14.1)
Zebrafish Chr 22
Mb
27.5 GDE1
27.6 cxcl12b
27.7 XPNPEP1
Tetraodon Chr 18
Mb
0.9 RPS6KB2
1.0 SORCS1
2.7 GDE1
2.7 CXCL12b
2.7 XPNPEP1
Chicken Chr 6
Mb
19.8 PGBD3
20.1 CXCL12
26.6 SORCS1
27.1 XPNPEP1

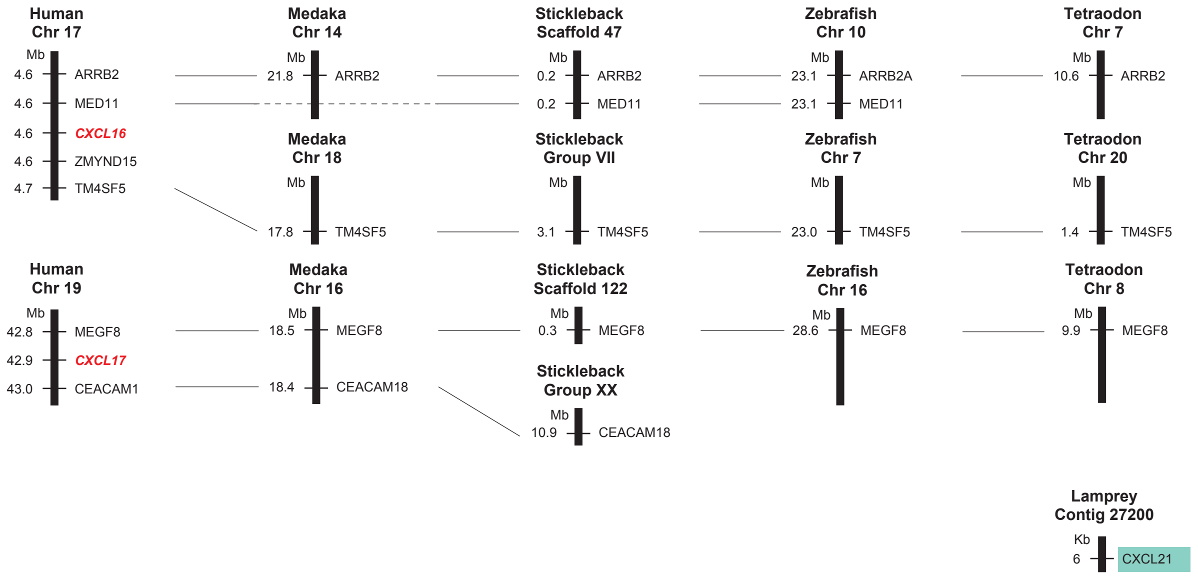

Human
Chr 17
Mb
4.6 ARRB2
4.6 MED11
4.6 CXCL16
4.6 ZMYND15
4.7 TM4SF5
Medaka
Chr 14
Mb
21.8 ARRB2
Medaka
Chr 18
Mb
17.8 TM4SF5
Stickleback
Scaffold 47
Mb
0.2 ARRB2
0.2 MED11
Stickleback
Group VII
Mb
3.1 TM4SF5
Zebrafish
Chr 10
Mb
23.1 ARRB2A
23.1 MED11
Zebrafish
Chr 7
Mb
23.0 TM4SF5
Tetraodon
Chr 7
Mb
10.6 ARRB2
Tetraodon
Chr 20
Mb
1.4 TM4SF5
Human
Chr 19
Mb
42.8 MEGF8
42.9 CXCL17
43.0 CEACAM1
Medaka
Chr 16
Mb
18.5 MEGF8
18.4 CEACAM18
Stickleback
Scaffold 122
Mb
0.3 MEGF8
Stickleback
Group XX
Mb
10.9 CEACAM18
Zebrafish
Chr 16
Mb
28.6 MEGF8
Tetraodon
Chr 8
Mb
9.9 MEGF8
Lamprey
Contig 27200
Kb
6 CXCL21

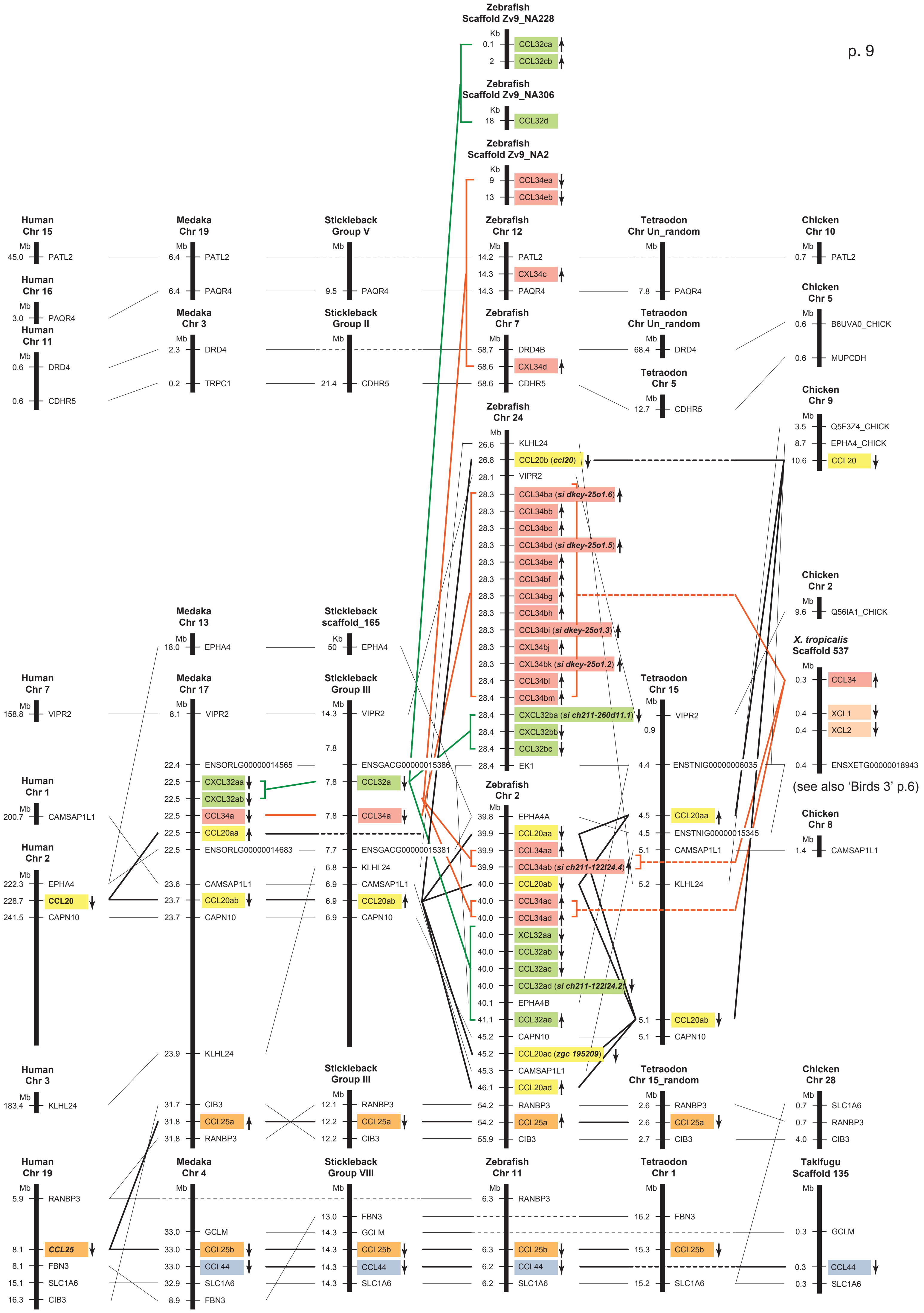
Zebrafish Scaffold Zv9_NA228
Kb
0.1 CCL32ca
2 CCL32cb
Zebrafish Scaffold Zv9_NA306
Kb
18 CCL32d
Zebrafish Scaffold Zv9_NA2
Kb
9 CCL34ea
13 CCL34eb
Human Chr 15
Mb
45.0 PATL2
Human Chr 16
Mb
3.0 PAQR4
Human Chr 11
Mb
0.6 DRD4
0.6 CDHR5
Medaka Chr 19
Mb
6.4 PATL2
6.4 PAQR4
Medaka Chr 3
Mb
2.3 DRD4
0.2 TRPC1
Stickleback Group V
Mb
9.5 PAQR4
Stickleback Group II
Mb
21.4 CDHR5
Zebrafish Chr 12
Mb
14.2 PATL2
14.3 CXL34c
14.3 PAQR4
Zebrafish Chr 7
Mb
58.7 DRD4B
58.6 CXL34d
58.6 CDHR5
Tetraodon Chr Un_random
Mb
7.8 PAQR4
Tetraodon Chr Un_random
Mb
68.4 DRD4
Tetraodon Chr 5
Mb
12.7 CDHR5
Chicken Chr 10
Mb
0.7 PATL2
Chicken Chr 5
Mb
0.6 B6UVA0_CHICK
0.6 MUPCDH
Chicken Chr 9
Mb
3.5 Q5F3Z4_CHICK
8.7 EPHA4_CHICK
10.6 CCL20
Zebrafish Chr 24
Mb
26.6 KLHL24
26.8 CCL20b (ccl20)
28.1 VIPR2
28.3 CCL34ba (si dkey-25o1.6)
28.3 CCL34bb
28.3 CCL34bc
28.3 CCL34bd (si dkey-25o1.5)
28.3 CCL34be
28.3 CCL34bf
28.3 CCL34bg
28.3 CCL34bh
28.3 CCL34bi (si dkey-25o1.3)
28.3 CXL34bj
28.3 CXL34bk (si dkey-25o1.2)
28.4 CCL34bl
28.4 CCL34bm
28.4 CXCL32ba (si ch211-260d11.1)
28.4 CXCL32bb
28.4 CCL32bc
28.4 EK1
Chicken Chr 2
Mb
9.6 Q56IA1_CHICK
X. tropicalis Scaffold 537
Mb
0.3 CCL34
0.4 XCL1
0.4 XCL2
0.4 ENSXETG00000018943
(see also 'Birds 3' p.6)
Medaka Chr 13
Mb
18.0 EPHA4
Stickleback scaffold_165
Kb
50 EPHA4
Human Chr 7
Mb
158.8 VIPR2
Medaka Chr 17
Mb
8.1 VIPR2
22.4 ENSORLG00000014565
22.5 CXCL32aa
22.5 CXCL32ab
22.5 CCL34a
22.5 CCL20aa
22.5 ENSORLG00000014683
23.6 CAMSAP1L1
23.7 CCL20ab
23.7 CAPN10
23.9 KLHL24
31.7 CIB3
31.8 CCL25a
31.8 RANBP3
Stickleback Group III
Mb
14.3 VIPR2
7.8
ENSGACG00000015386
7.8 CCL32a
7.8 CCL34a
7.7 ENSGACG00000015381
6.8 KLHL24
6.9 CAMSAP1L1
6.9 CCL20ab
6.9 CAPN10
Human Chr 1
Mb
200.7 CAMSAP1L1
Human Chr 2
Mb
222.3 EPHA4
228.7 CCL20
241.5 CAPN10
Tetraodon Chr 15
Mb
VIPR2
0.9
4.4 ENSTNIG00000006035
4.5 CCL20aa
4.5 ENSTNIG00000015345
5.1 CAMSAP1L1
5.2 KLHL24
5.1 CCL20ab
5.1 CAPN10
Chicken Chr 8
Mb
1.4 CAMSAP1L1
Zebrafish Chr 2
Mb
39.8 EPHA4A
39.9 CCL20aa
39.9 CCL34aa
39.9 CCL34ab (si ch211-122l24.4)
40.0 CCL20ab
40.0 CCL34ac
40.0 CCL34ad
40.0 XCL32aa
40.0 CCL32ab
40.0 CCL32ac
40.0 CCL32ad (si ch211-122l24.2)
40.1 EPHA4B
41.1 CCL32ae
45.2 CAPN10
45.2 CCL20ac (zgc 195209)
45.3 CAMSAP1L1
46.1 CCL20ad
Human Chr 3
Mb
183.4 KLHL24
Stickleback Group III
Mb
12.1 RANBP3
12.2 CCL25a
12.2 CIB3
54.2 RANBP3
54.2 CCL25a
55.9 CIB3
Tetraodon Chr 15_random
Mb
2.6 RANBP3
2.6 CCL25a
2.7 CIB3
Chicken Chr 28
Mb
0.7 SLC1A6
0.7 RANBP3
4.0 CIB3
Human Chr 19
Mb
5.9 RANBP3
8.1 CCL25
8.1 FBN3
15.1 SLC1A6
16.3 CIB3
Medaka Chr 4
Mb
33.0 GCLM
33.0 CCL25b
33.0 CCL44
32.9 SLC1A6
8.9 FBN3
Stickleback Group VIII
Mb
13.0 FBN3
14.3 GCLM
14.3 CCL25b
14.3 CCL44
14.3 SLC1A6
Zebrafish Chr 11
Mb
6.3 RANBP3
6.3 CCL25b
6.2 CCL44
6.2 SLC1A6
Tetraodon Chr 1
Mb
16.2 FBN3
15.3 CCL25b
15.2 SLC1A6
Takifugu Scaffold 135
Mb
0.3 GCLM
0.3 CCL44
0.3 SLC1A6

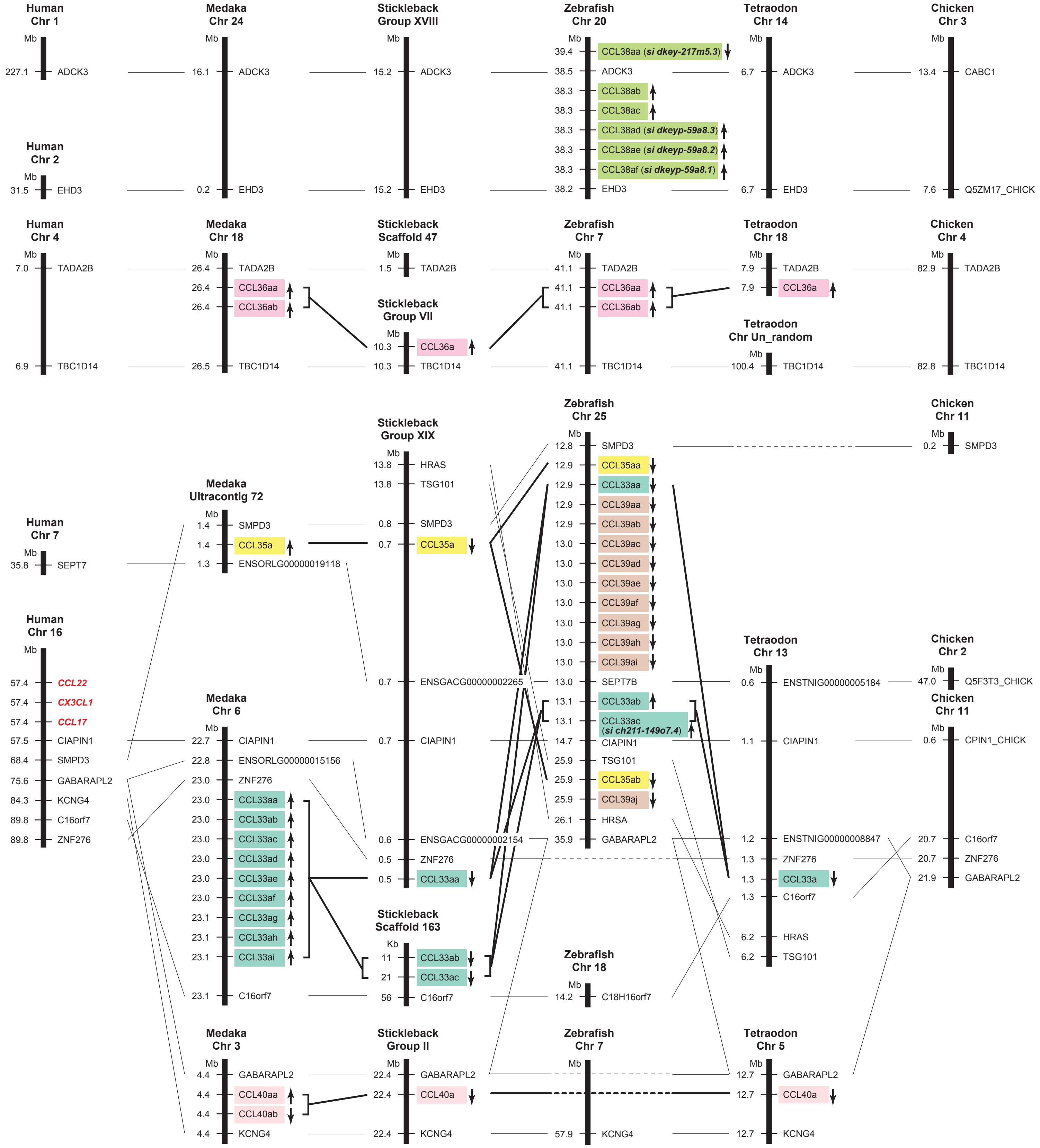

Human Chr 1
Mb
227.1 ADCK3
Human Chr 2
31.5 EHD3
Medaka Chr 24
16.1 ADCK3
0.2 EHD3
Stickleback Group XVIII
15.2 ADCK3
15.2 EHD3
Zebrafish Chr 20
39.4 CCL38aa (si dkey-217m5.3)
38.5 ADCK3
38.3 CCL38ab
38.3 CCL38ac
38.3 CCL38ad (si dkeyp-59a8.3)
38.3 CCL38ae (si dkeyp-59a8.2)
38.3 CCL38af (si dkeyp-59a8.1)
38.2 EHD3
Tetraodon Chr 14
6.7 ADCK3
6.7 EHD3
Chicken Chr 3
13.4 CABC1
7.6 Q5ZM17_CHICK
Human Chr 4
7.0 TADA2B
6.9 TBC1D14
Medaka Chr 18
26.4 TADA2B
26.4 CCL36aa
26.4 CCL36ab
26.5 TBC1D14
Stickleback Scaffold 47
1.5 TADA2B
Stickleback Group VII
10.3 CCL36a
10.3 TBC1D14
Zebrafish Chr 7
41.1 TADA2B
41.1 CCL36aa
41.1 CCL36ab
41.1 TBC1D14
Tetraodon Chr 18
7.9 TADA2B
7.9 CCL36a
Tetraodon Chr Un_random
100.4 TBC1D14
Chicken Chr 4
82.9 TADA2B
82.8 TBC1D14
Human Chr 7
35.8 SEPT7
Human Chr 16
57.4 CCL22
57.4 CX3CL1
57.4 CCL17
57.5 CIAPIN1
68.4 SMPD3
75.6 GABARAPL2
84.3 KCNG4
89.8 C16orf7
89.8 ZNF276
Medaka Ultracontig 72
1.4 SMPD3
1.4 CCL35a
1.3 ENSORLG00000019118
Medaka Chr 6
22.7 CIAPIN1
22.8 ENSORLG00000015156
23.0 ZNF276
23.0 CCL33aa
23.0 CCL33ab
23.0 CCL33ac
23.0 CCL33ad
23.0 CCL33ae
23.0 CCL33af
23.1 CCL33ag
23.1 CCL33ah
23.1 CCL33ai
23.1 C16orf7
Medaka Chr 3
4.4 GABARAPL2
4.4 CCL40aa
4.4 CCL40ab
4.4 KCNG4
Stickleback Group XIX
13.8 HRAS
13.8 TSG101
0.8 SMPD3
0.7 CCL35a
0.7 ENSGACG00000002265
0.7 CIAPIN1
0.6 ENSGACG00000002154
0.5 ZNF276
0.5 CCL33aa
Stickleback Scaffold 163
Kb
11 CCL33ab
21 CCL33ac
56 C16orf7
Stickleback Group II
22.4 GABARAPL2
22.4 CCL40a
22.4 KCNG4
Zebrafish Chr 25
12.8 SMPD3
12.9 CCL35aa
12.9 CCL33aa
12.9 CCL39aa
12.9 CCL39ab
13.0 CCL39ac
13.0 CCL39ad
13.0 CCL39ae
13.0 CCL39af
13.0 CCL39ag
13.0 CCL39ah
13.0 CCL39ai
13.0 SEPT7B
13.1 CCL33ab
13.1 CCL33ac (si ch211-149o7.4)
14.7 CIAPIN1
25.9 TSG101
25.9 CCL35ab
25.9 CCL39aj
26.1 HRSA
35.9 GABARAPL2
Zebrafish Chr 18
14.2 C18H16orf7
Zebrafish Chr 7
57.9 KCNG4
Tetraodon Chr 13
0.6 ENSTNIG00000005184
1.1 CIAPIN1
1.2 ENSTNIG00000008847
1.3 ZNF276
1.3 CCL33a
1.3 C16orf7
6.2 HRAS
6.2 TSG101
Tetraodon Chr 5
12.7 GABARAPL2
12.7 CCL40a
12.7 KCNG4
Chicken Chr 11
0.2 SMPD3
Chicken Chr 2
47.0 Q5F3T3_CHICK
Chicken Chr 11
0.6 CPIN1_CHICK
20.7 C16orf7
20.7 ZNF276
21.9 GABARAPL2

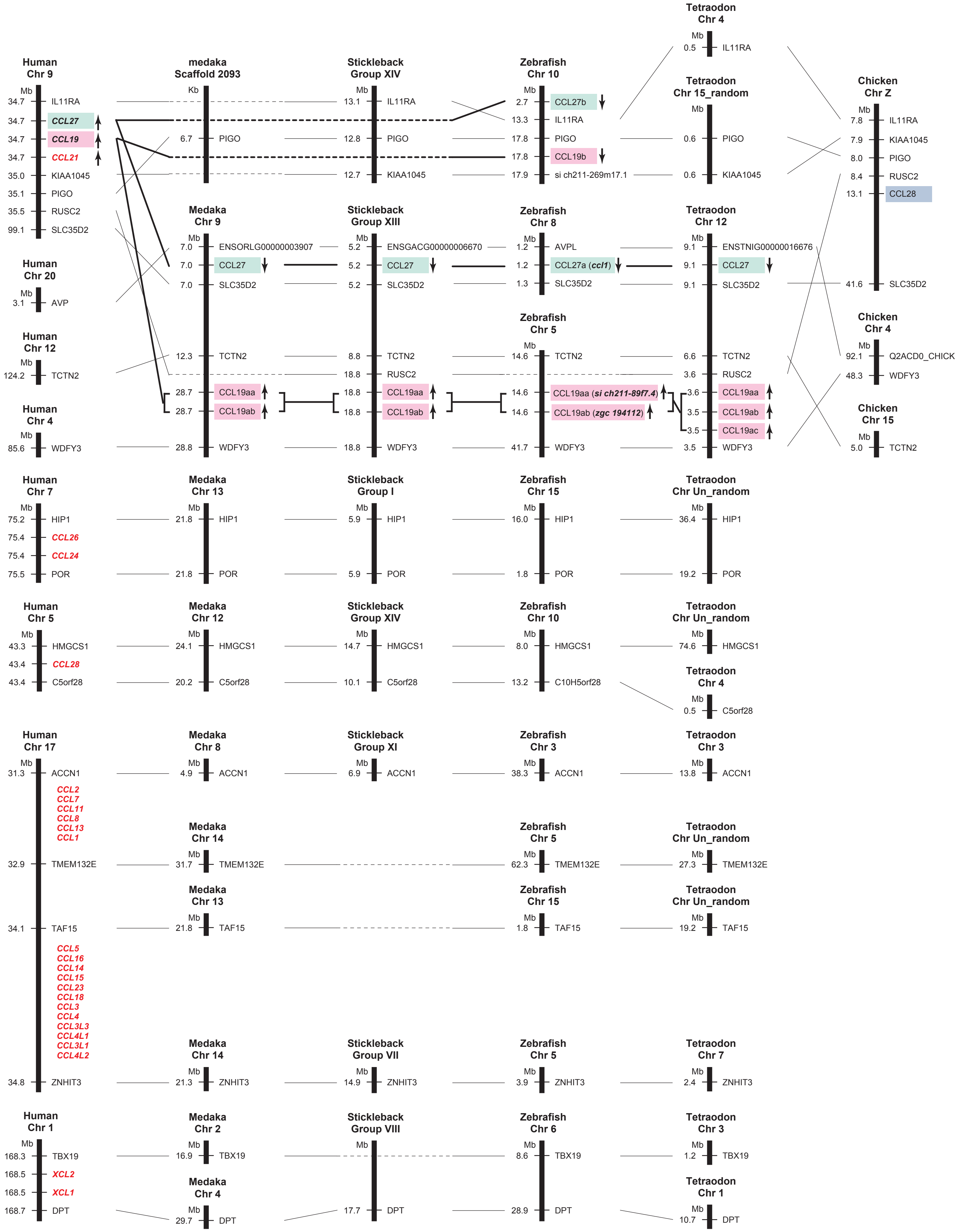

Human Chr 9
Mb
34.7 IL11RA
34.7 CCL27
34.7 CCL19
34.7 CCL21
35.0 KIAA1045
35.1 PIGO
35.5 RUSC2
99.1 SLC35D2
Human Chr 20
Mb
3.1 AVP
Human Chr 12
Mb
124.2 TCTN2
Human Chr 4
Mb
85.6 WDFY3
medaka Scaffold 2093
Kb
6.7 PIGO
Stickleback Group XIV
Mb
13.1 IL11RA
12.8 PIGO
12.7 KIAA1045
Zebrafish Chr 10
Mb
2.7 CCL27b
13.3 IL11RA
17.8 PIGO
17.8 CCL19b
17.9 si ch211-269m17.1
Tetraodon Chr 4
Mb
0.5 IL11RA
Tetraodon Chr 15_random
Mb
0.6 PIGO
0.6 KIAA1045
Chicken Chr Z
Mb
7.8 IL11RA
7.9 KIAA1045
8.0 PIGO
8.4 RUSC2
13.1 CCL28
41.6 SLC35D2
Medaka Chr 9
Mb
7.0 ENSORLG00000003907
7.0 CCL27
7.0 SLC35D2
12.3 TCTN2
28.7 CCL19aa
28.7 CCL19ab
28.8 WDFY3
Stickleback Group XIII
Mb
5.2 ENSGACG00000006670
5.2 CCL27
5.2 SLC35D2
8.8 TCTN2
18.8 RUSC2
18.8 CCL19aa
18.8 CCL19ab
18.8 WDFY3
Zebrafish Chr 8
Mb
1.2 AVPL
1.2 CCL27a (ccl1)
1.3 SLC35D2
Zebrafish Chr 5
Mb
14.6 TCTN2
14.6 CCL19aa (si ch211-89f7.4)
14.6 CCL19ab (zgc 194112)
41.7 WDFY3
Tetraodon Chr 12
Mb
9.1 ENSTNIG00000016676
9.1 CCL27
9.1 SLC35D2
6.6 TCTN2
3.6 RUSC2
3.6 CCL19aa
3.5 CCL19ab
3.5 CCL19ac
3.5 WDFY3
Chicken Chr 4
Mb
92.1 Q2ACD0_CHICK
48.3 WDFY3
Chicken Chr 15
Mb
5.0 TCTN2
Human Chr 7
Mb
75.2 HIP1
75.4 CCL26
75.4 CCL24
75.5 POR
Medaka Chr 13
Mb
21.8 HIP1
21.8 POR
Stickleback Group I
Mb
5.9 HIP1
5.9 POR
Zebrafish Chr 15
Mb
16.0 HIP1
1.8 POR
Tetraodon Chr Un_random
Mb
36.4 HIP1
19.2 POR
Human Chr 5
Mb
43.3 HMGCS1
43.4 CCL28
43.4 C5orf28
Medaka Chr 12
Mb
24.1 HMGCS1
20.2 C5orf28
Stickleback Group XIV
Mb
14.7 HMGCS1
10.1 C5orf28
Zebrafish Chr 10
Mb
8.0 HMGCS1
13.2 C10H5orf28
Tetraodon Chr Un_random
Mb
74.6 HMGCS1
Tetraodon Chr 4
Mb
0.5 C5orf28
Human Chr 17
Mb
31.3 ACCN1
CCL2
CCL7
CCL11
CCL8
CCL13
CCL1
32.9 TMEM132E
34.1 TAF15
CCL5
CCL16
CCL14
CCL15
CCL23
CCL18
CCL3
CCL4
CCL3L3
CCL4L1
CCL3L1
CCL4L2
34.8 ZNHIT3
Medaka Chr 8
Mb
4.9 ACCN1
Stickleback Group XI
Mb
6.9 ACCN1
Zebrafish Chr 3
Mb
38.3 ACCN1
Tetraodon Chr 3
Mb
13.8 ACCN1
Medaka Chr 14
Mb
31.7 TMEM132E
Zebrafish Chr 5
Mb
62.3 TMEM132E
Tetraodon Chr Un_random
Mb
27.3 TMEM132E
Medaka Chr 13
Mb
21.8 TAF15
Zebrafish Chr 15
Mb
1.8 TAF15
Tetraodon Chr Un_random
Mb
19.2 TAF15
Medaka Chr 14
Mb
21.3 ZNHIT3
Stickleback Group VII
Mb
14.9 ZNHIT3
Zebrafish Chr 5
Mb
3.9 ZNHIT3
Tetraodon Chr 7
Mb
2.4 ZNHIT3
Human Chr 1
Mb
168.3 TBX19
168.5 XCL2
168.5 XCL1
168.7 DPT
Medaka Chr 2
Mb
16.9 TBX19
Medaka Chr 4
Mb
29.7 DPT
Stickleback Group VIII
Mb
17.7 DPT
Zebrafish Chr 6
Mb
8.6 TBX19
28.9 DPT
Tetraodon Chr 3
Mb
1.2 TBX19
Tetraodon Chr 1
Mb
10.7 DPT

B. Chemokine receptors.

(a) Mammals

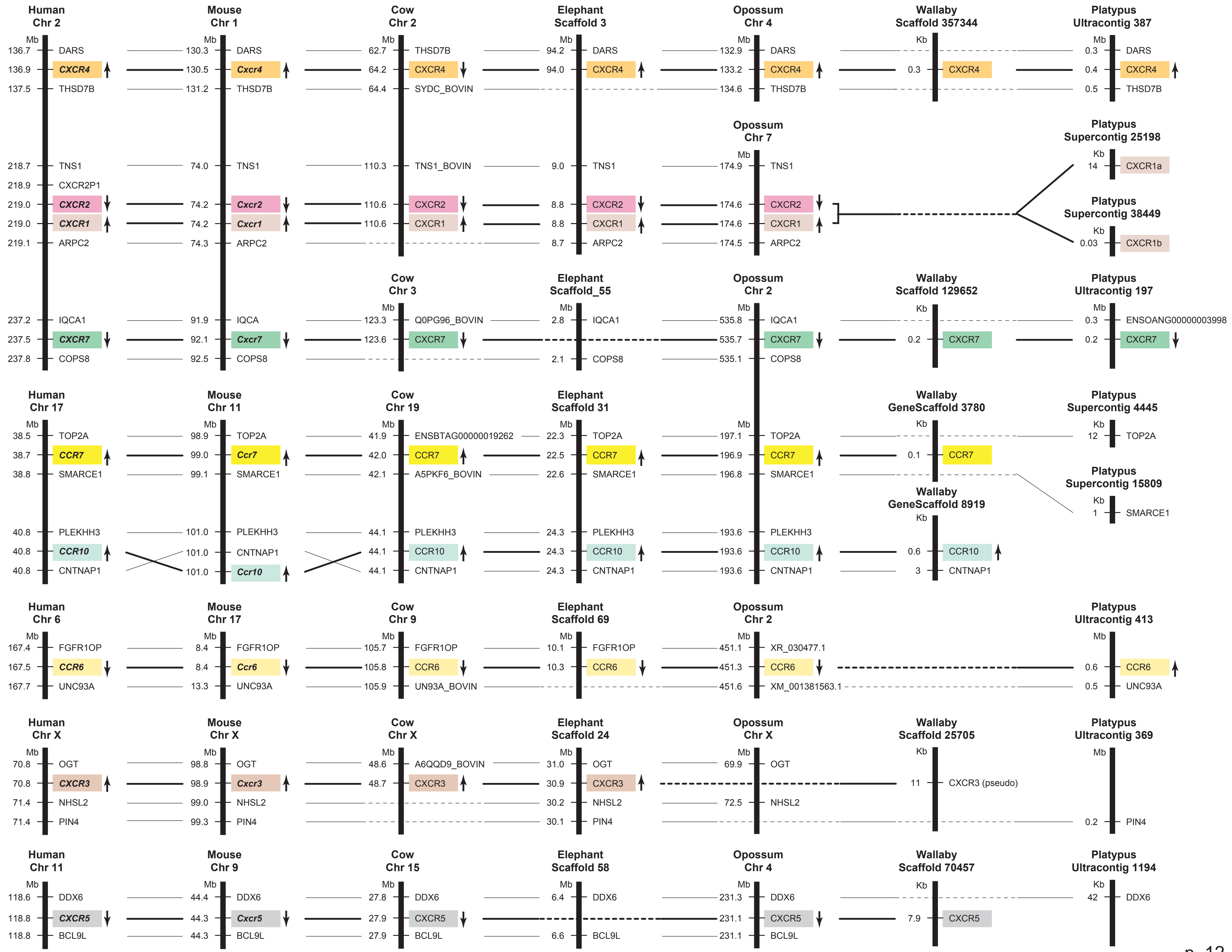

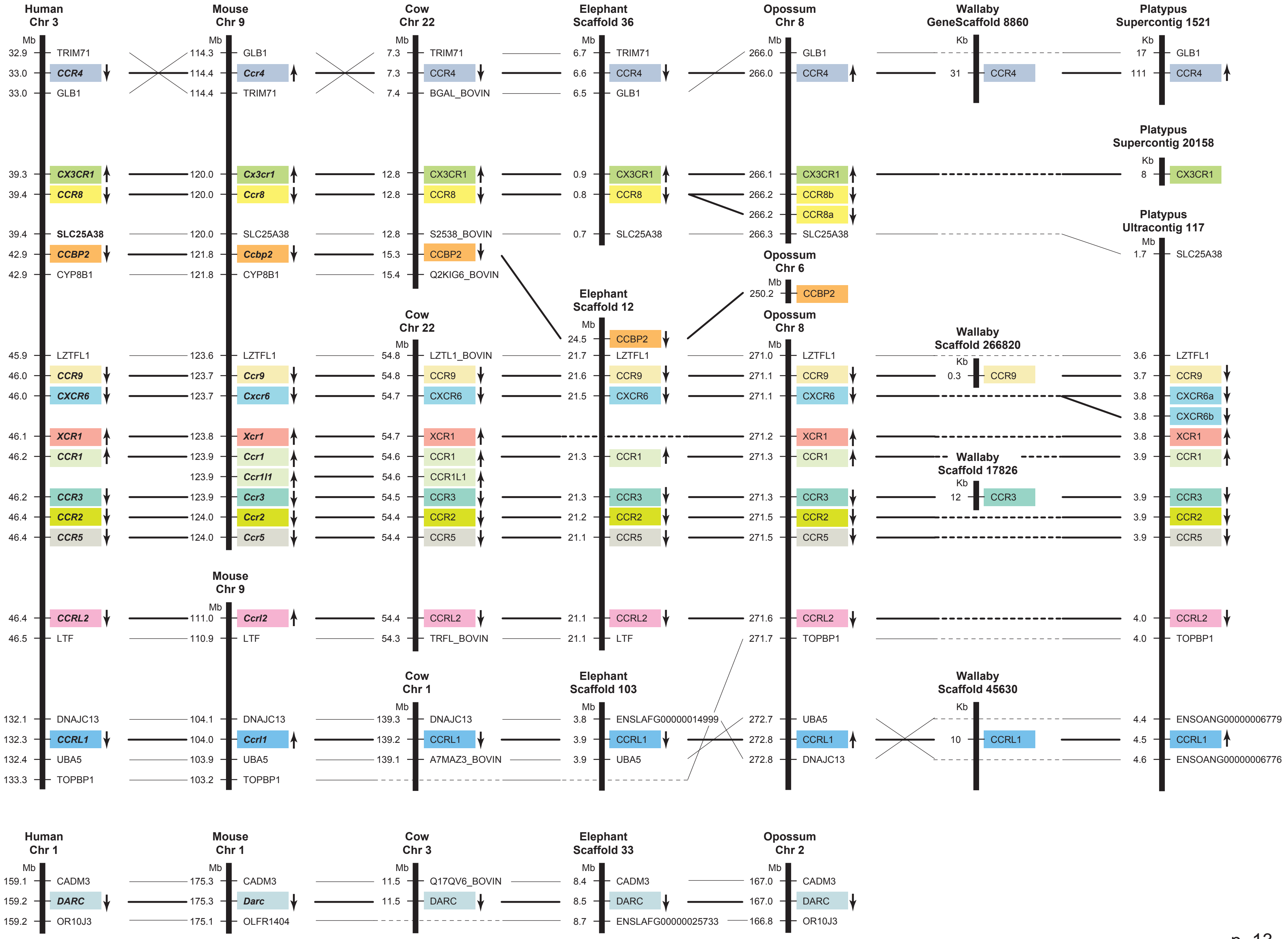

Human Chr 3
Mouse Chr 9
Cow Chr 22
Elephant Scaffold 36
Opossum Chr 8
Wallaby GeneScaffold 8860
Platypus Supercontig 1521
Mb
Kb
TRIM71
CCR4
GLB1
Platypus Supercontig 20158
CX3CR1
CCR8
CCR8b
CCR8a
SLC25A38
Platypus Ultracontig 117
CCBP2
CYP8B1
BGAL_BOVIN
S2538_BOVIN
Q2KIG6_BOVIN
Opossum Chr 6
Elephant Scaffold 12
Cow Chr 22
Opossum Chr 8
Wallaby Scaffold 266820
LZTFL1
LZTL1_BOVIN
CCR9
CXCR6
CXCR6a
CXCR6b
XCR1
CCR1
Ccr1l1
CCR1L1
Wallaby Scaffold 17826
CCR3
CCR2
CCR5
Mouse Chr 9
CCRL2
LTF
TRFL_BOVIN
TOPBP1
Cow Chr 1
Elephant Scaffold 103
Wallaby Scaffold 45630
DNAJC13
ENSLAFG00000014999
ENSOANG00000006779
CCRL1
UBA5
A7MAZ3_BOVIN
ENSOANG00000006776
Human Chr 1
Mouse Chr 1
Cow Chr 3
Elephant Scaffold 33
Opossum Chr 2
CADM3
Q17QV6_BOVIN
DARC
OR10J3
OLFR1404
ENSLAFG00000025733

(b) Birds, lizard and frog

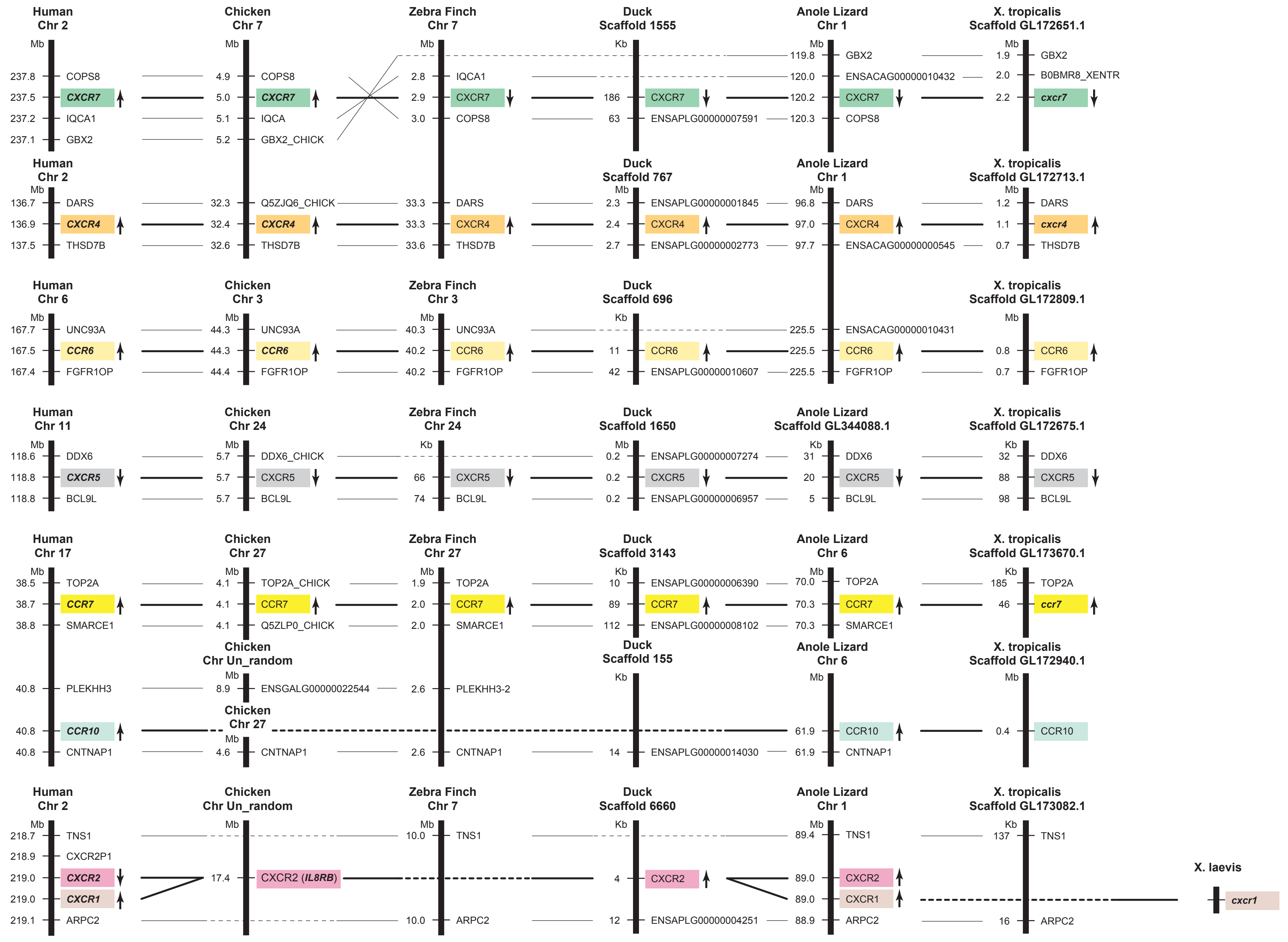

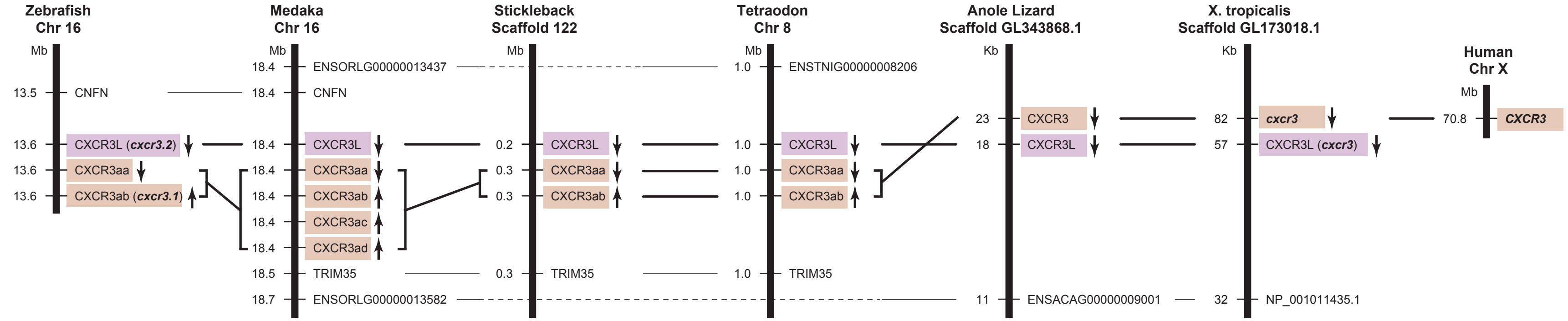

(see also (c) Fish p.18)

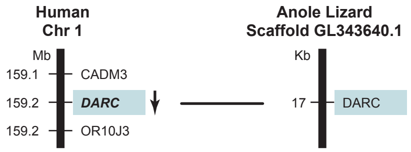

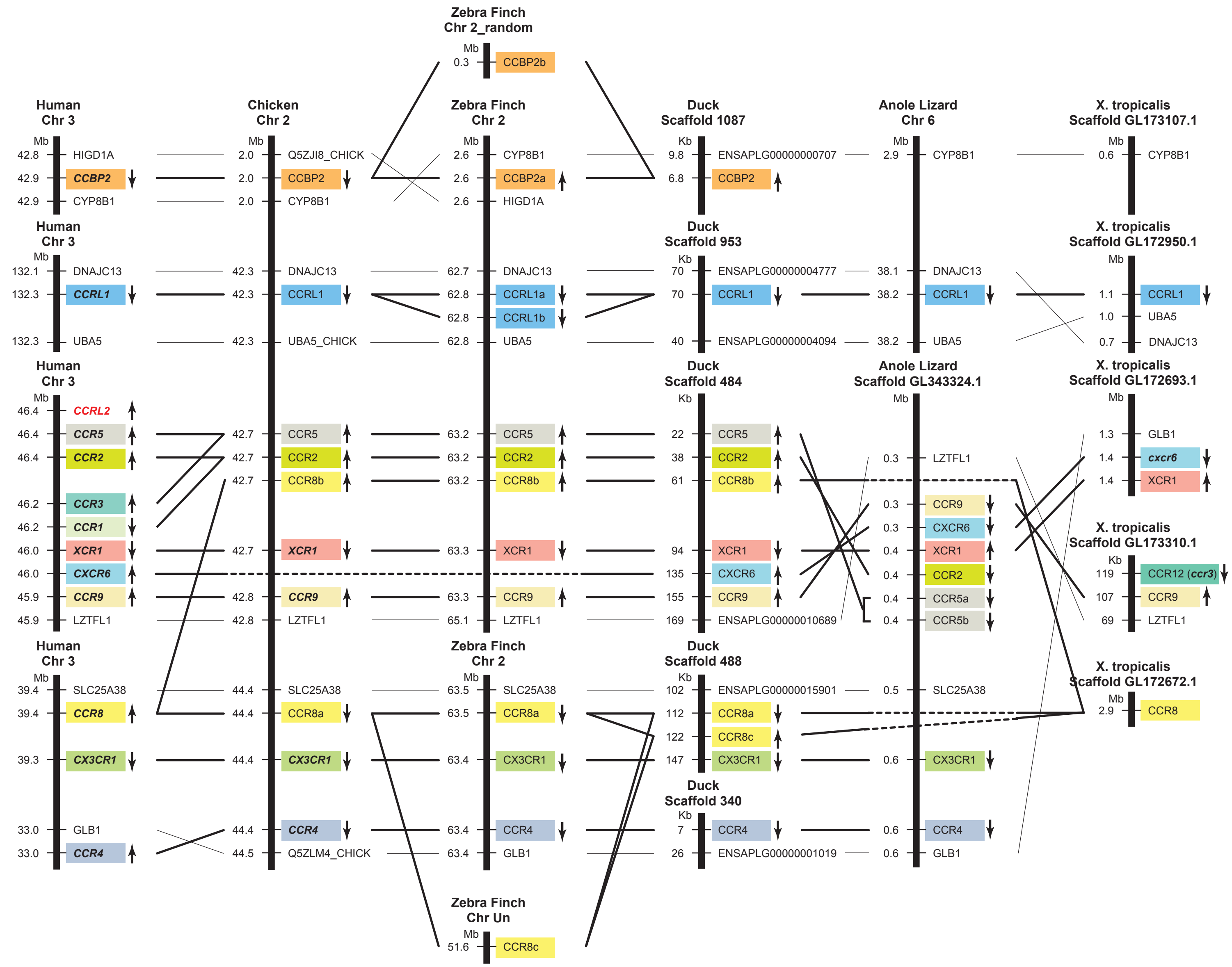
Zebra Finch
Chr 2_random
Mb
0.3 CCBP2b
Human
Chr 3
Mb
42.8 HIGD1A
42.9 CCBP2
42.9 CYP8B1
Chicken
Chr 2
Mb
2.0 Q5ZJI8_CHICK
2.0 CCBP2
2.0 CYP8B1
Zebra Finch
Chr 2
Mb
2.6 CYP8B1
2.6 CCBP2a
2.6 HIGD1A
Duck
Scaffold 1087
Kb
9.8 ENSAPLG00000000707
6.8 CCBP2
Anole Lizard
Chr 6
Mb
2.9 CYP8B1
X. tropicalis
Scaffold GL173107.1
Mb
0.6 CYP8B1
Human
Chr 3
Mb
132.1 DNAJC13
132.3 CCRL1
132.3 UBA5
42.3 DNAJC13
42.3 CCRL1
42.3 UBA5_CHICK
62.7 DNAJC13
62.8 CCRL1a
62.8 CCRL1b
62.8 UBA5
Duck
Scaffold 953
Kb
70 ENSAPLG00000004777
70 CCRL1
40 ENSAPLG00000004094
38.1 DNAJC13
38.2 CCRL1
38.2 UBA5
X. tropicalis
Scaffold GL172950.1
Mb
1.1 CCRL1
1.0 UBA5
0.7 DNAJC13
Human
Chr 3
Mb
46.4 CCRL2
46.4 CCR5
46.4 CCR2
46.2 CCR3
46.2 CCR1
46.0 XCR1
46.0 CXCR6
45.9 CCR9
45.9 LZTFL1
42.7 CCR5
42.7 CCR2
42.7 CCR8b
42.7 XCR1
42.8 CCR9
42.8 LZTFL1
63.2 CCR5
63.2 CCR2
63.2 CCR8b
63.3 XCR1
63.3 CCR9
65.1 LZTFL1
Duck
Scaffold 484
Kb
22 CCR5
38 CCR2
61 CCR8b
94 XCR1
135 CXCR6
155 CCR9
169 ENSAPLG00000010689
Anole Lizard
Scaffold GL343324.1
Mb
0.3 LZTFL1
0.3 CCR9
0.3 CXCR6
0.4 XCR1
0.4 CCR2
0.4 CCR5a
0.4 CCR5b
X. tropicalis
Scaffold GL172693.1
Mb
1.3 GLB1
1.4 cxcr6
1.4 XCR1
X. tropicalis
Scaffold GL173310.1
Kb
119 CCR12 (ccr3)
107 CCR9
69 LZTFL1
Human
Chr 3
Mb
39.4 SLC25A38
39.4 CCR8
39.3 CX3CR1
33.0 GLB1
33.0 CCR4
44.4 SLC25A38
44.4 CCR8a
44.4 CX3CR1
44.4 CCR4
44.5 Q5ZLM4_CHICK
Zebra Finch
Chr 2
Mb
63.5 SLC25A38
63.5 CCR8a
63.4 CX3CR1
63.4 CCR4
63.4 GLB1
Duck
Scaffold 488
Kb
102 ENSAPLG00000015901
112 CCR8a
122 CCR8c
147 CX3CR1
Duck
Scaffold 340
Kb
7 CCR4
26 ENSAPLG00000001019
0.5 SLC25A38
0.6 CX3CR1
0.6 CCR4
0.6 GLB1
X. tropicalis
Scaffold GL172672.1
Mb
2.9 CCR8
Zebra Finch
Chr Un
Mb
51.6 CCR8c

(c) Fish

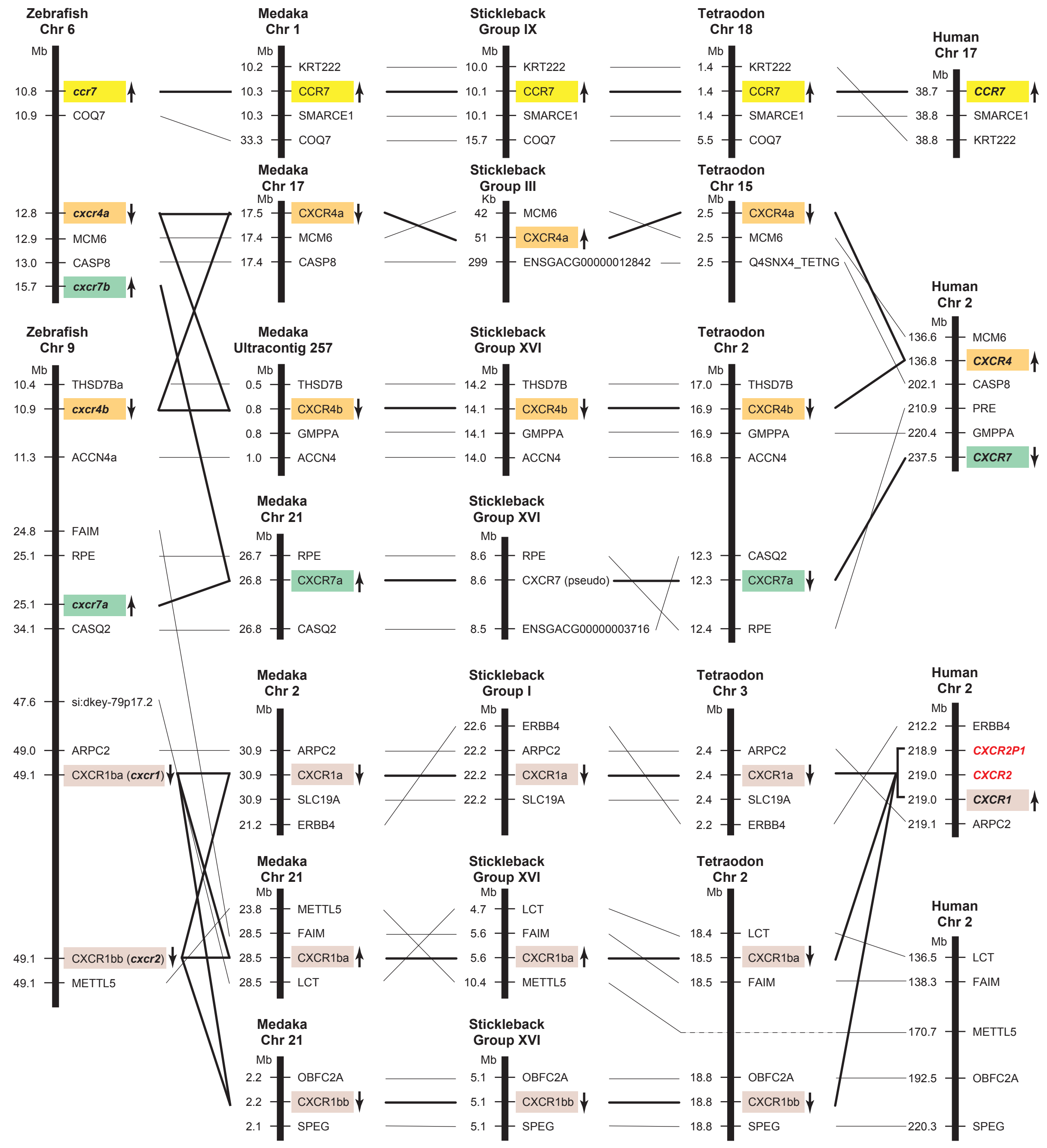

Zebrafish Chr 6
Medaka Chr 1
Stickleback Group IX
Tetraodon Chr 18
Human Chr 17
ccr7
CCR7
COQ7
SMARCE1
KRT222
Medaka Chr 17
Stickleback Group III
Tetraodon Chr 15
cxcr4a
CXCR4a
MCM6
CASP8
cxcr7b
ENSGACG00000012842
Q4SNX4_TETNG
Human Chr 2
CXCR4
PRE
GMPPA
CXCR7
Zebrafish Chr 9
Medaka Ultracontig 257
Stickleback Group XVI
Tetraodon Chr 2
THSD7Ba
THSD7B
cxcr4b
CXCR4b
ACCN4a
ACCN4
Medaka Chr 21
FAIM
RPE
cxcr7a
CXCR7a
CXCR7 (pseudo)
CASQ2
ENSGACG00000003716
si:dkey-79p17.2
Medaka Chr 2
Stickleback Group I
Tetraodon Chr 3
ARPC2
CXCR1ba (cxcr1)
CXCR1a
SLC19A
ERBB4
CXCR2P1
CXCR2
CXCR1
METTL5
LCT
CXCR1bb (cxcr2)
CXCR1ba
OBFC2A
CXCR1bb
SPEG

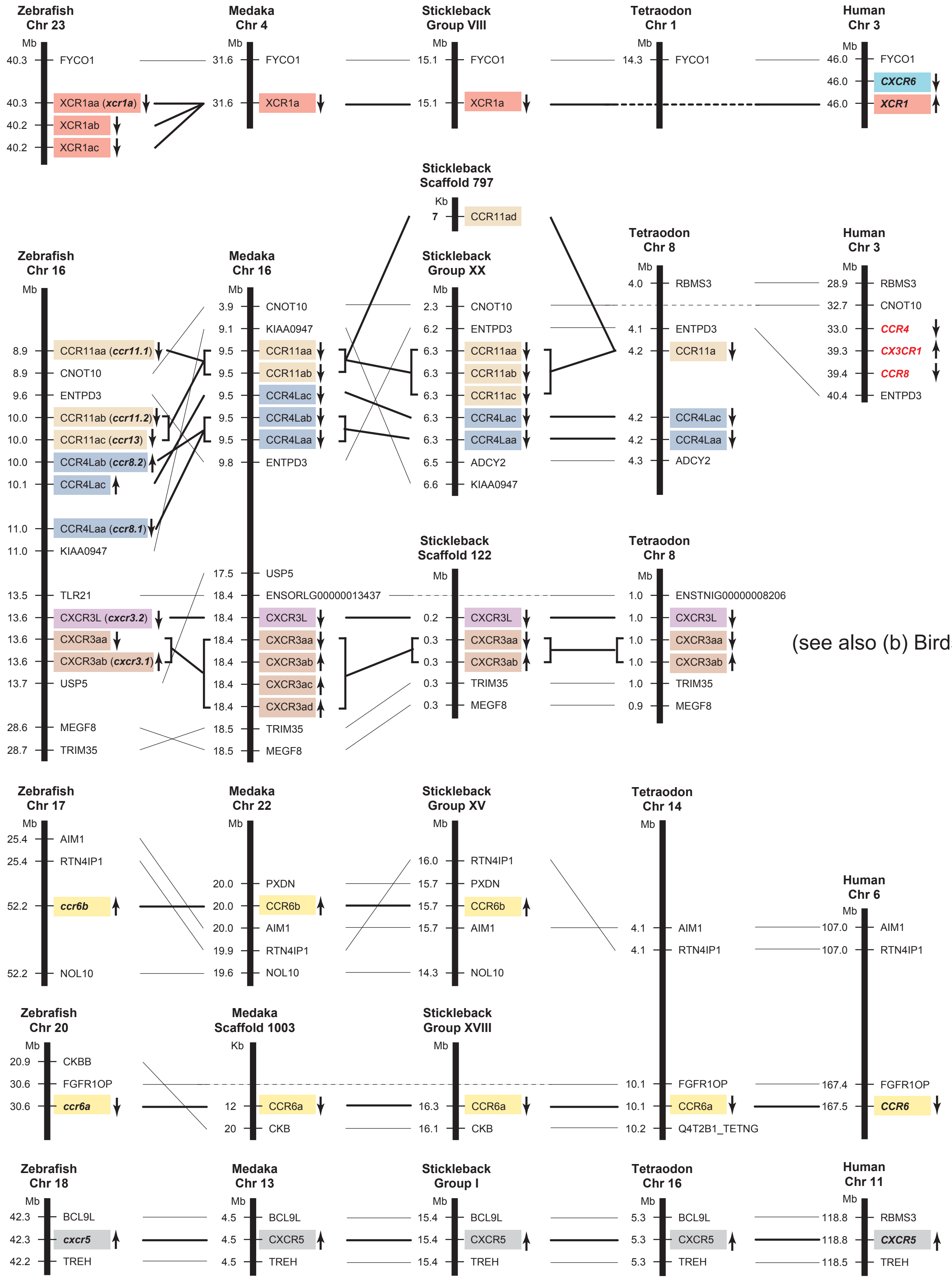

(see also (b) Birds p.15)

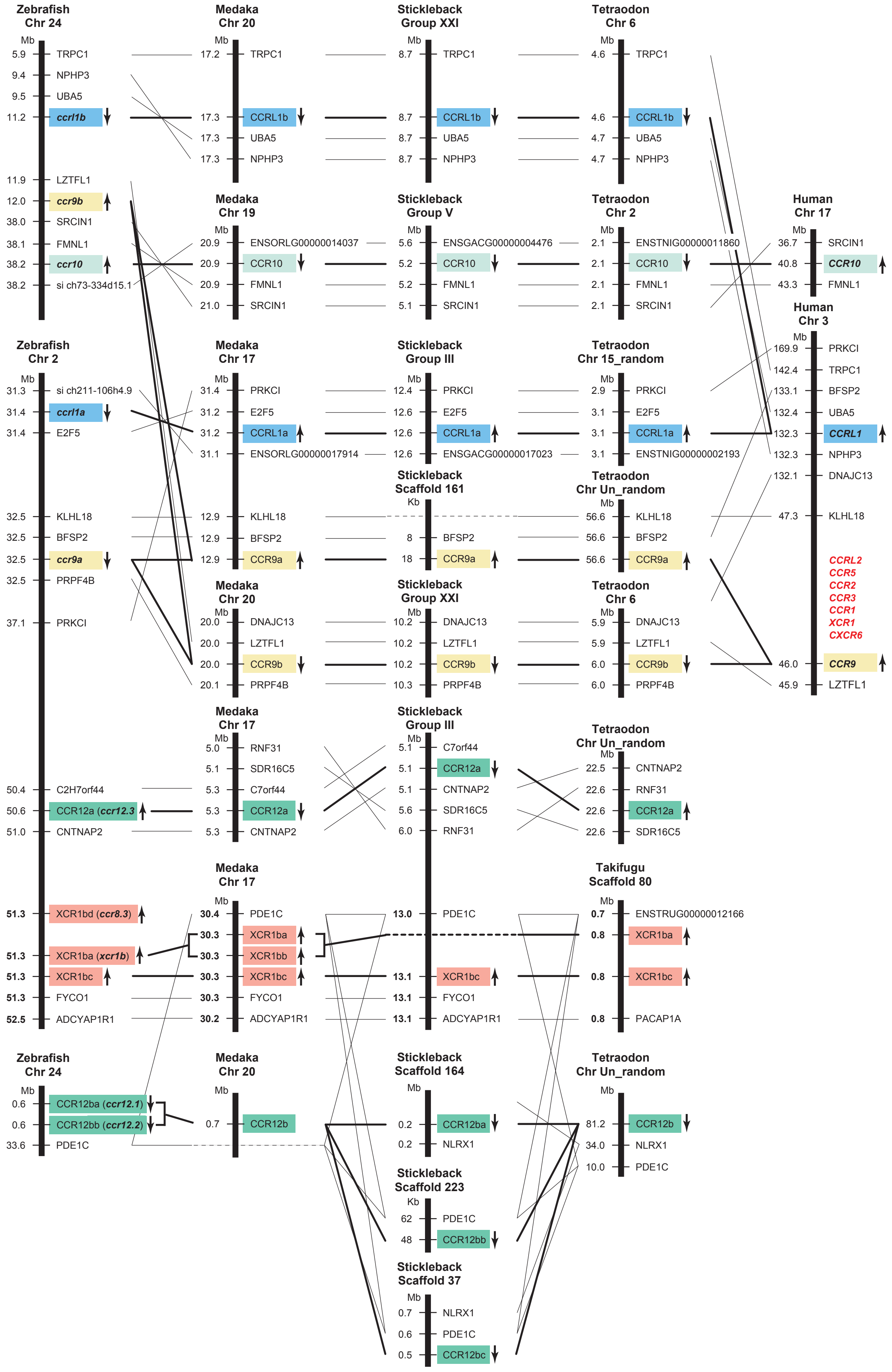
Zebrafish
Chr 24
Mb
5.9 TRPC1
9.4 NPHP3
9.5 UBA5
11.2 ccrl1b
11.9 LZTFL1
12.0 ccr9b
38.0 SRCIN1
38.1 FMNL1
38.2 ccr10
38.2 si ch73-334d15.1
Medaka
Chr 20
Mb
17.2 TRPC1
17.3 CCRL1b
17.3 UBA5
17.3 NPHP3
Stickleback
Group XXI
Mb
8.7 TRPC1
8.7 CCRL1b
8.7 UBA5
8.7 NPHP3
Tetraodon
Chr 6
Mb
4.6 TRPC1
4.6 CCRL1b
4.7 UBA5
4.7 NPHP3
Medaka
Chr 19
Mb
20.9 ENSORLG00000014037
20.9 CCR10
20.9 FMNL1
21.0 SRCIN1
Stickleback
Group V
Mb
5.6 ENSGACG00000004476
5.2 CCR10
5.2 FMNL1
5.1 SRCIN1
Tetraodon
Chr 2
Mb
2.1 ENSTNIG00000011860
2.1 CCR10
2.1 FMNL1
2.1 SRCIN1
Human
Chr 17
Mb
36.7 SRCIN1
40.8 CCR10
43.3 FMNL1
Human
Chr 3
Mb
169.9 PRKCI
142.4 TRPC1
133.1 BFSP2
132.4 UBA5
132.3 CCRL1
132.3 NPHP3
132.1 DNAJC13
47.3 KLHL18
CCRL2
CCR5
CCR2
CCR3
CCR1
XCR1
CXCR6
46.0 CCR9
45.9 LZTFL1
Zebrafish
Chr 2
Mb
31.3 si ch211-106h4.9
31.4 ccrl1a
31.4 E2F5
32.5 KLHL18
32.5 BFSP2
32.5 ccr9a
32.5 PRPF4B
37.1 PRKCI
Medaka
Chr 17
Mb
31.4 PRKCI
31.2 E2F5
31.2 CCRL1a
31.1 ENSORLG00000017914
12.9 KLHL18
12.9 BFSP2
12.9 CCR9a
Stickleback
Group III
Mb
12.4 PRKCI
12.6 E2F5
12.6 CCRL1a
12.6 ENSGACG00000017023
Tetraodon
Chr 15_random
Mb
2.9 PRKCI
3.1 E2F5
3.1 CCRL1a
3.1 ENSTNIG00000002193
Stickleback
Scaffold 161
Kb
8 BFSP2
18 CCR9a
Tetraodon
Chr Un_random
Mb
56.6 KLHL18
56.6 BFSP2
56.6 CCR9a
Medaka
Chr 20
Mb
20.0 DNAJC13
20.0 LZTFL1
20.0 CCR9b
20.1 PRPF4B
Stickleback
Group XXI
Mb
10.2 DNAJC13
10.2 LZTFL1
10.2 CCR9b
10.3 PRPF4B
Tetraodon
Chr 6
Mb
5.9 DNAJC13
5.9 LZTFL1
6.0 CCR9b
6.0 PRPF4B
Medaka
Chr 17
Mb
5.0 RNF31
5.1 SDR16C5
5.3 C7orf44
5.3 CCR12a
5.3 CNTNAP2
50.4 C2H7orf44
50.6 CCR12a (ccr12.3)
51.0 CNTNAP2
Stickleback
Group III
Mb
5.1 C7orf44
5.1 CCR12a
5.1 CNTNAP2
5.6 SDR16C5
6.0 RNF31
Tetraodon
Chr Un_random
Mb
22.5 CNTNAP2
22.6 RNF31
22.6 CCR12a
22.6 SDR16C5
Medaka
Chr 17
Mb
30.4 PDE1C
30.3 XCR1ba
30.3 XCR1bb
30.3 XCR1bc
30.3 FYCO1
30.2 ADCYAP1R1
Takifugu
Scaffold 80
Mb
0.7 ENSTRUG00000012166
0.8 XCR1ba
0.8 XCR1bc
0.8 PACAP1A
51.3 XCR1bd (ccr8.3)
51.3 XCR1ba (xcr1b)
51.3 XCR1bc
51.3 FYCO1
52.5 ADCYAP1R1
13.0 PDE1C
13.1 XCR1bc
13.1 FYCO1
13.1 ADCYAP1R1
Zebrafish
Chr 24
Mb
0.6 CCR12ba (ccr12.1)
0.6 CCR12bb (ccr12.2)
33.6 PDE1C
Medaka
Chr 20
Mb
0.7 CCR12b
Stickleback
Scaffold 164
Mb
0.2 CCR12ba
0.2 NLRX1
Tetraodon
Chr Un_random
Mb
81.2 CCR12b
34.0 NLRX1
10.0 PDE1C
Stickleback
Scaffold 223
Kb
62 PDE1C
48 CCR12bb
Stickleback
Scaffold 37
Mb
0.7 NLRX1
0.6 PDE1C
0.5 CCR12bc
