# Supplementary material for: Systematic classification of vertebrate chemokines based on conserved synteny and evolutionary history
Source: Genes Cells. 2012 Nov 12;18(1):1–16. doi: 10.1111/gtc.12013 (PMC3568907; doi:10.1111/gtc.12013)
Supplement: Supplementary file 7 [file gtc0018-0001-SD4.pdf]

Fig. S4

Phylogenetic trees of vertebrate chemokines and chemokine receptors.

The trees were constructed using the neighbour-joining method with Dayhoff’ s (PAM) matrix, and gaps were removed by pairwise deletion. Incomplete sequences and DARC sequences were omitted from the tree construction. DARC sequences are highly divergent from other chemokine receptor sequences, and therefore, their inclusion in the construction would have reduced the reliability of the tree.

A. Chemokines.

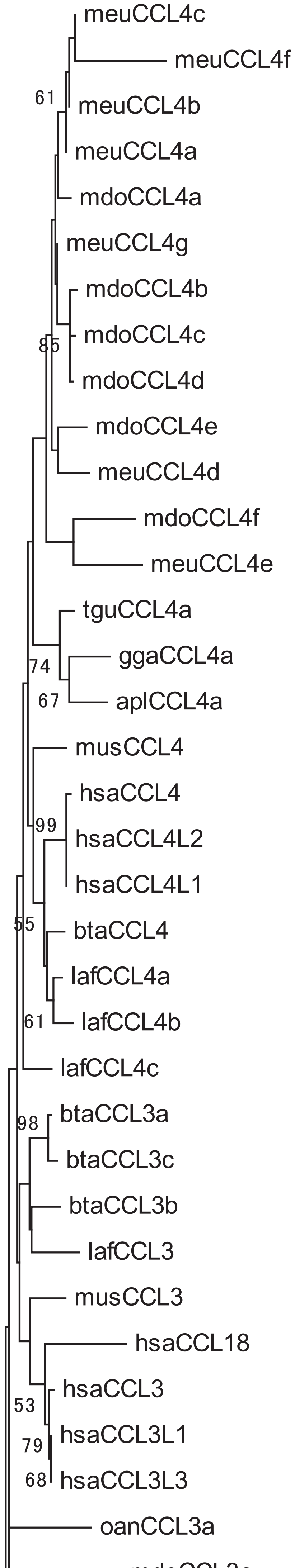

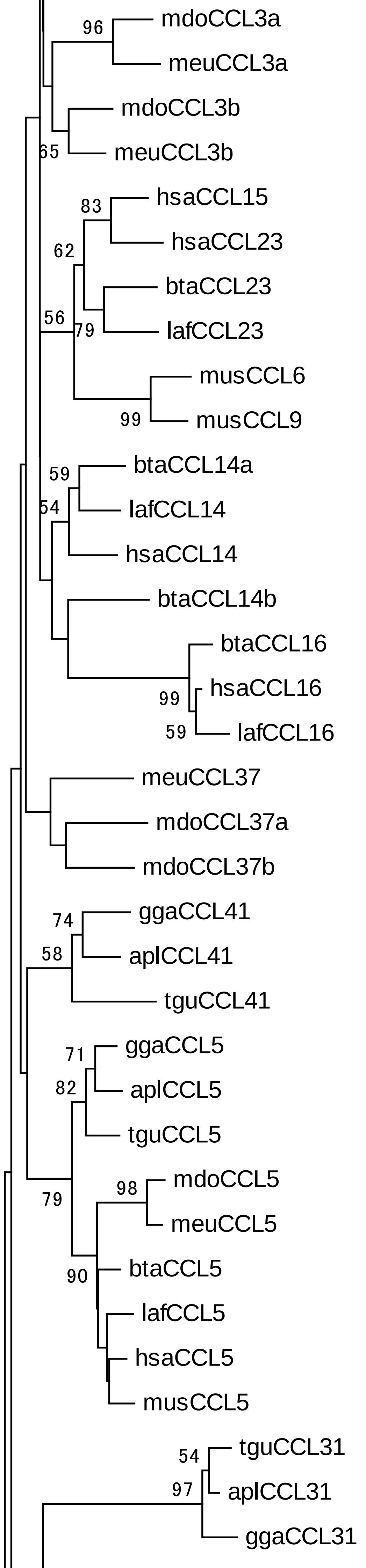
mdoCCL3a
meuCCL3a
mdoCCL3b
meuCCL3b
hsaCCL15
hsaCCL23
btaCCL23
lafCCL23
musCCL6
musCCL9
btaCCL14a
lafCCL14
hsaCCL14
btaCCL14b
btaCCL16
hsaCCL16
lafCCL16
meuCCL37
mdoCCL37a
mdoCCL37b
ggaCCL41
aplCCL41
tguCCL41
ggaCCL5
aplCCL5
tguCCL5
mdoCCL5
meuCCL5
btaCCL5
lafCCL5
hsaCCL5
musCCL5
tguCCL31
aplCCL31
ggaCCL31
96
65
83
62
56
79
99
59
54
99
59
74
58
71
82
79
98
90
54
97

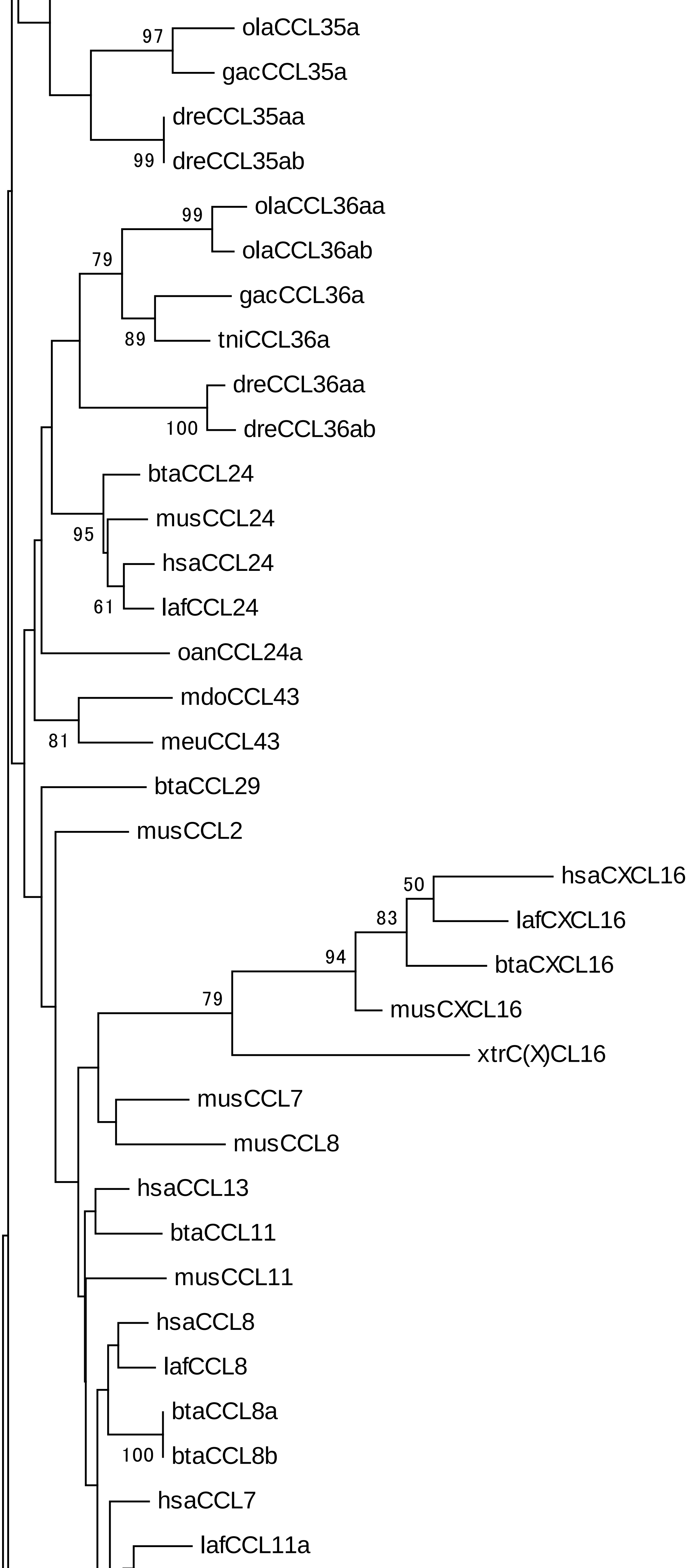
97
olaCCL35a
gacCCL35a
dreCCL35aa
99
dreCCL35ab
99
olaCCL36aa
79
olaCCL36ab
gacCCL36a
89
tniCCL36a
dreCCL36aa
100
dreCCL36ab
btaCCL24
95
musCCL24
hsaCCL24
61
lafCCL24
oanCCL24a
mdoCCL43
81
meuCCL43
btaCCL29
musCCL2
50
hsaCXCL16
83
lafCXCL16
94
btaCXCL16
79
musCXCL16
xtrC(X)CL16
musCCL7
musCCL8
hsaCCL13
btaCCL11
musCCL11
hsaCCL8
lafCCL8
btaCCL8a
100
btaCCL8b
hsaCCL7
lafCCL11a

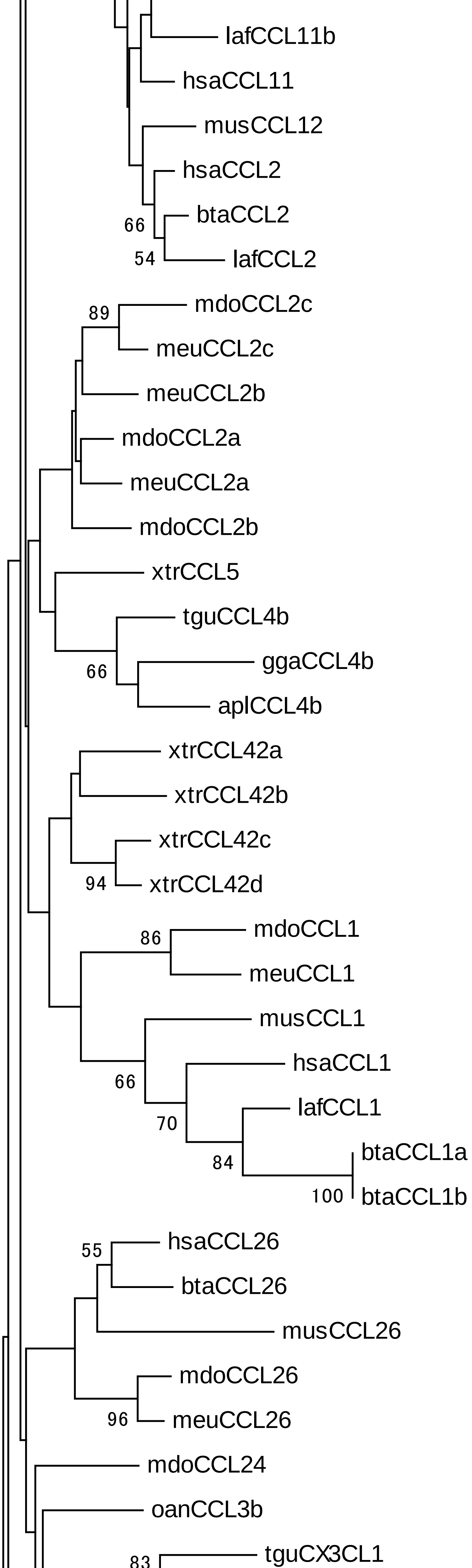

lafCCL11b
hsaCCL11
musCCL12
hsaCCL2
btaCCL2
66
54
lafCCL2
mdoCCL2c
89
meuCCL2c
meuCCL2b
mdoCCL2a
meuCCL2a
mdoCCL2b
xtrCCL5
tguCCL4b
ggaCCL4b
66
aplCCL4b
xtrCCL42a
xtrCCL42b
xtrCCL42c
94
xtrCCL42d
86
mdoCCL1
meuCCL1
musCCL1
hsaCCL1
66
lafCCL1
70
btaCCL1a
84
100
btaCCL1b
55
hsaCCL26
btaCCL26
musCCL26
mdoCCL26
96
meuCCL26
mdoCCL24
oanCCL3b
83
tguCX3CL1

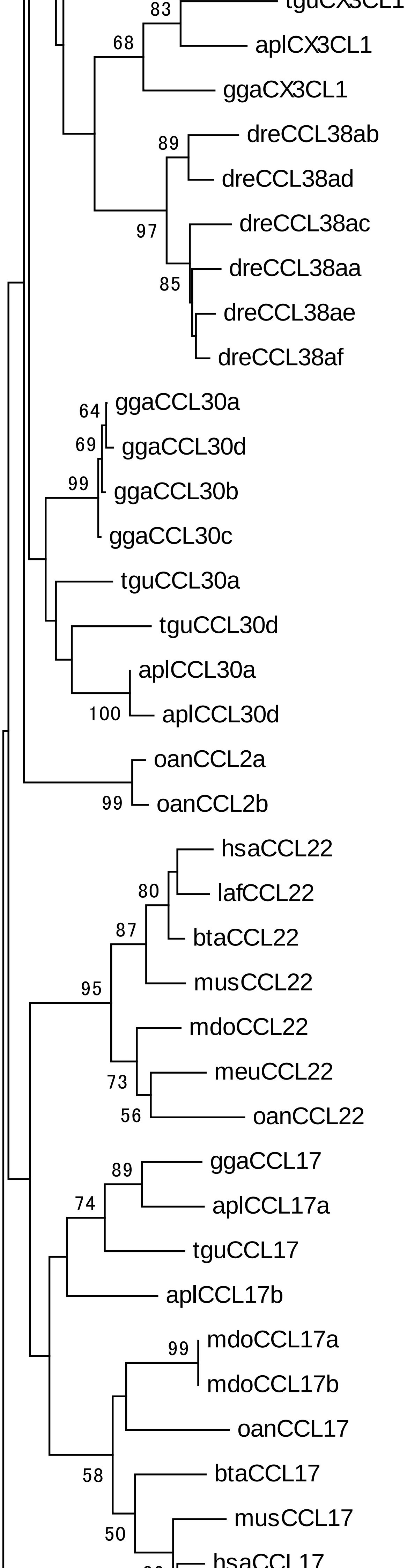
aplCX3CL1
ggaCX3CL1
dreCCL38ab
dreCCL38ad
dreCCL38ac
dreCCL38aa
dreCCL38ae
dreCCL38af
ggaCCL30a
ggaCCL30d
ggaCCL30b
ggaCCL30c
tguCCL30a
tguCCL30d
aplCCL30a
aplCCL30d
oanCCL2a
oanCCL2b
hsaCCL22
lafCCL22
btaCCL22
musCCL22
mdoCCL22
meuCCL22
oanCCL22
ggaCCL17
aplCCL17a
tguCCL17
aplCCL17b
mdoCCL17a
mdoCCL17b
oanCCL17
btaCCL17
musCCL17

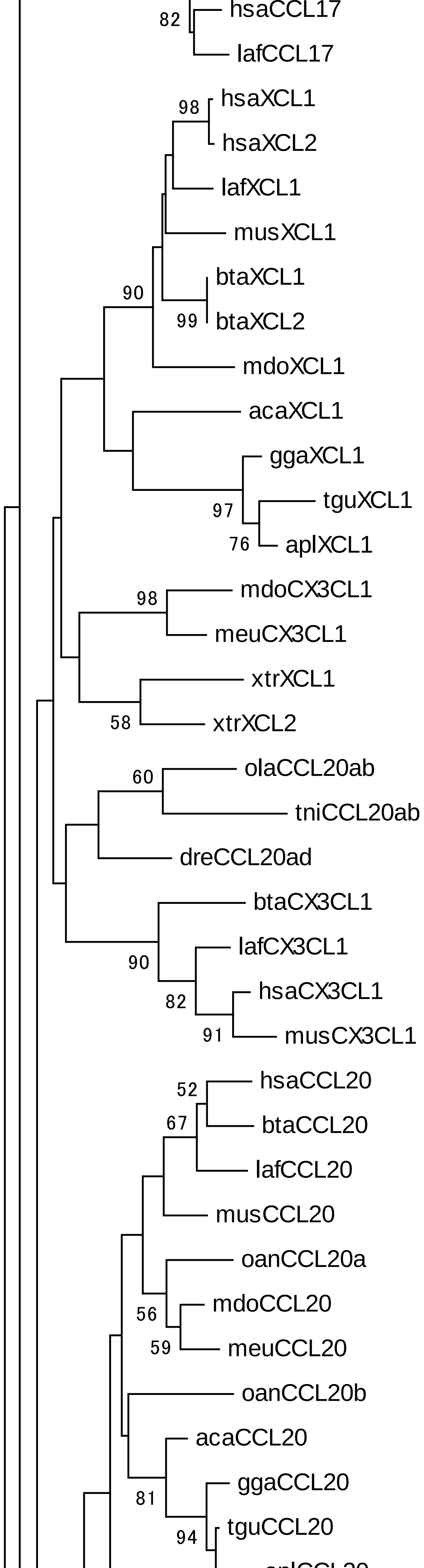

82
hsaCCL17
lafCCL17
98
hsaXCL1
hsaXCL2
lafXCL1
musXCL1
90
btaXCL1
99
btaXCL2
mdoXCL1
acaXCL1
ggaXCL1
97
tguXCL1
76
aplXCL1
98
mdoCX3CL1
meuCX3CL1
xtrXCL1
58
xtrXCL2
60
olaCCL20ab
tniCCL20ab
dreCCL20ad
btaCX3CL1
90
lafCX3CL1
82
hsaCX3CL1
91
musCX3CL1
52
hsaCCL20
67
btaCCL20
lafCCL20
musCCL20
oanCCL20a
56
mdoCCL20
59
meuCCL20
oanCCL20b
acaCCL20
81
ggaCCL20
94
tguCCL20

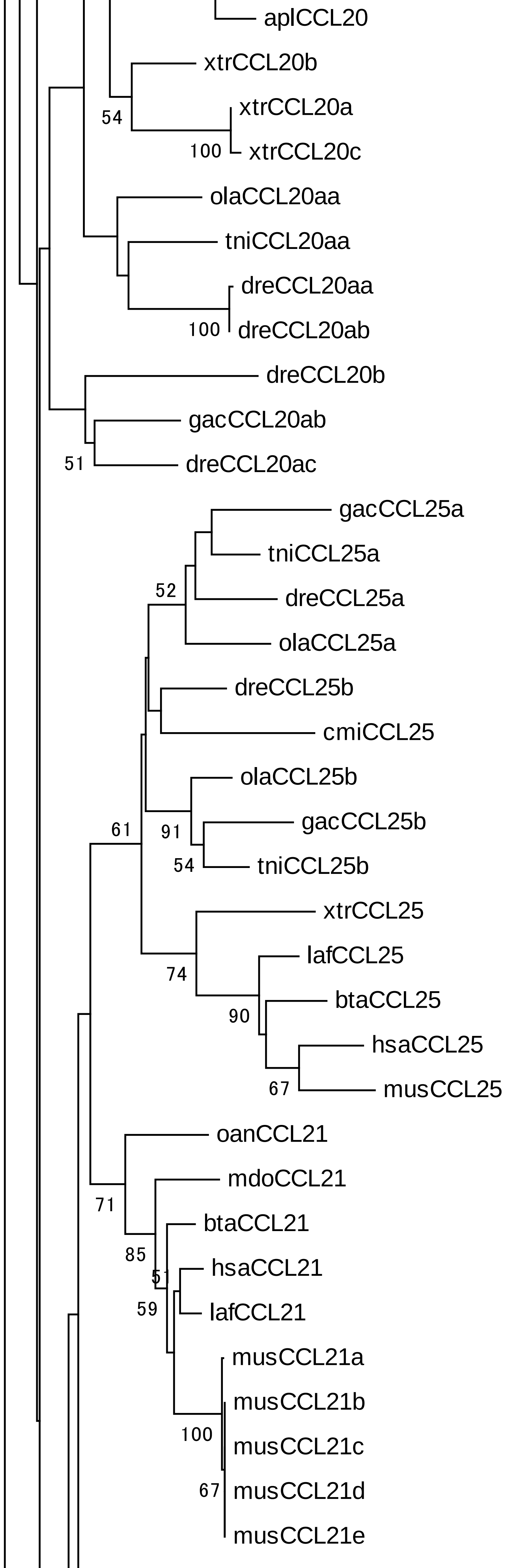

aplCCL20
xtrCCL20b
54
xtrCCL20a
100
xtrCCL20c
olaCCL20aa
tniCCL20aa
dreCCL20aa
100
dreCCL20ab
dreCCL20b
gacCCL20ab
51
dreCCL20ac
gacCCL25a
tniCCL25a
52
dreCCL25a
olaCCL25a
dreCCL25b
cmiCCL25
olaCCL25b
61
91
gacCCL25b
54
tniCCL25b
xtrCCL25
lafCCL25
74
btaCCL25
90
hsaCCL25
67
musCCL25
oanCCL21
mdoCCL21
71
btaCCL21
85
51
hsaCCL21
59
lafCCL21
musCCL21a
musCCL21b
100
musCCL21c
67
musCCL21d
musCCL21e

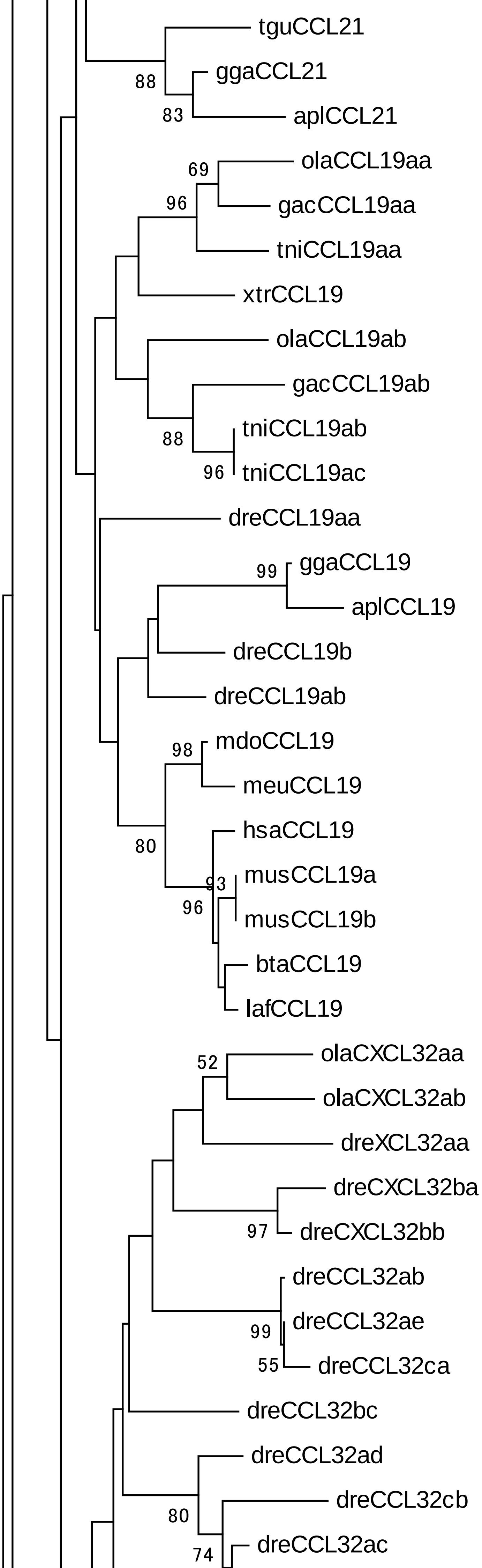
tguCCL21
88
ggaCCL21
83
aplCCL21
69
olaCCL19aa
96
gacCCL19aa
tniCCL19aa
xtrCCL19
olaCCL19ab
gacCCL19ab
88
tniCCL19ab
96
tniCCL19ac
dreCCL19aa
99
ggaCCL19
aplCCL19
dreCCL19b
dreCCL19ab
98
mdoCCL19
meuCCL19
80
hsaCCL19
93
musCCL19a
96
musCCL19b
btaCCL19
lafCCL19
52
olaCXCL32aa
olaCXCL32ab
dreXCL32aa
dreCXCL32ba
97
dreCXCL32bb
dreCCL32ab
99
dreCCL32ae
55
dreCCL32ca
dreCCL32bc
dreCCL32ad
dreCCL32cb
80
74
dreCCL32ac

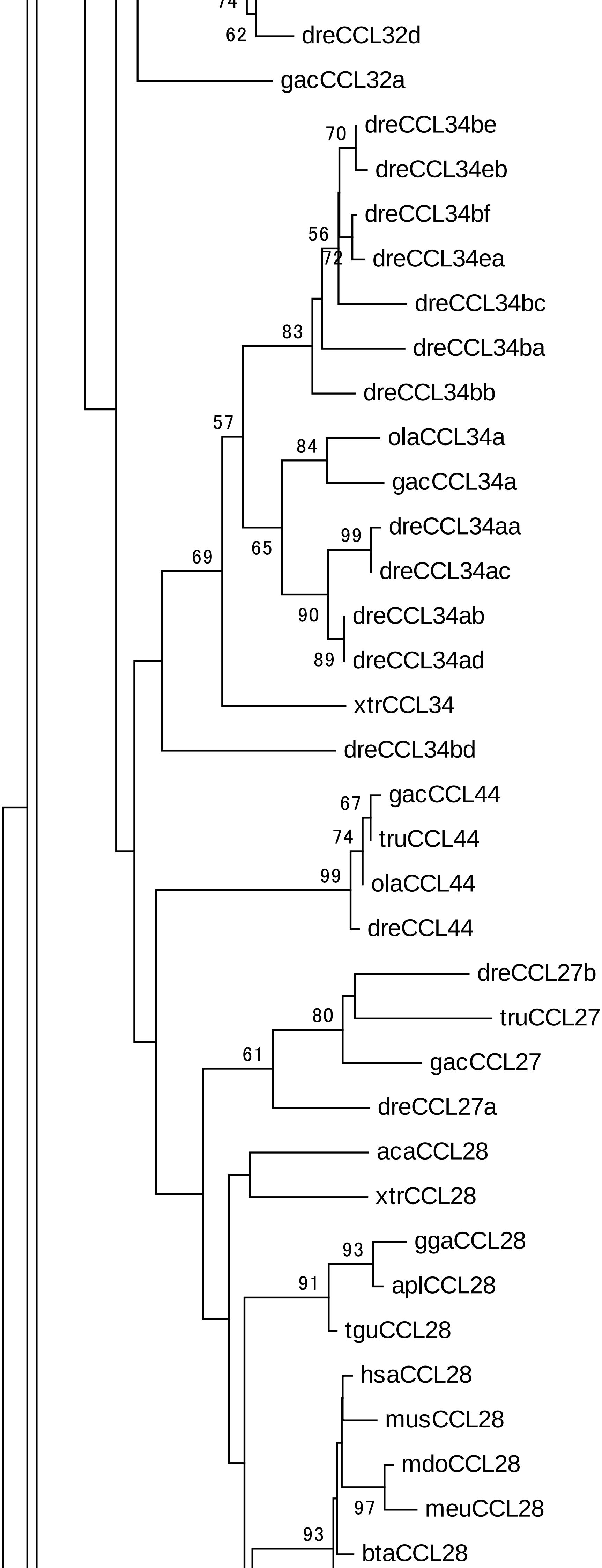
74
62
dreCCL32d
gacCCL32a
70
dreCCL34be
dreCCL34eb
dreCCL34bf
56
72
dreCCL34ea
dreCCL34bc
83
dreCCL34ba
dreCCL34bb
57
84
olaCCL34a
gacCCL34a
99
dreCCL34aa
69
65
dreCCL34ac
90
dreCCL34ab
89
dreCCL34ad
xtrCCL34
dreCCL34bd
67
gacCCL44
74
truCCL44
99
olaCCL44
dreCCL44
dreCCL27b
80
truCCL27
61
gacCCL27
dreCCL27a
acaCCL28
xtrCCL28
93
ggaCCL28
91
aplCCL28
tguCCL28
hsaCCL28
musCCL28
mdoCCL28
97
meuCCL28
93
btaCCL28

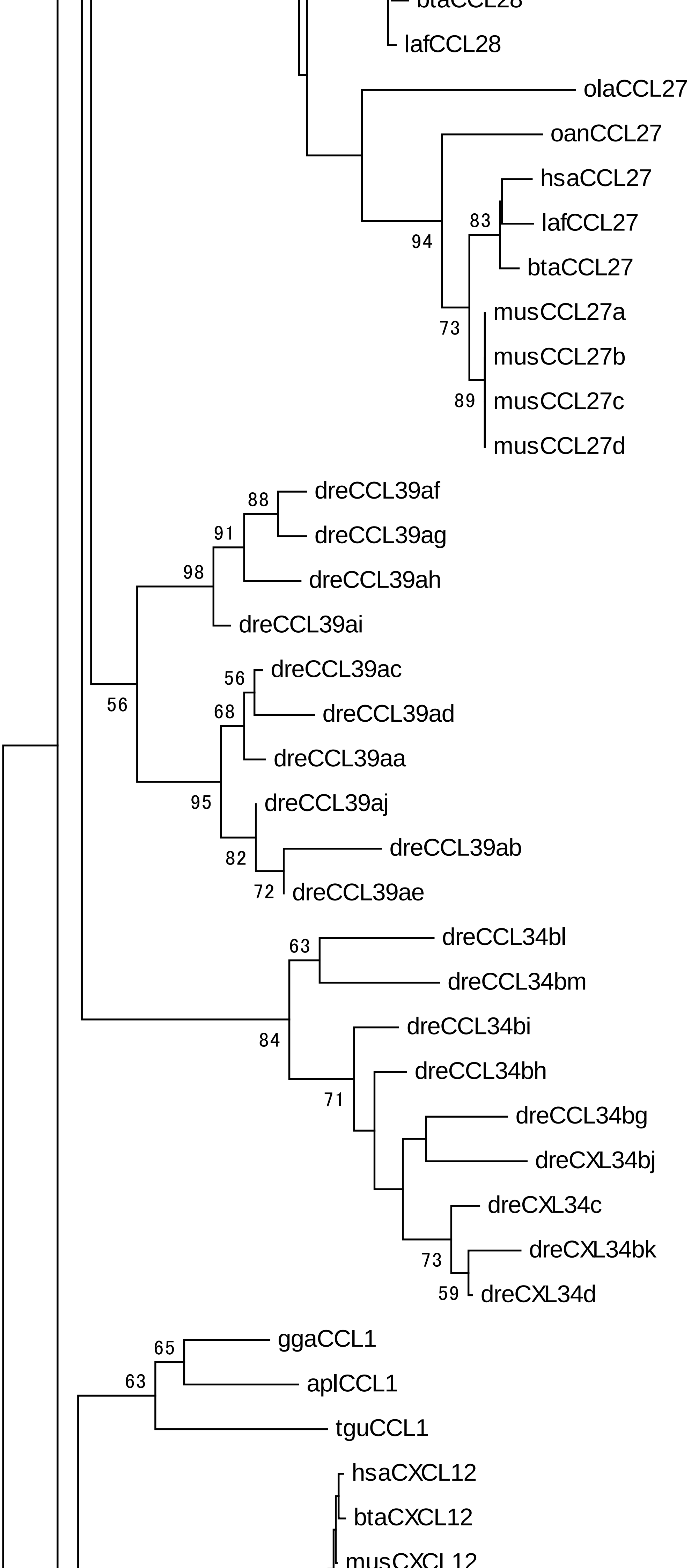
lafCCL28
olaCCL27
oanCCL27
hsaCCL27
83
lafCCL27
94
btaCCL27
musCCL27a
73
musCCL27b
89
musCCL27c
musCCL27d
88
dreCCL39af
91
dreCCL39ag
98
dreCCL39ah
dreCCL39ai
56
dreCCL39ac
56
68
dreCCL39ad
dreCCL39aa
95
dreCCL39aj
82
dreCCL39ab
72
dreCCL39ae
63
dreCCL34bl
dreCCL34bm
84
dreCCL34bi
71
dreCCL34bh
dreCCL34bg
dreCXL34bj
dreCXL34c
73
dreCXL34bk
59
dreCXL34d
65
ggaCCL1
63
aplCCL1
tguCCL1
hsaCXCL12
btaCXCL12
musCXCL12

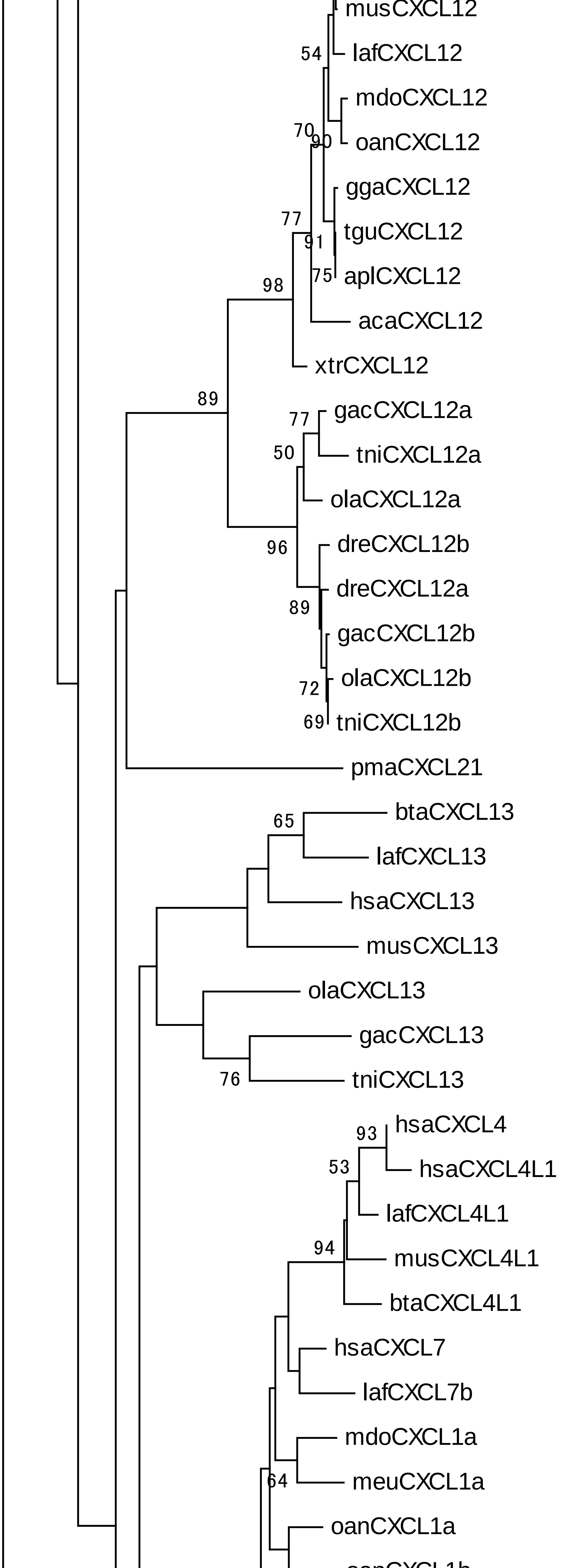
musCXCL12
54
lafCXCL12
mdoCXCL12
70
90
oanCXCL12
ggaCXCL12
77
91
tguCXCL12
75
aplCXCL12
98
acaCXCL12
xtrCXCL12
89
77
gacCXCL12a
50
tniCXCL12a
olaCXCL12a
dreCXCL12b
96
dreCXCL12a
89
gacCXCL12b
olaCXCL12b
72
69
tniCXCL12b
pmaCXCL21
65
btaCXCL13
lafCXCL13
hsaCXCL13
musCXCL13
olaCXCL13
gacCXCL13
76
tniCXCL13
93
hsaCXCL4
53
hsaCXCL4L1
lafCXCL4L1
94
musCXCL4L1
btaCXCL4L1
hsaCXCL7
lafCXCL7b
mdoCXCL1a
64
meuCXCL1a
oanCXCL1a

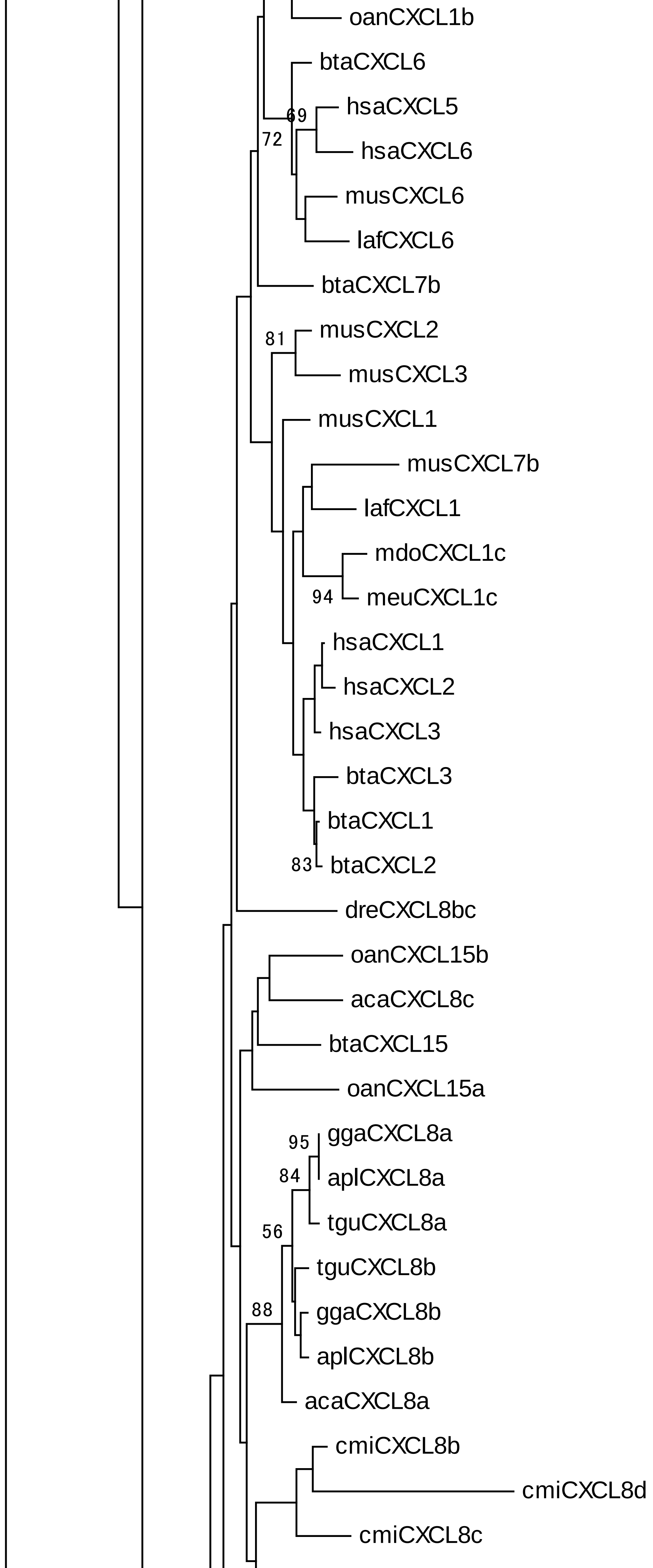
oanCXCL1b
btaCXCL6
69
hsaCXCL5
72
hsaCXCL6
musCXCL6
lafCXCL6
btaCXCL7b
81
musCXCL2
musCXCL3
musCXCL1
musCXCL7b
lafCXCL1
mdoCXCL1c
94
meuCXCL1c
hsaCXCL1
hsaCXCL2
hsaCXCL3
btaCXCL3
btaCXCL1
83
btaCXCL2
dreCXCL8bc
oanCXCL15b
acaCXCL8c
btaCXCL15
oanCXCL15a
95
ggaCXCL8a
84
aplCXCL8a
56
tguCXCL8a
tguCXCL8b
88
ggaCXCL8b
aplCXCL8b
acaCXCL8a
cmiCXCL8b
cmiCXCL8d
cmiCXCL8c

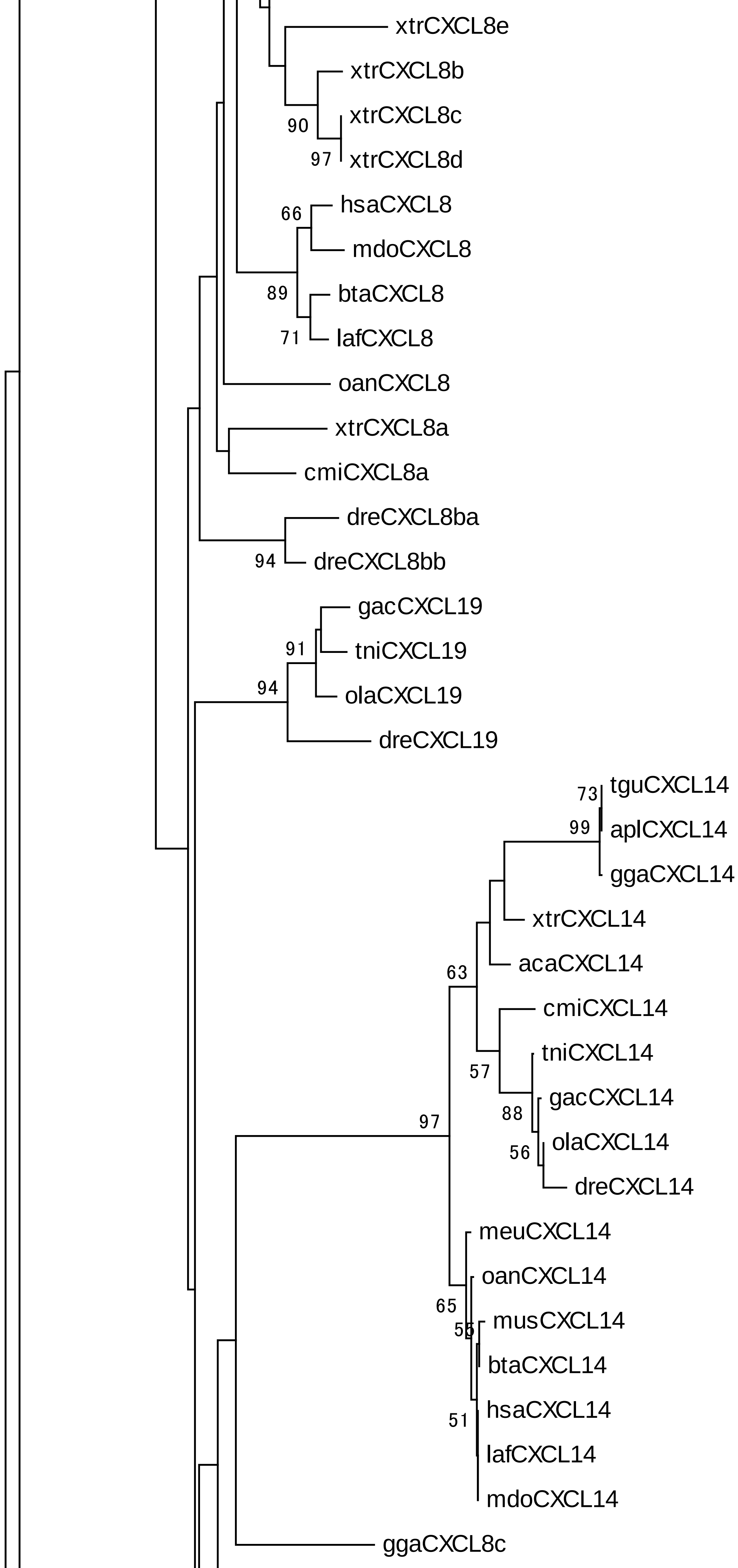
xtrCXCL8e
xtrCXCL8b
xtrCXCL8c
xtrCXCL8d
hsaCXCL8
mdoCXCL8
btaCXCL8
lafCXCL8
oanCXCL8
xtrCXCL8a
cmiCXCL8a
dreCXCL8ba
dreCXCL8bb
gacCXCL19
tniCXCL19
olaCXCL19
dreCXCL19
tguCXCL14
aplCXCL14
ggaCXCL14
xtrCXCL14
acaCXCL14
cmiCXCL14
tniCXCL14
gacCXCL14
olaCXCL14
dreCXCL14
meuCXCL14
oanCXCL14
musCXCL14
btaCXCL14
hsaCXCL14
lafCXCL14
mdoCXCL14
ggaCXCL8c

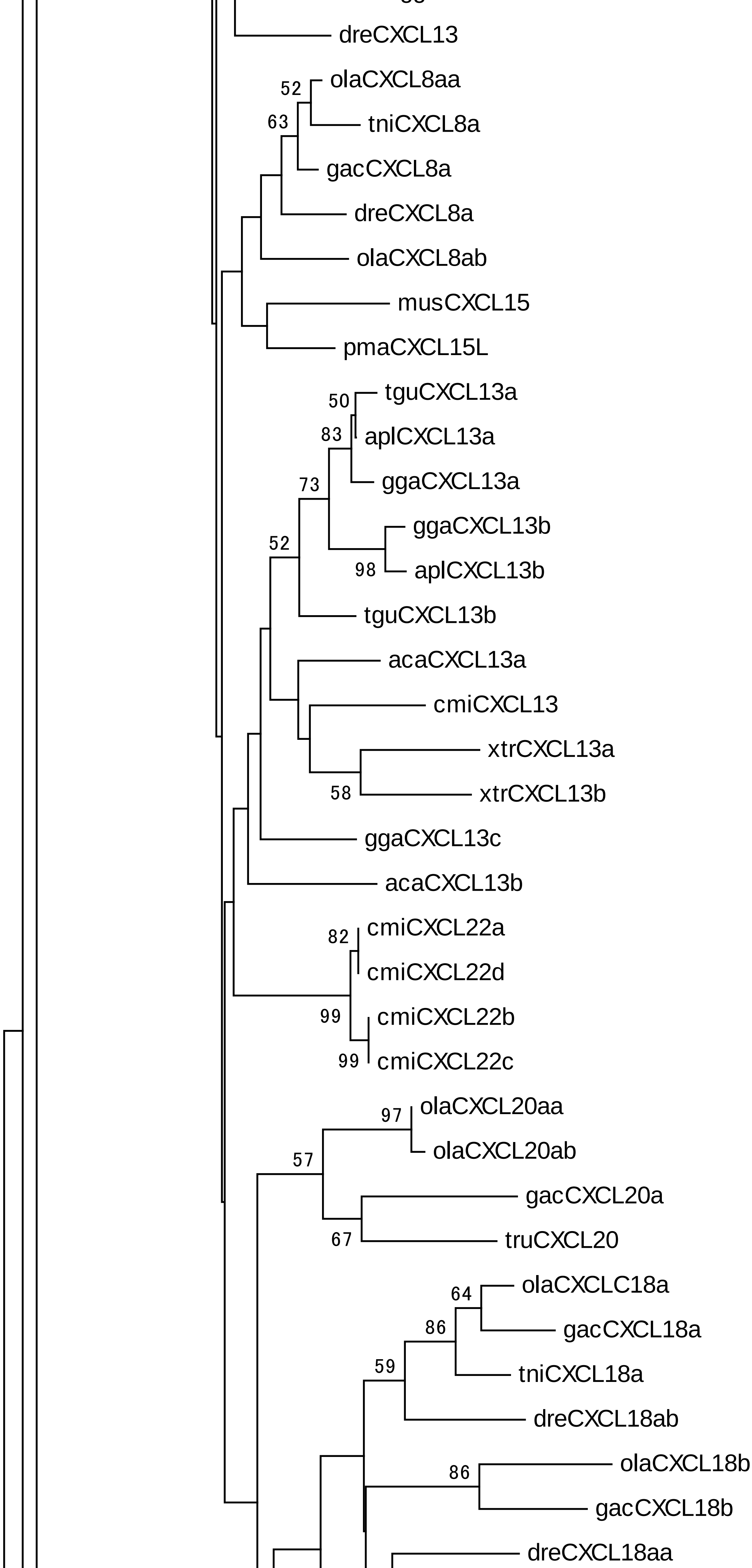

dreCXCL13
52
olaCXCL8aa
63
tniCXCL8a
gacCXCL8a
dreCXCL8a
olaCXCL8ab
musCXCL15
pmaCXCL15L
50
tguCXCL13a
83
aplCXCL13a
ggaCXCL13a
73
ggaCXCL13b
52
98
aplCXCL13b
tguCXCL13b
acaCXCL13a
cmiCXCL13
xtrCXCL13a
58
xtrCXCL13b
ggaCXCL13c
acaCXCL13b
82
cmiCXCL22a
cmiCXCL22d
99
cmiCXCL22b
99
cmiCXCL22c
97
olaCXCL20aa
olaCXCL20ab
57
gacCXCL20a
67
truCXCL20
64
olaCXCLC18a
86
gacCXCL18a
59
tniCXCL18a
dreCXCL18ab
86
olaCXCL18b
gacCXCL18b
dreCXCL18aa

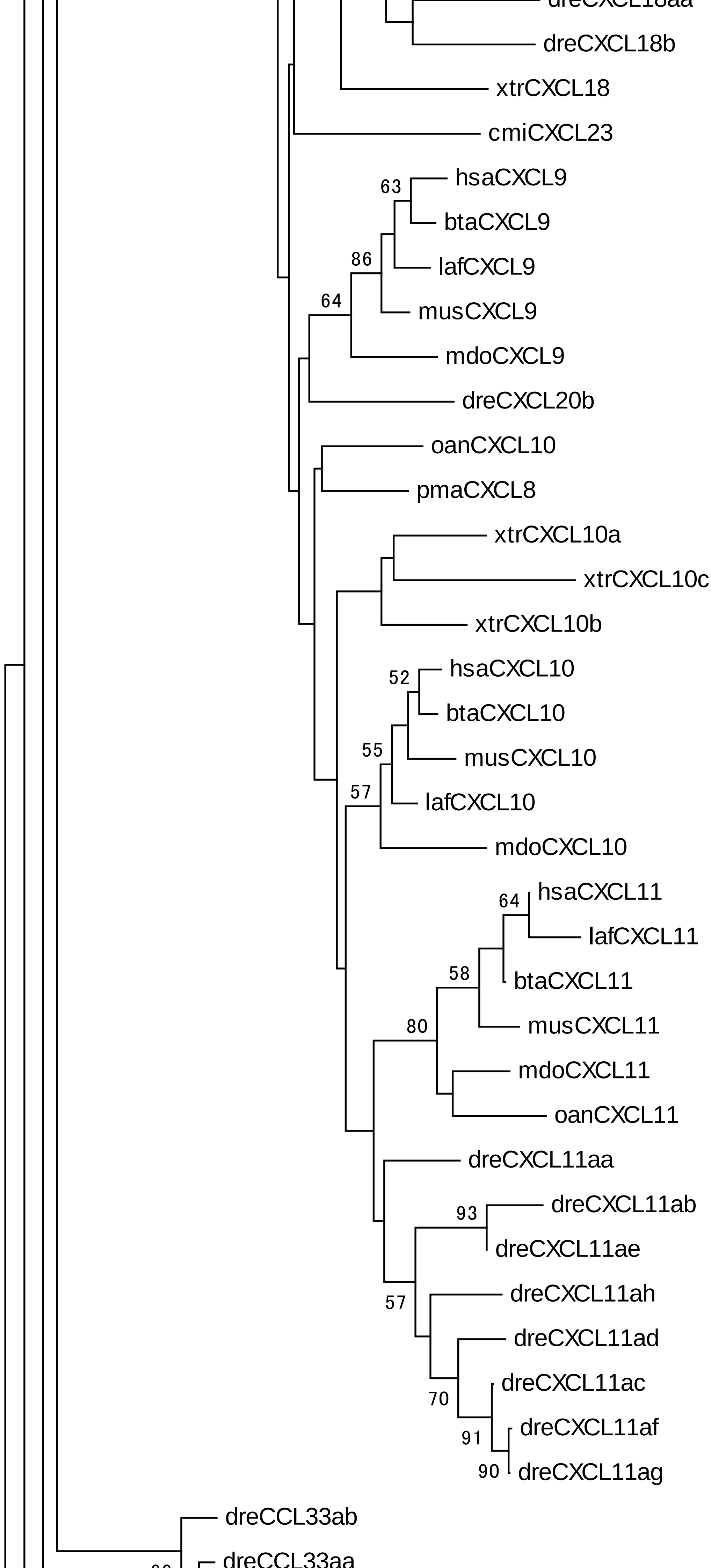
dreCXCL18b
xtrCXCL18
cmiCXCL23
63
hsaCXCL9
btaCXCL9
86
lafCXCL9
64
musCXCL9
mdoCXCL9
dreCXCL20b
oanCXCL10
pmaCXCL8
xtrCXCL10a
xtrCXCL10c
xtrCXCL10b
52
hsaCXCL10
btaCXCL10
55
musCXCL10
57
lafCXCL10
mdoCXCL10
64
hsaCXCL11
lafCXCL11
58
btaCXCL11
80
musCXCL11
mdoCXCL11
oanCXCL11
dreCXCL11aa
93
dreCXCL11ab
dreCXCL11ae
57
dreCXCL11ah
dreCXCL11ad
dreCXCL11ac
70
91
dreCXCL11af
90
dreCXCL11ag
dreCCL33ab
dreCCL33aa

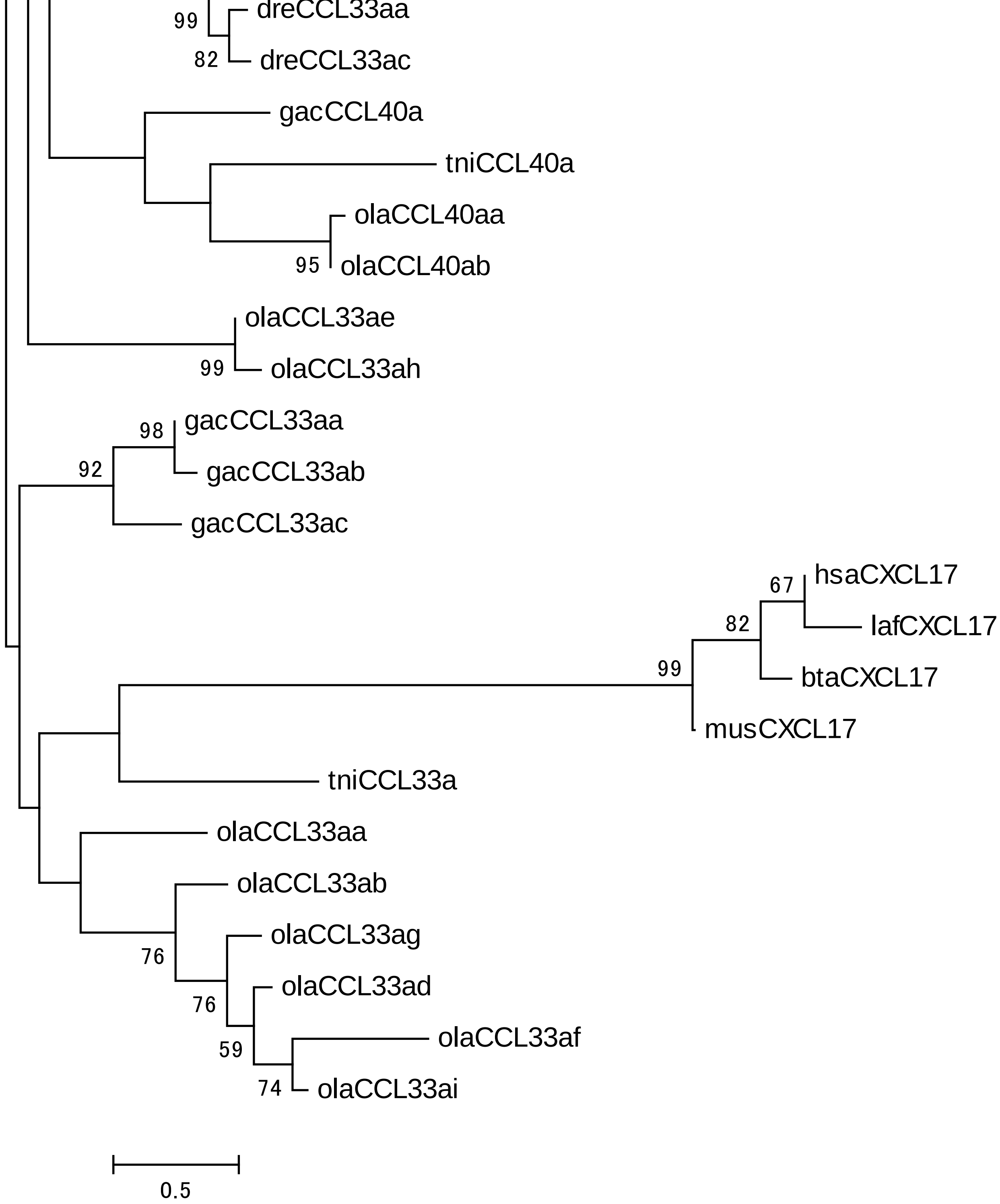

99
dreCCL33aa
82
dreCCL33ac
gacCCL40a
tniCCL40a
olaCCL40aa
95
olaCCL40ab
olaCCL33ae
99
olaCCL33ah
98
gacCCL33aa
92
gacCCL33ab
gacCCL33ac
67
hsaCXCL17
82
lafCXCL17
99
btaCXCL17
musCXCL17
tniCCL33a
olaCCL33aa
olaCCL33ab
olaCCL33ag
76
olaCCL33ad
76
olaCCL33af
59
74
olaCCL33ai
0.5

B. Chemokine receptors.

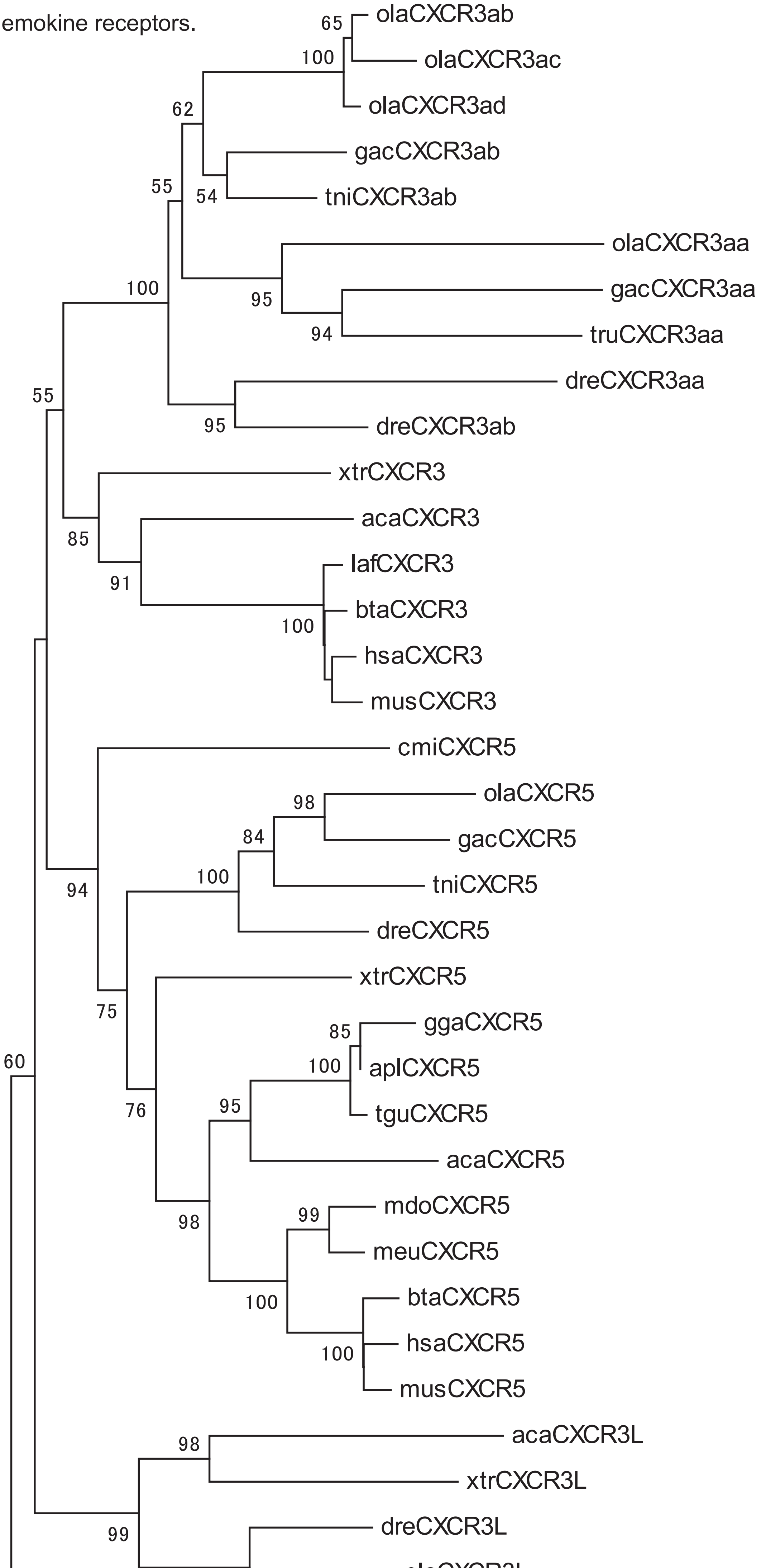

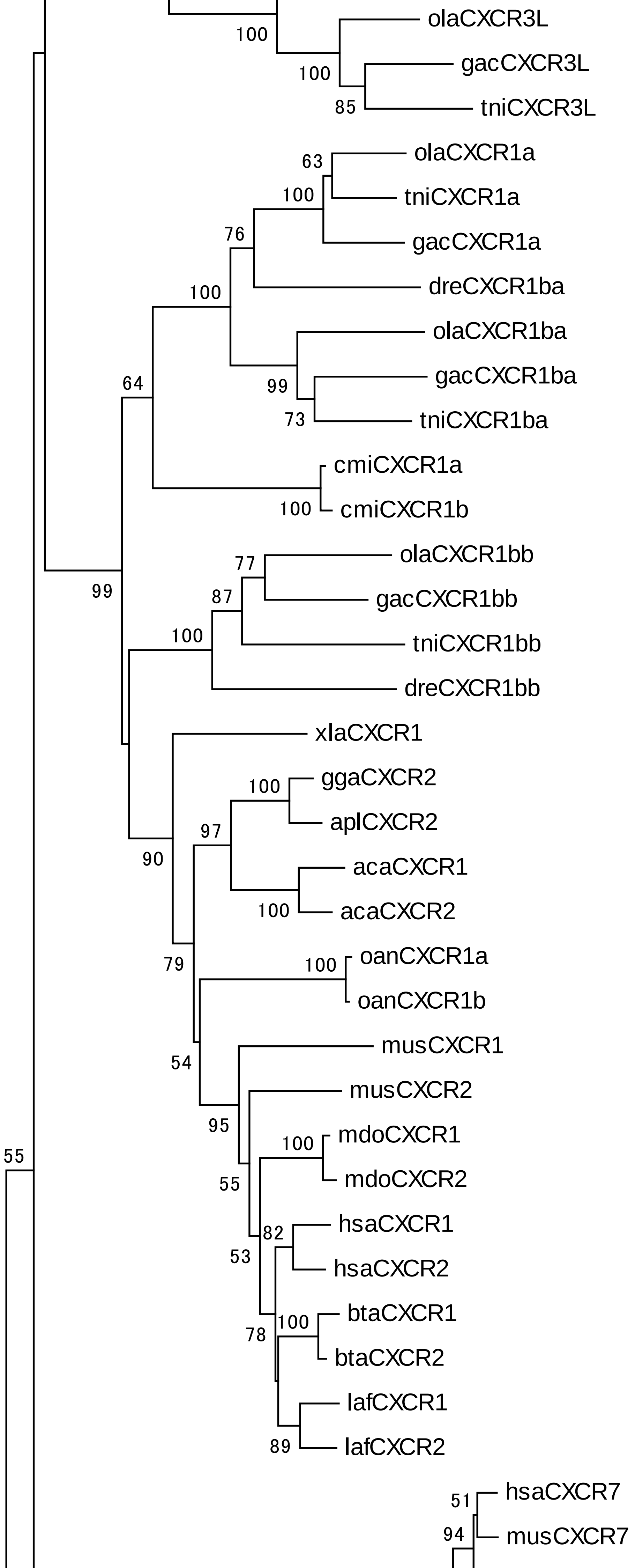
olaCXCR3L
gacCXCR3L
tniCXCR3L
olaCXCR1a
tniCXCR1a
gacCXCR1a
dreCXCR1ba
olaCXCR1ba
gacCXCR1ba
tniCXCR1ba
cmiCXCR1a
cmiCXCR1b
olaCXCR1bb
gacCXCR1bb
tniCXCR1bb
dreCXCR1bb
xlaCXCR1
ggaCXCR2
aplCXCR2
acaCXCR1
acaCXCR2
oanCXCR1a
oanCXCR1b
musCXCR1
musCXCR2
mdoCXCR1
mdoCXCR2
hsaCXCR1
hsaCXCR2
btaCXCR1
btaCXCR2
lafCXCR1
lafCXCR2
hsaCXCR7
musCXCR7
100
100
85
63
100
76
100
64
99
73
99
100
77
87
100
99
100
97
100
90
100
79
54
95
55
100
82
53
100
78
89
55
51
94

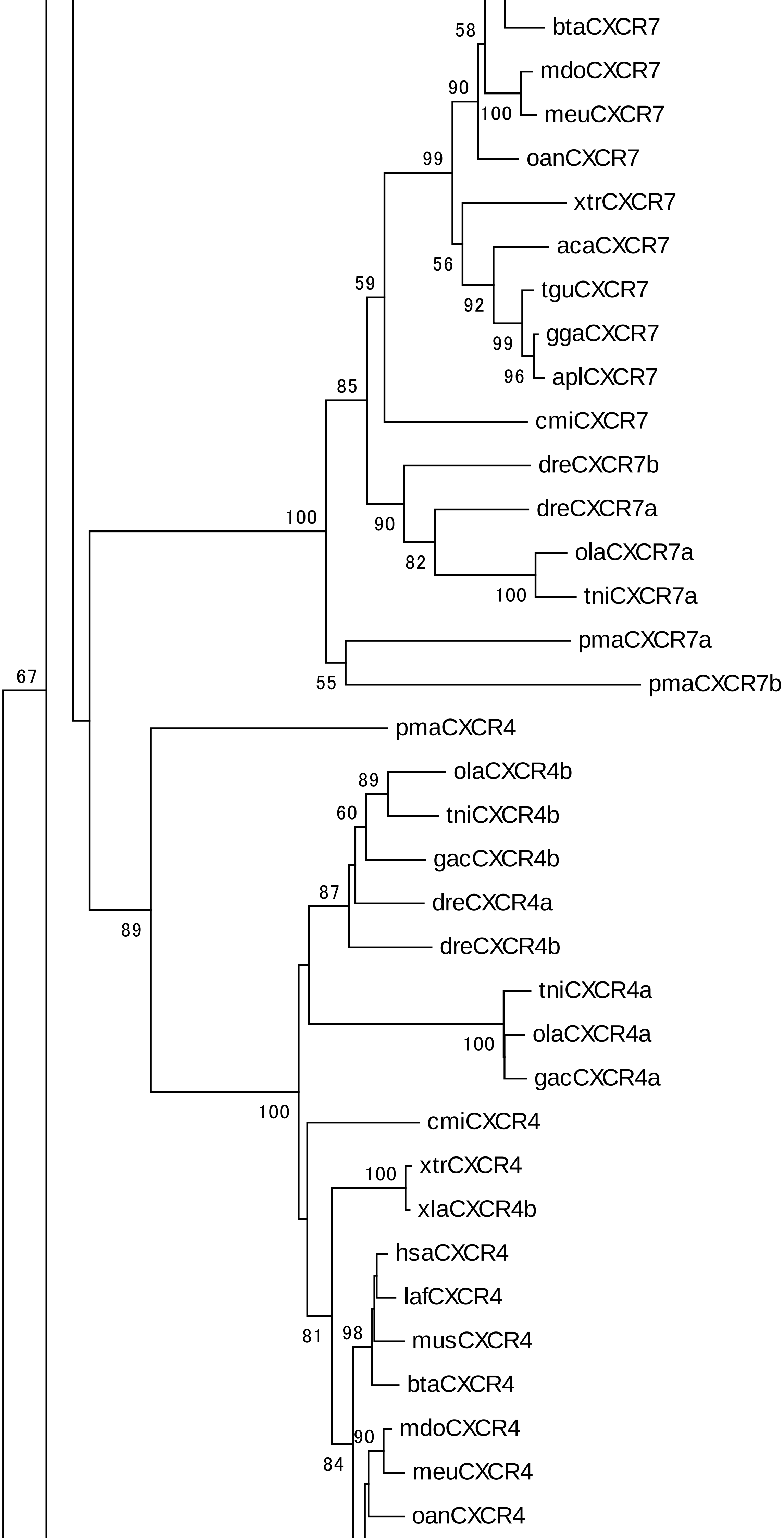
btaCXCR7
mdoCXCR7
meuCXCR7
oanCXCR7
xtrCXCR7
acaCXCR7
tguCXCR7
ggaCXCR7
aplCXCR7
cmiCXCR7
dreCXCR7b
dreCXCR7a
olaCXCR7a
tniCXCR7a
pmaCXCR7a
pmaCXCR7b
pmaCXCR4
olaCXCR4b
tniCXCR4b
gacCXCR4b
dreCXCR4a
dreCXCR4b
tniCXCR4a
olaCXCR4a
gacCXCR4a
cmiCXCR4
xtrCXCR4
xlaCXCR4b
hsaCXCR4
lafCXCR4
musCXCR4
btaCXCR4
mdoCXCR4
meuCXCR4
oanCXCR4
58
90
100
99
56
92
99
96
59
85
100
90
82
100
55
67
89
60
87
89
100
100
100
81
98
90
84

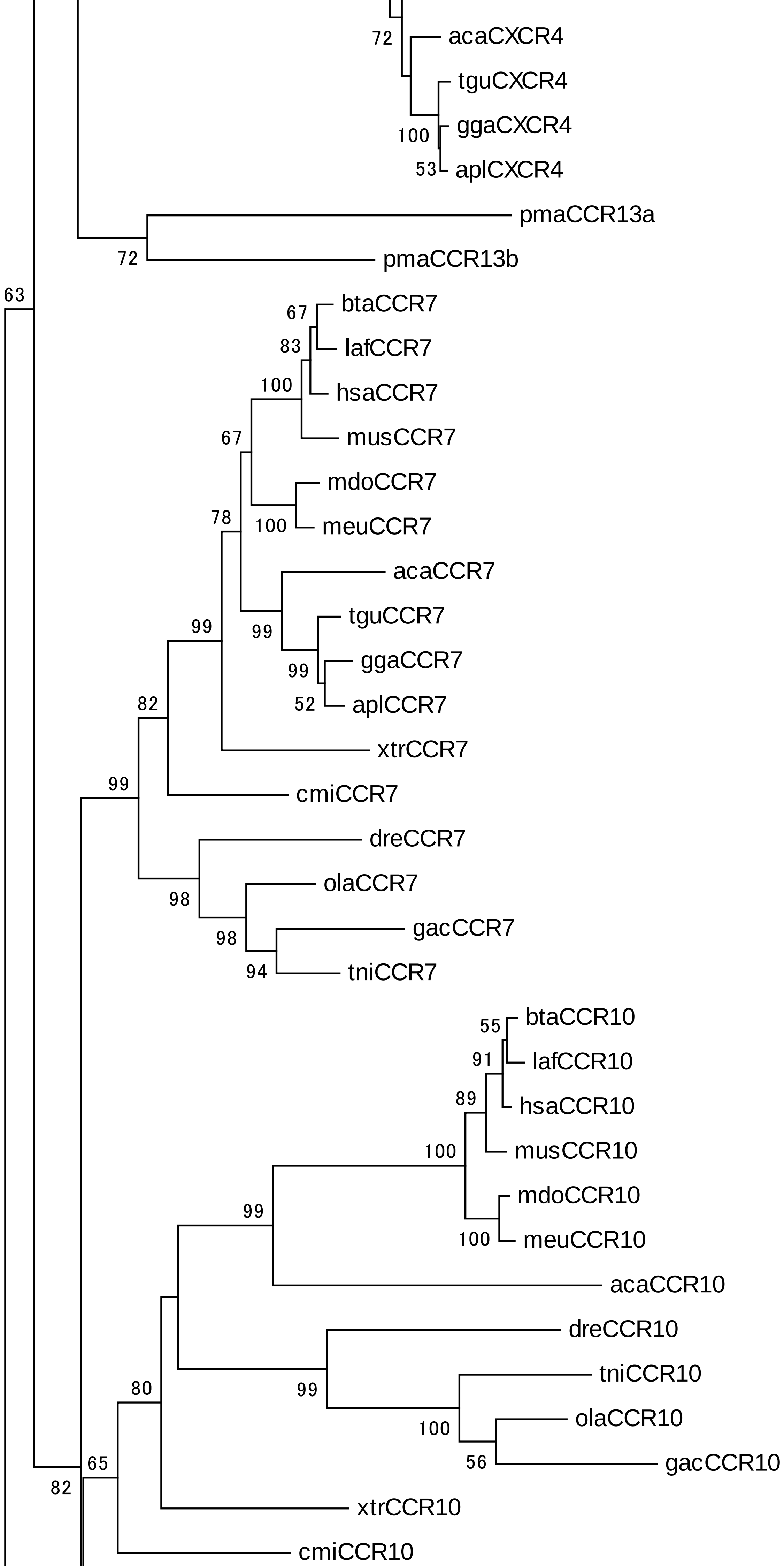

acaCXCR4
tguCXCR4
ggaCXCR4
aplCXCR4
pmaCCR13a
pmaCCR13b
btaCCR7
lafCCR7
hsaCCR7
musCCR7
mdoCCR7
meuCCR7
acaCCR7
tguCCR7
ggaCCR7
aplCCR7
xtrCCR7
cmiCCR7
dreCCR7
olaCCR7
gacCCR7
tniCCR7
btaCCR10
lafCCR10
hsaCCR10
musCCR10
mdoCCR10
meuCCR10
acaCCR10
dreCCR10
tniCCR10
olaCCR10
gacCCR10
xtrCCR10
cmiCCR10

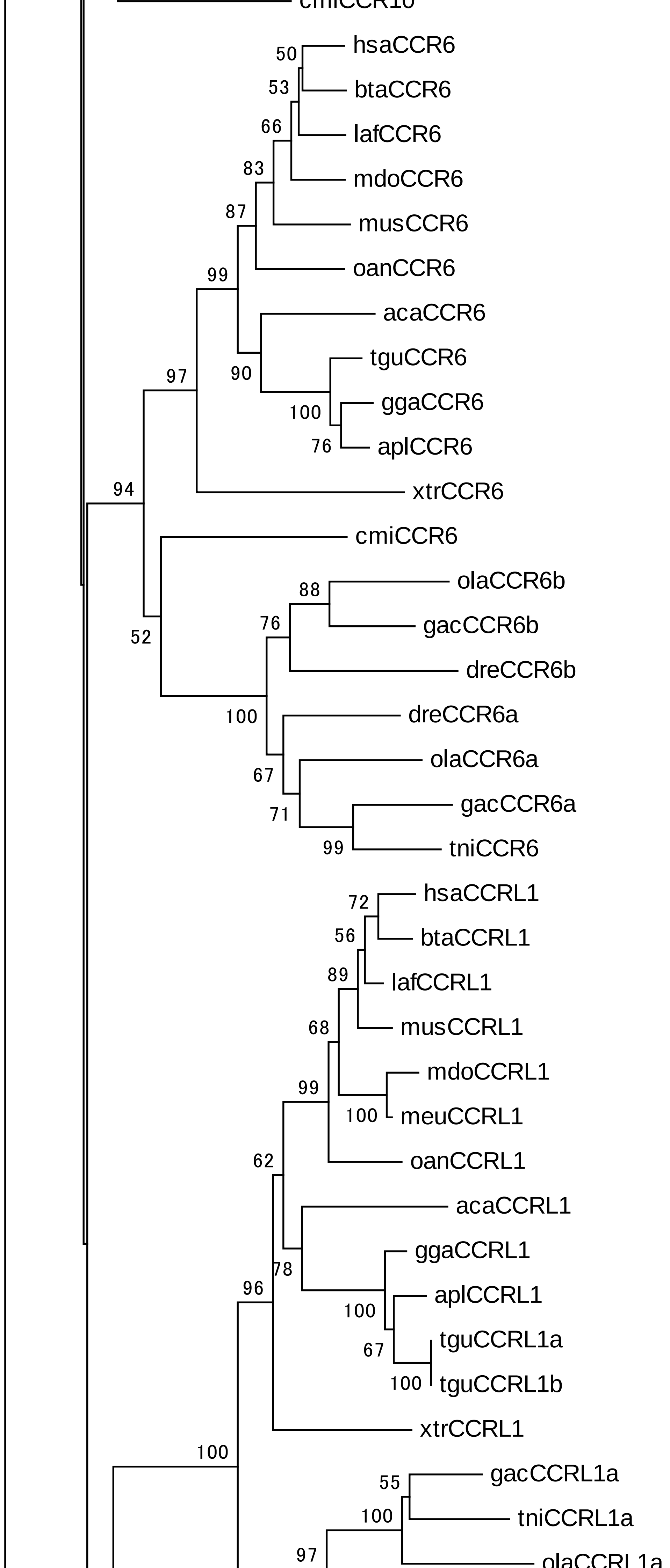
hsaCCR6
btaCCR6
lafCCR6
mdoCCR6
musCCR6
oanCCR6
acaCCR6
tguCCR6
ggaCCR6
aplCCR6
xtrCCR6
cmiCCR6
olaCCR6b
gacCCR6b
dreCCR6b
dreCCR6a
olaCCR6a
gacCCR6a
tniCCR6
hsaCCRL1
btaCCRL1
lafCCRL1
musCCRL1
mdoCCRL1
meuCCRL1
oanCCRL1
acaCCRL1
ggaCCRL1
aplCCRL1
tguCCRL1a
tguCCRL1b
xtrCCRL1
gacCCRL1a
tniCCRL1a

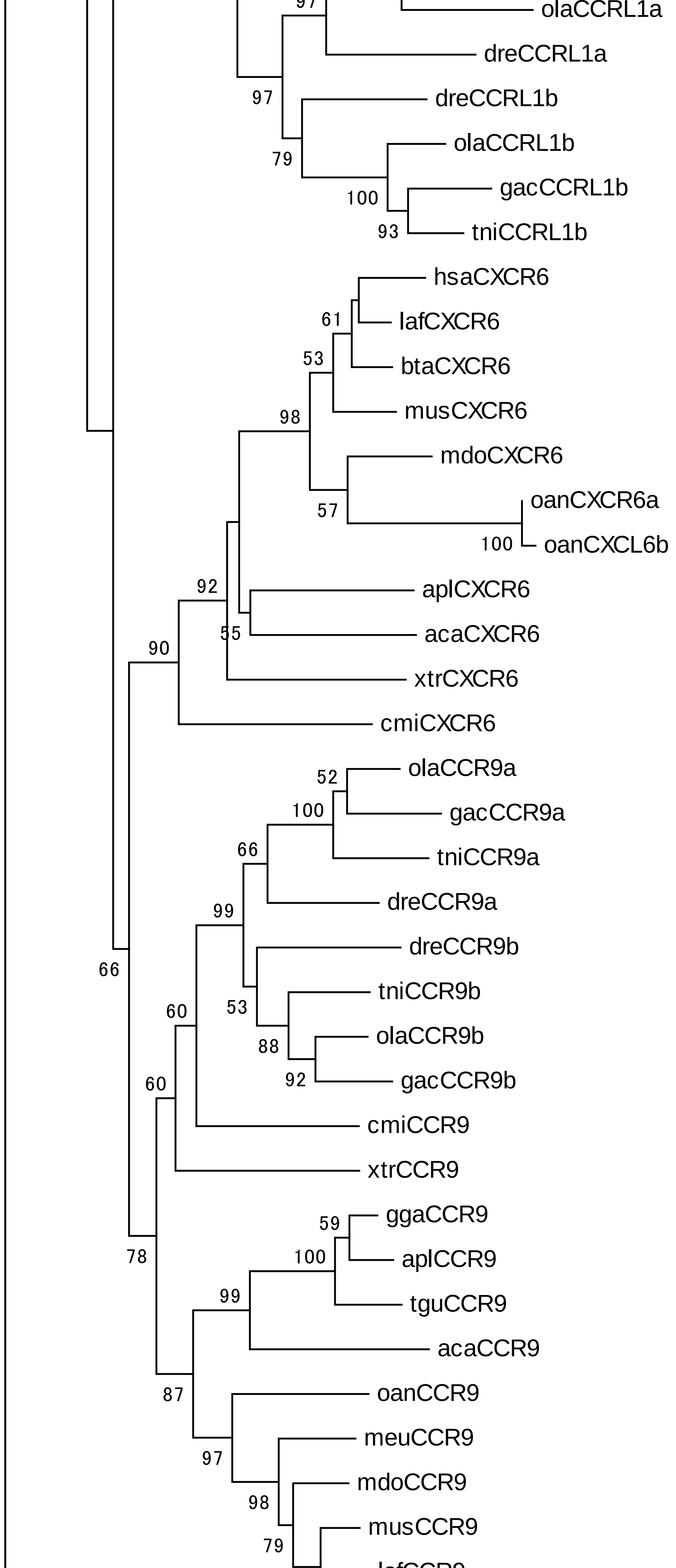
olaCCRL1a
dreCCRL1a
dreCCRL1b
olaCCRL1b
gacCCRL1b
tniCCRL1b
hsaCXCR6
lafCXCR6
btaCXCR6
musCXCR6
mdoCXCR6
oanCXCR6a
oanCXCL6b
aplCXCR6
acaCXCR6
xtrCXCR6
cmiCXCR6
olaCCR9a
gacCCR9a
tniCCR9a
dreCCR9a
dreCCR9b
tniCCR9b
olaCCR9b
gacCCR9b
cmiCCR9
xtrCCR9
ggaCCR9
aplCCR9
tguCCR9
acaCCR9
oanCCR9
meuCCR9
mdoCCR9
musCCR9

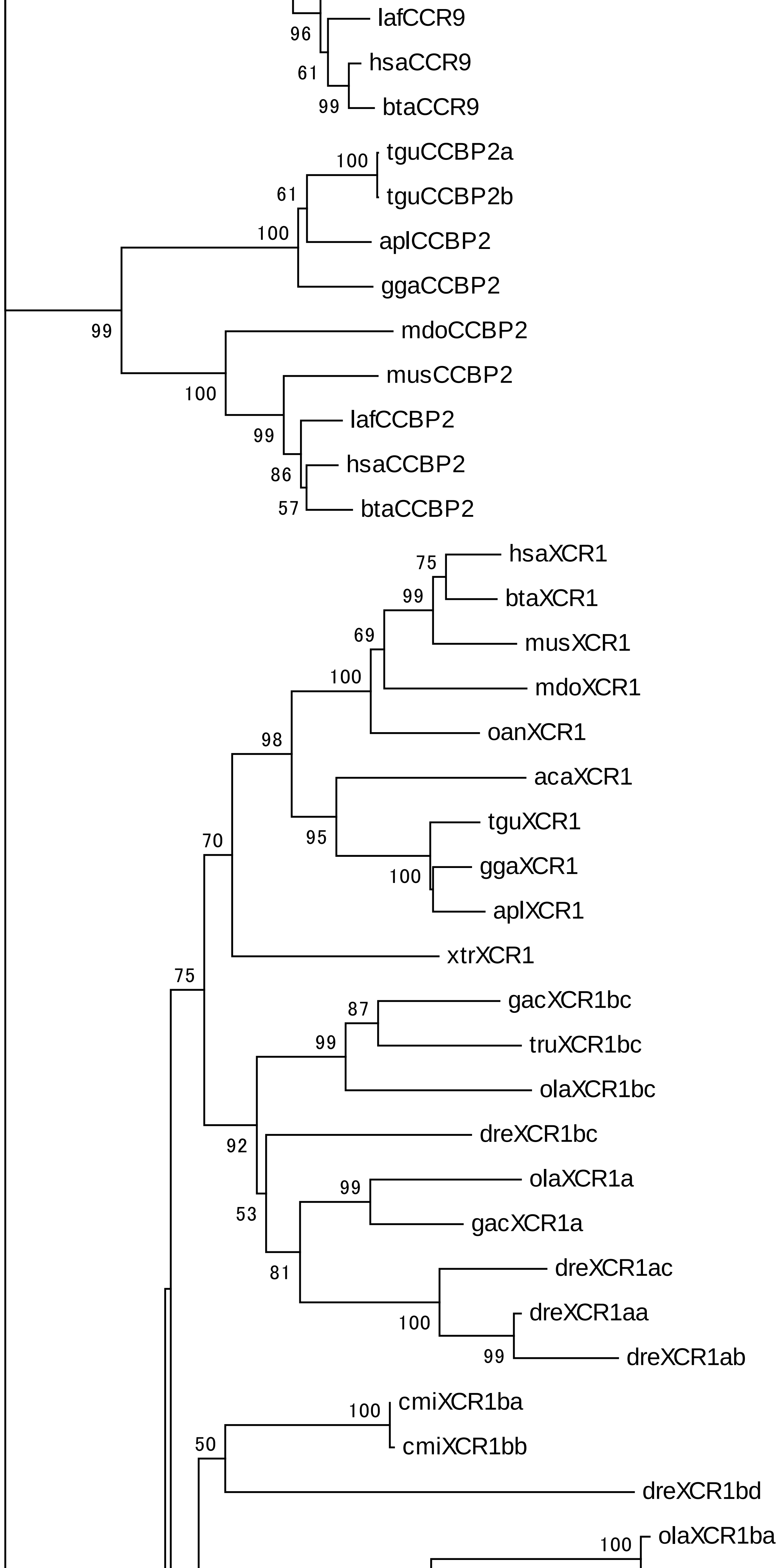

lafCCR9
96
61
hsaCCR9
99
btaCCR9
100
tguCCBP2a
tguCCBP2b
61
100
aplCCBP2
ggaCCBP2
99
mdoCCBP2
musCCBP2
100
99
lafCCBP2
86
hsaCCBP2
57
btaCCBP2
75
hsaXCR1
btaXCR1
99
69
musXCR1
100
mdoXCR1
oanXCR1
98
acaXCR1
95
tguXCR1
100
ggaXCR1
aplXCR1
70
xtrXCR1
75
87
gacXCR1bc
99
truXCR1bc
olaXCR1bc
92
dreXCR1bc
99
olaXCR1a
53
gacXCR1a
81
dreXCR1ac
100
dreXCR1aa
99
dreXCR1ab
100
cmiXCR1ba
50
cmiXCR1bb
dreXCR1bd
100
olaXCR1ba

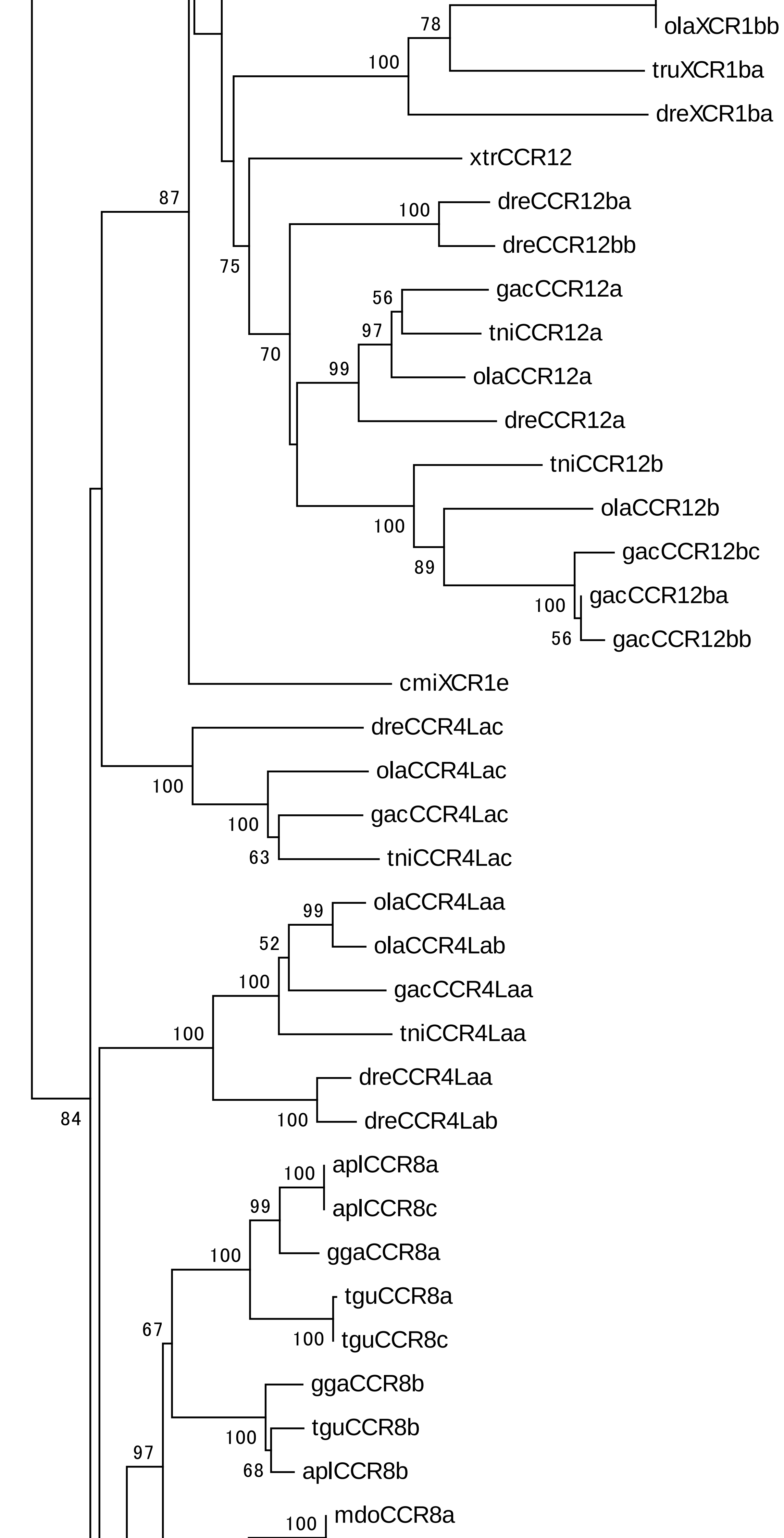

olaXCR1bb
truXCR1ba
dreXCR1ba
xtrCCR12
dreCCR12ba
dreCCR12bb
gacCCR12a
tniCCR12a
olaCCR12a
dreCCR12a
tniCCR12b
olaCCR12b
gacCCR12bc
gacCCR12ba
gacCCR12bb
cmiXCR1e
dreCCR4Lac
olaCCR4Lac
gacCCR4Lac
tniCCR4Lac
olaCCR4Laa
olaCCR4Lab
gacCCR4Laa
tniCCR4Laa
dreCCR4Laa
dreCCR4Lab
aplCCR8a
aplCCR8c
ggaCCR8a
tguCCR8a
tguCCR8c
ggaCCR8b
tguCCR8b
aplCCR8b
mdoCCR8a

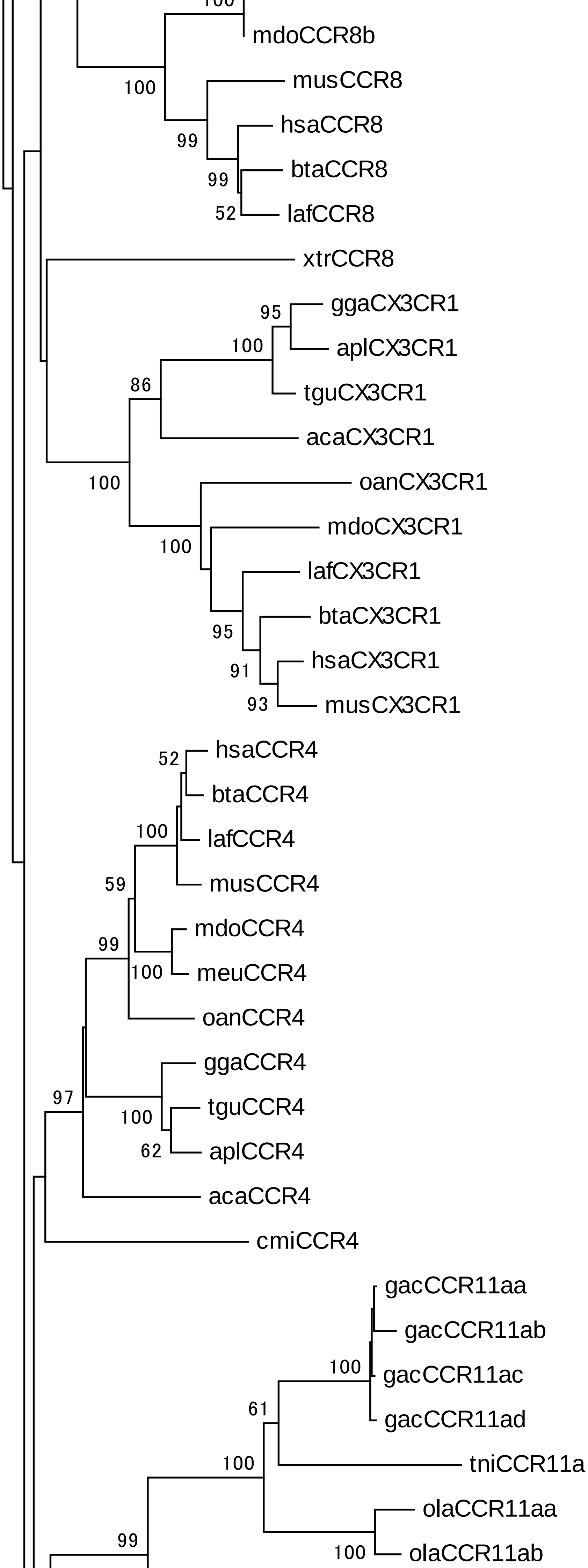

100
mdoCCR8b
100
musCCR8
hsaCCR8
99
btaCCR8
99
lafCCR8
52
xtrCCR8
95
ggaCX3CR1
100
aplCX3CR1
tguCX3CR1
86
acaCX3CR1
100
oanCX3CR1
mdoCX3CR1
100
lafCX3CR1
btaCX3CR1
95
hsaCX3CR1
91
musCX3CR1
93
52
hsaCCR4
btaCCR4
100
lafCCR4
59
musCCR4
mdoCCR4
99
100
meuCCR4
oanCCR4
ggaCCR4
tguCCR4
97
100
62
aplCCR4
acaCCR4
cmiCCR4
gacCCR11aa
gacCCR11ab
100
gacCCR11ac
61
gacCCR11ad
100
tniCCR11a
olaCCR11aa
99
100
olaCCR11ab

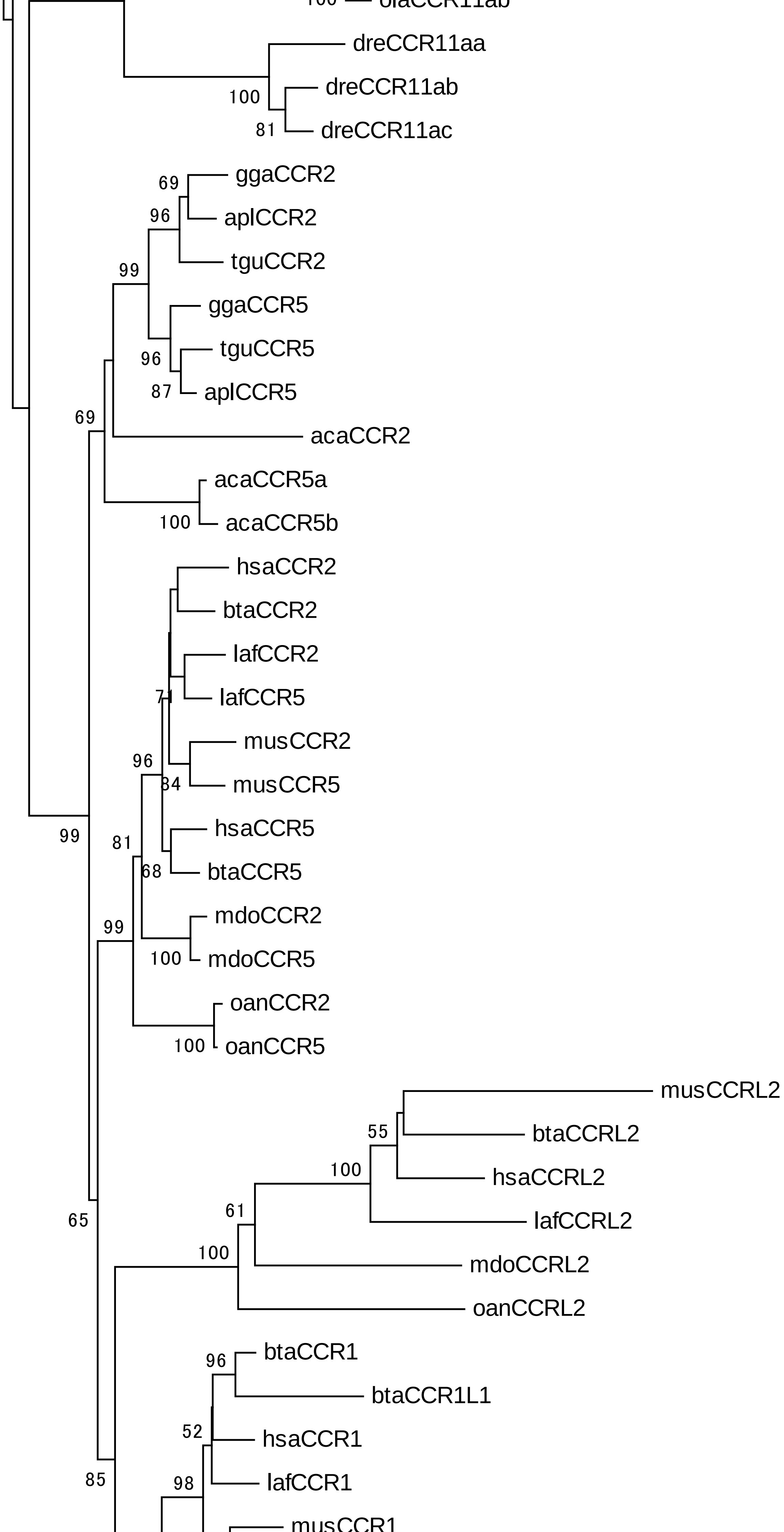
dreCCR11aa
100
dreCCR11ab
81
dreCCR11ac
69
ggaCCR2
96
aplCCR2
99
tguCCR2
ggaCCR5
96
tguCCR5
87
aplCCR5
69
acaCCR2
acaCCR5a
100
acaCCR5b
hsaCCR2
btaCCR2
lafCCR2
71
lafCCR5
musCCR2
96
84
musCCR5
99
81
hsaCCR5
68
btaCCR5
mdoCCR2
99
100
mdoCCR5
oanCCR2
100
oanCCR5
musCCRL2
55
btaCCRL2
100
hsaCCRL2
61
lafCCRL2
65
100
mdoCCRL2
oanCCRL2
96
btaCCR1
btaCCR1L1
52
hsaCCR1
85
98
lafCCR1
musCCR1

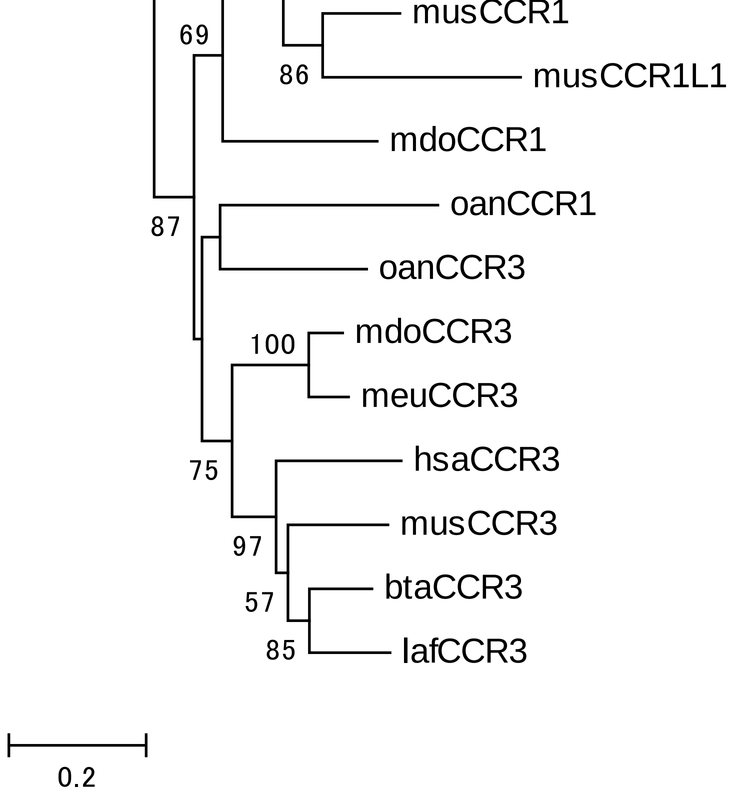

musCCR1
69
86
musCCR1L1
mdoCCR1
oanCCR1
87
oanCCR3
100
mdoCCR3
meuCCR3
75
hsaCCR3
musCCR3
97
57
btaCCR3
85
lafCCR3
0.2
