# Supplementary material for: Systematic classification of vertebrate chemokines based on conserved synteny and evolutionary history
Source: Genes Cells. 2012 Nov 12;18(1):1–16. doi: 10.1111/gtc.12013 (PMC3568907; doi:10.1111/gtc.12013)
Supplement: Supplementary file 9 [file gtc0018-0001-SD5.pdf]

Fig. S5
Conserved synteny dot plots of chemokine and chemokine receptor genes.

The plots were drawn using the Synteny Database (http://teleost.cs.uoregon.edu/synteny_db/). Orthologues of genes on the teleost (zebrafish, medaka or Tetraodon) chromosomes are plotted as red crosses on their respective chromosomes, but ordered relative to the position of the gene on the human chromosome. The teleost chromosomes containing chemokine or chemokine regions were derived from a part of the indicated teleost protochromosome. Official gene symbols are in bold italic letters.

A. Chemokine genes.

1) CXCL8

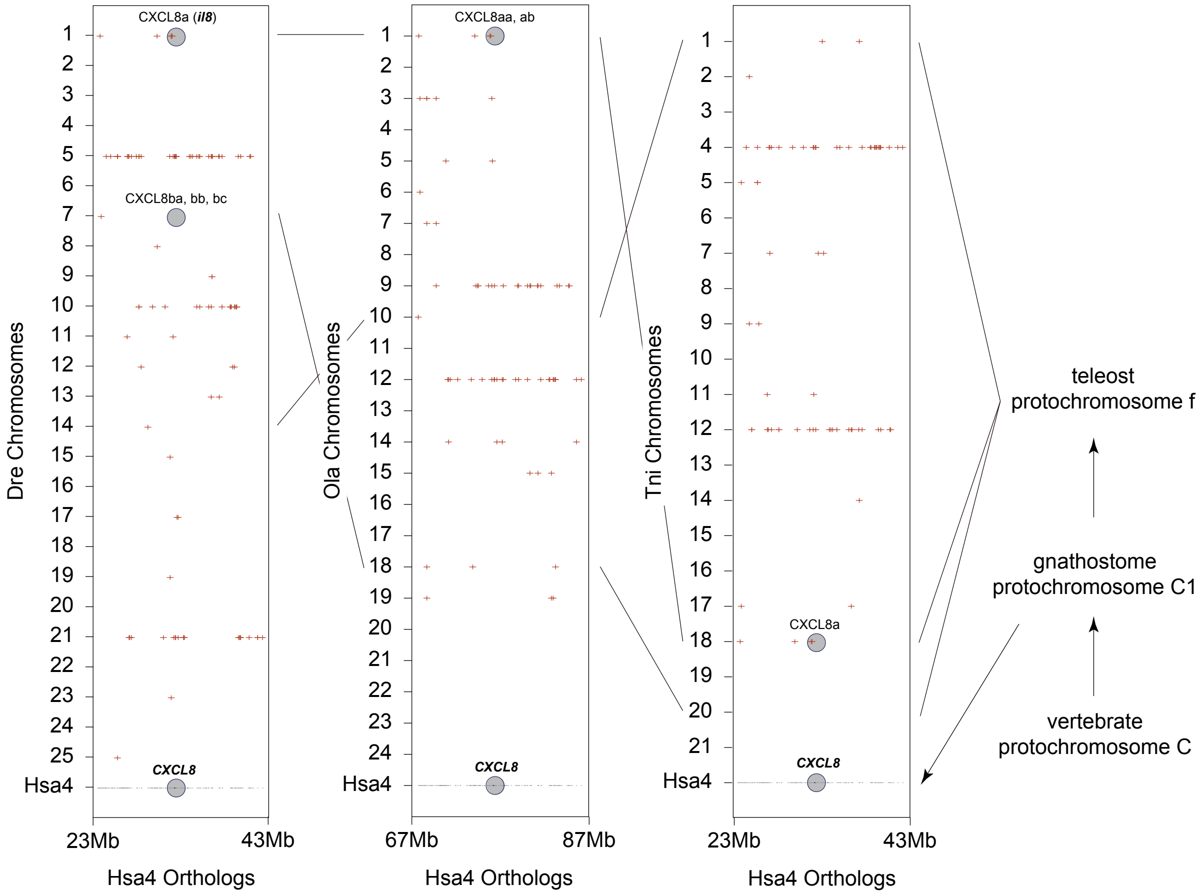

2) CXCL11, CXCL13

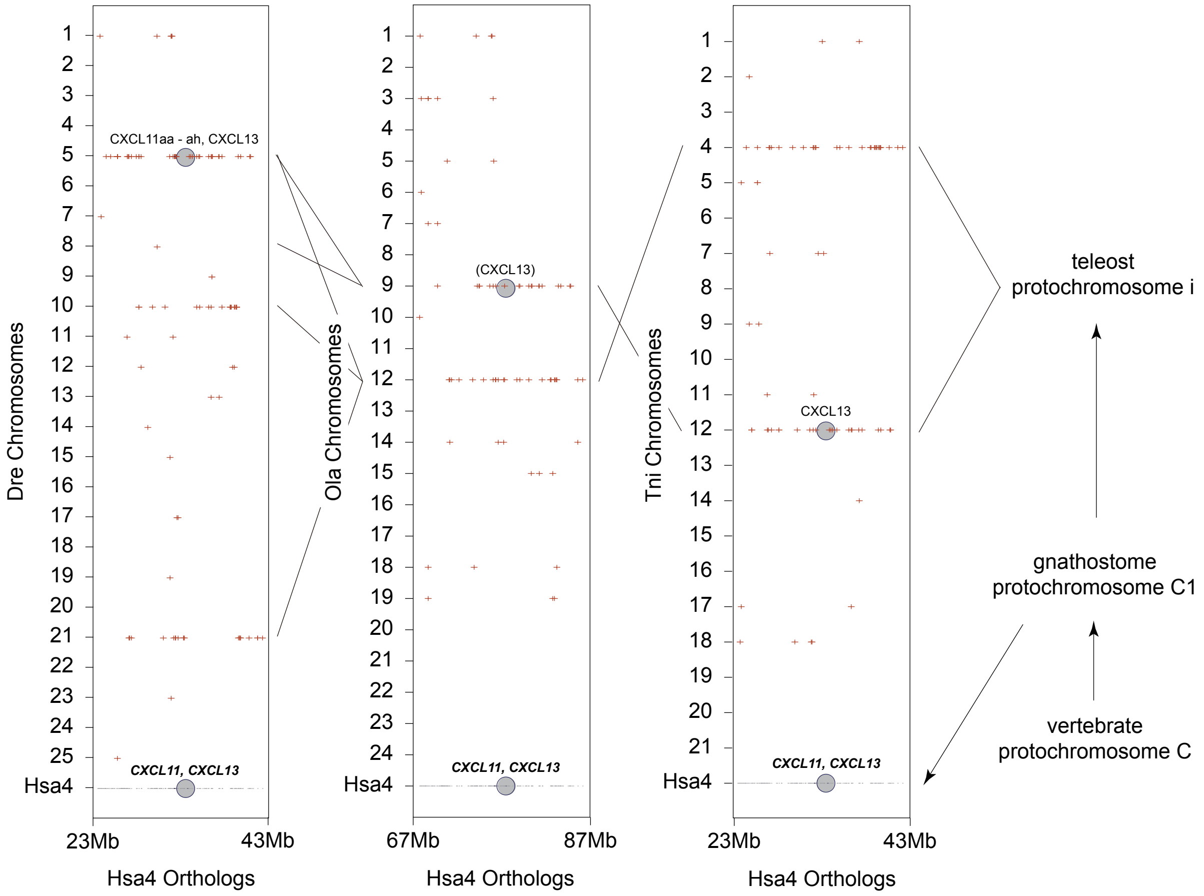

3) CXCL12

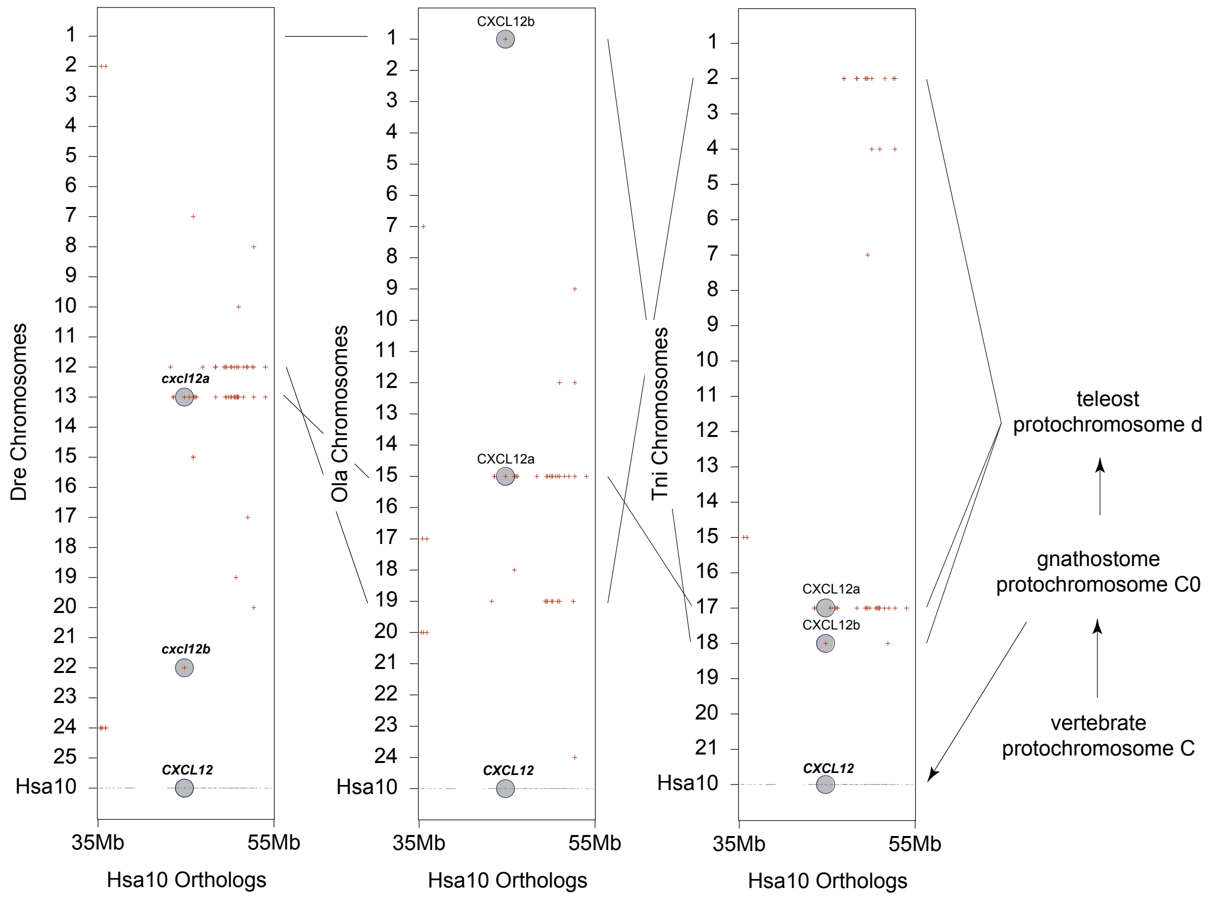

4) CXCL14

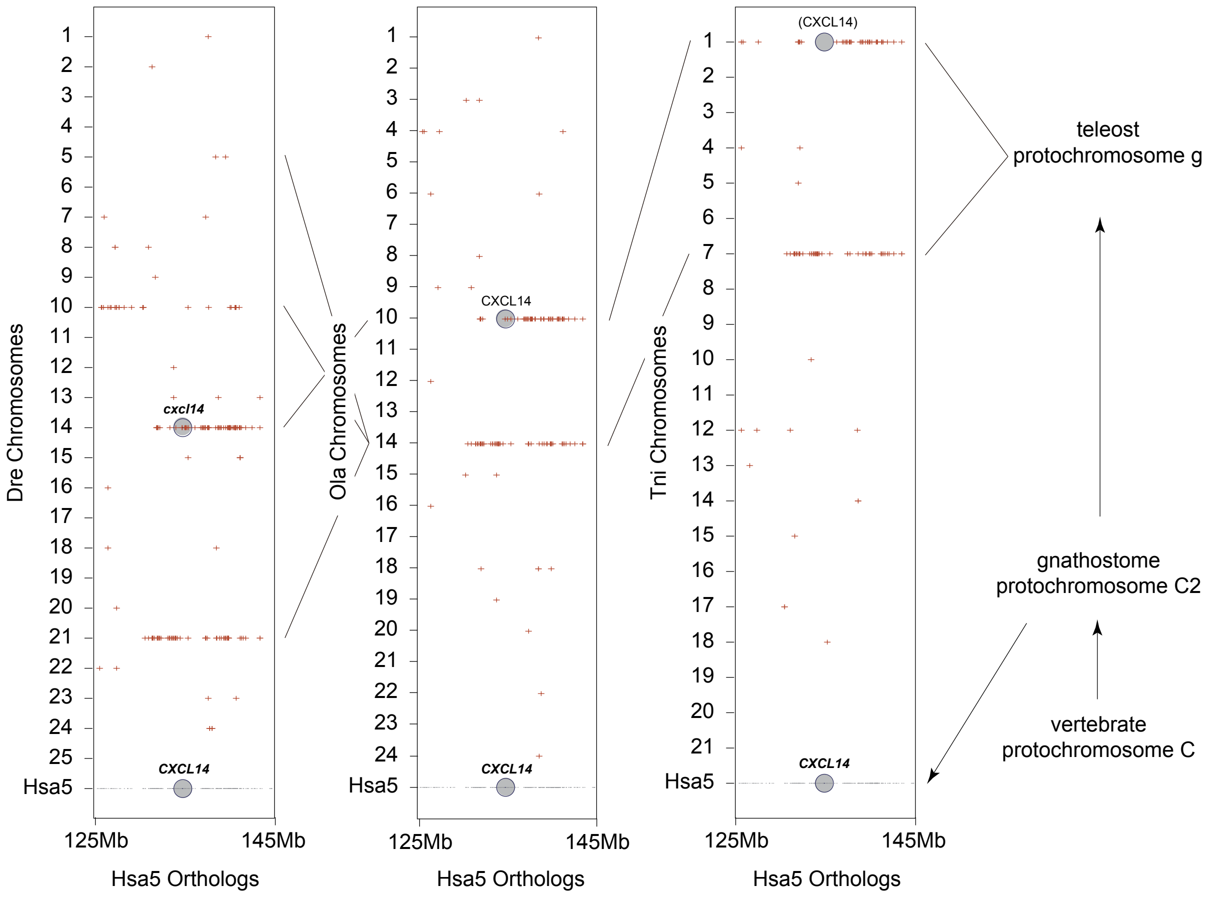

5) CCL19, CCL27

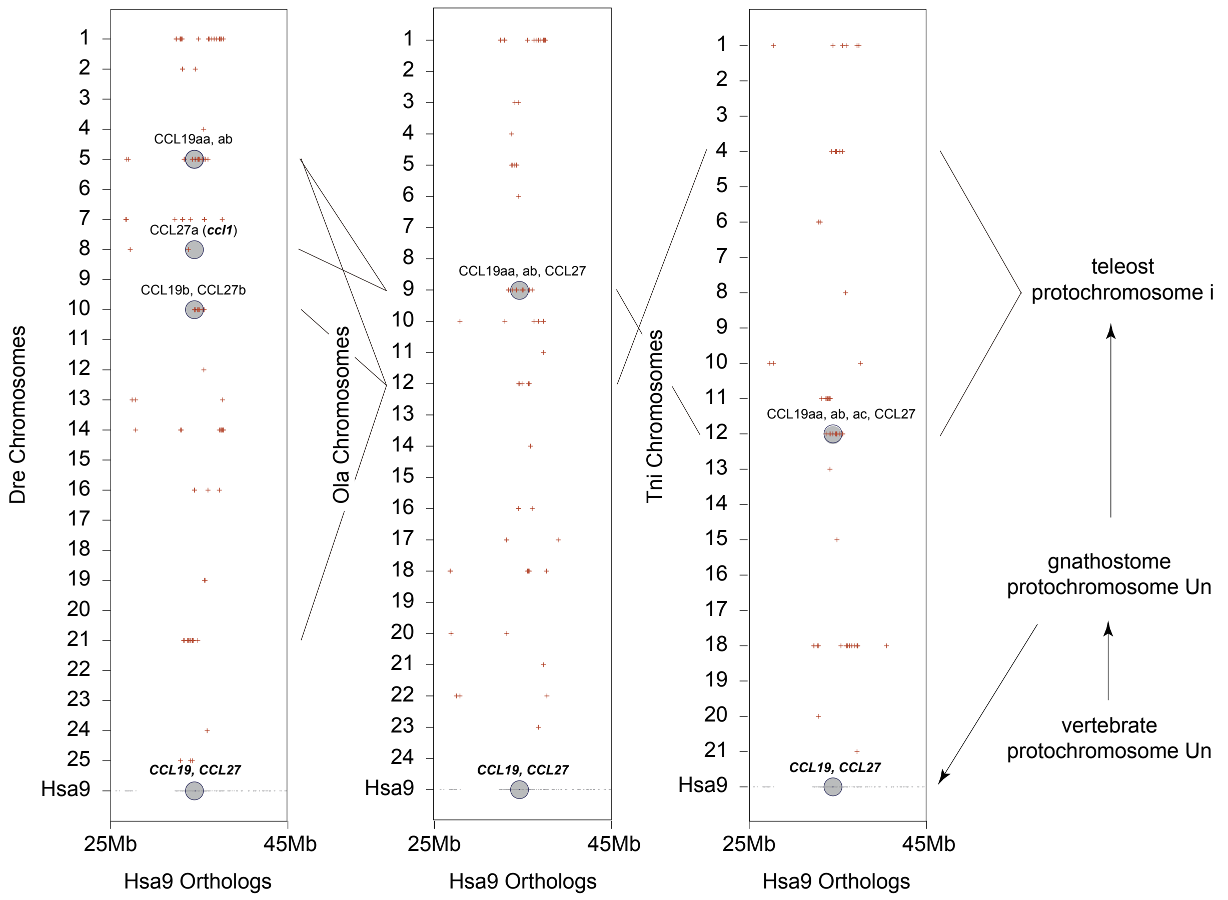

6) CCL20

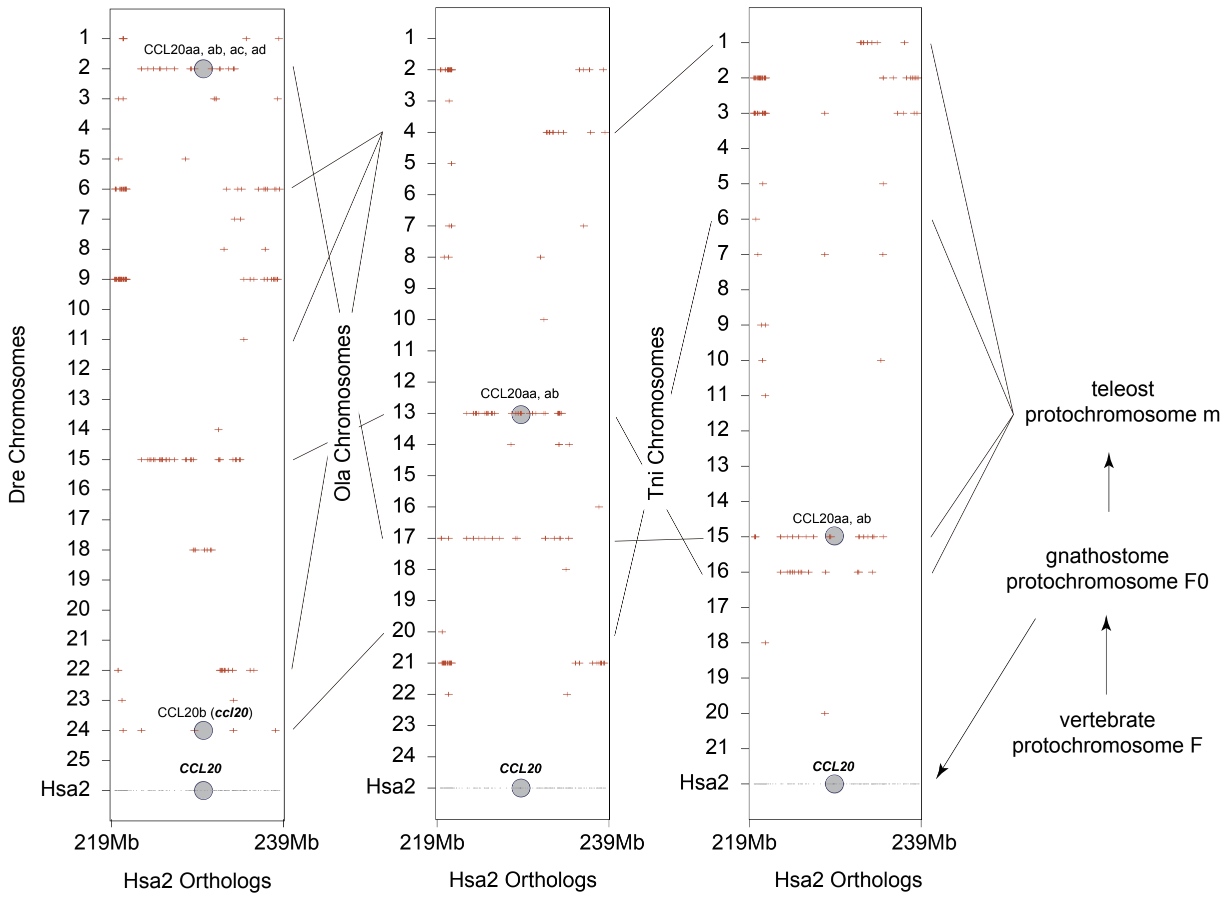

7) CCL25

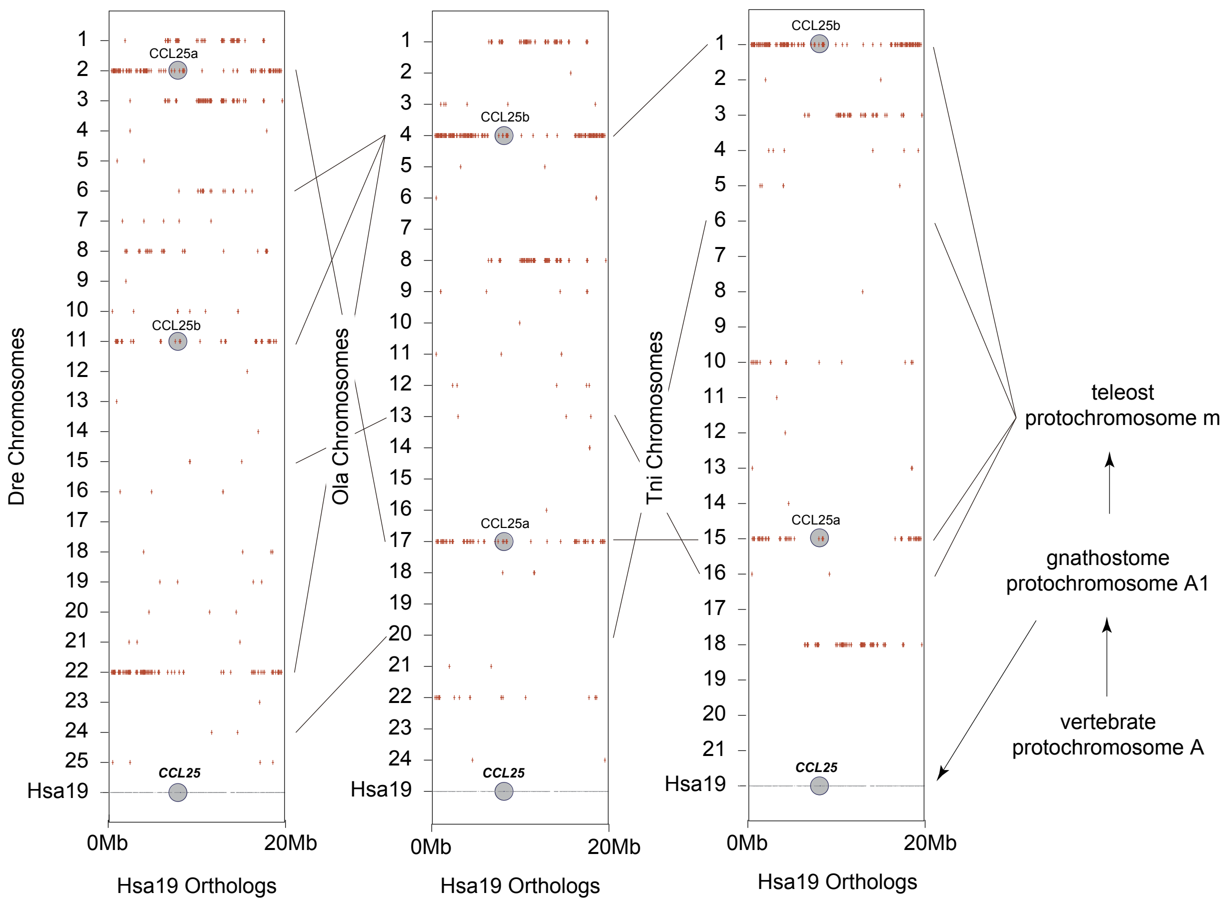

Dre Chromosomes
Ola Chromosomes
Tni Chromosomes
CCL25a
CCL25b
CCL25
Hsa19
0Mb
20Mb
Hsa19 Orthologs
teleost protochromosome m
gnathostome protochromosome A1
vertebrate protochromosome A

B. Chemokine receptor genes.

1) CXCR1, CXCR2

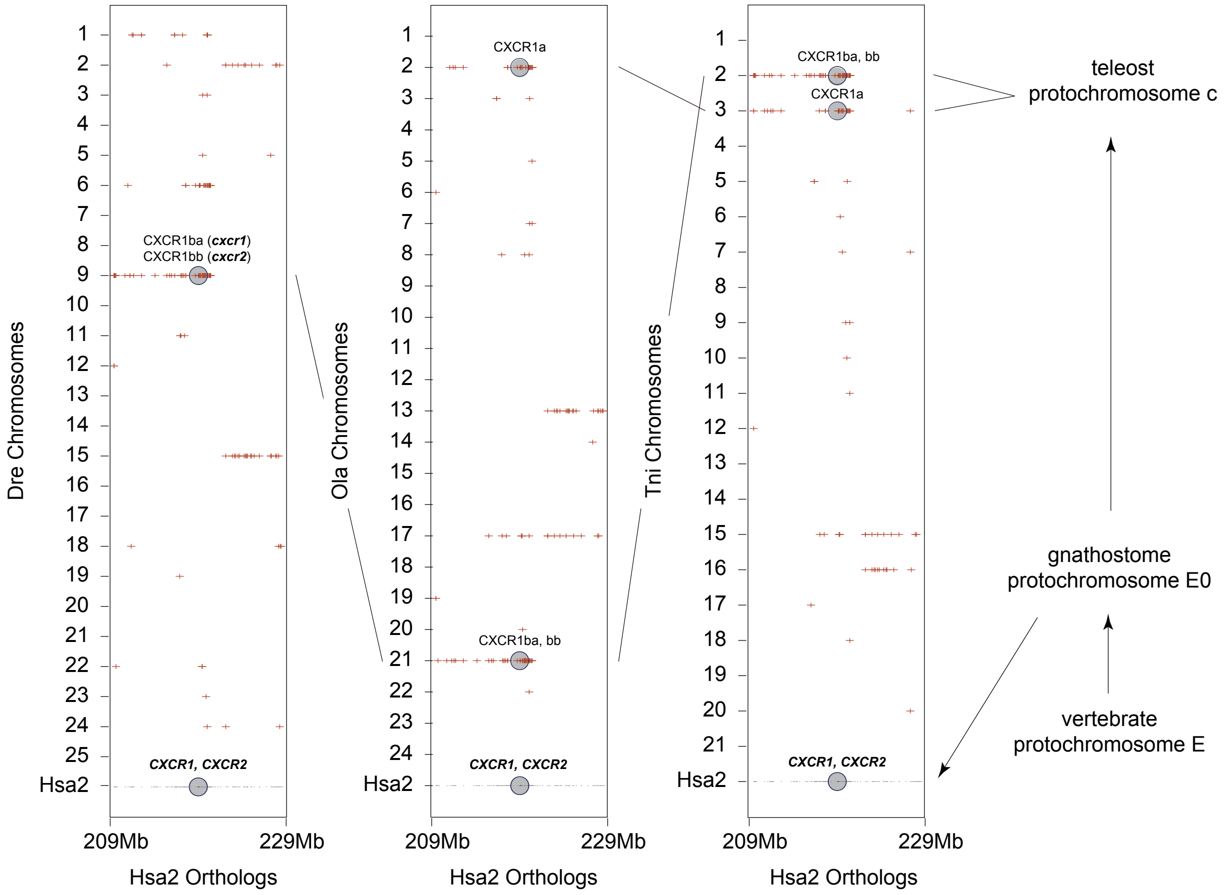

2) CXCR3

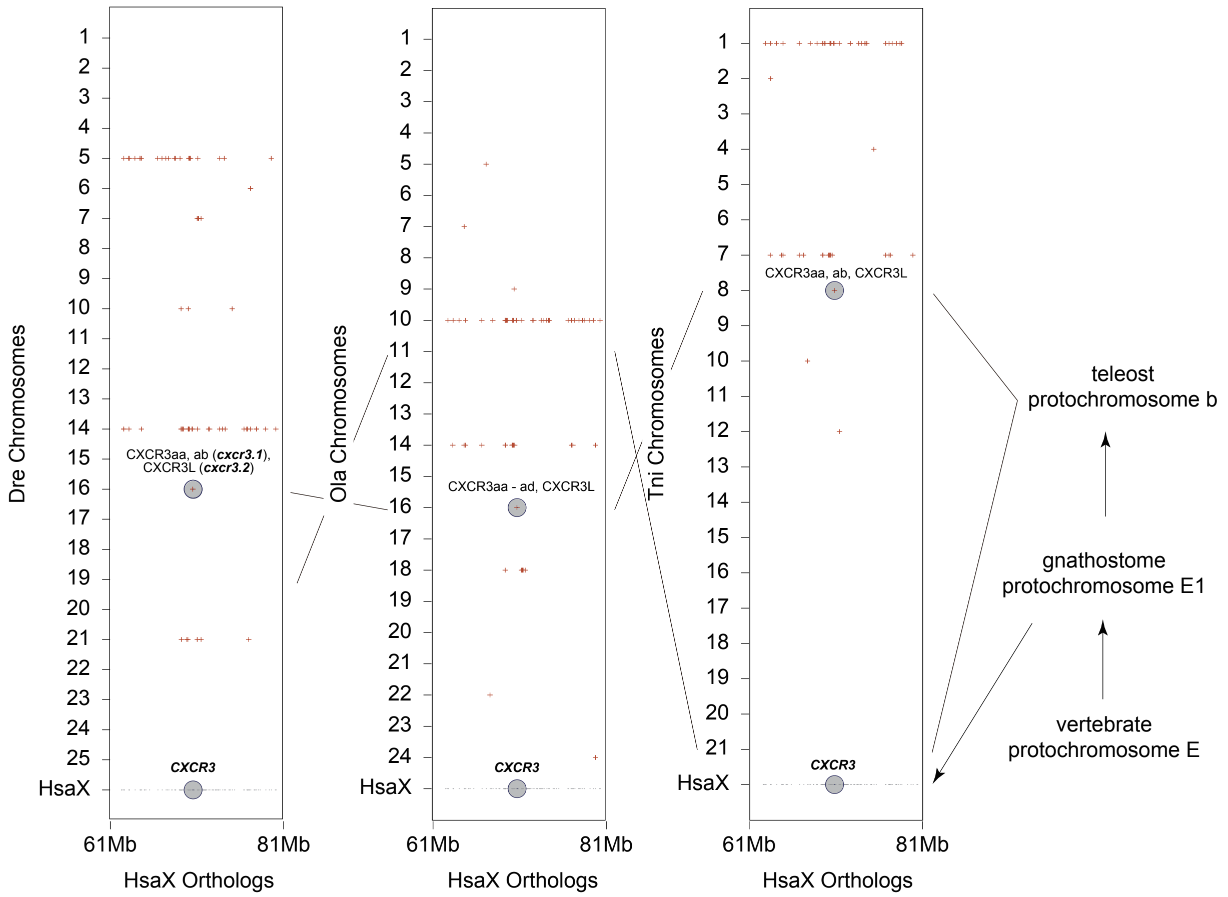

3) CXCR4

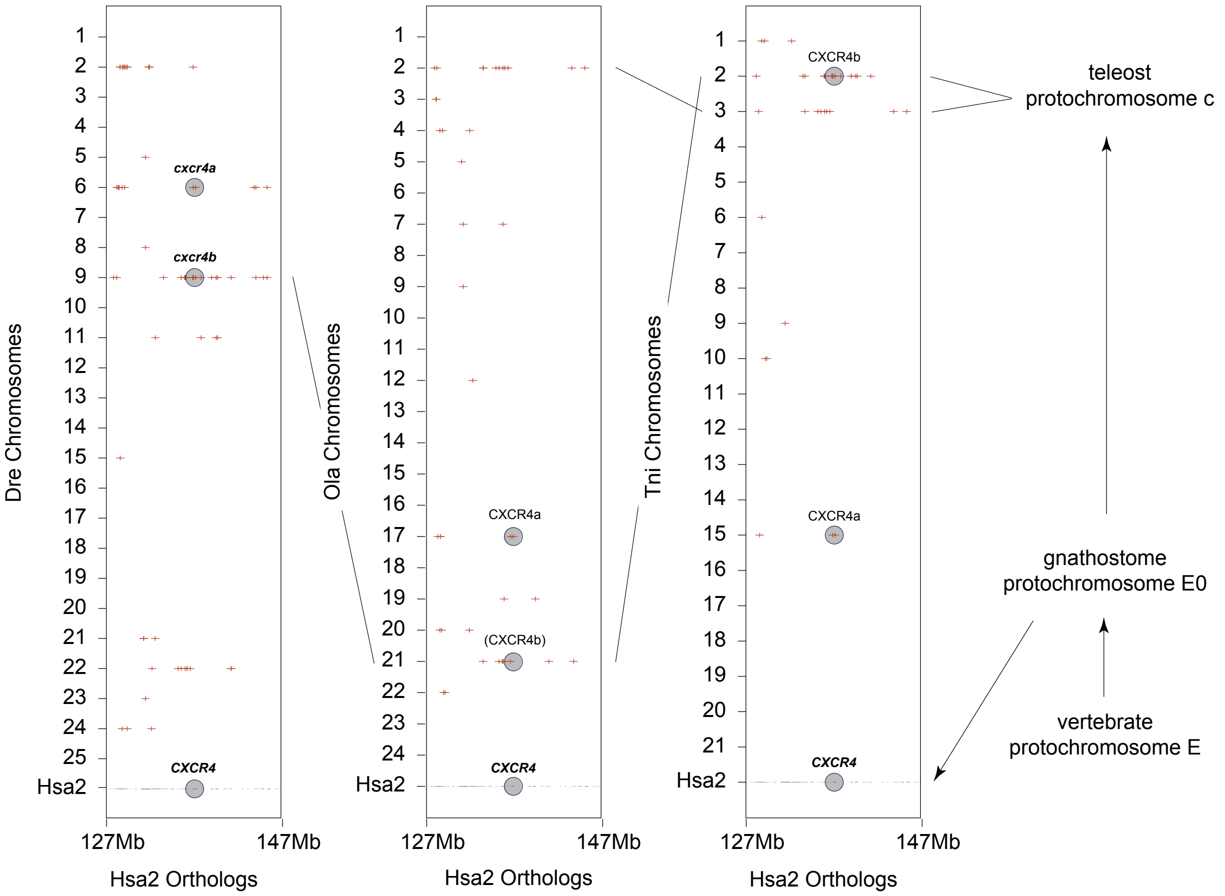

4) CXCR5

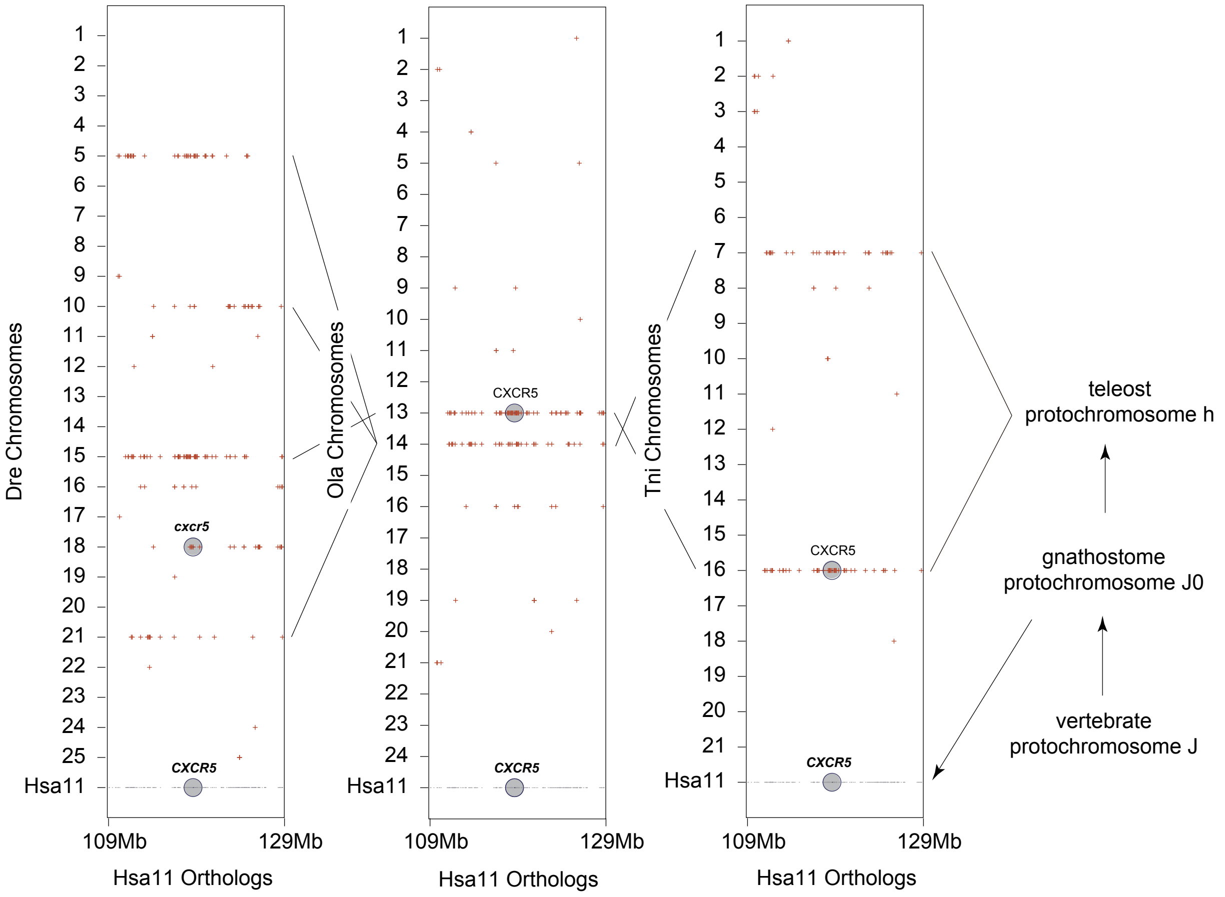

5) CXCR7

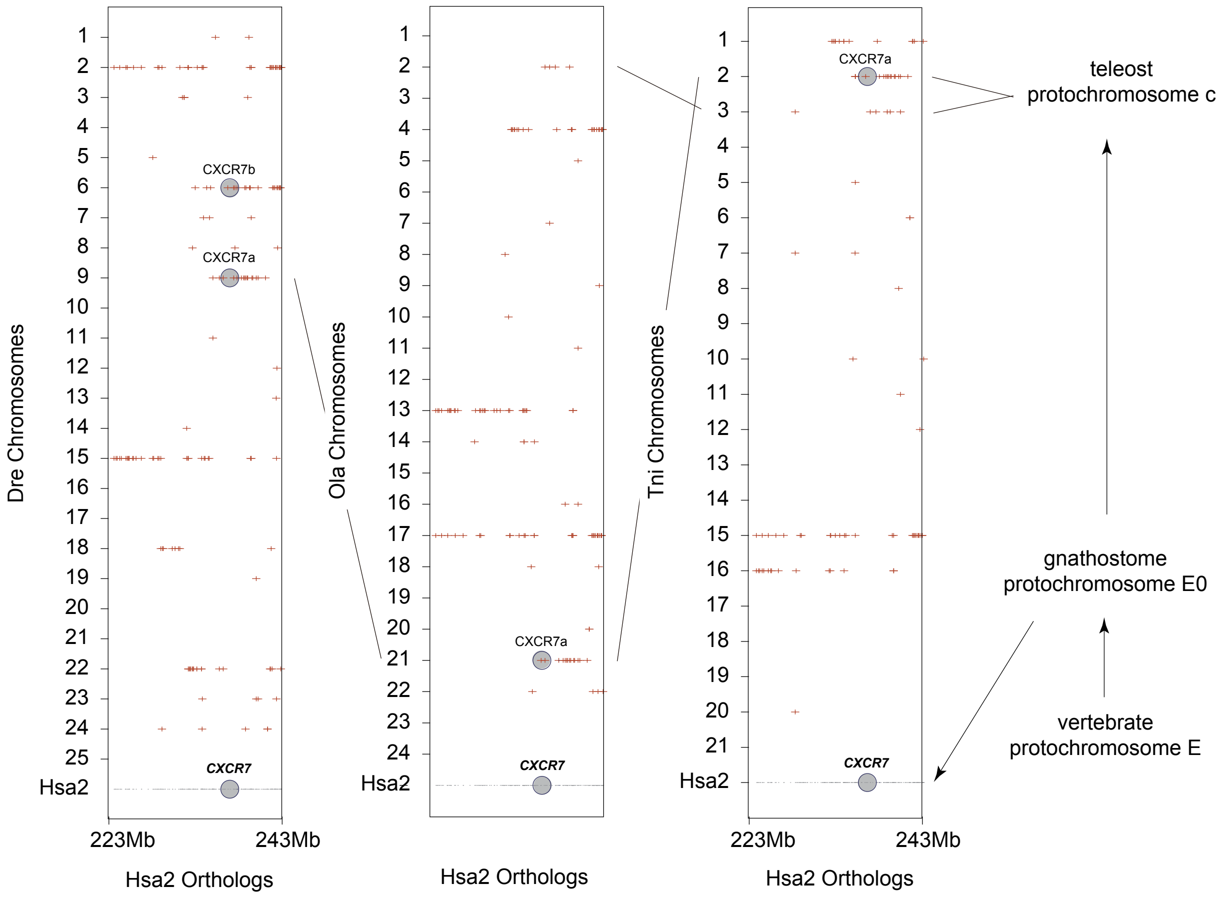

6) CCR4, CCR9, XCR1

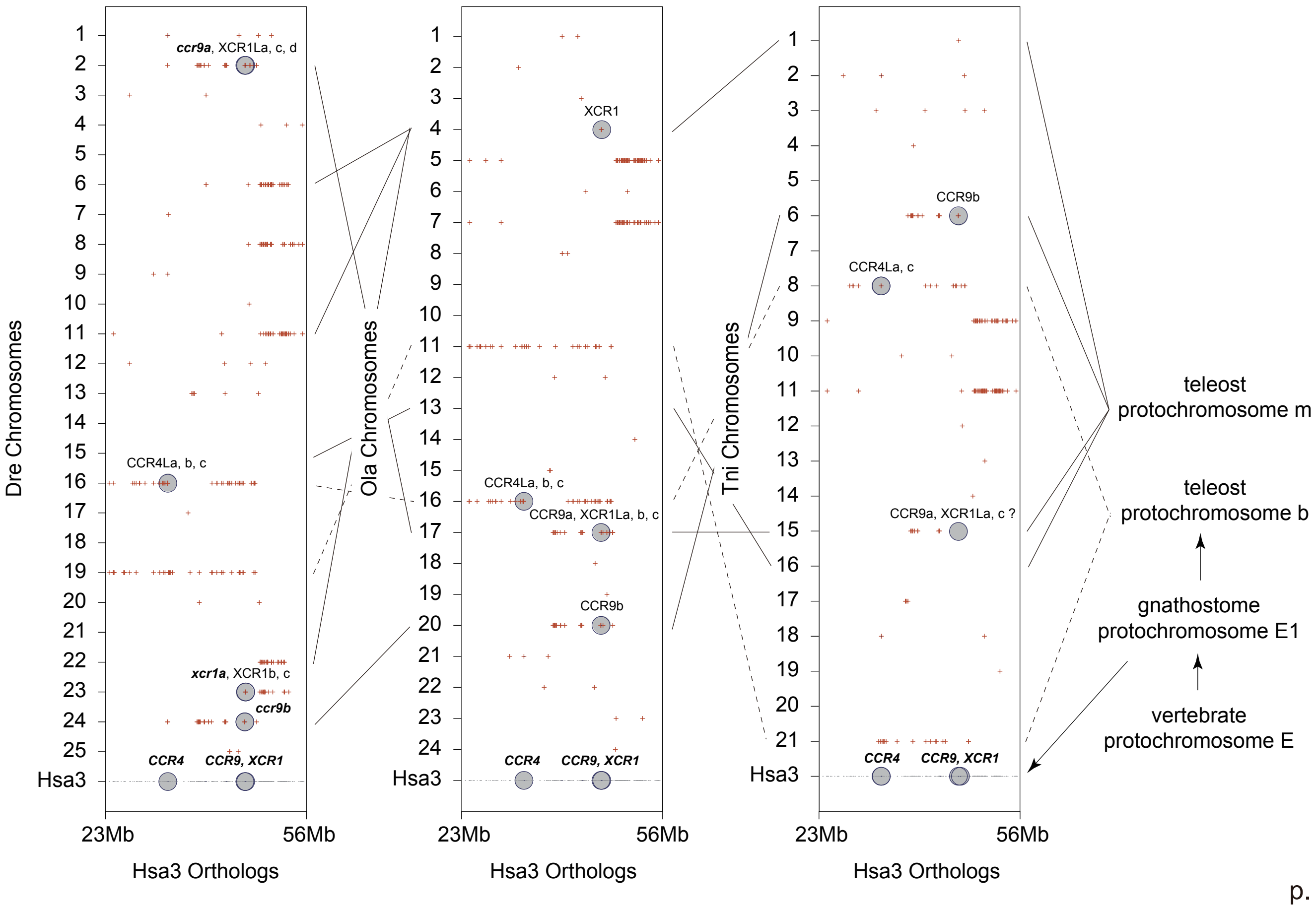

7) CCR6

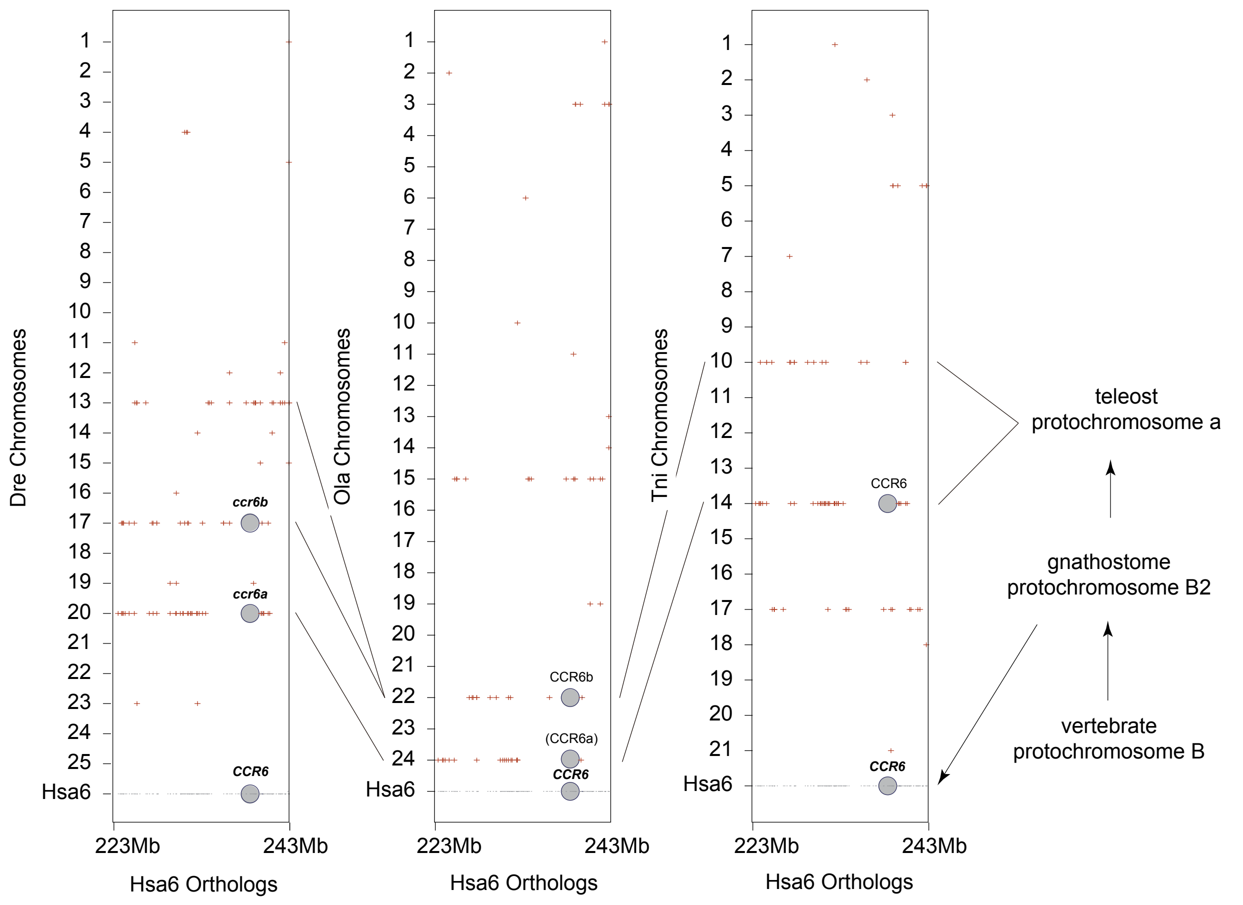

8) CCR7, CCR10

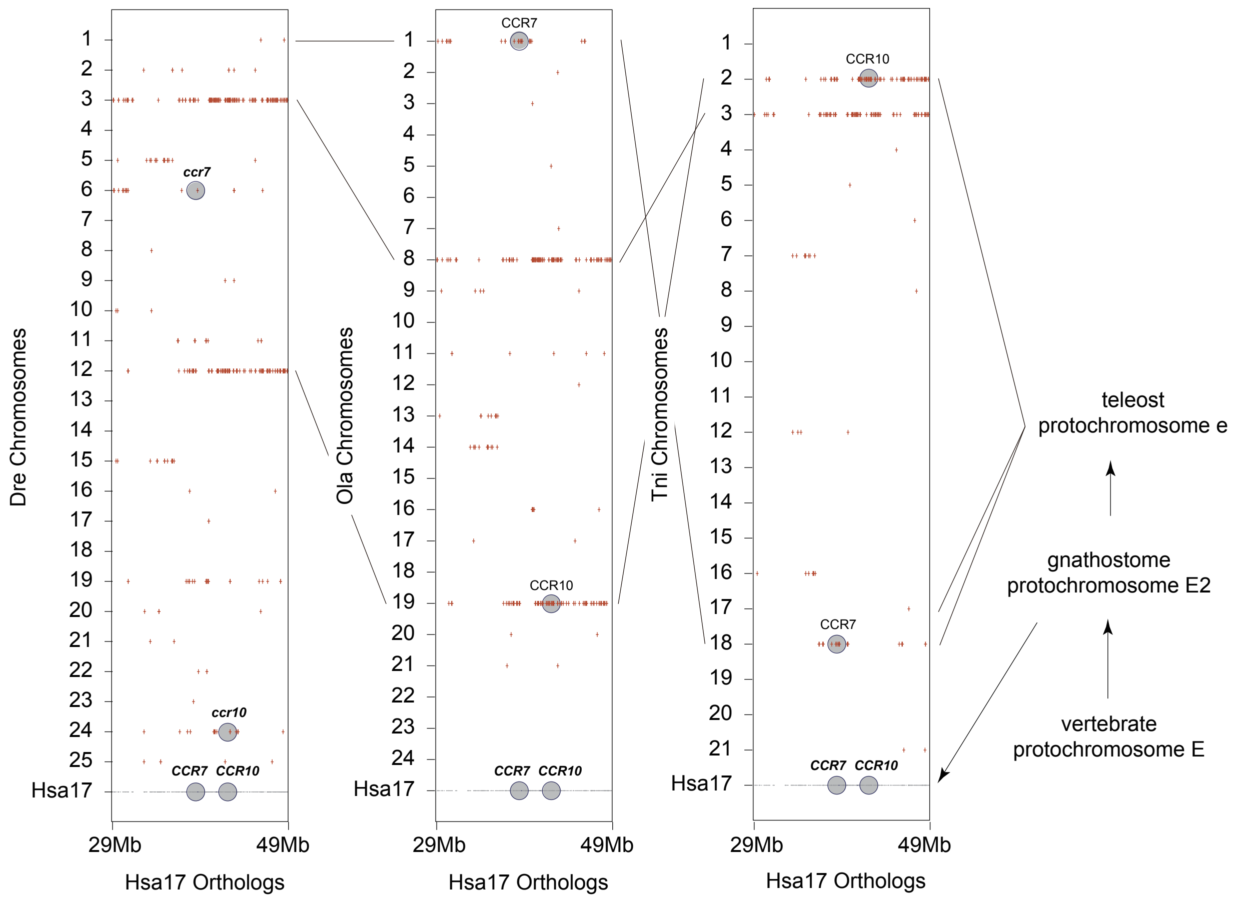

9) CCRL1

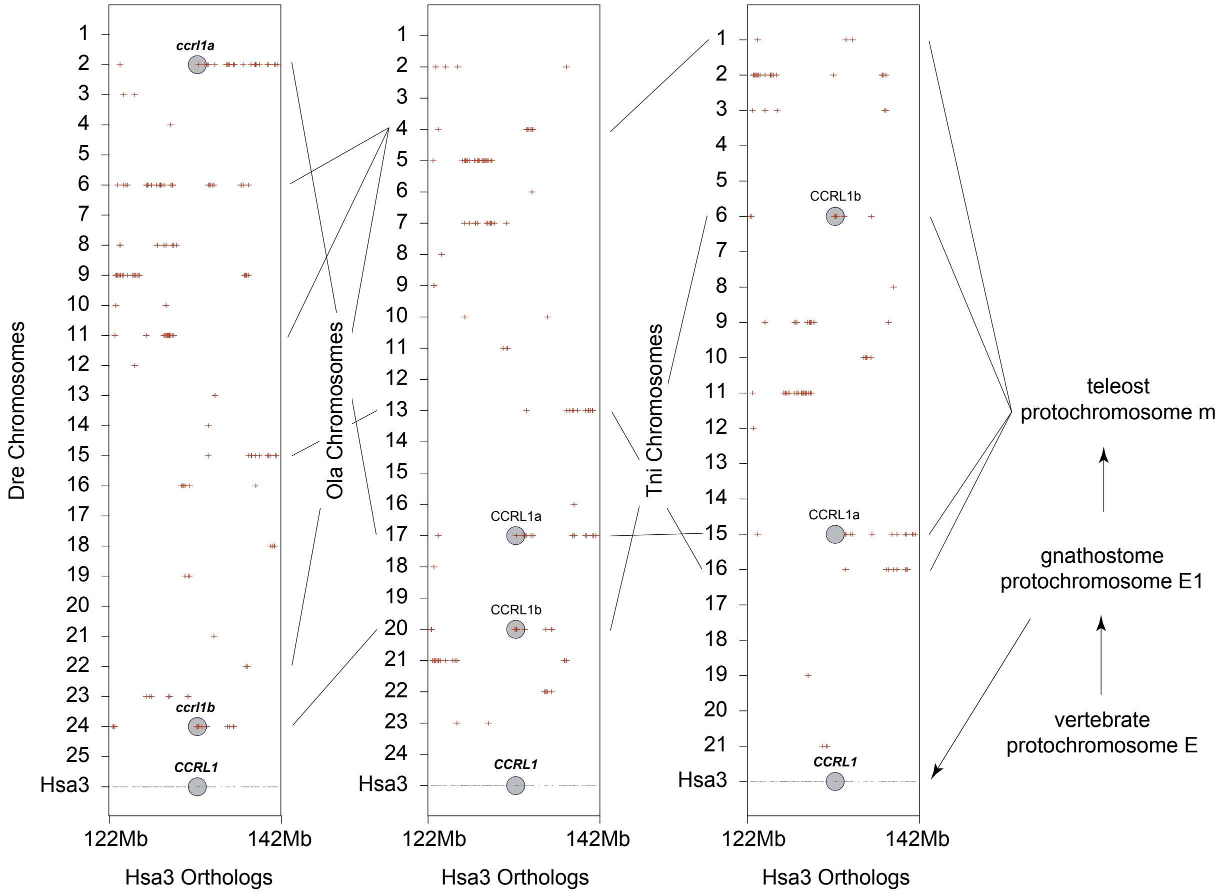

Dre Chromosomes
ccrl1a
ccrl1b
CCRL1
Hsa3
122Mb
142Mb
Hsa3 Orthologs
Ola Chromosomes
CCRL1a
CCRL1b
CCRL1
Hsa3
122Mb
142Mb
Hsa3 Orthologs
Tni Chromosomes
CCRL1b
CCRL1a
CCRL1
Hsa3
122Mb
142Mb
Hsa3 Orthologs
teleost
protochromosome m
gnathostome
protochromosome E1
vertebrate
protochromosome E
