# Supplementary material for: Systematic classification of vertebrate chemokines based on conserved synteny and evolutionary history
Source: Genes Cells. 2012 Nov 12;18(1):1–16. doi: 10.1111/gtc.12013 (PMC3568907; doi:10.1111/gtc.12013)
Supplement: Supplementary file 11 [file gtc0018-0001-SD6.pdf]

Fig. S6

Proposed ancestry of vertebrate chemokine and chemokine receptor genes.

Dotted boxes indicate hypothetical ancestral genes. Chromosome numbers in bold indicate chromosomes predicted to be derived from the same protochromosomes. HOX clusters are also shown together with chemokine receptors on page 4. However, human HOXA cluster is located on chromosome 7 and is not shown. 1R, 2R and 3R indicate the two successive rounds of WGD and the teleost-specific WGD, respectively.

A. Chemokine genes.

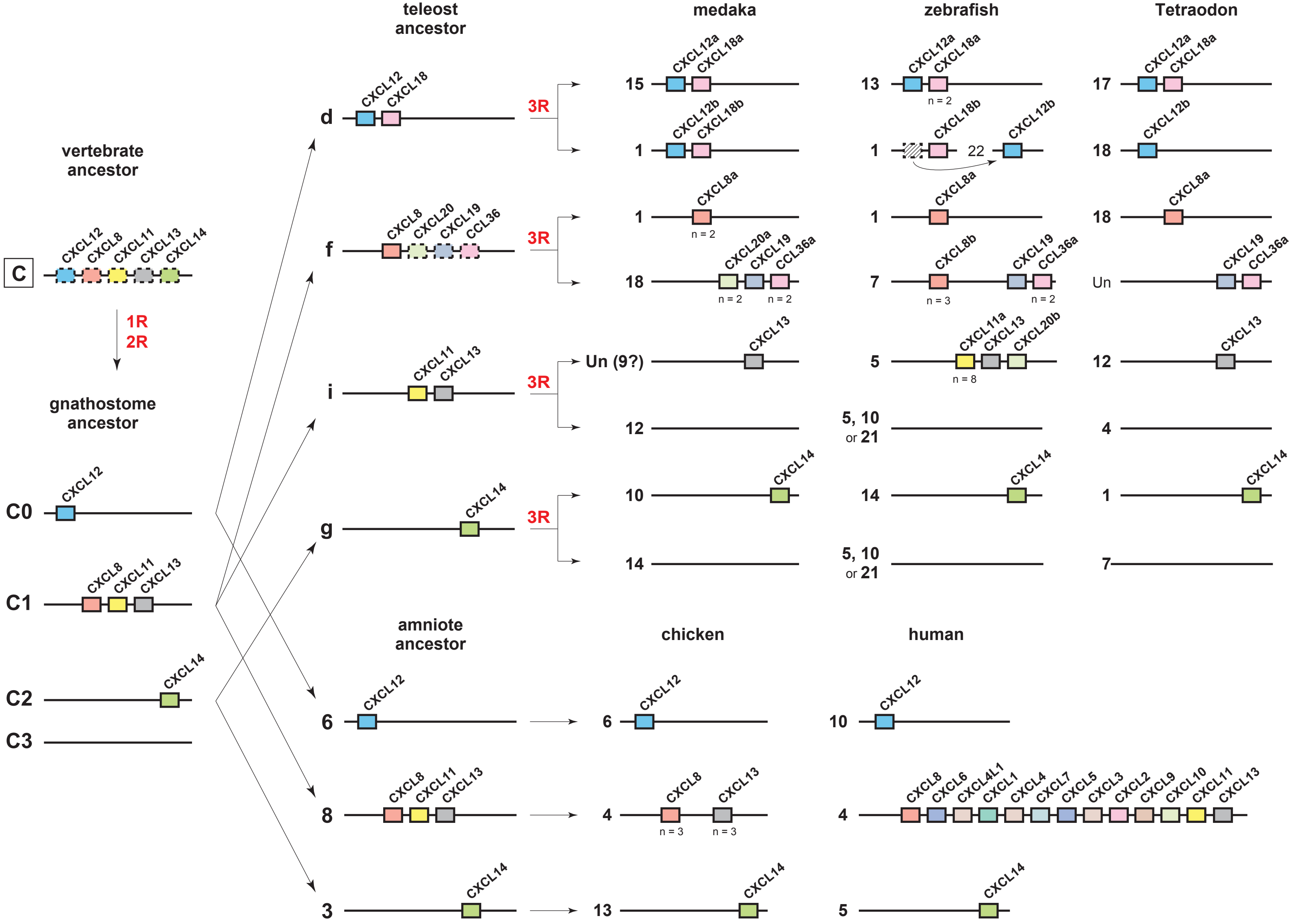

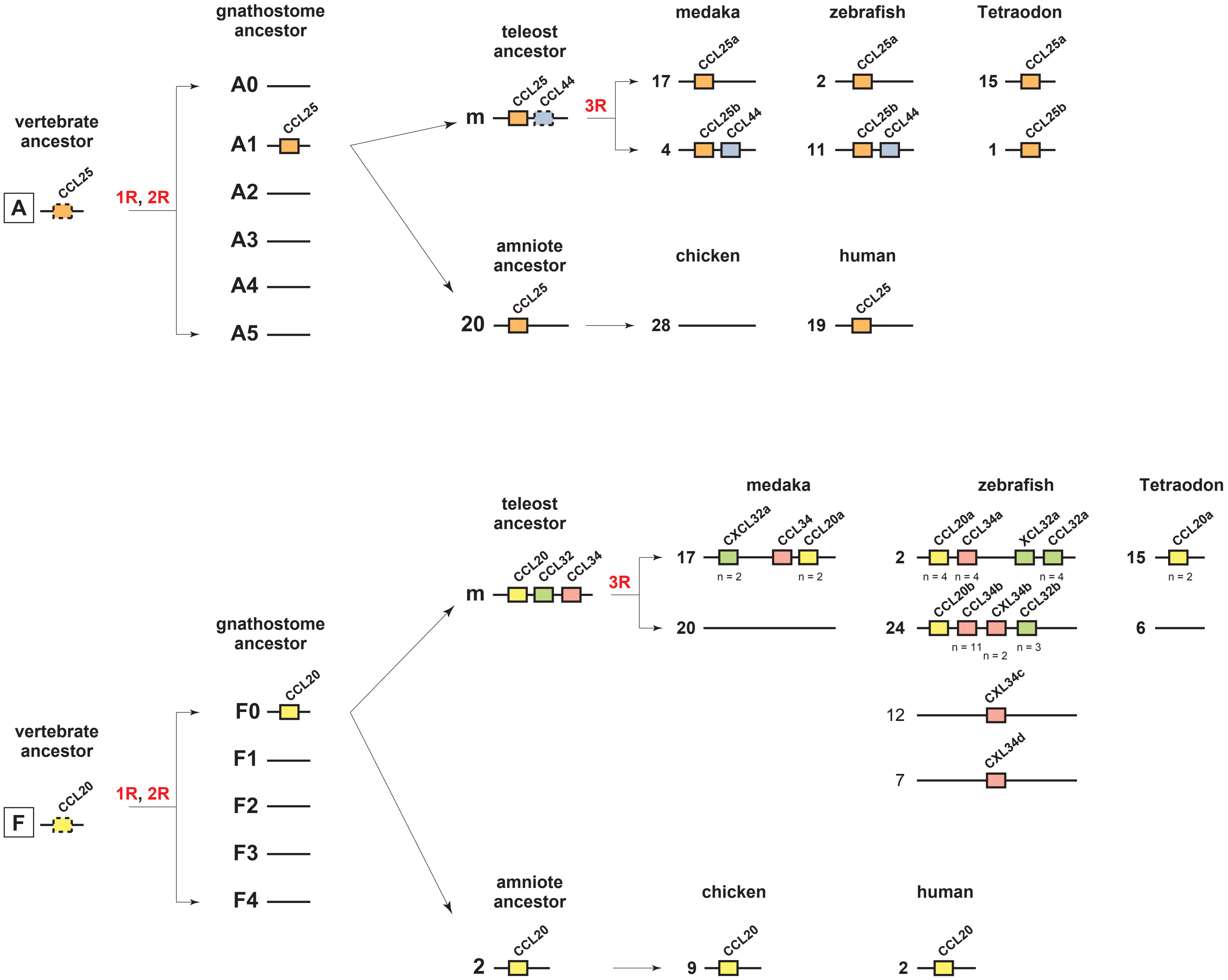

A
vertebrate ancestor
CCL25
1R, 2R
gnathostome ancestor
A0
A1
CCL25
A2
A3
A4
A5
teleost ancestor
m
CCL25
CCL44
3R
medaka
17
CCL25a
4
CCL25b
CCL44
zebrafish
2
CCL25a
11
CCL25b
CCL44
Tetraodon
15
CCL25a
1
CCL25b
amniote ancestor
20
CCL25
chicken
28
human
19
CCL25
F
vertebrate ancestor
CCL20
1R, 2R
gnathostome ancestor
F0
CCL20
F1
F2
F3
F4
teleost ancestor
m
CCL20
CCL32
CCL34
3R
medaka
17
CXCL32a
CCL34
CCL20a
n = 2
n = 2
20
zebrafish
2
CCL20a
CCL34a
XCL32a
CCL32a
n = 4
n = 4
n = 4
24
CCL20b
CCL34b
CXL34b
CCL32b
n = 11
n = 2
n = 3
12
CXL34c
7
CXL34d
Tetraodon
15
CCL20a
n = 2
6
amniote ancestor
2
CCL20
chicken
9
CCL20
human
2
CCL20

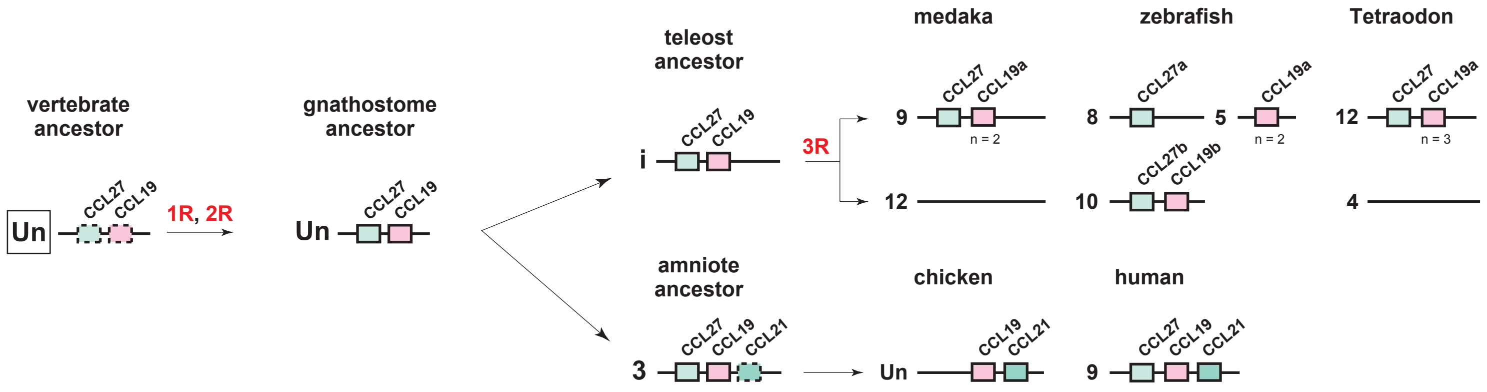
vertebrate ancestor
Un
CCL27
CCL19
1R, 2R
gnathostome ancestor
Un
CCL27
CCL19
teleost ancestor
i
CCL27
CCL19
3R
amniote ancestor
3
CCL27
CCL19
CCL21
medaka
9
CCL27
CCL19a
n = 2
12
zebrafish
8
CCL27a
5
CCL19a
n = 2
10
CCL27b
CCL19b
Tetraodon
12
CCL27
CCL19a
n = 3
4
chicken
Un
CCL19
CCL21
human
9
CCL27
CCL19
CCL21

B. Chemokine receptor genes.

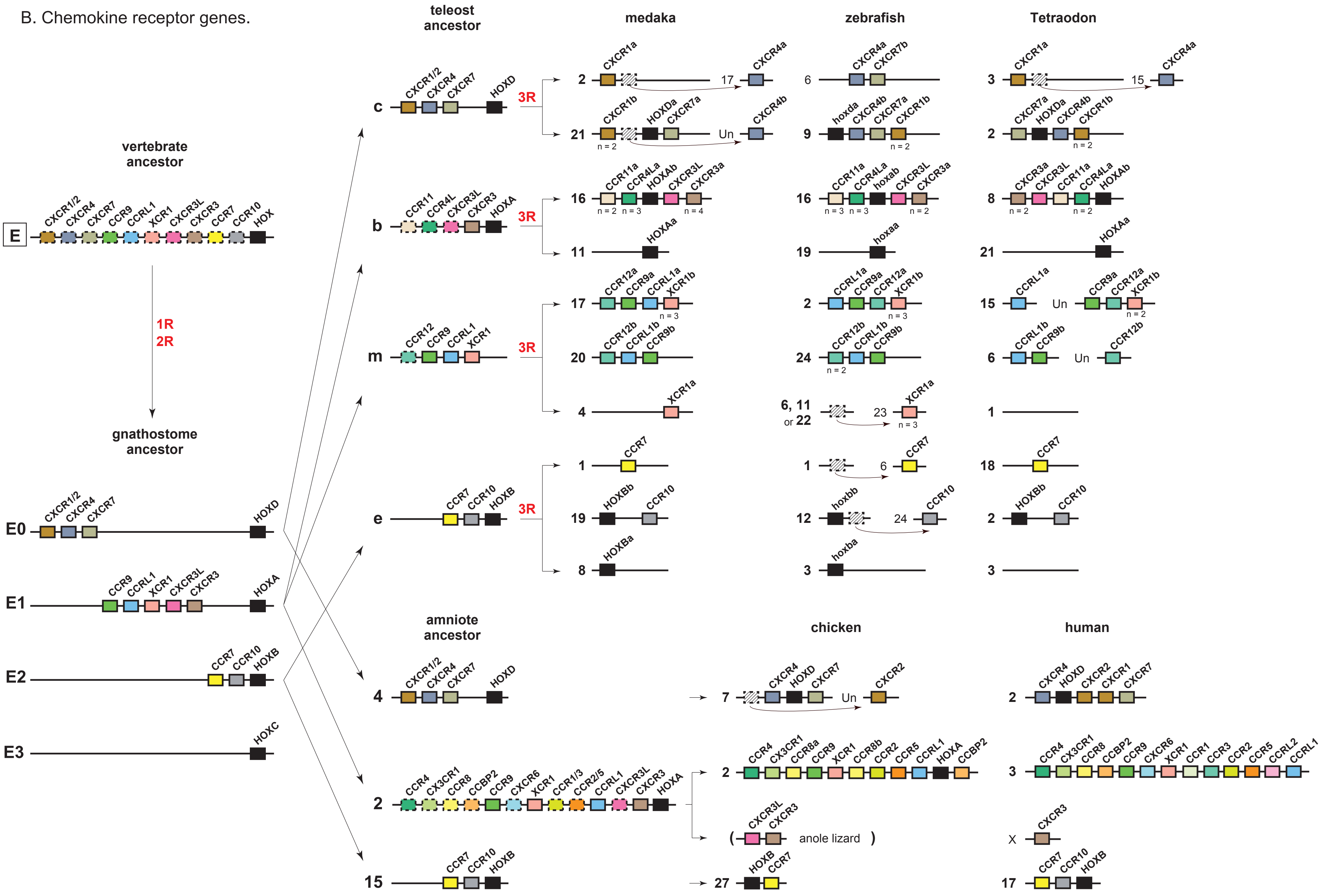

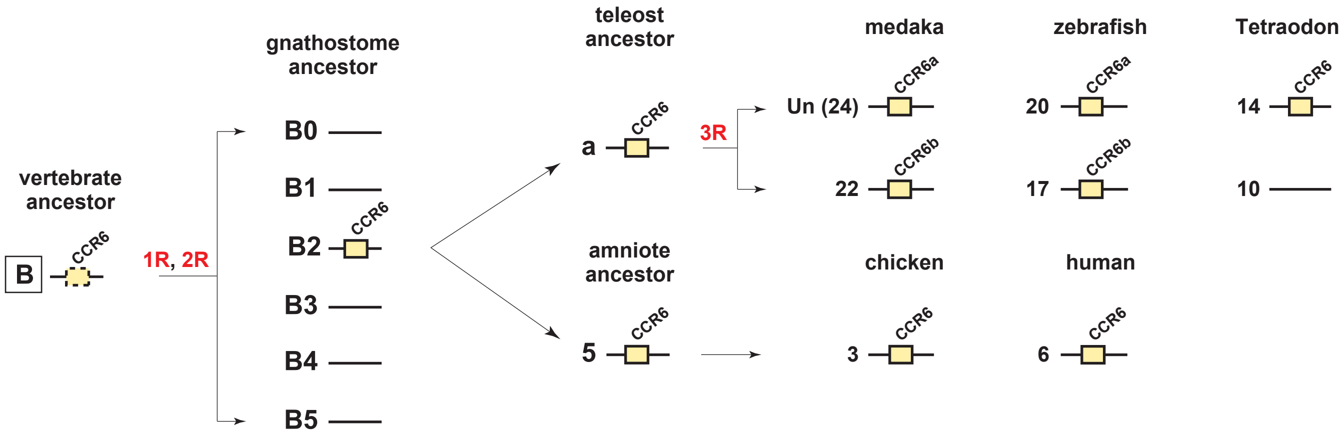
vertebrate ancestor
B
CCR6
1R, 2R
gnathostome ancestor
B0
B1
B2
CCR6
B3
B4
B5
teleost ancestor
a
CCR6
3R
amniote ancestor
5
CCR6
medaka
Un (24)
CCR6a
22
CCR6b
zebrafish
20
CCR6a
17
CCR6b
Tetraodon
14
CCR6
10
chicken
3
CCR6
human
6
CCR6

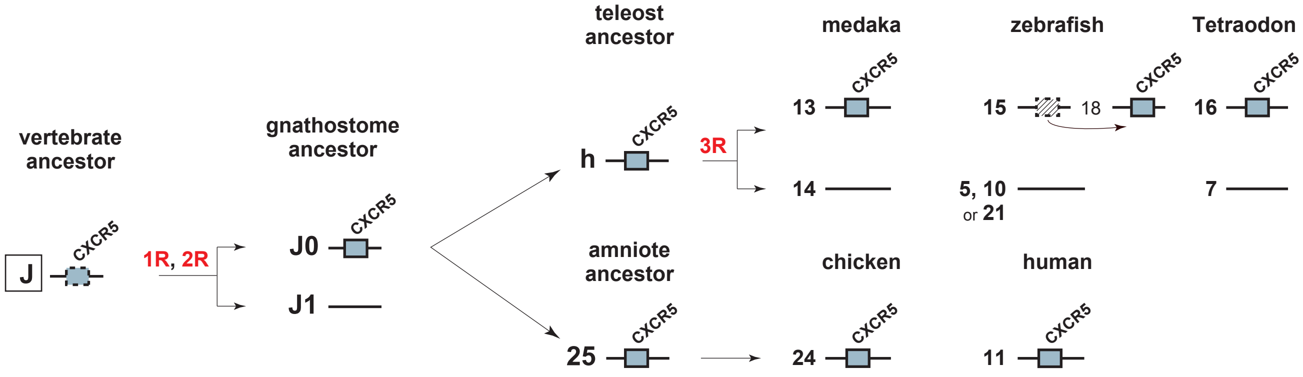
vertebrate ancestor
J
CXCR5
1R, 2R
gnathostome ancestor
J0
CXCR5
J1
teleost ancestor
h
CXCR5
3R
amniote ancestor
25
CXCR5
medaka
13
CXCR5
14
zebrafish
15
18
CXCR5
5, 10
or 21
Tetraodon
16
CXCR5
7
chicken
24
CXCR5
human
11
CXCR5
