# Supplementary material for: Systematic classification of vertebrate chemokines based on conserved synteny and evolutionary history
Source: Genes Cells. 2012 Nov 12;18(1):1–16. doi: 10.1111/gtc.12013 (PMC3568907; doi:10.1111/gtc.12013)

Fig. S7

Functional non-redundancy of the chemokine sysytem.

A. Cells are expressing two promiscuous receptors A and B, which shares some of their ligands. B. In this case, only non-promiscuous receptors C are expressed on the cells. Cross marks indicate the sites where chemokines are secreted.

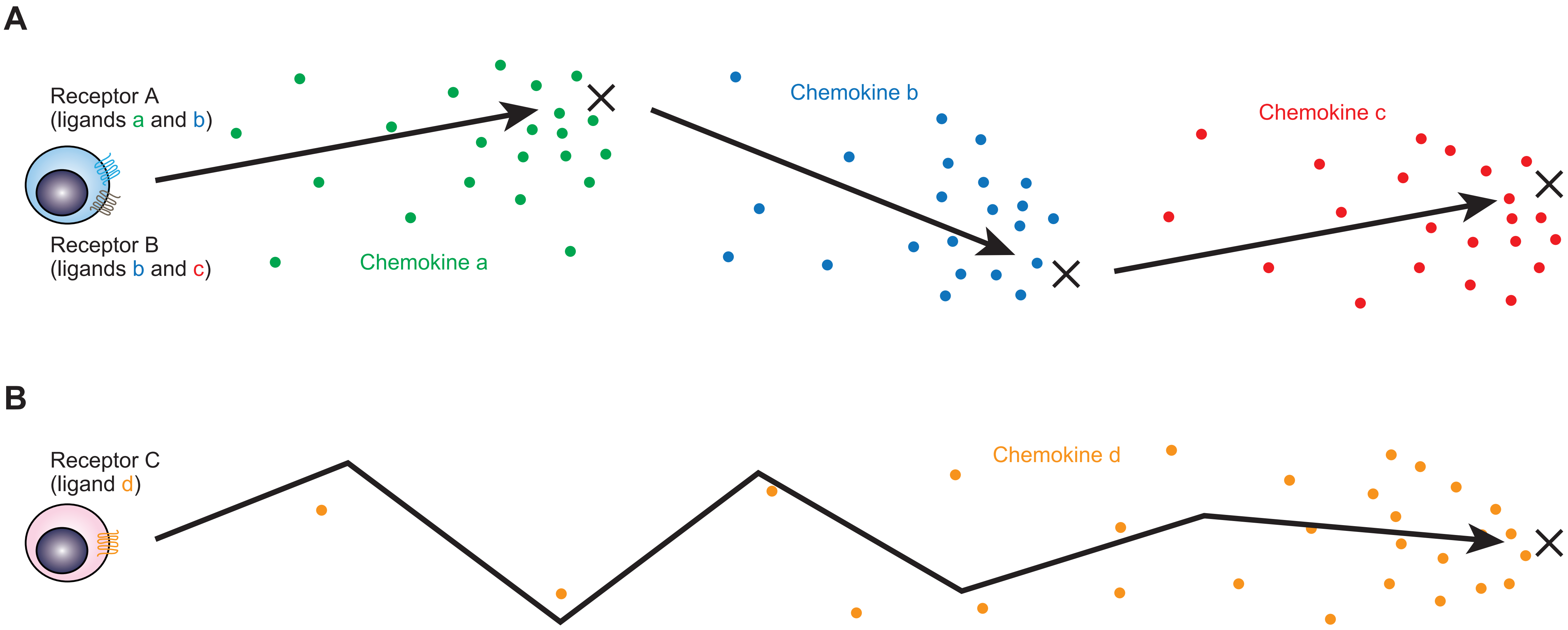

Supplement: Supplementary file 13 [file gtc0018-0001-SD7.pdf]
